# The transcriptomes, connections and development of submucosal neuron classes in the mouse small intestine

**DOI:** 10.1038/s41593-025-01962-x

**Published:** 2025-05-29

**Authors:** Wei Li, Khomgrit Morarach, Ziwei Liu, Sanghita Banerjee, Yanan Chen, Ashley L. Harb, Joel M. Kosareff, Charles R. Hall, Fernando López-Redondo, Elham Jalalvand, Suad H. Mohamed, Anastassia Mikhailova, David R. Linden, Ulrika Marklund

**Affiliations:** 1 Division of Molecular Neurobiology, Department of Medical Biochemistry and Biophysics, Karolinska Institutet, Stockholm, Sweden; 2 Enteric Neuroscience Program (ENSP), Mayo Clinic, Rochester, MN USA; 3 Department of Physiology and Biomedical Engineering, Mayo Clinic, Rochester, MN USA; 4 RIKEN Center for Integrative Medical Sciences, Yokohama, Japan

**Keywords:** Cell type diversity, Computational biology and bioinformatics, Differentiation, Enteric nervous system

## Abstract

The enteric submucosal plexus regulates essential digestive functions, yet its neuronal composition remains incompletely understood. We identified two putative secretomotor neuron classes and a previously unrecognized submucosal intrinsic primary afferent neuron class through single-cell RNA sequencing in the mouse small intestine. Using viral-mediated labeling of each class, we uncovered their morphologies and neural projections in the submucosa–mucosa context, finding connections among all classes and an unexpected close association with enterochromaffin cells. Further transcriptome analysis at the postnatal stage and lineage tracing revealed that neuron identities in the submucosal plexus emerge through an initial binary fate split at neurogenesis, followed by phenotypic diversification, akin to the developmental process of the myenteric plexus. We propose a unified developmental framework for neuronal diversification across the gut wall. Our study offers comprehensive molecular, developmental and morphological insights into submucosal neurons, opening new avenues for exploring physiological functions, circuit dynamics and formation of the submucosal plexus.

## Abstract (web summary)

The neuronal composition of the intestinal submucosal plexus is incompletely understood. Here Li et al. define their neuron classes, connectome and stepwise acquisition of identities.

## Main

The enteric nervous system (ENS) comprises the largest component of the peripheral nervous system and stands out for its ability to perform autonomous functions independently of the brain^1,2^. In most mammals, the intestinal ENS consists of the following two major concentric layers: the myenteric (Auerbach’s) plexus, located between the circular and longitudinal muscle layers, which drives peristalsis; and the submucosal plexus, near the mucosa, which is thought to mainly regulate luminal secretion and local blood flow^3,4^. Although myenteric neurons are better characterized, submucosal neurons were the first component of the ENS to ever be identified (by Georg Meissner in 1857 (ref. ^5^)). In guinea pigs, studies correlating neurotransmitter repertoire with physiological functions have categorized submucosal neuron types. These investigations suggested the presence of cholinergic (ACh^+^) and noncholinergic secretomotor neurons, both potentially contributing to vasodilation^4,6^. Based on major molecular markers, equivalents of secreto/vasomotor neurons have also been identified in the submucosa of mice^7^. However, while intrinsic primary afferent neurons (IPANs; sensory neurons) are present in the submucosa of guinea pigs^8^, immunohistochemical analysis in the mouse intestine suggested a lack of submucosal IPANs^7^. Likewise, combined electrophysiological and neurochemical analysis of mouse submucosa neurons failed to identify IPANs^9^. Nevertheless, advillin, a putative IPAN marker, is expressed in small intestine mucosa-projecting submucosal neurons^10^, and neurons with IPAN morphologies have been reported in the colonic submucosa^11^. To advance our understanding of submucosal neuron functions, development and roles in disease, the genetically tractable mouse model system is desirable to implement. Yet, full leverage of the model system requires a comprehensive molecular classification of mouse submucosal neurons.

Both submucosal and myenteric ENS originate from neural crest cells that invade the gut at early developmental time points and then migrate to populate the full extent of the gastrointestinal tract. The progenitor cells initially occupy the outer layer of the gut wall and differentiate into myenteric neurons and glia cells. At midgestational stages, progenitor cells migrate inwards toward the mucosa and differentiate into the submucosal plexus^12–14^. While myenteric neuron differentiation begins as early as embryonic day (E)10.5, the first neurons of the small intestine submucosa are observed 7 days later. We recently proposed a developmental model delineating a new principle by which myenteric neuron classes diversify, involving two major neurogenic differentiation paths that progress through phenotypic conversions^15^. However, the mechanism underlying the specification of neuronal subidentities from submucosal precursor cells remains unknown.

Single-cell RNA sequencing (scRNA-seq) enables unbiased identification and definition of neuronal classes. Myenteric neurons have been extensively characterized by scRNA-seq^15,16^ and single-nuclei RNA-seq^17,18^, yet isolated submucosal neurons lack specific interrogation. Here we conducted scRNA-seq on submucosal neurons in the small intestine of juvenile mice, identifying three cardinal submucosal (sm) enteric neuron classes (smENCs), including one class of IPANs (smENC1) and two presumed classes of secretomotor/vasodilatory neurons (smENC2 and smENC3). Viral-mediated labeling in transgenic mice specific to each neuronal class revealed their morphological features and intercellular proximities, suggesting extensive neuronal networks in the submucosa–mucosal space and unanticipated closeness to enterochromaffin cells of all three smENCs, but distance to arterioles. Transcriptionally equivalent myenteric neuron classes suggested shared developmental principles between the two ENS plexi. Through scRNA-seq at a postnatal stage combined with lineage tracing from an early neuronal state, we show that, similar to myenteric neurons, submucosal neurons develop through a stepwise process. Our submucosal single-cell transcriptome atlases, cellular connectivity map and delineation of fundamental developmental processes provide a solid foundation and new ideas for further interrogation of submucosal neuron formation, function and involvement in disease.

## Results

### Three submucosal neuron classes in the small intestine

**Fig. 1:**
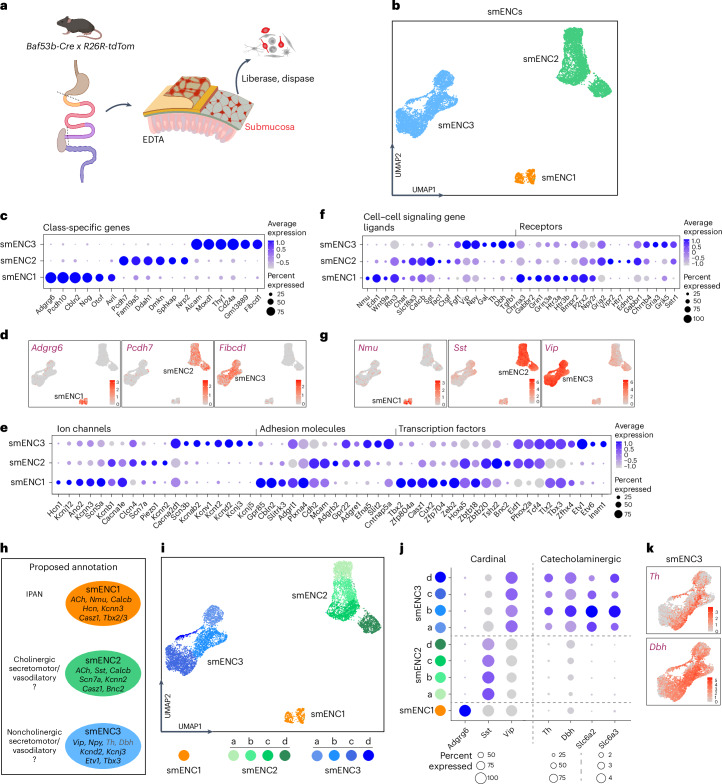
A molecular definition of neuron classes of the mouse small intestine submucosal plexus. **a**, Schematic representations of the experimental procedure indicating the dissection plane and dissociation procedure to obtain single submucosal neurons from the juvenile mouse small intestine. Part of **a** is created with BioRender.com. **b**, Uniform manifold approximation and projection (UMAP) of RNA-sequenced neurons, indicating three cardinal clusters. **c**–**g**, Dot plots and feature plots showing DE genes categorized as miscellaneous (**c**,**d**), ion channels, adhesion molecules, transcription factors (**e**) and cell–cell signaling genes (**f**,**g**). See Supplementary Fig. 2 for more comprehensive dot plots of genes with potential functional relevance. The color scale represents the *z* score, and the dot size represents the percentage of cells with nonzero expression within a given class. **h**, Proposed functional annotation of the submucosal neuron classes and selected marker genes. **i**, UMAP depicting refined clustering of smENC1–smENC3. **j**, Dot plot of cardinal and catecholaminergic gene expression in smENC subclusters, indicating no clear Th expression specificity. **k**, Feature plots depicting catecholaminergic marker genes in smENC3.

**Extended Data Fig. 1:**
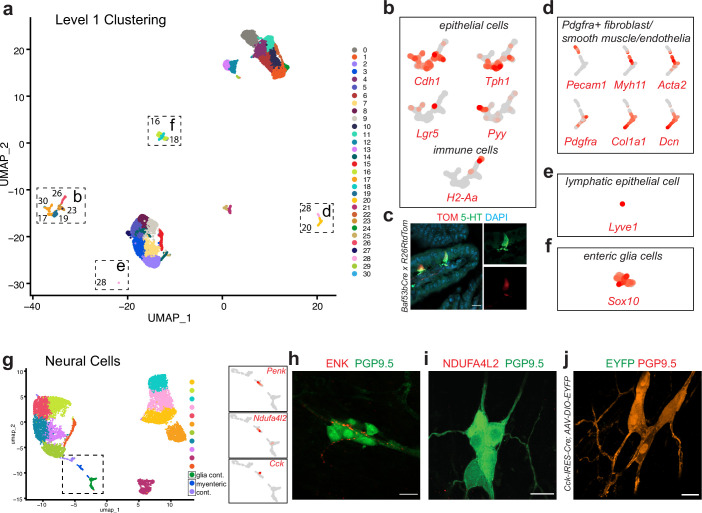
Identification of non-ENS cells, glial and plausible myenteric cells in the P24 submucosal scRNA-seq dataset. **a**, Uniform manifold approximation and projection (UMAP) of level 1 clustering of P24 submucosal cells. See Supplementary Table 1 for differentially expressed genes of the clusters. Jagged boxes are further analyzed in **b**–**f**. **b**, Feature plots indicating epithelial cells based on *Cdh1* and various subclass markers and immune-related cells (*H2-Aa*). **c**, Analysis of *Baf53b-Cre* x *R26RtdTom* mice revealed TOM^+^/5-HT^+^ double-positive cells, showing transgene expression in serotonergic enterochromaffin cells. Image represents observations from 10 fields of view from 2 mice. Feature plots displaying marker genes of clusters representing mesenchymal-derived lineages, including smooth muscle cells (**d**) and lymphatic epithelial cells (LECs; **e**). **f**, Feature plot of *Sox10* indicating an enteric glia cell cluster. **g**, UMAP of cluster level 2, displaying putative contaminating glial and myenteric classes as indicated in feature plots. **h**,**i**, Representative submucosa peels showing lack or little ENK (**h**) and NDUFA4L2 (**i**) expression in PGP9.5^+^ neurons. 0/2583 neurons expressed ENK; n = 3 mice. 2/7615 neurons expressed NDUFA4L2; n = 5 mice. **j**, CCK expression was investigated in *Cck-IRES-Cre* mice injected with AAV-DIO-EYFP or AAV-DIO-tdTOM. Only 1 of 2353 submucosal neurons expressed a fluorescent reporter; n = 4 mice (age, 8–12 weeks). Scale bars, 20 µm.

To achieve an unbiased view of the neuronal diversity within the submucosal plexus in mice, we aimed to conduct scRNA-seq. While we previously have isolated and characterized myenteric neurons by scRNA-seq^15,16^, submucosal neurons presented a challenge to isolate due to their small ganglia situated between the muscularis and mucosal layer. Consistent with myenteric neurons, we observed that submucosal neurons (PGP9.5^+^), but not enteric glia (SOX10^+^), showed selective reporter expression in juvenile *Baf53b-Cre;R26RtdTom* mice (Supplementary Fig. 1a–c). An optimized method for submucosal neuron isolation was developed using flow cytometry capture of fluorescent cells (~0.7% of sorted cells) from mucosa–submucosal small intestine peels of juvenile *Baf53b-Cre;R26tdTom* mice (Fig. 1a). Initial analysis of the resulting scRNA-seq dataset identified clear neural clusters, as well as smaller non-ENS clusters, including epithelial and mesenchymal cells (Extended Data Fig. 1a,b and Supplementary Table 1). Analysis of *Baf53b-Cre;R26RtdTom* gut tissue demonstrated TOM^+^/5-HT^+^ enterochromaffin cells (Extended Data Fig. 1c), while the remaining TOM^+^ cells appeared neuronal; thus, the profiles of blood, muscle and lymphatic cells (Extended Data Fig. 1d,e) probably resulted from faulty inclusion during flow cytometry. After excluding non-ENS cells, small clusters with glial or myenteric neuron character still remained (Extended Data Fig. 1f–j), which were removed to focus on 8,341 cells with unambiguous submucosal identities.

**Extended Data Fig. 2:**
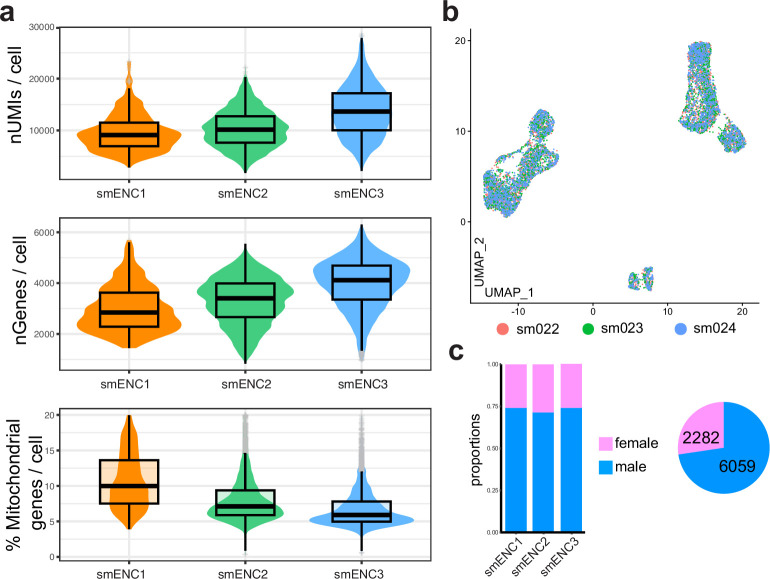
Quality control of final submucosal neuron scRNA-seq dataset. **a**, Box plots showing number of UMIs, detected genes and percentage of mitochondrial genes per cell for each of the cardinal smENCs. Box-and-whisker plots indicate max–min (whiskers), 25–75 percentile (boxes) with median as a center line. smENC1, 503 cells; smENC2, 3770 cells; smENC3, 4068 cells. **b**, Feature plot depicting cells from the three libraries distributed evenly over the clusters. Note, the same single-cell suspension was used for all libraries. **c**, Pie chart depicting the number of cells of each sex, and a bar graph verifying presence of female and male cells in all clusters, and their similar ratios between clusters. UMI, unique molecular identifier; smENC, submucosal enteric neuron class.

We applied different clustering resolutions and determined that the submucosal neurons consist of three cardinal classes, which we denoted smENC1–smENC3 (Fig. 1b and Extended Data Fig. 2a–c). smENC1–smENC3 exhibited markedly different and comprehensive transcriptional profiles (Supplementary Table 2 and Supplementary Fig. 2). Curiously, many of the genes with the most distinct expression have no known role in submucosal neurons, such as the G-protein-coupled receptor *Adgrg6* in smENC1, cadherin protein *Pcdh7* in smENC2 and acetyl-group-binding transmembrane protein *Fibcd1* in smENC3 (Fig. 1c,d). Genes important to conferring neural-specific activity patterns, including membrane trafficking genes, Ca^2+^-regulators and ion channels, displayed unique patterns (Fig. 1e and Supplementary Fig. 2a,b). All types of ion channels were differentially expressed (DE) among smENCs, with smENC3 showing a particularly extensive set of ion channels, including *Scn3b*, *Kcnd2*, *Cacna2d1* and *Kcnj5*. All three smENCs expressed *Calb2* (calretinin), none expressed *Calb1* (calbindin), while only smENC3 expressed *Scgn* (Supplementary Fig. 2a). The most DE adhesion molecules included *Gpr85*, *Adgrb2* and *Slit2* for smENC1, smENC2 and smENC3, respectively (Fig. 1e and Supplementary Fig. 2c). Selectively expressed transcription factors included *Tbx2* in smENC1, *Bnc2* in smENC2 and *Etv1* in smENC3 (Fig. 1e and Supplementary Fig. 2d). A large diversity of ligands and receptors were selectively or combinatorially expressed (Fig. 1f,g and Supplementary Fig. 2e,f). Of note, smENC1 and smENC2 were identified as cholinergic (*Chat* and *Slc18a3*) and CGRP^+^ (*Calcb*), with smENC1 additionally expressing *Nmu* and *Edn1* and smENC2 expressing *Sst* and *Tac1*. In contrast, smENC3 was noncholinergic and characterized by *Vip*, *Npy* and *Th*/*Dbh*. A wide range of receptors were identified, indicating responsiveness of smENC1 to glutamate (*Grin1* and *Grin3a*) and BMP/Activins (*Bmpr2* and *Acvr1*/*Acvr2*), smENC2 to vasoactive intestinal peptide (*Vipr2*) and endothelin (*Ednrb*) and smENC3 to somatostatin (*Sstr1*) and glutamate (*Gria2*, *Gria3* and *Gria4*). All smENCs appear responsive to ACh (for example, *Chrna3*, *Chrnb4*, *Chrm1* and *Chrm2*) and are capable of purinergic signaling (for example, *Adora1*, *P2rx2* and/or *P2ry6*). smENC1 and smENC3 may be responsive to noradrenaline (*Adra2a*). For a more comprehensive overview of gene categories likely relevant to the function of each smENC, please view Supplementary Fig. 2.

### Two putative secretomotor/vasodilatory classes and one IPAN

Prior immunohistochemical characterization of mouse submucosal neurons^7^ alongside comparative investigations of guinea pigs recognized that submucosal ganglia mainly consist of cholinergic (ChAT, CGRP, SST and CALR) and noncholinergic (VIP, NPY and CALR) secretomotor neurons. These neurons likely correspond to smENC2 and smENC3, respectively (Fig. 1h). Additionally, cholinergic neurons lacking secretomotor or IPAN characteristics were identified^7^, constituting 10% of the neurons. We surmise that smENC1 corresponds to these nonidentifiable neurons, but in contrast to the previous analysis, we propose that they are submucosal IPANs, as their transcriptional profile showed significant similarities to myenteric IPANs (myENC6; ref. ^15^), including *Nmu*, *Adgrg6* and *Syt15* (Supplementary Table 2, Fig. 1h and Supplementary Fig. 3).

**Extended Data Fig. 3:**
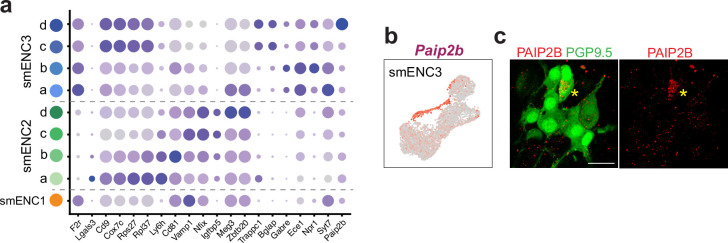
Differential gene expression in submucosal neuron subclusters. Supportive data related to Fig. 1i-k. **a**, Dot plot displaying examples of most differentially expressed genes in submucosal neuron subclusters. **b**, Feature plot showing preferential expression of PAIP2B in smENC3d. **c**, Validation of PAIP2B protein expression in a submucosal PGP9.5^+^ neuron (indicated with a star) in duodenum at P28. Image represents observations from 9 fields of view from 5 mice. Scale bar, 20 µm.

The distinction between noncholinergic secretomotor and secretomotor/vasodilatory neurons has been proposed to correlate with tyrosine hydroxylase (*Th*) expression^7^. To investigate whether *Th* expression marks a distinct subcluster within smENC3, we performed finer resolution clustering, revealing four subclusters each of smENC2 and smENC3 (denoted a–d), while smENC1 remained as one cluster (Fig. 1i, Extended Data Fig. 3a and Supplementary Table 3). *Th* correlated with other catecholaminergic genes and was most evidently expressed in smENC3b and smENC3d, although not exclusively (Fig. 1j,k). Thus, the catecholaminergic profile did not align with distinct subcluster(s) of smENC3. Further analysis revealed few uniquely expressed subcluster genes, yet several genes exhibited graded expression across the subclusters (for example, *Cd9* and *Zbtb20*; Extended Data Fig. 3a). *Paip2b* was more clearly enriched in smENC3d (Extended Data Fig. 3a,b), and we confirmed PAIP2B by immunohistochemistry in submucosal neurons primarily located in anterior small intestine (Extended Data Fig. 3c).

### smENCs are transcriptionally similar to myenteric ENCs

**Fig. 2:**
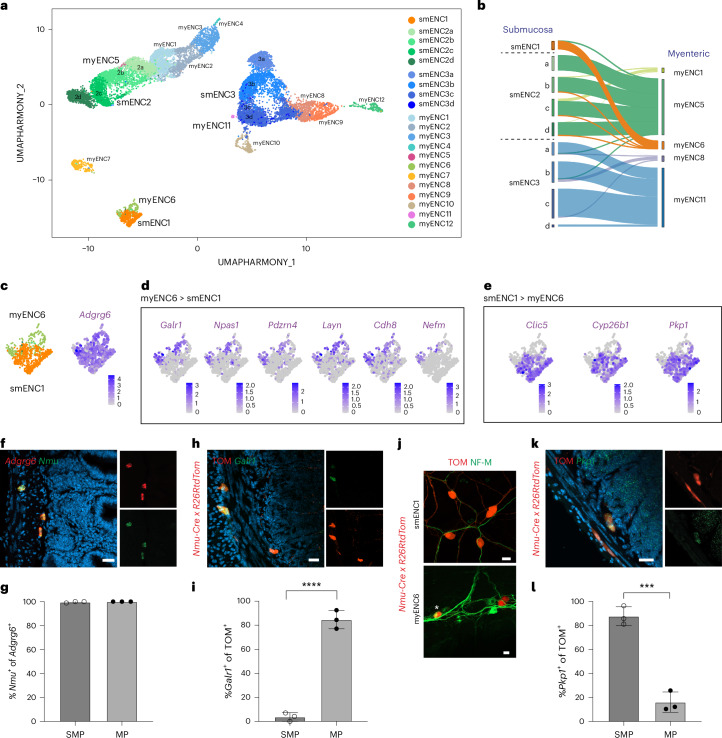
Submucosal neuron classes show transcriptional equivalence to three myenteric neuron classes. **a**, UMAP of Harmony-integrated myenteric and submucosal juvenile cells, labeled with their cluster identities defined in ref. ^15^ and in this study (Fig. 1i). **b**, Sankey plot indicating molecular similarity between transcriptionally similar pairs of smENCs and myENCs. **c**, Feature plot showing robust *Adgrg6* expression in both smENC1 and myENC6. **d**, Feature plot assembly displaying genes with more definitive expression in myENC6 than smENC1. **e**, Feature plot assembly displaying genes with more definitive expression in smENC1 than myENC6. **f**,**g**, Representative transverse sections (**f**) and graph statistical analysis (**g**) showing correlation between *Adgrg6* and *Nmu* expression identified by RNAscope. Number of cells analyzed—195 submucosal *Adgrg6*^*+*^ neurons; 239 myenteric *Adgrg6*^*+*^ neurons (*n* = 3 mice). **h**,**i**, Representative transverse section (**h**) and graph statistical analysis (**i**) showing *Galr1* in myENC6 but not smENC1 (*P* < 0.0001). Number of cells analyzed—239 tdTom^+^ submucosal neurons and 222 tdTom^+^ myenteric neurons (*n* = 3 mice). **j**, Immunohistochemistry showing expression of NF-M in myENC6 (indicated by star) but not smENC1 (0 of 1485 tdTom^+^ neurons, *n* = 3 mice). **k**,**l**, Representative transverse section (**k**) and graph statistical analysis (**l**) showing expression of *Pkp1* preferentially in smENC1 over myENC6 (*P* = 0.0004). Number of cells analyzed—220 tdTom^+^ submucosal neurons and 182 tdTom^+^ myenteric neurons (*n* = 3 mice). Two-tailed Student’s *t* test was performed to determine statistical significance. Data in **g**, **i** and **l** are presented as mean ± s.d. Each dot (*n*) represents one animal. ***P < 0.001 and ****P < 0.0001. Scale bars, 20 µm (**f**,**h**,**j**,**k**). Mice age = 8–12 weeks. SMP, submucosal plexus; MP, myenteric plexus. Source data

smENC1 showed clear transcriptional resemblance to myENC6, but gene expression parallels were also evident between smENC2, smENC3 and certain myenteric neuronal classes (Supplementary Fig. 3a–c). Marker genes such as *Sst*, *Vipr2* and *Avpr1a* were expressed in smENC2 and myENC5, while *Vip*, *Scgn* and *Prokr1* were expressed in smENC3 and myENC11. To further validate and explore these inferred similarities, the submucosal scRNA-seq dataset was integrated with our published myenteric scRNA-seq dataset^15^ (Fig. 2a). Indeed, smENC1 and myENC6 were found to segregate together, myENC5 aligned with smENC2 and myENC11 juxtaposed smENC3. Additionally, label transfer analysis confirmed the similarities between the myenteric–submucosa pairs (Fig. 2b).

While presumed IPANs in the myenteric (myENC6) and submucosal (smENC1) plexi show transcriptional similarities, they reside in distinct gut compartments and may differ in their specific functions, which should be reflected in their gene expression profiles. Screening for differences, we found that *Galr1*, *Npas1*, *Cdh8* and *Nefm* predominantly expressed in myENC6 (Fig. 2c,d), while *Clic5*, *Cyp26b1* and *Pkp1* associated with smENC1 (Fig. 2c,e). To assess the validity of Nmu as a marker for smENC1, we performed RNAscope with the transcriptionally more robust smENC1/myENC6 marker *Adgrg6* (Fig. 2c). We found that nearly all *Adgrg6*^+^ neurons co-expressed *Nmu* in both plexi (Fig. 2f,g). Therefore, we concluded that *Nmu-Cre* mice, previously used to examine myENC6, also serve as a suitable transgenic tool for smENC1. Using *Nmu-Cre;R26RtdTom* mice, we confirmed the exclusive expression of *Galr1* in myENC6 (Fig. 2h,i). Furthermore, neurofilament medium chain (NF-M*; Nfem*), previously detected in 63% of myENC6 (ref. ^15^), was not found in smENC1 (Fig. 2j). The absence of NF-M expression in smENC1 could explain unsuccessful attempts to identify IPANs in the mouse submucosa, as it was considered an IPAN marker^7^. We furthermore confirmed that most smENC1 but only a small subset of myENC6 expressed *Pkp1* (Fig. 2k,l). Thus, we identify several markers that could be used to distinguish myenteric and submucosal IPANs, while *Adgrg6* and *Nmu* are reliable markers for small intestine IPANs.

### Class morphologies and proportions across the small intestine

**Fig. 3:**
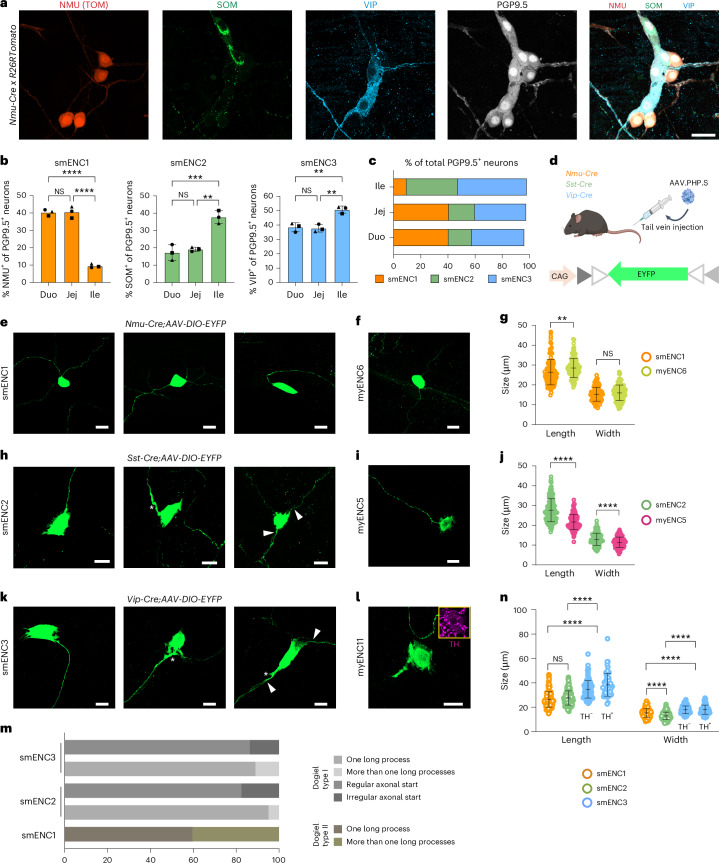
Submucosal neuron class morphologies and distribution across the small intestine. **a**, Representative confocal images of the three cardinal smENCs in small intestine regions. TOM, SOM, VIP and PGP9.5 were used to identify smENC1, smENC2, smENC3 and all neurons, respectively, in adult *Nmu-Cre* x *R26RtdTom* mice. See Supplementary Fig. 4a–c for representative pictures of all regions. **b**, Graphs showing the proportion of smENCs in small intestine regions (*n* = 3 mice). Data are presented as mean ± s.d. Each dot (*n*) represents one animal (NMU—duo versus jej, *P* = 0.9917; duo versus ile, *P* < 0.0001; jej versus ile, *P* < 0.0001; SOM—duo versus, *P* = 0.7934; duo versus ile, *P* = 0.0008). **c**, Stacked bar graph showing the mean proportion of the smENCs in the duodenum, jejunum and ileum. **d**, Schematic drawing of viral-mediated labeling of submucosal classes. Panel **d** is created with BioRender.com. **e**,**f**, Representative pictures displaying the similar morphologies of smENC1 (**e**) and myENC6 (**f**). **g**, Graph showing soma size comparison between smENC1 and myENC6. Number of cells analyzed in five mice—125 smENC1 neurons (length/width—26.37 ± 6.35/15.26 ± 3.50), 115 myENC6 neurons (length/width—28.54 ± 4.83/15.99 ± 3.94; length—*P* = 0.0034; width—*P* = 0.1274). **h**,**i**, Representative pictures displaying the morphologies of smENC2 (**h**) and myENC5 (**i**). **j**, Graph showing the soma size comparison between smENC2 and myENC5. Number of cells analyzed in five mice—143 smENC2 (length/width—27.64 ± 5.80/12.98 ± 3.08), 127 myENC5 (length/width—21.67 ± 3.83/11.48 ± 2.60; length—*P* < 0.0001; width—*P* < 0.0001). **k**, Representative pictures showing the morphology of smENC3. **l**, Picture showing one VIP/TH^+^ myENC11 neuron. TH expression excluded other VIP^+^ myenteric neurons, including myENC8–myENC10. **m**, Graph summarizing the smENC morphological types. Number of cells analyzed—62 smENC1 neurons in three mice; 102 smENC2 neurons in three mice; 73 smENC3 neurons in three mice. **n**, Graph depicting the size comparison between the three smENCs. Number of cells analyzed as indicated above and in four mice—99 smENC3TH^−^ neurons (length/width—34.68 ± 7.13/17.95 ± 3.09), 64 smENC3TH^+^ neurons (length/width—38.35 ± 9.24/18.02 ± 3.81; length, smENC1 versus smENC2—*P* = 0.4422; smENC1 versus smENC3—*P* < 0.0001; smENC2 versus smENC3—*P* < 0.0001; width, smENC1 versus smENC2—*P* < 0.0001; smENC1 versus smENC3—*P* < 0.0001; smENC2 versus smENC3—*P* < 0.0001). Graphical data in **g**, **j** and **n** are presented as mean ± s.d. Each circle represents one cell. Two-tailed Student’s *t* test (**g**,**j**) and one-way ANOVA with Tukey’s multiple-comparison test (**b**,**n**) were used to determine the statistical significance; **P < 0.01; ***P < 0.001 and ****P < 0.0001. All images show submucosal or myenteric peels. Arrowheads indicate two axons emanating from one soma. Stars (**h**,**k**) indicate irregular axonal start. Scale bars, (**a**) 30 µm and (**e**,**f**,**h**,**i**,**k**,**l**) 20 µm. Mice age = 8–12 weeks. Duo, duodenum; Jej, jejunum; Ile, ileum; ANOVA, analysis of variance; NS, not significant. Source data

We have unveiled three cardinal submucosal clusters, signified by *Nmu* (smENC1), *Sst* (smENC2) and *Vip* (smENC3). To validate the three clusters in vivo and determine their distribution across the small intestine, we performed immunohistochemistry for SOM, VIP and pan-neuronal PGP9.5 in adult *Nmu-Cre;R26RtdTom* peels. NMU-TOM, SOM and VIP rarely colocalized within the same neuron (0,09% neurons co-expressed >1 marker); thus, they are specific smENC1–smENC3 markers (Fig. 3a–c and Supplementary Fig. 4a–c). Notably, cells expressing either NMU-TOM, VIP or SOM constituted nearly all PGP9.5^+^ neurons (96–98%; Fig. 3c), suggesting the essentially complete classification of submucosal neurons by smENC1–smENC3. Although all regions contained the three neuron classes, the proportion of smENC1 was higher in the duodenum and jejunum (∼40%) than in the ileum (∼10%; Fig. 3b,c). Conversely, a complementary pattern was observed among smENC2 neurons (duodenum/jejunum, ∼18% and ileum, ∼38%) and smENC3 neurons (duodenum/jejunum, ∼38% and ileum, ∼51%).

We next acquired *Sst-Cre* and *Vip-Cre* mouse lines to target smENC2 and smENC3 selectively (see validation in Supplementary Fig. 4d–g), while *Nmu-Cre* mice were chosen for smENC1 (Fig. 2f,g). To elucidate the cellular morphology of the three smENCs and compare it with their transcriptionally equivalent myENCs, the class-specific Cre mice were administered adeno-associated virus (AAV.PHP.S) carrying Cre-inducible yellow fluorescent protein (DIO-EYFP; Fig. 3d), and the soma size was assessed in the three Cre lines crossed with *R26RtdTom*. smENC1 neurons displayed a Dogiel type II morphology (smooth cell body), consistent with myENC6 neurons (Fig. 3e,f and Supplementary Fig. 4h)^15^. smENC1s were slightly smaller than myENC6s (Fig. 3g), and 40% displayed more than one long process (Fig. 3e,m). smENC2 exhibited a Dogiel type I morphology (lamellar dendrites; Fig. 3h,m) and were larger than myENC5 (Fig. 3i,j and Supplementary Fig. 4i). Similarly, smENC3 displayed Dogiel type I morphology, in agreement with myENC11 (Fig. 3k–m and Supplementary Fig. 4j). A proportion of both smENC2 and smENC3 had two long processes, and some displayed irregularities at the presumed axonal start (Fig. 3h,k and Supplementary Fig. 4i,j), consistent with previous studies of secretomotor neurons in guinea pigs^6^. Among the submucosal neuron classes, the soma of smENC3 (both TH^+^ and TH^−^) was the largest considering both length and width (Fig. 3n).

Taken together, while smENC1–smENC3 are found in the entire small intestine, they distribute unevenly. The transcriptionally similar neuron pairs in the myenteric and submucosal plexus displayed similar morphologies, but differences in their soma size were observed.

### Response patterns of smENC1 suggest an IPAN identity

**Extended Data Fig. 4:**
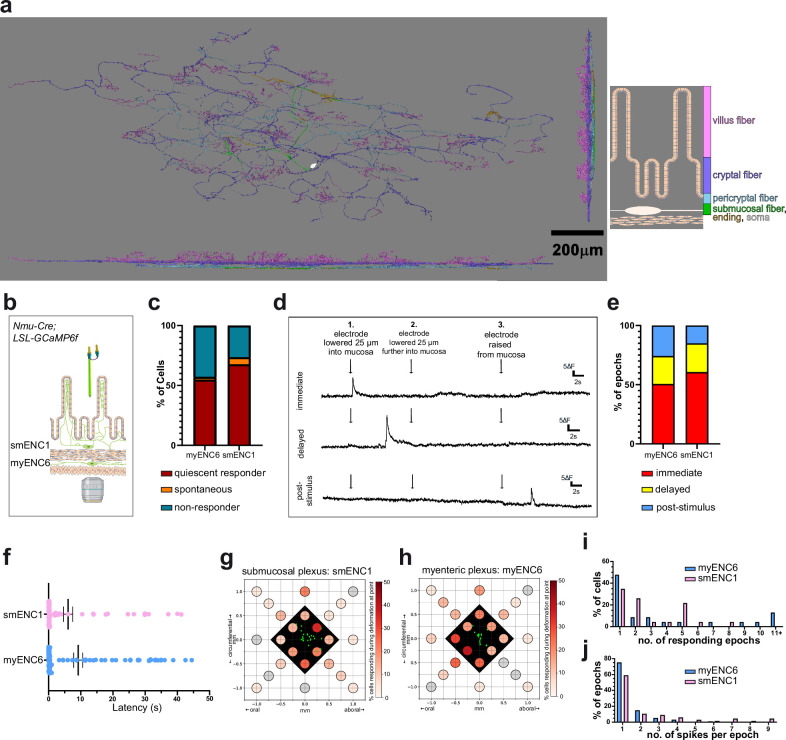
Morphological and functional interrogation of smENC1 suggests an IPAN identity. **a**, 3D reconstructed image showing one smENC1 neuron from a male mouse (32 weeks), with color-coded fibers within the submucosal plane (endings and fibers), the pericryptal and cryptal spaces and villi. Video representation is available in Supplementary Video 1. **b**, Schematic drawing of mucosal deformation experimental set-up enabling optical recording of neuronal Ca^2+^ responses in full-thickness tissue collected from ileum of *Nmu-Cre;LSL-GCaMP6f* adult mice (18–22 weeks). Part of panel **b** is created with BioRender.com. **c**, Bar graph indicating the response type of myenteric (n = 42 neurons) and submucosal (n = 34 neurons) NMU^+^ cells (p = 0.2838; chi-square). **d**, Representative traces of individual epochs from quiescent responding cells demonstrating immediate, delayed and poststimulus responses. **e**, Bar graph indicating the number of epochs in quiescent responding cells with either type of response pattern in each plexus (p = 0.2503; chi-square). Immediate response myENC6:47/93 epochs; smENC1:40/66 epochs. **f**, Scatter plot showing the response latency for each epoch and mean (±s.e.m.) in quiescent responding cells of each plexus (p = 0.1235, two-tailed Student’s unpaired t test). **g**,**h**, Heat map visualizations of the percentage of quiescent responding cells responding during deformation at each of the 24 stimulated spots in the submucosal (**g**; n = 23 cells) or myenteric plexus (**h**; n = 23 cells), which can be used as an estimate of the average mucosal receptive field. **i**, Distribution histogram indicating the percentage of cells responding to different numbers of epochs in each plexus (p = 0.1984; chi-square). **j**, Distribution histogram indicating percentage of epochs with different numbers of spikes, showing more spikes in responding smENC1 neurons than myENC6 neurons per epoch (p = 0.0387; chi-square). Source data

To further confirm that the smENC1 morphology was aligned with an IPAN identity, low-titer AAV-DIO-tdTOM was administered to *Nmu-Cre* mice, achieving very sparse single-neuron labeling. From a single soma, we observed an extensive axonal network in the submucosal plane and collateral projections within the mucosal region, penetrating multiple villi (Extended Data Fig. 4a and Supplementary Video 1). To assess the ability of smENC1 to respond to mechanical deformation of the epithelia, full-thickness tissue from *Nmu-Cre;LSL-GCaMP6f* was imaged at a controlled stage set-up for sequential motorized mucosal poking (Extended Data Fig. 4b). Recordings of myenteric (myENC6; *n* = 42) or submucosal (smENC1; *n* = 34) plexus within 24 epochs (measurement associated with poking at a defined spot) identified the following three response categories: nonresponding, quiescent responding and spontaneous (Extended Data Fig. 4c). In both plexi, more than half of the neurons were quiescent responders, which were analyzed further (Extended Data Fig. 4d–j) and categorized based on response latency (Extended Data Fig. 4d). An immediate response was measured in more than half of the epochs per cell in both plexi (Extended Data Fig. 4e). Mean latencies, distribution of response latencies and number of responding epochs per cell were comparable between plexi (Extended Data Fig. 4f–i). Although only one spike was recorded in most epochs, considering the full distribution, larger number of spikes were observed per epoch in smENC1s than in myENC6s (*P* = 0.0387; Extended Data Fig. 4j). Taken together, smENC1 extends processes throughout multiple villi and is, similar to myENC6, responsive to mucosal poking, with an overall similar response pattern, suggesting that IPANs exist in both ENS plexi of the mouse small intestine.

### smENCs communicate through homotypic and heterotypic connections

**Fig. 4:**
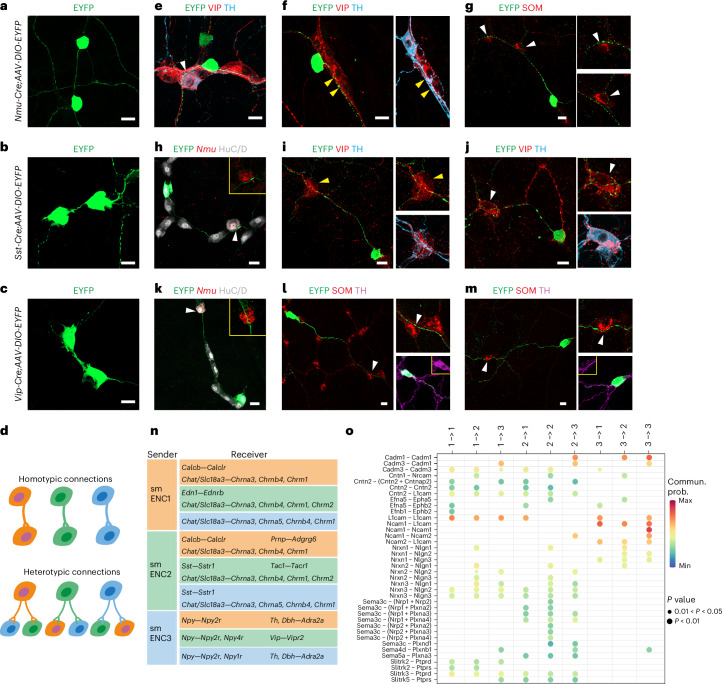
Neuronal projections of submucosal classes in the submucosal plane. **a**–**c**, Representative images of nerve processes between neurons belonging to the same submucosal class indicating potential homotypic connections. **d**, Schematic drawing indicating putative homotypic and heterotypic interactions between smENCs. **e**–**m**, Representative pictures of potential heterotypic connections indicated by the bouton-like structure of one neuron class close to the soma of another neuron class. White arrowheads (**e**,**j**)—TH^+^ VIP target cells; yellow arrowheads (**f**,**i**)—TH^−^ VIP target cells. **n**, Table summarizing possible ligand–receptor signaling interactions between smENC1–smENC3 based on manual screening (Supplementary Fig. 2) and CellChat analysis. **o**, CellChat dot plot of various cell-adhesion molecules that could mediate interactions between smENC1–smENC3. The dot color and size indicate the probability score of each interaction and the corresponding *P* values, respectively. The Benjamini–Hochberg correction is applied to adjust *P* values to control the false discovery rate. See also Extended Data Fig. 6 for the entire CellChat plot. All images show submucosal peels. Representative images in **a**–**c**, **e**–**m** were based on observations in five fields of view from each mouse (*n* = 3), Scale bars, 20 µm (**a**–**c**, **e**–**m**). Mice age = 8–12 weeks.

**Extended Data Fig. 5:**
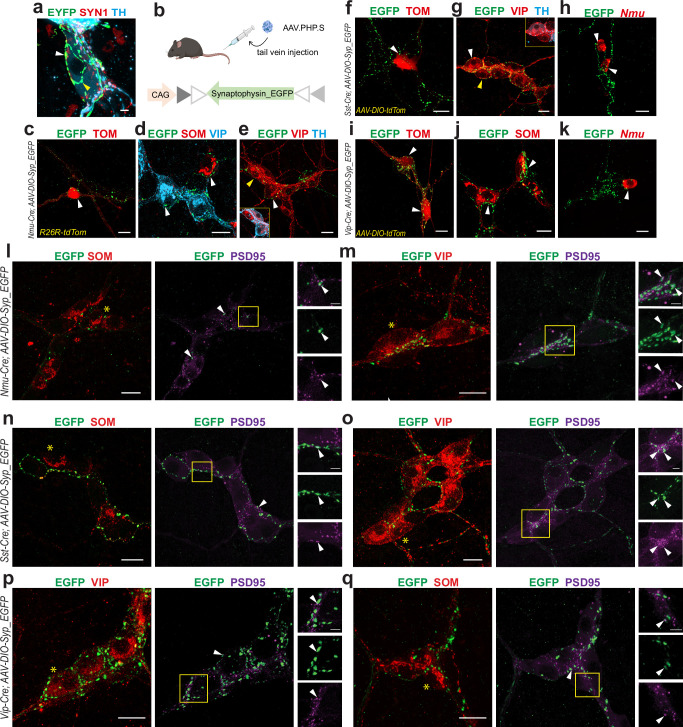
Close proximity of presynaptic and postsynaptic sites indicates synaptic homotypic and heterotypic connections of smENC1–3. **a**, Representative picture showing correlation between bouton-like structure (green puncta) and pre-synaptic marker SYN1 (white arrowhead: SYN1^+^, yellow arrowhead: SYN1^−^). **b**, Schematic drawing showing the viral-mediated strategy to label pre-synaptic sites in submucosal neuron classes. See Supplementary Fig. 5 for validation of strategy. Panel **b** is created with BioRender.com. **c**,**f**,**i**, Representative pictures addressing potential homotypic communication showing Syp-EGFP puncta surrounding TOM^+^ neuron somas in animals co-injected with AAV-DIO-Syp_EGFP and AAV-DIO-tdTOM (**f**,**i**) or AAV-DIO-Syp_EGFP (**c**). **d**,**e**,**g**,**h**,**j**,**k**, Representative pictures addressing potential heterotypic communication showing Syp-EGFP puncta in close proximity to somas of different neuron classes. **l**–**q**, Representative pictures showing proximity of presynaptic (Syp-EGFP) and postsynaptic (PSD95) sites indicating homotypic (**n**,**p**) or heterotypic communication (**l**,**m**,**o**,**q**). White arrowheads—colocalization between Syp-EGFP puncta and PSD95. Asterisk indicates identified potential target cell. Representative images were based on observations in 5 fields of view from each mouse (n = 3 in **a**,**d**,**e**,**g**,**h**,**j** and **k**; n = 2 in **c**,**f**,**i**,**l**–**q**). Scale bars, 20 µm, except for in the enlarged pictures, 5 µm. Mice age, 8–12 weeks.

Neuron-class-specific fluorescent labeling in Cre mice allowed intricate morphology analysis. However, as fluorescence is distributed across the neuronal projections, we reasoned that the methodology could be extended to investigate connections between smENCs by assessing axon-to-soma relationships. *Nmu-Cre*, *Sst-Cre* and *Vip-Cre* mice were injected with AAV-DIO-EYFP, followed by staining for complementary smENC1–smENC3 markers. All three neuron types exhibited homotypic connections, that is, nerve processes between neurons belonging to the same class (Fig. 4a–d). Furthermore, a variety of heterotypical connections (Fig. 4d) were indicated by the proximity of bouton-like structures (puncta) from one class near the soma of another class. For instance, puncta from smENC1 were detected close to neurons expressing VIP (TH^+^ and TH^−^; Fig. 4e,f) or SOM (Fig. 4g). Likewise, puncta from smENC2 juxtaposed *Nmu*^+^ (Fig. 4h) and VIP^+^ neurons (TH^+/−^; Fig. 4i,j), while puncta on axons emanating from smENC3 (TH^+/−^) appeared to contact *Nmu*^+^ (Fig. 4k) and SOM^+^ neurons (Fig. 4l,m). Although we found correlation between boutons and the presynaptic protein synapsin 1 (SYN1), not all puncta colocalized clearly and may not represent release sites (Extended Data Fig. 5a). To label presynaptic sites with higher fidelity, we used AAV to deliver Cre-inducible EGFP-fused synaptophysin (Extended Data Fig. 5b), which has previously been used to mark synapses in the central nervous system (CNS)^19^. A high correlation was confirmed between Syp-EGFP puncta and SYN1 in the three class-specific Cre lines injected with AAV-DIO-SYP-EGFP (Supplementary Fig. 5). To investigate homotypic connections, we co-injected AAV-DIO-SYP-EGFP and AAV-DIO-tdTOM into *Sst-Cre* and *Vip-Cre* mice or AAV-DIO-SYP-EGFP into *Nmu-Cre*; *R26RTom* mice. We identified EGFP^+^ puncta in close proximity to TOM^+^ soma in all three cases, again suggesting homotypic neuronal connections (Extended Data Fig. 5c,f,i). Further single injections of AAV-DIO-SYP-EGFP complemented with smENC markers confirmed all heterotypic connections (Extended Data Fig. 5d,e,g,h,j,k). Finally, a nanobody against PSD95 was applied to visualize postsynaptic sites. Colocalization of presynaptic and postsynaptic sites corroborated submucosal neuron–neuron synaptic interactions (Extended Data Fig. 5l–q).

**Extended Data Fig. 6:**
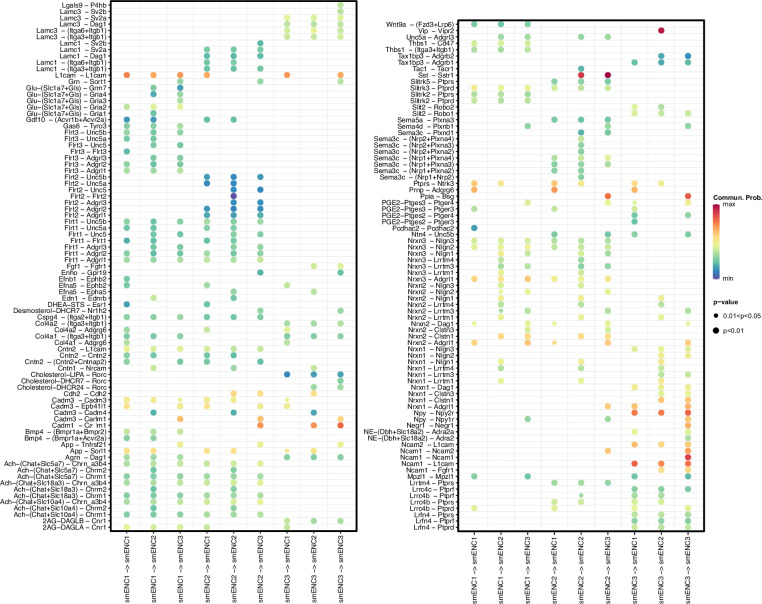
CellChat analysis addressing ligand–receptor communication between smENC1–3. Dotplots of CellChat analysis displaying possible ligand–receptor interactions between pairs of smENC1–3. The dot color shows the relative score of communication probability value of each interaction pair. The dot size indicates the level of significance of the interaction. The Benjamini–Hochberg (BH) correction is applied to adjust p-values to control the false discovery rate (FDR).

We reasoned that ligand–receptor analysis within the scRNA-seq profiles could help predict potential signaling pathways used in both homotypic and heterotypic connections. Through manual interrogation (Supplementary Fig. 2) and CellChat (v2), a tool for quantitative analysis of cell–cell communication (Extended Data Fig. 6), we identified, for instance, *Calcb–Calclr* in homotypic smENC1 connections and *Sst–Sstr1* between smENC2 and smENC3, while smENC3 to smENC2 connections may use *Vip–Vipr2* signaling (Fig. 4n). Additionally, nonsynaptic signaling interactions included *Ptger2* (prostaglandin E), *Bmp4*, *Tgfb1*, *Fgf1*, *Wnt9a* and *Gdf10* signaling (Fig. 4n and Extended Data Fig. 6). The cell–cell contacts may form and exhibit unique interaction patterns through synaptic adhesion molecules. CellChat predicted that each neuron pair has the possibility to form unique connections by using combinatorial patterns of neurexins/neuroligins, contactins, Ncam, semaphorins, Eph–Ephrin and Slitrk/Ptpr (Fig. 4o).

In summary, our connectome analysis implied comprehensive interconnectivity in the submucosal plexus, and we propose potential adhesion molecules within these connections along with signaling molecules that could mediate cell–cell communication.

### Connectivity of smENCs within the submucosa–mucosal region

**Fig. 5:**
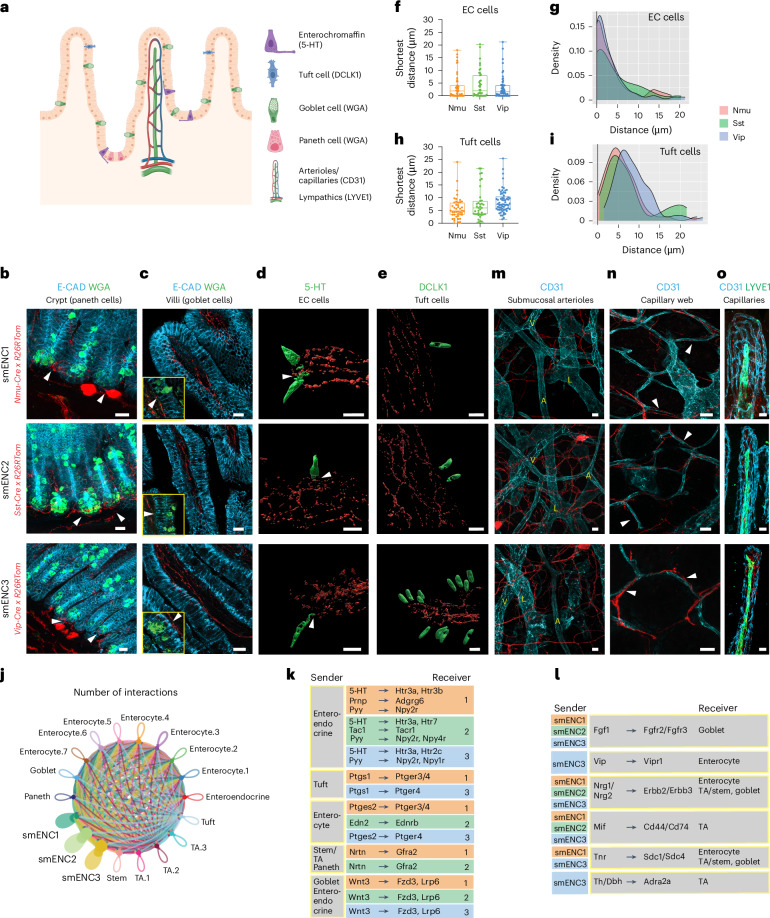
Neuronal projections of submucosal classes within the mucosa. **a**, Schematic drawing depicting non-ENS cell types/structures and their corresponding markers used in the study. Panel **a** is created with BioRender.com. **b**,**c**, Representative pictures showing TOM^+^ projections from the three smENCs around intestinal crypts (**b**) and inside villi (**c**). Arrowheads in insets in **c** indicate nerve fibers passing the basolateral side of goblet cells. **d**,**e**, Three-dimensional reconstructed Imaris images showing the spatial relation between the nerve fibers from smENCs and 5-HT^+^ EC cells (**d**) or DCLK^+^ tuft cells (**e**). See Supplementary Fig. 6 for original confocal pictures and for examples of long cell–cell distances. **f**–**i**, Analysis of the shortest distance between TOM^+^ nerve varicosity and EC cells or tuft cells using Imaris. **f**,**h**, Box-and-whisker plots showing individual shortest distances measured. Whiskers indicate the maximum and minimum values, boxes indicate the 25th–75th percentiles and the center line indicates the median. Number of cells analyzed from three mice of each line—51 (Nmu), 43 (Sst) and 60 (Vip) 5-HT^+^ EC cells; 44 (Nmu), 37 (Sst) and 64 (Vip) DCLK^+^ tuft cells. Each dot represents one cell. **g**,**i**, Density plots showing the distribution of the shortest distance measured. **j**, Aggregated circle plot showing number of interactions in CellChat analysis between smENCs and 15 epithelial cell types. **k**, Table showing examples of identified ligand–receptor pairs with epithelial cell types as senders and smENC1–smENC3 as receivers. **l**, Table showing examples of identified ligand–receptor pairs with smENC1–smENC3 as senders and epithelial cell types as receivers. Neurovascular spatial relation shown at three planes—submucosa (**m**), crypt (**n**) and villi (**o**). TOM^+^ fibers in all three Cre lines were most closely distributed to capillaries in the crypt and villi (Supplementary Videos 2–4). Representative images in **b**,**c**,**m**–**o** were based on observations in five fields of view from each mouse (*n* = 3). Scale bars, 20 µm (**b**,**c**,**m**–**o**). Mice age = 8–12 weeks. L, lymphatic vessel; A, arteriole; V, venule; EC, enterochromaffin; TA, transit amplifying. Source data

IPANs are believed to receive information from epithelial cells, in particular enterochromaffin cells, while secretomotor neurons are thought to give efferent input, mainly to secretory enterocytes of the crypts. To assess the proximity between the smENCs and epithelial cells (Fig. 5a), we visualized the innervation pattern in class-specific Cre mice crossed with *R26RtdTom* mice. We observed an extensive network of axons within the villi (Fig. 5b,c and Extended Data Fig. 4a) not only from smENC1 (presumed IPANs) but also from smENC2 and smENC3. Similarly, axonal tracts from all three smENCs surrounded the crypts, harboring Paneth cells (marked by wheat germ agglutinin (WGA); Fig. 5b). Within villi, WGA instead labeled goblet cells, which also appeared close to projections from all three smENCs (Fig. 5c). We next focused on the axonal proximity to enterochromaffin cells due to their acclaimed interaction with IPANs. We immunostained enterochromaffin cells for 5-HT and compared them with DCLK1^+^ tuft cells in the class-specific *R26RtdTom* mice (Fig. 5d,e). Surprisingly, TOM^+^ axons from all three submucosal classes were found in close and comparable proximity to enterochromaffin cells, which often displayed neuropod structures^20^, suggesting communication with all three smENCs, not just smENC1 (Fig. 5d,f,g and Supplementary Fig. 6a–c,g–l). In contrast, axons from all three smENCs showed larger distances to tuft cells (Fig. 5e,h,i and Supplementary Fig. 6d–f).

**Extended Data Fig. 7:**
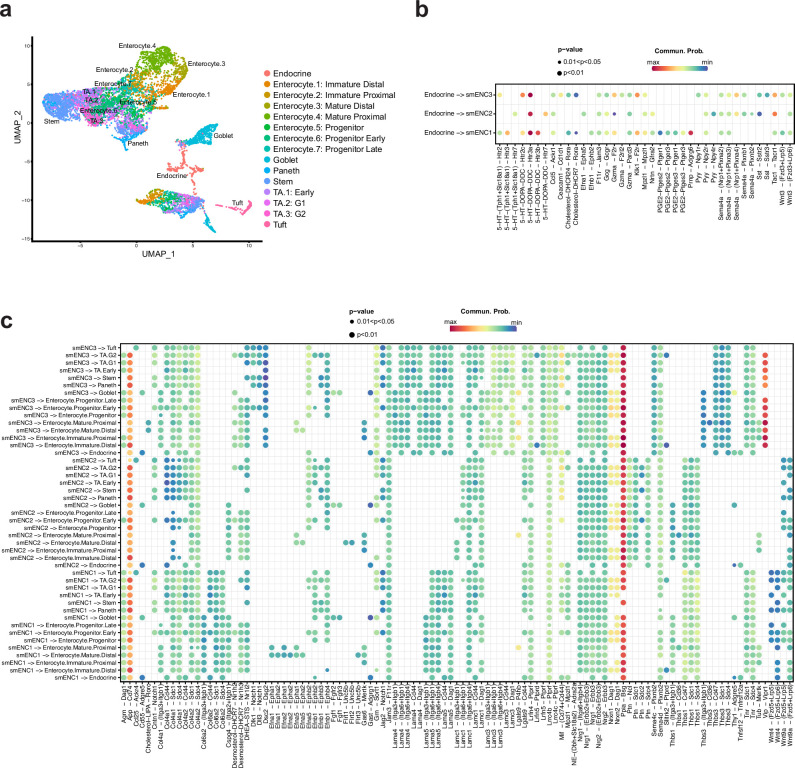
CellChat analysis addressing ligand–receptor communication between epithelial cell types and smENC1–3. Related to Fig. 5. **a**, Reconstructed UMAP of scRNA-seq of epithelial cell types^21^. **b**, Dot plots of enteroendocrine cells as senders to smENC1–3. **c**, Dot plots of smENC1–3 as senders to the epithelial cell types. The dot’s color and size indicate the probability score of each ligand–receptor pair interaction and the corresponding p-values, respectively. The Benjamini–Hochberg (BH) correction is applied to adjust p-values to control the false discovery rate (FDR). Note, dot plots for remaining epithelial cells as senders can be viewed in Supplementary Fig 7.

To predict plausible signaling pathways used by epithelial cells and smENCs, we incorporated a public small intestine epithelial scRNA-seq atlas^21^ (Fig. 5j and Extended Data Fig. 7a) and applied CellChat (v2) analysis, complemented by manual interrogation. Supporting interaction between the submucosal neurons and 5-HT^+^ enteroendocrine cells, we detected serotonin receptors in all three smENCs, not just in presumed IPANs (Fig. 5k, Supplementary Fig. 2 and Extended Data Fig. 7b). Moreover, enteroendocrine cells expressed *Tac1*, indicating tachykinin production, which could potentially be perceived by *Tacr1*-expressing smENC2. The overall analysis identified *Neurturin–Gfra2*, *Edn–Ednrb*, *Wnt*, *BMP* and *Prostaglandin E (Ptgs1*/*Ptges2—Ptger3*/*Ptger4)* as potential signaling pathways emanating from enterocytes, tuft, goblet, stem/TA cells and Paneth cells (Fig. 5k, Extended Data Fig. 7b and Supplementary Fig. 7a–e). Reciprocal signaling from smENCs to epithelial subpopulations included *Fgf1–Fgfr2*/*Fgfr3*, *Vip–Vipr1*, *Nrg1*/*Nrg2–Erbb2*/*Erbb3*, *Tnr–Sdc1*/*Sdc4* and *Mif–Cd44*/*Cd74* (Fig. 5l and Extended Data Fig. 7c).

Neural control of vascular tone is thought to necessitate proximity to blood vessels, in particular arterioles. We investigated the class-specific reporter mice at the level of submucosal arterioles, mucosal capillaries and villus capillaries in an unbiased manner. Axons from all three smENCs occasionally aligned with capillaries in the mucosal space and extended throughout the villus edges (Fig. 5n,o and Supplementary Videos 2–4); however, submucosal arterioles were not obviously innervated (Fig. 5m). In contrast, arterioles are densely innervated by TH^+^ sympathetic fibers, which establish a vasoconstrictive tone^7,22,23^. Taken together, our proximity analysis revealed extensive communication possibilities of smENC1–smENC3 with various epithelial cell types, all showing closer connections to enterochromaffin cells than anticipated. However, there was a lack of specific anatomical closeness to suggest substantial direct vasodilatory activities for any of the smENCs.

### Postnatal scRNA-seq captures submucosal neuron development

**Fig. 6:**
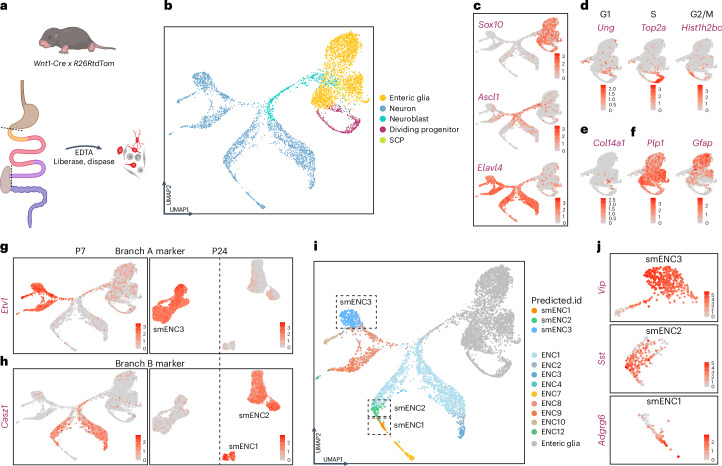
scRNA sequencing at P7 identifies submucosal development within branches A and B. **a**, Schematic drawing indicating the dissociated region from postnatal *Wnt1-Cre* x *R26RtdTom* pups and subsequent single-cell preparation. Panel **a** is created with BioRender.com. **b**, UMAP displaying generic differentiation states in the developing ENS. **c**–**f**, Feature plots showing expression of generic markers for glia/progenitors, neuroblasts and neurons (**c**), cell cycle phases (**d**), SCPs (**e**) and enteric glia (**f**). See Extended Data Figs. 8 and 9 for more marker expression used to delineate (**b**). **g**,**h**, Feature plots displaying branches A (**g**) and B (**h**) gene markers in postnatal ENS development (P7) and in juvenile smENCs (P24). **i**, UMAP with identity transfer and predicted.id for smENC1–smENC3; myENC1–myENC4, myENC7–myENC10, myENC12 and ‘enteric glia’. **j**, Feature plots displaying cardinal smENC marker expression validating the predicted ENC identities in the corresponding predicted.id areas in **i** (jagged boxes).

**Extended Data Fig. 8:**
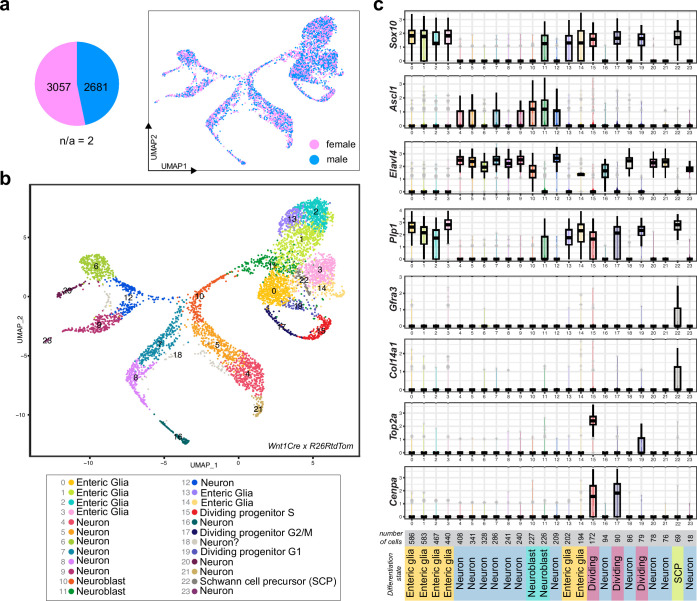
ENS clusters in scRNA-seq dataset at P7 and their differentiation state. Supportive data related to Fig. 6. **a**, Inferred male and female cell distribution shown on a pie chart and feature plot. **b**, UMAP of postnatal day 7 ENS clusters. Legend indicates proposed differentiation state of each cluster as determined based on differentially expressed genes (Supplementary Table 4 and analysis in **c**). **c**, Box plots indicating defining genes used to determine the differentiation states of clusters. Box-and-whisker plots indicate max–min (whiskers), 25–75 percentile (boxes) with median as a center line.

**Extended Data Fig. 9:**
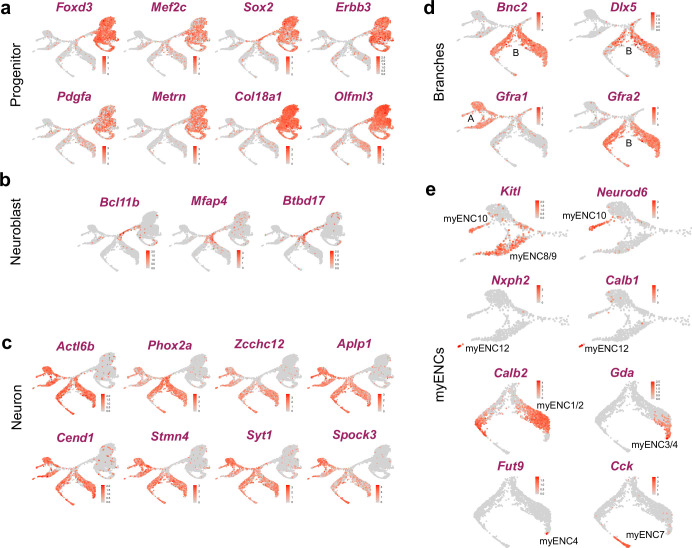
Expression of marker genes of different maturity stages or cell types at P7. Supportive data related to Fig. 6. Feature plots displaying marker genes of different maturity stages or cell types in the developing ENS at P7—**a**, progenitors; **b**, neuroblasts; **c**, neurons; **d**, early branch markers; **e**, myenteric neuron class markers (myENCs).

We have previously investigated myenteric neuron development by connecting transcriptomes of juvenile and embryonic ENS. Twelve myenteric neuron class identities (myENC1–myENC12) were found to be gradually acquired within two major neuronal trajectories, named branches A and B. These findings were based on scRNA-seq data dominated by myenteric neuron differentiation^15^. To instead investigate submucosa neuron differentiation, we performed scRNA-seq of ENS isolated from the small intestines of *Wnt1-Cre;R26RtdTom* mice at postnatal day (P) 7 (Fig. 6a), when submucosal neuron differentiation predominate^12^. We obtained 5,740 healthy ENS cell profiles and performed cluster analysis (Extended Data Fig. 8a,b and Supplementary Table 4). Clusters were annotated to generic differentiation states (glia/progenitors, neuroblasts or neurons) based on their relative expression of *Sox10*, *Ascl1* and *Elavl4* (Fig. 6b,c and Extended Data Fig. 8b,c). Glia/progenitor clusters were signified by the previously identified^15^ transcription and signaling factors *Foxd3*, *Mef2c*, *Pdgfa* and *Metrn* (Extended Data Fig. 9a). Three glia/progenitor clusters corresponded to the cell cycle phases (Fig. 6d and Extended Data Fig. 8c), while one cluster was identified as Schwann cell precursor (SCP) based on previously defined SCP markers in ENS^15^, including *Col14a1*, *Gfra3* and *Dhh* (Fig. 6e and Extended Data Fig. 8c). The remaining glia/progenitor clusters were categorized as presumed glia and expressed generic (*Plp1*) and subtype glial markers (*Gfap*; Fig. 6f). Neuroblasts expressed previously identified genes, including *Mfap4* and *Bcl11b*^15^, while transcriptional regulators *Actl6b*, *Phox2a* and *Zcchc12* marked neurons (Extended Data Fig. 9b,c).

The overall manifold projection mirrored that of earlier stages^15^, with no additional major neurogenic root. Neuroblasts appeared to undertake a binary decision, activating transcriptional programs equivalent to the previously described branch A (*Etv1*) or branch B (*Casz1*; Fig. 6g,h and Extended Data Fig. 9d). These branch markers were also detected at the juvenile stage (Fig. 6g,h), indicating that smENC3 arises within branch A and smENC1 and smENC2 within branch B. To identify the emergence of smENC1–smENC3, label transfer analysis was conducted by mapping the combined cardinal submucosal and myenteric transcriptome cluster profiles on the P7 cells (Fig. 6i). Cells with predicted identities of smENC1–smENC3 expressed their respective cardinal marker *Adgrg6*, *Sst* or *Vip* (Fig. 6j). Myenteric class markers^15^ were also confirmed in cells with the corresponding predicted identities (Extended Data Fig. 9e). Our analysis revealed overall conserved differentiation pathways for myenteric and submucosal neurons.

### Identity acquisition via diversification in neuron branches

**Fig. 7:**
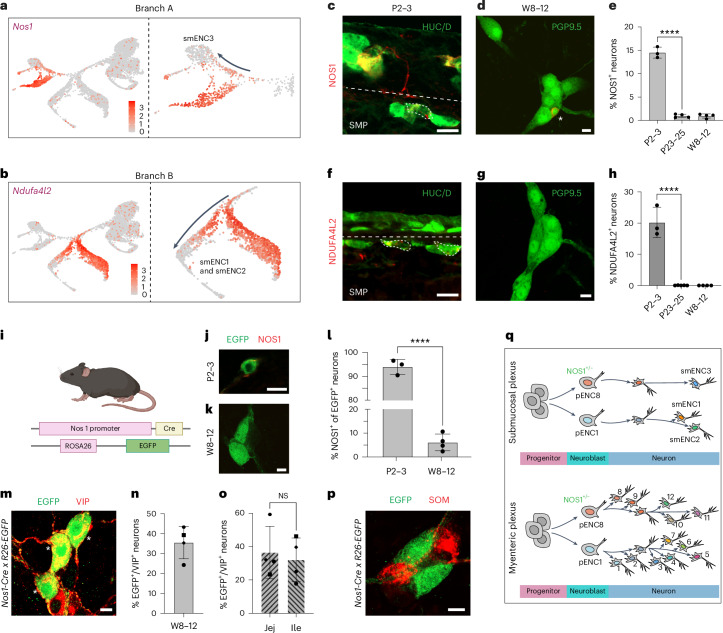
smENC identities are formed through neuronal phenotypic transitions. **a**,**b**, Feature plots displaying *Nos1* or *Ndufa4l2* in branch A (**a**) or B (**b**) at P7, indicating their expression in smENC1–smENC3 trajectories. **c**,**d**, Immunohistochemistry of NOS1 in submucosal neurons showing a decreased expression from early postnatal (**c**) to adult stages (**d**) in wild-type mice. **e**, Graph showing quantification of NOS1^+^ submucosal neurons exemplified in **c** and **d**. P2–3—14.51 ± 1.18% (*n* = 3 mice); P23–25—0.97 ± 0.37% (*n* = 4 mice); W8–W12—0.96 ± 0.44% (*n* = 4 mice; P2–3 versus P23–23—*P* < 0.0001). **f**,**g**, Immunohistochemistry of NDUFA4L2 in submucosal neurons showing a decreased expression from early postnatal (**f**) to adult stages (**g**) in wild-type mice. **h**, Graph showing quantification of NDUFA4L2^+^ submucosal neurons exemplified in **f** and **g**. P2–3, 20.19 ± 4.86% (*n* = 3 mice); P23–25, 2 NDUFA4L2^+^ of 7,615 submucosal neurons (*n* = 5 mice) and W8–W12, 0 NDUFA4L2^+^ of 8,087 submucosal neurons (*n* = 4; P2–3 versus P23–23, *P* < 0.0001). **i**, Schematic drawing of alleles in *Nos1-Cre* x *R26EGFP* mice. Panel **i** is created with BioRender.com. **j**–**l**, Immunostaining (**j**,**k**) and quantification (**l**) showing gradual loss of NOS1 expression in EGFP^+^ cells in submucosal peels of *Nos1-Cre* x *R26EGFP* mice. P2–3, 94.01 ± 3.16% (*n* = 3 mice); W8–W12, 6.12 ± 3.50% (*n* = 4 mice; P2–3 versus P23–23—*P* < 0.0001). **m**, Immunohistochemistry showing EGFP expression in VIP^+^ smENC3 submucosal cells (NOS1^−^) indicated with stars in adult *Nos1-Cre* x *R26EGFP* mice. **n**,**o**, Quantification of VIP^+^ EGFP^+^ cells exemplified in **m** (**n**) and with regard to ileum or jejunum (**o**; no difference between regions) in *Nos1-Cre* x *R26EGFP* mice. 35.53 ± 8.17% (*n* = 4 mice). Each animal was assigned a unique symbol. **p**, Representative immunostaining showing no overlap between EGFP and the smENC2 marker SOM. A total of 0 SOM^+^ of 815 EGFP^+^ neurons (*n* = 4 mice). **q**, Schematic drawing contrasting and comparing myenteric and submucosal neuron diversification. Both submucosal and myenteric neurons are generated through binary branching and phenotypic conversions of immature neurons. The later generation of submucosal neurons may lead to the generation of fewer classes of neurons. Two-tailed Student’s *t* test was performed to determine statistical significance. Data are presented as mean ± s.d. Each dot represents one animal. ****P < 0.0001 and NS, *P* > 0.05. Scale bars, 10 μm (**c**,**d**,**f**,**g**,**j**,**k**,**m**,**p**). Source data

During myenteric neuron differentiation, we previously discovered that maturing cells transition through ‘prototypic’ continuous states at the root of each neurogenic branch. These states resemble the transcriptional profiles of myENC8/9 (branch A; pENC8) and myENC1/myENC2 (branch B; pENC1)^15^ and subsequently transform into the various neuron class identities. We next investigated whether submucosal ENS differentiation follows a similar stepwise process. In branch A, the pENC8 marker NOS1 was associated with the smENC3 differentiation pathway (Fig. 7a), while in branch B, the marker NDUFA4L2 (pENC1) was linked to a subbranch giving rise to smENC1 and smENC2 (Fig. 7b). To validate submucosal prototypic states, we analyzed NOS1 and NDUFA4L2 protein expression in developing submucosal tissue (Fig. 7c–h). At P2–3, ~15% of submucosal neurons expressed NOS1, but this decreased sharply to <1% at juvenile (P23–25) and adult stages (W8–12; Fig. 7c–e). Similarly, ~20% of the submucosal neurons expressed NDUFA4L2 at P2–3, while expression was essentially lost at juvenile and adult stages (Fig. 7f–h). These results show that submucosal neurons transiently exhibit prototypic characteristics, which are lost as they differentiate into smENC1–smENC3.

**Extended Data Fig. 10:**
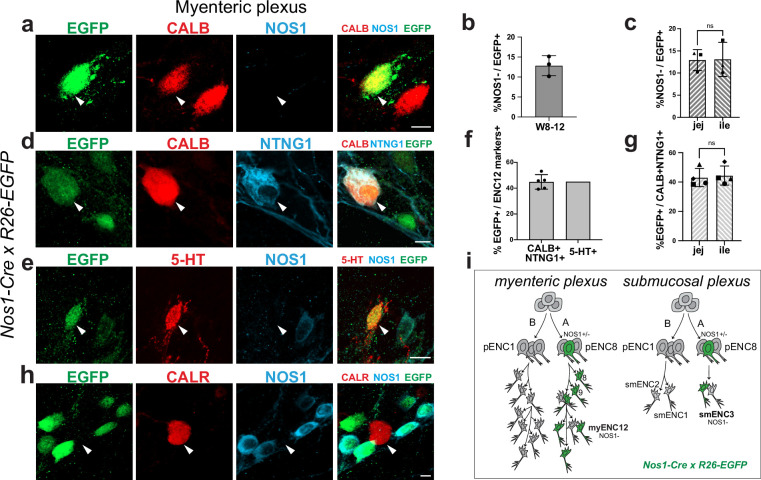
myENC12 is formed through phenotypic transition of NOS1^+^ branch A neurons. Supportive data related to Fig. 7. Representative images (**a**) and quantification (**b**,**c**) of EGFP^+^ myenteric neurons not maintaining NOS1 expression in *Nos1-Cre* x *R26EGFP* mice at W8–12. 12.84 ± 2.50% (n = 3 mice). No difference was detected between intestinal regions. Each animal was assigned a unique symbol in **c**. Representative images (**d**,**e**) and quantification (**f**,**g**) of EGFP^+^ myenteric neurons expressing NTNG1/CALB (44.84 ± 5.62%; n = 5 mice) or 5-HT (45%; 20 cells in 5 mice) in *Nos1-Cre* x *R26EGFP* mice at W8–12. No difference was detected between intestinal regions. Each animal was assigned a unique symbol in **g**. **h**, Representative image showing no overlap between EGFP and the branch B neuron marker CALR. 0 EGFP^+^ of 1903 CALR^+^ cells (n = 3 mice). **i**, Schematic drawing indicating observed and suggested EGFP expression, suggesting transient NOS1 expression within branch A in both myenteric and submucosal development. Two-tailed Student’s t test was performed to determine statistical significance. Each dot (*n*) represents one animal. Data are presented as mean ± s.d. NS, P > 0.05. Scale bars, 10 μm. jej, jejunum; ile, ileum. Source data

To more firmly determine if smENC3 arises from pENC8 transient neurons, we lineage-traced cells expressing Nos1 in *Nos1-Cre;R26EGFP* mice (Fig. 7i). At P2–3, 94% of EGFP^+^ submucosal neurons expressed NOS1, whereas this dropped to only 6% in adult mice, confirming a dramatic reduction in NOS1 expression during submucosal neuron development (Fig. 7j–l). Notably, neurons that no longer expressed NOS1 corresponded to smENC3, as all EGFP^+^ NOS1^−^ cells expressed VIP and over one-third of VIP^+^ neurons expressed EGFP (Fig. 7m–o). These findings suggest that smENC3 originates from a transient branch A prototypic identity that at least partially includes NOS1 expression (Extended Data Fig. 10i). Furthermore, no neurons were found to co-express EGFP and the smENC2 marker SOM (Fig. 7p), confirming that transient NOS1 expression is exclusive to branch A neurons.

In parallel, *Nos1-Cre;R26EGFP* mice were also used to confirm neuronal conversions during myenteric plexus development. Our previous study in this layer indicated that the generation of myENC12 depends on differentiation of NOS1^+/−^ pENC8 (ref. ^15^). Examining the myenteric plexus, we found that 13% of EGFP^+^ myenteric neurons had lost NOS1 expression by weeks 8–12 (Extended Data Fig. 10a–c) consistent with a previous study^24^. To investigate whether transient NOS1^+^ cells differentiate further into myENC12, we performed costaining with the class markers CALB/NTNG1 and the subclass marker 5-HT. In total, 45% of NTNG1/CALB^+^ neurons and 45% of 5-HT^+^ neurons were found to express EGFP (Extended Data Fig. 10d–g). As expected, no EGFP^+^ cells co-expressed CALR, a marker for branch B-derived myenteric neurons (that is, myENC1, myENC2, myENC5 and myENC6; Extended Data Fig. 10h). These results demonstrate the emergence of myENC12 from NOS1-expressing prototypic branch A neurons in the myenteric plexus, representing a nitrergic to a cholinergic phenotype switch (Extended Data Fig. 10i).

Collectively, our findings strongly suggest that all neurons in the small intestine arise within two primary developmental trajectories (branches A and B; Fig. 7q). The fundamental principle appears to be that differentiating progenitor cells adopt one of two prototypic states and subsequently diversify into distinct neuron classes. However, compared to the myenteric plexus, the final cellular composition of the submucosal plexus exhibits less neuronal diversity, likely due to its later developmental timeline (Fig. 7q).

## Discussion

This study offers an in-depth examination of the submucosal neuron classes in the mouse small intestine with regard to their molecular profiles, morphological attributes and connectivity. Furthermore, we reveal a stepwise differentiation process through which submucosal neuron identities arise, suggesting its applicability to the entire developing ENS across the gut wall.

Our scRNA-seq analysis revealed three cardinal submucosal neuron classes characterized by *Nmu*/*ChAT*/*Calcb* (smENC1), *Sst*/*ChAT*/*Calcb* (smENC2) and *Vip*/*Npy*/*Th*^+/^^−^ (smENC3). We provide morphological, transcriptional and functional evidence to support the classification of smENC1 as submucosal IPANs, concluding that the lack of NF-M in submucosal IPANs may have hindered proper identification in previous studies. Interestingly, smENC3 expresses genes for noradrenaline production and uptake, but the lack of Dopa decarboxylase (*Ddc*) suggests they cannot produce noradrenaline under steady-state conditions. Whether certain stimuli enable full biosynthesis or whether they reflect an evolutionary vestige remains unclear. Nevertheless, Th has been proposed to mark functionally distinct VIP neurons^7^, yet subclustering did not associate Th with a molecular subtype. Overall, subclustering revealed no smENC1 subgroup, while smENC2 and smENC3 split into subclusters that lacked unique marker genes, implying that subcluster heterogeneity stems from regional-, maturation- or activity-level variation.

Capitalizing on class-specific markers, we combined Cre mouse lines with transgenic reporters and viral-mediated labeling to uncover connectivity in the submucosa–mucosal space. A surprising finding was the presence of serotonin receptors in all three smENCs and the equal proximity of their projections to enterochromaffin cells, together implying responsiveness to luminal stimuli mediated by serotonin-secreting enterochromaffin cells. Secretomotor neurons, previously thought to rely on IPANs for sensory-evoked activation, may thus possess intrinsic sensory capabilities. IPANs share major neurochemical coding with cholinergic smENC2 (Calcb and Ach), indicating that the ‘division of labor’ may not be as rigid as previously thought and that submucosal neuron classes could respond to slightly different sensory modalities or with different thresholds to the same stimuli. Conversely, smENC1 may execute cholinergic secretomotor functions through axon reflex mechanisms^25^, supported by our observed axonal closeness between all smENCs and crypt enterocytes. VIP^+^ smENC3 projections in the crypt area, combined with the receptor *Vipr1* in enterocytes, fit the presumed major role of VIP^+^ secretomotor neurons in regulating secretion^4,26,27^. Tachykinins and acetylcholine in smENC2 corroborated a secretomotor role also of smENC2 (refs. ^4,26,28^). Conversely, none of the smENCs exhibited obvious connections with arterioles, aligning with several studies of the mouse intestine^22,23^. Hence, vasodilatory actions^4,29^ of mouse smENCs may be indirect, possibly mediated through connective tissue or capillaries.

Our systematic connectivity analysis indicated homotypic communication, potentially amplifying local activity, along with extensive heterotypic connections, likely ensuring coordinated and balanced overall responses to mechanical, chemical and osmotic stimuli. Between the two secretomotor types, *Sst–Sstr1* and *Vip–Vipr2* may mediate communication, although experimental validation is needed. Indeed, the ENS field would benefit from clarifying communication modes (synaptic versus volume transmission) and contact points (axon–soma versus axon–axon or axon–dendrite). Our combined visualization of presynaptic and postsynaptic proteins supports that submucosal neurons communicate at least in part through axon–soma synaptic contacts. We hope that the refined molecular profiles and connections of submucosal neurons presented here may help unravel the cellular and molecular interplay maintaining balance between absorption and secretion at homeostasis versus pathological conditions. However, for any translational purposes, we recognize the importance of careful comparison between the mouse and human submucosal neurochemical coding.

Clear transcriptional similarities were discovered between pairs of myenteric and submucosal neuron classes (smENC1–myENC6, smENC2–myENC5 and smENC3–myENC11). We propose to denote these pairs ‘transcriptional siblings’—a term that highlights molecular equivalence and similarity between cell types that in the adult body are anatomically and/or functionally distinct. One may wonder how similar neurons may emerge in both plexuses. Seminal studies from the Gershon Lab, later expanded by the Young Lab, established that neurons with different phenotypes are generated in birth waves that peak at different developmental time periods^24,30^. Although the neural birth-dating would need reinterpretation in the light of recent discrete molecular identity codes, some insights can be drawn from the original studies. For instance, 5-HT^+^ neurons, which we identified as a myENC12 subtype^15^, are the earliest generated neuron type (birth peak at E10–E12)^24,30^, while scRNA-seq identified myENC5 and myENC11 as late-arising neurons^15^. Therefore, it may be the overlapping neurogenesis window at late embryonic-to-early postnatal stages that allows transcriptionally similar cells to be formed in both plexi. Conversely, early born neuron types, such as myENC12 neurons^15^, are limited to the myenteric plexus, as they emerge before submucosal development has begun. We suggest a shared developmental program in the ENS, where neuron identities are shaped by the timing of generation. Deciphering the exact mechanisms and molecular programs involved in temporal cell fate specification will be a large endeavor for future studies of ENS development.

Regardless of developmental stage, ENS stem cells undergoing neurogenesis appear to make a binary choice between branch A or B, proceeding through branch-specific prototypic states followed by final identity acquisition. As proof-of-principle for the further diversification of immature but committed neurons, we demonstrate that both smENC3 and myENC12 arise from transient NOS1^+^ branch A prototypic neurons. The transient NOS1 expression could not be attributed to all smENC3 and myENC12 neurons, which either means that not all prototypic cells express NOS1 or that some neurons differentiate in a direct manner. Given that nitrergic neuron deficiencies are hallmarks of several enteric neuropathies, including achalasia, diabetic gastroparesis and Chagas disease^31^, there is considerable clinical interest in producing stem cell-derived enteric NOS1^+^ neurons in vitro^32,33^. However, the transient NOS1 expression in both myenteric and submucosal plexus development should raise awareness of the likely occurrence of nonstable NOS1 expression also in cell culture conditions, and measures may be needed to ensure the longevity of phenotypic profiles in manufactured enteric neurons.

Submucosal neurons are linked to a wide range of conditions, including infectious (for example, cholera), inflammatory (for example, inflammatory bowel disease) and functional (for example, irritable bowel syndrome) disorders^34,35^, all of which lack satisfactory treatments. We anticipate that the molecular profiles and the differentiation pathways uncovered in this study could pave the way for submucosal neuron derivation from pluripotent stem cells. Possibly by addressing the relative temporal age of progenitors, the generation of late-born neurons could be facilitated. Combined with human gut organoids, submucosal neurons could advance research on neural mechanisms in fluid control at homeostasis or in models of neurocristopathies and inflammatory/infectious conditions.

## Methods

### Mice

The generation of the *Baf53b-Cre* (JAX, 027826), *Nmu-Cre* (MMRRC, 036643-UCD), *Vip-IRES-Cre* (JAX, 010908), *Sst-IRES-Cre* (JAX, 013044), *Cck-IRES-Cre* (JAX, 12706), *Wnt1-Cre*^36^ and *Nos1-Cre*^37^ *(*JAX 017526*)* mouse strains have been previously described. Strains were crossed with *Ai14 (R26RtdTom*; JAX, 007908*)*, *R26EGFP* (JAX, 004077) or *Ai148D (LSL-GCaMP6f*; JAX, 030328). Wild-type mice used in the study were C57/B6J. Animals were group housed, with food and water ad libitum, under 12-h light/12-h dark cycle conditions, 22 °C ambient temperature and 50% humidity. Animal experiments were approved by the local ethics committee in northern Stockholm (Stockholm Norra Djurförsöksetiska Nämnd, Jordbruksverket; N5264/18, N6626-2019 and N5237-2023). Procedures described in Extended Data Fig. 4 were reviewed and approved by the Mayo Clinic Institutional Animal Care and Use Committee. The Mayo Clinic is American Association for Accreditation of Laboratory Animal Care (AAALAC)-accredited (000717) and has Assurance with the Office of Laboratory Animal Welfare, OLAW (A3291-01). All experiments included both male and female mice.

### Tissue preparation for scRNA-seq

#### Juvenile tissue preparation

A total of eight *Baf53b-Cre* x *R26RtdTom* mice (both sexes), postnatal day (P)24, were used for scRNA-seq. The tissue was kept in dissection solution—Tris–HEPES recovery solution containing 76 mM Tris–HCl, 19.5 mM Tris base, 2.5 mM KCl, 1.2 mM NaH_2_PO_4_, 20 mM HEPES, 30 mM NaHCO_3_, 25 mM glucose, 5 mM sodium ascorbate, 2 mM thiourea, 3 mM sodium pyruvate, 0.5 mM CaCl_2_ and 10 mM MgSO_4_ (pH 7.3–7.4). The dissection solution was equilibrated in 95% O_2_, 5% CO_2_ for 30 min before the start of the experiments and held on ice at all steps. Three fire-polished Pasteur pipettes with decreasing opening size (from ~70% to 10% of the original opening size) were coated in a 1% BSA solution for 1 h at room temperature. The small intestines were cut into 5 cm pieces, and each segment was flushed inside with ice-cold dissection solution. The mesentery was removed, and the intestinal pieces were opened along the mesenteric border, then transferred to prewarmed epithelial strip buffer, consisting of 5 mM EDTA, 1 mM DTT, 5% fetal calf serum (FCS) in Hanks’ balanced salt solution (HBSS) and incubated at 37 °C for 30 min with regular shaking. The pieces were transferred to cold dissection solution, and epithelial cells were brushed off and transferred to a new cold dissection solution. The tissue pieces were pinned, mucosa side down, onto a Sylgard (Dow Corning) coated dish. The smooth muscle layers, containing the myenteric plexus, were peeled off from the submucosa using watchmaker forceps, Peyer’s patches were removed and the submucosa/mucosa layers were stored in dissection solution on ice until all pieces were peeled. Peels were cut into 2–5 mm^2^ pieces and placed in prewarmed digestion solution (1 mg ml^−1^ Liberase TH Research Grade (Roche), 0.2 mg ml^−1^ DNaseI (Worthington Biochem, LK003172) and 12 U ml^−1^ dispase (Corning, 354235) in dissection solution at 37 °C. The tissue was distributed into tubes with 5 ml of digestion solution in each. After 15 min, the tissue was dissociated with the wide-tip Pasteur pipette by pipetting slowly ten times. After an additional 15 min, the tissue was dissociated with a medium-tipped Pasteur pipette ten times. The digestion was stopped by adding 10% BSA and 5 mM EDTA (final concentration) and doubling the volume of dissection solution. A narrow-tipped Pasteur pipette was used for final slow trituration 20 times on ice. The cell suspension was applied to a MACS SmartStrainer (70 m; Miltenyi Biotec) followed by 2 ml fresh cold dissection solution. Single cells were collected by centrifugation at 150*g* for 10 min at 4 °C. The pellet was resuspended in cold DPBS with 1% BSA. Tomato^+^ cells were sorted by flow cytometry on a BD Biosciences InFlux (v7) cell sorter and collected in ice-cold PBS containing 0.04% BSA (see Supplementary Fig. 8 for representative plots). We captured ~0.7% tdTOM^+^ cells out of the total singlets. Cell gating was performed on BD FACS Software (v.1.0.0.650).

#### Postnatal tissue preparation

Four *Wnt1-Cre*;*R26RtdTom* P7 mice (both sexes) were used for scRNA-seq experiments. During all stages of the dissociation protocol, the tissue was kept in ice-cold DMEM/F12 medium (Invitrogen), except if mentioned otherwise. Small intestines were dissected and flushed with a blunt needle. The mesentery and Peyer’s patches were removed. The intestines were opened longitudinally, washed with PBS, cut into 2–3 cm pieces and put into predigestion solution (5 mM EDTA and 2% BSA in PBS) for 10 min at 37 °C. Epithelial cells were shaken off by vortex, and tissues were washed in DMEM/F12 twice. The tissues were then merged in dissociation solution (0.75 mg ml^−1^ Liberase TH Research Grade, 3 U ml^−1^ dispase and 0.2 mg ml^−1^ DNAse I in DMEM/F12) at 37 °C for 20 min with shaking every 5 min. The enzyme mixture was replaced with DMEM/F12 medium containing 2% BSA and 5 mM EDTA. The cells were manually triturated using three fire-polished Pasteur pipettes with decreasing opening size (from ~70% to 10% of the original opening size) that were previously coated with 1% BSA solution for 1 h at room temperature. The single-cell suspension was filtered through a DMEM/F12-equilibrated 40 μm cell strainer (Miltenyi Biotec) and centrifuged at 150*g* for 5 min at 4 °C. Cells were resuspended in DPBS containing 1% BSA and 1% DRAQ7 (Biostatus). tdTom^+^ cells were sorted on a BD Influx (v7) Cell Sorter and collected in ice-cold PBS containing 0.04% BSA. We captured 7.2% tdTom cells out of the total Draq7 singlets. The gating of cells was performed on BD FACS Software (v.1.0.0.650).

### scRNA-seq

The sampling was carried out with the 10x Genomics Chromium Single-Cell Kit (10x Genomics) version 2 (P7) and version 3 (P24) at the Eukaryotic Single-Cell Genomics (ESCG). Cell suspensions were adjusted to 500–1,000 cells per μl and added to 10x Chromium reverse transcription mix to achieve loading target numbers between 2,500 and 5,000 cells. The manufacturer’s instructions were followed for downstream cDNA synthesis, library preparation and sequencing (NovaSeq S2 reagent kit for P24 and NovaSeq S1 for P7) using the NovaSeq 6000 platform (Illumina). Cellranger (v.3.0.1) was used.

### Virally mediated neuron labeling

ssAAV-PHP.S/2-shortCAG-dlox-EYFP(rev)-dlox-WPRE-hGHp(A) (AAV-DIO-EYFP), ssAAV-PHP.S/2-shortCAG-dlox-tdTomato(rev)-dlox-WPRE-hGHp(A) (AAV-DIO-tdTomato) and ssAAV-PHP.S/2-shortCAG-dlox-rSyp1_EGFP-dlox-WPRE-bGHp(A) (AAV-DIO-SYP_EGFP) were constructed at the Viral Vector Facility of the Neuroscience Center Zurich (v309-PHP.S and v167-PHP.S). Plasmid pAAV-pCAG-DIO-rSyp1_EGFP was a kind gift from S. Arber. Virus was injected intravenously into the tail vein of 8–12 week-old mice. Virus titer and injected dosage are listed in Supplementary Table 5c. Animals were killed 2.5–4 weeks after injection. The procedure was different for obtaining the single-neuron morphology (‘Whole-neuron imaging’).

### Tissue preparation for histochemical analysis

#### Juvenile and adult small intestine sections

The small intestine was dissected from juvenile and adult mice, the mesentery was removed and the duodenum, jejunum and ileum were separated. The tissues were prepared as tubes or Swiss rolls. For tubes, the intestinal pieces were flushed clean with ice-cold PBS and fixed overnight in 4% paraformaldehyde (PFA) in PBS at 4 °C. Tissues were then washed three times with PBS and incubated in 30% sucrose in PBS overnight at 4 °C and embedded in Optimal Cutting Temperature (OCT) Cryomount (Histolab), snap frozen and stored at −80 °C. The tissues were cut at 20 μm or 100 μm. For Swiss rolls, briefly, the tissues were cut open along the longitudinal axis and placed flat, mucosa side down, on the bottom of a Petri dish. A toothpick was placed at one end, and the tissue was rolled up to form a roll. The rolls were pinned to a Sylgard-coated dissection dish and fixed in 4% PFA at 4 °C overnight. Tissues were then washed three times with PBS and incubated in 30% sucrose in PBS overnight at 4 °C and embedded in OCT Cryomount (Histolab), snap frozen and stored at −80 °C. The tissue was cut at 14 μm or 20 μm.

#### Submucosal plexus peels

Duodenum, jejunum and ileum intestinal segments from juvenile and adult mice were cleaned of mesentery and opened lengthwise along the mesenteric border. The intestinal contents were rinsed out with ice-cold PBS. The tissue was stretched and pinned with the mucosa side down on a Sylgard-coated dish. Longitudinal muscle-myenteric plexus-circular muscle layer was peeled off, and the remaining tissue containing submucosal plexus was fixed in 4% PFA at 4 °C overnight. Tissue was then washed three times with PBS and cut into 1 cm^2^ segments and either used directly for histochemical analysis or kept in PBS at 4 °C.

#### Postnatal small intestine sections

Small intestines were dissected out from P2–3 pups and fixed in 4% PFA in PBS at 4 °C for 2 h. Samples were subsequently washed in PBS and cryoprotected in 30% sucrose in PBS at 4 °C overnight. The tissue was then dissected into distinct anatomical regions and embedded in OCT Cryomount and kept frozen at −80 °C. Samples were sectioned at 10–12 μm.

### Immunofluorescence analysis

Frozen tissue sections were air-dried at room temperature for 1 h, rinsed with PBS and incubated in blocking solution containing donkey anti-mouse Fab (Jackson Laboratories, 715-007-003) diluted 1:50 in PBS for 1 h at room temperature. Sections were then blocked with 2% normal donkey serum (NDS; Jackson Laboratories) and 0.1% Triton X-100 (Sigma) in PBS for 1 h and incubated with primary antibodies diluted in the same solution overnight at 4 °C. They were washed three times with PBS (10 min each) and incubated with secondary antibodies at room temperature for 1 h. Tissue was washed three times with PBS (10 min each) and mounted in DAKO mounting medium (Agilent) containing DAPI (4′,6-diamidino-2-phenylindole).

Submucosal peels and 100 μm floating transverse sections were incubated in blocking solution containing donkey anti-mouse Fab diluted 1:50 in PBS and 0.3% Triton X-100 overnight at 4 °C. The blocking solution was replaced by incubation buffer containing 2% NDS, 1% BSA and 0.3% Triton X-100 in PBS for about 8 h at 4 °C. The tissue was incubated in primary antibodies (and nanobody) diluted in incubation buffer for 48 h at 4 °C, washed three times with PBS (30 min each) and then placed in the secondary antibodies diluted in incubation buffer for 2 h at room temperature. The tissue was washed three times with PBS (15 min each) and mounted on glass slides using DAKO mounting medium containing DAPI. WGA staining was performed after antibody staining using fluorescein-conjugated WGA (5 μg ml^−1^; Thermo Fisher Scientific, W834) for 30 min at room temperature, followed by PBS washing. For a complete list of primary and secondary antibodies, see Supplementary Table 5a,b.

### RNAscope

RNAscope on 20 μm sections was performed according to the manufacturer’s instructions (RNAscope Multiplex Fluorescent Reagent Kit v2 Assay; Bio-Techne). Protease III was used for tissue digestion. The following probes were used for the analysis: *Mm-Adgrg6* (467351-C2), *Mm-Nmu* (446831-C3), *Mm-Galr1* (448821-C2) and *Mm-Pkp1* (1247231-C1). Immunohistochemistry (as described above) was applied after completion of RNAscope. RNAscope was also conducted on submucosal peel tissue based on protocols from ref. ^38^. In brief, tissue was prepared as for immunohistochemistry, but villi were scraped off to allow better probe penetration. Experiments were carried out in 48-well plates. Protease IV was used for tissue digestion. Probes were incubated overnight for better hybridization.

### Confocal imaging

Imaging of z-stacks with intervals of 1 μm was performed using Zeiss LSM800 or LSM980 confocal microscopes using ×20, ×40 or ×63 objectives with Zeiss ZEN 3.3 software. The images were then processed in Fiji image analysis software (v.2.0.0-rc-69/1.52i; National Institutes of Health (NIH)). Color was, in some cases, changed, and each channel was individually adjusted. Distance analysis (Fig. 5f–i) was performed in Imaris (v10). Briefly, three-dimensional (3D) surfaces were created for enterochromaffin cells, tuft cells and nerve processes individually, and the shortest distance between enterochromaffin cells or tuft cells and nerve processes was calculated.

### Whole-neuron imaging

*Nmu-Cre* mice aged 14 weeks (males and females) were injected intraperitoneally with 100 µl of the final dose 1.5–3 × 10^11^ vector genomes PHP.S-DIO-tdTomato. Viral doses were determined in preliminary experiments to label <0.5% of enteric neurons and used to achieve sparse labeling. After 18–23 weeks, mice were killed and the ileum was collected, opened along the mesenteric border, pinned flat and fixed in 4% PFA at 4 °C for 24 h. The 5 cm segments of ileum were cleared for at least 10 days in Ce3D as described^39^ with the addition of 2.5% DABCO-33LV (Sigma) as antifading agent. Tissues were mounted on glass slides and imaged using a Nikon AXR microscope at a resolution of 0.43 × 0.43 × 0.63 µm. Nikon Elements (v.5.41.01) was used to stitch tiles, and the image was imported to the Volume Render subroutine of ANALYZE software (v.12; Mayo Foundation). Using the Trace Tool, starting with the neuron cell soma, a series of clipped volumes were set to subjective binarization thresholds until the annotator considered the nerve fibers as fully isolated objects, and these voxels were selected and manually assigned to a new object. This was repeated for every branched neurite until further fluorescence was not observed. The resulting single binarized object contained the 3D reconstructed neuron. Neurites were subjectively assigned to tissue regions, identified by autofluorescence. One reconstructed submucosal ileal Nmu^*+*^ neuron was chosen to represent several observations, as its fibers did not overlap with fibers from other labeled Nmu^+^ neurons. The 3D color-coded reconstructed neuron was rendered in different orientations to create the three-plane visualizations. In addition, sequences of renderings were generated using ANALYZE (v.14) at different orientations and zoom levels to generate video sequences.

### Epithelial deformation functional experiment

Experiments were performed on eight *Nmu-Cre;LSL-GCaMP6f* mice (six males and two females) at 18–22 weeks. Segments of the ileum were excised into normal Krebs solution with 10 μM nifedipine and 100 nM atropine to minimize contractions. Tissues were opened along the mesenteric border and pinned flat on a thin silicon elastomer in a glass bottom dish that was mounted on an inverted IX70 microscope (Olympus) equipped with an ES107 motorized stage and PS3H122R focus motor with Optiscan III controller (Prior), X-Cite X-LED1 light source (Excelitas Technologies) with QuadCube and GFP filters (Semrock), ×10 UPlanFL and ×20 and ×40 LUCPlanFLN objectives and ORCA-Flash4.0 CMOS camera (Hamamatsu) with two parallel Camera Link outputs to a Firebird Frame Grabber (Active Silicon). The 76 mm platinum iridium concentric bipolar microelectrode with a 2–3 μm tip (World Precision Instruments) was used to deliver focal mechanical stimuli from an S88 stimulator (Grass) and was positioned in X and Y with two stacked CONEX-SAG-LS16P closed-loop linear piezo stages with 16 mm range at 25 nm resolution (Newport), as well as in Z with a P-625.1CD closed-loop linear piezo stage with 500 μm range at <1 nm resolution (Physik Instrumente). Automated acquisitions (50 fps) controlled these stages to maneuver the electrode to deform 24 points in a 4 mm^2^ area over 19 min. Each epoch started with the lowering of the electrode 25 μm into the mucosa and a further lowering of 25 μm 10 s later. The electrode was raised after an additional 15 s. The baseline period of the subsequent epoch, during which the electrode was raised, lasted 20 s and overlapped with the poststimulus period. Peripherals were controlled through Clampex (v11.2) and MetaMorph (v7.10) with custom protocols. Image series were motion corrected via a template matching plugin for ImageJ Fiji and mean intensity values were extracted from manually curated ~150 μm^2^ regions of interest that were transferred to a custom JupyterLab (v3.5.3; Python 3 kernel) notebook for trace feature extraction, especially ‘spike’ detection, analysis and visualization. Cells in the submucosal or myenteric plexus were subjectively categorized into nonresponding, quiescent responding and spontaneous cells based on ongoing activity and responses during any of the 24 mucosal deformations. Note that all spontaneous cells had at least one epoch with increased firing frequency during mechanical deformation, so they could also be regarded as responsive but were not included in subsequent analysis. Epochs in quiescent responding cells were further analyzed. Each stimulus epoch was analyzed for the number of spikes and the latency to the first spike. Epoch latencies were categorized into immediate (<0.5 s from the start of the mechanical stimulus), delayed (>0.5 s after the mechanical stimulus but during the 25-s period of mechanical deformation) and poststimulus (response within 20 s after the deformation was released). Comparisons were made between submucosal (total 34 neurons) and myenteric (total 42 neurons) Nmu^+^ neurons.

### Cell counting, statistical analysis and reproducibility

Counting of fluorescent cells was performed on confocal images or directly under a Zeiss fluorescent microscope. GraphPad Prism (versions 9 and 10) was used to generate bar, dot and box-and-whisker plots. In all graphs, error bars indicate mean ± s.d. of the mean. Cell sizes were measured on z-stack images. The density plots of the shortest distance were generated using RStudio (Fig. 5f–i). No statistical methods were used to predetermine sample sizes, but sample sizes are similar to those reported in previous publications. Data collection and analysis were not performed blind to the conditions of the experiments. Samples with poor immunohistochemical staining quality were excluded from further analysis. Data distribution was assumed to be normal, but this was not formally tested.

### Analysis of scRNA-seq data

#### Clustering analysis

For the juvenile submucosal (P24) dataset, we started with 11,428 cell-containing droplets from CellRanger (3.0.1). Cells with less than 200 unique molecular identifiers (UMIs) and genes that were expressed in less than three cells were excluded. From the frequency distribution, we applied a global thresholding—(1) excluded cells with a fraction of mitochondrial genes more than 0.2, (2) retained cells with a number of genes within the range of 500–6,500 and (3) excluded cells with more than 40,000 UMIs. Given that our data also contained non-ENS cell types, a more fine-tuned filtering was required. We performed clustering across a range of resolutions (0.2, 0.6, 1.0 and 1.8), resulting in plausible clusters, from each of which we removed cells with the total number of UMIs below the 1st percentile and above the 99th percentile. This step helped further eliminate unhealthy cells and homotypic doublets or triplets and was followed by the usage of the DoubletFinder (v2.0.3) algorithm. After these procedures, we retained 9,515 nonambiguous high-quality cells for further analysis. Initially, we normalized the expression data using the regularized negative binomial regression method implemented in SCTransform(). For principal component analysis (PCA), we used 3,000 variable genes and excluded sex-specific genes (*Xist*, *Gm13305*, *Tsix*, *Gm8730*, *Eif253y*, *Ddx3y*, *Uty* and *Kdm5d*) from the variable genes to prevent artifacts in the dimensionality reduction. However, these genes were retained for subsequent analyses to ensure their inclusion in the dataset. In the PCA space, we constructed a *K*-nearest neighbor graph based on Euclidean distance and identified distinct cell communities using the Louvain algorithm (res. = 1.8). From here, we performed iterative clustering and excluded non-ENS cells at each round. After each round, the raw data containing more purified enteric neurons were analyzed again. At level 1, we removed non-ENS clusters (Extended Data Fig. 1a–e). In further iterations, we identified and eliminated clusters with glial identity (*Sox10*) and suspected contaminating myenteric cells based on their similarity in DE genes to major myenteric plexus clusters, their small fraction compared to other clusters in the dataset and validating histochemical experiments (Extended Data Fig. 1f–j). Finally, only submucosal neurons (8,341) underwent third-level 30 principal component (PC) clustering, where res. = 0.04 resulted in three cardinal clusters and res. = 0.4 resulted in nine clusters. Visualization of the clusters was performed on two dimensions using the RunUMAP() function (min.dist = 0.5, *n*_neighbors = 30L, umap.method = ‘umap-learn’, metric = ‘correlation’).

Likewise, for the postnatal day (P) 7 datasets, we analyzed 8,179 starting cells. After removing cells with more than 0.1 fraction of mitochondrial genes, with more than 6,500 and less than 500 genes and with more than 40,000 UMI counts, we performed first-level clustering of the remaining 6,062 cells in the same manner as we did for the juvenile dataset. Here we excluded sex-specific genes (*Xist*, *Gm13305*, *Tsix*, *Gm8730*, *Eif253y*, *Ddx3y*, *Uty* and *Kdm5d*) and a set of immediate early genes (*Fos*, *Jun*, *Junb* and *Egr1*) from variable genes before computing PCAs. We then constructed a *K*-nearest neighbor graph and identified communities of cells using the Louvain algorithm. At this step, we refined the cell population by removing five non-ENS clusters. One cluster corresponded to fibroblast-like cells described previously in embryonic scRNA-seq in ref. ^15^. One cluster likely constituted myocytes (*Myh11*, *Cnn1* and *Ckm*); three other clusters corresponded to hematopoietic (*Lcp1*), endothelial (*Lyve1* and *Cldn5*) and epithelial (*Muc13*) cells. The remaining ENS cells (5,740) were then subjected to SCTransform() and second-level clustering (50 PCs, res. = 1.8), yielding 24 clusters. We then performed differential expression analysis based on DE genes, categorizing each cluster as one of the following major cell types: neuron (*Elavl4*^*high*^), neuroblast (*Ascl1*^high^), dividing progenitors (for example, *Cenpa* and *Top2a*), enteric glia (*S100b* and *Plp1*) and SCP cells (*Dhh*, *Col14a1* and *Gfra3*). Clusters considered to share a generic differentiation state were merged (Fig. 6b). The clusters were visualized in two dimensions using the RunUMAP() function (min.dist = 0.4, *n*_neighbors = 60L, umap.method = ‘umap-learn’, metric = ‘correlation’).

#### Sex inference, dataset integration and identity mapping

Sex inference (predicting the sex of cells in the scRNA-seq datasets) was performed using classifySex() in the Oshlack/speckle (v0.0.3) package^40^. The classifier had been trained on mouse data. Cells with zero counts on *Xist* and the sum of the Y chromosome genes were annotated as N/As. To integrate different scRNA-seq datasets we used the raw counts from myenteric neurons^15^ and from our submucosal neurons (myENC1–myENC12 and smENC1, smENC2a–smENC2d; smENC3a–smENC3d) for integration analysis. Each dataset was normalized separately using SCTransform() to obtain 3,000 variable genes. Subsequently, we merged the two lists of variable genes from each dataset to obtain a shared set of variable genes, which were then used to compute PCAs (50 PCs) in the Harmony algorithm. Cells in this integrated reduced space were visualized using uniform manifold approximation and projection (UMAP; min.dist = 0.4, *n*_neighbors = 45L, umap.method = ‘uwot’, metric = ‘cosine’; Fig. 2a). Label transfer was performed using the FindTransferAnchors() function followed by the TransferData() function, with myENC1–myENC12 as the reference dataset. Finally, the mapping identities were presented as Alluvial plot using the R package ggalluvial (Fig. 2b). For label transfer to the P7 dataset, we first built a reference dataset by combining our previously published myenteric neuron dataset^15^ with the submucosal neuron dataset, as well as enteric glia^16^, representing the most comprehensive mature reference dataset so far. After merging the three datasets from the mature ENS, we performed SCTransform (method = ‘glmGamPoi’). Using 3,000 variable genes, we computed PCAs and built a reference reduced space (100 PCs). We retained annotations for published myenteric neuron classes and submucosal classes (as defined in this study) as labels for transfer to the P7 dataset. For this, we used the FindTransferAnchors() function and then the TransferData() function (with smENC1–smENC3, myENC1–myENC5, myENC10, myENC12 and enteric glia as labels). To avoid competition between transcriptionally similar neuron classes, myENC5, myENC6 and myENC11 were masked from the reference dataset when performing label transfer. P7 cells were assigned according to their maximum predicted score (Fig. 6i).

#### Ligand–receptor analysis

To infer possible signaling communication between submucosal neuron classes and between these neurons and intestinal epithelial cell types, we performed ligand–receptor analysis with CellChat (v2)^41,42^. Epithelial cell clusters were retrieved from the scRNA-seq dataset GSE92332 (ref. ^21^). CellChat identifies overexpressed ligands or receptors in one cell group and then identifies overexpressed ligand–receptor interactions if either ligand or receptor is overexpressed. Based on the extent of overexpression combined with prior knowledge of the interactions between signaling molecules (ligands, receptors and their cofactors), CellChat determines and weights biologically significant cell–cell communication. We then calculated the overexpressed genes and the corresponding interactions using the CellChat functions identifyOverExpressedGenes() and identifyOverExpressedInteractions(), respectively. To identify the significant interactions between submucosal neurons (smENC–smENC), we used 20% truncated means, while 10% truncated means were used to identify smENC–epithelial cell communications. A ten percent threshold was used to detect ligands expressed in epithelial cell clusters that were small and heterogeneous (for example, enterochromaffin cells). Circle plot was generated using netVisual_circle() to show the total number of interactions among all cell groups. The communication probability of interactions was calculated using computeCommunProb(). Significant interactions (L–R pairs) from senders to the receiver cell groups were plotted using netVisual_bubble().

### Reporting summary

Further information on research design is available in the Nature Portfolio Reporting Summary linked to this article.

## Online content

Any methods, additional references, Nature Portfolio reporting summaries, source data, extended data, supplementary information, acknowledgements, peer review information; details of author contributions and competing interests; and statements of data and code availability are available at 10.1038/s41593-025-01962-x.

## Supplementary information

Supplementary Information Supplementary Figs. 1–8.

Reporting Summary

Supplementary Data Supporting data for Supplementary Figs. 1, 4 and 5.

Supplementary Video 1 Video depicting single-cell 3D reconstruction of a smENC1 neuron imaged in the ileum of an *Nmu-Cre* mouse, injected with AAV-DIO-tdTom. Color-coding of fibers indicates their estimated location within the submucosa–mucosa space (Extended Data Fig. 4a).

Supplementary Video 2 Video depicting 3D stack of ileal villi imaged in a *Nmu-Cre* x *R26RtdTom* mouse, immunostained for capillaries (CD31^+^LYVE1^−^) and the central lacteal (CD31^+^LYVE1^+^; Fig. 5o).

Supplementary Video 3 Video depicting 3D stack of ileal villi imaged in a *Sst-Cre* x *R26RtdTom* mouse, immunostained for capillaries (CD31^+^LYVE1^−^) and the central lacteal (CD31^+^LYVE1^+^; Fig. 5o).

Supplementary Video 4 Video depicting 3D stack of ileal villi imaged in a *Vip-Cre* x *R26RtdTom* mouse, immunostained for capillaries (CD31^+^LYVE1^−^) and the central lacteal (CD31^+^LYVE1^+^; Fig. 5o).

Supplementary Table 1 DE genes in level 1 clusters of P24 submucosa.

Supplementary Table 2 DE genes in smENC1–smENC3.

Supplementary Table 3 DE genes in subclusters of smENC1–smENC3.

Supplementary Table 4 DE genes in P7 submucosal ENS clusters.

Supplementary Table 5 Primary antibodies, secondary antibodies and AAV virus used in the study.

## Source data

Source Data Figs. 2, 3, 5 and 7 Statistical source data.

Source Data Extended Data Figs. 4 and 10 Statistical source data.

## Data Availability

Raw sequence and processed data for submucosal small intestine *Baf53b-Cre;R26RtdTom* (P24) and small intestine *Wnt1-Cre;R26RtdTom* (P7) are available on the Gene Expression Omnibus database under the identifier GSE263422. We have additionally used mouse small intestine epithelial cell scRNA-seq from GSE92334 (ref. ^21^), myenteric mouse small intestine neuron scRNA-seq from GSE149524 (ref. ^15^) and glial cells from mouse small intestine myenteric scRNA-seq from SRP135960 (ref. ^16^). Source data are provided with this paper.

## Code availability

The data analysis code for P24 and P7 is available at https://github.com/UMarklundLab/SubmucosaENS. Code used for calcium imaging analysis is available at https://github.com/Linden-Laboratory/Mucosal-Deformation.
