# Supplementary material for: The transcriptomes, connections and development of submucosal neuron classes in the mouse small intestine
Source: Nat Neurosci. 2025 May 29;28(6):1146–59. doi: 10.1038/s41593-025-01962-x (PMC12148937; doi:10.1038/s41593-025-01962-x)
Supplement: Supplementary file 1 — Supplementary Figs. 1–8. [file 41593_2025_1962_MOESM1_ESM.pdf]

nature neuroscience

Supplementary information https://doi.org/10.1038/s41593-025-01962-x

The transcriptomes, connections and development of submucosal neuron classes in the mouse small intestine

In the format provided by the authors and unedited

Supplementary Figure 1

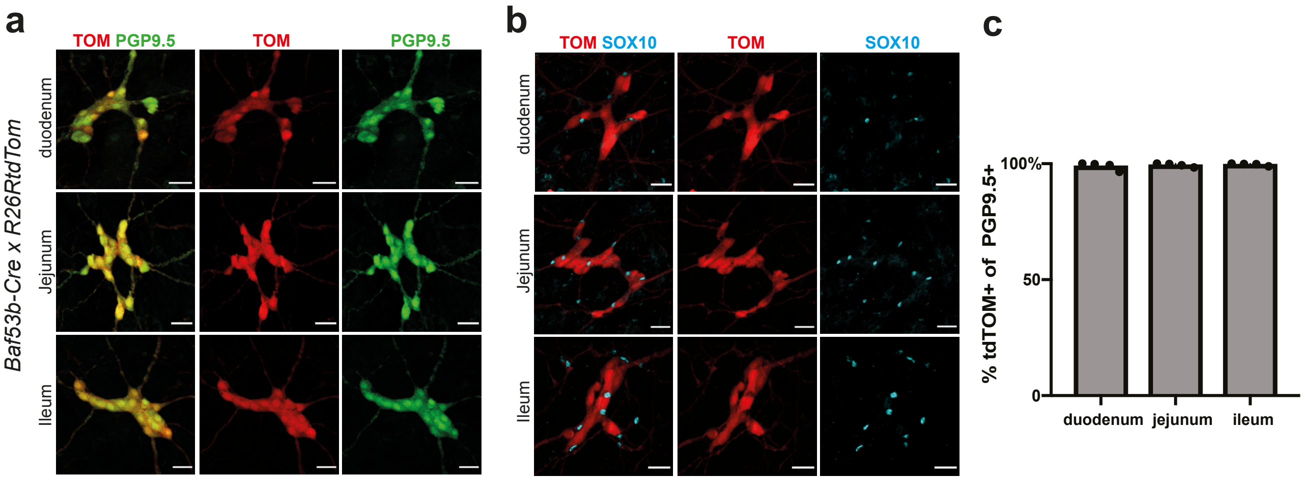

Supplementary Figure 1: Validation of tdTomato expression in submucosal neurons of *Baf53b-Cre x R26RtdTomato* mice.
Representative pictures from submucosal plexus of duodenum, jejunum and ileum of P24 *Baf53b-Cre;R26R-Tomato* mice, showing tdTomato expression in neurons (**a**) but not in enteric glia (**b**). **c**, Graph showing the average percentage of PGP9.5+ cells that express tdTomato within the duodenum (98.9±1.64;2875 cells), jejunum (99.29±0.72; 2575 cells) and ileum (99.64±0.50; 2585 cells); n=4 mice;TOM:tdTomato; Scale bars:30µm

Supplementary Figure 2

a) Ion Channels

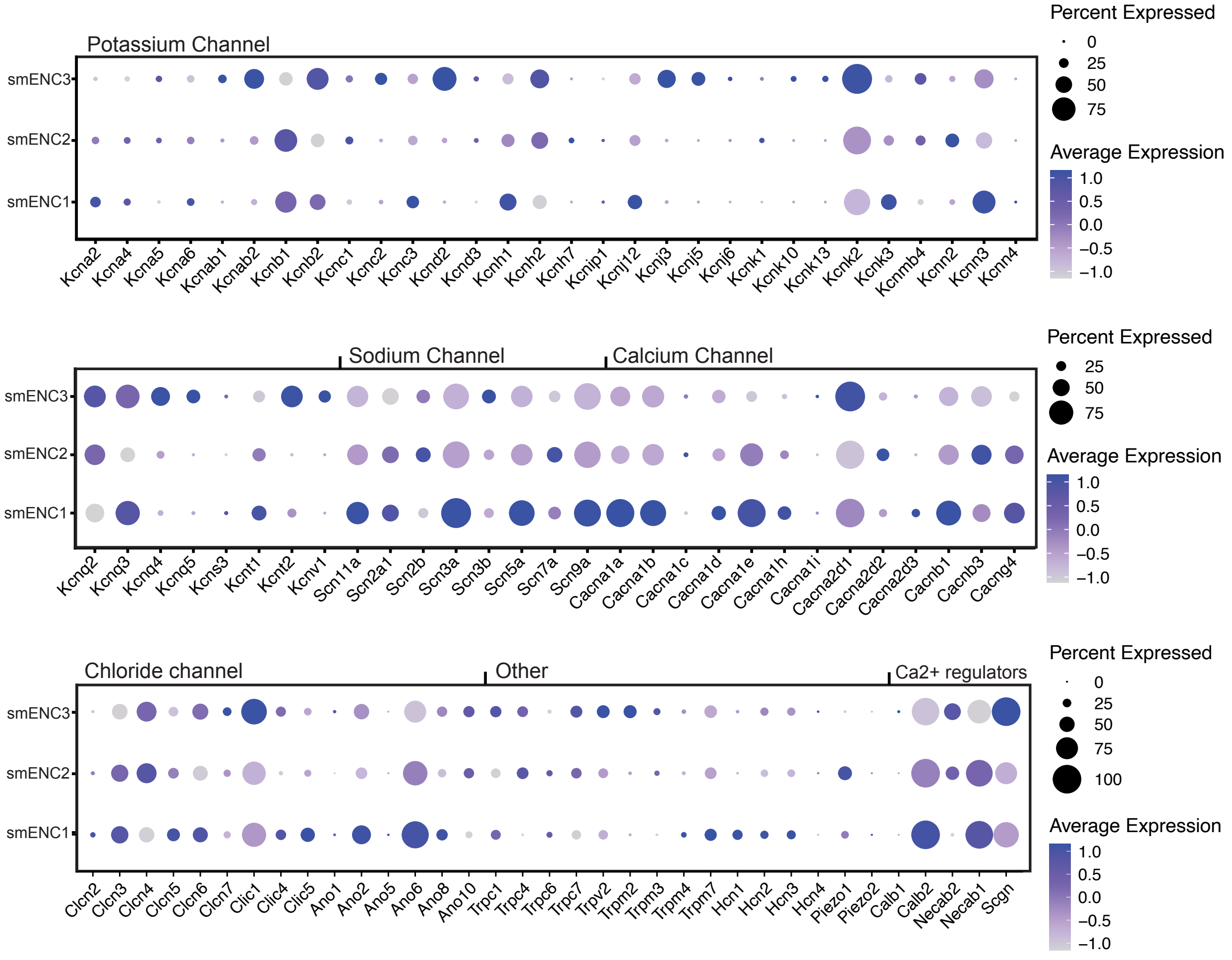

b) Membrane Trafficking

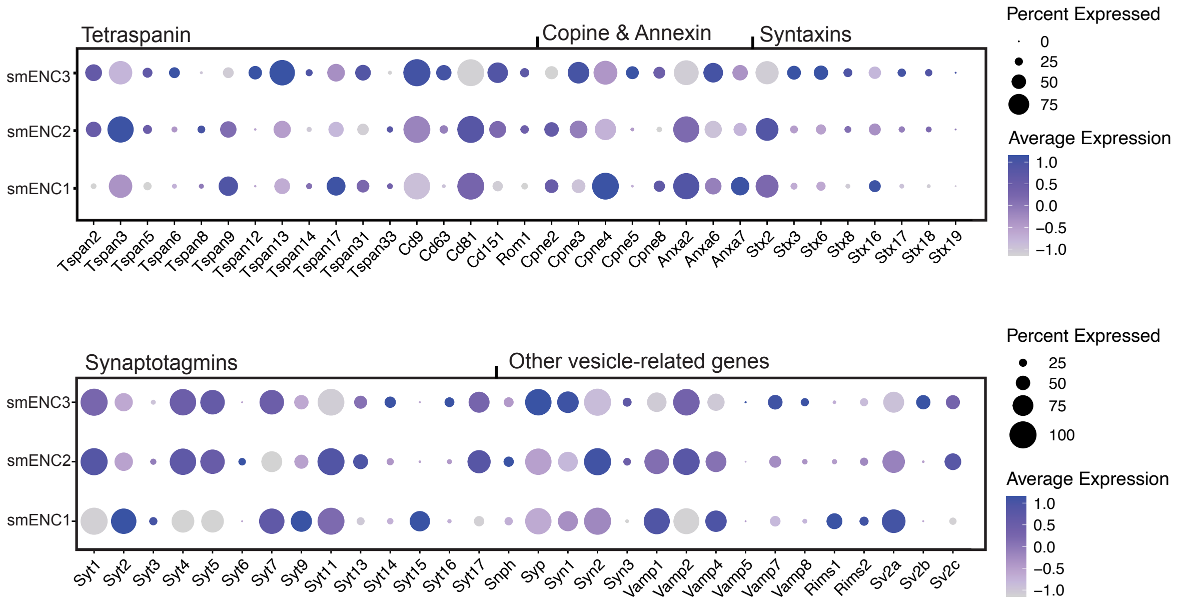

c) Adhesion molecules

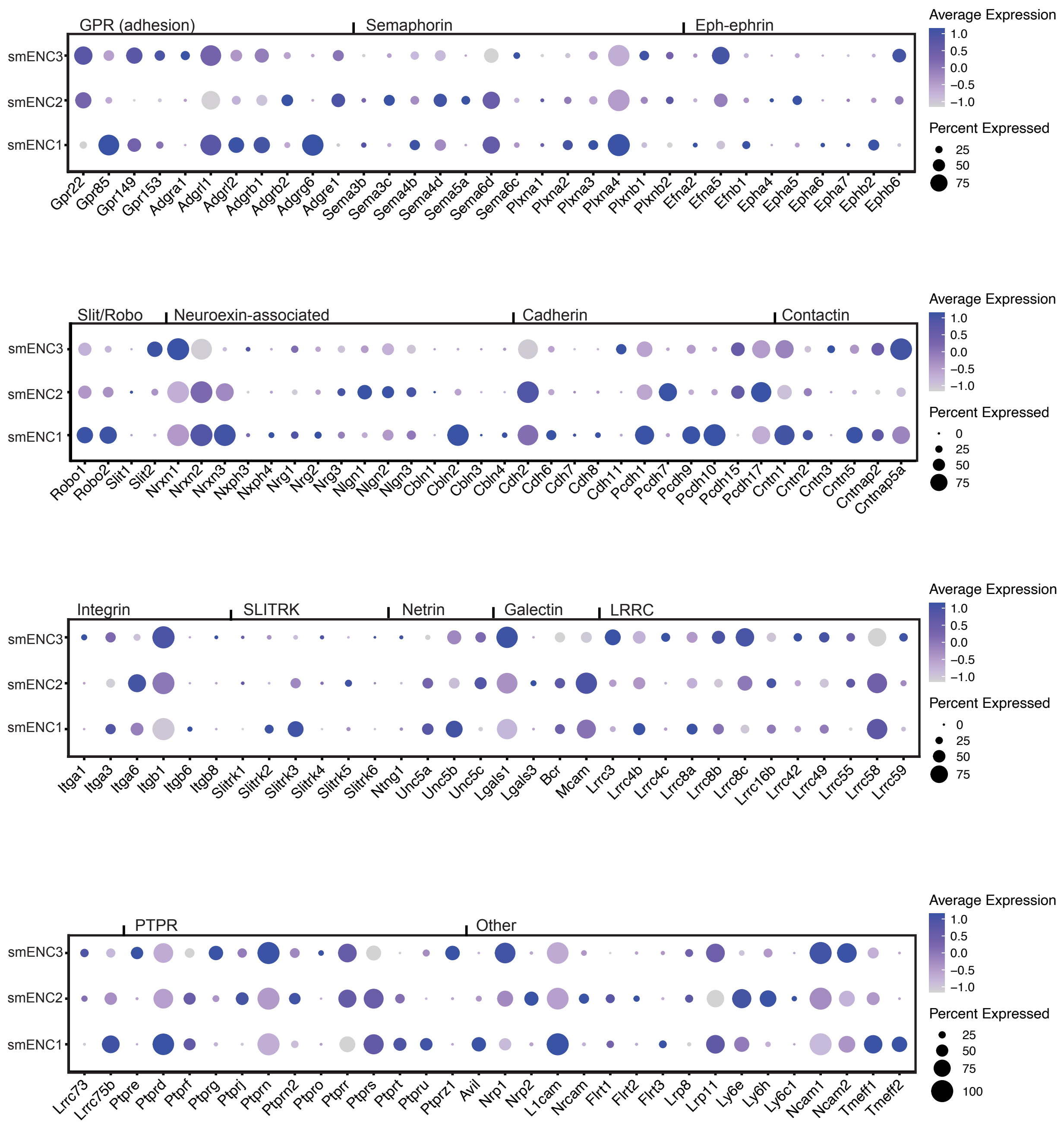

d) Transcription Factors

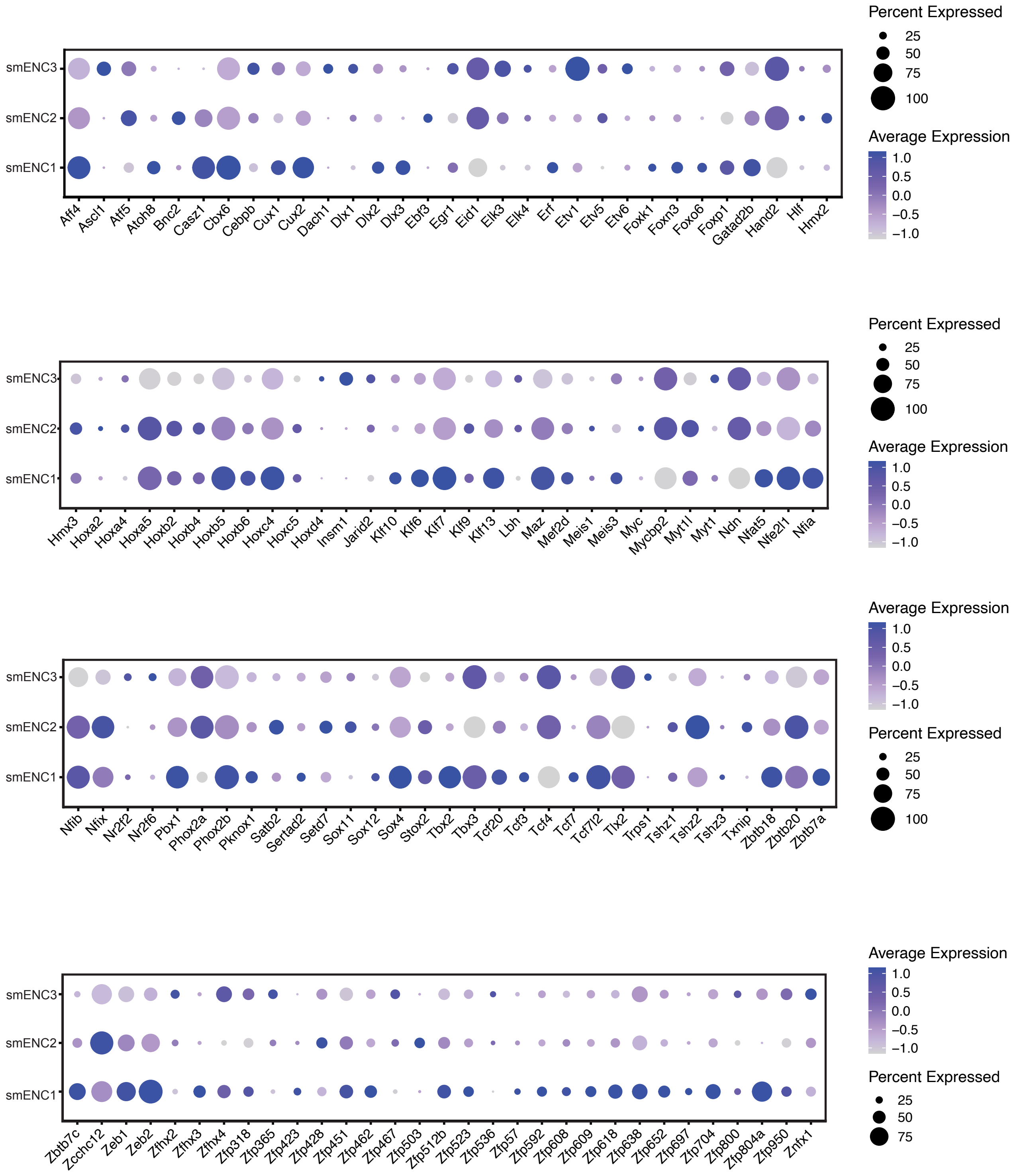

e) Neurotransmission

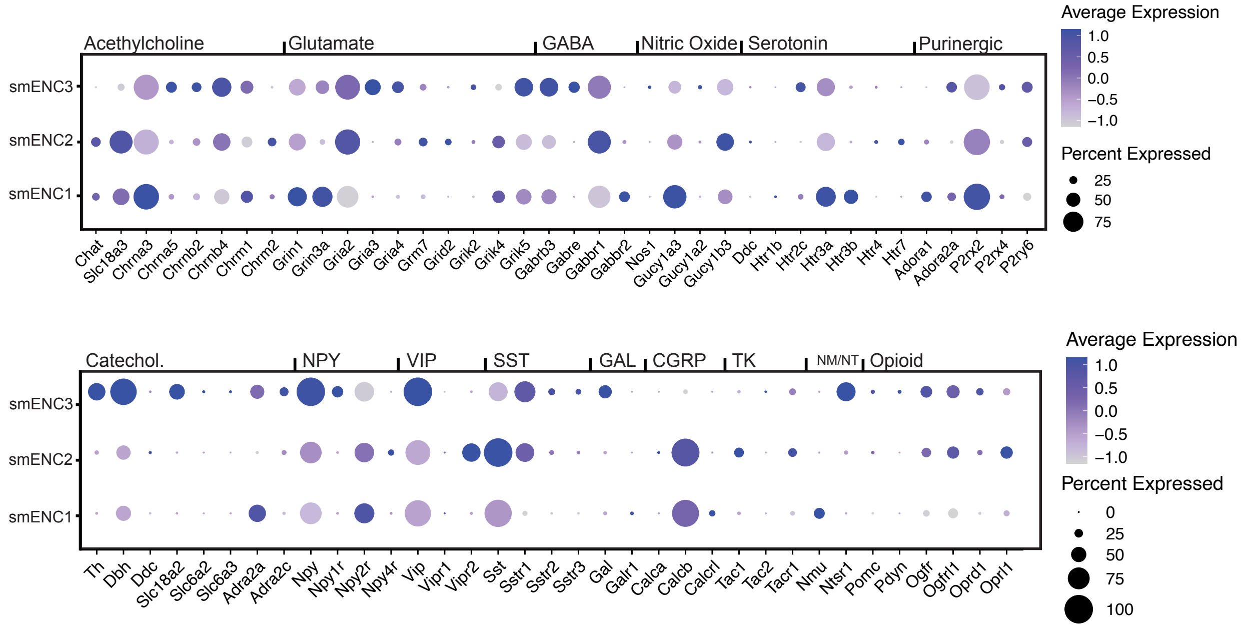

f) Cell-cell signaling

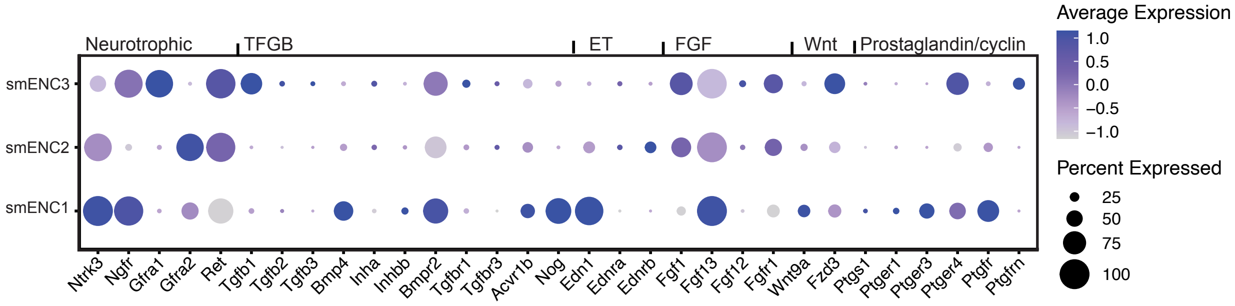

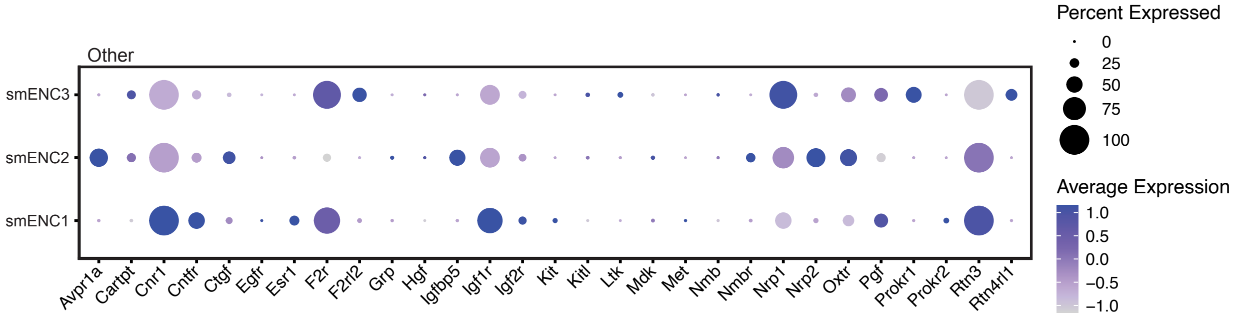

Supplementary Figure 2. Dot plots displaying expression of genes conferring neuronal phenotypes in smENC1-3.
Gene categories involved in a) ion transport, b) membrane trafficking, c) adhesion d) transcription e) neurotransmission f) other cell-cell signaling. Color scale represents z-score and dot size represents percent of cells with non-zero expression within a given class. smENC: submucosal Enteric Neuron Class; GPR: G-protein coupled receptor; GABA: Gamma-aminobutyric acid; NPY:Neuropeptide Y; TK: Tachykinin; CGRP: Calcitonin gene-related peptide; GAL: Galanin; VIP: Vasoactive intestinal peptide; SST: Somatostatin; NM: Neuromedin U; NT: Neurotensin; CCK: Cholecystokinin; TGFB: Transforming growth factor beta; ET: Endothelin; FGF: Fibroblast growth factor; LRRC: Leucine Rich Repeat Containing;
PTPR: Protein tyrosine phosphatase receptor.

Supplementary Figure 3

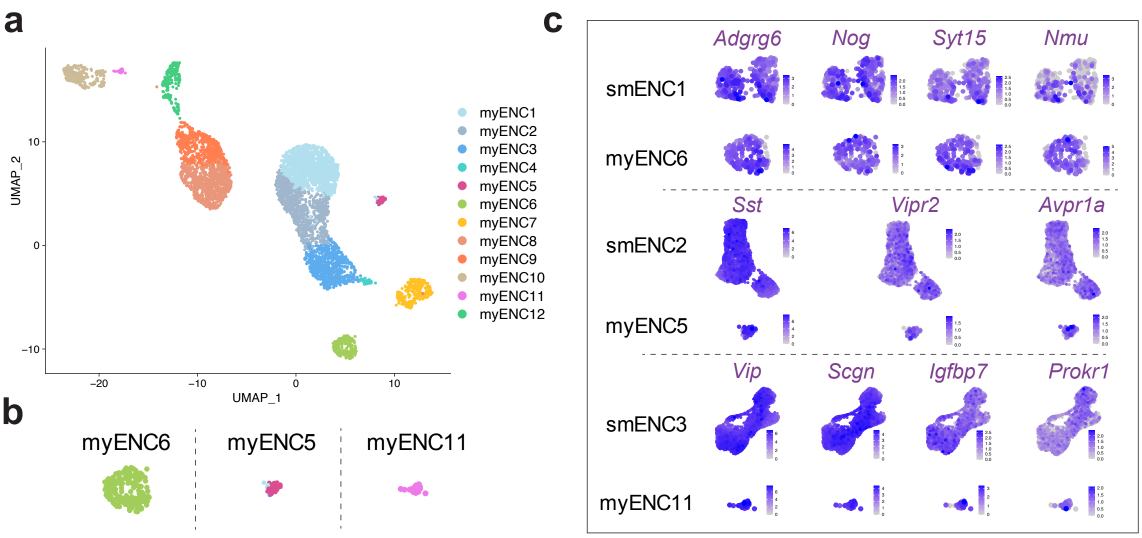

Supplementary Figure 3. Supportive Data related to Figure 2.
a, UMAP representing scRNA-seq analysis of neurons in the juvenile myenteric plexus (Morarach et al., 2021). **b,** Closeup of the three myenteric clusters that are transcriptionally similar to submucosal clusters. **c**, Feature plot assembly showing examples of gene expression that is shared between transcriptionally similar smENCs and myENCs.

Supplementary Figure 4

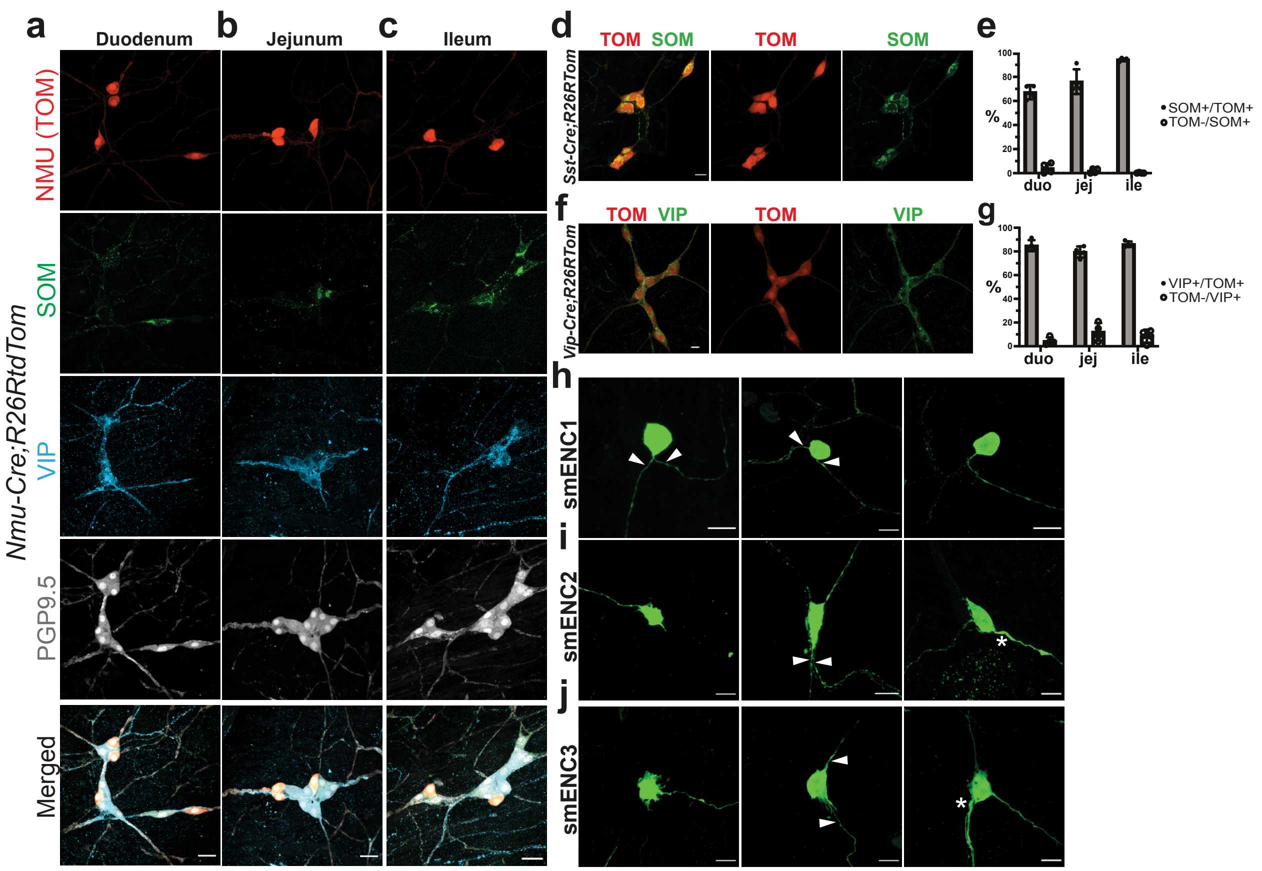

Supplementary Figure 4. Supportive Data related to Figure 3.
a-c, Representative images showing smENC1(*Nmu-Cre;R26RTomato*), smENC2 (SOM) or smENC3 (VIP) in duodenum (**a**), jejunum (**b**) and ileum (**c**). Scale bars: 30µm.**d,** Representative pictures from ileum submucosal peels showing colocalization of SOM and tdTomato in *Sst-Cre;R26RtdTomato* mice. **e,** Graph showing the percentage of specificity (%SOM+/TOM+) and efficiency (%TOM-/SOM+) of *Sst-Cre;R26RtdTomato* mice (n=4 mice) **f,** Representative pictures from ileum submucosal peels showing colocalization of VIP and tdTomato in *Vip-Cre;R26RtdTomato* mice. **g,** Graph showing the specificity (%VIP+/TOM+) and efficiency(%TOM-/VIP+) of *Vip-Cre;R26RtdTomato mice* (n=4 mice). **h-j**, Images showing examples of neuron morphologies of smENC1-3. Scale bars:20µm. Data are presented as mean values±SD. Arrowheads indicate multiple axons emanating from a single soma.
Stars indicate irregular axonal start. duo:duodenum; jej:jejenum; ile: ileum; TOM:tdTomato

Supplementary Figure 5

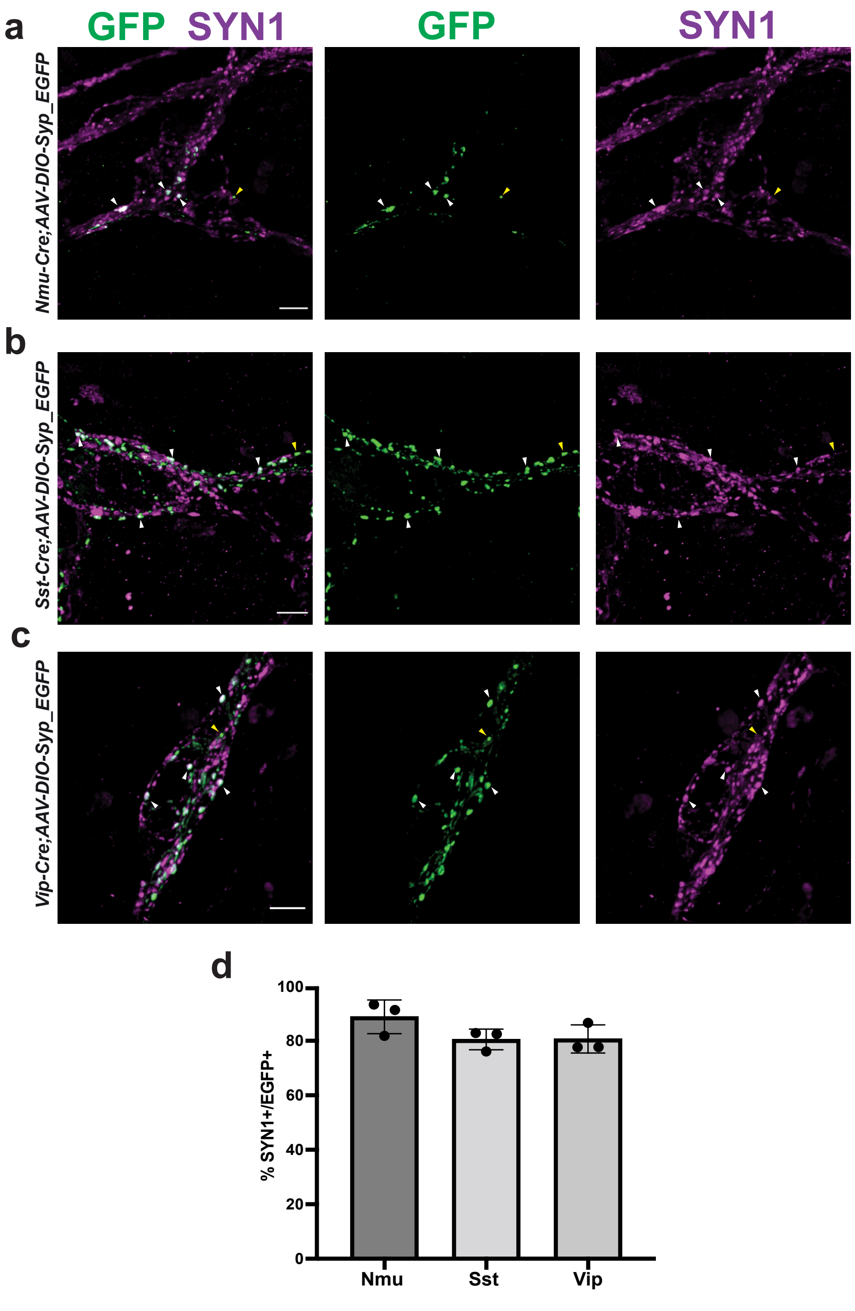

Supplementary Figure 5. Validation of Syp-EGFP labeling in SYN1+ puncta in the class Cre mouse lines. a,b,c. Representative pictures showing colocalization of SYN1 and Syp-EGFP in *Nmu-Cre, Sst-Cre* and *Vip-Cre* mice injected with AAV-PHP.S-DIO-SYP-EGFP. Scale bars:10μm. White arrowhead: SYN1+ Syp-EGFP+ puncta, yellow arrowhead: SYN1- Syp-EGFP+ puncta. **d**, Graph showing the percentage of colocalization (n=3 mice). Data are presented as mean values±SD. SYN1: Synapsin1**.**

Supplementary Figure 6

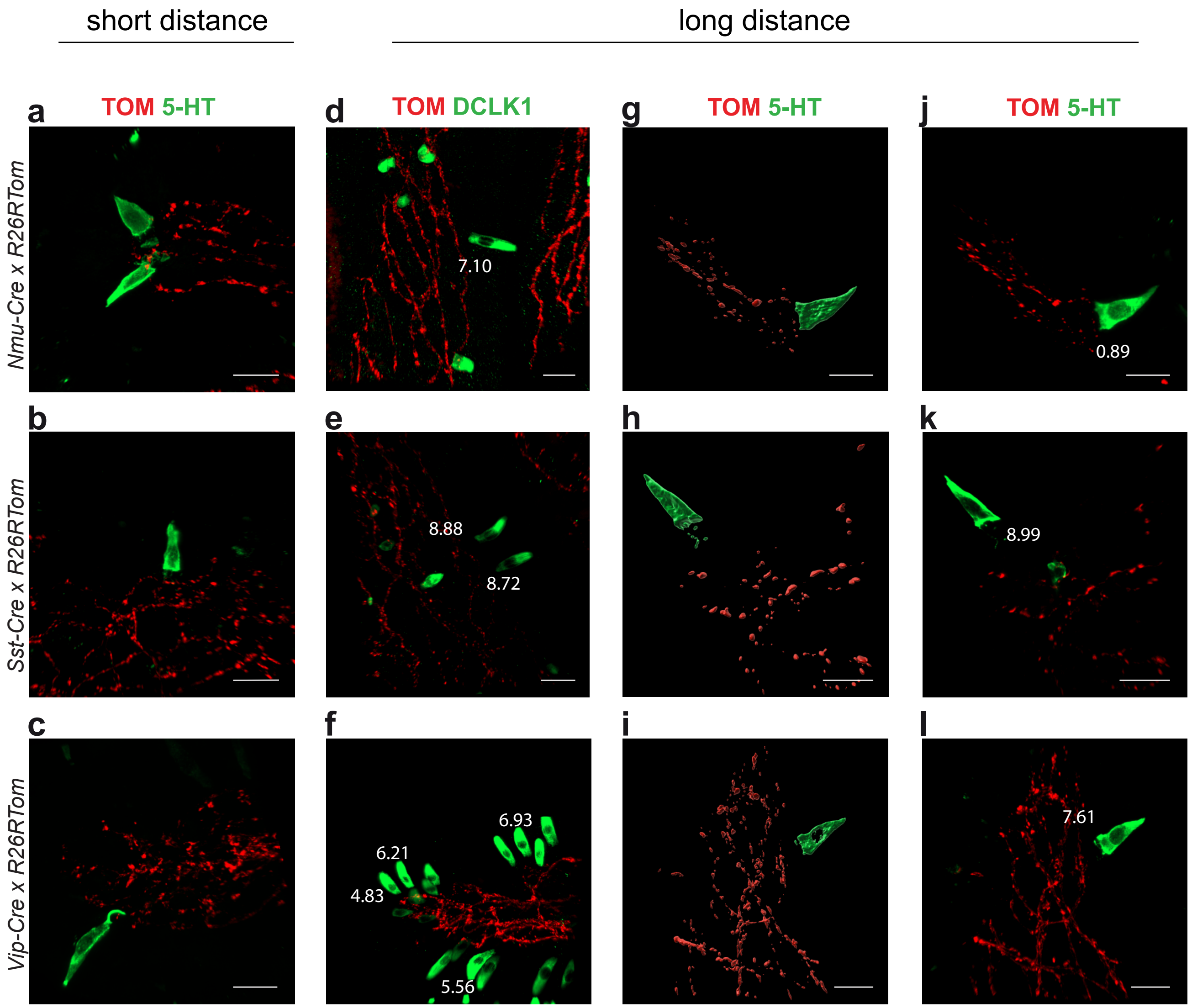

Supplementary Figure 6. Supportive Data to Figure 5.
a-f, Original confocal images of Imaris representations shown in Fig. 5d and e.
g-l, Examples of enterochromaffin cells with the nerve processes from smENCs at a long distance as Imaris representations (g-i) and original confocal images (j-l). Distances are displayed in μm. Scale bars: 20μm.

Supplementary Figure 7

a

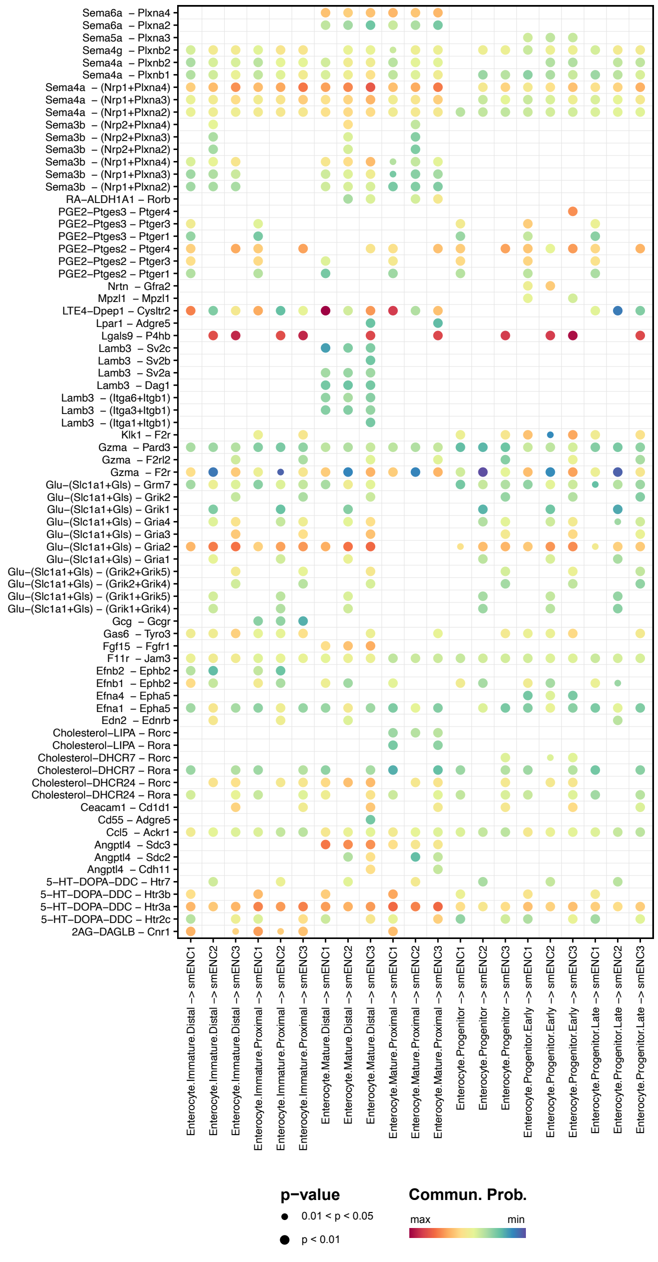

b

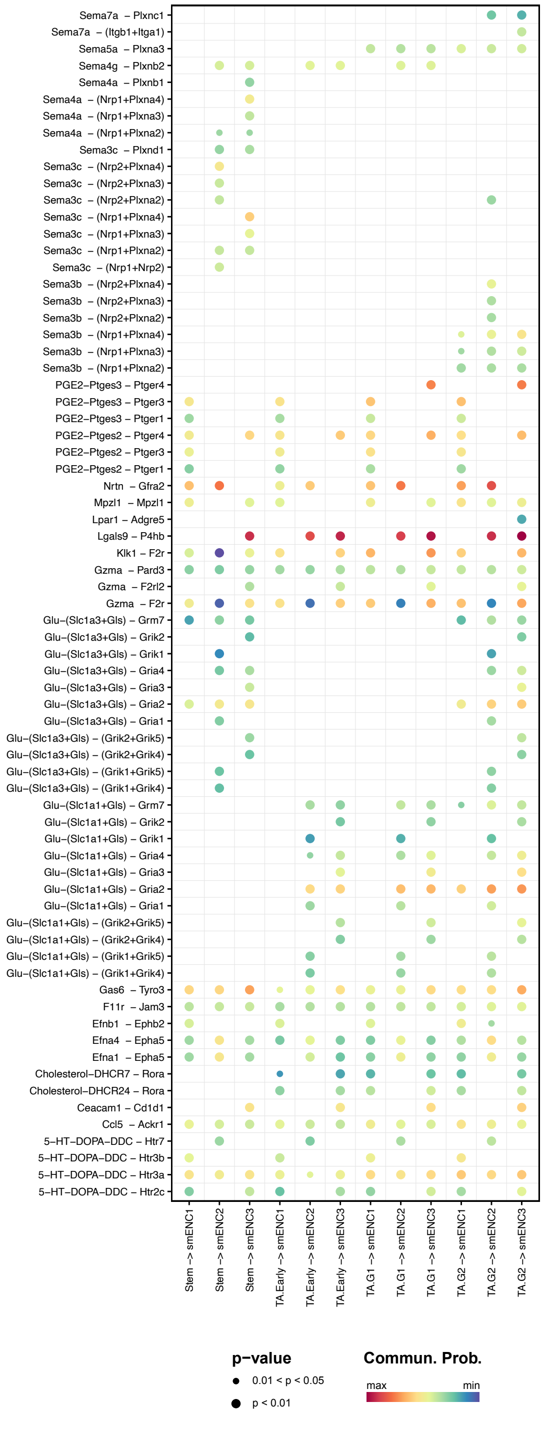

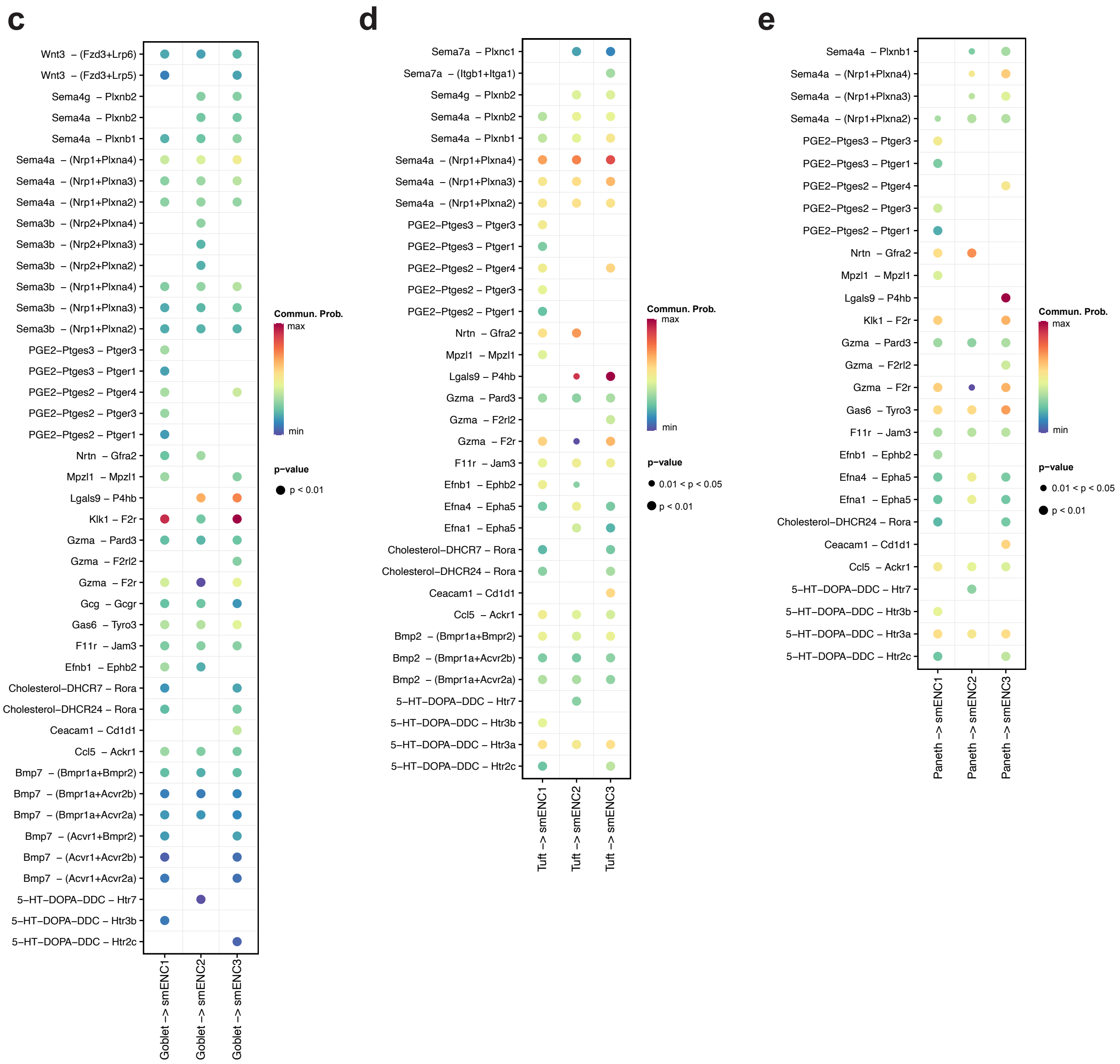

Supplementary Figure 7: Cell Chat analysis addressing ligand-receptor communication between epithelial cell types and smENC1-3. Related to Fig. 5.
Dot plots showing interaction from **a,** enterocytes; **b,** stem and TA cells; **c,** goblet cells; **d,** tuft cells; or **e,** Paneth cells to smENC1-3. The dot colour shows the relative score of communication probability value of each interaction pair. The dot size indicates the level of significance of the interaction.
Note: Dot plots for smENC1-3 or enteroendocrine cells as senders can be viewed in Extended Data Fig. 7.

Supplementary Figure 8

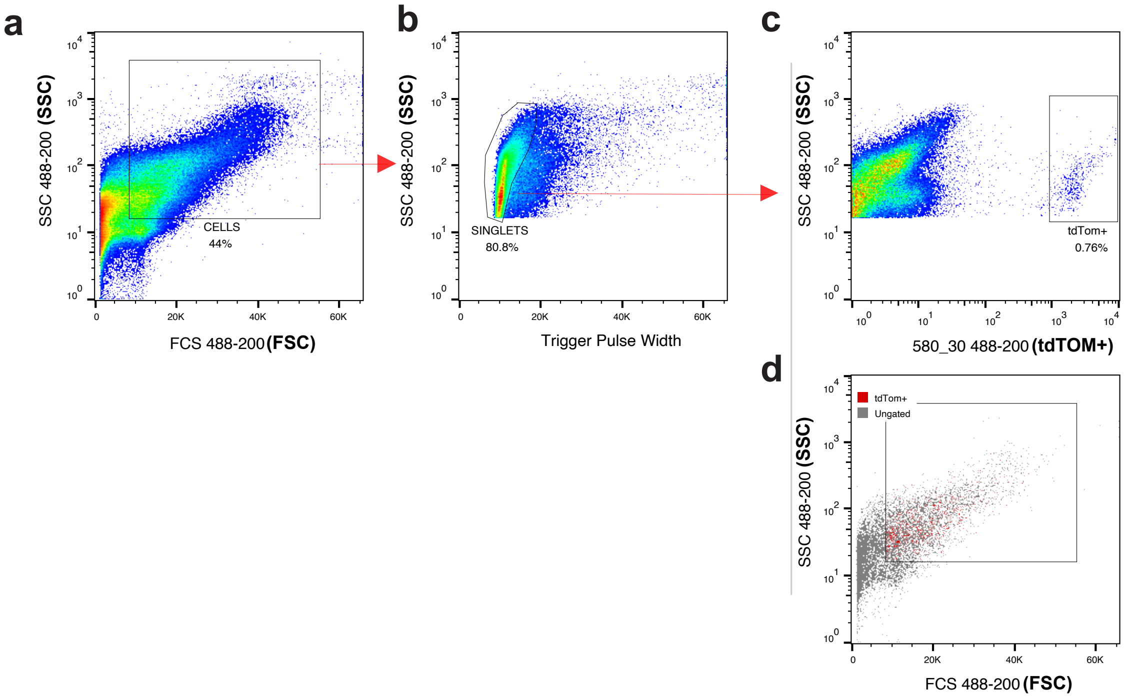

Supplementary Figure 8: Representative Flow Cytometry Data Plots.
Plots show gating strategy for sorting tdTom+ cells from *Baf53b-Cre;R26RtdTom* mice at P24. **a,** Forward vs Side Scatter plot gating on cells **b,** Side Scatter vs Trigger Pulse Width to gate out doublets and multiplets. **c,** Red fluorescence (580/30) to distinguish TOM+ cells from autofluorescent cells. **d,** Plot of cells indicating sorted (red, tdTom+) versus non-sorted cells (grey, ungated).
SSC: Side-scattered light; FSC: Forward-scattered light
