# Supplementary material for: The transcriptomes, connections and development of submucosal neuron classes in the mouse small intestine
Source: Nat Neurosci. 2025 May 29;28(6):1146–59. doi: 10.1038/s41593-025-01962-x (PMC12148937; doi:10.1038/s41593-025-01962-x)
Supplement: Supplementary file 2 — Reporting Summary [file 41593_2025_1962_MOESM2_ESM.pdf]

nature portfolio

Corresponding author(s): Ulrika Marklund

Last updated by author(s): Mar 17, 2025

Reporting Summary

Nature Portfolio wishes to improve the reproducibility of the work that we publish. This form provides structure for consistency and transparency in reporting. For further information on Nature Portfolio policies, see our Editorial Policies and the Editorial Policy Checklist.

Statistics

For all statistical analyses, confirm that the following items are present in the figure legend, table legend, main text, or Methods section.

n/a	Confirmed	
☐	☒	The exact sample size (*n*) for each experimental group/condition, given as a discrete number and unit of measurement
☐	☒	A statement on whether measurements were taken from distinct samples or whether the same sample was measured repeatedly
☐	☒	The statistical test(s) used AND whether they are one- or two-sided<br>*Only common tests should be described solely by name; describe more complex techniques in the Methods section.*
☐	☒	A description of all covariates tested
☐	☒	A description of any assumptions or corrections, such as tests of normality and adjustment for multiple comparisons
☐	☒	A full description of the statistical parameters including central tendency (e.g. means) or other basic estimates (e.g. regression coefficient) AND variation (e.g. standard deviation) or associated estimates of uncertainty (e.g. confidence intervals)
☐	☒	For null hypothesis testing, the test statistic (e.g. *F*, *t*, *r*) with confidence intervals, effect sizes, degrees of freedom and *P* value noted<br>*Give P values as exact values whenever suitable.*
☒	☐	For Bayesian analysis, information on the choice of priors and Markov chain Monte Carlo settings
☒	☐	For hierarchical and complex designs, identification of the appropriate level for tests and full reporting of outcomes
☒	☐	Estimates of effect sizes (e.g. Cohen's *d*, Pearson's *r*), indicating how they were calculated

Our web collection on statistics for biologists contains articles on many of the points above.

Software and code

Policy information about availability of computer code

Data collection	Single cell RNA sequencing: Cellranger version version 3.0.1 for postnatal day (P)7 and P24 dataset.<br>Image acqusition: Zeiss ZEN 3.3, Nikon Elements software(v.5.41.01), ANALYZE software (v.12;v.14 Mayo Foundation), Clampex (v11.2.) and MetaMorph (v7.10).<br>Biorender.com (premium plan license) was used to create part of figures. Figure 1a: https://BioRender.com/h62a257; https://BioRender.com/y27u756; Figure 3d, Extended Data Fig. 5b: https://BioRender.com/x22c309; Extended Data Figure 4b: https://BioRender.com/s52d750; Figure 5a: https://BioRender.com/p26i878, https://BioRender.com/q20b527; Figure 6a: https://BioRender.com/c31n296; Figure 7i: https://BioRender.com/a15a001.
Data analysis	For single cell data analysis we used seurat (v4.1.3), ggplot (v3.5.1), Oshlack/speckle (v0.0.3), and DoubletFInder (v2.0.3). We used CellChat v2 for ligand-receptor analysis. For Immunofluorescent data analysis we used Fiji (ImageJ) version2.0.0-rc-69/1.52p and Imaris (v10). For statistical testing of cell counts we used Prism Graphpad version (v9 or 10) (Fig. 2g,i,l. 3b,c,g,j,m,n. 5f,h. Fig. 7e,h,l,n,o; Supplementary figure 1c. 4e,g. 5d ; Extended Data Fig 4c,e,h,i,j. 10b,c,f,g)<br>Calcium indicator analysis: JupyterLab (v3.5.3), described here: https://github.com/Linden-Laboratory/Mucosal-Deformation<br>Computational analysis of scRNA-seq data can be found here: https://github.com/UMarklundLab/SubmucosaENS

For manuscripts utilizing custom algorithms or software that are central to the research but not yet described in published literature, software must be made available to editors and reviewers. We strongly encourage code deposition in a community repository (e.g. GitHub). See the Nature Portfolio guidelines for submitting code & software for further information.

Data

Policy information about availability of data

All manuscripts must include a data availability statement. This statement should provide the following information, where applicable:

- Accession codes, unique identifiers, or web links for publicly available datasets
- A description of any restrictions on data availability
- For clinical datasets or third party data, please ensure that the statement adheres to our policy

Raw sequencing data and processed expression matrix used for analysis in this paper are accessible on the GEO using accession number GSE263422.
Analysis for Figure 1 and 2 was based on the raw data file P24_1*, P24_2* and P24_3*. Seurat Object of processed data: sm_p24_lv3.rds.gz
Analysis for Figure 6 and 7 was based on the raw data file P7_1* and P7_2*. Seurat Object of processed data: ens_p7_lv2.rds.gz

Research involving human participants, their data, or biological material

Policy information about studies with human participants or human data. See also policy information about sex, gender (identity/presentation), and sexual orientation and race, ethnicity and racism.

Reporting on sex and gender	N/A
Reporting on race, ethnicity, or other socially relevant groupings	N/A
Population characteristics	N/A
Recruitment	N/A
Ethics oversight	N/A

Note that full information on the approval of the study protocol must also be provided in the manuscript.

Field-specific reporting

Please select the one below that is the best fit for your research. If you are not sure, read the appropriate sections before making your selection.

☒ Life sciences ☐ Behavioural & social sciences ☐ Ecological, evolutionary & environmental sciences

For a reference copy of the document with all sections, see nature.com/documents/nr-reporting-summary-flat.pdf

Life sciences study design

All studies must disclose on these points even when the disclosure is negative.

Sample size	No sample size calculation was performed. Previous publications (PMID:33288908; PMID:36070775) in the field and own experience were used to determine approximate number of cells and animals needed for experiments. A minimum of three animals were used per experiment with statistical analysis, depending on the amount of biological variability. More animals or cells were used when variability was expected to be higher, such as Fig.3g,j,n; Fig.7e,h,l,n,o. No significant inter-individual variability was observed in our results. We thus reasoned that the sample sizes were sufficient to demonstrate the findings. The precise number of animals and cells is reported in the figure legends and methods.
Data exclusions	Data exclusions are reported also in the Material part of the manuscript.<br>For Single Cell RNA Sequencing analysis in the juvenile small intestine: Cells with <500 genes or >6,500 genes were removed. To mitigate potential doublets, we also excluded cells with >40,000 UMI counts. Of the remaining cells we further removed cells with the fraction of counts from mitochondrial genes >0.2 to account for injured cells. Prominent sex specific genes (Xist, Gm13305, Tsix, Eif253y, Ddx3y, Uty, Kdm5d) were removed from variable genes before principal component (PC) analysis. It is wildly appreciated that these genes can confound biological variances that relevant to intrinsic neuron heterogeneity. We reiteratively clustered, removing first non-ENS clusters and then also myenteric and glial cell contaminating clusters (Extended Data Fig. 1).<br><br>For Single Cell RNA Sequencing of postnatal day 7 small intestine: Cells with <500 genes or >6,500 genes were removed. To mitigate potential doublets, we also excluded cells with >40,000 UMI counts. Cells with > 0.1 fraction of mitochondrial genes (damaged cells) were also removed. Prominent sex specific genes (Xist, Gm13305, Tsix, Eif253y, Ddx3y, Uty, Kdm5d) and a set of immediate early genes, which could be upregulated in response to cell dissociation procedure (Fos, Jun, Junb, Egr1) were removed from variable genes before principal component (PC) analysis. Non-enteric clusters were removed because they can confound relevant biological variances.<br><br>Samples with poor immunohistochemical or RNAScope staining quality were excluded from further analysis.
Replication	All the measurements that didn't require statistical testing were taken from at least 2 animals in independent experiments (otherwise at least 3). All replication attempts were successful (except cases listed as reason for exclusion).

Randomization	There was no randomization when animal/samples were assigned to the various experimental groups, the groups were determined by animal's genotype. Experimental conditions were the same for each group. Animals from different litters were used for repeat experiments to avoid confounding.
Blinding	No biological groups of animals were compared in the majority of experiments and therefore did not require blinding. Some experiments were however repeated by several researchers, with similar findings. Data collection was not blinded due to the inherent characteristics of cellular attributes within the experiments.

Reporting for specific materials, systems and methods

We require information from authors about some types of materials, experimental systems and methods used in many studies. Here, indicate whether each material, system or method listed is relevant to your study. If you are not sure if a list item applies to your research, read the appropriate section before selecting a response.

Materials & experimental systems

n/a	Involved in the study
☐	☒ Antibodies
☒	☐ Eukaryotic cell lines
☒	☐ Palaeontology and archaeology
☐	☒ Animals and other organisms
☒	☐ Clinical data
☒	☐ Dual use research of concern
☒	☐ Plants

Methods

n/a	Involved in the study
☒	☐ ChIP-seq
☐	☒ Flow cytometry
☒	☐ MRI-based neuroimaging

Antibodies

Antibodies used	5-HT Goat 1:500 Abcam ab66047<br>CALB Mouse 1:500 Swant CB300<br>CALR Mouse 1:500 Santa Cruz sc-365956<br>CD31 goat 1:500 Novus Bio AF3628<br>ENK Mouse 1:300 Abcam ab150346<br>GFP Chicken 1:1000 Abcam ab13970<br>HuC/D Mouse 1:300 Molecular Probes A21271<br>HuC/D Rabbit 1:300 Abcam ab184267<br>LYVE-1 rat 1:400 Novus Bio MAB2125<br>DCLK1 Rabbit 1:400 Abcam ab31704<br>NDUFA4L2 Rabbit 1:200 Proteintech 16480-1-AP<br>NF-M Mouse 1:500 Abcam ab7794<br>NOS1 Goat 1:1,000 Abcam ab1376<br>NOS1 Rabbit 1:200 Santa Cruz sc-648<br>NTNG1 Rabbit 1:200 Abcam ab221456<br>PAIP2B Rabbit 1:200 Invitrogen PA5-61323<br>PGP9.5 Rabbit 1:500-1000 Invitrogen PA5-29012<br>PGP9.5 Mouse 1:300 Novus Bio NB600-1160<br>PSD95 nanobody Camel 1:300 N3702-AB635P-L<br>RFP/Tomato Rat 1:1000 Chromotek (Proteintech), 5F8<br>SOM Rat 1:100 Merck MAB354<br>SOX10 Goat 1:2000 R&D Systems AF2864<br>TH Sheep 1:300 Novus Bio NB300-110<br>VIP Rabbit 1:1000 Immunostar 20077<br>Secondary antibodies used in this study are listed in the Supplementary Table 5
Validation	5-HT Abcam ab66047 was validated by the manufacturer for immunofluorescence application; reactivity species independent<br>CALB Swant CB300 was validated by the manufacturer for immunofluorescence application and reacts with mouse<br>CALR Santa Cruz sc-365956 was validated by the manufacturer for immunohistochemistry application and reacts with mouse<br>CD31 Novus Bio AF3628 was validated by the manufacturer for immunohistochemistry application and reacts with mouse<br>ENK Abcam ab150346 was validated by the manufacturer for immunohistochemistry application and reacts with mouse<br>GFP Abcam ab13970 was validated by the manufacturer for immunofluorescence application and eactivity against mouse observed in this manuscript<br>HuC/D Molecular Probes A21271 was validated by the manufacturer for immunofluorescence application; reactivity against mouse was previously published (Senzacqua et al, Frontiers in Neuroscience, 2016; Tirou et al, Plos One, 2020)<br>HuC/D Abcam ab184267 was validated by the manufacturer for immunohistochemistry application and reacts with mouse<br>LYVE-1 Novus Bio MAB2125 was validated by the manufacturer for immunohistochemistry application and reacts with mouse<br>DCLK1 Abcam ab31704 was validated by the manufacturer for immunofluorescence application and reacts with mouse<br>NDUFA4L2 Proteintech 16480-1-AP was validated by the manufacturer for immunohistochemistry application and reacts with mouse<br>NF-M Abcam ab7794 was validated by the manufacturer for immunofluorescence application and reacts with mouse<br>NOS1 Abcam ab1376 was validated by the manufacturer for immunohistochemistry application and reacts with mouse

NOS1 Santa Cruz sc-648 was validated by the manufacturer for immunohistochemistry application and reacts with mouse
NTNG1 Abcam ab221456 was validated by the manufacturer for immunofluorescence application, predicted to work in mouse by the manufacturer; reactivity against mouse observed in this manuscript
PAIP2B Invitrogen PA5-61323 was validatd by the manufacturer for immunohistochemistry application; Reactivity against mouse observed in this manuscript
PGP9.5 Invitrogen PA5-29012 was observed to give reactivity in immunofluorescence application in this manuscript; validated to react against mouse by the manufacturer
PGP9.5 Novus Bio NB600-1160 was observed to give reactivity in immunofluorescence application in this manuscript; validated to react against mouse by the manufacturer
PSD95 nanobody N3702-AB635P-L was validated by the manufacturer for immunofluorescence application and reacts with mouse
RFP/Tomato Chromotek (Proteintech) 5F8 was validated by the manufacturer for immunofluorescence application and reacts with mouse
SOM Merck MAB354 was validated by the manufacturer for immunohistochemistry application; shown to react in mouse tissue in Hioki et al, Journal of Neuroscience, 2013
SOX10 R&D Systems AF2864 was validated for immunofluorescence in mouse species by Fazal et al, Journal of Neuroscience, 2017
TH Novus Bio NB300-110 was validated by the manufacturer for immunohistochemistry application and validated to react against mouse by the manufacturer
VIP Immunostar 20077 was validated by the manufacturer for immunohistochemistry application and validated to react against mouse

Animals and other research organisms

Policy information about studies involving animals; ARRIVE guidelines recommended for reporting animal research, and Sex and Gender in Research

Laboratory animals	Mice were used in our study. Animals were group-housed, with food and water ad libitum, under 12-h light-dark cycle conditions, 22° C ambient temperature and 50% humidity.<br>For juvenile Single cell sequencing, Baf53b-Cre(JAX,#027826) was crossed with Ai14(R26RtdTOM;JAX #007908) and eight Baf53b-Cre;R26RTomato animals were used at postnatal day (P)24 age, both male and female.<br>For single cell RNA-sequencing of postnatal day 7 animals, four Wnt1-Cre;R26Tomato animals were used, both male and female.<br>For immunofluorescence analysis these are the general ages used (also specified in the figure legend, or results): Wild type (C57BL6) mice (P2-P90 months old); Baf53-R26Tomato animals P24-P26 age; Nmu-Cre(MMRRC,036643-UCD), Sst-IRES-Cre(JAX,#013044), Vip-IRES-Cre(JAX,#010908) animals mainly 2-3 months old unless otherwise specified; Nos1-Cre(JAX,#017526) was crossed with R26-EGFP(JAX#004077) and the Nos1-Cre;R26-EGFP of various stages(P2-3, P21, 8-12w) as specified in the manuscript. Both females and males were used.<br>For single neuron morphology analysis, Nmu-Cre mice at age of 14w were used.<br>For calcium indicator imaging analysis, Nmu-Cre was crossed with Ai148D(LSL-GCaMP6f,JAX#030328), and the Nmu-Cre;LSL-GCaMP6f mice at 18-22w were used, both males and females.
Wild animals	This study did not involve wild animals
Reporting on sex	Both females and males were included in the scRNA-sequencing datasets. Cells from each sex could be distinguished by expression of sex-specific genes such as Xist and Ddx3y.
Field-collected samples	This study did not involve field-collected samples
Ethics oversight	Animal experiments were approved by the local ethics committee in northern Stockholm (Stockholm Norra djurförsöksetiska nämnd, Jordbruksverket) N5264/18, N6626-2019 and N5237-2023. The experiments carried out at Mayo Clinic, Minnesota (US) were approved by the Mayo Clinic Institutional Animal Care and Use Committee (Extended Data Figure 4).

Note that full information on the approval of the study protocol must also be provided in the manuscript.

Plants

Seed stocks	*Report on the source of all seed stocks or other plant material used. If applicable, state the seed stock centre and catalogue number. If plant specimens were collected from the field, describe the collection location, date and sampling procedures.*
Novel plant genotypes	*Describe the methods by which all novel plant genotypes were produced. This includes those generated by transgenic approaches, gene editing, chemical/radiation-based mutagenesis and hybridization. For transgenic lines, describe the transformation method, the number of independent lines analyzed and the generation upon which experiments were performed. For gene-edited lines, describe the editor used, the endogenous sequence targeted for editing, the targeting guide RNA sequence (if applicable) and how the editor was applied.*
Authentication	*Describe any authentication procedures for each seed stock used or novel genotype generated. Describe any experiments used to assess the effect of a mutation and, where applicable, how potential secondary effects (e.g. second site T-DNA insertions, mosiacism, off-target gene editing) were examined.*

Flow Cytometry

Plots

Confirm that:

☒ The axis labels state the marker and fluorochrome used (e.g. CD4-FITC).

☒ The axis scales are clearly visible. Include numbers along axes only for bottom left plot of group (a 'group' is an analysis of identical markers).

☒ All plots are contour plots with outliers or pseudocolor plots.

☒ A numerical value for number of cells or percentage (with statistics) is provided.

Methodology

Sample preparation	Preparation of single cell solutions from juvenile and early postnatal mice are explained in the material and methods section. In short, dissected intestines were dissociated using EDTA, Liberase TH and dispase, manually triturated using fire-polished pasteur pipettes and then filtered through 70 (P24) or 40 (P7) mikrometer cell strainers. Solutions were centrifuged at 150g. Pellets were resuspended in PBS with 1% BSA with or without Draq7.
Instrument	Becton Dickinson Influx 2011
Software	BD FACS Sortware v. 1.0.0.650
Cell population abundance	The purity of post-sorted fraction was determined by fluorescent microscopy by sorting the cells on a microscope slide and visualizing tdTOM fluorescence. The relative abundance of ENS cells was further confirmed by scRNAseq.
Gating strategy	The first gate was set up based on SSC/FSC plot to eliminate debris (low FSC). Then the sample was gated on singlets (SSC vs Trigger pulse width). This was set up based on deviation of the dots from the well defined population in the Trigger Pulse Width direction. Finally, tdTOM+ cells were gated based on clearly positive 580/30 (tdTomato) fluorescent population. Supplementary Figure 8 shows representative plots for the gating strategy.

☒ Tick this box to confirm that a figure exemplifying the gating strategy is provided in the Supplementary Information.
