# Supplementary material for: The transcriptomes, connections and development of submucosal neuron classes in the mouse small intestine
Source: Nat Neurosci. 2025 May 29;28(6):1146–59. doi: 10.1038/s41593-025-01962-x (PMC12148937; doi:10.1038/s41593-025-01962-x)
Supplement: Supplementary file 8 — DE genes in level 1 clusters of P24 submucosa. [file 41593_2025_1962_MOESM8_ESM.pdf]

Supplementary Table 1. Enriched genes in Level 1 clustering, submucosa P24

p-val: unadjusted p value; avg_logFC: log fold-change of the average expression between two groups. Positive values indicate high gene expression; pct.1: the percentage of cells where the gene is detected in the group; pct.2: The percentage of cells where the gene is detected in the rest of the dataset; p_val_adj: adjusted p-value based on Bonferroni correction using all genes in the dataset; Statistical analysis was two-sided but only enriched genes are shown.

gene	p_val	avg_log2FC	pct.1	pct.2	p_val_adj	cluster
Meg3	0	1,419856776	1	0,993	0	0
Snhg11	5,8668E-279	1,085062359	1	0,982	1,0502E-274	0
mt-Nd2	2,8065E-275	0,903216467	1	1	5,0237E-271	0
6330403A02Rik	5,5541E-269	1,185759298	1	0,917	9,9418E-265	0
Ppp3ca	2,9684E-268	1,149077866	1	0,954	5,3134E-264	0
Gfra2	6,3798E-265	1,262961651	0,927	0,422	1,142E-260	0
Elavl4	1,1641E-263	1,023726323	1	0,984	2,0837E-259	0
Zbtb20	4,3962E-258	1,241474831	0,998	0,919	7,8691E-254	0
mt-Atp8	3,9405E-255	0,98360915	1	1	7,0534E-251	0
mt-Nd4l	7,5483E-249	0,915797723	1	1	1,3511E-244	0
Hoxa5	1,2286E-247	1,296332502	1	0,925	2,1992E-243	0
Scube1	1,1027E-239	1,101095232	0,999	0,881	1,9739E-235	0
Ntrk3	2,8111E-238	1,246532094	0,975	0,675	5,0318E-234	0
mt-Nd1	1,6826E-234	0,800615406	1	1	3,0119E-230	0
Pcdh7	1,0281E-218	1,146885064	0,824	0,347	1,8404E-214	0
Mcam	3,7764E-216	1,16075759	0,976	0,64	6,7598E-212	0
Enah	5,5201E-207	1,02241079	0,99	0,858	9,8809E-203	0
Dst	1,0545E-204	0,817672691	1	0,99	1,8875E-200	0
mt-Nd3	1,5599E-203	0,81169821	0,999	0,999	2,7923E-199	0
Pak3	8,6776E-203	1,131703492	0,935	0,67	1,5533E-198	0
Adrbk2	3,3551E-201	0,931933641	0,998	0,931	6,0056E-197	0
Gsk3b	1,1909E-200	0,866240565	0,999	0,976	2,1317E-196	0
Bche	8,1257E-197	1,067757898	0,985	0,759	1,4545E-192	0
Rbms3	1,6348E-194	0,904685465	1	0,978	2,9264E-190	0
Tshz2	9,9781E-192	1,038132414	0,995	0,831	1,7861E-187	0
mt-Atp6	2,2488E-191	0,521036773	1	1	4,0253E-187	0
Plod2	1,7376E-190	1,013712619	0,968	0,81	3,1103E-186	0
Nrxn3	6,6168E-185	0,967494203	0,845	0,403	1,1844E-180	0
Slc5a7	1,2671E-182	1,113604315	0,893	0,592	2,2681E-178	0
Slc36a1	7,9248E-181	0,903132879	1	0,942	1,4185E-176	0
Itga6	9,4389E-179	1,024676183	0,827	0,459	1,6896E-174	0
Snap25	5,8E-178	0,77920051	1	0,993	1,0382E-173	0
Cd47	1,6203E-177	0,910020884	0,996	0,921	2,9003E-173	0
Tcf4	6,406E-177	0,758617615	0,998	0,98	1,1467E-172	0
Celf4	2,0985E-174	0,787602229	0,999	0,959	3,7564E-170	0
Rbms1	1,242E-173	0,954915182	0,958	0,78	2,2232E-169	0
mt-Co3	1,5818E-173	0,464689772	1	1	2,8314E-169	0

Spock2	1,2941E-172	0,787919722	1	0,963	2,3165E-168	0
Prnp	8,0466E-172	0,859648721	1	0,97	1,4403E-167	0
Ptprs	4,0629E-171	0,931639201	0,953	0,737	7,2727E-167	0
Bcl2	2,5551E-170	1,042365003	0,798	0,433	4,5736E-166	0
Syt17	3,5613E-167	0,953907492	0,949	0,731	6,3746E-163	0
mt-Cytb	5,2904E-166	0,443541506	1	1	9,4697E-162	0
Gria2	3,1355E-164	0,895861081	0,993	0,905	5,6126E-160	0
Kcnb1	8,865E-164	1,001773591	0,817	0,509	1,5868E-159	0
Nfib	8,4317E-163	0,870004595	0,983	0,862	1,5093E-158	0
mt-Nd4	1,0785E-161	0,50398022	1	1	1,9304E-157	0
Bnc2	1,4594E-161	0,773706631	0,593	0,204	2,6123E-157	0
Slc10a4	1,1944E-160	0,861821659	0,983	0,777	2,138E-156	0
Nfix	1,6762E-160	0,91218848	0,955	0,738	3,0004E-156	0
Adamts9	3,0345E-160	0,881219405	0,647	0,249	5,4318E-156	0
Smarca2	4,7131E-160	0,797040683	1	0,932	8,4364E-156	0
Syn2	2,1746E-159	0,619150712	1	0,991	3,8925E-155	0
Ssbp3	1,3301E-158	0,78977451	0,996	0,935	2,3809E-154	0
Cnr1	6,8331E-158	0,675224269	1	0,979	1,2231E-153	0
Scg2	9,4237E-157	0,665463446	1	0,994	1,6868E-152	0
Slc7a14	1,5451E-156	0,897194664	0,975	0,807	2,7657E-152	0
Parm1	6,6279E-156	1,029628784	0,994	0,865	1,1864E-151	0
Fam19a5	6,4822E-154	0,934635879	0,771	0,366	1,1603E-149	0
Mapt	1,3748E-152	0,746722576	0,998	0,951	2,4609E-148	0
Casz1	4,8063E-152	0,866936423	0,768	0,351	8,6032E-148	0
Nsg2	5,4478E-152	0,716061154	1	0,981	9,7516E-148	0
Mycbp2	2,9857E-151	0,725325691	1	0,95	5,3444E-147	0
Gse1	6,1739E-151	0,843456881	0,973	0,805	1,1051E-146	0
Prkce	5,8397E-147	0,843782741	0,953	0,792	1,0453E-142	0
Hoxb5	1,3476E-146	0,792401028	0,999	0,941	2,4122E-142	0
Pura	4,7152E-146	0,749343287	0,998	0,962	8,4402E-142	0
Nlgn1	3,5738E-145	0,901737206	0,712	0,367	6,397E-141	0
Elavl3	1,0429E-144	0,827780921	0,987	0,895	1,8667E-140	0
Syt11	2,7361E-144	0,709837608	1	0,978	4,8976E-140	0
Timp3	8,75E-144	0,889685808	0,862	0,456	1,5663E-139	0
Ryr2	1,8856E-143	0,819658119	0,615	0,25	3,3752E-139	0
Prkacb	6,5192E-143	0,792875997	0,985	0,903	1,1669E-138	0
Atp1b1	1,0631E-142	0,773612179	1	0,865	1,9029E-138	0
Ywhag	6,4383E-140	0,661200656	0,999	0,98	1,1525E-135	0
Rtn3	6,548E-139	0,647639877	1	0,992	1,1721E-134	0
Rbfox1	8,6417E-139	0,864904349	0,865	0,633	1,5469E-134	0
Phox2b	3,7788E-137	0,685068304	0,999	0,98	6,764E-133	0
mt-Nd5	1,1724E-136	0,614918379	1	0,999	2,0985E-132	0
Peg3	3,153E-135	0,682509832	0,999	0,973	5,6439E-131	0
Klf7	3,1762E-135	0,751508109	0,989	0,929	5,6855E-131	0
Tcaf1	4,5627E-134	0,743519907	0,99	0,902	8,1672E-130	0
Kcnma1	5,5933E-134	0,922318841	0,749	0,478	1,0012E-129	0

1810041L15Rik	2,4984E-133	0,826978448	0,914	0,708	4,4721E-129	0
Sdc3	1,482E-132	0,783278054	0,959	0,79	2,6528E-128	0
Thra	2,8675E-131	0,635414605	1	0,986	5,1328E-127	0
Kcnq1ot1	1,1453E-130	0,933982208	0,964	0,833	2,0502E-126	0
Ppm1h	2,2056E-129	0,786921204	0,886	0,669	3,948E-125	0
Tcf7l2	5,1905E-129	0,725437398	0,988	0,832	9,291E-125	0
Mapre2	6,2099E-129	0,810467718	0,942	0,805	1,1116E-124	0
Calcb	2,3508E-128	0,685401916	0,979	0,506	4,208E-124	0
Caln1	1,3919E-125	0,678552858	0,486	0,166	2,4916E-121	0
Rab6b	1,693E-125	0,83707312	0,993	0,891	3,0305E-121	0
Ddx5	2,4946E-125	0,553609992	1	0,999	4,4654E-121	0
mt-Co2	1,3124E-124	0,37591909	1	1	2,3492E-120	0
Plekha6	2,0318E-124	0,721468558	0,935	0,761	3,6369E-120	0
Kif1b	2,981E-124	0,614739633	1	0,985	5,336E-120	0
Atp2b4	3,8288E-124	0,777813593	0,913	0,706	6,8535E-120	0
Ppp2r2c	2,7015E-123	0,783473365	0,944	0,774	4,8356E-119	0
Nktr	3,1426E-123	0,808302788	0,922	0,749	5,6253E-119	0
Calcb	0	1,925306872	1	0,505	0	1
Ifitm2	0	1,302355077	1	0,939	0	1
Csrp2	0	1,240013244	0,928	0,364	0	1
Ifi27	3,5971E-302	1,248796888	0,998	0,767	6,4387E-298	1
Lgals3	2,995E-286	0,788920892	0,44	0,06	5,3611E-282	1
Pdlim2	8,8655E-286	1,226482296	0,775	0,248	1,5869E-281	1
Rpl24	7,3314E-283	1,05546807	1	0,943	1,3123E-278	1
Sst	2,5396E-280	1,799644832	1	0,817	4,546E-276	1
Stmn3	8,3498E-277	1,132353584	1	0,908	1,4946E-272	1
Cox6c	1,0068E-275	1,146104452	1	0,867	1,8022E-271	1
Prph	7,352E-275	0,948037348	1	0,96	1,316E-270	1
Atp6v1e1	6,3161E-265	0,994615235	1	0,951	1,1306E-260	1
Hint1	5,586E-264	1,079658371	1	0,875	9,9989E-260	1
Dmkn	7,7827E-260	1,0691954	0,885	0,299	1,3931E-255	1
Ubb	2,7623E-259	1,068763466	1	0,986	4,9444E-255	1
Rpl37	3,3298E-255	0,980157567	1	0,985	5,9604E-251	1
Ly6h	7,4518E-255	1,104844802	0,942	0,453	1,3339E-250	1
Gap43	9,248E-250	0,843714191	1	0,985	1,6554E-245	1
Rpl6	2,5375E-247	0,997840359	1	0,97	4,5421E-243	1
S100a1	1,1455E-245	1,095020271	0,998	0,813	2,0504E-241	1
Rpl9	2,9449E-244	1,068664269	1	0,815	5,2713E-240	1
Rps8	4,0382E-241	0,901241311	1	0,976	7,2284E-237	1
Rps27	4,6822E-239	0,99646585	1	0,863	8,3812E-235	1
Higd1a	1,3423E-237	0,786824317	0,72	0,213	2,4026E-233	1
Atp5k	6,1394E-237	0,96587877	1	0,955	1,0989E-232	1
Slc18a3	2,5681E-236	1,044389806	0,973	0,492	4,5969E-232	1
Fxyd7	4,5395E-236	1,136694283	1	0,74	8,1257E-232	1
Tpt1	4,9561E-236	0,861117391	1	0,955	8,8714E-232	1
Atpif1	1,0567E-235	0,989323294	1	0,946	1,8915E-231	1

Rpl35a	6,7156E-235	0,932397396	1	0,92	1,2021E-230	1
Rpl39	1,6014E-233	1,013949567	1	0,933	2,8664E-229	1
Rpl37a	2,2657E-233	0,885518401	1	0,986	4,0556E-229	1
Ddah1	3,1358E-233	0,999151256	0,9	0,37	5,613E-229	1
Chchd2	5,1921E-232	0,957498206	1	0,914	9,2939E-228	1
Aldoa	3,8022E-231	0,770991179	1	0,994	6,806E-227	1
Ass1	7,8421E-231	1,046808605	0,932	0,49	1,4037E-226	1
Rps21	7,0057E-230	1,053706019	1	0,948	1,254E-225	1
Atp5j2	3,8918E-229	1,0132369	1	0,789	6,9662E-225	1
H2afz	5,6627E-228	1,049394592	0,979	0,719	1,0136E-223	1
Atp5e	8,4023E-228	1,019372124	1	0,839	1,504E-223	1
Vwc2	7,1585E-227	0,811818914	0,693	0,208	1,2814E-222	1
Rps3a1	2,7025E-226	0,967736002	0,999	0,831	4,8375E-222	1
Rps15a	1,705E-225	0,855131011	1	0,883	3,052E-221	1
Map1lc3a	2,7116E-225	0,812858145	1	0,968	4,8537E-221	1
Rps27a	2,1008E-224	0,917082443	1	0,926	3,7605E-220	1
Rps29	7,611E-224	0,855978777	1	0,996	1,3624E-219	1
Rpl3	3,003E-223	0,913053154	1	0,916	5,3753E-219	1
Rpl22l1	1,5515E-220	0,93835145	0,998	0,771	2,7772E-216	1
Rpl13	5,1042E-220	0,852928412	1	0,953	9,1365E-216	1
Rps3	8,8359E-217	0,903001954	0,998	0,863	1,5816E-212	1
Fth1	1,1204E-216	0,799851393	1	0,993	2,0055E-212	1
Atp5h	8,2559E-216	0,892858399	0,999	0,865	1,4778E-211	1
Ndufa4	5,9709E-214	0,936064352	0,999	0,832	1,0688E-209	1
Kif22	4,7819E-213	1,097287603	0,975	0,709	8,5596E-209	1
Ndufb9	1,5484E-211	0,953568254	0,981	0,634	2,7717E-207	1
Adgre1	2,6329E-211	0,963134084	0,87	0,41	4,713E-207	1
Pfdn5	2,6623E-210	0,917945906	0,991	0,746	4,7654E-206	1
Rpl17	1,7568E-207	0,816020811	1	0,912	3,1446E-203	1
Rps5	7,6823E-207	0,842128438	1	0,882	1,3751E-202	1
Rpl19	8,0074E-207	0,889452547	1	0,929	1,4333E-202	1
Rpl30	1,1552E-206	0,873843407	1	0,814	2,0679E-202	1
Slc25a4	1,7179E-206	0,796111257	1	0,967	3,075E-202	1
Rpl32	2,8484E-205	0,764367291	1	0,965	5,0986E-201	1
Sec62	1,4429E-203	0,803059691	0,999	0,933	2,5828E-199	1
Uqcr10	1,5E-203	0,902339568	0,995	0,736	2,685E-199	1
Rprm	4,501E-202	0,742549575	0,726	0,234	8,0567E-198	1
Rpl36a	1,3341E-201	0,875355593	0,995	0,762	2,3881E-197	1
Ndufb10	2,3031E-201	0,882790628	0,957	0,504	4,1225E-197	1
S100a6	4,9554E-201	0,86804987	1	0,993	8,8702E-197	1
Tspo	8,2264E-201	0,916618246	0,968	0,633	1,4725E-196	1
Rps10	1,5107E-200	0,809037499	1	0,917	2,7041E-196	1
Ndufa5	1,7411E-200	0,874625412	0,999	0,794	3,1166E-196	1
Rpl23	1,8326E-199	0,663943306	1	0,991	3,2803E-195	1
Rpl34	2,5678E-199	0,800479768	1	0,914	4,5964E-195	1
Ndufa3	2,7983E-199	0,888237811	0,995	0,765	5,0089E-195	1

Nme1	4,1701E-199	0,905675652	0,974	0,649	7,4646E-195	1
Rab3b	6,5336E-197	0,828848734	0,92	0,418	1,1695E-192	1
Hmgb1	3,7189E-196	0,766577133	1	0,972	6,6568E-192	1
Rps4x	1,0323E-195	0,849963493	1	0,881	1,8477E-191	1
6330403K07Rik	1,1331E-195	0,876862489	0,996	0,908	2,0282E-191	1
Gapdh	1,8394E-194	0,924365774	1	0,925	3,2926E-190	1
Rps24	1,8711E-194	0,783154408	1	0,965	3,3493E-190	1
C1ql3	3,1155E-193	0,482650795	0,33	0,049	5,5767E-189	1
Cox5a	7,5013E-193	0,879000869	0,983	0,625	1,3427E-188	1
Cox4i1	3,123E-192	0,871748129	1	0,883	5,5902E-188	1
Rps7	7,0332E-192	0,88352564	0,995	0,781	1,2589E-187	1
Rps18	1,8328E-191	0,750354948	1	0,931	3,2807E-187	1
Avpr1a	1,2445E-190	0,669701334	0,693	0,215	2,2276E-186	1
Rps20	6,3829E-190	0,766120758	1	0,897	1,1425E-185	1
Ndufc1	8,46E-188	0,831943783	0,999	0,811	1,5143E-183	1
Rpl21	4,7409E-187	0,719962496	1	0,965	8,4861E-183	1
Rps9	5,1587E-187	0,778238853	1	0,916	9,2341E-183	1
Tmsb10	1,9857E-186	0,825178363	0,999	0,944	3,5545E-182	1
Gng3	3,7765E-186	0,848272139	0,986	0,78	6,7599E-182	1
Rtn1	4,9648E-186	0,660315738	1	0,989	8,887E-182	1
Gm10076	2,8919E-185	0,926088437	1	0,844	5,1764E-181	1
Uqcr11	1,2357E-184	0,852556208	0,999	0,82	2,2118E-180	1
Eef1b2	1,3898E-184	0,7862945	0,995	0,809	2,4877E-180	1
Rps11	5,3501E-184	0,741084619	1	0,932	9,5766E-180	1
Psme1	1,0424E-183	0,852305349	0,996	0,837	1,866E-179	1
Ftl1	4,157E-183	0,780898788	1	0,925	7,4411E-179	1
Etv1	0	1,73256374	0,999	0,579	0	2
Camk2a	0	1,536768654	0,985	0,674	0	2
Tcf4	3,4554E-263	1,078520959	1	0,98	6,1852E-259	2
Adgrl1	3,656E-253	1,133167209	0,997	0,848	6,5442E-249	2
F2r	1,0329E-248	1,574312575	0,956	0,535	1,8488E-244	2
Syt7	1,3631E-242	1,222957343	0,988	0,796	2,4399E-238	2
Fam155a	7,76E-232	1,085743293	0,84	0,343	1,389E-227	2
Man2a1	1,8151E-227	1,230970062	0,984	0,816	3,249E-223	2
Cntnap5a	4,5745E-227	1,162450568	0,991	0,636	8,1884E-223	2
Plekha5	8,1799E-224	1,199127041	0,933	0,612	1,4642E-219	2
Moxd1	4,0061E-219	1,206440656	0,939	0,452	7,171E-215	2
Kcnd2	1,2953E-214	1,184542412	0,814	0,35	2,3185E-210	2
Cd24a	5,8283E-214	1,303743707	0,995	0,608	1,0433E-209	2
Asic2	1,6175E-212	1,11947933	0,972	0,729	2,8954E-208	2
Ncam1	9,9406E-210	0,812996921	1	0,986	1,7794E-205	2
Ugcg	1,1195E-207	1,202987184	0,964	0,746	2,0039E-203	2
Pxylp1	2,2101E-207	1,122853922	0,952	0,614	3,956E-203	2
Kcnt2	2,6228E-207	0,974080806	0,762	0,283	4,6948E-203	2
Tbx3	1,4395E-200	0,932344157	1	0,927	2,5767E-196	2
Vip	7,9394E-195	1,426031936	1	0,92	1,4212E-190	2

Lrrc3	1,182E-194	1,267317651	0,797	0,389	2,1157E-190	2
Gria3	3,4267E-194	0,917663862	0,704	0,239	6,1339E-190	2
Gpr149	1,6537E-193	0,965073004	0,789	0,314	2,9601E-189	2
9530059O14Rik	5,1826E-193	1,060833853	0,971	0,719	9,2769E-189	2
Ptger4	1,787E-191	1,022009168	0,845	0,413	3,1987E-187	2
Ptbp3	3,6933E-191	1,018715913	0,98	0,841	6,611E-187	2
Rimbp2	3,6248E-185	0,880041869	0,72	0,262	6,4883E-181	2
Etnk1	1,6791E-184	0,963123407	0,983	0,856	3,0056E-180	2
Arl8a	1,2778E-183	0,829669347	0,997	0,969	2,2873E-179	2
Auts2	4,5896E-182	0,975003613	0,963	0,659	8,2153E-178	2
Cmip	1,321E-180	0,935080536	0,981	0,839	2,3646E-176	2
Spock3	5,1903E-180	1,032661706	0,94	0,567	9,2906E-176	2
Efna5	7,8811E-180	1,117586822	0,903	0,601	1,4107E-175	2
Pam	3,0582E-179	0,847564827	1	0,981	5,4742E-175	2
Alcam	1,1805E-178	1,095007863	0,969	0,595	2,1131E-174	2
R3hdm1	1,8754E-178	0,951437923	0,985	0,834	3,357E-174	2
Stxbp5	9,6765E-178	1,016395217	0,931	0,655	1,7321E-173	2
Kcnj3	5,0848E-175	0,797214561	0,674	0,232	9,1019E-171	2
Nav1	2,0235E-173	0,890641599	0,992	0,898	3,622E-169	2
Ptprn	1,0869E-172	0,728207097	1	0,992	1,9456E-168	2
Prokr1	2,7858E-172	0,806917793	0,595	0,184	4,9866E-168	2
Clmn	4,8243E-170	0,989617013	0,836	0,447	8,6355E-166	2
Nrxn1	3,3024E-168	0,757490897	0,996	0,972	5,9113E-164	2
Pde3a	4,48E-167	1,009469143	0,809	0,41	8,0191E-163	2
Rbms3	9,5379E-166	0,865528025	1	0,979	1,7073E-161	2
Adcyap1r1	1,3887E-165	0,90893826	0,784	0,365	2,4858E-161	2
Kcnq3	2,0954E-161	0,965545226	0,883	0,544	3,7508E-157	2
Garnl3	2,2841E-161	0,883871371	0,941	0,695	4,0885E-157	2
mt-Co2	7,6541E-160	0,46716963	1	1	1,3701E-155	2
Elavl3	1,1016E-157	0,859956241	0,985	0,896	1,9719E-153	2
Hap1	5,1448E-157	0,90406747	0,979	0,837	9,2092E-153	2
Ret	2,7875E-155	0,787506726	0,996	0,947	4,9896E-151	2
Nsg2	8,7671E-155	0,75945752	1	0,982	1,5693E-150	2
Cadm1	6,6839E-154	0,707438236	1	0,979	1,1964E-149	2
Ank2	2,1062E-153	0,670378485	1	0,983	3,7702E-149	2
Gsk3b	1,0496E-150	0,715212865	0,995	0,977	1,8787E-146	2
Gnaq	1,6569E-149	0,952068484	0,944	0,779	2,9658E-145	2
mt-Atp6	1,1773E-148	0,455150271	1	1	2,1074E-144	2
Chl1	1,0121E-147	0,898484591	0,964	0,743	1,8117E-143	2
Frmd4a	2,5397E-145	0,770096899	0,989	0,905	4,5461E-141	2
mt-Co3	2,0095E-144	0,42882795	1	1	3,597E-140	2
Enpp1	1,4601E-143	0,73749062	0,696	0,283	2,6135E-139	2
Gfra1	5,9677E-143	0,870351846	0,888	0,436	1,0682E-138	2
Klf7	2,3623E-142	0,776668342	0,993	0,929	4,2285E-138	2
Gm13889	2,4992E-142	0,891803858	0,784	0,358	4,4736E-138	2
Arpp21	8,2438E-142	0,874526968	0,976	0,859	1,4756E-137	2

Eml5	9,5548E-142	0,905467704	0,85	0,529	1,7103E-137	2
Nbea	3,4778E-141	0,830776493	0,883	0,613	6,2252E-137	2
Ndst4	9,1691E-141	0,793967728	0,726	0,304	1,6413E-136	2
Celf3	1,1155E-140	0,850750223	0,971	0,824	1,9968E-136	2
Ngfr	9,0103E-139	0,891583401	0,925	0,522	1,6128E-134	2
Nrip1	6,7375E-138	0,872481353	0,972	0,847	1,206E-133	2
Atp8a1	7,2573E-138	0,85277159	0,928	0,752	1,2991E-133	2
Meg3	4,1108E-137	0,679831759	1	0,993	7,3584E-133	2
Mapt	1,2625E-136	0,738730806	0,995	0,952	2,2599E-132	2
Msi2	2,6532E-136	0,86903518	0,947	0,765	4,7493E-132	2
Pitpnc1	1,1168E-135	0,898900668	0,818	0,483	1,999E-131	2
Cbx6	1,8711E-134	0,739902391	0,997	0,93	3,3493E-130	2
mt-Atp8	2,2227E-133	0,71566227	1	1	3,9787E-129	2
Ttc39b	8,4822E-133	0,815324185	0,684	0,309	1,5183E-128	2
Wnk1	1,082E-132	0,776152914	0,96	0,794	1,9367E-128	2
Zfhx4	1,5913E-132	0,855159549	0,734	0,365	2,8484E-128	2
Camk2d	2,933E-131	0,806715452	0,94	0,754	5,2501E-127	2
Syne1	1,0542E-129	0,83517929	0,88	0,628	1,8869E-125	2
Elavl4	3,1997E-128	0,634484706	1	0,984	5,7275E-124	2
Gabrb3	1,4158E-127	0,894481579	0,818	0,543	2,5342E-123	2
Cacna2d1	1,6696E-127	0,761744241	0,977	0,901	2,9886E-123	2
D430019H16Rik	5,1197E-126	0,816919684	0,862	0,596	9,1643E-122	2
Ids	1,2722E-125	0,871928759	0,976	0,865	2,2771E-121	2
Prkar1a	1,7435E-125	0,640755516	0,999	0,987	3,1208E-121	2
Sstr1	8,2958E-125	0,88233102	0,848	0,603	1,485E-120	2
Dpysl2	6,3124E-124	0,730143361	0,997	0,971	1,1299E-119	2
Arhgap26	4,8792E-123	0,745005769	0,952	0,795	8,7337E-119	2
Actn1	1,4097E-121	0,774575183	0,88	0,614	2,5233E-117	2
Peg3	1,4714E-120	0,749464711	0,997	0,974	2,6338E-116	2
Tns1	2,5565E-120	0,746445716	0,943	0,771	4,576E-116	2
Bmpr2	6,4479E-118	0,789934236	0,903	0,719	1,1542E-113	2
Slc7a8	1,3348E-117	0,818523359	0,757	0,421	2,3893E-113	2
Camk4	3,2806E-117	0,851026193	0,853	0,551	5,8723E-113	2
Cds2	4,3319E-117	0,697291142	0,988	0,894	7,7542E-113	2
Rbfox2	6,1687E-117	0,719436065	0,941	0,833	1,1042E-112	2
Dbh	0	3,191662177	1	0,632	0	3
Th	0	2,093519536	0,883	0,254	0	3
Fibcd1	0	1,435383889	0,978	0,346	0	3
Gabre	0	1,297369956	0,7	0,136	0	3
Ntsr1	0	1,200740138	0,864	0,277	0	3
Scgn	7,7953E-305	1,658441452	1	0,847	1,3954E-300	3
Lamc3	2,0047E-281	1,009372319	0,816	0,237	3,5884E-277	3
Npr1	7,8649E-267	1,134226756	0,85	0,292	1,4078E-262	3
Gfra1	7,5095E-261	1,290374076	0,968	0,43	1,3442E-256	3
Nrsn2	2,3419E-258	1,080670203	0,692	0,186	4,192E-254	3
Moxd1	9,8551E-251	1,226598515	0,981	0,449	1,7641E-246	3

Tmem130	7,8516E-250	1,249253083	0,957	0,522	1,4054E-245	3
Cd24a	2,455E-248	1,447653315	0,992	0,609	4,3945E-244	3
Thy1	4,1265E-246	1,29906715	0,968	0,493	7,3865E-242	3
Thsd7a	5,3258E-242	0,88037726	0,745	0,214	9,5332E-238	3
Galnt6	2,0555E-218	0,920719449	0,66	0,185	3,6793E-214	3
Spock1	1,7931E-208	0,885899973	0,735	0,238	3,2097E-204	3
Ptprn	1,4314E-206	0,75053533	1	0,992	2,5623E-202	3
Hap1	2,9815E-205	0,974717949	0,996	0,836	5,3369E-201	3
Ece1	1,0006E-203	0,97819031	0,988	0,713	1,7911E-199	3
Pwwp2b	1,4899E-203	0,790864812	0,768	0,273	2,6669E-199	3
Chst8	4,4046E-203	0,658866636	0,617	0,163	7,8843E-199	3
Alcam	1,7108E-202	1,099754013	0,988	0,594	3,0623E-198	3
Npy	2,1931E-199	1,37084599	0,999	0,836	3,9257E-195	3
Tmod1	4,5059E-198	0,99979962	0,953	0,593	8,0656E-194	3
Asic2	6,18E-195	0,963457357	0,98	0,729	1,1062E-190	3
Adcyap1r1	8,2652E-194	0,827647428	0,861	0,359	1,4795E-189	3
F2r	4,5165E-193	1,046279671	0,968	0,534	8,0844E-189	3
Kcnj3	4,7957E-192	0,76492063	0,713	0,229	8,5843E-188	3
Kcnab2	1,5243E-186	0,866360262	0,819	0,339	2,7284E-182	3
Sertm1	1,7244E-181	0,732640068	0,65	0,2	3,0868E-177	3
Slc18a2	8,5747E-180	0,766961514	0,694	0,22	1,5349E-175	3
Kcnd2	3,0922E-178	0,776429157	0,864	0,346	5,535E-174	3
Prokr1	7,1826E-178	0,673943697	0,622	0,183	1,2857E-173	3
Fbn1	5,011E-176	0,694640437	0,758	0,272	8,9697E-172	3
Camk4	6,4838E-176	0,938064813	0,943	0,544	1,1606E-171	3
Etv1	2,6042E-171	1,04125987	1	0,58	4,6615E-167	3
Kcnj5	6,3034E-169	0,553050829	0,559	0,15	1,1283E-164	3
Spock3	1,8813E-168	0,950455398	0,955	0,566	3,3676E-164	3
Fxyd5	2,395E-166	0,824242487	0,897	0,392	4,2871E-162	3
Syp	3,1434E-164	0,738501991	0,999	0,958	5,6267E-160	3
Csrp1	1,7871E-160	0,793021735	0,999	0,95	3,1989E-156	3
Prkar1b	2,7102E-159	0,809919055	0,974	0,786	4,8513E-155	3
Astn2	5,9055E-159	0,746846936	0,74	0,3	1,0571E-154	3
Ptgfrn	1,3762E-158	0,534786427	0,532	0,141	2,4633E-154	3
Akap12	1,2434E-157	0,772932098	0,995	0,906	2,2257E-153	3
Pxylp1	3,4222E-155	0,87276061	0,946	0,615	6,1257E-151	3
Sod1	7,0344E-154	0,806537946	0,985	0,915	1,2591E-149	3
Gpr153	1,6407E-153	0,587633275	0,619	0,2	2,9368E-149	3
Entpd3	7,4687E-152	0,747422599	0,816	0,359	1,3369E-147	3
Stxbp5	1,5419E-151	0,777905109	0,972	0,652	2,76E-147	3
Vip	1,9873E-148	1,214990718	1	0,92	3,5573E-144	3
Neurl1a	3,0481E-147	0,812601109	0,955	0,69	5,4561E-143	3
Synpo2	2,4341E-146	0,553940753	0,543	0,16	4,3571E-142	3
Ptpro	3,1639E-144	0,422045233	0,332	0,062	5,6633E-140	3
Pdlim5	2,0506E-143	0,745355237	0,781	0,349	3,6705E-139	3
Rimbp2	1,1148E-139	0,588908797	0,704	0,264	1,9955E-135	3

F2rl2	2,7796E-138	0,520023659	0,551	0,168	4,9755E-134	3
Pnmal2	3,2195E-137	0,752045643	0,973	0,831	5,7629E-133	3
Dlk1	5,7929E-135	0,561965817	0,445	0,117	1,0369E-130	3
Pvrl1	7,255E-133	0,513156985	0,544	0,172	1,2986E-128	3
Tgfb1	9,7626E-133	0,596097398	0,771	0,304	1,7475E-128	3
Gpr149	2,9503E-132	0,658594691	0,764	0,317	5,2811E-128	3
Parva	8,6415E-132	0,730524121	0,958	0,824	1,5468E-127	3
Enpp2	9,6748E-131	0,520696386	0,541	0,172	1,7318E-126	3
Ngfr	4,1762E-130	0,771446331	0,958	0,519	7,4753E-126	3
Nsg1	1,308E-128	0,661252443	1	0,97	2,3413E-124	3
Fitm2	2,0717E-128	0,718875637	0,796	0,405	3,7084E-124	3
Cntnap2	1,0624E-127	0,595335888	0,71	0,292	1,9017E-123	3
Rasl10b	2,9765E-126	0,651026936	0,783	0,374	5,328E-122	3
Kcnt2	4,9891E-125	0,552763957	0,727	0,286	8,9305E-121	3
Myo16	8,0995E-124	0,393691632	0,387	0,095	1,4498E-119	3
Actb	2,3482E-123	0,45397039	1	1	4,2032E-119	3
Pld3	3,4658E-123	0,725861624	0,966	0,799	6,2038E-119	3
Insm1	3,8577E-123	0,563439765	0,63	0,23	6,9052E-119	3
Capn5	2,8396E-122	0,664934787	0,837	0,454	5,0828E-118	3
Cntnap5a	2,922E-122	0,694310021	0,988	0,636	5,2304E-118	3
Dclk2	3,0372E-122	0,625767776	0,816	0,415	5,4365E-118	3
Ttc39b	9,3868E-122	0,616482927	0,707	0,307	1,6802E-117	3
Cpm	1,3367E-121	0,501956604	0,486	0,148	2,3926E-117	3
Hpca	2,1749E-121	0,605195531	0,757	0,322	3,8931E-117	3
Tmem108	2,2248E-120	0,557920791	0,664	0,259	3,9825E-116	3
Gm26888	2,5065E-120	0,39651916	0,34	0,077	4,4867E-116	3
Apbb1	5,0466E-119	0,639870603	0,992	0,916	9,0335E-115	3
Chl1	1,341E-118	0,672808375	0,972	0,742	2,4004E-114	3
Saraf	2,1607E-118	0,677525871	0,985	0,908	3,8677E-114	3
Pcolce	2,4972E-117	0,514117387	0,591	0,218	4,4701E-113	3
Kif5c	3,4895E-115	0,588849869	0,995	0,932	6,2461E-111	3
Tspan13	1,0581E-114	0,701556493	0,96	0,732	1,894E-110	3
Gng4	2,5533E-113	0,722331571	0,918	0,65	4,5704E-109	3
Trpm2	1,2276E-112	0,461581721	0,56	0,198	2,1974E-108	3
Psap	2,101E-111	0,501964204	1	0,996	3,7607E-107	3
Gm13889	1,2375E-110	0,561254287	0,815	0,356	2,2152E-106	3
Tspan12	1,7568E-110	0,514789067	0,547	0,196	3,1446E-106	3
Ddb1	1,5638E-109	0,731087524	0,908	0,709	2,7992E-105	3
Pcsk1	1,8883E-109	0,728453047	0,919	0,623	3,38E-105	3
Fam155a	2,0019E-109	0,552347721	0,762	0,35	3,5834E-105	3
Plekhb2	1,21E-106	0,780327836	0,899	0,681	2,1659E-102	3
Enpp1	2,6619E-106	0,529047415	0,676	0,285	4,7649E-102	3
Eef1e1	3,9486E-106	0,666465394	0,876	0,505	7,0679E-102	3
Pde3a	6,799E-106	0,612026906	0,792	0,412	1,217E-101	3
Trp53i11	1,0087E-276	1,462548417	0,999	0,651	1,8056E-272	4
Mcam	1,6079E-271	1,368908499	0,989	0,643	2,8781E-267	4

Gm42418	1,5329E-248	1,289765146	1	1	2,7438E-244	4
Ly6e	4,3213E-240	1,358155872	0,954	0,488	7,7351E-236	4
Scube1	1,0227E-237	1,122871025	1	0,883	1,8307E-233	4
Prnp	1,2455E-234	0,951489812	1	0,97	2,2294E-230	4
Hoxa5	8,1857E-224	1,081485873	0,999	0,926	1,4652E-219	4
Bche	7,0895E-219	1,139519735	0,996	0,761	1,269E-214	4
Sphkap	4,9315E-218	0,945540806	0,769	0,242	8,8273E-214	4
Ebf3	4,6733E-214	0,714604744	0,564	0,126	8,3652E-210	4
Vat1l	1,0781E-210	1,02702068	0,993	0,804	1,9298E-206	4
Pcsk1n	2,8314E-209	0,766808556	1	0,995	5,0681E-205	4
Sez6l	2,1161E-206	1,086988067	0,906	0,459	3,7879E-202	4
Igfbp5	8,4835E-205	0,897559549	0,668	0,188	1,5186E-200	4
Timp3	3,6448E-204	1,103076689	0,95	0,454	6,5241E-200	4
Sst	3,2438E-196	1,464030837	1	0,819	5,8063E-192	4
Smarca2	8,0111E-193	0,919489333	0,997	0,933	1,434E-188	4
Slc10a4	2,9457E-192	0,988175073	0,985	0,779	5,2728E-188	4
Rspo2	9,0327E-185	0,789902249	0,691	0,212	1,6169E-180	4
Fam19a5	2,013E-183	0,943061616	0,867	0,363	3,6032E-179	4
Fxyd7	4,4188E-182	1,061918995	0,999	0,743	7,9096E-178	4
Vamp1	3,7178E-178	1,025332768	0,971	0,779	6,6548E-174	4
Snap25	1,0494E-177	0,698806615	1	0,993	1,8784E-173	4
Gsg1l	1,878E-173	0,687021787	0,484	0,113	3,3616E-169	4
Hoxb5	1,6675E-170	0,834569107	1	0,942	2,9848E-166	4
Camk2b	4,0168E-158	0,878098694	0,962	0,707	7,1902E-154	4
Pcdh7	4,1594E-158	0,800087932	0,863	0,35	7,4454E-154	4
Gfra2	7,3145E-157	0,847269947	0,937	0,427	1,3093E-152	4
Slc36a1	8,0722E-156	0,776041395	1	0,943	1,4449E-151	4
Calcb	3,8235E-151	0,937400258	0,985	0,512	6,8441E-147	4
Satb2	2,366E-150	0,790624983	0,785	0,353	4,2351E-146	4
Ache	4,7265E-149	0,744985207	0,999	0,948	8,4604E-145	4
Wbscr17	1,8477E-147	0,642701803	0,612	0,198	3,3075E-143	4
Spock2	2,9479E-145	0,675586242	0,997	0,964	5,2767E-141	4
Rph3a	5,1698E-145	0,911651152	0,935	0,607	9,254E-141	4
Mdga1	5,2712E-143	0,406496004	0,293	0,048	9,4354E-139	4
Nrxn2	6,8721E-143	0,70170079	0,999	0,945	1,2301E-138	4
Nfix	1,1907E-142	0,829073178	0,979	0,738	2,1313E-138	4
Ppp2r2c	5,2529E-140	0,764906248	0,968	0,774	9,4027E-136	4
Vipr2	6,646E-139	0,679183301	0,689	0,247	1,1896E-134	4
Atp1b1	4,1223E-137	0,749309697	0,996	0,867	7,379E-133	4
Caln1	1,6763E-136	0,556667418	0,55	0,165	3,0007E-132	4
Dst	1,2736E-135	0,610133541	1	0,991	2,2797E-131	4
Bnc2	2,3449E-134	0,561915464	0,624	0,206	4,1974E-130	4
Chd3	1,8417E-133	0,656023429	1	0,942	3,2967E-129	4
Avpr1a	1,887E-133	0,581668534	0,655	0,223	3,3777E-129	4
Nrp2	2,4696E-132	0,670021313	0,708	0,272	4,4205E-128	4
Atxn1	7,6673E-131	0,605957833	0,586	0,199	1,3724E-126	4

Itga6	1,4051E-130	0,777243329	0,872	0,46	2,5152E-126	4
Slc7a14	1,7186E-130	0,747443855	0,972	0,809	3,0764E-126	4
Serping1	4,7149E-129	0,777637577	0,801	0,372	8,4397E-125	4
Dmkn	2,3307E-128	0,767959727	0,778	0,314	4,1719E-124	4
Ina	2,0201E-126	0,774807576	0,942	0,721	3,616E-122	4
Tox	6,8294E-126	0,56643918	0,536	0,171	1,2225E-121	4
Piezo1	1,1194E-123	0,57778476	0,568	0,191	2,0037E-119	4
Apba2	1,9822E-123	0,760621907	0,95	0,681	3,5481E-119	4
Pak3	6,6247E-122	0,720983396	0,942	0,673	1,1858E-117	4
Tmem229b	1,28E-121	0,803094177	0,864	0,502	2,2912E-117	4
Fam19a2	1,1791E-120	0,472767166	0,383	0,095	2,1106E-116	4
Fat4	1,5641E-120	0,475149526	0,467	0,134	2,7998E-116	4
Sulf2	1,3944E-119	0,566853576	0,654	0,241	2,4961E-115	4
L1cam	3,3137E-118	0,590967748	1	0,966	5,9315E-114	4
Zcchc12	4,595E-118	0,74916247	0,976	0,858	8,2251E-114	4
Slc5a7	2,1236E-117	0,743582278	0,907	0,594	3,8013E-113	4
Nell1	2,5269E-117	0,665869091	0,665	0,262	4,5232E-113	4
6330403A02Rik	3,2249E-115	0,51534665	0,999	0,918	5,7726E-111	4
Casz1	4,3095E-114	0,548445043	0,827	0,352	7,7139E-110	4
P2rx2	6,8358E-114	0,637348388	1	0,963	1,2236E-109	4
App	1,897E-111	0,533267912	0,999	0,99	3,3957E-107	4
Dio2	4,3466E-110	0,484640938	0,415	0,116	7,7804E-106	4
Abca2	3,2482E-108	0,693059253	0,87	0,56	5,8143E-104	4
Myrip	3,6742E-108	0,687456134	0,763	0,39	6,5768E-104	4
Enah	9,3211E-108	0,613748341	0,985	0,86	1,6685E-103	4
Lynx1	2,6178E-107	0,551960594	0,624	0,242	4,6859E-103	4
Gdf10	5,9977E-107	0,523328873	0,459	0,143	1,0736E-102	4
Sel1l3	6,9687E-106	0,68031963	0,881	0,57	1,2474E-101	4
Smarcc2	7,56E-106	0,665984395	0,933	0,701	1,3532E-101	4
Adcy1	1,5668E-105	0,587753314	0,677	0,292	2,8045E-101	4
Oprl1	1,7646E-105	0,574892327	0,588	0,239	3,1586E-101	4
Ddah1	2,4633E-105	0,596124472	0,818	0,382	4,4092E-101	4
Sez6	1,4767E-104	0,589519954	0,703	0,324	2,6433E-100	4
Nsg2	4,3864E-104	0,512308961	1	0,982	7,8517E-100	4
Foxo3	4,8113E-104	0,62680518	0,727	0,361	8,6122E-100	4
Pth1r	1,3208E-103	0,577394383	0,561	0,212	2,3643E-99	4
Kcnc4	1,0701E-102	0,563297174	0,583	0,234	1,91554E-98	4
Tshz2	1,1055E-101	0,635557738	0,992	0,834	1,97888E-97	4
Flot2	3,0666E-101	0,59744185	0,986	0,904	5,48929E-97	4
Rprm	1,174E-99	0,596893616	0,612	0,249	2,10148E-95	4
Snrpn	1,8686E-99	0,567765555	0,997	0,968	3,34478E-95	4
Adamts9	2,7088E-99	0,559033527	0,628	0,256	4,84869E-95	4
Gtf2i	7,9214E-99	0,651979323	0,924	0,707	1,41793E-94	4
Slc18a3	9,0064E-99	0,74774146	0,872	0,506	1,61214E-94	4
Mapre3	1,11829E-98	0,669507043	0,91	0,709	2,00174E-94	4
Spata13	1,29068E-98	0,554755687	0,574	0,23	2,31032E-94	4

Ptprt	1,31636E-98	0,511230167	0,499	0,173	2,35628E-94	4
Dlgap3	2,08461E-98	0,647354325	0,753	0,403	3,73146E-94	4
Ctnna1	2,5519E-98	0,673073306	0,823	0,489	4,56789E-94	4
Gdi1	3,21642E-98	0,541197156	0,99	0,963	5,75738E-94	4
Kif21a	2,42466E-97	0,546867952	0,992	0,963	4,34013E-93	4
Ppp2r1a	2,48682E-97	0,545187568	0,997	0,944	4,45141E-93	4
Ptprj	4,08704E-97	0,644076434	0,721	0,369	7,31581E-93	4
Resp18	2,0633E-300	1,529006708	1	0,922	3,6933E-296	5
Th	1,8237E-290	1,58245144	0,863	0,26	3,2644E-286	5
Fxyd5	2,7343E-258	1,221528777	0,95	0,391	4,8945E-254	5
Vip	6,647E-253	1,729562743	1	0,921	1,1898E-248	5
Nsg1	1,8969E-247	1,025045926	1	0,97	3,3955E-243	5
Ccdc109b	4,189E-239	0,637314119	0,576	0,113	7,4984E-235	5
Igfbp7	3,7639E-229	0,836367415	0,96	0,368	6,7373E-225	5
Cd9	2,5486E-217	1,011012785	1	0,984	4,562E-213	5
Cst3	1,4603E-213	0,918056745	1	0,929	2,6139E-209	5
Moxd1	8,0697E-198	1,024358022	0,997	0,451	1,4445E-193	5
Npy	2,5621E-197	1,361847775	1	0,837	4,5862E-193	5
Cox7c	2,1334E-193	0,924187936	1	0,956	3,8187E-189	5
Pcolce	5,1706E-193	0,6446944	0,719	0,21	9,2554E-189	5
Tgfb1	3,0842E-188	0,821884445	0,84	0,302	5,5207E-184	5
Dbh	1,1215E-187	1,028159786	0,987	0,636	2,0074E-183	5
Ndst4	3,0006E-187	0,830939173	0,832	0,299	5,371E-183	5
Mpc1	4,0338E-186	0,956460317	0,981	0,816	7,2205E-182	5
Sec61g	1,0478E-182	0,897827481	1	0,868	1,8756E-178	5
Gapdh	5,0021E-180	0,978036035	1	0,926	8,9538E-176	5
A730017C20Rik	3,5177E-179	0,967387774	0,988	0,671	6,2968E-175	5
Gfra1	9,2485E-178	0,924474199	0,975	0,433	1,6555E-173	5
Sh3bgrl3	3,1634E-174	0,896719436	0,971	0,615	5,6625E-170	5
Scgn	1,6603E-173	1,28714156	1	0,848	2,9719E-169	5
Crabp1	4,2012E-167	1,320135499	0,869	0,445	7,5202E-163	5
Alcam	3,706E-165	0,958425433	0,994	0,596	6,6338E-161	5
Tuba1a	1,8982E-164	0,894859503	1	0,984	3,3977E-160	5
Id3	3,3774E-162	0,850280816	0,926	0,438	6,0456E-158	5
Bex2	4,0947E-158	0,635262048	1	0,991	7,3296E-154	5
Ppa1	2,9986E-157	0,851000543	0,941	0,563	5,3674E-153	5
Tceal6	8,6365E-157	0,65584008	0,684	0,229	1,5459E-152	5
Atp5j2	1,0427E-156	0,856201572	1	0,792	1,8665E-152	5
Thsd7a	2,6094E-154	0,591888799	0,701	0,221	4,6709E-150	5
Eef1e1	4,6642E-153	0,835772709	0,935	0,503	8,3488E-149	5
Atpif1	4,987E-153	0,833204198	1	0,947	8,9267E-149	5
Lamc3	1,2647E-152	0,648625721	0,734	0,248	2,2639E-148	5
F2rl2	1,4078E-152	0,549477994	0,588	0,168	2,52E-148	5
Bloc1s5	9,3198E-152	0,6180422	0,712	0,252	1,6683E-147	5
Cidea	1,3695E-148	0,803731887	0,981	0,74	2,4513E-144	5
Crip1	1,5422E-148	0,843750121	1	0,985	2,7605E-144	5

Gm13889	3,0101E-148	0,758054296	0,854	0,356	5,3881E-144	5
Qpct	3,1791E-147	0,490681911	0,531	0,141	5,6905E-143	5
Thy1	8,9413E-147	0,874163915	0,962	0,497	1,6005E-142	5
Scg5	2,1357E-146	0,793524806	0,982	0,826	3,8229E-142	5
Sertm1	1,0219E-145	0,542063102	0,651	0,203	1,8292E-141	5
Ncoa7	7,3318E-144	0,836044706	0,969	0,772	1,3124E-139	5
Dlk1	7,8941E-144	0,520011808	0,475	0,117	1,413E-139	5
Lst1	1,3126E-142	0,760499221	0,894	0,447	2,3496E-138	5
Dstn	7,3236E-142	0,746983089	1	0,947	1,3109E-137	5
B3glct	2,1454E-141	0,664800587	0,697	0,259	3,8403E-137	5
Rps21	3,2636E-141	0,86286408	1	0,949	5,8419E-137	5
Hpca	1,0839E-139	0,692786295	0,799	0,322	1,9401E-135	5
Calm2	2,0085E-139	0,57151848	1	1	3,5952E-135	5
Atp6v0e	7,9375E-139	0,754178356	0,91	0,537	1,4208E-134	5
Uqcr11	1,1358E-138	0,777287969	1	0,823	2,033E-134	5
Lgals1	1,6379E-138	0,863400352	0,996	0,899	2,9318E-134	5
Cox7a2l	2,9255E-138	0,731962308	0,969	0,624	5,2367E-134	5
Hint1	1,3439E-137	0,773768873	1	0,876	2,4055E-133	5
Ndufa1	1,4563E-137	0,758870848	0,969	0,584	2,6067E-133	5
Romo1	1,0296E-136	0,728840007	0,994	0,863	1,8429E-132	5
Gm10076	1,0357E-136	0,815502587	1	0,846	1,8538E-132	5
Cox4i1	1,7994E-136	0,761911971	1	0,885	3,2209E-132	5
Cela1	4,9443E-136	0,397240019	0,431	0,102	8,8504E-132	5
Serpinb6a	7,1013E-136	0,696374769	0,991	0,826	1,2711E-131	5
Bglap	4,0439E-135	0,627021252	0,603	0,193	7,2387E-131	5
S100a4	1,0928E-134	0,974074947	0,969	0,731	1,9562E-130	5
Npy1r	4,6259E-133	0,501230381	0,529	0,151	8,2804E-129	5
Stmn3	4,2429E-132	0,770856823	1	0,91	7,5948E-128	5
Rps4x	1,1978E-130	0,720774336	1	0,883	2,1441E-126	5
Rabac1	1,2E-130	0,674522858	1	0,886	2,1481E-126	5
Kcnd2	1,7945E-130	0,609058071	0,85	0,351	3,2122E-126	5
Cox7a2	2,4725E-130	0,707424515	0,999	0,847	4,4258E-126	5
Rps27	6,3068E-130	0,723560802	1	0,865	1,1289E-125	5
Gstm5	7,6545E-130	0,741925697	0,869	0,463	1,3702E-125	5
Cd1d1	1,3799E-129	0,462032293	0,453	0,118	2,47E-125	5
Clec14a	1,9143E-129	0,579453536	0,666	0,238	3,4266E-125	5
Rps7	3,1755E-129	0,717147955	0,999	0,784	5,6841E-125	5
Slc25a4	3,7068E-129	0,646769613	1	0,968	6,6351E-125	5
Psmb5	1,358E-128	0,709932583	0,988	0,767	2,4308E-124	5
Nrsn2	2,086E-128	0,549771084	0,604	0,196	3,7339E-124	5
Acot7	2,6606E-128	0,704466372	0,975	0,716	4,7625E-124	5
S100a6	1,6975E-127	0,71021829	1	0,993	3,0385E-123	5
Fth1	2,249E-127	0,628137362	1	0,993	4,0257E-123	5
Agrp	4,3828E-127	0,418395961	0,306	0,056	7,8453E-123	5
Rpl19	7,0622E-127	0,712069588	1	0,93	1,2641E-122	5
Pfdn5	8,4142E-127	0,699863888	0,982	0,75	1,5061E-122	5

Ssr4	3,2834E-126	0,708140272	0,934	0,563	5,8772E-122	5
Tmem256	3,431E-126	0,706867283	0,937	0,517	6,1416E-122	5
Spock3	3,977E-126	0,735168658	0,957	0,569	7,1188E-122	5
Cox6a1	4,3082E-126	0,681053251	0,999	0,843	7,7117E-122	5
Cntnap5a	8,6812E-126	0,75251561	0,99	0,639	1,5539E-121	5
Psme2	1,4315E-125	0,718830532	0,925	0,549	2,5623E-121	5
Ubl5	2,0135E-125	0,687842999	0,988	0,768	3,6042E-121	5
Cd24a	3,5786E-125	0,789134378	0,996	0,611	6,4058E-121	5
Cox6c	3,5891E-125	0,741955645	0,999	0,869	6,4245E-121	5
Atp5h	5,4502E-125	0,670773399	1	0,867	9,7558E-121	5
Ndufa4	8,0071E-125	0,725889138	0,994	0,835	1,4333E-120	5
Prdx2	1,2555E-124	0,683073599	0,997	0,819	2,2473E-120	5
Chchd2	1,267E-124	0,710253765	0,999	0,916	2,2678E-120	5
Cox8a	1,7145E-123	0,648282838	1	0,929	3,0689E-119	5
Tomm7	2,8846E-123	0,697281051	0,994	0,77	5,1635E-119	5
Tubb4b	3,0688E-123	0,713251604	0,993	0,776	5,4931E-119	5
Dmkn	2,0945E-235	1,297111918	0,907	0,312	3,7492E-231	6
Slc18a3	8,7073E-214	1,265080225	0,98	0,504	1,5586E-209	6
Serping1	4,2563E-202	1,148014021	0,903	0,371	7,6187E-198	6
Calcb	6,9188E-187	1,320146599	0,997	0,518	1,2385E-182	6
Ly6e	4,5373E-178	1,155673686	0,963	0,494	8,1217E-174	6
Sst	8,7207E-171	1,495967423	1	0,821	1,561E-166	6
Gfra2	1,0147E-168	0,998729748	0,956	0,433	1,8164E-164	6
Slc10a4	2,3041E-162	0,989186683	0,993	0,781	4,1243E-158	6
Smarca2	3,1181E-159	0,912525699	0,995	0,934	5,5814E-155	6
Vipr2	1,431E-152	0,846027619	0,732	0,25	2,5614E-148	6
Trp53i11	8,036E-144	0,983485955	0,998	0,656	1,4384E-139	6
Cd81	6,6295E-143	0,737597578	1	0,988	1,1867E-138	6
Nell1	2,5973E-140	0,76857875	0,744	0,262	4,6492E-136	6
Rasd2	4,0011E-139	0,725453738	0,686	0,233	7,162E-135	6
Sphkap	1,1236E-135	0,730905258	0,724	0,252	2,0113E-131	6
P2rx2	5,3516E-132	0,769134841	1	0,964	9,5793E-128	6
Ddah1	3,2611E-131	0,791564365	0,854	0,386	5,8373E-127	6
Wbscr17	1,0689E-129	0,623485743	0,629	0,203	1,9133E-125	6
Sez6l	1,2525E-127	0,850198719	0,876	0,468	2,2419E-123	6
Ache	3,5576E-127	0,77999162	0,998	0,949	6,3681E-123	6
Tpi1	4,1325E-125	0,825829181	0,981	0,874	7,3972E-121	6
Parm1	9,693E-124	0,801438584	0,99	0,868	1,735E-119	6
Krt19	5,9271E-123	0,820610812	0,805	0,335	1,061E-118	6
Fxyd7	1,4012E-122	0,898654681	1	0,746	2,5081E-118	6
Pcdh7	7,5682E-122	0,710355622	0,859	0,357	1,3547E-117	6
Scube1	3,9514E-117	0,765860879	1	0,884	7,0731E-113	6
Samd14	2,2872E-114	0,753338075	0,961	0,687	4,094E-110	6
Aqp1	3,4314E-114	0,62935223	0,583	0,196	6,1423E-110	6
Ly6h	2,4794E-112	0,782153252	0,871	0,47	4,4381E-108	6
Nrp2	7,7274E-112	0,663992189	0,715	0,277	1,3832E-107	6

Piezo1	1,5285E-110	0,572893913	0,583	0,195	2,736E-106	6
Wscd1	4,7226E-110	0,615113259	0,441	0,12	8,4535E-106	6
Pla2g7	9,4988E-110	0,606696043	0,558	0,181	1,7003E-105	6
Rab3b	2,1684E-109	0,726612719	0,871	0,434	3,8814E-105	6
Bche	1,2753E-107	0,741582494	0,981	0,765	2,2828E-103	6
Avpr1a	4,6314E-106	0,607693007	0,636	0,23	8,2901E-102	6
Csrp2	1,4477E-105	0,654597842	0,831	0,384	2,5913E-101	6
Itga6	1,8292E-104	0,707715013	0,876	0,466	3,2742E-100	6
Rspo2	2,6133E-103	0,592866322	0,622	0,223	4,6778E-99	6
Fam19a5	6,8297E-103	0,66883868	0,827	0,373	1,22252E-98	6
Psap	2,505E-102	0,574915128	1	0,996	4,48398E-98	6
Adamts9	5,2804E-102	0,58126823	0,683	0,257	9,4519E-98	6
Emb	7,7826E-102	0,582610425	0,59	0,212	1,39309E-97	6
Mcam	1,2715E-101	0,706439414	0,971	0,649	2,27591E-97	6
Bcl2	8,3688E-101	0,644215585	0,871	0,438	1,49801E-96	6
Spock2	1,8641E-97	0,584316512	1	0,964	3,33674E-93	6
Clstn1	9,09193E-96	0,687449395	0,971	0,842	1,62746E-91	6
Galnt10	1,34943E-95	0,461856	0,432	0,126	2,41548E-91	6
Lynx1	2,17146E-95	0,546340587	0,637	0,246	3,88692E-91	6
Pth1r	1,11585E-94	0,561154292	0,588	0,215	1,99738E-90	6
Npy4r	1,6585E-94	0,401586406	0,286	0,06	2,96871E-90	6
Ptprt	7,84021E-93	0,574734078	0,515	0,176	1,4034E-88	6
Tmem229b	1,11698E-91	0,680830183	0,869	0,506	1,99939E-87	6
Nisch	1,28614E-91	0,532367461	1	0,984	2,3022E-87	6
Tcaf1	1,29911E-90	0,62410716	0,99	0,904	2,32542E-86	6
Timp3	2,09357E-89	0,620862079	0,895	0,465	3,74749E-85	6
Brinp1	9,1901E-89	0,613901535	0,681	0,315	1,64503E-84	6
Cnih3	1,70931E-86	0,443867848	0,458	0,146	3,05967E-82	6
Prph	1,77316E-86	0,539513551	1	0,961	3,17396E-82	6
Gaa	5,02042E-86	0,653833348	0,978	0,832	8,98654E-82	6
Mapk3	4,46731E-84	0,55483571	1	0,985	7,99649E-80	6
Dusp26	1,41678E-83	0,660320496	0,893	0,625	2,53604E-79	6
Rprm	9,03146E-82	0,560223106	0,608	0,254	1,61663E-77	6
Dio2	2,16826E-80	0,465905695	0,398	0,121	3,88119E-76	6
Ass1	4,44626E-80	0,64553562	0,837	0,507	7,95881E-76	6
Caln1	5,99789E-80	0,430919843	0,498	0,173	1,07362E-75	6
Sez6	2,4912E-79	0,562570379	0,69	0,33	4,45924E-75	6
Cct7	2,89056E-79	0,575163938	0,997	0,919	5,1741E-75	6
Susd2	8,56328E-79	0,590681123	0,563	0,235	1,53283E-74	6
Map1lc3a	5,46771E-78	0,514343378	0,998	0,969	9,7872E-74	6
Gcgr	8,28879E-78	0,553459497	0,632	0,284	1,48369E-73	6
Eno2	9,80773E-78	0,616738851	0,947	0,806	1,75558E-73	6
Igfbp5	1,01653E-77	0,476150454	0,546	0,203	1,81959E-73	6
Ryr2	3,61578E-76	0,48390579	0,615	0,259	6,47225E-72	6
Hdac11	1,09545E-75	0,608637593	0,739	0,41	1,96086E-71	6
Sod1	9,33375E-75	0,553224682	0,985	0,916	1,67074E-70	6

Gtf2i	4,77429E-73	0,60712557	0,931	0,71	8,54598E-69	6
Rtp4	1,44715E-72	0,43361295	0,495	0,185	2,5904E-68	6
Calb2	3,37961E-72	0,493971914	1	0,967	6,04951E-68	6
Tmem59l	1,7328E-71	0,571095268	0,697	0,363	3,10172E-67	6
Tle3	2,3071E-71	0,451172842	0,525	0,209	4,12971E-67	6
Kif21a	1,76401E-70	0,508645537	0,995	0,963	3,15757E-66	6
Gsg1l	4,18621E-70	0,421059986	0,385	0,125	7,49331E-66	6
Slc22a17	4,55152E-70	0,553037684	0,988	0,909	8,14723E-66	6
Pkm	9,60755E-70	0,530234573	0,993	0,949	1,71975E-65	6
Atp9a	1,82518E-69	0,517544026	0,983	0,903	3,26707E-65	6
Grina	7,65124E-69	0,518568469	0,985	0,907	1,36957E-64	6
Scarb2	2,32274E-68	0,536891408	0,812	0,482	4,15771E-64	6
St3gal6	2,34762E-68	0,477170964	0,544	0,228	4,20223E-64	6
Rab11fip4	1,23252E-67	0,508889365	0,561	0,258	2,20621E-63	6
6330403K07Rik	2,51422E-67	0,551079813	0,99	0,911	4,50046E-63	6
Fat4	3,68868E-67	0,40732523	0,414	0,142	6,60273E-63	6
Kcnc4	6,2794E-67	0,47030394	0,558	0,241	1,12401E-62	6
Atp5a1	1,36098E-66	0,525798234	0,968	0,872	2,43615E-62	6
Faim2	1,41751E-66	0,568023775	0,705	0,376	2,53734E-62	6
Dpysl5	2,04015E-66	0,524525036	0,814	0,482	3,65187E-62	6
Flot2	2,26731E-66	0,511926468	0,983	0,905	4,05848E-62	6
Calr	9,90754E-66	0,500920723	0,99	0,936	1,77345E-61	6
Vat1l	1,04728E-65	0,538500354	0,956	0,809	1,87462E-61	6
Gdf10	6,05481E-65	0,43558711	0,419	0,15	1,08381E-60	6
Atp1b1	1,6891E-64	0,480293161	0,995	0,869	3,02348E-60	6
Gm42418	4,2911E-148	1,236607591	1	1	7,6811E-144	7
Fibcd1	5,8428E-122	0,990661272	0,824	0,37	1,0459E-117	7
Scgn	6,4944E-104	1,09834421	0,998	0,851	1,1625E-99	7
Npr1	1,3518E-102	0,945513424	0,702	0,314	2,41979E-98	7
Etv1	1,6907E-96	0,878296099	0,994	0,59	3,02636E-92	7
Pxylp1	9,27416E-96	0,896718805	0,886	0,626	1,66007E-91	7
Akap12	6,91743E-86	0,713123125	0,983	0,908	1,23822E-81	7
Cd24a	9,47072E-86	0,920506428	0,968	0,619	1,69526E-81	7
Hap1	1,64786E-79	0,720574812	0,973	0,841	2,94967E-75	7
Auts2	3,94605E-77	0,732187183	0,918	0,669	7,06342E-73	7
Ank2	4,17476E-70	0,496027779	1	0,983	7,47282E-66	7
Cntnap5a	5,57142E-70	0,64089893	0,962	0,646	9,97284E-66	7
Asic2	1,55339E-69	0,673461491	0,918	0,738	2,78057E-65	7
Ngfr	1,80965E-67	0,660021795	0,898	0,533	3,23926E-63	7
Ncam1	5,70798E-66	0,477530512	1	0,986	1,02173E-61	7
F2r	2,5867E-65	0,793339326	0,863	0,551	4,6302E-61	7
Stxbp5	1,72185E-64	0,635339573	0,886	0,665	3,08211E-60	7
Ptgfrn	9,25083E-60	0,447091838	0,427	0,157	1,6559E-55	7
Alpl	4,85499E-59	0,619875987	0,491	0,211	8,69043E-55	7
Tbx3	2,29874E-58	0,53821161	0,994	0,929	4,11475E-54	7
Ascl1	3,7267E-58	0,517255225	0,543	0,237	6,6708E-54	7

Tubb3	5,06707E-58	0,455321974	1	0,988	9,07005E-54	7
Eef1a2	9,19445E-57	0,526875836	0,994	0,942	1,64581E-52	7
Kcnj3	3,04632E-54	0,561990648	0,539	0,25	5,45291E-50	7
Adgrb1	1,21561E-53	0,649692089	0,736	0,481	2,17594E-49	7
Npy	1,5365E-51	0,601374353	1	0,84	2,75034E-47	7
Prkar1b	6,92234E-51	0,616252201	0,907	0,795	1,2391E-46	7
Ehd3	5,07507E-49	0,685970476	0,797	0,63	9,08437E-45	7
Fam155a	1,06924E-48	0,475744324	0,664	0,366	1,91394E-44	7
Ptger4	1,44091E-48	0,559766619	0,712	0,432	2,57923E-44	7
Thy1	3,10267E-48	0,580305612	0,829	0,512	5,55378E-44	7
Synpo2	4,2201E-48	0,411031946	0,425	0,176	7,55399E-44	7
Gpr149	6,87203E-48	0,488934046	0,639	0,335	1,23009E-43	7
L1cam	4,53426E-47	0,457557802	0,996	0,967	8,11632E-43	7
Pwwp2b	8,20713E-47	0,505465668	0,56	0,297	1,46908E-42	7
Grin1	8,04085E-46	0,52696191	0,808	0,577	1,43931E-41	7
Gpr153	2,36785E-43	0,451055091	0,465	0,219	4,23845E-39	7
Parva	3,73615E-43	0,565564196	0,947	0,828	6,68771E-39	7
Slco3a1	5,51329E-42	0,411640146	0,552	0,281	9,86879E-38	7
Map1b	5,5352E-42	0,377291041	1	0,995	9,908E-38	7
Syt7	7,4608E-42	0,506295794	0,917	0,805	1,33548E-37	7
Apba2	3,04277E-41	0,472579252	0,88	0,69	5,44656E-37	7
Chl1	4,06175E-41	0,527160275	0,915	0,751	7,27053E-37	7
Ece1	6,25785E-40	0,486469912	0,879	0,726	1,12015E-35	7
Prokr1	8,57446E-40	0,386311912	0,444	0,203	1,53483E-35	7
Alcam	1,76699E-39	0,457698127	0,884	0,609	3,1629E-35	7
Msn	8,29699E-39	0,482420362	0,939	0,853	1,48516E-34	7
Ntsr1	9,47496E-39	0,508895847	0,56	0,309	1,69602E-34	7
Ap2s1	1,62411E-38	0,402567274	0,979	0,929	2,90716E-34	7
R3hdm1	5,94023E-38	0,397869411	0,937	0,841	1,0633E-33	7
Akt1	8,20278E-38	0,472168483	0,843	0,686	1,4683E-33	7
Arhgap22	2,77093E-36	0,478214263	0,579	0,352	4,95997E-32	7
Ctbp1	3,4275E-36	0,408284651	0,956	0,865	6,13522E-32	7
Spock3	4,32566E-36	0,506186585	0,827	0,583	7,74293E-32	7
Moxd1	1,5451E-35	0,44865642	0,791	0,473	2,76573E-31	7
Vip	3,56585E-35	0,321068915	1	0,922	6,38288E-31	7
Pvrl1	4,81918E-35	0,361908573	0,402	0,189	8,62633E-31	7
Ppp2r1a	9,8311E-35	0,353639375	0,996	0,945	1,75977E-30	7
Dact3	1,17045E-34	0,366249181	0,581	0,333	2,09511E-30	7
Pdlim5	1,31002E-34	0,418674286	0,615	0,369	2,34493E-30	7
Tmod1	5,00226E-34	0,533678782	0,778	0,612	8,95405E-30	7
Ptprn	5,41738E-34	0,344663324	1	0,993	9,6971E-30	7
Tgfb1	5,63217E-33	0,31950293	0,603	0,325	1,00816E-28	7
Lars2	2,66846E-32	0,478271214	0,991	0,989	4,77655E-28	7
9530059O14Rik	1,92467E-31	0,363170508	0,892	0,73	3,44515E-27	7
Cmip	6,77467E-31	0,390141123	0,947	0,844	1,21267E-26	7
Kcnab2	1,5084E-30	0,453164509	0,583	0,364	2,70003E-26	7

Plekha5	1,66781E-30	0,369294512	0,82	0,627	2,98539E-26	7
Gfra1	1,79987E-30	0,436175739	0,732	0,456	3,22178E-26	7
Lamc3	4,90926E-30	0,346966012	0,501	0,269	8,78757E-26	7
Rimbp2	1,4541E-29	0,335550233	0,512	0,286	2,60285E-25	7
Pde10a	2,18132E-29	0,439657544	0,879	0,775	3,90456E-25	7
Dach1	3,86596E-29	0,291515312	0,343	0,159	6,92007E-25	7
Apba1	4,07145E-29	0,381053988	0,858	0,722	7,2879E-25	7
Ptpru	4,79776E-29	0,277282871	0,309	0,135	8,58798E-25	7
Strip1	5,2216E-29	0,349129081	0,653	0,434	9,34667E-25	7
Eif4a1	7,8137E-29	0,342609548	0,972	0,971	1,39865E-24	7
Sdcbp	1,84794E-28	0,427966755	0,935	0,868	3,30781E-24	7
Fscn1	7,22728E-28	0,441105961	0,761	0,619	1,29368E-23	7
Nrep	7,35236E-28	0,404645481	0,579	0,368	1,31607E-23	7
Ints3	1,63588E-27	0,401634885	0,655	0,466	2,92823E-23	7
Chd5	2,48973E-27	0,358327129	0,996	0,947	4,45662E-23	7
Hspb8	4,33049E-27	0,426049054	0,972	0,935	7,75157E-23	7
Dclk2	1,20637E-26	0,403166181	0,634	0,435	2,1594E-22	7
Socs2	1,84113E-26	0,349891142	0,584	0,356	3,29562E-22	7
Garnl3	2,06473E-26	0,367767864	0,846	0,707	3,69588E-22	7
Man2a1	3,08147E-26	0,382749446	0,915	0,824	5,51582E-22	7
Arvcf	6,49055E-26	0,452668751	0,774	0,656	1,16181E-21	7
Nav3	1,15402E-25	0,335929995	0,571	0,357	2,0657E-21	7
Galnt6	1,25726E-25	0,319888851	0,406	0,211	2,25049E-21	7
Mtss1	1,60463E-25	0,401453316	0,626	0,435	2,87229E-21	7
Entpd3	1,67721E-25	0,402468414	0,584	0,383	3,0022E-21	7
Cald1	1,8347E-25	0,318861274	0,51	0,314	3,28412E-21	7
Zfhx4	3,18821E-25	0,377819846	0,59	0,383	5,70689E-21	7
Sod1	3,96561E-25	0,412311129	0,949	0,919	7,09844E-21	7
Fbn1	4,30956E-25	0,275164648	0,514	0,298	7,71411E-21	7
Nrp1	5,05238E-25	0,371281219	0,886	0,764	9,04376E-21	7
Lmna	1,06124E-24	0,341571673	0,943	0,866	1,89962E-20	7
Kcnt2	1,09689E-24	0,29213501	0,533	0,308	1,96344E-20	7
Plxna4	1,19218E-24	0,338006592	0,991	0,939	2,134E-20	7
Ppp6r1	1,57672E-24	0,3891126	0,719	0,551	2,82233E-20	7
Cd9	6,9598E-237	1,375786508	1	0,984	1,2458E-232	8
Bglap	4,1409E-198	1,182611796	0,723	0,192	7,4121E-194	8
Tuba1a	2,2483E-185	1,168065856	1	0,985	4,0245E-181	8
Vip	5,1037E-177	1,574979768	1	0,922	9,1355E-173	8
Cox7c	5,9344E-172	0,988865795	1	0,957	1,0623E-167	8
Gapdh	9,0413E-172	1,092617474	1	0,927	1,6184E-167	8
Ubb	4,1134E-159	0,991440564	1	0,986	7,3629E-155	8
Sh3bgrl3	7,696E-159	0,994751735	0,989	0,62	1,3776E-154	8
Atpif1	3,9854E-156	0,967838568	1	0,948	7,1339E-152	8
Stmn3	2,8669E-155	0,98719126	1	0,911	5,1318E-151	8
Igfbp7	4,1425E-154	0,71271894	0,949	0,379	7,4151E-150	8
Mpc1	5,419E-154	0,966536257	0,994	0,818	9,6999E-150	8

Uchl1	3,5254E-149	0,641114328	1	0,991	6,3105E-145	8
Ppa1	4,9793E-146	0,953193048	0,962	0,568	8,9129E-142	8
Slc25a4	5,0966E-144	0,79529567	1	0,968	9,123E-140	8
Prdx2	2,2172E-139	0,86933294	0,994	0,823	3,9688E-135	8
Rps21	3,7497E-139	0,970807052	1	0,949	6,712E-135	8
Rpl39	5,0931E-138	0,925492962	1	0,935	9,1167E-134	8
Tmsb4x	1,1807E-137	0,645844446	1	0,998	2,1135E-133	8
Calm2	9,2386E-137	0,656907813	1	1	1,6537E-132	8
Rpl19	1,529E-136	0,864563423	1	0,931	2,737E-132	8
Rps27	7,6366E-135	0,880332035	1	0,867	1,3669E-130	8
Rpl9	1,3041E-134	0,911013146	1	0,821	2,3344E-130	8
Tgfb1	1,9596E-134	0,77090355	0,827	0,312	3,5077E-130	8
Scgn	3,3101E-133	1,247336412	1	0,851	5,9251E-129	8
Dstn	2,2667E-132	0,842264078	1	0,948	4,0574E-128	8
Hint1	3,9336E-132	0,873389331	1	0,879	7,0411E-128	8
Gm10076	1,1936E-131	0,938009939	1	0,849	2,1365E-127	8
Nsg1	3,3786E-131	0,802425469	1	0,971	6,0476E-127	8
Rps4x	4,857E-131	0,848564159	1	0,885	8,6941E-127	8
Crip1	1,925E-130	0,909245431	1	0,985	3,4458E-126	8
Rps7	2,4025E-130	0,839839145	1	0,787	4,3006E-126	8
Rpl14	2,5296E-130	0,721419496	1	0,981	4,5279E-126	8
Chchd2	5,9054E-128	0,819098199	1	0,917	1,0571E-123	8
Atp5j2	6,5178E-128	0,885342173	0,998	0,796	1,1667E-123	8
Cidea	1,2568E-127	0,866690161	0,992	0,743	2,2497E-123	8
Sec61g	9,272E-127	0,831805374	1	0,87	1,6597E-122	8
Tmsb10	3,9378E-125	0,813565354	1	0,946	7,0487E-121	8
Npy	5,0873E-125	1,279975997	0,992	0,84	9,1063E-121	8
Fxyd5	2,2087E-124	0,915152391	0,88	0,405	3,9535E-120	8
Ttc9b	5,1754E-124	0,835970324	0,939	0,558	9,264E-120	8
Cox4i1	5,1918E-124	0,833317999	1	0,887	9,2934E-120	8
Atp5k	1,5109E-123	0,849453083	1	0,957	2,7046E-119	8
Id3	1,936E-123	0,863902408	0,909	0,448	3,4654E-119	8
Fau	4,6451E-123	0,776171237	1	0,922	8,3147E-119	8
S100a6	9,6929E-123	0,8135295	1	0,993	1,735E-118	8
Romo1	1,9283E-122	0,785195388	0,996	0,865	3,4516E-118	8
Ndufa1	1,9782E-122	0,840944054	0,975	0,59	3,5409E-118	8
Rps24	1,9937E-122	0,729779016	1	0,966	3,5687E-118	8
Rps28	2,1019E-122	0,797843691	1	0,829	3,7625E-118	8
Gabarapl2	1,1693E-121	0,781222333	0,998	0,862	2,093E-117	8
Cox6b1	1,9008E-120	0,780402252	0,998	0,869	3,4024E-116	8
Cox6c	4,5945E-120	0,821809289	1	0,871	8,2242E-116	8
Cox7a2	1,1995E-119	0,785602705	0,996	0,85	2,147E-115	8
Dync1i2	1,2117E-119	0,622773078	1	0,998	2,169E-115	8
Rps23	5,9786E-119	0,762398599	1	0,903	1,0702E-114	8
Basp1	2,7211E-118	0,625867734	1	0,982	4,8708E-114	8
Pfdn5	1,355E-117	0,820898053	0,996	0,753	2,4255E-113	8

Rpl35a	7,8965E-117	0,748851406	1	0,922	1,4135E-112	8
Myl1	4,7064E-116	0,794092918	0,996	0,908	8,4245E-112	8
Atp5f1	4,8349E-116	0,794890057	0,977	0,676	8,6544E-112	8
Ndufa4	8,436E-116	0,800238437	0,998	0,837	1,51E-111	8
Eef1e1	9,0859E-116	0,825182819	0,928	0,51	1,6264E-111	8
Rpl22l1	1,8808E-115	0,793208796	0,996	0,778	3,3666E-111	8
Rps27a	2,8316E-115	0,760622823	1	0,928	5,0686E-111	8
Tubb4b	4,5215E-115	0,815742015	0,991	0,78	8,0935E-111	8
Fth1	6,1441E-115	0,690093914	1	0,994	1,0998E-110	8
Rps8	7,5761E-115	0,681084678	1	0,977	1,3561E-110	8
Ndufb10	8,1882E-115	0,767264855	0,949	0,519	1,4657E-110	8
Atp5e	5,0332E-114	0,832140799	0,998	0,845	9,0094E-110	8
Uqcrh	5,5531E-114	0,766533143	0,989	0,772	9,94E-110	8
Rabac1	8,6102E-114	0,708526021	0,998	0,888	1,5412E-109	8
S100a4	3,3049E-113	1,096272413	0,962	0,736	5,9158E-109	8
Uqcr11	7,4049E-113	0,793454511	1	0,826	1,3255E-108	8
Lst1	7,4604E-112	0,800570812	0,886	0,455	1,3354E-107	8
Phlda3	6,9581E-111	0,790401958	0,964	0,625	1,2455E-106	8
Rps29	1,6045E-110	0,710663011	1	0,996	2,8721E-106	8
Rpl41	3,0338E-110	0,601076163	1	0,998	5,4306E-106	8
Stmn2	3,3432E-110	0,537803009	1	0,99	5,9843E-106	8
Cryab	4,6442E-110	0,755197363	0,839	0,394	8,3131E-106	8
Cox5a	1,4609E-109	0,761914735	0,97	0,637	2,6149E-105	8
Hist3h2ba	2,1002E-109	0,767600857	0,867	0,447	3,7593E-105	8
Tubb2a	2,2107E-109	0,628363674	1	0,983	3,9572E-105	8
Ftl1	3,5905E-109	0,71189322	1	0,928	6,4271E-105	8
Ndufa7	4,2502E-109	0,767114201	0,994	0,704	7,6079E-105	8
Rpl37a	5,1951E-109	0,696026814	1	0,986	9,2991E-105	8
Rps2	9,1965E-109	0,709382722	1	0,847	1,6462E-104	8
Cuedc2	1,0238E-108	0,758440607	0,934	0,52	1,8326E-104	8
Mrpl20	1,1718E-108	0,72465535	0,884	0,444	2,0975E-104	8
Rps3a1	3,1276E-108	0,748525979	0,998	0,836	5,5985E-104	8
Fdps	9,7931E-108	0,843121638	0,983	0,74	1,753E-103	8
Rps20	1,0381E-107	0,657567691	1	0,9	1,8583E-103	8
Rps3	1,5166E-107	0,722991319	0,996	0,867	2,7147E-103	8
Rpl17	4,4352E-107	0,661726665	1	0,915	7,939E-103	8
Ndufc1	5,3249E-107	0,736581096	1	0,817	9,5316E-103	8
Rpl35	1,0465E-106	0,763167307	1	0,831	1,8732E-102	8
Psmb5	1,0526E-106	0,748490762	0,991	0,771	1,8841E-102	8
Rpl10	1,3466E-106	0,690257524	1	0,878	2,4104E-102	8
Cox6a1	3,1056E-106	0,729388718	0,994	0,846	5,559E-102	8
Rps13	3,2072E-106	0,686737013	1	0,825	5,741E-102	8
Ldha	9,7446E-106	0,632467875	0,998	0,97	1,7443E-101	8
Cox8b	0	2,017426571	0,714	0,056	0	9
Tuba1a	3,1592E-173	1,242262976	1	0,985	5,655E-169	9
Tmsb4x	3,0261E-167	0,808392074	1	0,998	5,4167E-163	9

Calm2	1,023E-164	0,839713611	1	1	1,8312E-160	9
Lgals1	5,8152E-150	1,174277857	0,998	0,901	1,0409E-145	9
S100a4	8,2721E-147	1,383104095	0,971	0,736	1,4807E-142	9
Alpl	7,6153E-143	0,800010853	0,68	0,202	1,3631E-138	9
Atpif1	3,5109E-141	1,005321765	1	0,948	6,2844E-137	9
Rps24	2,0962E-135	0,894749783	1	0,966	3,7523E-131	9
Crip1	3,3276E-135	1,046740928	1	0,985	5,9565E-131	9
Rpl41	1,8306E-134	0,732467576	1	0,998	3,2767E-130	9
Uchl1	3,295E-130	0,647173128	1	0,991	5,898E-126	9
Rpl14	1,1731E-129	0,77873879	1	0,981	2,0998E-125	9
Gapdh	2,0261E-125	0,977956173	1	0,927	3,6268E-121	9
Smpd3	5,0461E-124	0,736814237	1	0,992	9,0326E-120	9
Ubb	1,5295E-123	0,926367741	1	0,986	2,7379E-119	9
Rtn4rl1	5,9983E-123	0,615146687	0,535	0,136	1,0737E-118	9
S100a6	4,1796E-122	0,862604649	1	0,993	7,4815E-118	9
Cd9	5,3801E-122	0,925431627	1	0,984	9,6304E-118	9
Cox7c	8,8653E-122	0,912488647	1	0,957	1,5869E-117	9
Rpl39	1,2071E-119	0,930875261	1	0,935	2,1606E-115	9
Csrp1	1,4797E-119	0,838452356	1	0,951	2,6487E-115	9
Tmsb10	3,226E-119	0,863710416	1	0,946	5,7745E-115	9
Dpysl3	5,1422E-117	0,748675105	1	0,977	9,2046E-113	9
Rpl6	9,824E-116	0,860400072	1	0,971	1,7585E-111	9
H3f3a	3,8302E-115	0,726256453	1	0,987	6,856E-111	9
Dync1i2	5,3085E-115	0,693588931	1	0,998	9,5022E-111	9
Rpl19	1,0664E-114	0,851955147	1	0,931	1,9088E-110	9
Rpl38	4,273E-114	0,807930398	1	0,993	7,6486E-110	9
Ldha	2,3109E-112	0,783131204	1	0,97	4,1365E-108	9
Vim	2,6433E-112	0,911766161	0,934	0,604	4,7314E-108	9
Igfbp7	1,9537E-110	0,646670013	0,88	0,385	3,4971E-106	9
Dstn	2,57E-109	0,813405891	1	0,948	4,6003E-105	9
Rpl35	1,0738E-108	0,857782754	0,996	0,833	1,9222E-104	9
Ppia	7,3801E-107	0,579173649	1	0,999	1,321E-102	9
Rps29	1,8633E-104	0,735993354	1	0,996	3,3353E-100	9
Rps21	2,3001E-104	0,886168982	1	0,95	4,1172E-100	9
Ftl1	2,9367E-103	0,790471078	0,998	0,928	5,2568E-99	9
Basp1	5,2562E-102	0,636500539	1	0,982	9,40864E-98	9
Stmn3	3,5695E-101	0,831413576	1	0,912	6,38947E-97	9
Hint1	4,6891E-101	0,825686119	1	0,879	8,3935E-97	9
Prdx2	5,8846E-101	0,828319512	0,988	0,824	1,05335E-96	9
Rps9	1,8732E-100	0,738580475	1	0,919	3,35295E-96	9
Stmn2	2,2489E-100	0,589402281	1	0,99	4,02552E-96	9
Cidea	4,4907E-100	0,843878885	0,967	0,746	8,03826E-96	9
Rpl37a	2,86507E-98	0,695627377	1	0,986	5,12848E-94	9
Tubb2b	5,86715E-98	0,698051468	1	0,969	1,05022E-93	9
Gm10076	7,93559E-98	0,886135282	1	0,849	1,42047E-93	9
Fabp5	3,26976E-97	0,670482668	1	0,968	5,85287E-93	9

Rpl13	2,68519E-96	0,703227881	1	0,955	4,80649E-92	9
Rps23	5,91306E-96	0,722025628	1	0,904	1,05844E-91	9
Nsg1	1,86065E-95	0,7362561	1	0,971	3,33056E-91	9
Areg	2,80472E-95	0,518429541	0,297	0,056	5,02046E-91	9
Etv1	3,26077E-95	0,915531926	0,996	0,592	5,83677E-91	9
Atp5e	6,15959E-95	0,804639855	1	0,845	1,10257E-90	9
Tubb2a	5,43784E-94	0,645159654	1	0,983	9,73373E-90	9
Rpl37	1,61613E-93	0,716521485	1	0,986	2,89287E-89	9
Rps11	3,07485E-93	0,659255716	1	0,934	5,50399E-89	9
Cox4i1	4,46995E-93	0,762385108	1	0,887	8,00122E-89	9
Rpl11	8,16754E-93	0,66796837	1	0,952	1,46199E-88	9
Atp5k	1,12416E-92	0,757138815	1	0,957	2,01225E-88	9
Rps16	1,18132E-92	0,650839816	1	0,924	2,11456E-88	9
Bglap	1,42728E-92	0,73438598	0,585	0,202	2,55483E-88	9
Rps4x	7,33642E-92	0,746882124	1	0,885	1,31322E-87	9
Rpl18	1,05142E-91	0,698571693	1	0,869	1,88204E-87	9
Dynll1	2,13672E-91	0,648427848	1	0,982	3,82474E-87	9
Fau	3,67962E-91	0,707223938	1	0,922	6,58652E-87	9
Rps8	3,72648E-91	0,680180702	1	0,977	6,6704E-87	9
Id3	1,01891E-90	0,810679755	0,855	0,453	1,82385E-86	9
Rps28	1,13678E-90	0,737037008	0,996	0,83	2,03484E-86	9
Rpl34	1,87562E-90	0,678539293	1	0,917	3,35736E-86	9
Rpl9	5,22486E-90	0,797056641	0,992	0,822	9,35249E-86	9
Cox6b1	7,15244E-90	0,704125364	0,992	0,87	1,28029E-85	9
Rpl32	7,86857E-90	0,608093034	1	0,967	1,40847E-85	9
Rpl27a	1,15025E-89	0,616140106	1	0,962	2,05894E-85	9
Rps12	1,28671E-89	0,69119266	0,99	0,833	2,30321E-85	9
Ramp2	7,63507E-89	0,648390267	0,573	0,205	1,36668E-84	9
Rps27a	4,79709E-88	0,716114591	0,998	0,929	8,5868E-84	9
Stmn1	9,9653E-88	0,743499488	0,988	0,886	1,78379E-83	9
Rplp2	9,02584E-87	0,651252475	0,998	0,931	1,61563E-82	9
Thy1	2,21341E-86	0,729527463	0,94	0,508	3,962E-82	9
Rps18	4,17846E-84	0,632684503	1	0,933	7,47945E-80	9
Rpl24	5,62191E-84	0,678956164	1	0,945	1,00632E-79	9
Rps10	1,64084E-83	0,641495981	1	0,92	2,9371E-79	9
S100a13	4,91364E-82	0,735412075	0,988	0,886	8,79542E-78	9
Eef1a1	1,07374E-81	0,518785629	1	0,999	1,92199E-77	9
Ttc9b	1,58024E-81	0,767398659	0,876	0,564	2,82863E-77	9
Cryab	2,7374E-81	0,74903167	0,786	0,399	4,89994E-77	9
Lst1	3,17787E-81	0,716229167	0,853	0,459	5,68839E-77	9
Uqcr11	3,95094E-81	0,702765639	0,998	0,827	7,07218E-77	9
Atp5j2	3,18747E-80	0,732402258	0,992	0,797	5,70558E-76	9
Rps5	3,87816E-80	0,660187513	0,996	0,887	6,94191E-76	9
Romo1	4,22431E-80	0,673323692	0,988	0,866	7,56151E-76	9
Cox7a2	4,71414E-80	0,686792334	0,994	0,851	8,43831E-76	9
Tceb2	5,27213E-80	0,599540625	0,998	0,954	9,43711E-76	9

Rps27	6,65243E-80	0,660281464	0,998	0,868	1,19078E-75	9
Rplp0	7,23691E-80	0,705535601	0,971	0,785	1,29541E-75	9
Rps19	1,12574E-79	0,54712985	0,998	0,944	2,01507E-75	9
Eln	3,10802E-79	0,265072741	0,309	0,068	5,56336E-75	9
S100a16	4,5986E-79	0,680674983	0,99	0,889	8,2315E-75	9
Rps7	4,88748E-78	0,703336049	0,983	0,789	8,74858E-74	9
Eif2s2	7,51395E-78	0,643857931	0,994	0,912	1,345E-73	9
Sst	9,2149E-180	1,818741913	1	0,824	1,6495E-175	10
Ifitm2	1,9816E-102	0,875712564	1	0,941	3,54708E-98	10
Tshz2	7,7909E-101	0,928415097	0,996	0,838	1,39458E-96	10
Rpl6	1,394E-99	0,83157259	1	0,971	2,49531E-95	10
Fxyd7	2,87309E-95	0,904527183	1	0,75	5,14283E-91	10
Rpl38	1,35885E-94	0,750389796	1	0,993	2,43234E-90	10
Atp5k	3,70717E-88	0,793351596	1	0,957	6,63583E-84	10
Kif22	1,03488E-81	0,917517858	0,942	0,721	1,85243E-77	10
Csrp2	4,69673E-78	0,740121914	0,777	0,393	8,40715E-74	10
Rps29	1,70839E-75	0,645845604	1	0,996	3,05801E-71	10
Ptma	9,33192E-73	0,484784649	1	1	1,67041E-68	10
Slc18a3	2,19833E-70	0,729737595	0,874	0,516	3,93501E-66	10
Rpl37	4,61319E-70	0,652536599	1	0,986	8,2576E-66	10
Dmkn	3,04554E-69	0,678988265	0,74	0,329	5,45152E-65	10
Ahi1	7,49115E-69	0,58676021	1	0,99	1,34092E-64	10
Hmgb1	2,18086E-65	0,574129576	0,998	0,973	3,90373E-61	10
Ubb	5,04639E-61	0,647916926	1	0,986	9,03303E-57	10
Ap2a2	6,15568E-61	0,558236592	0,996	0,972	1,10187E-56	10
Ache	7,2678E-59	0,641412842	0,991	0,95	1,30094E-54	10
Calcb	2,02319E-58	0,584809217	0,95	0,527	3,6215E-54	10
Vipr2	7,74306E-58	0,56384827	0,602	0,264	1,38601E-53	10
S100a1	2,06798E-57	0,649862245	0,961	0,822	3,70168E-53	10
Ifi27	2,32489E-57	0,638235664	0,97	0,778	4,16156E-53	10
Cyth3	6,15325E-57	0,5240729	1	0,982	1,10143E-52	10
Prph	8,77566E-56	0,531672103	1	0,962	1,57084E-51	10
Tceb2	9,12044E-56	0,55073309	1	0,954	1,63256E-51	10
Tmsb10	8,11549E-55	0,550847479	1	0,946	1,45267E-50	10
Ly6h	1,4507E-54	0,632229055	0,788	0,48	2,59676E-50	10
Mrpl52	8,58176E-54	0,585371399	0,946	0,771	1,53614E-49	10
Atp5e	1,07673E-52	0,601539971	0,996	0,846	1,92735E-48	10
Ndufc1	5,0617E-52	0,564863284	0,985	0,819	9,06045E-48	10
Rpl13	2,17288E-51	0,494763864	1	0,955	3,88946E-47	10
S100a6	1,98222E-50	0,530910981	1	0,993	3,54818E-46	10
Serping1	2,55187E-50	0,573734432	0,706	0,389	4,56784E-46	10
Gfra2	1,08562E-49	0,529057793	0,829	0,447	1,94326E-45	10
Ndufa5	1,4387E-48	0,56710503	0,963	0,804	2,57527E-44	10
Pcdh7	1,64888E-48	0,536381702	0,725	0,371	2,95149E-44	10
Ncl	1,66513E-48	0,549952238	0,998	0,957	2,98058E-44	10
Rpl32	9,11402E-48	0,444642654	1	0,967	1,63141E-43	10

Rpl36	1,12431E-47	0,493818686	0,998	0,946	2,01251E-43	10
Ntm	2,73262E-47	0,542727831	0,394	0,144	4,89139E-43	10
Zc3h15	5,41459E-47	0,517974414	0,976	0,896	9,69211E-43	10
Ly6e	1,38755E-46	0,49303399	0,833	0,507	2,48372E-42	10
Tcf7l2	6,45797E-46	0,500280482	0,981	0,838	1,15598E-41	10
Rab3b	9,28592E-46	0,51338839	0,76	0,446	1,66218E-41	10
Atp6v1e1	1,09188E-45	0,473549369	1	0,953	1,95446E-41	10
Atpif1	5,82673E-45	0,514095817	1	0,949	1,04299E-40	10
Smarca2	2,03597E-44	0,502589941	0,996	0,935	3,64439E-40	10
Trp53i11	1,94653E-43	0,407056335	0,981	0,661	3,48429E-39	10
Psme1	8,9017E-43	0,534298708	0,976	0,845	1,5934E-38	10
1110004F10Rik	1,08958E-42	0,506306536	0,987	0,925	1,95034E-38	10
Pdcd5	1,56791E-42	0,515736082	0,92	0,778	2,80655E-38	10
Meg3	2,84476E-42	0,280420977	1	0,993	5,09211E-38	10
mt-Nd2	1,28409E-41	0,305774743	1	1	2,29851E-37	10
Metap2	6,32953E-41	0,462517323	0,994	0,964	1,13299E-36	10
Rpl31	1,14348E-39	0,467095196	0,968	0,879	2,04683E-35	10
Snrnp27	1,27455E-39	0,490807883	0,959	0,886	2,28145E-35	10
Ndufa3	1,49604E-39	0,482324234	0,974	0,775	2,67791E-35	10
Ptms	2,16359E-39	0,36870706	1	0,997	3,87283E-35	10
Nap1l1	3,77475E-39	0,522080448	0,89	0,727	6,75681E-35	10
Rplp1	7,09177E-39	0,371692492	1	0,977	1,26943E-34	10
Rgs9	4,03738E-38	0,48434518	0,879	0,731	7,22691E-34	10
Dync1i2	4,40763E-38	0,378879536	1	0,998	7,88966E-34	10
Snhg11	6,71419E-38	0,349662657	1	0,983	1,20184E-33	10
Uqcr10	7,34167E-38	0,470948146	0,95	0,748	1,31416E-33	10
Tomm7	1,28171E-37	0,445013621	0,97	0,777	2,29426E-33	10
Krt19	2,24443E-37	0,339665718	0,645	0,349	4,01752E-33	10
Ndufs5	2,46712E-37	0,454794871	0,974	0,851	4,41614E-33	10
Rpl36al	2,67087E-37	0,450212838	0,968	0,837	4,78085E-33	10
Pdap1	5,04377E-37	0,448236071	0,994	0,934	9,02834E-33	10
Rpl39	7,24845E-37	0,421346839	1	0,936	1,29747E-32	10
Hand2	9,86773E-37	0,411665016	1	0,976	1,76632E-32	10
Skp1a	1,01559E-36	0,438325142	0,968	0,908	1,81791E-32	10
Atp1a1	1,35748E-36	0,384583227	1	0,996	2,42988E-32	10
Ndufa2	3,89182E-36	0,461753525	0,985	0,883	6,96635E-32	10
Pcsk1n	4,44789E-36	0,35102934	1	0,995	7,96172E-32	10
Rpl37a	8,03112E-36	0,373156217	1	0,986	1,43757E-31	10
mt-Nd1	1,6174E-35	0,290913924	1	1	2,89515E-31	10
Uchl1	1,6493E-35	0,333095439	1	0,991	2,95226E-31	10
Fam19a5	1,72218E-35	0,447280802	0,684	0,387	3,0827E-31	10
Nisch	1,94274E-35	0,385205437	1	0,984	3,4775E-31	10
Wscd1	2,53402E-35	0,349850427	0,335	0,13	4,5359E-31	10
Rpl24	3,43466E-35	0,427171761	1	0,946	6,14805E-31	10
Rpl18	6,83527E-35	0,382622824	0,991	0,87	1,22351E-30	10
Rpl35	1,47304E-34	0,410264008	0,994	0,833	2,63674E-30	10

Map1lc3a	2,06741E-34	0,413144256	0,994	0,97	3,70066E-30	10
Rspo2	2,42655E-34	0,381398944	0,494	0,235	4,34352E-30	10
Uqcr11	5,77534E-34	0,43679398	0,985	0,828	1,03379E-29	10
Hoxa5	9,83022E-34	0,351096352	0,981	0,929	1,75961E-29	10
Lars2	1,54693E-33	0,365329789	1	0,988	2,76901E-29	10
Fau	2,0043E-33	0,399340601	1	0,922	3,58769E-29	10
Rpl23	2,12446E-33	0,317447841	1	0,992	3,80278E-29	10
Pcdh17	3,053E-33	0,508268319	0,926	0,821	5,46487E-29	10
Eif4a2	9,95932E-33	0,369669476	0,994	0,974	1,78272E-28	10
Dynlrb1	1,032E-32	0,418084888	0,983	0,966	1,84728E-28	10
Ndufa13	1,86182E-32	0,426739125	0,965	0,808	3,33266E-28	10
Serf1	2,46534E-32	0,466798509	0,918	0,792	4,41297E-28	10
B3gnt2	1,03951E-31	0,408440082	0,509	0,274	1,86071E-27	10
St13	3,40628E-31	0,408605762	0,976	0,917	6,09724E-27	10
Nrp2	3,98954E-31	0,364564212	0,558	0,292	7,14128E-27	10
mt-Nd3	8,0368E-31	0,354246769	1	0,999	1,43859E-26	10
Gm42418	7,7356E-186	1,87658478	1	1	1,3847E-181	11
Lars2	1,2859E-146	1,18483638	1	0,988	2,3017E-142	11
Tshz2	2,293E-82	0,935119957	0,997	0,839	4,10446E-78	11
Ntm	1,0733E-77	0,853296059	0,491	0,142	1,92121E-73	11
Trp53i11	2,43118E-60	0,698406526	0,997	0,664	4,35181E-56	11
Nfix	1,27055E-59	0,735667298	0,976	0,747	2,27429E-55	11
Apba2	7,9772E-59	0,81242714	0,887	0,693	1,42792E-54	11
AY036118	9,93287E-59	0,722324031	0,987	0,847	1,77798E-54	11
Hoxa5	3,13667E-49	0,580573026	1	0,929	5,61464E-45	11
Tcf7l2	5,2791E-44	0,521103528	0,984	0,839	9,44958E-40	11
Sst	1,22827E-43	0,608967893	1	0,825	2,1986E-39	11
Ptms	4,8708E-43	0,434006826	1	0,997	8,71874E-39	11
Fxyd7	2,27855E-42	0,616475822	1	0,752	4,0786E-38	11
mt-Nd2	1,63404E-40	0,372285622	1	1	2,92493E-36	11
mt-Nd1	2,99866E-39	0,374190952	1	1	5,3676E-35	11
Pcsk1n	1,93333E-37	0,438810589	1	0,996	3,46066E-33	11
Mcam	1,20141E-36	0,548403438	0,909	0,659	2,15052E-32	11
mt-Nd4l	1,36987E-36	0,356897572	1	1	2,45208E-32	11
Scube1	3,75306E-32	0,444499905	1	0,887	6,71797E-28	11
Tnrc6c	3,91229E-29	0,454697176	0,925	0,838	7,00299E-25	11
Smarca2	1,14617E-28	0,451783602	0,997	0,935	2,05164E-24	11
mt-Nd3	2,13817E-28	0,356580567	1	0,999	3,82733E-24	11
mt-Atp8	2,48027E-27	0,325868514	1	1	4,43968E-23	11
Dst	2,00592E-26	0,35904236	1	0,991	3,59061E-22	11
Ctbp1	2,74332E-26	0,412171397	0,949	0,867	4,91055E-22	11
Gpatch8	5,89608E-25	0,440395187	0,903	0,84	1,0554E-20	11
Igfbp5	1,40496E-21	0,348337779	0,426	0,216	2,51487E-17	11
Pcdh7	1,582E-21	0,382727205	0,627	0,378	2,83177E-17	11
Nrxn2	3,02906E-21	0,384180953	0,987	0,947	5,42202E-17	11
Ankrd11	1,90073E-20	0,414996641	0,928	0,866	3,4023E-16	11

Camk2b	3,63023E-20	0,416053347	0,826	0,722	6,49811E-16	11
Maz	1,35922E-19	0,3461045	0,941	0,857	2,433E-15	11
Vamp1	2,79477E-19	0,367425006	0,917	0,788	5,00263E-15	11
Flywch1	4,07322E-19	0,429875889	0,834	0,779	7,29106E-15	11
Casz1	3,29076E-18	0,306913508	0,611	0,378	5,89046E-14	11
Smarcc2	5,86692E-18	0,405482788	0,807	0,715	1,05018E-13	11
Gfra2	1,06126E-17	0,277012591	0,74	0,454	1,89966E-13	11
Gm26917	2,29143E-17	0,49015595	0,662	0,519	4,10166E-13	11
Enah	2,60253E-17	0,284116701	0,946	0,866	4,65853E-13	11
Chd5	8,71476E-17	0,305817604	0,981	0,948	1,55994E-12	11
Rspo2	1,08911E-16	0,395223842	0,424	0,241	1,94952E-12	11
Hoxb5	1,67935E-16	0,269503741	0,995	0,944	3,00604E-12	11
Rph3a	6,89768E-16	0,401808664	0,735	0,627	1,23468E-11	11
Tmem63b	2,36994E-15	0,304064708	0,836	0,743	4,24219E-11	11
Chd3	4,69868E-15	0,311542613	0,984	0,945	8,41063E-11	11
Nrxn3	5,4056E-15	0,274276596	0,651	0,433	9,67603E-11	11
Bnc2	6,99694E-15	0,324585122	0,399	0,231	1,25245E-10	11
Plod2	1,01956E-14	0,341373689	0,885	0,822	1,82501E-10	11
Clk1	4,01962E-14	0,313934468	0,847	0,802	7,19512E-10	11
Eef1a2	4,3925E-14	0,300690608	0,995	0,943	7,86258E-10	11
Filip1	6,4917E-14	0,390841523	0,853	0,804	1,16201E-09	11
Pnisr	1,39561E-13	0,265720566	0,823	0,736	2,49813E-09	11
Adamts9	1,44224E-13	0,2610827	0,458	0,277	2,58161E-09	11
Ina	4,60945E-13	0,313225196	0,804	0,734	8,25092E-09	11
Dlgap3	1,8756E-12	0,388727629	0,542	0,425	3,35732E-08	11
Ahi1	2,7187E-11	0,259611234	0,997	0,99	4,86647E-07	11
Begain	5,90311E-11	0,354651101	0,601	0,501	1,05666E-06	11
PISD	5,48167E-10	0,290056656	0,786	0,738	9,81219E-06	11
Satb2	2,31018E-09	0,292457141	0,501	0,38	4,13522E-05	11
Cabp1	4,44752E-09	0,338033996	0,542	0,457	7,96107E-05	11
Sema6d	5,38906E-09	0,277553829	0,71	0,674	9,64642E-05	11
Slit3	7,75431E-09	0,303123254	0,48	0,371	0,000138802	11
Gpc6	4,03825E-08	0,254968329	0,582	0,489	0,000722847	11
5330434G04Rik	6,4941E-08	0,250853802	0,807	0,781	0,001162444	11
Sel1l3	9,81881E-07	0,268327141	0,63	0,591	0,017575663	11
Map1a	1,29963E-06	0,259162761	0,89	0,855	0,023263328	11
Med25	1,82917E-05	0,250601761	0,528	0,484	0,327421034	11
Pcdh10	0	3,054698747	0,996	0,185	0	12
Cbln2	0	2,619760745	0,965	0,132	0	12
Adgrg6	0	2,46229244	0,947	0,075	0	12
Nog	0	1,706372159	0,845	0,094	0	12
Cyp26b1	0	1,496360379	0,643	0,033	0	12
Syt15	0	1,453565601	0,77	0,038	0	12
Tmeff2	0	1,35512578	0,668	0,04	0	12
Slc35d3	0	1,344139553	0,696	0,027	0	12
Cdkn1c	0	1,324114393	0,686	0,055	0	12

Otof	0	1,314672288	0,657	0,023	0	12
Sgcz	0	0,728372004	0,47	0,021	0	12
Ptger3	0	0,700057598	0,459	0,019	0	12
Islr2	1,7193E-276	1,341842623	0,76	0,097	3,0775E-272	12
Dgkg	5,5097E-253	1,426167628	0,883	0,154	9,8623E-249	12
Edn1	2,6999E-249	1,934865708	0,954	0,202	4,8329E-245	12
Pde2a	1,8564E-247	1,928581424	0,954	0,22	3,323E-243	12
Avil	5,2091E-239	1,378810464	0,728	0,104	9,3243E-235	12
Pkp1	1,7258E-229	1,260848512	0,65	0,084	3,0892E-225	12
Serpine2	1,7111E-228	3,035361616	0,986	0,31	3,0628E-224	12
Phgdh	7,912E-228	1,174792278	0,64	0,081	1,4162E-223	12
Slc25a48	3,1224E-221	0,894757389	0,449	0,038	5,589E-217	12
Tbx2	8,6234E-215	1,962694215	0,958	0,285	1,5436E-210	12
Kctd12	6,592E-203	0,848589208	0,551	0,061	1,18E-198	12
Thsd7b	4,9901E-199	0,513784268	0,325	0,02	8,9322E-195	12
Bcl11a	2,5955E-177	0,742726239	0,505	0,061	4,646E-173	12
Rgs6	5,8497E-174	0,45738488	0,343	0,027	1,0471E-169	12
Gpr85	2,0312E-172	1,728169101	0,936	0,316	3,6358E-168	12
C130060K24Rik	8,5524E-169	0,537501176	0,339	0,027	1,5309E-164	12
Zfp804a	1,4725E-168	1,548797461	0,852	0,236	2,6358E-164	12
Nmu	5,3868E-168	0,936394913	0,3	0,021	9,6424E-164	12
Dapk2	6,418E-165	0,825715426	0,576	0,088	1,1488E-160	12
Esr1	7,0954E-165	0,44204412	0,297	0,021	1,2701E-160	12
Ptgfr	1,2479E-164	1,17745731	0,739	0,155	2,2338E-160	12
Htr3b	8,9852E-163	0,797228982	0,505	0,067	1,6083E-158	12
Adgrg6	0	2,945248746	0,973	0,08	0	13
Cbln2	0	2,840960727	0,977	0,138	0	13
Otof	0	1,430941764	0,665	0,027	0	13
Tmeff2	0	1,287617315	0,665	0,044	0	13
Syt15	0	1,285193935	0,683	0,045	0	13
Slc35d3	0	1,165833693	0,652	0,033	0	13
Sgcz	0	0,869717166	0,493	0,024	0	13
Nog	5,3436E-290	1,798199868	0,851	0,099	9,565E-286	13
Ptger3	1,0648E-283	0,787058152	0,452	0,022	1,9061E-279	13
Cdkn1c	4,1149E-260	1,44170974	0,661	0,06	7,3657E-256	13
Pcdh10	6,8904E-249	2,846780955	0,982	0,191	1,2334E-244	13
Dgkg	8,8123E-238	2,141589666	0,905	0,159	1,5774E-233	13
Bcl11a	7,0671E-235	1,100278701	0,633	0,061	1,265E-230	13
Nmu	5,5519E-226	1,587265647	0,385	0,021	9,9379E-222	13
Cyp26b1	1,3167E-216	1,274759699	0,507	0,04	2,3569E-212	13
Edn1	4,9671E-196	2,03249249	0,937	0,207	8,891E-192	13
Kctd12	7,5559E-193	1,093841475	0,593	0,064	1,3525E-188	13
Pde2a	5,8102E-189	2,017211912	0,928	0,225	1,04E-184	13
Islr2	1,2232E-187	1,350185951	0,71	0,103	2,1896E-183	13
C130060K24Rik	3,3229E-168	0,586676674	0,38	0,028	5,948E-164	13
Dapk2	7,1142E-162	1,023276485	0,629	0,09	1,2734E-157	13

Rgs6	2,5694E-160	0,658268125	0,367	0,028	4,5992E-156	13
Thsd7b	5,6518E-160	0,517494427	0,33	0,022	1,0117E-155	13
Serpine2	1,8258E-153	2,640376199	0,946	0,315	3,2682E-149	13
Gpr85	4,6034E-146	1,862825478	0,928	0,32	8,24E-142	13
Slc25a48	3,7783E-141	0,708888196	0,412	0,041	6,7631E-137	13
Ccbe1	1,6457E-135	1,28632538	0,733	0,158	2,9458E-131	13
Nrxn3	2,317E-135	1,916496787	0,982	0,429	4,1474E-131	13
Htr3b	1,6177E-134	0,798833071	0,516	0,069	2,8956E-130	13
Cysltr2	2,2278E-132	1,228165054	0,719	0,153	3,9877E-128	13
Tbx2	9,1181E-130	1,577479172	0,887	0,291	1,6321E-125	13
Sulf2	1,7101E-128	1,530251326	0,864	0,258	3,0611E-124	13
Cnr1	5,2974E-127	1,670246099	1	0,981	9,4824E-123	13
Ntrk3	1,7885E-126	2,049666408	0,995	0,694	3,2014E-122	13
Efr3a	5,6795E-126	2,136292747	0,932	0,436	1,0166E-121	13
6330403A02Rik	5,6013E-124	2,105112465	1	0,922	1,0026E-119	13
Pkp1	6,54E-122	1,019209574	0,557	0,09	1,1707E-117	13
Snhg11	1,2709E-120	1,660833683	1	0,983	2,2749E-116	13
Id4	3,7105E-119	1,295104642	0,683	0,151	6,6418E-115	13
Casz1	2,625E-118	1,694301037	0,937	0,375	4,6987E-114	13
Rims1	5,7412E-117	0,899727466	0,602	0,111	1,0277E-112	13
Zfp804a	2,8878E-112	1,52648522	0,796	0,242	5,1692E-108	13
9530059O14Rik	1,7022E-110	1,826871899	0,995	0,732	3,047E-106	13
Zeb2	4,3135E-110	1,753923619	0,991	0,645	7,7211E-106	13
Hcn1	2,0132E-109	0,596701991	0,394	0,048	3,6036E-105	13
Tcf7l2	3,0406E-108	1,515646583	1	0,841	5,4426E-104	13
Lhfpl2	2,9213E-105	1,593499768	0,955	0,486	5,2291E-101	13
mt-Nd2	4,9557E-103	1,141275137	1	1	8,8708E-99	13
Pcdh9	1,8257E-102	1,55060626	0,774	0,257	3,268E-98	13
Fam19a1	1,0051E-101	1,456727002	0,928	0,428	1,79905E-97	13
Rab3c	2,0944E-101	1,458003657	1	0,986	3,74896E-97	13
mt-Atp6	1,5554E-100	0,895688625	1	1	2,78415E-96	13
Phox2b	2,3308E-97	1,308761826	1	0,981	4,17213E-93	13
Cpne4	5,60564E-97	1,345919979	0,995	0,793	1,00341E-92	13
Hey1	9,95714E-97	0,910588066	0,548	0,111	1,78233E-92	13
mt-Atp8	2,20943E-94	1,242654277	1	1	3,95488E-90	13
mt-Nd4l	1,23539E-92	1,150904536	1	1	2,21134E-88	13
Ptgfr	3,48611E-92	1,053801188	0,647	0,161	6,24013E-88	13
Tubb3	8,27569E-92	1,215048766	1	0,988	1,48135E-87	13
A330102I10Rik	4,50012E-91	0,450364206	0,321	0,038	8,05521E-87	13
Wif1	2,85955E-89	0,956819923	0,566	0,125	5,1186E-85	13
Hoxb5	2,65199E-88	1,30024316	1	0,945	4,74706E-84	13
Chst15	1,65723E-87	1,070241065	0,706	0,204	2,96644E-83	13
mt-Nd4	3,57105E-87	0,83794059	1	1	6,39217E-83	13
Gse1	9,77914E-86	1,270295964	0,995	0,816	1,75047E-81	13
Gucy1a3	2,71162E-85	1,365884488	0,905	0,498	4,8538E-81	13
Cntn5	1,63403E-83	1,2656608	0,71	0,235	2,92492E-79	13

Cacna1a	1,38848E-82	1,343226421	0,914	0,579	2,48538E-78	13
mt-Cytb	8,0594E-82	0,692994772	1	1	1,44263E-77	13
Calb2	2,0017E-81	1,263686009	1	0,969	3,58305E-77	13
Psd3	4,05701E-81	1,260128742	0,729	0,25	7,26205E-77	13
Trp53i11	2,65668E-80	1,411972521	1	0,669	4,75546E-76	13
Ank2	3,08695E-80	0,982910747	1	0,984	5,52564E-76	13
Slc35g1	3,30757E-79	0,839658249	0,579	0,145	5,92056E-75	13
Pdzd2	7,11429E-78	0,824878687	0,557	0,131	1,27346E-73	13
Kcnq1ot1	2,23259E-77	1,567487133	0,982	0,841	3,99633E-73	13
Proser2	7,62858E-77	0,835876624	0,538	0,128	1,36552E-72	13
Ngfr	1,55639E-76	1,356647245	0,955	0,544	2,78593E-72	13
Ptprd	2,19191E-76	1,065654567	0,991	0,86	3,92352E-72	13
Timp3	1,80691E-74	1,424864287	0,932	0,481	3,23436E-70	13
Syt2	6,03164E-74	1,182380194	0,914	0,637	1,07966E-69	13
Ccser2	1,28389E-73	1,155257802	0,986	0,806	2,29816E-69	13
Bche	4,61358E-73	1,317083693	0,991	0,774	8,2583E-69	13
Col5a3	5,49355E-73	0,571187324	0,43	0,08	9,83345E-69	13
mt-Nd1	1,33772E-72	0,837631761	1	1	2,39451E-68	13
Tcerg1l	1,62485E-72	1,028466479	0,643	0,199	2,90849E-68	13
Myl1	2,37921E-72	1,101897315	1	0,911	4,25878E-68	13
Hpcal1	2,28303E-71	1,216019371	0,701	0,27	4,08663E-67	13
Gm10600	5,73763E-71	0,500452954	0,353	0,058	1,02704E-66	13
Nedd4l	7,35842E-71	1,140262015	0,837	0,439	1,31716E-66	13
Cacna1e	8,06248E-70	1,218030621	0,86	0,478	1,44318E-65	13
Grin3a	1,07061E-69	1,20396526	0,751	0,31	1,91639E-65	13
Bmp4	3,73729E-69	0,858641428	0,538	0,138	6,68975E-65	13
Tubb5	9,16943E-69	0,84184048	1	0,994	1,64133E-64	13
Sgcd	1,24364E-68	0,765248626	0,552	0,145	2,22612E-64	13
Smad6	1,81447E-68	0,810635486	0,593	0,17	3,2479E-64	13
mt-Nd5	5,55174E-68	0,964985511	1	0,999	9,93762E-64	13
Apba1	3,25194E-65	1,02767788	0,968	0,723	5,82098E-61	13
Adcy1	2,19123E-64	1,063582287	0,751	0,311	3,9223E-60	13
Fmnl2	6,61147E-64	1,057010124	0,824	0,422	1,18345E-59	13
Scn3a	9,92742E-64	1,012079526	0,982	0,816	1,77701E-59	13
Gal	2,5467E-245	5,769695011	1	0,209	4,5587E-241	14
Rprml	3,3262E-177	1,537683844	0,834	0,151	5,9538E-173	14
Adcyap1	2,5533E-167	0,784104618	0,36	0,025	4,5704E-163	14
Ntng1	3,0385E-143	1,119834906	0,431	0,044	5,4389E-139	14
Tmc3	2,4379E-128	1,141034994	0,521	0,074	4,3638E-124	14
Ebf1	1,5657E-113	0,978510177	0,431	0,054	2,8026E-109	14
Slc18a2	4,1126E-102	1,263200524	0,844	0,243	7,36154E-98	14
Tm4sf4	1,35961E-92	1,263968123	0,934	0,349	2,4337E-88	14
Ngb	9,53643E-77	0,703775681	0,403	0,068	1,70702E-72	14
Agrp	1,68551E-75	0,77678052	0,393	0,067	3,01706E-71	14
Nos1	2,41737E-67	2,524404917	0,256	0,032	4,3271E-63	14
Gch1	1,29979E-64	0,608177786	0,498	0,114	2,32663E-60	14

Ndst4	4,90482E-64	0,892961839	0,858	0,325	8,77962E-60	14
Edil3	2,2143E-60	0,714064462	0,521	0,135	3,96361E-56	14
Luzp2	1,63734E-59	0,387057043	0,265	0,038	2,93083E-55	14
Ltk	6,6091E-59	0,52608114	0,313	0,053	1,18303E-54	14
Tesc	1,17499E-58	0,570116926	0,389	0,079	2,10324E-54	14
Ccdc109b	1,41243E-58	0,55328246	0,531	0,137	2,52826E-54	14
Gm13889	1,42306E-57	0,900375849	0,882	0,381	2,54728E-53	14
St18	9,56506E-57	0,452959933	0,289	0,047	1,71215E-52	14
Ckb	1,10119E-56	1,036297267	0,962	0,74	1,97114E-52	14
Resp18	1,87835E-56	1,257993832	0,976	0,926	3,36224E-52	14
Crispld1	6,70877E-55	0,436533218	0,37	0,075	1,20087E-50	14
Ncoa7	1,03837E-54	0,978009255	0,986	0,781	1,85868E-50	14
Abcb1a	1,66078E-51	0,529878925	0,488	0,131	2,97279E-47	14
Rgs10	3,69159E-51	0,630722415	0,73	0,273	6,60795E-47	14
Cox7c	5,91862E-51	0,885105248	0,995	0,958	1,05943E-46	14
Klc3	3,43823E-50	0,398182228	0,322	0,063	6,15443E-46	14
Etv1	4,82584E-50	1,07530369	1	0,604	8,63826E-46	14
Tfcp2l1	1,04778E-49	0,35549538	0,251	0,04	1,87553E-45	14
Rtn4rl1	3,26555E-49	0,599982065	0,521	0,148	5,84534E-45	14
Gapdh	4,10978E-49	0,93223856	0,995	0,93	7,35651E-45	14
Alcam	4,61663E-49	1,158512254	0,981	0,616	8,26377E-45	14
Slitrk4	5,6394E-49	0,423635308	0,308	0,059	1,00945E-44	14
Sh3bgrl3	1,71546E-47	0,900595599	0,929	0,634	3,07067E-43	14
Nsg1	1,14428E-46	0,775280957	1	0,972	2,04825E-42	14
Scg5	3,09535E-46	0,842549146	0,986	0,834	5,54068E-42	14
Ptpre	4,80407E-46	0,579370501	0,659	0,237	8,59928E-42	14
Calm2	2,19298E-45	0,596754697	1	1	3,92544E-41	14
Cst3	4,03376E-45	0,797770084	0,967	0,933	7,22043E-41	14
Sec11c	4,94958E-45	0,634292922	0,635	0,237	8,85975E-41	14
Kcnd2	6,33341E-45	0,715664856	0,848	0,376	1,13368E-40	14
Fxyd5	1,39039E-44	0,869492992	0,844	0,422	2,4888E-40	14
Bex2	5,18792E-44	0,624514134	0,995	0,992	9,28638E-40	14
Chchd10	7,78916E-44	0,839229492	0,716	0,304	1,39426E-39	14
Vip	9,58765E-44	1,182428658	1	0,925	1,71619E-39	14
Ndufa1	1,62015E-43	0,826973079	0,934	0,604	2,90007E-39	14
Synpo2	2,06841E-43	0,509204252	0,559	0,181	3,70245E-39	14
Fam89a	1,24693E-42	0,614163045	0,63	0,241	2,232E-38	14
Tmem150c	1,62407E-42	0,388385838	0,341	0,079	2,90708E-38	14
Prdx4	6,52795E-42	0,557993352	0,63	0,234	1,1685E-37	14
Acot7	6,62017E-42	0,754974256	0,953	0,729	1,18501E-37	14
Tceal6	2,278E-41	0,666313145	0,645	0,252	4,07762E-37	14
Moxd1	4,13621E-41	0,865302203	0,882	0,481	7,40382E-37	14
Ndufa4	4,16425E-41	0,762255021	0,981	0,843	7,45401E-37	14
Pcolce	8,86638E-41	0,526521882	0,64	0,238	1,58708E-36	14
Tspan13	2,03216E-40	0,737583897	0,962	0,745	3,63756E-36	14
Ppa1	2,03949E-40	0,832634903	0,877	0,584	3,65069E-36	14

Hotairm1	3,49517E-40	0,66861577	0,806	0,412	6,25635E-36	14
Nxn	4,22564E-40	0,54524609	0,474	0,151	7,56389E-36	14
Thy1	5,67698E-40	0,760318064	0,934	0,521	1,01618E-35	14
Gpx3	2,46728E-39	0,537940673	0,739	0,324	4,41643E-35	14
Enpp1	7,4327E-39	0,569228151	0,73	0,306	1,33045E-34	14
Stmn3	1,06376E-38	0,753383436	0,986	0,915	1,90414E-34	14
S100a4	1,35199E-38	1,031957803	0,905	0,745	2,42006E-34	14
Tmem108	3,21848E-38	0,549808657	0,687	0,282	5,76107E-34	14
Cdh11	8,17121E-38	0,522085783	0,536	0,188	1,46265E-33	14
Fth1	1,22586E-37	0,621332622	1	0,994	2,19428E-33	14
Crip1	4,44477E-37	0,822069154	0,995	0,986	7,95614E-33	14
Kitl	6,61121E-37	0,412922791	0,256	0,053	1,18341E-32	14
Slc25a4	8,20137E-37	0,640089059	0,995	0,969	1,46804E-32	14
Gfra1	3,3894E-36	0,621584689	0,934	0,461	6,06702E-32	14
Eef1e1	6,14542E-36	0,665950005	0,9	0,525	1,10003E-31	14
Mpc1	1,92286E-35	0,747992936	0,976	0,824	3,44193E-31	14
Tubb4b	3,01368E-35	0,735991175	0,948	0,788	5,39448E-31	14
Cntnap5a	6,38572E-35	0,686829816	0,972	0,657	1,14304E-30	14
Gna14	1,24226E-34	0,375211574	0,36	0,098	2,22365E-30	14
Clmp	3,55741E-34	0,413039461	0,408	0,125	6,36776E-30	14
Dstn	6,41542E-34	0,67466101	0,995	0,95	1,14836E-29	14
Tmem130	1,35616E-33	0,634677636	0,896	0,548	2,42753E-29	14
Hspa2	1,67083E-33	0,553996399	0,592	0,251	2,99078E-29	14
Cox6c	2,31342E-33	0,727959635	0,981	0,876	4,14102E-29	14
Spock1	7,26226E-33	0,441230747	0,659	0,268	1,29994E-28	14
Chchd2	9,52933E-33	0,651221402	0,986	0,92	1,70575E-28	14
Kcnd3	1,03366E-32	0,362815886	0,289	0,072	1,85025E-28	14
Stmn2	1,98229E-32	0,449771913	1	0,99	3,5483E-28	14
Rpl22l1	2,66927E-32	0,642172804	0,957	0,786	4,778E-28	14
Sec61g	2,92068E-32	0,668604955	0,967	0,875	5,22802E-28	14
Atp5f1	3,25991E-32	0,653790824	0,919	0,688	5,83524E-28	14
Ngfrap1	3,45676E-32	0,571328063	0,986	0,94	6,18761E-28	14
Cox5a	4,1343E-32	0,666670777	0,91	0,65	7,4004E-28	14
A730017C20Rik	4,78749E-32	0,676312547	0,938	0,688	8,5696E-28	14
Tmem256	7,50938E-32	0,666254719	0,863	0,54	1,34418E-27	14
Myeov2	1,05306E-31	0,611150304	0,877	0,525	1,88498E-27	14
Rexo2	1,16826E-31	0,6166618	0,905	0,612	2,09118E-27	14
Rps21	1,82328E-31	0,6990661	0,991	0,951	3,26367E-27	14
Ywhaq	2,24864E-31	0,566361365	0,995	0,945	4,02507E-27	14
S100a6	2,54868E-31	0,642286488	0,995	0,994	4,56214E-27	14
Gabrg2	3,47871E-31	0,430162424	0,441	0,152	6,22689E-27	14
Oaz1	4,00235E-31	0,553964938	0,995	0,929	7,16421E-27	14
Rps8	4,03414E-31	0,528938814	0,995	0,977	7,22111E-27	14
Paip2b	2,6587E-152	4,320302017	1	0,497	4,759E-148	15
Vip	2,06174E-51	1,278051995	1	0,925	3,69052E-47	15
Scgn	8,37683E-51	1,160590493	1	0,856	1,49945E-46	15

Gfra1	3,40827E-47	0,84915348	0,928	0,462	6,1008E-43	15
Cd24a	1,90154E-46	0,916673991	1	0,63	3,40376E-42	15
Npy	5,49073E-46	1,088943983	1	0,845	9,8284E-42	15
Moxd1	2,9982E-44	0,887401288	0,928	0,48	5,36677E-40	15
Etv1	1,00804E-42	0,927503491	1	0,604	1,8044E-38	15
Fxyd5	3,61737E-41	0,806169941	0,852	0,422	6,47509E-37	15
Thy1	6,11528E-41	0,810347175	0,933	0,521	1,09464E-36	15
Nsg1	3,30804E-40	0,678386192	1	0,972	5,92139E-36	15
Alcam	9,56443E-40	0,831096649	0,967	0,617	1,71203E-35	15
Cntnap5a	3,82204E-38	0,751256252	0,986	0,657	6,84145E-34	15
Spock3	1,81856E-37	0,753141064	0,938	0,589	3,25523E-33	15
Dbh	1,90546E-37	1,07194081	0,943	0,654	3,41078E-33	15
Kcnd2	1,95217E-37	0,652411253	0,813	0,377	3,49438E-33	15
Igfbp7	6,61324E-36	0,464825085	0,847	0,4	1,18377E-31	15
Csrp1	1,97633E-35	0,611541325	1	0,953	3,53763E-31	15
Eef1e1	1,99139E-34	0,660049941	0,904	0,525	3,56458E-30	15
Sertm1	3,94924E-34	0,485999996	0,593	0,227	7,06914E-30	15
Prokr1	9,35623E-34	0,425176913	0,574	0,209	1,67476E-29	15
Tgfb1	9,66599E-34	0,566183572	0,742	0,331	1,73021E-29	15
Gm13889	1,28701E-33	0,582338249	0,813	0,382	2,30374E-29	15
Fibcd1	3,14411E-33	0,612456315	0,823	0,385	5,62795E-29	15
Ndst4	1,04454E-32	0,533955708	0,737	0,328	1,86972E-28	15
Thsd7a	1,24846E-32	0,536506215	0,622	0,247	2,23475E-28	15
F2r	1,26639E-32	0,67002793	0,923	0,56	2,26683E-28	15
Lamc3	2,04068E-30	0,516753679	0,641	0,274	3,65281E-26	15
Ascl1	3,97592E-29	0,42809255	0,603	0,246	7,11689E-25	15
Ntsr1	4,4593E-29	0,513520275	0,684	0,315	7,98214E-25	15
Ngfr	5,72413E-29	0,591039979	0,943	0,545	1,02462E-24	15
Tspan13	2,87309E-28	0,623501346	0,952	0,745	5,14283E-24	15
Th	7,84251E-28	0,843054044	0,641	0,296	1,40381E-23	15
Hpca	9,283E-28	0,540415822	0,722	0,348	1,66166E-23	15
Tmem130	4,33675E-27	0,583445154	0,861	0,549	7,76278E-23	15
Id3	4,93417E-27	0,601039055	0,828	0,465	8,83216E-23	15
Tmem108	5,15502E-27	0,441629343	0,636	0,283	9,22749E-23	15
Basp1	7,66667E-27	0,487894943	1	0,983	1,37233E-22	15
Camk4	2,1109E-26	0,63891756	0,852	0,569	3,7785E-22	15
A730017C20Rik	7,54851E-26	0,621540437	0,928	0,688	1,35118E-21	15
Kcnj3	2,44112E-25	0,438559363	0,589	0,259	4,36961E-21	15
Gpr149	2,48619E-25	0,48924691	0,703	0,344	4,45028E-21	15
Rimbp2	3,95838E-25	0,43281839	0,632	0,291	7,08551E-21	15
Socs2	8,47226E-25	0,550474601	0,689	0,362	1,51654E-20	15
Enpp1	2,92274E-24	0,439133497	0,651	0,308	5,23171E-20	15
Chl1	1,89418E-23	0,514893161	0,967	0,756	3,39057E-19	15
Ptpre	6,04463E-23	0,42115928	0,536	0,24	1,08199E-18	15
Bglap	6,6579E-23	0,519348659	0,493	0,216	1,19176E-18	15
Gm28905	1,06863E-22	0,331846664	0,388	0,144	1,91285E-18	15

S100a4	1,49373E-22	0,730375649	0,923	0,744	2,67377E-18	15
F2rl2	2,54679E-22	0,343616706	0,469	0,192	4,55875E-18	15
Nrsn2	2,65367E-22	0,368729334	0,517	0,219	4,75007E-18	15
Phlda3	3,05801E-22	0,592912166	0,866	0,639	5,47383E-18	15
Kcnt2	3,84962E-22	0,434489744	0,646	0,313	6,89082E-18	15
Wls	4,09264E-22	0,458456903	0,708	0,382	7,32583E-18	15
Entpd3	4,57716E-22	0,405045274	0,742	0,387	8,19312E-18	15
1500009L16Rik	5,05934E-22	0,531760881	0,78	0,486	9,05622E-18	15
Kif5c	6,56901E-22	0,428614787	1	0,935	1,17585E-17	15
Cd9	6,69793E-22	0,607294127	1	0,985	1,19893E-17	15
Tspan12	8,65873E-22	0,367826974	0,502	0,217	1,54991E-17	15
Cidea	1,08183E-21	0,547036777	0,933	0,753	1,93648E-17	15
Cpne5	2,69546E-21	0,399562383	0,493	0,222	4,82488E-17	15
Isoc1	2,97407E-21	0,45291917	0,694	0,379	5,32358E-17	15
Pcolce	6,4457E-21	0,401235029	0,522	0,241	1,15378E-16	15
Fbn1	1,06353E-20	0,354762253	0,622	0,302	1,90371E-16	15
Cpm	1,08526E-20	0,399896473	0,411	0,169	1,94261E-16	15
Insm1	2,66324E-20	0,408275075	0,541	0,255	4,76721E-16	15
Spock1	2,87181E-20	0,469446117	0,555	0,271	5,14053E-16	15
Sv2b	4,04368E-20	0,357819229	0,517	0,234	7,23818E-16	15
Tmod1	4,80793E-20	0,542948504	0,876	0,615	8,6062E-16	15
Gria3	7,35873E-20	0,423263045	0,56	0,269	1,31721E-15	15
Clec14a	8,29126E-20	0,378086916	0,55	0,262	1,48414E-15	15
Nrp1	9,49626E-20	0,477222157	0,938	0,767	1,69983E-15	15
Nfe2l2	1,53969E-19	0,363163083	0,593	0,292	2,75604E-15	15
Calm2	1,88E-19	0,385201102	1	1	3,36521E-15	15
Smpd3	2,16785E-19	0,408670609	1	0,992	3,88045E-15	15
Resp18	2,30942E-19	0,635845661	0,986	0,926	4,13385E-15	15
Bex2	3,6497E-19	0,39270499	1	0,992	6,53296E-15	15
Phactr1	3,85251E-19	0,481379447	0,89	0,686	6,89598E-15	15
Chst8	6,77964E-19	0,32455584	0,44	0,193	1,21356E-14	15
Auts2	8,23979E-19	0,42749262	0,904	0,678	1,47492E-14	15
Ptprz1	1,575E-18	0,415572599	0,598	0,301	2,81924E-14	15
Kcnq5	2,06127E-18	0,300694271	0,416	0,176	3,68967E-14	15
Ncoa7	2,27797E-18	0,537563123	0,914	0,783	4,07757E-14	15
Dach1	3,02317E-18	0,286571154	0,397	0,164	5,41148E-14	15
A830010M20Rik	3,98422E-18	0,397714227	0,684	0,394	7,13176E-14	15
Adcyap1r1	9,24853E-18	0,389304072	0,703	0,391	1,65549E-13	15
Slco3a1	9,59959E-18	0,343464097	0,565	0,29	1,71833E-13	15
Vcan	1,04395E-17	0,384351946	0,44	0,199	1,86868E-13	15
Lnx1	1,77171E-17	0,319875072	0,44	0,201	3,17137E-13	15
Mpc1	2,56003E-17	0,516372965	0,943	0,825	4,58245E-13	15
Tceal6	2,75631E-17	0,391813004	0,507	0,256	4,9338E-13	15
Gnas	2,88938E-17	0,296882851	1	1	5,17198E-13	15
B3glct	3,66599E-17	0,379580682	0,555	0,284	6,56213E-13	15
Cst3	3,82552E-17	0,396973824	0,986	0,933	6,84768E-13	15

Myo1b	4,4134E-17	0,334042019	0,541	0,271	7,89998E-13	15
Rit2	1,33739E-16	0,414503727	0,828	0,559	2,39393E-12	15
Rassf5	1,34192E-16	0,303526354	0,459	0,217	2,40203E-12	15
Atp7a	2,95074E-16	0,348488288	0,459	0,223	5,28182E-12	15
Npr1	3,62297E-16	0,389959386	0,612	0,33	6,48511E-12	15
Pcsk1	3,9971E-16	0,433470073	0,852	0,641	7,1548E-12	15
Apoe	0	4,411004243	1	0,057	0	16
Plp1	0	2,215798436	0,974	0,03	0	16
Fabp7	0	2,120444708	0,967	0,063	0	16
Rarres2	0	1,805174948	0,901	0,02	0	16
Tgfb2	0	1,748445692	0,94	0,072	0	16
Lpar1	0	1,741600279	0,927	0,029	0	16
Sostdc1	0	1,595622462	0,821	0,01	0	16
Abca8a	0	1,485030353	0,868	0,017	0	16
Postn	0	1,448779209	0,768	0,021	0	16
Atp1a2	0	1,385623141	0,848	0,02	0	16
Entpd2	0	1,372434892	0,868	0,02	0	16
Col12a1	0	1,314887358	0,762	0,029	0	16
Cdh19	0	1,287184457	0,788	0,011	0	16
Sox10	0	1,266697248	0,821	0,011	0	16
Col18a1	0	1,215359039	0,748	0,021	0	16
Sfrp1	0	1,151739923	0,682	0,018	0	16
Metrn	0	1,118741143	0,715	0,04	0	16
S1pr3	0	1,103241692	0,623	0,011	0	16
Nid1	0	1,098921277	0,768	0,038	0	16
Mal	0	1,073732097	0,656	0,01	0	16
Slc35f1	0	0,998214706	0,689	0,018	0	16
C4b	0	0,978861193	0,536	0,013	0	16
Kcna1	0	0,961410932	0,57	0,008	0	16
Foxd3	0	0,919606272	0,57	0,007	0	16
Art3	0	0,901992643	0,596	0,017	0	16
Fbln5	0	0,890481554	0,589	0,018	0	16
Hspg2	0	0,866604259	0,583	0,02	0	16
Olfml2a	0	0,865237142	0,636	0,011	0	16
Slc43a3	0	0,864364196	0,589	0,016	0	16
Pdlim4	0	0,858698893	0,583	0,023	0	16
Nkain4	0	0,82145168	0,517	0,014	0	16
Pla2g16	0	0,793960715	0,536	0,019	0	16
Cmtm5	0	0,777435528	0,517	0,009	0	16
Col20a1	0	0,76300864	0,424	0,007	0	16
Col5a1	0	0,710251184	0,543	0,019	0	16
Gjc3	0	0,701725528	0,477	0,009	0	16
Col28a1	0	0,679143203	0,338	0,005	0	16
Myl9	0	0,636557805	0,503	0,015	0	16
Paqr6	0	0,604403565	0,411	0,007	0	16
Tmprss5	0	0,594821463	0,371	0,006	0	16

Sfrp5	0	0,590113965	0,384	0,008	0	16
Mmd2	0	0,579465048	0,377	0,006	0	16
Gfap	0	0,573733541	0,331	0,005	0	16
Kcnj10	0	0,47602102	0,325	0,006	0	16
Wnt6	0	0,405270566	0,272	0,003	0	16
Lamb1	1,0275E-302	0,646815752	0,51	0,018	1,8391E-298	16
Gm2115	1,7487E-298	0,431248536	0,291	0,004	3,1301E-294	16
Gm12688	1,0866E-292	0,367243596	0,278	0,004	1,945E-288	16
Col5a2	3,1553E-284	0,68309471	0,563	0,025	5,648E-280	16
Lgi4	1,9299E-279	1,434972595	0,815	0,063	3,4546E-275	16
Vcam1	2,9735E-278	0,565975052	0,318	0,007	5,3226E-274	16
Heyl	4,1232E-262	0,505536559	0,351	0,009	7,3805E-258	16
Col11a1	3,3207E-261	0,530205765	0,338	0,008	5,9441E-257	16
Gpr37l1	6,1751E-257	1,283561659	0,881	0,08	1,1053E-252	16
Col16a1	1,3976E-250	0,871861581	0,576	0,032	2,5017E-246	16
Pmp22	1,2932E-244	1,164648148	0,854	0,077	2,3148E-240	16
Fign	2,0038E-235	0,3451439	0,252	0,005	3,5868E-231	16
Fxyd1	1,217E-234	2,019407446	0,993	0,13	2,1785E-230	16
Axl	1,0198E-233	0,478013768	0,338	0,01	1,8254E-229	16
Slitrk6	2,1748E-231	0,665647782	0,417	0,017	3,8928E-227	16
Col8a1	1,5762E-229	0,555243026	0,331	0,01	2,8215E-225	16
Mmp17	3,9618E-228	0,411910951	0,305	0,008	7,0916E-224	16
Itih5	2,1759E-226	1,238182708	0,755	0,064	3,8949E-222	16
Aspa	1,95E-215	0,4507923	0,298	0,008	3,4905E-211	16
Lmo4	9,7115E-213	0,98523824	0,715	0,06	1,7384E-208	16
Gpm6b	2,6568E-209	1,341856437	0,868	0,099	4,7557E-205	16
Kirrel	7,1846E-207	0,422096285	0,298	0,009	1,286E-202	16
Ltbp1	7,9018E-203	0,582825973	0,391	0,017	1,4144E-198	16
Sorbs2	2,8822E-194	1,081497429	0,742	0,074	5,1591E-190	16
Capg	5,244E-193	0,469320715	0,291	0,009	9,3867E-189	16
Igfbp4	1,1218E-187	0,792833911	0,556	0,039	2,0079E-183	16
Sdc4	8,7228E-185	0,845980023	0,642	0,055	1,5614E-180	16
Gpx8	9,5413E-185	0,406128599	0,291	0,01	1,7079E-180	16
Gatm	6,8408E-181	0,959975351	0,675	0,065	1,2245E-176	16
Hmgcs2	2,124E-178	0,646549327	0,464	0,029	3,802E-174	16
Sash1	1,7935E-177	0,825078345	0,636	0,058	3,2103E-173	16
Grik3	7,4354E-175	0,491451031	0,338	0,015	1,3309E-170	16
Acot1	2,082E-173	0,381766363	0,325	0,014	3,7268E-169	16
Pdgfb	4,4083E-173	0,536726629	0,404	0,022	7,8908E-169	16
Ppic	1,5591E-168	0,419284922	0,331	0,015	2,7907E-164	16
Car12	1,4091E-159	0,661635106	0,411	0,025	2,5223E-155	16
Lims2	2,8401E-158	0,427748869	0,278	0,011	5,0838E-154	16
Gpsm2	3,2423E-156	0,465815685	0,331	0,016	5,8037E-152	16
Lhfp	1,3828E-155	0,37662252	0,285	0,012	2,4753E-151	16
Ctnnal1	3,9773E-154	1,050905831	0,728	0,09	7,1194E-150	16
Cdk6	2,7563E-153	0,397951236	0,258	0,009	4,9339E-149	16

Plekhb1	1,4667E-152	1,240741711	0,874	0,14	2,6254E-148	16
Ptrf	3,0645E-152	0,647563705	0,477	0,036	5,4854E-148	16
Shc4	2,6971E-151	0,578048339	0,397	0,025	4,8279E-147	16
Adam11	2,134E-148	0,385560335	0,265	0,01	3,8198E-144	16
Olfml3	6,8245E-148	0,975010135	0,755	0,1	1,2216E-143	16
Rbp1	9,4416E-148	0,940143743	0,669	0,078	1,69E-143	16
Fam129a	1,6191E-143	0,663786344	0,523	0,047	2,8981E-139	16
Kcna2	6,8932E-141	1,169829046	0,788	0,121	1,2339E-136	16
Cyba	4,5823E-139	0,47494466	0,377	0,024	8,2024E-135	16
Notch1	1,6428E-138	0,457417449	0,344	0,02	2,9406E-134	16
Zfp36l1	1,3065E-134	0,485140574	0,404	0,029	2,3386E-130	16
Arpc1b	1,7898E-134	1,687354986	0,954	0,218	3,2038E-130	16
Col15a1	6,1116E-132	0,39140589	0,258	0,011	1,094E-127	16
Tspan15	9,9337E-130	0,473967849	0,391	0,028	1,7781E-125	16
Zcchc24	6,242E-128	0,346908606	0,252	0,011	1,1173E-123	16
Neurod1	0	2,433407299	0,721	0,015	0	17
Cldn7	0	2,082577626	0,836	0,03	0	17
Gm609	0	1,677936964	0,615	0,01	0	17
Pax6	0	1,552722598	0,508	0,011	0	17
Krt7	0	1,424676652	0,582	0,019	0	17
Hepacam2	0	1,30286986	0,516	0,014	0	17
Isl1	0	1,248833047	0,303	0,003	0	17
Epcam	4,2088E-295	1,97543371	0,656	0,028	7,5338E-291	17
Lhx1	6,4845E-291	1,964206937	0,426	0,011	1,1607E-286	17
Fxyd3	9,9676E-280	2,175768199	0,541	0,02	1,7842E-275	17
Krt8	3,4462E-254	1,614038979	0,68	0,037	6,1686E-250	17
Rbm47	1,3181E-242	0,989474732	0,467	0,017	2,3595E-238	17
Tph1	6,3764E-232	1,889749701	0,434	0,015	1,1414E-227	17
Ccnd2	3,4969E-223	2,140220261	0,672	0,042	6,2595E-219	17
Adgrg4	1,5908E-219	0,911300369	0,336	0,009	2,8476E-215	17
Slc18a1	1,6389E-216	1,646687477	0,434	0,017	2,9335E-212	17
Prox1	6,8866E-210	0,612735824	0,279	0,006	1,2327E-205	17
Vsig2	1,6534E-208	1,008138445	0,254	0,005	2,9596E-204	17
Phldb2	4,8737E-207	0,824665243	0,402	0,015	8,7239E-203	17
Apob	1,5545E-205	1,075111548	0,418	0,016	2,7826E-201	17
Smim22	1,5833E-202	1,176938568	0,475	0,022	2,8341E-198	17
Klf5	7,0846E-198	0,917904802	0,434	0,018	1,2681E-193	17
C2cd4b	1,0225E-190	0,653593513	0,279	0,007	1,8302E-186	17
Cdh1	4,0557E-189	0,948475857	0,434	0,019	7,2597E-185	17
Foxa1	1,3477E-188	0,600923042	0,328	0,01	2,4123E-184	17
Sis	4,43E-185	1,380855032	0,426	0,019	7,9297E-181	17
Meis2	8,7399E-184	1,38194734	0,287	0,008	1,5644E-179	17
Dsp	4,4918E-183	0,750189576	0,385	0,015	8,0402E-179	17
Hk2	1,6398E-178	1,854942446	0,467	0,025	2,9352E-174	17
Arhgef38	5,8795E-177	0,557378683	0,262	0,007	1,0524E-172	17
Nt5e	3,4199E-175	0,893242748	0,32	0,011	6,1217E-171	17

Ambp	1,0631E-172	1,009385857	0,303	0,01	1,903E-168	17
Baiap2l2	1,8402E-169	0,867759172	0,41	0,02	3,294E-165	17
Rfx6	1,3438E-167	0,715056624	0,287	0,009	2,4053E-163	17
Krt20	8,3432E-165	1,129372131	0,385	0,017	1,4934E-160	17
Ddc	4,9765E-160	1,509520789	0,598	0,049	8,908E-156	17
Ms4a8a	1,1062E-159	0,785310527	0,344	0,014	1,98E-155	17
Gipc2	9,2442E-156	0,611812409	0,385	0,019	1,6547E-151	17
Atp2a3	1,673E-151	0,762012564	0,27	0,009	2,9947E-147	17
Krt18	1,7989E-151	1,341784471	0,377	0,019	3,22E-147	17
Itpr3	3,4297E-147	1,049887098	0,41	0,023	6,1391E-143	17
Pitx2	6,9386E-145	0,901249202	0,27	0,009	1,242E-140	17
Tmc4	5,3779E-141	1,035007255	0,557	0,048	9,6265E-137	17
Cdcp1	1,7569E-137	0,517947351	0,27	0,01	3,1448E-133	17
Smim6	5,5028E-137	0,550878343	0,27	0,01	9,8501E-133	17
Cdhr5	2,2268E-136	0,919690078	0,352	0,018	3,9859E-132	17
Nkx2-2	4,329E-135	0,507525282	0,262	0,009	7,749E-131	17
Vil1	1,1647E-132	0,783345207	0,393	0,023	2,0848E-128	17
Prr15l	2,2213E-132	0,676669775	0,361	0,019	3,9762E-128	17
Atp8b1	1,1841E-131	0,691868253	0,385	0,022	2,1195E-127	17
Sct	1,2539E-130	1,605703253	0,5	0,041	2,2445E-126	17
Elf3	4,5586E-129	0,735953888	0,336	0,017	8,1598E-125	17
Sox6	1,2094E-124	0,498826845	0,262	0,011	2,1648E-120	17
Neurl3	3,0353E-123	0,664168398	0,295	0,014	5,4332E-119	17
Plac8	2,037E-122	0,859721943	0,467	0,036	3,6463E-118	17
Gata6	4,1835E-120	0,567993981	0,279	0,013	7,4885E-116	17
Sepp1	6,7147E-116	1,335053202	0,631	0,075	1,2019E-111	17
Tspan1	2,9418E-115	0,612518714	0,328	0,018	5,2658E-111	17
Irf6	3,3705E-114	0,624115971	0,311	0,017	6,0332E-110	17
F11r	1,4732E-113	0,588382157	0,311	0,017	2,637E-109	17
Runx1t1	1,525E-112	0,853466976	0,377	0,026	2,7298E-108	17
Lgals2	3,8564E-112	1,08996692	0,443	0,036	6,903E-108	17
Rreb1	3,8962E-111	0,466937184	0,279	0,014	6,9742E-107	17
Slc38a11	1,0558E-108	1,208468085	0,369	0,026	1,8899E-104	17
Fam83e	1,2675E-106	0,523585017	0,262	0,013	2,2688E-102	17
Myo15b	1,1884E-101	0,600167671	0,287	0,016	2,12723E-97	17
Klf4	2,26016E-96	1,453579795	0,467	0,049	4,04568E-92	17
Gucy2c	5,78094E-94	0,578069752	0,279	0,017	1,03479E-89	17
Degs2	4,04352E-91	0,66590639	0,311	0,022	7,2379E-87	17
Lgals4	1,33489E-90	1,24405869	0,533	0,067	2,38944E-86	17
Dsg2	5,12497E-88	0,722263602	0,328	0,025	9,1737E-84	17
Pigr	7,67468E-88	1,620254257	0,459	0,051	1,37377E-83	17
Muc13	2,79176E-87	1,00383838	0,336	0,026	4,99725E-83	17
Rgs2	1,33412E-86	1,738354594	0,459	0,052	2,38807E-82	17
Foxp4	4,81172E-86	0,566640794	0,311	0,023	8,61298E-82	17
Gpa33	1,77495E-85	0,616606999	0,27	0,017	3,17717E-81	17
Tff3	5,69376E-85	0,956193961	0,32	0,025	1,01918E-80	17

Pde11a	1,35842E-84	0,616478743	0,279	0,019	2,43156E-80	17
Cdh17	3,19459E-81	0,78881592	0,311	0,024	5,71831E-77	17
Sned1	2,14192E-78	0,614236095	0,27	0,019	3,83403E-74	17
Myo10	1,50683E-76	0,71382466	0,303	0,025	2,69722E-72	17
Ghr	2,97595E-70	0,738513647	0,352	0,038	5,32694E-66	17
Hnf4g	1,05838E-68	0,740884546	0,361	0,04	1,89449E-64	17
Mical2	4,40736E-61	1,127327753	0,443	0,07	7,88917E-57	17
Eps8	1,88537E-59	0,517776612	0,279	0,027	3,37481E-55	17
Kctd12	6,07774E-55	1,292533403	0,434	0,071	1,08791E-50	17
Ssfa2	3,39923E-54	0,90626868	0,41	0,066	6,08463E-50	17
Iqgap1	5,4359E-53	1,646859279	0,779	0,294	9,73026E-49	17
Lpp	5,71252E-53	0,96881348	0,402	0,065	1,02254E-48	17
Chgb	5,10672E-51	2,798387354	0,967	0,764	9,14102E-47	17
Cldn3	1,02186E-50	0,505725257	0,262	0,028	1,82913E-46	17
Myo6	1,08347E-49	1,415173816	0,77	0,309	1,93941E-45	17
Fam46a	1,73561E-49	1,880297256	0,73	0,274	3,10675E-45	17
Neat1	3,48047E-49	2,910261643	0,762	0,309	6,23004E-45	17
Lsr	9,51599E-47	0,607044476	0,295	0,038	1,70336E-42	17
Lcorl	1,91092E-46	2,462201429	0,705	0,274	3,42055E-42	17
Reg4	2,10897E-46	1,367570946	0,336	0,051	3,77506E-42	17
Btg2	9,65072E-43	1,672039761	0,664	0,243	1,72748E-38	17
5330417C22Rik	7,14742E-42	1,559038378	0,762	0,375	1,27939E-37	17
Nefm	9,37782E-41	0,911272721	0,254	0,033	1,67863E-36	17
Pon2	1,50359E-38	0,598809975	0,295	0,045	2,69143E-34	17
Apoe	0	5,656009335	1	0,062	0	18
Plp1	0	3,256592763	0,99	0,036	0	18
Col12a1	0	2,631086767	0,897	0,031	0	18
Lpar1	0	2,57267768	0,938	0,034	0	18
Postn	0	2,568079617	0,835	0,025	0	18
Rarres2	0	2,54328298	0,907	0,025	0	18
Entpd2	0	2,413665162	0,876	0,024	0	18
Atp1a2	0	2,397524161	0,897	0,024	0	18
Abca8a	0	2,356866695	0,845	0,022	0	18
Nid1	0	2,268870651	0,866	0,041	0	18
Sostdc1	0	2,255073945	0,763	0,015	0	18
Col18a1	0	2,174734418	0,835	0,024	0	18
Cdh19	0	2,152856524	0,825	0,015	0	18
Sfrp1	0	2,05425744	0,722	0,022	0	18
Sox10	0	1,925428452	0,825	0,015	0	18
S1pr3	0	1,745553818	0,773	0,013	0	18
Hspg2	0	1,686920749	0,711	0,022	0	18
Kcna1	0	1,63757707	0,619	0,01	0	18
Olfml2a	0	1,584032306	0,763	0,013	0	18
Slc35f1	0	1,546121562	0,742	0,021	0	18
Slc43a3	0	1,521537947	0,66	0,018	0	18
C4b	0	1,494548264	0,588	0,015	0	18

Mal	0	1,391094874	0,608	0,014	0	18
Foxd3	0	1,382010624	0,588	0,01	0	18
Gjc3	0	1,318610302	0,68	0,009	0	18
Col11a1	0	1,229278839	0,526	0,008	0	18
Cmtm5	0	1,212897161	0,66	0,01	0	18
Col20a1	0	1,14246507	0,505	0,009	0	18
Tmprss5	0	1,107920442	0,515	0,007	0	18
Gfap	0	0,886818931	0,423	0,006	0	18
Mmd2	0	0,828096105	0,464	0,008	0	18
Kcnj10	0	0,789219316	0,402	0,007	0	18
Gm2115	0	0,657559553	0,371	0,005	0	18
Col3a1	9,1255E-301	1,071245498	0,732	0,029	1,6335E-296	18
Col28a1	1,5118E-296	0,827489803	0,381	0,006	2,7061E-292	18
Col9a2	2,2473E-295	1,04594372	0,392	0,007	4,0227E-291	18
Fbln5	3,6997E-289	1,415514027	0,619	0,021	6,6225E-285	18
Col8a1	6,9365E-285	1,188962258	0,454	0,01	1,2416E-280	18
Nkain4	5,2618E-273	1,175476825	0,536	0,016	9,4187E-269	18
Megf10	1,7248E-272	0,406573481	0,278	0,003	3,0874E-268	18
Paqr6	2,7987E-270	0,793179724	0,423	0,009	5,0097E-266	18
Col5a2	5,4867E-264	1,363278174	0,66	0,027	9,8211E-260	18
Metrn	1,8789E-263	2,130658199	0,794	0,044	3,3633E-259	18
Col15a1	1,1039E-262	1,001825586	0,443	0,011	1,9761E-258	18
Vcam1	3,9166E-260	0,98869771	0,381	0,008	7,0108E-256	18
Pla2g16	3,6524E-257	1,18407162	0,588	0,022	6,5378E-253	18
Slitrk6	5,6868E-256	1,244788685	0,536	0,018	1,0179E-251	18
Sfrp5	4,9569E-255	1,038974166	0,423	0,01	8,8728E-251	18
Tgfb2	1,7667E-246	3,231106126	0,969	0,077	3,1623E-242	18
Aspa	3,3034E-244	0,81237114	0,392	0,009	5,913E-240	18
Col5a1	4,5679E-243	1,384316244	0,577	0,022	8,1766E-239	18
Adam11	1,8195E-239	0,758373899	0,412	0,01	3,257E-235	18
Grik3	3,7945E-232	1,032639931	0,474	0,015	6,7922E-228	18
Megf6	5,9164E-231	0,426938202	0,268	0,004	1,059E-226	18
Fam198b	9,8374E-231	0,831067199	0,371	0,008	1,7609E-226	18
Wnt6	6,0088E-230	0,552014792	0,289	0,005	1,0756E-225	18
Heyl	6,3593E-224	0,70405174	0,402	0,011	1,1383E-219	18
Col16a1	3,4304E-223	1,477262216	0,66	0,034	6,1404E-219	18
Pdlim4	2,3851E-220	1,292620167	0,588	0,027	4,2693E-216	18
Art3	6,1993E-214	1,230865553	0,526	0,021	1,1097E-209	18
Fabp7	4,0429E-207	3,135719377	0,856	0,069	7,2368E-203	18
Erbb3	1,7961E-206	1,274807646	0,588	0,028	3,2149E-202	18
Ptrf	2,0711E-205	1,379282879	0,66	0,037	3,7072E-201	18
Gm12688	2,1635E-203	0,581511055	0,289	0,006	3,8727E-199	18
Igfbp4	7,688E-203	1,766624675	0,691	0,041	1,3761E-198	18
Gpr37l1	1,7446E-200	2,210740837	0,918	0,084	3,1228E-196	18
Kirrel	1,5565E-198	0,713645807	0,361	0,01	2,7861E-194	18
Zcchc24	1,9994E-196	0,693796793	0,381	0,011	3,5788E-192	18

Lmo4	6,1704E-192	1,809179733	0,804	0,063	1,1045E-187	18
Gpsm2	1,6706E-187	0,848357194	0,443	0,017	2,9903E-183	18
Car12	2,1136E-187	1,601292165	0,536	0,026	3,7833E-183	18
Lgi4	2,4768E-186	2,224959291	0,804	0,067	4,4335E-182	18
Crym	3,0799E-185	0,536720164	0,309	0,007	5,5129E-181	18
Notch1	3,9521E-180	1,033671561	0,474	0,021	7,0743E-176	18
Tspan15	9,7915E-180	1,160635208	0,546	0,029	1,7527E-175	18
Sdc4	2,1613E-179	1,819234992	0,742	0,058	3,8687E-175	18
Sorbs2	3,6553E-178	2,157724351	0,835	0,077	6,5431E-174	18
Lims2	1,0426E-174	0,608372706	0,361	0,011	1,8663E-170	18
Gpx8	5,9844E-174	0,496834823	0,351	0,011	1,0712E-169	18
Fzd8	5,2404E-173	0,520767547	0,361	0,012	9,3803E-169	18
Ltbp1	1,9592E-172	0,897574609	0,443	0,019	3,5069E-168	18
Antxr1	3,0574E-170	1,122822645	0,536	0,029	5,4728E-166	18
Shc4	2,5989E-167	1,126600123	0,505	0,026	4,652E-163	18
Fkbp10	2,0646E-166	0,410100051	0,278	0,007	3,6956E-162	18
Itih5	5,957E-161	2,189401417	0,763	0,068	1,0663E-156	18
Fign	3,3618E-159	0,467436418	0,258	0,006	6,0177E-155	18
Rassf2	5,6103E-159	0,64966683	0,309	0,009	1,0042E-154	18
Pmp22	2,8609E-157	2,141860546	0,814	0,082	5,1209E-153	18
Pdgfb	5,7818E-154	1,061089598	0,464	0,024	1,0349E-149	18
Gpm6b	1,7356E-153	2,164823103	0,876	0,104	3,1067E-149	18
Fxyd1	2,1587E-151	3,067087531	0,959	0,135	3,864E-147	18
Cdh4	1,3491E-149	0,567033941	0,278	0,008	2,4148E-145	18
Mmp14	1,4257E-148	0,977438241	0,443	0,022	2,552E-144	18
Vgll3	6,3638E-140	0,639965427	0,309	0,011	1,1391E-135	18
Itgav	1,3743E-139	0,961457324	0,433	0,023	2,46E-135	18
Myl9	2,6127E-135	0,855432115	0,392	0,019	4,6766E-131	18
Mmp17	5,4349E-132	0,544114588	0,289	0,01	9,7284E-128	18
Hmgcs2	1,5527E-131	1,169616658	0,485	0,031	2,7792E-127	18
Col1a2	5,6542E-130	0,690765552	0,464	0,028	1,0121E-125	18
Fam129a	1,0611E-129	1,534986943	0,588	0,049	1,8994E-125	18
Lamb1	3,0584E-128	0,945307563	0,412	0,022	5,4746E-124	18
Reg4	0	8,310071548	0,988	0,046	0	19
Afp	0	6,021526295	0,894	0,012	0	19
Tff3	0	4,811042997	0,941	0,02	0	19
Tph1	0	4,247041089	0,929	0,012	0	19
Lgals2	0	4,051965241	0,965	0,033	0	19
Ddc	0	3,685566356	0,988	0,048	0	19
Cldn7	0	3,674242099	1	0,032	0	19
Krt8	0	3,609281446	0,988	0,036	0	19
Trpa1	0	3,527035091	0,941	0,003	0	19
Neurod1	0	3,503990862	1	0,016	0	19
Slc18a1	0	3,379638626	0,953	0,013	0	19
Ambp	0	3,324315377	0,729	0,007	0	19
Tpbg	0	3,23019503	0,988	0,009	0	19

Krt7	0	3,099179275	0,847	0,018	0	19
Cdh17	0	3,088535833	0,965	0,019	0	19
Epcam	0	3,043052084	0,953	0,028	0	19
Krt20	0	3,020639425	0,812	0,015	0	19
Tm4sf5	0	2,808587936	0,929	0,01	0	19
Serpinb1a	0	2,77351174	0,918	0,019	0	19
Plac8	0	2,769512634	0,871	0,034	0	19
Lypd8	0	2,736347716	0,965	0,022	0	19
Smim22	0	2,724150919	0,894	0,02	0	19
Itpr3	0	2,679348735	0,953	0,02	0	19
Hepacam2	0	2,550725817	0,882	0,013	0	19
Fxyd3	0	2,501768666	0,682	0,021	0	19
Tspan1	0	2,494387475	0,941	0,014	0	19
Adh1	0	2,395115174	0,871	0,012	0	19
Lhx1	0	2,36811592	0,894	0,008	0	19
Adgrg4	0	2,233930265	0,788	0,006	0	19
Cdhr5	0	2,201550083	0,894	0,014	0	19
Slc38a11	0	2,087320437	0,847	0,023	0	19
Hopx	0	2,082185898	0,871	0,02	0	19
Tm4sf20	0	2,076776943	0,8	0,015	0	19
Krt18	0	2,011063488	0,824	0,016	0	19
Muc13	0	1,993810251	0,882	0,023	0	19
Atp8b1	0	1,915235217	0,894	0,019	0	19
Fbp2	0	1,913338757	0,859	0,015	0	19
Rbp2	0	1,903771127	0,6	0,011	0	19
Lmx1a	0	1,852313813	0,882	0,004	0	19
Ceacam20	0	1,829216479	0,812	0,008	0	19
Cldn3	0	1,816081353	0,847	0,023	0	19
Ucn3	0	1,809973425	0,4	0,001	0	19
Fev	0	1,789696444	0,812	0,004	0	19
Acvrl1	0	1,752716522	0,753	0,008	0	19
Foxa1	0	1,742744377	0,706	0,008	0	19
Hnf4a	0	1,740392487	0,882	0,014	0	19
Onecut2	0	1,737437083	0,447	0,007	0	19
Smim24	0	1,688805388	0,788	0,022	0	19
Pcp4l1	0	1,678724884	0,612	0,007	0	19
Reep6	0	1,678371383	0,788	0,016	0	19
Slc13a1	0	1,647432383	0,776	0,006	0	19
Cdh1	0	1,638650932	0,812	0,017	0	19
Vil1	0	1,628673394	0,788	0,021	0	19
Gpa33	0	1,616619231	0,812	0,013	0	19
Slc5a9	0	1,611494633	0,647	0,003	0	19
Cyp2c65	0	1,610176727	0,729	0,007	0	19
2210407C18Rik	0	1,563242364	0,506	0,007	0	19
Gucy2c	0	1,550755064	0,776	0,013	0	19
Gm609	0	1,543811875	0,588	0,013	0	19

Prlr	0	1,486042696	0,541	0,01	0	19
Irf6	0	1,483346909	0,824	0,013	0	19
Ffar2	0	1,481373195	0,647	0,007	0	19
Nkx2-2	0	1,458487895	0,741	0,006	0	19
Myo15b	0	1,420950641	0,682	0,014	0	19
Rbm47	0	1,419495783	0,706	0,016	0	19
Gipc2	0	1,41519878	0,765	0,017	0	19
Pax6	0	1,389977899	0,671	0,011	0	19
Cldn4	0	1,385694161	0,635	0,007	0	19
H2-Q2	0	1,379355328	0,682	0,011	0	19
Pitx2	0	1,370084953	0,494	0,008	0	19
Pappa2	0	1,367386365	0,518	0,002	0	19
Rfx6	0	1,353423712	0,671	0,006	0	19
Agr3	0	1,346967994	0,494	0,002	0	19
Apoc3	0	1,328592642	0,518	0,004	0	19
Nt5e	0	1,321582675	0,506	0,01	0	19
Plet1	0	1,320126381	0,494	0,003	0	19
Egfr	0	1,300135497	0,694	0,009	0	19
Dpp4	0	1,291693619	0,682	0,011	0	19
Cwh43	0	1,290186733	0,341	0,004	0	19
Ms4a8a	0	1,250747621	0,576	0,013	0	19
Prr15l	0	1,246389328	0,647	0,018	0	19
Pde11a	0	1,232756379	0,659	0,016	0	19
Cdhr2	0	1,23067491	0,612	0,012	0	19
Il18r1	0	1,227292587	0,624	0,002	0	19
Tmigd3	0	1,202873143	0,729	0,001	0	19
Gata6	0	1,187485497	0,671	0,01	0	19
Serpinf2	0	1,177873798	0,506	0,003	0	19
Glis3	0	1,154205682	0,647	0,003	0	19
Cldn2	0	1,152623598	0,6	0,01	0	19
C1qa	0	1,1499468	0,518	0,003	0	19
Baiap2l2	0	1,120929597	0,682	0,019	0	19
Rpp25	0	1,119392037	0,529	0,001	0	19
Pls1	0	1,105151015	0,718	0,017	0	19
Foxa2	0	1,097462663	0,494	0,004	0	19
Slc6a19	0	1,096165928	0,588	0,014	0	19
Sned1	0	1,094554484	0,624	0,017	0	19
Gm15200	0	1,090470082	0,4	0,003	0	19
Phldb2	0	1,089766247	0,624	0,014	0	19
Elf3	0	1,085221889	0,624	0,016	0	19
Myl7	0	1,065730665	0,4	0,004	0	19
Fam83e	0	1,058790991	0,635	0,01	0	19
Col3a1	0	6,714962104	0,964	0,028	0	20
Col1a2	0	6,398214284	0,976	0,024	0	20
Col1a1	0	6,342951608	0,94	0,009	0	20
Adamdec1	0	5,881756954	0,702	0,008	0	20

Dcn	0	5,459504957	0,905	0,004	0	20
Mgp	0	5,132593421	0,667	0,005	0	20
Acta2	0	3,937560309	0,464	0,006	0	20
Col6a1	0	3,598490771	0,929	0,02	0	20
Col6a3	0	3,558959241	0,952	0,009	0	20
Dpt	0	3,295387766	0,726	0,001	0	20
Lum	0	3,209779996	0,798	0,001	0	20
Cxcl12	0	3,108492252	0,905	0,012	0	20
Col5a1	0	2,959368093	0,893	0,02	0	20
Sfrp1	0	2,957347834	0,81	0,022	0	20
Col5a2	0	2,728829368	0,857	0,026	0	20
Mfap4	0	2,722192021	0,702	0,001	0	20
Aspn	0	2,563408512	0,786	0,001	0	20
Lamb1	0	2,558045245	0,857	0,019	0	20
Rarres2	0	2,522632646	0,857	0,026	0	20
Rgs5	0	2,469750591	0,726	0,007	0	20
Fn1	0	2,410224833	0,81	0,008	0	20
Clec3b	0	2,398431738	0,702	0,003	0	20
Fbln1	0	2,378642088	0,702	0,01	0	20
Ccl11	0	2,351598864	0,655	0,001	0	20
Sparcl1	0	2,34725037	0,762	0,014	0	20
Lama2	0	2,180354146	0,821	0,013	0	20
Ifitm1	0	2,117885846	0,679	0,003	0	20
Snhg18	0	2,072648757	0,821	0,027	0	20
Pdgfra	0	2,002911252	0,762	0,001	0	20
Htra3	0	1,996791123	0,69	0,002	0	20
Spon2	0	1,858207966	0,643	0,001	0	20
Svep1	0	1,805673192	0,631	0,001	0	20
Ppic	0	1,77053159	0,774	0,013	0	20
Loxl2	0	1,727096926	0,762	0,02	0	20
Tnxb	0	1,725082717	0,667	0,004	0	20
Ogn	0	1,676088705	0,393	0,002	0	20
Ms4a4d	0	1,64522546	0,643	0,001	0	20
Igf1	0	1,622045359	0,464	0,001	0	20
C1s1	0	1,618791896	0,69	0,013	0	20
Cd34	0	1,57978875	0,56	0,008	0	20
Tgfbi	0	1,550501219	0,595	0,006	0	20
Nkx2-3	0	1,550147737	0,702	0,003	0	20
Nupr1	0	1,54567211	0,56	0,006	0	20
Col6a5	0	1,51228039	0,429	0,001	0	20
Angptl1	0	1,475296394	0,452	0,002	0	20
P2ry14	0	1,474474197	0,702	0,019	0	20
Pltp	0	1,457882932	0,595	0,003	0	20
Pi15	0	1,422180508	0,583	0,007	0	20
Tcf21	0	1,420240498	0,619	0,001	0	20
Kdelr3	0	1,416424447	0,643	0,003	0	20

Mfap5	0	1,368010539	0,369	0,001	0	20
Adamts2	0	1,352660897	0,595	0,006	0	20
Tgfbr2	0	1,282820811	0,619	0,012	0	20
Lhfp	0	1,262169014	0,607	0,011	0	20
Bicc1	0	1,239352484	0,69	0,008	0	20
Ltbp1	0	1,184200095	0,631	0,017	0	20
Tnc	0	1,167474699	0,464	0,007	0	20
Lpl	0	1,107408932	0,452	0,007	0	20
Abi3bp	0	1,101471837	0,488	0,008	0	20
Pcsk5	0	1,092008921	0,571	0,006	0	20
Cd302	0	1,090496344	0,512	0,01	0	20
Agt	0	1,080207132	0,381	0,002	0	20
Cpz	0	1,075135162	0,286	0	0	20
Anxa1	0	1,053701936	0,512	0,003	0	20
Tnfaip2	0	0,9779876	0,417	0,003	0	20
Aldh1a2	0	0,964775734	0,488	0	0	20
Meis2	0	0,959872269	0,464	0,007	0	20
Axl	0	0,942920899	0,548	0,01	0	20
Rhoj	0	0,937716607	0,512	0,008	0	20
Lox	0	0,935848553	0,464	0,001	0	20
Thbd	0	0,88621735	0,369	0,004	0	20
Fzd2	0	0,858202974	0,464	0,004	0	20
Pdgfrb	0	0,782095098	0,405	0,003	0	20
Shisa3	0	0,776690028	0,345	0,001	0	20
Thbs2	0	0,712082645	0,429	0,003	0	20
C1qtnf2	0	0,679916247	0,44	0,003	0	20
Oaf	0	0,675196826	0,417	0,006	0	20
Stc2	0	0,67193725	0,333	0,001	0	20
Foxf1	0	0,639729472	0,381	0,001	0	20
D630033O11Rik	0	0,596061349	0,381	0	0	20
Lrrc32	0	0,591023095	0,298	0,002	0	20
Vstm4	0	0,568579989	0,321	0,003	0	20
Ggt5	0	0,559586105	0,321	0,002	0	20
Hoxc8	0	0,513197066	0,262	0,001	0	20
Prelp	0	0,504512072	0,321	0,003	0	20
Mrc2	0	0,499902129	0,321	0,002	0	20
Srpx2	0	0,489408076	0,262	0	0	20
Rem1	0	0,400569517	0,25	0,001	0	20
Pcdh18	0	0,387666184	0,25	0,001	0	20
Adamts19	0	0,375713936	0,262	0	0	20
Egflam	1,4468E-306	0,544548129	0,333	0,004	2,5898E-302	20
Eva1b	1,6335E-306	1,127901802	0,583	0,015	2,924E-302	20
Ntn1	1,3255E-305	0,888904057	0,44	0,008	2,3727E-301	20
Mmp14	1,4401E-301	1,564576006	0,667	0,021	2,5778E-297	20
Zfp36l1	2,6415E-301	1,703940202	0,762	0,028	4,7284E-297	20
Hmcn2	5,0841E-297	2,298367504	0,845	0,037	9,1006E-293	20

Myl9	3,1598E-295	2,913147956	0,607	0,017	5,6561E-291	20
Hspg2	1,8168E-292	1,617338616	0,69	0,023	3,252E-288	20
Fkbp10	1,6326E-291	0,642015596	0,393	0,006	2,9224E-287	20
Il33	3,9604E-290	0,841047996	0,25	0,002	7,0891E-286	20
Fam198b	1,7628E-283	0,945332929	0,44	0,008	3,1555E-279	20
Brinp2	0	1,903251888	0,81	0,026	0	21
Oprk1	0	1,899772131	0,833	0,006	0	21
Ndufa4l2	0	1,664685144	0,452	0,005	0	21
Gm2694	0	0,807226761	0,464	0,006	0	21
Cbln1	0	0,49746102	0,333	0,003	0	21
Penk	8,2716E-274	2,281800364	0,405	0,007	1,4806E-269	21
Nxph4	2,1404E-197	1,250103881	0,667	0,034	3,8314E-193	21
Cckar	5,8711E-190	0,41871219	0,25	0,004	1,0509E-185	21
Cyb561	4,69E-159	1,423291049	0,69	0,046	8,3951E-155	21
Specc1	2,2812E-158	0,699369299	0,476	0,021	4,0833E-154	21
Rgs4	5,1412E-147	1,948417015	0,881	0,09	9,2028E-143	21
Pi15	2,5491E-143	0,596932201	0,31	0,009	4,5629E-139	21
Agtr1a	8,1892E-138	0,555586713	0,381	0,015	1,4659E-133	21
Rbp4	9,8792E-134	0,906407768	0,429	0,02	1,7684E-129	21
Pcp4	2,9264E-124	2,04402448	0,607	0,047	5,2382E-120	21
Gch1	3,1773E-119	1,897244229	0,893	0,115	5,6874E-115	21
Pmp22	5,6275E-114	1,386572422	0,774	0,083	1,0073E-109	21
Spon1	2,7116E-113	0,668215568	0,393	0,02	4,8538E-109	21
Ptn	6,4356E-110	1,975842039	0,798	0,097	1,152E-105	21
Tac1	1,30922E-98	3,724493962	0,94	0,173	2,3435E-94	21
Gda	1,29906E-94	0,792018587	0,274	0,011	2,32532E-90	21
Rgcc	2,764E-83	1,018340352	0,5	0,047	4,94756E-79	21
Dsc3	2,16453E-67	0,629421635	0,381	0,032	3,87451E-63	21
Shf	6,91356E-67	0,753399026	0,536	0,064	1,23753E-62	21
Tns3	9,00741E-66	1,443407707	0,845	0,193	1,61233E-61	21
Tm4sf4	4,67624E-65	2,63853806	0,988	0,356	8,37046E-61	21
Hgf	1,159E-64	0,390767007	0,25	0,014	2,07462E-60	21
Tpd52l1	3,66481E-62	1,133677165	0,655	0,108	6,56002E-58	21
Csmd3	1,85848E-59	0,373472302	0,25	0,016	3,32667E-55	21
Hmcn2	1,70161E-57	0,473291107	0,405	0,041	3,04588E-53	21
Sag	9,83094E-53	0,80946942	0,512	0,074	1,75974E-48	21
Prkcb	2,43437E-45	0,714722068	0,381	0,048	4,35752E-41	21
Rps29	6,9676E-45	1,345870869	1	0,996	1,2472E-40	21
Chgb	5,18463E-44	0,812247219	1	0,765	9,28049E-40	21
Rps21	1,74256E-43	1,465610989	1	0,952	3,11917E-39	21
Higd1a	9,97047E-43	1,336518572	0,81	0,251	1,78471E-38	21
Rps8	3,63551E-42	1,291287923	1	0,978	6,50755E-38	21
Pdpn	6,34296E-42	0,600855138	0,405	0,056	1,13539E-37	21
Syt6	2,06617E-41	0,979693457	0,548	0,105	3,69845E-37	21
Aldoa	1,20949E-40	1,107690367	1	0,995	2,16499E-36	21
Rps3a1	3,27681E-40	1,370278804	1	0,844	5,86549E-36	21

Satb1	7,64706E-40	1,015957089	0,667	0,164	1,36882E-35	21
Fam183b	1,01399E-38	1,03275192	0,56	0,119	1,81505E-34	21
Oaz1	1,97733E-37	1,214303974	1	0,929	3,53942E-33	21
Chchd2	5,68316E-37	1,271171675	1	0,921	1,01729E-32	21
Phgdh	7,66134E-37	0,707296572	0,5	0,094	1,37138E-32	21
Dmkn	7,89597E-36	1,335256498	0,881	0,344	1,41338E-31	21
Slc25a4	1,50271E-35	1,068027629	1	0,97	2,68984E-31	21
Atp1b1	2,2842E-35	1,325090725	1	0,876	4,08872E-31	21
Tmsb4x	3,53459E-35	0,891550678	1	0,998	6,32691E-31	21
Rpl3	3,74546E-35	1,225454809	1	0,922	6,70438E-31	21
Rpl21	9,11923E-35	1,044821692	1	0,968	1,63234E-30	21
Rps27	1,98585E-34	1,262730844	1	0,873	3,55467E-30	21
Rps27a	2,50245E-34	1,15442107	1	0,932	4,47939E-30	21
Ngb	2,82425E-34	0,710320394	0,417	0,072	5,05541E-30	21
Metrn	3,64545E-34	0,413781655	0,345	0,049	6,52535E-30	21
Gapdh	4,31382E-34	1,267260152	1	0,93	7,72175E-30	21
Ubb	7,06584E-34	1,134527814	1	0,987	1,26479E-29	21
Ramp1	7,49938E-34	1,391693586	0,952	0,56	1,34239E-29	21
Stmn2	9,2356E-34	0,879791975	1	0,99	1,65317E-29	21
Susd4	9,27523E-34	0,401929517	0,274	0,032	1,66027E-29	21
Ank1	1,1045E-33	0,363942032	0,274	0,032	1,97706E-29	21
Tpt1	1,89275E-33	1,053223107	1	0,959	3,38803E-29	21
Hint1	6,39679E-33	1,136719848	1	0,884	1,14502E-28	21
Tmem158	1,0924E-32	1,314274342	0,964	0,71	1,9554E-28	21
Rpl39	1,437E-32	1,137402658	1	0,938	2,57223E-28	21
Rpl37	2,58265E-32	1,051159238	1	0,986	4,62295E-28	21
Rps24	2,66166E-32	1,119489882	1	0,967	4,76437E-28	21
Rps3	7,31327E-32	1,144845113	1	0,873	1,30908E-27	21
Rpl6	1,33949E-31	1,06143871	1	0,973	2,39769E-27	21
Rpl30	2,49583E-31	1,098270264	1	0,828	4,46754E-27	21
Rps12	3,9383E-31	1,060704859	0,988	0,84	7,04955E-27	21
Ly6h	4,85999E-31	1,390537401	0,905	0,491	8,69938E-27	21
Rpl23	5,84201E-31	0,937842698	1	0,992	1,04572E-26	21
Rps4x	7,37901E-31	1,109738634	1	0,89	1,32084E-26	21
Rpl24	1,42813E-30	1,079262919	1	0,948	2,55636E-26	21
Rpl9	2,45487E-30	1,162485725	1	0,829	4,39422E-26	21
Rpl27a	5,21162E-30	0,94649423	1	0,964	9,32881E-26	21
Eef1a1	6,44082E-30	0,888383267	1	0,999	1,15291E-25	21
Rpl37a	6,78008E-30	0,950821463	1	0,987	1,21363E-25	21
Ndufa4	7,99981E-30	1,150453481	1	0,845	1,43197E-25	21
Nap1l5	9,79947E-30	1,086752745	0,988	0,923	1,75411E-25	21
Rps20	1,90877E-29	1,019417679	1	0,905	3,4167E-25	21
Rpl35a	2,52102E-29	0,99181426	1	0,926	4,51262E-25	21
Cox4i1	3,73183E-29	1,059956056	1	0,892	6,67998E-25	21
Sncb	4,85563E-29	1,129703756	0,679	0,237	8,69157E-25	21
Rpl12	5,23259E-29	0,927447261	1	0,82	9,36634E-25	21

Naca	5,6971E-29	0,989360055	1	0,879	1,01978E-24	21
Rps5	1,01371E-28	0,977821094	1	0,891	1,81455E-24	21
Tubb4b	1,04961E-28	1,294468845	1	0,79	1,87879E-24	21
Rps6	3,12723E-28	0,986068068	1	0,855	5,59774E-24	21
Rpl17	4,63341E-28	1,003321634	1	0,919	8,2938E-24	21
Rpl36a	4,99011E-28	1,084474326	1	0,78	8,9323E-24	21
Cox6c	7,29964E-28	1,048081907	1	0,877	1,30664E-23	21
Rps15a	8,78995E-28	0,928551044	1	0,892	1,5734E-23	21
Ffar3	1,73803E-27	0,460885601	0,381	0,071	3,11107E-23	21
Rpl38	2,61021E-27	0,920196027	1	0,993	4,67228E-23	21
Ociad2	4,34378E-27	0,694116518	0,56	0,154	7,77536E-23	21
Rps10	4,89439E-27	0,958527958	1	0,924	8,76096E-23	21
Cox8a	6,38731E-27	0,972631644	1	0,934	1,14333E-22	21
Tomm7	7,42972E-27	1,022385354	1	0,784	1,32992E-22	21
Paip2b	1,02095E-58	4,235874424	1	0,504	1,82751E-54	22
Sst	1,08032E-23	1,414401449	1	0,831	1,93378E-19	22
Avpr1a	2,03344E-16	0,54921314	0,662	0,252	3,63985E-12	22
Tshz2	2,93556E-14	0,791254499	1	0,844	5,25465E-10	22
Fxyd7	3,8382E-14	0,742906634	1	0,76	6,87038E-10	22
Trp53i11	8,58081E-12	0,655172461	0,988	0,674	1,53596E-07	22
Hoxa5	9,70522E-12	0,624570254	1	0,931	1,73723E-07	22
Calcb	2,46555E-11	0,692523375	0,912	0,544	4,41334E-07	22
Smarca2	8,05749E-11	0,576574705	0,988	0,937	1,44229E-06	22
Ly6e	1,29478E-10	0,488020767	0,887	0,52	2,31765E-06	22
Rspo2	2,19954E-10	0,534476146	0,55	0,245	3,93719E-06	22
Gm42418	2,2593E-10	0,872199156	1	1	4,04415E-06	22
Pcsk1n	3,60377E-10	0,405039954	1	0,996	6,45075E-06	22
Slitrk5	3,615E-10	0,264783586	0,35	0,116	6,47085E-06	22
Atp1b1	3,91638E-10	0,545589475	0,988	0,876	7,01032E-06	22
Gfra2	4,13183E-10	0,581214839	0,787	0,463	7,39597E-06	22
Ryr2	9,64007E-10	0,429250539	0,588	0,279	1,72557E-05	22
Lrrtm1	1,42819E-09	0,411865805	0,613	0,301	2,55647E-05	22
Bche	5,75E-09	0,461046191	0,963	0,777	0,000102925	22
Pcdh7	1,3976E-08	0,473015913	0,713	0,385	0,000250171	22
Igfbp5	1,71757E-08	0,436492396	0,488	0,222	0,000307444	22
Dmkn	2,11311E-08	0,558689548	0,65	0,346	0,000378247	22
Vipr2	3,81784E-08	0,400054366	0,575	0,278	0,000683393	22
Mcam	4,33634E-08	0,582414411	0,85	0,667	0,000776205	22
Bnc2	4,55657E-08	0,404161736	0,5	0,235	0,000815627	22
Ache	6,49372E-08	0,460786932	0,975	0,952	0,001162377	22
Sphkap	8,30591E-08	0,475400347	0,562	0,279	0,001486759	22
Csrp2	1,25121E-07	0,530716583	0,662	0,41	0,002239668	22
Filip1	1,38988E-07	0,40644457	0,95	0,804	0,002487882	22
Nfix	1,52557E-07	0,522724104	0,887	0,755	0,002730765	22
Brinp1	2,27305E-07	0,413267234	0,6	0,336	0,004068752	22
Flrt1	2,58861E-07	0,263493205	0,4	0,173	0,004633607	22

Pla2g7	2,87857E-07	0,258340104	0,45	0,202	0,005152642	22
Fam19a5	2,91721E-07	0,347536913	0,725	0,398	0,005221802	22
Serping1	4,65566E-07	0,56231138	0,637	0,402	0,00833364	22
Kif22	5,79124E-07	0,514269937	0,912	0,73	0,010366324	22
Slc10a4	9,20508E-07	0,452930713	0,938	0,793	0,016477087	22
Bcl2	1,05294E-06	0,498925819	0,7	0,463	0,018847656	22
Zfp503	1,34849E-06	0,35712925	0,412	0,201	0,024138009	22
Slit3	1,57817E-06	0,347390822	0,625	0,373	0,028249223	22
Scube1	1,70019E-06	0,386211611	0,975	0,891	0,030433447	22
Tcf7l2	2,15757E-06	0,317870845	0,975	0,844	0,038620566	22
Rprm	2,5526E-06	0,301349087	0,525	0,274	0,045691623	22
Spock2	3,14928E-06	0,282088586	1	0,966	0,056372105	22
Cdh2	4,35419E-06	0,395428277	0,963	0,913	0,077940054	22
Prmt8	4,52679E-06	0,275313952	0,325	0,144	0,08102947	22
Vamp1	7,61902E-06	0,429453206	0,887	0,792	0,136380494	22
Chd3	8,56434E-06	0,375493982	1	0,946	0,153301766	22
Epha5	1,04721E-05	0,370046474	0,425	0,226	0,187450796	22
Gdf10	1,0835E-05	0,273264939	0,35	0,165	0,193946945	22
Rtp4	1,79898E-05	0,274844362	0,4	0,203	0,322016943	22
Ebf3	2,41181E-05	0,310418939	0,325	0,158	0,431714515	22
Rasd2	2,49426E-05	0,29637573	0,475	0,259	0,44647284	22
Rph3a	3,37725E-05	0,381508891	0,825	0,63	0,604528118	22
Nrp2	3,38417E-05	0,302679187	0,525	0,303	0,605766637	22
Lars2	3,52826E-05	0,369925089	0,988	0,989	0,631559143	22
Rab3b	4,44501E-05	0,309710856	0,688	0,459	0,795656784	22
Tspan3	6,82862E-05	0,320609484	0,975	0,9	1	22
Ddah1	8,71922E-05	0,342486053	0,65	0,413	1	22
Dip2b	9,19564E-05	0,336108221	0,75	0,574	1	22
Sema3c	9,26037E-05	0,283716709	0,45	0,254	1	22
Lrrn2	9,40965E-05	0,335806888	0,713	0,512	1	22
Hoxb5	0,000107314	0,365059812	1	0,946	1	22
Bend5	0,000120297	0,427500317	0,588	0,412	1	22
Prnp	0,000125939	0,250572732	1	0,972	1	22
Tcaf1	0,000126803	0,268853313	0,988	0,909	1	22
Pth1r	0,000131031	0,281635389	0,412	0,237	1	22
Casz1	0,000138281	0,315693414	0,6	0,386	1	22
Cabp1	0,00018567	0,349457841	0,637	0,459	1	22
Adamts5	0,000199636	0,267577849	0,462	0,276	1	22
Sez6l	0,000221205	0,316748893	0,675	0,491	1	22
Ctnna1	0,000237428	0,325249963	0,675	0,513	1	22
Piezo1	0,000276746	0,280467799	0,388	0,218	1	22
Ahi1	0,000334165	0,26766845	1	0,99	1	22
Ly6h	0,000372849	0,305155975	0,675	0,493	1	22
Slc18a3	0,000377081	0,297085706	0,738	0,532	1	22
Ntm	0,000393332	0,389798254	0,3	0,155	1	22
Susd2	0,000398467	0,274493592	0,425	0,254	1	22

Apba2	0,000435843	0,482999748	0,8	0,7	1	22
Nrxn2	0,000508809	0,312526436	0,988	0,948	1	22
Slc5a7	0,000529031	0,278819691	0,812	0,616	1	22
Kif21a	0,000609511	0,2668247	0,988	0,964	1	22
Chd5	0,000758073	0,308688984	0,988	0,949	1	22
Map1a	0,000790956	0,30493871	0,887	0,856	1	22
Itga6	0,000795795	0,27133996	0,7	0,489	1	22
Cxxc4	0,000881542	0,256822855	0,8	0,607	1	22
Sel1l3	0,00088262	0,34843548	0,713	0,592	1	22
B3gnt2	0,000885654	0,280301921	0,438	0,284	1	22
Zcchc12	0,000995945	0,301972078	0,95	0,866	1	22
Ina	0,00123719	0,326143322	0,825	0,736	1	22
Myh10	0,001612509	0,294928567	0,637	0,548	1	22
Fam21	0,001797981	0,325480735	0,65	0,55	1	22
Higd1a	0,002253661	0,325658032	0,388	0,255	1	22
Dlgap2	0,002457016	0,265052233	0,462	0,322	1	22
Gucy1b3	0,002511787	0,256023776	0,725	0,593	1	22
Prkcdbp	0,002776123	0,304199789	0,475	0,335	1	22
Stat3	0,002913414	0,256545895	0,887	0,733	1	22
Bscl2	0,003579225	0,26933142	0,738	0,658	1	22
Dlgap3	0,003713071	0,340300845	0,537	0,428	1	22
Tekt2	0,004946272	0,255950339	0,637	0,486	1	22
Vat1l	0,005408695	0,296063514	0,912	0,817	1	22
Fabp2	0	4,817732413	0,905	0,016	0	23
Lypd8	0	4,760928868	0,946	0,023	0	23
Spink1	0	4,749488357	0,676	0,01	0	23
Cldn7	0	4,573304674	0,986	0,033	0	23
Fabp1	0	4,367048375	0,527	0,008	0	23
2200002D01Rik	0	4,169233033	0,892	0,021	0	23
Krt8	0	4,080099638	0,973	0,038	0	23
Epcam	0	3,925529527	0,986	0,029	0	23
Smim24	0	3,923307517	0,892	0,022	0	23
Reg1	0	3,786920836	0,338	0,003	0	23
Apob	0	3,763398623	0,811	0,015	0	23
Anpep	0	3,677848582	0,757	0,009	0	23
Guca2b	0	3,657923892	0,554	0,005	0	23
Plac8	0	3,645651802	0,932	0,034	0	23
Muc13	0	3,64017564	0,959	0,023	0	23
Prap1	0	3,568475789	0,824	0,008	0	23
Aldob	0	3,548924739	0,946	0,011	0	23
S100g	0	3,512173945	0,568	0,006	0	23
Mgam	0	3,501906035	0,946	0,008	0	23
Slc5a1	0	3,259507751	0,77	0,008	0	23
Guca2a	0	3,230816567	0,473	0,008	0	23
Clec2h	0	3,225814533	0,635	0,004	0	23
Muc3	0	3,058386535	0,838	0,006	0	23

Pls1	0	2,994973043	0,811	0,017	0	23
Vil1	0	2,975094776	0,878	0,022	0	23
2210407C18Rik	0	2,953933274	0,581	0,007	0	23
Slc51a	0	2,832332027	0,662	0,003	0	23
Phgr1	0	2,806714409	0,824	0,009	0	23
Cdhr5	0	2,731430314	0,757	0,016	0	23
Ces2e	0	2,711464407	0,703	0,004	0	23
Cps1	0	2,703123792	0,608	0,01	0	23
Maf	0	2,692668523	0,676	0,015	0	23
Cdh17	0	2,641434576	0,919	0,021	0	23
Aldh1b1	0	2,6146944	0,865	0,032	0	23
Tm4sf5	0	2,583005767	0,77	0,013	0	23
Gpx2	0	2,574720083	0,77	0,02	0	23
Enpep	0	2,520562524	0,568	0,004	0	23
Ace2	0	2,485448699	0,595	0,005	0	23
Serpinb1a	0	2,382534346	0,838	0,021	0	23
Myo1a	0	2,375737195	0,838	0,01	0	23
Fbp2	0	2,345537842	0,892	0,016	0	23
Gsta1	0	2,303193472	0,338	0,002	0	23
Ifi27l2b	0	2,289987586	0,757	0,008	0	23
Myo15b	0	2,258206193	0,784	0,014	0	23
Cldn3	0	2,249097681	0,824	0,024	0	23
Tm4sf20	0	2,188206806	0,811	0,015	0	23
Gda	0	2,187467704	0,554	0,009	0	23
Clca4b	0	2,146571331	0,351	0,001	0	23
Mapk13	0	2,141353324	0,892	0,011	0	23
Hnf4a	0	2,126900848	0,892	0,015	0	23
Cdh1	0	2,126553355	0,824	0,018	0	23
Cdhr2	0	2,093419732	0,716	0,012	0	23
Ggt1	0	2,086554961	0,5	0,004	0	23
Mep1a	0	2,067634612	0,514	0,005	0	23
Atp8b1	0	2,025401423	0,851	0,021	0	23
Smim22	0	2,004874219	0,784	0,022	0	23
Aoc1	0	1,978080837	0,689	0,001	0	23
Dpp4	0	1,931045307	0,676	0,012	0	23
Alpi	0	1,918302343	0,554	0,001	0	23
Lct	0	1,912682761	0,311	0,001	0	23
Ppp1r1b	0	1,890903658	0,784	0,008	0	23
Klf5	0	1,88013899	0,865	0,017	0	23
Prr15l	0	1,878658391	0,892	0,017	0	23
Dsg2	0	1,869229925	0,865	0,023	0	23
Cyp4f14	0	1,854345469	0,541	0,002	0	23
Mgst1	0	1,846798707	0,757	0,012	0	23
Npc1l1	0	1,830648236	0,581	0,005	0	23
Misp	0	1,819716428	0,838	0,025	0	23
Elf3	0	1,812494992	0,811	0,015	0	23

Dpep1	0	1,795217906	0,446	0,003	0	23
Gpa33	0	1,784931329	0,851	0,014	0	23
Cldn15	0	1,7604579	0,784	0,008	0	23
Reep6	0	1,74186627	0,73	0,018	0	23
Adh6a	0	1,735603256	0,446	0,003	0	23
Slc34a2	0	1,732602778	0,446	0	0	23
Naaladl1	0	1,729161432	0,473	0,004	0	23
Dsp	0	1,728260135	0,811	0,014	0	23
H2-Q2	0	1,717137127	0,689	0,012	0	23
Rbm47	0	1,694438339	0,824	0,016	0	23
Mogat2	0	1,672425828	0,459	0,001	0	23
Sult1b1	0	1,671061562	0,581	0,004	0	23
Sult1d1	0	1,668841111	0,649	0,008	0	23
Creb3l3	0	1,648481065	0,473	0,004	0	23
Ugt2b34	0	1,624375654	0,635	0,007	0	23
Cyp3a13	0	1,597365132	0,541	0,006	0	23
Ceacam1	0	1,593492846	0,676	0,012	0	23
Gipc2	0	1,584916458	0,851	0,017	0	23
Tmigd1	0	1,57554869	0,284	0,002	0	23
Ppp1r14d	0	1,554676337	0,77	0,007	0	23
Myo7b	0	1,542592128	0,689	0,01	0	23
Chp2	0	1,520222262	0,595	0,002	0	23
Cldn2	0	1,512499982	0,743	0,01	0	23
Cideb	0	1,490437359	0,581	0,004	0	23
Ace	0	1,477752619	0,392	0,004	0	23
Gm1123	0	1,453616756	0,635	0,007	0	23
Glod5	0	1,451914909	0,541	0,002	0	23
Prlr	0	1,422201776	0,662	0,01	0	23
Arg2	0	1,415478508	0,419	0,003	0	23
Slc51b	0	1,40372917	0,392	0,001	0	23
Lad1	0	1,394376613	0,77	0,011	0	23
Cdx2	0	1,375285812	0,689	0,009	0	23
Lgals3	9,36609E-51	1,084055885	0,592	0,089	1,67653E-46	24
Tspan8	4,36448E-34	1,274543977	0,62	0,14	7,81242E-30	24
Sst	2,14505E-33	1,885945783	1	0,831	3,83963E-29	24
Npy	6,11033E-32	1,708859508	1	0,847	1,09375E-27	24
Emb	1,32046E-29	0,797877984	0,775	0,231	2,36362E-25	24
Ifitm2	4,03475E-29	1,135122703	1	0,944	7,22219E-25	24
Stmn3	1,79873E-27	1,101238896	1	0,916	3,21972E-23	24
Dmkn	4,61069E-27	1,0932552	0,901	0,345	8,25314E-23	24
Csrp2	5,82174E-26	0,944878269	0,958	0,408	1,04209E-21	24
Calcb	9,60645E-26	1,323795551	1	0,544	1,71955E-21	24
Atp5e	5,13657E-25	1,001021338	1	0,852	9,19446E-21	24
Rpl22l1	8,43339E-25	0,989265672	1	0,788	1,50958E-20	24
Cox6c	9,48823E-25	1,03338982	1	0,877	1,69839E-20	24
Ubb	1,16765E-24	0,993162661	1	0,987	2,0901E-20	24

Ifi27	1,34467E-24	1,029435877	1	0,785	2,40696E-20	24
Atp5k	3,18745E-24	0,939254387	1	0,959	5,70554E-20	24
Hint1	5,33708E-24	0,972136926	1	0,884	9,55337E-20	24
Rpl24	7,57897E-24	0,922820692	1	0,948	1,35664E-19	24
Rpl6	7,76112E-24	0,941447388	1	0,973	1,38924E-19	24
Pdlim2	8,78067E-24	0,952528988	0,775	0,289	1,57174E-19	24
Atp6v1e1	9,41982E-24	0,959010777	1	0,955	1,68615E-19	24
Gm10076	1,71292E-23	1,025266293	1	0,856	3,06612E-19	24
S100a6	2,11041E-23	0,925006289	1	0,994	3,77763E-19	24
Ly6h	2,2932E-23	0,996352324	0,958	0,491	4,10483E-19	24
Rpl37	3,60448E-23	0,892576756	1	0,986	6,45202E-19	24
Rps8	4,04802E-23	0,832607319	1	0,978	7,24596E-19	24
Rpl9	4,68599E-23	1,030688355	1	0,829	8,38793E-19	24
Rpl35a	5,50107E-23	0,862324041	1	0,926	9,84692E-19	24
S100a1	6,10593E-23	0,986670475	1	0,827	1,09296E-18	24
Rps3a1	6,38197E-23	0,979009685	1	0,844	1,14237E-18	24
Prph	7,20619E-23	0,804028685	1	0,963	1,28991E-18	24
Ndufa4	8,35933E-23	0,9318094	1	0,845	1,49632E-18	24
Rps24	9,69807E-23	0,827146569	1	0,967	1,73595E-18	24
Fxyd7	1,09892E-22	1,065722576	1	0,76	1,96706E-18	24
Rpl39	1,83422E-22	0,95859414	1	0,938	3,28325E-18	24
Higd1a	2,89138E-22	0,710785307	0,746	0,252	5,17557E-18	24
Gapdh	3,94741E-22	0,957456399	1	0,931	7,06586E-18	24
Hcrtr1	4,42138E-22	0,376940699	0,366	0,069	7,91428E-18	24
Rps21	4,55247E-22	0,954714036	1	0,952	8,14893E-18	24
Rpl35	5,01006E-22	0,911228905	1	0,84	8,968E-18	24
Rps29	5,17843E-22	0,806116155	1	0,996	9,26939E-18	24
Fdps	5,65285E-22	0,987322988	0,986	0,752	1,01186E-17	24
Aldoa	7,59822E-22	0,721427541	1	0,995	1,36008E-17	24
Fth1	9,30168E-22	0,768075906	1	0,994	1,665E-17	24
Uqcr11	1,11264E-21	0,891282896	1	0,834	1,99162E-17	24
Atp5j2	1,12414E-21	0,973604979	1	0,806	2,01221E-17	24
Rtp4	1,56362E-21	0,664895597	0,648	0,201	2,79888E-17	24
Rpl37a	1,60659E-21	0,818440206	1	0,987	2,87579E-17	24
Cox8a	3,57307E-21	0,801979606	1	0,934	6,3958E-17	24
Rps27a	5,39424E-21	0,836995082	1	0,932	9,65569E-17	24
Rpl38	6,10309E-21	0,795290561	1	0,993	1,09245E-16	24
Atpif1	6,97317E-21	0,905519749	1	0,951	1,2482E-16	24
Rpl19	8,07866E-21	0,844829928	1	0,934	1,44608E-16	24
Rps11	8,25875E-21	0,7697405	1	0,937	1,47832E-16	24
Prkcdbp	1,00539E-20	0,80540003	0,803	0,332	1,79965E-16	24
Rps18	1,0232E-20	0,732959297	1	0,936	1,83152E-16	24
Rps3	1,24185E-20	0,858114669	1	0,873	2,2229E-16	24
Rpl13	1,57208E-20	0,750126115	1	0,957	2,81403E-16	24
Vwc2	1,68312E-20	0,71415663	0,704	0,246	3,01278E-16	24
Ndufa1	1,79497E-20	0,872541419	1	0,608	3,213E-16	24

Rpl3	2,42739E-20	0,839439786	1	0,922	4,34503E-16	24
Map1lc3a	3,52385E-20	0,736637867	1	0,971	6,30768E-16	24
Rps15a	5,94907E-20	0,748528047	1	0,892	1,06488E-15	24
Rpl34	6,74034E-20	0,782304164	1	0,921	1,20652E-15	24
Fst	7,53676E-20	0,884440642	0,746	0,302	1,34908E-15	24
Slc18a3	8,5047E-20	0,879658862	0,972	0,53	1,52234E-15	24
Rps5	1,21781E-19	0,781670684	1	0,891	2,17988E-15	24
Tpt1	1,23004E-19	0,728212812	1	0,959	2,20177E-15	24
Rpl32	1,3787E-19	0,688771634	1	0,968	2,46788E-15	24
Rps14	1,4303E-19	0,72381346	1	0,954	2,56024E-15	24
Rpl21	1,50047E-19	0,668116961	1	0,968	2,68585E-15	24
Gap43	1,92047E-19	0,712056255	1	0,986	3,43765E-15	24
Ndufa3	2,20358E-19	0,829096896	1	0,783	3,9444E-15	24
Rps27	2,23361E-19	0,809466977	1	0,874	3,99816E-15	24
Rps10	2,34824E-19	0,750548781	1	0,924	4,20335E-15	24
Atp5h	2,66289E-19	0,784538468	1	0,875	4,76657E-15	24
Gnb2l1	2,72387E-19	0,748057484	1	0,818	4,87573E-15	24
Nme1	2,82009E-19	0,816050157	0,986	0,674	5,04796E-15	24
Rps7	3,49408E-19	0,854466561	1	0,798	6,25441E-15	24
Rpl18	3,74331E-19	0,754198456	1	0,875	6,70053E-15	24
Ndufb9	3,79641E-19	0,857842982	0,986	0,661	6,79558E-15	24
Sec62	3,86835E-19	0,807791895	1	0,938	6,92434E-15	24
Nell1	4,84374E-19	0,641147134	0,789	0,288	8,6703E-15	24
Rpl23	7,21508E-19	0,609775793	1	0,992	1,2915E-14	24
Rpl36a	7,85656E-19	0,797200442	0,986	0,78	1,40632E-14	24
Kif22	8,39509E-19	0,98560604	0,958	0,73	1,50272E-14	24
Rps13	1,01983E-18	0,726406891	1	0,833	1,82549E-14	24
Pfdn5	1,08438E-18	0,786135335	1	0,765	1,94104E-14	24
Uqcr10	1,12503E-18	0,834613445	1	0,756	2,01381E-14	24
Sep15	1,19629E-18	0,685118792	1	0,832	2,14136E-14	24
Rpl30	1,26344E-18	0,739307347	1	0,828	2,26156E-14	24
Rpl17	1,63264E-18	0,746821137	1	0,919	2,92242E-14	24
Tmsb10	1,83757E-18	0,771663763	1	0,949	3,28924E-14	24
Tm4sf1	1,88091E-18	0,84652213	0,944	0,737	3,36683E-14	24
Crip1	2,00661E-18	0,863312323	1	0,986	3,59183E-14	24
Fam162a	2,54216E-18	0,799728827	0,831	0,399	4,55048E-14	24
Mt3	2,90496E-18	1,033917868	0,958	0,717	5,19988E-14	24
Atp5f1	3,14987E-18	0,843206873	0,986	0,691	5,63826E-14	24
Cox6b1	3,38358E-18	0,777482514	1	0,875	6,05661E-14	24
Chchd2	3,43663E-18	0,811653642	1	0,921	6,15157E-14	24
Tspo	3,90083E-18	0,772423351	0,958	0,659	6,98249E-14	24
Oprk1	0	1,480956981	0,557	0,01	0	25
Brinp2	1,6949E-117	1,476937514	0,514	0,029	3,0338E-113	25
Pi15	7,1604E-113	0,793038414	0,3	0,01	1,2817E-108	25
Specc1	4,0661E-80	0,844849492	0,371	0,022	7,27832E-76	25
Sidt1	2,21355E-55	0,509326433	0,3	0,021	3,96226E-51	25

Tns3	9,96275E-55	2,081728249	0,814	0,194	1,78333E-50	25
Rgs4	4,41839E-52	1,442537819	0,6	0,093	7,90891E-48	25
Cyb561	1,89094E-44	0,843391308	0,414	0,049	3,38479E-40	25
Hmcn2	1,14413E-41	0,828407905	0,371	0,042	2,04799E-37	25
Prkcb	3,11078E-37	0,928901277	0,371	0,048	5,5683E-33	25
Sdk2	5,16127E-32	0,687781475	0,329	0,043	9,23866E-28	25
Nxph4	8,05624E-30	0,555106056	0,3	0,038	1,44207E-25	25
Plce1	5,18807E-25	1,52138491	0,857	0,478	9,28665E-21	25
Dsc3	1,10865E-24	0,52761544	0,257	0,034	1,98448E-20	25
Syt6	4,23943E-24	1,050142606	0,457	0,106	7,58858E-20	25
Lrfn5	4,91884E-22	0,854948339	0,471	0,12	8,80472E-18	25
Gpc6	9,39196E-22	1,224640117	0,857	0,489	1,68116E-17	25
mt-Nd5	5,15529E-20	1,203248646	1	0,999	9,22797E-16	25
Pmp22	6,52041E-20	0,831485971	0,386	0,087	1,16715E-15	25
Sertad4	1,04961E-19	0,755095341	0,371	0,083	1,87879E-15	25
Dlc1	1,06288E-19	0,537036898	0,271	0,045	1,90255E-15	25
Unc80	6,58812E-19	1,113876961	0,914	0,646	1,17927E-14	25
Reps2	1,00916E-18	0,564335673	0,257	0,044	1,8064E-14	25
Col5a3	1,7718E-18	0,689154239	0,371	0,086	3,17152E-14	25
Syne2	4,14699E-18	1,053455452	0,586	0,226	7,42311E-14	25
Akap13	2,53177E-17	1,113661452	1	0,877	4,53186E-13	25
Lrrfip1	2,56839E-17	0,988121297	1	0,904	4,59741E-13	25
mt-Nd1	3,6051E-17	0,68289883	1	1	6,45314E-13	25
Tac1	4,24255E-17	3,129890216	0,5	0,178	7,59416E-13	25
Kcnq1ot1	9,55566E-17	1,160559283	1	0,843	1,71046E-12	25
Meg3	1,08693E-16	1,052062182	1	0,993	1,9456E-12	25
Gch1	2,66758E-15	0,979924362	0,4	0,12	4,77497E-11	25
Pcp4	4,78939E-15	0,50441898	0,257	0,051	8,57302E-11	25
mt-Nd4l	1,8165E-14	0,769931781	1	1	3,25154E-10	25
Shf	4,11708E-14	0,588564587	0,286	0,067	7,36957E-10	25
Ptn	8,55661E-14	0,734676207	0,357	0,101	1,53163E-09	25
Zbtb20	1,04243E-13	1,015746704	1	0,925	1,86594E-09	25
Fam19a1	1,24016E-13	1,074986831	0,714	0,437	2,21989E-09	25
Meis1	2,81186E-13	0,926952213	0,429	0,15	5,03323E-09	25
mt-Atp8	3,0506E-13	0,652072505	1	1	5,46057E-09	25
Gm26917	2,58976E-12	1,442316799	0,771	0,523	4,63568E-08	25
AY036118	2,85787E-12	0,747296042	0,986	0,852	5,11559E-08	25
Dner	7,6034E-12	0,80177531	0,843	0,542	1,36101E-07	25
Gabrg3	9,95039E-12	0,711059491	0,271	0,073	1,78112E-07	25
Pclo	2,1522E-11	0,882529823	0,986	0,807	3,85244E-07	25
Casz1	2,23156E-11	0,94789777	0,671	0,385	3,9945E-07	25
Tm4sf4	2,54884E-11	1,059321143	0,643	0,36	4,56243E-07	25
Kmt2a	2,89172E-11	0,702861724	0,957	0,714	5,17618E-07	25
Edil3	3,24726E-11	0,941240735	0,386	0,141	5,8126E-07	25
Celf4	3,58179E-11	0,659654906	1	0,962	6,41141E-07	25
Sulf2	4,56639E-11	0,784862694	0,571	0,27	8,17383E-07	25

Negr1	5,22001E-11	0,659511168	1	0,945	9,34381E-07	25
Sorl1	6,30564E-11	0,845395816	0,857	0,59	1,12871E-06	25
Gucy1a3	6,6893E-11	0,900748248	0,8	0,505	1,19738E-06	25
1700025G04Rik	1,37819E-10	0,607526554	0,357	0,123	2,46696E-06	25
Snhg11	2,13444E-10	0,730301588	1	0,983	3,82065E-06	25
Eml5	2,4322E-10	0,907453679	0,829	0,552	4,35364E-06	25
Stxbp5	2,66932E-10	0,763755519	0,943	0,675	4,77808E-06	25
Atp1b1	2,71245E-10	0,830170305	1	0,876	4,85528E-06	25
mt-Nd4	3,32821E-10	0,445675287	1	1	5,9575E-06	25
Pura	4,1966E-10	0,71367366	1	0,965	7,51191E-06	25
Hoxb5	6,09342E-10	0,643954892	1	0,946	1,09072E-05	25
Ahi1	7,85797E-10	0,519754278	1	0,99	1,40658E-05	25
Mapt	8,76329E-10	0,685181678	1	0,955	1,56863E-05	25
Satb1	9,2462E-10	0,662518156	0,4	0,167	1,65507E-05	25
Slc5a7	1,22469E-09	0,827015212	0,929	0,615	2,19219E-05	25
Msi2	1,33218E-09	0,820845781	0,929	0,779	2,38459E-05	25
Srrm2	1,66592E-09	0,62215488	1	0,968	2,982E-05	25
Adrbk2	1,94458E-09	0,669429734	1	0,936	3,4808E-05	25
Sgip1	2,96332E-09	0,726797737	0,986	0,831	5,30435E-05	25
Bcar3	2,97309E-09	0,428316915	0,271	0,082	5,32183E-05	25
Rgmb	3,17145E-09	0,963247694	0,543	0,327	5,67689E-05	25
Ubn2	1,01256E-08	0,696429902	0,757	0,565	0,000181248	25
Tshz3	1,02236E-08	0,426519508	0,286	0,093	0,000183002	25
Tnrc6c	1,09636E-08	0,672511647	0,986	0,84	0,000196249	25
Kcnq3	1,17939E-08	0,955022514	0,814	0,569	0,000211111	25
Zeb2	1,24383E-08	0,669895073	0,857	0,651	0,000222646	25
Bub3	1,26637E-08	0,789001137	0,957	0,814	0,000226681	25
Tulp4	1,26863E-08	0,788469903	0,971	0,828	0,000227084	25
Elavl3	1,38627E-08	0,663630861	0,986	0,902	0,000248143	25
Neat1	1,46235E-08	1,014567095	0,529	0,313	0,000261761	25
mt-Nd2	1,48219E-08	0,420540252	1	1	0,000265313	25
Fus	1,49659E-08	0,712929984	0,986	0,941	0,000267891	25
mt-Nd3	1,70519E-08	0,516187017	1	0,999	0,000305229	25
Arhgef28	1,97928E-08	0,699625165	0,843	0,731	0,000354292	25
Sphkap	2,13572E-08	0,893609023	0,5	0,28	0,000382295	25
Rassf8	2,51842E-08	0,611532054	0,271	0,091	0,000450797	25
Luc7l2	2,57277E-08	0,683934946	0,971	0,809	0,000460525	25
Tshz2	2,77227E-08	0,954785255	0,957	0,845	0,000496237	25
Tnrc6a	3,50515E-08	0,669246273	1	0,855	0,000627421	25
Hoxa5	3,60539E-08	0,597974578	0,986	0,931	0,000645364	25
Pcdh7	3,96547E-08	0,922745818	0,614	0,387	0,00070982	25
Myt1l	4,06004E-08	0,6897658	0,786	0,567	0,000726746	25
Plxna2	4,37018E-08	0,869692824	0,414	0,207	0,000782262	25
Igf1r	4,51599E-08	0,726126542	0,814	0,63	0,000808362	25
Ogt	5,10083E-08	0,612302443	0,843	0,65	0,000913049	25
Elavl4	5,51181E-08	0,625947783	1	0,985	0,000986614	25

Chd5	5,51321E-08	0,608671693	1	0,949	0,000986864	25
Mical3	5,58623E-08	0,760433531	0,514	0,314	0,000999935	25
Fem1b	6,21718E-08	0,675699678	0,843	0,6	0,001112875	25
Ank2	1,51112E-07	0,531455856	1	0,984	0,002704896	25
H2-Aa	0	3,321291412	0,533	0,006	0	26
H2-Eb1	0	2,939924111	0,533	0,008	0	26
Cd52	0	2,798638673	0,783	0,001	0	26
Arhgdib	0	1,75341877	0,717	0,002	0	26
Laptm5	0	1,367861194	0,567	0,001	0	26
Rac2	0	1,330903931	0,55	0	0	26
Lcp1	0	1,321630028	0,55	0,006	0	26
Ptprc	0	1,177523761	0,583	0,002	0	26
Srgn	0	1,062628384	0,55	0,003	0	26
Gmfg	0	0,917783247	0,417	0,004	0	26
Samsn1	0	0,87294407	0,333	0,001	0	26
Gpr183	0	0,845862291	0,283	0,001	0	26
Cd53	0	0,807354922	0,417	0	0	26
Cd79b	0	0,8064397	0,4	0,001	0	26
Hcls1	0	0,6623548	0,367	0	0	26
Ms4a1	0	0,616518783	0,25	0	0	26
Inpp5d	0	0,534832733	0,283	0,001	0	26
Arhgap30	0	0,37805394	0,25	0	0	26
Cd74	6,3868E-306	4,652978825	0,567	0,01	1,1432E-301	26
H2-Ab1	5,5289E-303	3,430451245	0,567	0,01	9,8967E-299	26
Il2rg	1,473E-302	0,582827885	0,267	0,001	2,6367E-298	26
Napsa	2,0711E-292	0,675937289	0,267	0,001	3,7072E-288	26
Hcst	1,1414E-285	0,566860587	0,25	0,001	2,0431E-281	26
Ptprcap	1,6283E-284	0,703401044	0,333	0,003	2,9147E-280	26
Cd79a	1,0434E-262	1,627999697	0,433	0,007	1,8677E-258	26
Gimap4	5,103E-250	0,67395797	0,267	0,002	9,1344E-246	26
Cd37	1,4887E-213	0,549340283	0,25	0,002	2,6647E-209	26
Gimap9	3,3891E-213	0,356542965	0,25	0,002	6,0665E-209	26
Gimap6	2,8224E-191	0,6535356	0,317	0,005	5,052E-187	26
AI662270	3,7652E-187	0,532091124	0,25	0,003	6,7397E-183	26
Ptpn6	2,6404E-181	0,462970237	0,267	0,003	4,7263E-177	26
Ctss	6,114E-146	0,869179577	0,3	0,006	1,0944E-141	26
Ltb	1,5507E-135	0,820757513	0,35	0,009	2,7758E-131	26
Cnn2	1,3049E-115	0,871461418	0,367	0,012	2,3358E-111	26
Ptpn18	2,0335E-114	1,249302482	0,433	0,018	3,64E-110	26
Tnfaip8	1,9507E-113	0,673321273	0,333	0,01	3,4917E-109	26
Ly6d	6,9951E-107	1,126407569	0,267	0,007	1,2521E-102	26
Cyba	1,1311E-106	1,42374657	0,5	0,027	2,0246E-102	26
4930523C07Rik	6,4107E-103	0,489588532	0,283	0,008	1,14752E-98	26
Gimap1	4,23501E-95	0,691282577	0,283	0,009	7,58068E-91	26
Hmha1	3,17785E-85	0,783295577	0,35	0,016	5,68835E-81	26
Selplg	5,00869E-80	0,85552092	0,3	0,012	8,96555E-76	26

AW112010	5,49629E-69	1,126461808	0,267	0,011	9,83836E-65	26
Zfp36l1	4,16932E-66	1,141663708	0,433	0,032	7,46309E-62	26
Lyn	4,36618E-66	0,824981489	0,367	0,023	7,81547E-62	26
Arhgap15	1,23564E-54	0,621090111	0,283	0,017	2,2118E-50	26
Ccnd2	1,01076E-53	1,515264246	0,483	0,048	1,80926E-49	26
Stk17b	6,37398E-50	0,701546387	0,283	0,018	1,14094E-45	26
Rpl17	2,8137E-40	2,636347278	1	0,919	5,03653E-36	26
Rpl37a	4,12442E-40	2,638589164	1	0,987	7,38271E-36	26
Rps29	4,62635E-40	2,817556211	1	0,996	8,28116E-36	26
Rpsa	7,44508E-40	2,394328738	1	0,991	1,33267E-35	26
Rpl8	8,10764E-40	2,105750656	1	0,971	1,45127E-35	26
Tpt1	9,68817E-40	2,447165075	1	0,959	1,73418E-35	26
Rps19	1,46044E-39	2,588080481	1	0,947	2,61419E-35	26
Rps8	2,41496E-39	2,580418462	1	0,978	4,32277E-35	26
Rps16	2,49218E-39	2,630384129	1	0,927	4,46101E-35	26
Rps10	4,04785E-39	2,417627453	1	0,924	7,24565E-35	26
Rpl32	4,55739E-39	2,485088545	1	0,968	8,15773E-35	26
Rps20	4,9845E-39	2,659660367	1	0,905	8,92226E-35	26
Rps27	5,20908E-39	3,29638379	1	0,874	9,32426E-35	26
Gnb2l1	6,99407E-39	2,403745226	1	0,818	1,25194E-34	26
Rpl13a	7,47002E-39	2,045742081	1	0,988	1,33713E-34	26
Rpl38	8,84139E-39	2,142882567	1	0,993	1,58261E-34	26
Rpl12	9,31947E-39	2,68427417	1	0,821	1,66819E-34	26
Rpl23	1,17848E-38	2,239741395	1	0,992	2,10948E-34	26
Rps13	2,53182E-38	2,653764121	1	0,834	4,53195E-34	26
Rpl27a	3,29489E-38	2,181526256	1	0,964	5,89785E-34	26
Rps18	3,66888E-38	2,462534136	1	0,936	6,56729E-34	26
Rps3	4,08645E-38	2,405675678	1	0,874	7,31474E-34	26
Rpl35a	5,15539E-38	2,429129393	1	0,926	9,22815E-34	26
Rps15a	5,39979E-38	2,883550753	1	0,892	9,66563E-34	26
Rps9	5,71974E-38	2,240048829	1	0,922	1,02383E-33	26
Rpl13	2,08375E-37	2,320238467	1	0,957	3,72991E-33	26
Rpl36	2,30329E-37	1,913719395	1	0,948	4,12288E-33	26
Rps24	3,23889E-37	2,68930323	1	0,967	5,79761E-33	26
Rps3a1	4,31628E-37	2,455137332	1	0,844	7,72615E-33	26
Rpl34	4,79245E-37	2,512511051	1	0,921	8,57848E-33	26
Rpl19	5,3175E-37	2,077049546	1	0,934	9,51832E-33	26
Rpl18a	5,38801E-37	2,346511135	1	0,977	9,64455E-33	26
Rplp2	8,22788E-37	2,07013653	1	0,934	1,47279E-32	26
Coro1a	9,4856E-37	2,382889382	0,833	0,312	1,69792E-32	26
Rps23	1,1439E-36	2,093048476	1	0,908	2,04758E-32	26
Rps14	1,26426E-36	2,312102653	1	0,954	2,26303E-32	26
Rps4x	1,3375E-36	2,291293505	1	0,891	2,39413E-32	26
Rps27a	1,4367E-36	2,158424154	1	0,932	2,5717E-32	26
Rpl11	3,16772E-36	1,894209846	1	0,954	5,67023E-32	26
Rpl28	3,18518E-36	2,163067315	1	0,965	5,70147E-32	26

Rpl39	3,48793E-36	2,691630661	1	0,938	6,2434E-32	26
Rpl30	6,42686E-36	2,324236048	1	0,828	1,15041E-31	26
Rps12	6,67077E-36	2,347885776	1	0,84	1,19407E-31	26
Rps26	8,85731E-36	2,230290586	1	0,921	1,58546E-31	26
Eef1a1	1,04962E-35	1,765785894	1	0,999	1,87882E-31	26
Rpl5	1,80477E-35	2,170526809	1	0,865	3,23054E-31	26
Rpl21	2,53714E-35	1,810349864	1	0,968	4,54147E-31	26
Rps5	2,57476E-35	2,301741894	1	0,891	4,60882E-31	26
Rpl35	3,47075E-35	2,252210056	1	0,84	6,21265E-31	26
Rpl41	3,83853E-35	1,775054387	1	0,998	6,87098E-31	26
H2-Q7	5,32062E-35	0,890872877	0,3	0,029	9,52392E-31	26
Rpl37	6,11735E-35	2,106089771	1	0,986	1,09501E-30	26
Rpl22	6,52791E-35	2,386961287	1	0,701	1,1685E-30	26
A330069E16Rik	3,5648E-156	3,743329971	1	0,064	6,3809E-152	27
Igfbp7	1,88949E-16	0,732350913	0,957	0,407	3,38219E-12	27
Fxyd5	1,24695E-15	0,973567079	0,915	0,429	2,23203E-11	27
Calm2	2,04243E-15	0,709598355	1	1	3,65594E-11	27
Cox7c	4,24528E-15	0,921569894	1	0,959	7,59905E-11	27
Nfe2l2	1,05246E-14	0,716939915	0,787	0,296	1,8839E-10	27
Tgfb1	1,27241E-14	0,702554122	0,872	0,338	2,27762E-10	27
Vip	4,1446E-14	1,315949794	1	0,926	7,41884E-10	27
Cd9	4,71099E-14	0,909611668	1	0,985	8,43267E-10	27
Basp1	7,70972E-14	0,63787943	1	0,983	1,38004E-09	27
Nsg1	9,50657E-14	0,779043557	1	0,972	1,70168E-09	27
Gapdh	1,04362E-13	0,887412659	1	0,931	1,86808E-09	27
S100a4	1,18751E-13	1,033222577	1	0,747	2,12565E-09	27
Tuba1a	1,30425E-13	0,903939731	1	0,986	2,3346E-09	27
Scgn	4,47933E-13	1,154118935	1	0,858	8,018E-09	27
Id3	8,39608E-13	0,725198122	0,957	0,471	1,5029E-08	27
Bglap	4,78495E-12	0,662600852	0,638	0,22	8,56506E-08	27
Ndufs5	7,0099E-12	0,696221968	1	0,856	1,25477E-07	27
Nme1	1,0134E-11	0,795549298	0,936	0,675	1,81399E-07	27
Cidea	2,14152E-11	0,701264514	1	0,756	3,83332E-07	27
Dstn	2,52963E-11	0,760173494	1	0,951	4,52803E-07	27
Lgals1	3,17286E-11	0,865552194	1	0,906	5,67942E-07	27
Crip1	3,33134E-11	0,767438851	1	0,986	5,9631E-07	27
Sec61g	6,21503E-11	0,702193424	1	0,877	1,11249E-06	27
Rpl14	6,80934E-11	0,628475512	1	0,982	1,21887E-06	27
Gm13889	8,29444E-11	0,674433021	0,851	0,389	1,48471E-06	27
Rpl35	1,13087E-10	0,720070742	1	0,84	2,02426E-06	27
Ubb	1,25885E-10	0,764746717	1	0,987	2,25334E-06	27
Mrpl20	1,35066E-10	0,63310291	0,872	0,466	2,41769E-06	27
S100a6	1,3914E-10	0,685409076	1	0,994	2,4906E-06	27
Atpif1	1,70585E-10	0,713981285	1	0,951	3,05348E-06	27
Tubb4b	1,91664E-10	0,653679361	1	0,791	3,43078E-06	27
Sh3bgrl3	1,93689E-10	0,726534148	0,957	0,639	3,46704E-06	27

Tmsb4x	1,99806E-10	0,504926563	1	0,998	3,57653E-06	27
Rprml	2,04028E-10	0,51590804	0,511	0,164	3,6521E-06	27
Cox4i1	2,86062E-10	0,702987079	1	0,893	5,12052E-06	27
Rps21	2,94815E-10	0,774542745	1	0,952	5,27719E-06	27
Tmem256	3,0368E-10	0,750921014	0,915	0,545	5,43587E-06	27
Rpl35a	3,0854E-10	0,671178924	1	0,926	5,52287E-06	27
Rps13	3,19447E-10	0,635802033	1	0,834	5,71809E-06	27
Dync1i2	3,21527E-10	0,551750473	1	0,998	5,75533E-06	27
Atp5e	3,36777E-10	0,723337469	1	0,852	6,02831E-06	27
Uchl1	4,51072E-10	0,515251275	1	0,991	8,07418E-06	27
Rpl19	4,77433E-10	0,696091944	1	0,934	8,54605E-06	27
Uqcr11	4,95E-10	0,699205453	1	0,835	8,8605E-06	27
Hint1	6,06651E-10	0,703255705	1	0,885	1,08591E-05	27
Cetn2	6,18027E-10	0,74369112	0,957	0,705	1,10627E-05	27
Rps23	8,10073E-10	0,611516636	1	0,908	1,45003E-05	27
Cox7a2l	8,20854E-10	0,721452711	0,957	0,647	1,46933E-05	27
Rpl39	8,68585E-10	0,714752206	1	0,938	1,55477E-05	27
Ttc9b	8,73541E-10	0,659694731	0,872	0,578	1,56364E-05	27
Alpl	9,73326E-10	0,419919444	0,617	0,224	1,74225E-05	27
Cox6b1	9,78838E-10	0,664964298	1	0,875	1,75212E-05	27
Arl3	1,07048E-09	0,606921457	0,872	0,482	1,91616E-05	27
Stmn4	1,1365E-09	0,631887326	0,83	0,435	2,03433E-05	27
Dnlz	1,14144E-09	0,402557561	0,766	0,314	2,04318E-05	27
Gm10076	1,29177E-09	0,765746037	1	0,856	2,31226E-05	27
Plpp1	1,55736E-09	0,62329983	0,915	0,486	2,78768E-05	27
Npy	1,58607E-09	0,911075426	0,979	0,848	2,83907E-05	27
Rps24	1,65328E-09	0,594335392	1	0,967	2,95938E-05	27
Atp5j2	1,67704E-09	0,694823217	1	0,806	3,00191E-05	27
Resp18	1,81285E-09	0,793949091	1	0,927	3,24501E-05	27
Cuedc2	1,93313E-09	0,612451912	0,936	0,541	3,4603E-05	27
Cntnap5a	1,97601E-09	0,684733709	0,979	0,662	3,53705E-05	27
Acyp2	1,99985E-09	0,663373454	0,936	0,554	3,57974E-05	27
Csrp1	2,06314E-09	0,575728096	1	0,954	3,69301E-05	27
Uqcrh	2,29209E-09	0,710476698	0,957	0,783	4,10284E-05	27
Rps27a	2,30889E-09	0,637956579	1	0,932	4,13291E-05	27
Tarbp2	2,40737E-09	0,320131974	0,426	0,127	4,30919E-05	27
Cox7a2	2,45424E-09	0,656259519	1	0,857	4,39309E-05	27
Cox8a	2,4702E-09	0,570340712	1	0,934	4,42165E-05	27
Ndufb11	2,91603E-09	0,615430191	0,979	0,637	5,21969E-05	27
Nars	3,17413E-09	0,609042742	0,979	0,807	5,68169E-05	27
Ncoa7	3,21546E-09	0,759819268	0,957	0,785	5,75568E-05	27
Cox7b	3,30847E-09	0,597831331	1	0,846	5,92216E-05	27
Mpc1	3,32098E-09	0,659695234	0,936	0,827	5,94455E-05	27
Ndufc1	3,52987E-09	0,654145003	0,979	0,826	6,31846E-05	27
Etv1	3,64048E-09	0,755153522	1	0,61	6,51646E-05	27
Nmt2	3,65996E-09	0,53779706	0,979	0,805	6,55133E-05	27

Uqcrq	4,69088E-09	0,618390819	1	0,865	8,39668E-05	27
Rpl22l1	5,30295E-09	0,671859837	0,979	0,789	9,49228E-05	27
Rps7	5,31046E-09	0,642087507	0,979	0,798	9,50573E-05	27
Psmg4	5,89425E-09	0,485293797	0,83	0,396	0,000105507	27
Dbh	6,2904E-09	0,589963751	0,915	0,659	0,000112598	27
Atp5f1	6,74954E-09	0,594754675	0,957	0,692	0,000120817	27
Ndufa3	8,08843E-09	0,64102798	1	0,783	0,000144783	27
Gm17619	9,16276E-09	0,280498547	0,255	0,058	0,000164013	27
Nop10	9,42068E-09	0,534153235	0,979	0,576	0,00016863	27
Lst1	9,73891E-09	0,61802306	0,83	0,477	0,000174327	27
Saysd1	1,06837E-08	0,375710211	0,489	0,172	0,000191238	27
Sat1	1,10101E-08	0,520985622	0,787	0,406	0,000197081	27
Tmsb10	1,11946E-08	0,599501494	1	0,949	0,000200384	27
Gfra1	1,13219E-08	0,617355556	0,915	0,47	0,000202661	27
Rpl37	1,15387E-08	0,596265516	1	0,986	0,000206542	27
Gstm5	1,19561E-08	0,630090938	0,83	0,49	0,000214015	27
Tmem141	1,3585E-08	0,291078585	0,362	0,104	0,000243171	27
Btf3	1,38815E-08	0,580019763	1	0,769	0,000248478	27
Rpl7a	1,42169E-08	0,505512304	1	0,834	0,000254482	27
1110004F10Rik	1,42266E-08	0,58254163	1	0,928	0,000254656	27
Ndufa4	1,56857E-08	0,637323757	1	0,845	0,000280774	27
Fau	1,7419E-08	0,59380832	1	0,926	0,0003118	27
Fabp4	0	4,289410137	0,824	0,004	0	28
Sdpr	0	3,463662033	0,971	0,012	0	28
Mmrn1	0	3,040907463	0,265	0,001	0	28
Cav1	0	3,038013447	0,882	0,003	0	28
Cldn5	0	2,478897165	0,735	0,001	0	28
Mecom	0	2,241167744	0,618	0,006	0	28
Cd36	0	2,169164367	0,735	0,003	0	28
Cdh5	0	2,140443136	0,853	0,001	0	28
Adgrf5	0	2,050961001	0,735	0,009	0	28
Madcam1	0	2,041059541	0,294	0,001	0	28
Emcn	0	1,8047704	0,735	0,002	0	28
Tnfaip2	0	1,679512954	0,559	0,005	0	28
Podxl	0	1,677377688	0,647	0,002	0	28
S1pr1	0	1,608267396	0,647	0,001	0	28
Cd93	0	1,475561452	0,618	0,004	0	28
Ecscr	0	1,449567204	0,647	0,001	0	28
Tie1	0	1,268568296	0,647	0,001	0	28
Sox18	0	1,268112348	0,647	0,001	0	28
Entpd1	0	1,194443776	0,5	0,002	0	28
Dab2	0	1,077601016	0,441	0,003	0	28
Flt4	0	1,040451323	0,353	0,001	0	28
Slfn5	0	1,038780042	0,471	0,002	0	28
Tek	0	0,998023184	0,588	0,001	0	28
She	0	0,956018565	0,529	0,001	0	28

Gngt2	0	0,952221797	0,5	0,003	0	28
Mall	0	0,938335109	0,588	0,006	0	28
C130074G19Rik	0	0,928831097	0,529	0,003	0	28
Ptpn14	0	0,88662511	0,412	0,002	0	28
Sox17	0	0,843274496	0,265	0	0	28
Robo4	0	0,818971326	0,471	0	0	28
Grrp1	0	0,770213852	0,441	0	0	28
Gimap4	0	0,739353298	0,441	0,002	0	28
Bmx	0	0,719587779	0,412	0	0	28
Prkch	0	0,719131447	0,471	0,001	0	28
Erg	0	0,69359257	0,294	0	0	28
Adgrl4	0	0,667120359	0,324	0	0	28
Fgd5	0	0,638176912	0,353	0,002	0	28
Gata2	0	0,554112648	0,353	0,001	0	28
Unc45b	0	0,527094844	0,265	0	0	28
Adcy4	0	0,526486369	0,294	0,001	0	28
Sult1a1	4,697E-306	0,611000061	0,324	0,001	8,4076E-302	28
Ccm2l	1,0346E-303	0,434578247	0,294	0,001	1,852E-299	28
Rasgrp3	1,0349E-303	0,465453144	0,294	0,001	1,8524E-299	28
Myct1	1,1811E-303	0,465453144	0,294	0,001	2,1141E-299	28
Flt1	1,0035E-296	2,418228772	0,588	0,007	1,7963E-292	28
Epas1	2,4517E-295	2,374605629	0,706	0,01	4,3886E-291	28
Pecam1	1,2002E-291	2,538546977	0,941	0,02	2,1484E-287	28
Reln	5,03E-290	1,830183574	0,265	0,001	9,0038E-286	28
Lrg1	1,8139E-289	0,932928156	0,294	0,001	3,2469E-285	28
Fli1	3,4514E-289	0,434426248	0,294	0,001	6,1781E-285	28
Arap3	7,1971E-284	0,68873245	0,441	0,003	1,2883E-279	28
Pear1	3,3579E-277	0,811990774	0,5	0,005	6,0107E-273	28
Rassf9	2,9918E-273	0,433210835	0,265	0,001	5,3554E-269	28
Kdr	7,9118E-261	2,053812326	0,706	0,012	1,4162E-256	28
Lmo2	1,1186E-257	1,03181195	0,529	0,006	2,0023E-253	28
Hoxd3os1	6,5355E-256	0,740262751	0,382	0,003	1,1699E-251	28
Gatsl3	7,2331E-253	0,433362706	0,324	0,002	1,2947E-248	28
Ackr3	1,2691E-247	0,884501045	0,412	0,004	2,2717E-243	28
Ushbp1	7,9225E-243	0,638024994	0,294	0,002	1,4181E-238	28
Gja1	1,6169E-236	0,860053611	0,324	0,002	2,8942E-232	28
Gja4	1,0942E-233	1,119406002	0,294	0,002	1,9587E-229	28
Tgfbr2	4,5717E-233	1,817651093	0,735	0,015	8,1834E-229	28
Hhex	9,6061E-229	0,575861251	0,324	0,002	1,7195E-224	28
Apold1	8,2117E-227	1,112738072	0,5	0,006	1,4699E-222	28
Gbp4	2,2703E-223	0,58025302	0,353	0,003	4,0638E-219	28
Stab1	1,5129E-221	1,716675923	0,588	0,009	2,7081E-217	28
Esam	1,0587E-210	1,73119392	0,559	0,009	1,8951E-206	28
Heg1	5,9642E-206	1,561717068	0,588	0,01	1,0676E-201	28
Thbd	2,8724E-205	0,781248333	0,441	0,005	5,1416E-201	28
Srgn	6,5312E-197	0,965900615	0,412	0,005	1,1691E-192	28

Meox1	2,9376E-192	0,491274265	0,382	0,004	5,2582E-188	28
Cysltr1	3,6066E-189	0,637417479	0,265	0,002	6,4559E-185	28
Gimap6	4,5253E-185	0,902377826	0,412	0,005	8,1003E-181	28
Ace	6,8616E-182	1,503552436	0,412	0,005	1,2282E-177	28
Nkx2-3	1,9666E-179	1,515357935	0,471	0,007	3,5202E-175	28
Fgl2	7,7052E-177	2,538398177	0,529	0,01	1,3792E-172	28
Klf2	2,5074E-176	2,665979798	0,824	0,026	4,4882E-172	28
Prss23	3,8598E-173	2,231813427	0,647	0,016	6,909E-169	28
Icam1	1,0243E-172	0,431237971	0,294	0,003	1,8335E-168	28
Lyve1	6,5527E-170	2,508195605	0,265	0,002	1,1729E-165	28
Ly6a	3,0025E-166	2,262701135	0,647	0,016	5,3745E-162	28
Gimap5	3,4662E-166	0,550167954	0,324	0,003	6,2045E-162	28
Rhoj	4,8775E-166	1,366073682	0,529	0,011	8,7308E-162	28
Lrrc32	5,8684E-159	0,787137817	0,324	0,004	1,0504E-154	28
Prelp	1,0014E-155	1,225968971	0,353	0,005	1,7925E-151	28
Igfbp3	6,6749E-150	1,494454829	0,382	0,006	1,1948E-145	28
Mmrn2	1,9296E-149	1,985650766	0,824	0,032	3,4539E-145	28
Vegfc	6,5638E-149	0,660896274	0,324	0,004	1,1749E-144	28
Slfn2	8,0377E-149	0,57994971	0,265	0,003	1,4388E-144	28
Tmem88	5,5729E-147	1,704537882	0,647	0,019	9,9756E-143	28
Megf6	4,2947E-142	0,547140896	0,353	0,005	7,6875E-138	28
Meis2	2,1112E-139	0,864647437	0,471	0,01	3,7791E-135	28
Arhgdib	6,0833E-137	0,77238405	0,353	0,005	1,0889E-132	28
Plvap	1,1563E-136	3,591459421	0,676	0,023	2,0697E-132	28
Gpr182	1,6018E-136	0,522992607	0,265	0,003	2,8672E-132	28
Hspa12b	2,8978E-136	0,431844719	0,265	0,003	5,1871E-132	28
Tmem252	7,1003E-136	1,189745673	0,324	0,005	1,2709E-131	28
Lbp	3,38E-133	1,162531275	0,441	0,009	6,0503E-129	28
Gng11	1,124E-126	2,722983485	0,824	0,038	2,012E-122	28
Jam2	6,1661E-124	0,810175302	0,353	0,006	1,1037E-119	28
Acvrl1	1,1863E-121	1,359447517	0,5	0,013	2,1234E-117	28
Syt15	4,4832E-104	1,708386577	1	0,057	8,025E-100	29
Emilin2	1,28508E-91	0,817879159	0,483	0,014	2,30029E-87	29
Cdkn1c	5,21353E-85	2,76837587	0,966	0,071	9,33222E-81	29
Slc35d3	1,25322E-83	1,65538468	0,793	0,045	2,24325E-79	29
Otof	4,76171E-75	1,117276931	0,724	0,04	8,52346E-71	29
Htr3b	3,32428E-72	1,738205563	0,931	0,077	5,95047E-68	29
Phgdh	8,90976E-72	2,8606993	1	0,094	1,59485E-67	29
Abhd11os	7,89113E-60	1,553253958	0,793	0,064	1,41251E-55	29
Nog	1,11741E-58	2,238574813	1	0,114	2,00017E-54	29
Adgrg6	2,1407E-58	2,383418812	0,966	0,098	3,83185E-54	29
Ptger3	7,42391E-57	0,721189517	0,552	0,03	1,32888E-52	29
Tmeff2	4,88368E-53	1,127767765	0,724	0,057	8,74178E-49	29
Pdpn	1,10257E-52	0,89233542	0,724	0,057	1,97359E-48	29
Ada	9,07326E-52	0,510504645	0,379	0,016	1,62411E-47	29
Sgcz	1,17618E-51	0,6557668	0,552	0,033	2,10535E-47	29

Avil	1,21264E-50	1,842164236	0,966	0,12	2,17062E-46	29
Nmu	2,60505E-49	2,55249458	0,483	0,028	4,66304E-45	29
Islr2	4,14667E-47	1,657963585	0,931	0,114	7,42255E-43	29
Slc25a48	5,42342E-46	0,97145754	0,621	0,048	9,70792E-42	29
Shf	2,18005E-45	0,980106491	0,724	0,066	3,9023E-41	29
Cbln2	2,58014E-42	2,181422183	1	0,154	4,61845E-38	29
Id4	9,17245E-42	1,888880852	1	0,161	1,64187E-37	29
Tpm2	4,70073E-40	1,320730908	0,517	0,039	8,41431E-36	29
Camp	1,00027E-38	1,45439565	0,828	0,108	1,79049E-34	29
Cdc42ep5	2,34488E-37	0,714370995	0,517	0,041	4,19733E-33	29
Ifi27l2a	2,83898E-37	1,485995809	0,517	0,041	5,08177E-33	29
Wif1	3,38893E-35	1,450006677	0,862	0,133	6,06619E-31	29
Dapk2	7,92524E-35	1,184747215	0,759	0,1	1,41862E-30	29
Pcdh10	1,92249E-34	2,596490991	1	0,207	3,44126E-30	29
Dgat2	9,83082E-32	0,917361779	0,759	0,105	1,75972E-27	29
Dgkg	4,82438E-31	1,472659196	0,931	0,174	8,63565E-27	29
Edn1	5,25647E-31	1,884888751	1	0,222	9,40907E-27	29
Krt15	2,73915E-30	0,924179266	0,655	0,083	4,90308E-26	29
Cysltr2	7,71519E-29	1,393289756	0,862	0,164	1,38102E-24	29
6430573F11Rik	6,25291E-28	0,501259436	0,31	0,02	1,11927E-23	29
Pkp1	6,84686E-28	1,124134751	0,69	0,099	1,22559E-23	29
Prom1	5,62314E-27	0,915009301	0,724	0,109	1,00654E-22	29
Krt19	2,40128E-26	3,202905219	1	0,362	4,29829E-22	29
Hey1	5,17043E-26	0,958250287	0,724	0,119	9,25508E-22	29
Bcl11a	9,33548E-26	0,667377299	0,586	0,073	1,67105E-21	29
Hpcal1	1,7833E-25	1,679371059	0,966	0,278	3,19211E-21	29
Hes1	2,36162E-25	1,195638739	0,724	0,113	4,2273E-21	29
Pdlim2	2,65797E-25	2,081759044	0,966	0,291	4,75776E-21	29
Kctd12	2,68576E-24	0,688138031	0,586	0,074	4,8075E-20	29
Rab3b	3,13527E-23	2,32192009	1	0,459	5,61214E-19	29
C130060K24Rik	3,49989E-23	0,599441832	0,379	0,035	6,2648E-19	29
Gm3636	3,86258E-23	0,321222297	0,276	0,019	6,91402E-19	29
Gm20754	5,11105E-23	0,459796039	0,448	0,048	9,14877E-19	29
Serpinf1	6,06192E-23	1,382333334	0,931	0,239	1,08508E-18	29
Tbx2	6,96735E-23	1,731551338	0,966	0,303	1,24715E-18	29
Fbxo2	1,31714E-22	1,215189129	0,793	0,164	2,35767E-18	29
Rnaseh2b	2,08895E-22	0,937797382	0,793	0,162	3,73923E-18	29
Tnnt1	2,28495E-22	0,528977377	0,31	0,025	4,09005E-18	29
Nt5dc2	2,47217E-22	1,567949706	0,931	0,288	4,42518E-18	29
Serinc2	8,67028E-22	0,589597011	0,379	0,038	1,55198E-17	29
Mt3	2,99748E-21	2,877757005	1	0,718	5,36548E-17	29
Zfp804a	3,32023E-21	1,52742239	0,931	0,252	5,94321E-17	29
Hspb1	7,95585E-21	2,001700004	0,897	0,293	1,4241E-16	29
Ptgfr	9,98533E-21	1,024120727	0,793	0,171	1,78737E-16	29
Pde2a	1,13998E-20	1,443210266	0,897	0,239	2,04057E-16	29
Iqgap2	1,23892E-20	0,761275008	0,69	0,122	2,21767E-16	29

Fam129a	1,26449E-20	0,550876083	0,448	0,053	2,26343E-16	29
S100a11	3,99614E-20	2,087732735	1	0,85	7,1531E-16	29
Bmp4	8,40275E-20	0,99382267	0,724	0,145	1,50409E-15	29
Myl1	1,35147E-19	1,931450907	1	0,913	2,41913E-15	29
Cdh8	1,85357E-19	0,452589129	0,31	0,029	3,3179E-15	29
Fndc5	2,53369E-19	0,502144631	0,379	0,042	4,53531E-15	29
Serpina3n	3,03807E-19	0,696317794	0,621	0,108	5,43814E-15	29
Rims1	3,64055E-19	0,714739452	0,655	0,121	6,51659E-15	29
Rgs6	3,81331E-19	0,43619469	0,345	0,035	6,82582E-15	29
Calb2	4,70622E-19	1,779473673	1	0,969	8,42413E-15	29
Dclk3	4,9111E-19	0,684036661	0,586	0,098	8,79087E-15	29
Tmsb10	5,30856E-19	1,645357442	1	0,949	9,50232E-15	29
Proser2	8,10099E-19	0,822093861	0,69	0,136	1,45008E-14	29
Prph	9,26065E-19	1,35502879	1	0,964	1,65766E-14	29
Serpinb6a	1,11242E-18	1,519104958	1	0,838	1,99123E-14	29
Gpr85	1,15799E-18	1,31885162	0,931	0,333	2,07281E-14	29
Cyp26b1	1,43745E-18	0,70119784	0,414	0,05	2,57304E-14	29
A330102I10Rik	1,44865E-18	0,4969243	0,379	0,044	2,59309E-14	29
Layn	2,88118E-18	0,573701449	0,345	0,038	5,1573E-14	29
Rps29	3,66149E-18	1,478328708	1	0,996	6,55407E-14	29
Aldh2	4,1385E-18	1,320283085	0,931	0,334	7,40792E-14	29
Crybb2	4,20326E-18	0,463266491	0,379	0,044	7,52383E-14	29
S100a10	5,38257E-18	1,324044026	1	0,978	9,6348E-14	29
Psd3	7,67891E-18	1,594904042	0,828	0,259	1,37452E-13	29
Dleu7	2,83609E-17	0,964151069	0,621	0,126	5,0766E-13	29
Mfge8	3,73106E-17	0,305761737	0,31	0,031	6,6786E-13	29
Fez1	5,84364E-17	1,556830643	1	0,786	1,04601E-12	29
Tbc1d1	2,11622E-16	0,517122383	0,379	0,049	3,78804E-12	29
Arhgap6	2,22478E-16	0,791229131	0,621	0,127	3,98236E-12	29
Gpsm3	3,49993E-16	1,137849563	0,793	0,22	6,26488E-12	29
Serpine2	4,44577E-16	1,710975317	0,897	0,328	7,95793E-12	29
Fth1	7,0516E-16	1,278515728	1	0,994	1,26224E-11	29
Anxa2	9,13807E-16	1,168265169	1	0,922	1,63571E-11	29
Gsta4	1,23294E-15	0,42027234	0,345	0,042	2,20696E-11	29
Cpne4	1,23428E-15	1,313307401	1	0,797	2,20936E-11	29
Stmn3	1,51953E-15	1,299677276	1	0,916	2,71996E-11	29
Adra2a	1,95309E-15	1,053457375	0,828	0,256	3,49604E-11	29
Ntan1	3,1955E-15	1,23885791	0,931	0,401	5,71994E-11	29
Medag	3,32239E-15	0,634458841	0,448	0,073	5,94707E-11	29
Lgals1	5,7501E-15	1,418184408	1	0,906	1,02927E-10	29
Gcg	0	8,080568987	0,75	0,005	0	30
Cck	0	5,176730102	0,917	0,011	0	30
Car8	0	3,006194102	0,917	0,008	0	30
Ace2	0	2,697812959	0,917	0,007	0	30
Ffar1	0	2,64006102	0,958	0,002	0	30
Slc15a1	0	2,582393086	0,917	0,007	0	30

Pax6	0	2,522783132	1	0,015	0	30
Naaladl1	0	2,514235075	0,958	0,006	0	30
Hoxb9	0	2,137344643	0,708	0,003	0	30
Arhgef38	0	1,951650022	0,875	0,008	0	30
Isl1	0	1,831771365	0,958	0,004	0	30
Cldn4	0	1,811067951	0,917	0,01	0	30
Tmem45b	0	1,602167485	0,917	0,008	0	30
Prox1	0	1,437108239	0,833	0,007	0	30
Arx	0	1,410939147	0,75	0,002	0	30
Gfra3	0	1,361804645	0,708	0,004	0	30
Sstr5	0	1,340767318	0,875	0,003	0	30
Cdx1	0	1,275656545	0,875	0,009	0	30
Gpr119	0	1,193511974	0,625	0,002	0	30
Ms4a10	0	0,984277295	0,625	0,005	0	30
Aqp4	0	0,934804285	0,5	0,002	0	30
Syndig1l	0	0,904612296	0,5	0,002	0	30
Myzap	0	0,897798905	0,625	0,005	0	30
Wnt3	0	0,867342437	0,625	0,004	0	30
Grpr	0	0,770462979	0,458	0,001	0	30
Klb	0	0,768491151	0,542	0,002	0	30
Capsl	0	0,69770619	0,5	0,002	0	30
Gpbar1	0	0,662357114	0,375	0	0	30
Fcna	0	0,661597601	0,292	0	0	30
Gprc5c	0	0,660990278	0,5	0,001	0	30
Gm29440	0	0,501588589	0,292	0,001	0	30
Scin	3,6281E-302	1,29945505	0,708	0,007	6,4943E-298	30
Cyp2c68	1,3276E-300	0,96174358	0,583	0,004	2,3765E-296	30
Nostrin	6,465E-279	1,882921755	0,833	0,011	1,1572E-274	30
Gm53	7,3895E-279	0,581773888	0,375	0,002	1,3227E-274	30
Cdhr2	2,8379E-278	2,208511494	0,958	0,015	5,0798E-274	30
Slc5a1	1,3216E-275	1,799845106	0,875	0,012	2,3656E-271	30
Sdcbp2	6,0926E-275	1,043020633	0,75	0,009	1,0906E-270	30
Gm609	1,0225E-260	1,881427508	0,958	0,016	1,8302E-256	30
Tnfrsf11b	6,1959E-246	1,102614167	0,542	0,005	1,1091E-241	30
Sult1d1	1,2348E-244	2,031598195	0,792	0,011	2,2103E-240	30
Krt20	2,9793E-240	3,61202597	1	0,019	5,3329E-236	30
Ms4a8a	5,05E-240	2,650101962	0,917	0,016	9,0394E-236	30
Guca2a	9,8368E-234	2,218698327	0,708	0,009	1,7608E-229	30
Itgb7	1,2283E-232	0,965224879	0,375	0,002	2,1987E-228	30
Malrd1	1,3235E-230	1,558603608	0,75	0,011	2,3691E-226	30
Hepacam2	1,3641E-228	2,195441691	0,958	0,018	2,4417E-224	30
Slc6a19	9,3337E-227	2,355000073	0,917	0,017	1,6707E-222	30
Amn	6,0293E-226	1,061999754	0,625	0,007	1,0792E-221	30
Slc16a11	7,947E-226	0,8278367	0,667	0,009	1,4225E-221	30
Pax6os1	5,0155E-222	0,413670088	0,25	0,001	8,9777E-218	30
Kif12	2,7372E-220	0,58010647	0,417	0,003	4,8996E-216	30

Rnf186	3,0168E-220	1,46690836	0,542	0,006	5,4002E-216	30
Smim6	2,2223E-218	1,316433936	0,75	0,011	3,9779E-214	30
Gcnt3	1,6793E-216	1,22650245	0,542	0,006	3,0059E-212	30
Cdhr5	6,4801E-216	2,324985283	0,958	0,02	1,1599E-211	30
Ugt2b34	4,3331E-215	1,37975101	0,708	0,01	7,7562E-211	30
Neurod1	5,9006E-214	3,359758571	1	0,022	1,0562E-209	30
Myl7	9,3006E-214	1,902006264	0,542	0,006	1,6648E-209	30
Tspan1	1,2579E-213	2,232204809	0,958	0,02	2,2517E-209	30
Gm15200	4,1871E-213	1,328849761	0,5	0,005	7,4948E-209	30
4930539E08Rik	5,0735E-213	0,977377306	0,625	0,008	9,0816E-209	30
Xpnpep2	9,5654E-210	0,950149559	0,583	0,007	1,7122E-205	30
Krt18	8,7448E-207	2,577886895	0,958	0,021	1,5653E-202	30
Hoxb8	5,5981E-206	1,883071265	0,75	0,012	1,0021E-201	30
Adh1	1,0353E-205	2,605300492	0,875	0,017	1,8532E-201	30
Gucy2c	1,0602E-202	2,228619926	0,875	0,018	1,8978E-198	30
Tm4sf20	1,7506E-202	2,151079793	0,917	0,019	3,1335E-198	30
Cdx2	5,0773E-198	1,468549474	0,75	0,013	9,0884E-194	30
Fxyd3	6,1484E-196	2,721714431	1	0,024	1,1006E-191	30
Gpa33	7,6388E-195	1,822779508	0,875	0,018	1,3673E-190	30
Prss30	5,6701E-192	0,828891247	0,458	0,005	1,0149E-187	30
Smim22	2,2641E-191	2,64569426	1	0,025	4,0527E-187	30
Eps8l3	1,8985E-190	1,317476938	0,708	0,012	3,3982E-186	30
Osr2	1,1238E-189	0,828891247	0,5	0,006	2,0117E-185	30
Tox3	2,7594E-186	1,012371637	0,583	0,008	4,9393E-182	30
H2-Q2	8,9189E-185	1,400750755	0,792	0,015	1,5965E-180	30
Gm12511	8,8468E-183	0,367410882	0,25	0,001	1,5836E-178	30
Clrn3	4,0546E-177	1,12408116	0,583	0,009	7,2577E-173	30
Fev	5,9641E-177	1,020939658	0,625	0,01	1,0676E-172	30
Hopx	1,1991E-174	1,695923669	0,958	0,025	2,1464E-170	30
Rassf9	8,1009E-173	0,734687294	0,25	0,001	1,4501E-168	30
Cdh17	1,1712E-172	2,720357714	0,958	0,025	2,0965E-168	30
Muc13	1,5001E-171	2,53079849	1	0,028	2,6852E-167	30
Fabp2	2,2789E-170	1,275706448	0,875	0,021	4,0793E-166	30
Fut2	2,4575E-169	0,536437746	0,375	0,003	4,3989E-165	30
Krt7	3,4997E-168	1,905599117	0,917	0,023	6,2645E-164	30
Prr15l	7,9348E-167	1,68689608	0,875	0,022	1,4203E-162	30
Bace2	1,9217E-163	1,699603924	0,833	0,02	3,4398E-159	30
Cdh1	1,9899E-160	1,610002403	0,875	0,022	3,562E-156	30
Pipox	3,9022E-159	0,613436837	0,458	0,006	6,9849E-155	30
Serpinb1a	1,1372E-158	1,935809405	0,917	0,025	2,0357E-154	30
Mlxipl	5,9299E-158	1,267743985	0,625	0,011	1,0614E-153	30
Fbp2	9,4033E-158	1,72800719	0,833	0,021	1,6832E-153	30
AW112010	3,734E-156	1,701679104	0,625	0,011	6,6839E-152	30
Tmem30b	5,0844E-156	0,852445115	0,583	0,01	9,1011E-152	30
S100a14	2,9084E-155	0,897345821	0,458	0,006	5,206E-151	30
Mctp2	9,6012E-155	0,760178496	0,458	0,006	1,7186E-150	30

Pyy	8,1878E-154	9,572045997	0,917	0,027	1,4656E-149	30
Galnt4	1,4213E-153	0,91358448	0,667	0,013	2,5442E-149	30
Insl5	3,1115E-151	3,645468563	0,292	0,002	5,5696E-147	30
