# Supplementary material for: The transcriptomes, connections and development of submucosal neuron classes in the mouse small intestine
Source: Nat Neurosci. 2025 May 29;28(6):1146–59. doi: 10.1038/s41593-025-01962-x (PMC12148937; doi:10.1038/s41593-025-01962-x)
Supplement: Supplementary file 9 — DE genes in smENC1–smENC3. [file 41593_2025_1962_MOESM9_ESM.pdf]

Supplementary Table 2. Enriched genes in cardinal smENC 1-3.

p-val: unadjusted p value; avg_logFC: log fold-change of the average expression between two groups. Positive values indicate high gene expression; pct.1: the percentage of cells where the gene is detected in the group; pct.2: The percentage of cells where the gene is detected in the rest of the dataset; p_val_adj: adjusted p-value based on Bonferroni correction using all genes in the dataset; Statistical analysis was two-sided but only enriched genes are shown. smENC: Enteric Neuron Class as defined in Figure 1.
Top 30 genes for each cluster are highlighted by their specific color (Fig. 1).

gene	p_val	avg_log2FC	pct.1	pct.2	p_val_ad	smENC
Pcdh10	0	3,300894631	0,99	0,17	0	1
Serpine2	0	3,201922934	0,966	0,292	0	1
Adgrg6	0	3,008911364	0,96	0,042	0	1
Cbln2	0	2,989402684	0,968	0,116	0	1
Pde2a	0	2,114370773	0,942	0,202	0	1
Edn1	0	2,100057751	0,946	0,193	0	1
Tbx2	0	1,903472751	0,924	0,287	0	1
Dgkg	0	1,902387226	0,893	0,139	0	1
Nog	0	1,868298447	0,845	0,079	0	1
Cdkn1c	0	1,510169801	0,674	0,028	0	1
Cyp26b1	0	1,497129679	0,584	0,021	0	1
Syt15	0	1,475959781	0,734	0,019	0	1
Otof	0	1,455627426	0,66	0,005	0	1
Islr2	0	1,424209183	0,74	0,084	0	1
Tmeff2	0	1,412762233	0,666	0,022	0	1
Slc35d3	0	1,357176211	0,674	0,005	0	1
Nmu	0	1,348760563	0,338	0,01	0	1
Pkp1	0	1,226007609	0,61	0,072	0	1
Kctd12	0	1,137817412	0,569	0,018	0	1
Phgdh	0	1,067828619	0,541	0,05	0	1
Bcl11a	0	0,983518812	0,563	0,042	0	1
Dapk2	0	0,964387942	0,598	0,077	0	1
Slc25a48	0	0,85551695	0,433	0,028	0	1
Sgcz	0	0,851663655	0,479	0,007	0	1
Ptger3	0	0,786975384	0,457	0,005	0	1
Rgs6	0	0,592742343	0,354	0,014	0	1
C130060K24Rik	0	0,590458497	0,358	0,019	0	1
Thsd7b	0	0,544513821	0,326	0,012	0	1
Gpr85	1,2639E-303	1,875160635	0,93	0,324	2E-299	1
Htr3b	2,3244E-289	0,845168533	0,509	0,056	4E-285	1
Sulf2	1,7805E-283	1,509768992	0,859	0,233	3E-279	1
Avil	1,1971E-272	1,159989568	0,61	0,094	2E-268	1
Nrxn3	2,5003E-270	1,770227202	0,978	0,431	4E-266	1
Zfp804a	3,4762E-264	1,613178874	0,827	0,239	6E-260	1
Efr3a	2,617E-252	1,999145327	0,924	0,431	4E-248	1
6330403A02Rik	2,587E-251	1,865553342	1	0,934	4E-247	1
Lhfpl2	2,1716E-249	1,787427839	0,954	0,481	4E-245	1

Ccbe1	3,5795E-249	1,142454652	0,706	0,151	6E-245	1
Casz1	2,7115E-246	1,584461973	0,934	0,349	5E-242	1
Ntrk3	1,0148E-241	1,7400423	1	0,7	2E-237	1
Tcf7l2	4,8941E-241	1,522827162	1	0,836	8E-237	1
Ptgfr	1,1363E-240	1,173567078	0,7	0,152	2E-236	1
Id4	1,2812E-238	1,149590252	0,662	0,137	2E-234	1
Cnr1	2,1765E-237	1,35490273	1	0,997	4E-233	1
Zeb2	1,684E-236	1,726808697	0,986	0,63	3E-232	1
Bmp4	1,9467E-228	0,997772216	0,614	0,117	3E-224	1
Cysltr2	7,9664E-227	1,081436884	0,672	0,15	1E-222	1
Shf	8,682E-224	0,56653094	0,382	0,039	1E-219	1
Esr1	8,1916E-218	0,407311624	0,264	0,016	1E-213	1
Rab3c	1,3596E-216	1,317749282	1	0,995	2E-212	1
Hpcal1	1,6685E-216	1,411216146	0,779	0,25	3E-212	1
Trp53i11	4,3764E-216	1,668876032	1	0,65	7E-212	1
Tubb3	2,3039E-210	1,146708236	1	0,999	4E-206	1
Proser2	6,9736E-210	0,975906817	0,581	0,114	1E-205	1
Pcdh9	1,0999E-207	1,334304013	0,781	0,251	2E-203	1
S100a11	1,9969E-207	1,653146336	0,992	0,854	3E-203	1
Hey1	2,4573E-207	0,849919494	0,545	0,098	4E-203	1
Rims1	2,1777E-206	0,833849355	0,557	0,103	4E-202	1
Phox2b	9,1576E-201	1,188424098	1	0,993	2E-196	1
Serpina3n	1,5419E-194	0,829990378	0,483	0,08	3E-190	1
Wif1	6,3104E-194	0,889738276	0,567	0,112	1E-189	1
Krt19	1,2742E-193	1,974671713	0,863	0,328	2E-189	1
Pdzd2	3,8689E-192	0,841490315	0,543	0,103	6E-188	1
Fam19a1	6,2597E-192	1,317270102	0,915	0,432	1E-187	1
Fam129a	6,3535E-192	0,445875897	0,29	0,024	1E-187	1
Chst15	1,3241E-187	1,041147226	0,72	0,2	2E-183	1
Snhg11	1,6808E-184	1,23368208	1	0,994	3E-180	1
Gabbr2	2,8974E-183	0,547051082	0,368	0,045	5E-179	1
Timp3	4,6459E-183	1,577088785	0,936	0,449	8E-179	1
Hes1	1,8181E-180	0,967346026	0,408	0,06	3E-176	1
Cpne4	5,3023E-178	1,210542236	0,988	0,822	9E-174	1
Psd3	6,5331E-173	1,194294751	0,714	0,228	1E-168	1
Hoxb5	1,5462E-170	1,141409563	1	0,956	3E-166	1
Ngfr	2,4929E-163	1,335440168	0,974	0,553	4E-159	1
Cntn5	1,9111E-161	1,103592441	0,714	0,237	3E-157	1
Fgf13	5,8135E-161	1,044504067	1	0,992	1E-156	1
Dgat2	2,3204E-158	0,649509871	0,453	0,085	4E-154	1
9530059O14Rik	9,4252E-158	1,350812114	0,982	0,756	2E-153	1
Tcerg1l	4,387E-157	0,986276657	0,668	0,203	7E-153	1
Gucy1a3	3,3571E-156	1,253229324	0,877	0,493	6E-152	1
Myl1	1,53E-155	1,092846144	1	0,935	3E-151	1
Nedd4l	1,0781E-154	1,107892694	0,863	0,45	2E-150	1
Hcn1	9,1032E-154	0,494459294	0,31	0,038	2E-149	1

Dlx3	1,1653E-152	0,864645512	0,563	0,141	2E-148	1
Prom1	3,582E-145	0,633278797	0,453	0,09	6E-141	1
Sncg	9,4505E-144	1,289699687	1	0,943	2E-139	1
Krt15	2,3193E-143	0,650110945	0,394	0,069	4E-139	1
Ank2	2,6092E-142	0,792452378	1	0,998	4E-138	1
Tubb5	1,8096E-140	0,771530579	1	1	3E-136	1
Tmem229b	4,708E-140	1,207031555	0,911	0,529	8E-136	1
A330102I10Rik	7,3557E-140	0,394196134	0,274	0,032	1E-135	1
Rph3a	4,5089E-139	1,194906807	0,95	0,638	8E-135	1
Syt2	4,1697E-138	1,072159256	0,934	0,654	7E-134	1
Iqgap2	1,815E-136	0,653780318	0,445	0,093	3E-132	1
Zfhx3	1,6355E-135	0,732894397	0,477	0,109	3E-131	1
Calb2	2,4735E-134	1,071975949	1	0,978	4E-130	1
Ptpru	6,0903E-133	0,771040921	0,517	0,132	1E-128	1
Bche	1,0161E-132	1,147108212	0,986	0,784	2E-128	1
Nkd1	1,2428E-132	0,609173473	0,463	0,102	2E-128	1
Gse1	1,5253E-131	1,030262299	0,988	0,823	3E-127	1
Adamts14	5,5635E-131	0,688463977	0,519	0,132	9E-127	1
Grin3a	7,8792E-130	1,100660316	0,748	0,324	1E-125	1
Cacna1e	4,0592E-129	1,051340695	0,885	0,488	7E-125	1
Hspb1	4,7872E-129	1,175846417	0,704	0,28	8E-125	1
mt-Nd2	1,2932E-128	0,756399367	1	1	2E-124	1
Robo2	6,3444E-128	0,959610604	0,757	0,321	1E-123	1
Rbfox3	1,8403E-126	0,895945392	0,718	0,273	3E-122	1
Fmnl2	1,902E-125	0,993401542	0,825	0,435	3E-121	1
Epb41l1	2,6618E-124	0,96100672	0,946	0,76	4E-120	1
mt-Atp6	3,2774E-124	0,576966714	1	1	6E-120	1
Snrpn	3,7847E-124	0,887010268	1	0,983	6E-120	1
Arhgap6	3,9935E-121	0,615507689	0,463	0,112	7E-117	1
Cacna1a	1,2057E-120	1,066163107	0,887	0,574	2E-116	1
Slitrk3	4,9936E-120	0,901197478	0,668	0,25	8E-116	1
Adcy1	2,993E-119	0,917309252	0,744	0,309	5E-115	1
Thbs1	5,948E-119	0,717416544	0,443	0,104	1E-114	1
Tmem63b	2,6239E-118	0,921579192	0,972	0,745	4E-114	1
Lzts1	2,1281E-116	0,640411922	0,475	0,122	4E-112	1
mt-Nd4	3,4711E-116	0,573247389	1	1	6E-112	1
Ccser2	5,3773E-116	0,976814451	0,97	0,811	9E-112	1
Apba1	8,2362E-116	0,933439586	0,962	0,747	1E-111	1
Adora1	1,0278E-115	0,501746009	0,354	0,066	2E-111	1
Prkca	3,9438E-111	0,399317179	0,288	0,046	7E-107	1
L1cam	6,1985E-111	0,781029706	1	0,981	1E-106	1
Tmeff1	3,2662E-110	1,119007401	0,815	0,489	5E-106	1
Mapk3	1,494E-109	0,861493139	1	0,991	3E-105	1
Sgcd	3,0303E-108	0,640554568	0,491	0,136	5E-104	1
Scube1	1,159E-107	0,880939779	0,996	0,904	2E-103	1
Dleu7	4,9747E-107	0,72870477	0,431	0,11	8E-103	1

Rab3b	7,3553E-107	1,060132104	0,827	0,445	1E-102	1
Zbtb7c	1,7182E-106	0,838011448	0,676	0,281	3E-102	1
Ephb2	2,0249E-106	0,562741358	0,437	0,111	3E-102	1
Nfia	4,3854E-106	0,989953051	0,853	0,518	7E-102	1
Ctbp1	2,6696E-104	0,836737618	0,984	0,873	4E-100	1
Smad6	5,14E-104	0,692218692	0,531	0,168	9E-100	1
Slc35g1	4,571E-102	0,622545095	0,467	0,13	7,7E-98	1
Tln2	9,3817E-102	0,876162792	0,95	0,804	1,6E-97	1
Cux2	7,4816E-100	0,876560995	0,871	0,578	1,3E-95	1
Thra	4,2403E-99	0,659077422	1	0,993	7,1E-95	1
Maz	6,2314E-99	0,819417548	0,978	0,866	1E-94	1
Maoa	1,68094E-97	0,913583927	0,926	0,73	2,8E-93	1
Lrrc75b	3,42723E-97	0,86792999	0,781	0,426	5,8E-93	1
Sbk1	4,18258E-97	0,657146244	0,507	0,161	7E-93	1
Chd3	6,09916E-97	0,734840662	0,992	0,951	1E-92	1
Pvrl3	6,07009E-96	0,363303148	0,262	0,043	1E-91	1
Plcb3	1,04554E-95	0,860337689	0,722	0,369	1,8E-91	1
Slitrk2	5,34675E-95	0,452670614	0,324	0,067	9E-91	1
Zbtb18	2,19536E-93	0,923039007	0,857	0,606	3,7E-89	1
Slc1a7	3,63101E-92	0,526822278	0,417	0,111	6,1E-88	1
Gm10600	8,50113E-92	0,386529125	0,29	0,055	1,4E-87	1
Lynx1	1,33569E-91	0,903784355	0,628	0,263	2,2E-87	1
Mcf2l	1,65443E-91	0,747631685	0,757	0,397	2,8E-87	1
Neat1	2,92167E-91	1,096034185	0,642	0,276	4,9E-87	1
Fam117a	1,9076E-90	0,534747768	0,35	0,082	3,2E-86	1
Tuba1b	5,15888E-90	0,707823	0,996	0,993	8,7E-86	1
Slc52a3	6,72833E-90	0,473436707	0,326	0,071	1,1E-85	1
mt-Nd4l	7,9009E-90	0,714198493	1	1	1,3E-85	1
Ptprt	9,26396E-90	0,648271788	0,543	0,182	1,6E-85	1
Col5a3	4,069E-89	0,406418719	0,308	0,062	6,8E-85	1
Ywhab	3,14123E-88	0,671162829	0,998	0,993	5,3E-84	1
Vamp1	8,18056E-88	0,953702655	0,96	0,801	1,4E-83	1
Pbx1	1,324E-87	0,829632148	0,932	0,756	2,2E-83	1
mt-Atp8	1,63323E-86	0,762197671	1	1	2,7E-82	1
Msn	4,20775E-86	0,721406224	0,974	0,874	7,1E-82	1
mt-Cytb	1,02938E-85	0,417687093	1	1	1,7E-81	1
Flot2	1,10383E-85	0,764741011	0,986	0,921	1,9E-81	1
Calm3	3,85524E-84	0,734101981	0,974	0,879	6,5E-80	1
P2rx2	5,3208E-83	0,81967993	0,998	0,983	8,9E-79	1
Lingo1	5,98101E-83	0,544481156	0,402	0,113	1E-78	1
Ptprd	9,2976E-81	0,712591903	0,972	0,88	1,6E-76	1
Nos1ap	1,23011E-80	0,608703889	0,541	0,201	2,1E-76	1
Hoxc4	2,63826E-80	0,684561122	0,986	0,906	4,4E-76	1
Atoh8	5,24074E-80	0,647587917	0,505	0,186	8,8E-76	1
Nfic	6,67065E-80	0,657035747	0,99	0,95	1,1E-75	1
Ppp2r1a	4,35883E-79	0,666080808	0,996	0,953	7,3E-75	1

Sgms1	6,21823E-79	0,473270455	0,34	0,086	1E-74	1
Fam171b	6,90816E-79	0,760988068	0,946	0,782	1,2E-74	1
Elavl3	8,83948E-79	0,691408611	0,988	0,92	1,5E-74	1
Tsc22d1	7,21752E-78	0,722238135	0,976	0,896	1,2E-73	1
Tmem178b	8,1147E-78	0,861881073	0,821	0,547	1,4E-73	1
Eml2	3,54145E-77	0,776068481	0,833	0,609	5,9E-73	1
Kif26b	2,43537E-76	0,522974538	0,437	0,139	4,1E-72	1
5330434G04Rik	3,04007E-76	0,763305725	0,942	0,789	5,1E-72	1
Cntn1	3,13839E-75	0,796772589	0,897	0,716	5,3E-71	1
Syt9	2,73008E-73	0,806102826	0,765	0,492	4,6E-69	1
Actb	4,21429E-73	0,616493206	1	1	7,1E-69	1
mt-Nd1	2,65318E-72	0,511084032	1	1	4,5E-68	1
Aatk	7,12826E-71	0,723152152	0,861	0,597	1,2E-66	1
Mpp2	7,73217E-71	0,802504506	0,702	0,409	1,3E-66	1
Mex3b	1,17301E-70	0,495864842	0,41	0,132	2E-66	1
Plekhm3	1,54279E-70	0,734460857	0,799	0,53	2,6E-66	1
Klf6	1,75377E-70	0,815822507	0,712	0,422	2,9E-66	1
Tmem189	5,5715E-70	0,725745378	0,646	0,34	9,4E-66	1
Cbx6	3,86508E-69	0,646080034	0,998	0,944	6,5E-65	1
Pak7	6,58335E-69	0,638111701	0,481	0,187	1,1E-64	1
Tmem64	6,72996E-69	0,663570186	0,966	0,888	1,1E-64	1
Smad7	6,73444E-69	0,770735583	0,734	0,448	1,1E-64	1
Ssbp3	1,24685E-68	0,610005464	0,994	0,949	2,1E-64	1
Adra2a	3,04714E-68	0,687133156	0,579	0,258	5,1E-64	1
Kif26a	6,50773E-68	0,691420583	0,917	0,701	1,1E-63	1
Il10rb	1,24282E-67	0,715894954	0,66	0,348	2,1E-63	1
Pik3r1	1,82349E-67	0,703498176	0,831	0,602	3,1E-63	1
Crmp1	1,89133E-67	0,659694154	0,982	0,902	3,2E-63	1
Mgat4b	4,54892E-67	0,72070677	0,763	0,488	7,6E-63	1
Gas6	6,04181E-67	0,523808615	0,455	0,166	1E-62	1
Slc4a4	1,60201E-66	0,645980697	0,948	0,803	2,7E-62	1
Fam102b	5,95119E-66	0,709355025	0,676	0,376	1E-61	1
Jph3	6,90858E-66	0,707996145	0,853	0,662	1,2E-61	1
Rhobtb1	2,11656E-65	0,319104363	0,29	0,072	3,6E-61	1
Scn3a	3,8903E-65	0,713044108	0,952	0,823	6,5E-61	1
Medag	7,03449E-65	0,425630833	0,268	0,065	1,2E-60	1
mt-Nd5	2,16753E-64	0,641693417	1	1	3,6E-60	1
Flrt3	3,70232E-64	0,388982699	0,28	0,07	6,2E-60	1
Tacc2	7,89743E-64	0,644351773	0,565	0,263	1,3E-59	1
Paqr8	8,14751E-64	0,685643644	0,759	0,494	1,4E-59	1
Cyfip2	1,24173E-63	0,689888743	0,841	0,653	2,1E-59	1
Ano6	2,71791E-63	0,634428721	0,942	0,796	4,6E-59	1
Dclk3	7,24838E-63	0,394480757	0,318	0,089	1,2E-58	1
Cxxc5	5,53939E-62	0,587447798	0,958	0,847	9,3E-58	1
Gnb2	7,78928E-62	0,416132223	1	1	1,3E-57	1
Mgat4a	7,83882E-62	0,655042252	0,6	0,298	1,3E-57	1

Cbfa2t3	1,58129E-61	0,462396755	0,332	0,098	2,7E-57	1
Igf1r	2,04114E-61	0,70214626	0,837	0,63	3,4E-57	1
Mt3	2,69989E-61	0,824895603	0,934	0,724	4,5E-57	1
Kcnq1ot1	5,49621E-61	0,957055422	0,95	0,851	9,2E-57	1
Slc44a1	6,34826E-61	0,745171096	0,857	0,649	1,1E-56	1
Syne2	1,41743E-60	0,601949675	0,487	0,202	2,4E-56	1
Ppp3ca	1,11618E-59	0,536726258	0,996	0,965	1,9E-55	1
Nt5dc3	2,04661E-59	0,73274067	0,849	0,695	3,4E-55	1
Klc1	2,20152E-59	0,47238568	1	1	3,7E-55	1
Sntg1	4,10527E-59	0,574787807	0,505	0,215	6,9E-55	1
Pex5l	4,15187E-59	0,646252116	0,716	0,437	7E-55	1
Mtus1	4,63251E-59	0,662142388	0,785	0,548	7,8E-55	1
Dlgap3	5,89555E-58	0,703136358	0,708	0,443	9,9E-54	1
Zfp423	6,00839E-58	0,342409886	0,27	0,07	1E-53	1
Cd164	1,67578E-57	0,701529423	0,644	0,381	2,8E-53	1
Dgki	1,67816E-57	0,608337005	0,575	0,285	2,8E-53	1
Zmynd11	3,96806E-57	0,608980467	0,891	0,714	6,7E-53	1
Susd2	8,81452E-57	0,654038725	0,541	0,253	1,5E-52	1
Plec	1,61715E-56	0,589651671	0,958	0,853	2,7E-52	1
Cnnm4	2,43184E-56	0,569987914	0,457	0,189	4,1E-52	1
Akt1	1,08177E-55	0,613354535	0,877	0,697	1,8E-51	1
Klf13	1,42004E-55	0,655773209	0,871	0,714	2,4E-51	1
Clock	8,47214E-55	0,727522961	0,763	0,537	1,4E-50	1
Foxo6	1,26845E-54	0,401107536	0,338	0,11	2,1E-50	1
Ahnak	5,03991E-54	0,7067713	0,744	0,482	8,5E-50	1
Atp2b1	6,28948E-54	0,621775349	0,885	0,746	1,1E-49	1
Galnt1	1,33486E-53	0,639814993	0,692	0,438	2,2E-49	1
Ank3	1,34682E-53	0,648046737	0,718	0,46	2,3E-49	1
Atp2b2	1,56741E-53	0,642920853	0,73	0,475	2,6E-49	1
Pcdh1	1,82749E-53	0,612642282	0,847	0,674	3,1E-49	1
Wnt9a	2,95491E-53	0,411589461	0,358	0,123	5E-49	1
Tnk2	4,78781E-53	0,652674561	0,769	0,539	8E-49	1
Ap2s1	9,70988E-53	0,568674353	0,978	0,938	1,6E-48	1
Fxyd7	2,56631E-52	0,594069966	0,996	0,745	4,3E-48	1
Adgrl2	3,06067E-52	0,640614334	0,674	0,406	5,1E-48	1
Rbfox1	3,73222E-52	0,621068146	0,847	0,662	6,3E-48	1
Prkar1b	6,82211E-52	0,576251189	0,954	0,817	1,1E-47	1
Shoc2	5,03445E-51	0,639455473	0,777	0,569	8,5E-47	1
Srrm2	5,22259E-51	0,604967335	0,99	0,968	8,8E-47	1
Frmd4b	1,00575E-50	0,509305244	0,483	0,213	1,7E-46	1
Map1b	1,0218E-50	0,470311534	1	1	1,7E-46	1
Jakmip1	1,58305E-50	0,626652595	0,706	0,465	2,7E-46	1
Nav1	1,59942E-50	0,547968547	0,978	0,913	2,7E-46	1
Aff2	2,51121E-50	0,4118211	0,338	0,117	4,2E-46	1
Ppp2r2c	3,08032E-50	0,563504897	0,942	0,799	5,2E-46	1
Stxbp5	4,66895E-50	0,583489117	0,883	0,682	7,8E-46	1

App	7,51117E-50	0,429497837	0,996	0,994	1,3E-45	1
Smarcc2	9,34654E-50	0,619919873	0,881	0,722	1,6E-45	1
Atxn7l3	2,65019E-49	0,61538148	0,732	0,507	4,4E-45	1
Gcnt2	2,68627E-49	0,645308053	0,807	0,634	4,5E-45	1
Map1a	3,80551E-49	0,625776779	0,948	0,87	6,4E-45	1
Lrrn2	6,07172E-49	0,575762976	0,773	0,529	1E-44	1
Necab1	1,14912E-48	0,528776993	0,964	0,868	1,9E-44	1
Cntfr	2,73681E-48	0,519419674	0,509	0,251	4,6E-44	1
Sdc3	3,6694E-48	0,574125115	0,952	0,82	6,2E-44	1
Thsd4	5,50034E-48	0,381017022	0,346	0,12	9,2E-44	1
Arl8a	9,4862E-48	0,471559031	0,992	0,985	1,6E-43	1
Them6	9,70474E-48	0,694850517	0,586	0,347	1,6E-43	1
Clic5	1,26533E-47	0,460325132	0,461	0,203	2,1E-43	1
Anp32b	1,5032E-47	0,624041355	0,807	0,645	2,5E-43	1
Ino80d	1,75865E-47	0,586839615	0,775	0,542	3E-43	1
Cacna1h	4,031E-47	0,409803864	0,38	0,145	6,8E-43	1
Sh3bgrl2	6,74903E-47	0,371919667	0,302	0,1	1,1E-42	1
mt-Nd3	6,88896E-47	0,447527638	1	1	1,2E-42	1
Agrn	1,6672E-46	0,613738566	0,485	0,235	2,8E-42	1
Klf7	1,66843E-46	0,486126671	0,986	0,947	2,8E-42	1
Map3k1	2,71132E-46	0,591414093	0,708	0,471	4,6E-42	1
Clgn	3,8274E-46	0,515162761	0,467	0,22	6,4E-42	1
Kcnk3	8,17803E-46	0,513202414	0,465	0,217	1,4E-41	1
Myadm	3,7557E-45	0,498590902	0,964	0,831	6,3E-41	1
Cadm3	5,15638E-45	0,532160488	0,907	0,755	8,7E-41	1
Hivep3	5,23987E-45	0,537436122	0,771	0,561	8,8E-41	1
Plcl1	9,62062E-45	0,33687505	0,326	0,115	1,6E-40	1
Prkag2	1,1303E-44	0,559966473	0,624	0,376	1,9E-40	1
Maob	1,10613E-43	0,506250066	0,515	0,26	1,9E-39	1
Mbp	1,83174E-43	0,47047966	0,439	0,196	3,1E-39	1
Pnisr	2,27945E-43	0,561431931	0,879	0,73	3,8E-39	1
Hmbox1	1,62385E-42	0,556464917	0,732	0,524	2,7E-38	1
2610001J05Rik	2,70176E-42	0,534030843	0,559	0,314	4,5E-38	1
Gdpd5	1,11085E-41	0,480796938	0,515	0,269	1,9E-37	1
Rbms3	1,66468E-41	0,426573096	1	0,992	2,8E-37	1
Cacnb1	1,73393E-41	0,552605689	0,775	0,594	2,9E-37	1
Nyap2	5,06169E-41	0,326031831	0,298	0,105	8,5E-37	1
Ppp3cb	5,08099E-41	0,540977815	0,841	0,694	8,5E-37	1
Plxna4	5,53215E-41	0,436860129	0,996	0,965	9,3E-37	1
Nrxn2	5,90586E-41	0,443470249	0,996	0,961	9,9E-37	1
Arap1	9,8915E-41	0,406730682	0,398	0,173	1,7E-36	1
Il13ra1	1,78773E-40	0,576996661	0,612	0,394	3E-36	1
mt-Co2	2,25763E-40	0,271339005	1	1	3,8E-36	1
Hs3st5	2,56407E-40	0,468246534	0,435	0,204	4,3E-36	1
F2r	3,50887E-40	0,536556898	0,873	0,579	5,9E-36	1
Fryl	4,13427E-40	0,475587132	0,523	0,28	6,9E-36	1

Zfp704	1,02257E-39	0,523405125	0,596	0,353	1,7E-35	1
Nek1	1,07638E-39	0,561861649	0,746	0,536	1,8E-35	1
Unc5b	1,43431E-39	0,551669138	0,716	0,501	2,4E-35	1
B3gnt8	1,75129E-39	0,29103203	0,296	0,105	2,9E-35	1
Apba2	2,53838E-39	0,575848581	0,869	0,713	4,3E-35	1
Tcf7	3,18107E-39	0,364654498	0,362	0,149	5,3E-35	1
1700025G04Rik	3,94915E-39	0,308260943	0,272	0,092	6,6E-35	1
Tspan9	4,42595E-39	0,541724859	0,696	0,46	7,4E-35	1
Hoxb6	5,11676E-39	0,53027146	0,588	0,339	8,6E-35	1
Marcks	5,428E-39	0,449244731	0,958	0,892	9,1E-35	1
Chd4	6,11703E-39	0,494755719	0,932	0,854	1E-34	1
Ino80dos	7,51415E-39	0,465674316	0,465	0,235	1,3E-34	1
Cic	8,94884E-39	0,582207382	0,67	0,487	1,5E-34	1
Fam124a	9,83482E-39	0,295960026	0,274	0,093	1,7E-34	1
Dclk1	7,04935E-38	0,510498456	0,905	0,793	1,2E-33	1
Tnks1bp1	8,50317E-38	0,45786447	0,527	0,289	1,4E-33	1
Asap1	1,20338E-37	0,487426684	0,632	0,405	2E-33	1
Zfp618	1,23818E-37	0,495720023	0,561	0,323	2,1E-33	1
Glce	1,29126E-37	0,382304523	0,368	0,157	2,2E-33	1
Ap1s2	1,29343E-37	0,578296	0,638	0,435	2,2E-33	1
Spock2	1,40764E-37	0,370827873	1	0,978	2,4E-33	1
Pcbp4	1,77868E-37	0,471973679	0,875	0,75	3E-33	1
Fus	2,82019E-37	0,458204422	0,984	0,941	4,7E-33	1
Tub	3,22667E-37	0,523810726	0,887	0,762	5,4E-33	1
Grem2	5,97771E-37	0,412172991	0,276	0,101	1E-32	1
Mesdc1	1,54855E-36	0,387738426	0,388	0,175	2,6E-32	1
Fam49a	1,61755E-36	0,574649688	0,644	0,432	2,7E-32	1
Csnk1d	1,82902E-36	0,509619541	0,817	0,64	3,1E-32	1
Npc2	6,69033E-36	0,524129064	0,823	0,706	1,1E-31	1
Nfib	6,78525E-36	0,463059125	0,952	0,879	1,1E-31	1
Gan	8,22365E-36	0,493921703	0,505	0,28	1,4E-31	1
Gpc1	9,41291E-36	0,600304401	0,612	0,419	1,6E-31	1
Ankrd11	1,1499E-35	0,455377417	0,948	0,87	1,9E-31	1
Man1c1	2,13496E-35	0,384206826	0,378	0,169	3,6E-31	1
Slc29a4	2,29635E-35	0,529038483	0,583	0,367	3,9E-31	1
Chl1	3,82272E-35	0,483318975	0,92	0,772	6,4E-31	1
Tnr	4,80905E-35	0,33401829	0,268	0,098	8,1E-31	1
Zmiz1	5,81296E-35	0,452717711	0,875	0,721	9,8E-31	1
Bsn	9,22119E-35	0,513738177	0,698	0,515	1,5E-30	1
Asap2	1,45583E-34	0,430491176	0,485	0,263	2,4E-30	1
Srgap3	2,6632E-34	0,473745035	0,841	0,67	4,5E-30	1
Ano2	2,79944E-34	0,585043266	0,636	0,436	4,7E-30	1
Gnaq	3,24096E-34	0,442539358	0,899	0,8	5,4E-30	1
Btbd2	4,67309E-34	0,528464681	0,698	0,497	7,8E-30	1
Sh3pxd2a	6,2251E-34	0,502593989	0,763	0,606	1E-29	1
Igsf3	7,16102E-34	0,493228389	0,74	0,528	1,2E-29	1

Rnaseh2b	7,649E-34	0,34730288	0,346	0,151	1,3E-29	1
Spsb1	9,41255E-34	0,352114435	0,304	0,122	1,6E-29	1
Dlg4	9,42232E-34	0,507215069	0,769	0,607	1,6E-29	1
Ebf4	1,55947E-33	0,305350666	0,282	0,108	2,6E-29	1
Adgrb1	2,69144E-33	0,514832622	0,706	0,52	4,5E-29	1
Cachd1	7,1155E-33	0,35672518	0,398	0,186	1,2E-28	1
Ei24	7,59459E-33	0,682418602	0,744	0,641	1,3E-28	1
Pald1	7,78947E-33	0,310974721	0,308	0,126	1,3E-28	1
Rybp	2,04434E-32	0,466190558	0,658	0,45	3,4E-28	1
Pip4k2b	2,19838E-32	0,502017027	0,668	0,486	3,7E-28	1
Brd3	2,52912E-32	0,499188906	0,757	0,618	4,2E-28	1
Kcna2	3,00076E-32	0,296509212	0,284	0,111	5E-28	1
Scn5a	4,76148E-32	0,47433686	0,803	0,658	8E-28	1
Eif4a1	6,65699E-32	0,373442167	0,99	0,977	1,1E-27	1
Ubtf	7,27076E-32	0,465917232	0,815	0,689	1,2E-27	1
Cacng4	1,18523E-31	0,460192169	0,628	0,4	2E-27	1
Robo1	1,63978E-31	0,499695814	0,71	0,549	2,8E-27	1
Gdi1	2,9948E-31	0,378702046	0,992	0,975	5E-27	1
Rgs9	3,49226E-31	0,446518813	0,889	0,75	5,9E-27	1
Cacna1b	3,88448E-31	0,454524716	0,813	0,668	6,5E-27	1
Arid1a	4,05409E-31	0,487388608	0,694	0,507	6,8E-27	1
Kcnn3	5,08179E-31	0,447152548	0,73	0,543	8,5E-27	1
Ppp1r9b	6,84823E-31	0,499355812	0,724	0,568	1,1E-26	1
Arl5a	7,74511E-31	0,511415381	0,59	0,389	1,3E-26	1
Astn2	8,96539E-31	0,45530607	0,567	0,348	1,5E-26	1
Sox4	1,39714E-30	0,415250917	0,946	0,863	2,3E-26	1
Camta1	2,2448E-30	0,424163591	0,934	0,864	3,8E-26	1
Kcnq3	3,12044E-30	0,489619078	0,753	0,583	5,2E-26	1
Lars2	4,59824E-30	0,410296724	0,996	0,989	7,7E-26	1
Hspb8	9,02763E-30	0,437040428	0,986	0,959	1,5E-25	1
Sgpl1	9,03016E-30	0,48130731	0,553	0,363	1,5E-25	1
Mtcl1	1,10344E-29	0,501079636	0,658	0,492	1,9E-25	1
Ppfia2	1,721E-29	0,347500199	0,453	0,242	2,9E-25	1
Nxpe3	5,16092E-29	0,315140368	0,314	0,139	8,7E-25	1
Nfat5	8,273E-29	0,450610578	0,736	0,567	1,4E-24	1
Elavl4	9,16729E-29	0,326388372	0,996	0,998	1,5E-24	1
Dkk3	1,28001E-28	0,449064527	0,501	0,299	2,1E-24	1
Soga3	1,59646E-28	0,409192236	0,95	0,857	2,7E-24	1
Itpk1	1,63019E-28	0,406193541	0,423	0,23	2,7E-24	1
Faah	1,6696E-28	0,379129157	0,384	0,195	2,8E-24	1
Spin1	1,97735E-28	0,535939803	0,7	0,537	3,3E-24	1
Hsp90ab1	2,15547E-28	0,265390623	1	1	3,6E-24	1
Gpsm3	2,53148E-28	0,364906714	0,427	0,215	4,3E-24	1
Slc12a7	2,79332E-28	0,438735556	0,529	0,334	4,7E-24	1
Map4	3,26682E-28	0,374179352	0,992	0,976	5,5E-24	1
Abca8b	4,74015E-28	0,372989405	0,501	0,295	8E-24	1

Mtpn	4,90883E-28	0,462480199	0,863	0,773	8,2E-24	1
Klhl21	6,2227E-28	0,383286758	0,429	0,232	1E-23	1
Cpt1a	7,56489E-28	0,440577865	0,68	0,491	1,3E-23	1
Cdh6	8,03117E-28	0,384296535	0,392	0,203	1,3E-23	1
Snap47	8,55106E-28	0,433665298	0,924	0,882	1,4E-23	1
Arhgef11	9,42499E-28	0,429140438	0,847	0,739	1,6E-23	1
Tnpo1	1,07319E-27	0,467363404	0,765	0,622	1,8E-23	1
Esyt1	1,12216E-27	0,448831833	0,632	0,443	1,9E-23	1
Mcu	2,26085E-27	0,306310746	0,296	0,13	3,8E-23	1
Ptprs	2,56039E-27	0,380728995	0,899	0,76	4,3E-23	1
Tapt1	3,4684E-27	0,435270233	0,457	0,269	5,8E-23	1
N4bp2	3,85403E-27	0,403559153	0,423	0,23	6,5E-23	1
Dusp11	4,99402E-27	0,461272875	0,751	0,603	8,4E-23	1
Vamp4	5,10908E-27	0,525036173	0,775	0,679	8,6E-23	1
Klf10	5,55205E-27	0,423156296	0,459	0,268	9,3E-23	1
Syt7	7,01019E-27	0,364498788	0,934	0,829	1,2E-22	1
Plxna2	7,04573E-27	0,329060959	0,388	0,196	1,2E-22	1
Nfkbia	9,17011E-27	0,441475275	0,509	0,322	1,5E-22	1
Parp1	1,2963E-26	0,415646253	0,571	0,387	2,2E-22	1
Cbarp	1,55205E-26	0,367337195	0,952	0,892	2,6E-22	1
Prkaca	2,30891E-26	0,376892767	0,905	0,825	3,9E-22	1
Fkbp1a	6,33989E-26	0,334232567	0,996	0,995	1,1E-21	1
Hoxa5	7,58608E-26	0,294067811	0,992	0,939	1,3E-21	1
Nsfl1c	9,33851E-26	0,440266645	0,771	0,639	1,6E-21	1
Ier5l	1,19544E-25	0,337157015	0,322	0,155	2E-21	1
Prrc2c	1,50556E-25	0,342681969	0,986	0,932	2,5E-21	1
Rtn3	1,85713E-25	0,30876161	1	0,998	3,1E-21	1
Dnmt3a	2,03585E-25	0,388601661	0,833	0,707	3,4E-21	1
Samd14	2,17067E-25	0,478335524	0,837	0,722	3,6E-21	1
Cdc42bpb	2,64516E-25	0,482640131	0,688	0,569	4,4E-21	1
Slc41a3	2,77307E-25	0,318244664	0,256	0,109	4,7E-21	1
Ddx5	4,5199E-25	0,315059974	1	0,999	7,6E-21	1
Erf	4,82534E-25	0,398393836	0,406	0,228	8,1E-21	1
Fam101b	5,86889E-25	0,35432501	0,396	0,213	9,9E-21	1
Kdsr	7,42311E-25	0,406721626	0,563	0,376	1,2E-20	1
Manea	1,13815E-24	0,352768171	0,328	0,161	1,9E-20	1
Irs3	1,17046E-24	0,415359766	0,433	0,244	2E-20	1
Gstm1	1,21785E-24	0,44901563	0,604	0,433	2E-20	1
Clk1	1,58096E-24	0,465259256	0,873	0,797	2,7E-20	1
Nipal3	2,38892E-24	0,414168996	0,789	0,658	4E-20	1
Sqstm1	3,43737E-24	0,389413924	0,952	0,901	5,8E-20	1
H1f0	3,52385E-24	0,427731633	0,781	0,65	5,9E-20	1
Cspg4	3,71305E-24	0,329430891	0,284	0,128	6,2E-20	1
Adam10	4,5024E-24	0,38255351	0,612	0,427	7,6E-20	1
PISD	4,91187E-24	0,478039491	0,841	0,729	8,2E-20	1
Aldh2	7,41669E-24	0,43991925	0,487	0,321	1,2E-19	1

Ddx3x	7,95114E-24	0,379745152	0,849	0,731	1,3E-19	1
Ppp4c	9,28345E-24	0,410118618	0,499	0,323	1,6E-19	1
Mmp24	1,04631E-23	0,304045696	0,396	0,215	1,8E-19	1
Msl2	1,16271E-23	0,355032265	0,433	0,249	2E-19	1
Hr	1,27555E-23	0,376662249	0,592	0,404	2,1E-19	1
Plekha6	1,92446E-23	0,340900668	0,905	0,779	3,2E-19	1
Ppfibp1	1,97671E-23	0,316952943	0,362	0,188	3,3E-19	1
Osbpl10	1,97902E-23	0,251526136	0,252	0,109	3,3E-19	1
Srrm4	2,10477E-23	0,39494574	0,777	0,679	3,5E-19	1
Unc5a	2,92422E-23	0,37329053	0,453	0,272	4,9E-19	1
Zeb1	4,38927E-23	0,401108182	0,779	0,652	7,4E-19	1
Myh10	4,87706E-23	0,454289463	0,714	0,563	8,2E-19	1
Arhgap5	5,49966E-23	0,420071892	0,588	0,406	9,2E-19	1
Foxn3	7,09681E-23	0,335725056	0,427	0,25	1,2E-18	1
Larp1	7,93622E-23	0,403363446	0,722	0,609	1,3E-18	1
Cabp1	7,96113E-23	0,465385296	0,634	0,482	1,3E-18	1
Jup	1,15413E-22	0,388926365	0,767	0,649	1,9E-18	1
Ski	1,78788E-22	0,389568551	0,751	0,621	3E-18	1
Alms1	1,9065E-22	0,288682589	0,298	0,142	3,2E-18	1
Gm20342	3,20209E-22	0,438232473	0,517	0,344	5,4E-18	1
Id2	3,99185E-22	0,405542972	0,938	0,884	6,7E-18	1
Rcor3	4,57549E-22	0,390789668	0,608	0,44	7,7E-18	1
Hpca	5,2174E-22	0,460287017	0,557	0,376	8,8E-18	1
Spata13	5,989E-22	0,351794783	0,443	0,259	1E-17	1
Fhl1	9,27686E-22	0,41661759	0,656	0,521	1,6E-17	1
Ddx17	1,23429E-21	0,373738568	0,895	0,802	2,1E-17	1
Tcf20	1,25217E-21	0,400280654	0,602	0,444	2,1E-17	1
Magee1	1,62429E-21	0,416550054	0,779	0,662	2,7E-17	1
Cds2	1,90244E-21	0,317877593	0,976	0,915	3,2E-17	1
Kmt2a	3,19844E-21	0,345908278	0,843	0,712	5,4E-17	1
Anxa7	4,34448E-21	0,386035808	0,656	0,495	7,3E-17	1
Sv2a	4,43776E-21	0,372479972	0,865	0,786	7,5E-17	1
Zfp652	4,65758E-21	0,359493626	0,463	0,291	7,8E-17	1
Kcnh1	5,05454E-21	0,344094164	0,513	0,335	8,5E-17	1
Msl1	5,49756E-21	0,405130419	0,734	0,605	9,2E-17	1
Dpf1	6,38461E-21	0,297496272	0,31	0,158	1,1E-16	1
Rnf44	6,97024E-21	0,377456783	0,581	0,414	1,2E-16	1
Cux1	6,99662E-21	0,398219109	0,567	0,422	1,2E-16	1
Adam23	1,58615E-20	0,368276611	0,509	0,344	2,7E-16	1
Gnb1	2,6726E-20	0,266811253	0,994	0,989	4,5E-16	1
Pitpnm2	2,92622E-20	0,351709929	0,795	0,661	4,9E-16	1
Hmgcs1	2,94407E-20	0,411886847	0,946	0,926	4,9E-16	1
H2-K1	3,00517E-20	0,433570234	0,817	0,724	5E-16	1
Lcorl	3,66838E-20	0,324379507	0,437	0,265	6,2E-16	1
Sgk3	4,20091E-20	0,296033747	0,28	0,141	7,1E-16	1
Magi1	4,43779E-20	0,377355689	0,549	0,389	7,5E-16	1

Fam129b	4,79125E-20	0,319136296	0,463	0,291	8E-16	1
Fam168a	5,97915E-20	0,377090681	0,656	0,513	1E-15	1
Nell1	6,19784E-20	0,283621862	0,499	0,296	1E-15	1
Miat	6,67731E-20	0,330173792	0,394	0,227	1,1E-15	1
Inf2	8,02575E-20	0,258051689	0,27	0,131	1,3E-15	1
Arhgef1	8,77726E-20	0,382041315	0,692	0,568	1,5E-15	1
Pth1r	1,25395E-19	0,298001403	0,41	0,232	2,1E-15	1
Pde4a	2,10141E-19	0,309043396	0,322	0,172	3,5E-15	1
Nt5dc2	2,27284E-19	0,361877479	0,457	0,293	3,8E-15	1
Erc1	2,70061E-19	0,349488927	0,783	0,68	4,5E-15	1
Efnb1	3,4978E-19	0,270784746	0,288	0,145	5,9E-15	1
Map3k5	3,60497E-19	0,279613766	0,296	0,152	6,1E-15	1
Tbx3	3,62215E-19	0,30108722	0,99	0,944	6,1E-15	1
Atp8a1	4,11718E-19	0,325466558	0,871	0,779	6,9E-15	1
Ccdc64	4,66135E-19	0,328667092	0,469	0,307	7,8E-15	1
Osbpl6	5,11929E-19	0,322533708	0,382	0,226	8,6E-15	1
Celsr3	5,52789E-19	0,330858732	0,483	0,319	9,3E-15	1
Adrbk1	5,73365E-19	0,370766335	0,777	0,688	9,6E-15	1
Adgrl1	5,83788E-19	0,316934751	0,934	0,877	9,8E-15	1
Tubb4a	5,8533E-19	0,371286307	0,869	0,804	9,8E-15	1
Dpysl2	6,3359E-19	0,348387172	0,984	0,984	1,1E-14	1
Myo1b	6,49502E-19	0,304753331	0,441	0,276	1,1E-14	1
Epb41l3	6,82865E-19	0,366926671	0,612	0,471	1,1E-14	1
Gigyf1	7,45088E-19	0,36949989	0,586	0,435	1,3E-14	1
Rab11b	7,93609E-19	0,326118715	0,903	0,857	1,3E-14	1
Hk1	1,02994E-18	0,303729717	0,932	0,875	1,7E-14	1
Kmt2d	1,22621E-18	0,354368156	0,563	0,403	2,1E-14	1
Ywhag	1,24604E-18	0,284877763	0,998	0,992	2,1E-14	1
Lrrn1	1,90025E-18	0,304226928	0,358	0,204	3,2E-14	1
Aff4	2,07023E-18	0,286941919	0,938	0,894	3,5E-14	1
Rbm5	2,07692E-18	0,377111374	0,793	0,705	3,5E-14	1
Map4k4	2,7453E-18	0,344244399	0,767	0,662	4,6E-14	1
Ccp110	3,16253E-18	0,309681452	0,443	0,277	5,3E-14	1
Prune	3,47173E-18	0,338168581	0,445	0,288	5,8E-14	1
Epb41l4b	4,00852E-18	0,302409884	0,39	0,231	6,7E-14	1
Smug1	4,5666E-18	0,278250342	0,314	0,168	7,7E-14	1
Ak1	5,81972E-18	0,36028109	0,706	0,603	9,8E-14	1
Pds5a	5,93382E-18	0,387024339	0,551	0,403	1E-13	1
Capzb	7,08102E-18	0,349089656	0,855	0,79	1,2E-13	1
Gnao1	8,11005E-18	0,291492366	0,982	0,966	1,4E-13	1
Bmf	9,08121E-18	0,257002189	0,276	0,143	1,5E-13	1
Matr3	1,59324E-17	0,307568945	0,926	0,879	2,7E-13	1
Larp4b	2,0867E-17	0,348674118	0,74	0,627	3,5E-13	1
Bcor	2,11811E-17	0,367100682	0,427	0,283	3,6E-13	1
Madd	2,3465E-17	0,347003976	0,748	0,644	3,9E-13	1
Hnrnpc	2,65164E-17	0,328034137	0,875	0,808	4,5E-13	1

Acvr1b	2,67341E-17	0,302345814	0,425	0,266	4,5E-13	1
Pfn1	2,7353E-17	0,310831218	0,95	0,923	4,6E-13	1
Dlx2	2,96965E-17	0,375454452	0,453	0,316	5E-13	1
Clasp2	3,20297E-17	0,328993907	0,841	0,755	5,4E-13	1
Mafg	3,54276E-17	0,357911205	0,761	0,666	5,9E-13	1
Vezt	3,98024E-17	0,376588433	0,628	0,508	6,7E-13	1
Samd1	4,20098E-17	0,323441405	0,487	0,332	7,1E-13	1
Rgs11	4,28071E-17	0,295983756	0,382	0,231	7,2E-13	1
Rap1gap	4,39812E-17	0,346821913	0,553	0,401	7,4E-13	1
Kif5a	5,28632E-17	0,291448224	0,988	0,979	8,9E-13	1
Krit1	5,57149E-17	0,331278473	0,736	0,614	9,4E-13	1
Ttc14	5,8357E-17	0,37745206	0,75	0,64	9,8E-13	1
Gpr149	5,85253E-17	0,282098955	0,569	0,375	9,8E-13	1
Actg1	7,16902E-17	0,290541624	1	1	1,2E-12	1
Ptms	7,197E-17	0,273299301	0,998	1	1,2E-12	1
Htt	7,63168E-17	0,306248072	0,614	0,466	1,3E-12	1
Fbxl16	8,03581E-17	0,322203627	0,763	0,64	1,3E-12	1
Mark2	8,63444E-17	0,340238391	0,688	0,556	1,4E-12	1
Adamts9	1,0176E-16	0,28082034	0,467	0,291	1,7E-12	1
Appl2	1,06709E-16	0,2671724	0,322	0,181	1,8E-12	1
Man1a	1,09171E-16	0,326009621	0,441	0,286	1,8E-12	1
Atf7	1,10284E-16	0,328569933	0,586	0,438	1,9E-12	1
S100a10	1,37457E-16	0,286410116	0,996	0,985	2,3E-12	1
Grin1	1,81977E-16	0,393156033	0,704	0,609	3,1E-12	1
Usf2	2,64431E-16	0,383611402	0,688	0,596	4,4E-12	1
Tspan17	3,19948E-16	0,392748229	0,672	0,58	5,4E-12	1
Gm15800	3,813E-16	0,320453141	0,829	0,699	6,4E-12	1
Nyap1	4,94808E-16	0,31276243	0,584	0,44	8,3E-12	1
Hnrnph1	5,42219E-16	0,315900198	0,795	0,739	9,1E-12	1
Shank2	5,86457E-16	0,300943914	0,467	0,316	9,8E-12	1
Pik3cd	6,02913E-16	0,27242173	0,336	0,197	1E-11	1
Dync1i1	6,12734E-16	0,339912107	0,67	0,552	1E-11	1
Men1	6,98385E-16	0,287540531	0,384	0,239	1,2E-11	1
Tom1l2	7,45217E-16	0,332551031	0,716	0,608	1,3E-11	1
Hnrnpa0	8,74085E-16	0,279591382	0,903	0,838	1,5E-11	1
Atf4	8,82758E-16	0,29738185	0,926	0,894	1,5E-11	1
Hipk2	9,05611E-16	0,252860306	0,382	0,234	1,5E-11	1
Numbl	1,02489E-15	0,335584007	0,724	0,629	1,7E-11	1
Mgll	1,42934E-15	0,355938075	0,823	0,764	2,4E-11	1
Anxa2	1,61586E-15	0,338937509	0,964	0,935	2,7E-11	1
Cep170b	1,98789E-15	0,330124136	0,706	0,581	3,3E-11	1
Nktr	2,22696E-15	0,312744987	0,845	0,755	3,7E-11	1
Pcsk1n	2,24233E-15	0,29917184	1	1	3,8E-11	1
Srrm3	2,65985E-15	0,355101571	0,642	0,543	4,5E-11	1
Nr3c1	2,84047E-15	0,299660647	0,425	0,279	4,8E-11	1
Kmt2e	3,74449E-15	0,298387341	0,847	0,759	6,3E-11	1

Scd2	3,88715E-15	0,259626715	0,956	0,917	6,5E-11	1
Map2k3	4,21737E-15	0,311446748	0,396	0,261	7,1E-11	1
Celf3	4,33772E-15	0,309440109	0,879	0,85	7,3E-11	1
Spire2	4,44302E-15	0,294040697	0,392	0,253	7,5E-11	1
Adrbk2	4,44571E-15	0,274717383	0,98	0,943	7,5E-11	1
Mpzl1	4,87987E-15	0,26644334	0,388	0,244	8,2E-11	1
Mbd6	5,04246E-15	0,308574975	0,449	0,308	8,5E-11	1
Ackr1	5,18822E-15	0,312655998	0,445	0,307	8,7E-11	1
Boc	6,74522E-15	0,262922124	0,318	0,186	1,1E-10	1
Cntnap2	8,97626E-15	0,317631559	0,483	0,338	1,5E-10	1
Plod2	9,15947E-15	0,388449255	0,871	0,84	1,5E-10	1
Zfp462	9,48928E-15	0,292463986	0,481	0,334	1,6E-10	1
Kalrn	1,03419E-14	0,27318323	0,342	0,21	1,7E-10	1
Maea	1,04986E-14	0,364706535	0,638	0,533	1,8E-10	1
Glul	1,08239E-14	0,338840175	0,636	0,526	1,8E-10	1
Rsrp1	1,4916E-14	0,25688002	0,978	0,957	2,5E-10	1
Pabpn1	1,53588E-14	0,357140336	0,63	0,516	2,6E-10	1
Sult4a1	1,88601E-14	0,296603926	0,95	0,942	3,2E-10	1
Furin	2,27702E-14	0,335451107	0,38	0,246	3,8E-10	1
Cntn2	2,29269E-14	0,28561226	0,392	0,252	3,8E-10	1
Luc7l3	2,79482E-14	0,324739224	0,861	0,805	4,7E-10	1
Smad1	2,82367E-14	0,325771773	0,584	0,465	4,7E-10	1
Wnk2	3,47778E-14	0,270497788	0,414	0,27	5,8E-10	1
Nudt4	4,02478E-14	0,463796656	0,525	0,425	6,8E-10	1
Dock11	4,77656E-14	0,311973641	0,646	0,528	8E-10	1
Arhgef10l	4,99023E-14	0,271817511	0,477	0,335	8,4E-10	1
Arl5b	5,06946E-14	0,252847399	0,334	0,204	8,5E-10	1
Stxbp5l	5,14611E-14	0,330057895	0,551	0,427	8,6E-10	1
Bmpr2	6,64046E-14	0,29864287	0,823	0,744	1,1E-09	1
Pogz	7,41923E-14	0,256166199	0,425	0,285	1,2E-09	1
Arhgef25	7,45902E-14	0,33565491	0,527	0,401	1,3E-09	1
Tet3	9,35765E-14	0,305559421	0,521	0,388	1,6E-09	1
Ctnna2	1,05424E-13	0,33756803	0,626	0,513	1,8E-09	1
2700081O15Rik	1,10116E-13	0,309314657	0,583	0,455	1,8E-09	1
Csnk1e	1,10477E-13	0,298409757	0,801	0,734	1,9E-09	1
Hnrnpu	1,12681E-13	0,326735278	0,692	0,605	1,9E-09	1
Ubqln4	1,45244E-13	0,312691953	0,451	0,322	2,4E-09	1
Copa	1,82923E-13	0,269897108	0,831	0,748	3,1E-09	1
Clip3	2,01921E-13	0,310466317	0,891	0,847	3,4E-09	1
Chsy1	2,37788E-13	0,296766083	0,451	0,323	4E-09	1
Zcchc14	2,61111E-13	0,316836823	0,479	0,35	4,4E-09	1
Magi3	2,70642E-13	0,282578942	0,455	0,324	4,5E-09	1
Eno2	2,75798E-13	0,310730088	0,895	0,834	4,6E-09	1
Gatad2b	2,77046E-13	0,280933912	0,684	0,552	4,7E-09	1
Npr2	2,97972E-13	0,319993999	0,583	0,47	5E-09	1
Gm26917	3,15379E-13	0,434803296	0,618	0,523	5,3E-09	1

Gtf3c1	3,3092E-13	0,306211129	0,628	0,514	5,6E-09	1
Tbc1d8	3,329E-13	0,31070955	0,481	0,354	5,6E-09	1
Tmem151a	3,89159E-13	0,40428855	0,696	0,649	6,5E-09	1
Ciapin1	4,10394E-13	0,378373934	0,738	0,675	6,9E-09	1
Ulk1	4,22174E-13	0,292595176	0,555	0,428	7,1E-09	1
BC037034	4,35214E-13	0,290261192	0,425	0,295	7,3E-09	1
Diras1	4,38051E-13	0,291933769	0,457	0,326	7,4E-09	1
Atf7ip	4,42484E-13	0,311196639	0,561	0,438	7,4E-09	1
Zbtb7a	4,5324E-13	0,283682129	0,696	0,601	7,6E-09	1
Tspyl1	5,7243E-13	0,355206241	0,64	0,536	9,6E-09	1
Phf12	6,11962E-13	0,281960298	0,443	0,312	1E-08	1
Tns1	6,52702E-13	0,255395495	0,865	0,801	1,1E-08	1
Ogt	8,49694E-13	0,327366762	0,732	0,649	1,4E-08	1
Ankrd10	9,05432E-13	0,276347686	0,567	0,441	1,5E-08	1
Pfkp	1,19585E-12	0,301982221	0,831	0,783	2E-08	1
Gtf2ird1	1,31594E-12	0,298384135	0,606	0,504	2,2E-08	1
Hnrnpl	1,3587E-12	0,304046841	0,686	0,598	2,3E-08	1
Caskin1	1,3907E-12	0,3001142	0,521	0,4	2,3E-08	1
Arhgap12	1,40836E-12	0,305795839	0,535	0,42	2,4E-08	1
B3galt1	1,49062E-12	0,291513796	0,449	0,328	2,5E-08	1
Tulp4	1,55271E-12	0,258298414	0,899	0,831	2,6E-08	1
Pank3	1,86454E-12	0,270679153	0,706	0,61	3,1E-08	1
Atxn7	2,08783E-12	0,252746276	0,33	0,209	3,5E-08	1
Fzr1	2,49475E-12	0,28538518	0,453	0,333	4,2E-08	1
Usp31	2,50412E-12	0,278407825	0,539	0,412	4,2E-08	1
Dpysl5	2,57122E-12	0,288552857	0,632	0,521	4,3E-08	1
1700020I14Rik	3,02524E-12	0,286667566	0,499	0,375	5,1E-08	1
Adam19	3,18905E-12	0,292833122	0,503	0,378	5,4E-08	1
Bbx	3,76891E-12	0,313675994	0,497	0,391	6,3E-08	1
Litaf	3,94237E-12	0,250628994	0,36	0,237	6,6E-08	1
Wbp2	4,04293E-12	0,297782366	0,775	0,732	6,8E-08	1
Rnps1	4,1214E-12	0,304858964	0,767	0,702	6,9E-08	1
Setd1b	4,26026E-12	0,287525138	0,485	0,359	7,2E-08	1
Htr3a	4,30446E-12	0,341247774	0,738	0,667	7,2E-08	1
Ttbk2	4,41402E-12	0,285147925	0,757	0,645	7,4E-08	1
Srrm1	5,00694E-12	0,298692015	0,789	0,73	8,4E-08	1
Chfr	5,18484E-12	0,278223339	0,447	0,326	8,7E-08	1
Tle2	5,6733E-12	0,307515229	0,431	0,316	9,5E-08	1
Arf1	6,21304E-12	0,264714059	0,97	0,953	1E-07	1
Eif5a2	7,03916E-12	0,279574317	0,443	0,317	1,2E-07	1
Clic4	7,58431E-12	0,334413502	0,318	0,201	1,3E-07	1
Sept8	8,66587E-12	0,27651917	0,588	0,485	1,5E-07	1
Ppp1cb	1,10222E-11	0,294584269	0,714	0,624	1,9E-07	1
Bean1	1,33384E-11	0,353535856	0,577	0,484	2,2E-07	1
Cnot6	1,41562E-11	0,310611392	0,567	0,466	2,4E-07	1
Fry	1,42506E-11	0,3106268	0,634	0,542	2,4E-07	1

Zcchc7	1,96778E-11	0,291619619	0,718	0,654	3,3E-07	1
Brd4	2,21325E-11	0,287683709	0,67	0,586	3,7E-07	1
Ccnt2	2,3519E-11	0,283547451	0,573	0,459	3,9E-07	1
Brwd1	2,51393E-11	0,320580506	0,561	0,457	4,2E-07	1
Cpeb2	2,73685E-11	0,25704595	0,539	0,418	4,6E-07	1
Ube2g1	2,99543E-11	0,261691294	0,39	0,274	5E-07	1
Nat8l	3,2367E-11	0,25254724	0,336	0,221	5,4E-07	1
Apc2	3,33693E-11	0,279055225	0,658	0,552	5,6E-07	1
Hn1	3,45356E-11	0,25830945	0,972	0,93	5,8E-07	1
Ankhd1	3,55739E-11	0,288002552	0,483	0,377	6E-07	1
Prpf4b	3,57101E-11	0,264627895	0,96	0,936	6E-07	1
Ablim2	4,33124E-11	0,26546539	0,499	0,38	7,3E-07	1
Atxn1l	4,37787E-11	0,268048007	0,374	0,26	7,4E-07	1
Trappc10	4,46526E-11	0,250953016	0,614	0,506	7,5E-07	1
Rnf38	4,6662E-11	0,25371387	0,475	0,351	7,8E-07	1
mt-Nd6	5,5957E-11	0,28044094	0,429	0,314	9,4E-07	1
Arid2	5,9727E-11	0,277502534	0,535	0,418	1E-06	1
Scamp1	6,20296E-11	0,281686046	0,785	0,732	1E-06	1
Camk2d	7,07709E-11	0,261866482	0,813	0,77	1,2E-06	1
Kmt2c	7,71964E-11	0,265912441	0,68	0,601	1,3E-06	1
Poldip3	8,23333E-11	0,259269214	0,618	0,501	1,4E-06	1
Kcnj12	8,63718E-11	0,260221321	0,419	0,305	1,5E-06	1
Unc13a	8,76496E-11	0,275294627	0,632	0,538	1,5E-06	1
Dstyk	8,8104E-11	0,257204067	0,425	0,309	1,5E-06	1
Sppl3	1,02505E-10	0,263975303	0,662	0,563	1,7E-06	1
Kansl1	1,02583E-10	0,279072945	0,588	0,486	1,7E-06	1
Arhgap31	1,11196E-10	0,259586169	0,525	0,412	1,9E-06	1
Srsf2	1,19043E-10	0,28606005	0,744	0,666	2E-06	1
Celf1	1,21592E-10	0,252887901	0,584	0,473	2E-06	1
Mtmr6	1,3195E-10	0,252798529	0,791	0,737	2,2E-06	1
Zzef1	1,32022E-10	0,267816869	0,513	0,414	2,2E-06	1
Myo9a	1,79857E-10	0,305205735	0,726	0,676	3E-06	1
Flywch1	2,50907E-10	0,294919591	0,835	0,797	4,2E-06	1
Parvb	2,75907E-10	0,262225825	0,688	0,604	4,6E-06	1
Mau2	3,02489E-10	0,256711374	0,471	0,365	5,1E-06	1
Cbl	4,15081E-10	0,286948936	0,61	0,526	7E-06	1
Arpc4	4,57914E-10	0,267983789	0,567	0,474	7,7E-06	1
Tomm34	5,30579E-10	0,262615205	0,561	0,459	8,9E-06	1
Iqgap1	5,63578E-10	0,254717726	0,376	0,267	9,5E-06	1
Med25	6,8093E-10	0,273788049	0,586	0,501	1,1E-05	1
Pkia	7,24275E-10	0,276463839	0,457	0,348	1,2E-05	1
Odf2	9,14462E-10	0,291145378	0,469	0,37	1,5E-05	1
Mxd4	1,16304E-09	0,255806341	0,682	0,623	2E-05	1
Sorl1	1,3715E-09	0,251760848	0,676	0,595	2,3E-05	1
Pianp	1,55859E-09	0,279542538	0,533	0,447	2,6E-05	1
Vps4b	1,84494E-09	0,290852936	0,654	0,6	3,1E-05	1

Fam178a	1,87369E-09	0,2541661	0,513	0,404	3,1E-05	1
Leprot	2,16445E-09	0,259970325	0,662	0,578	3,6E-05	1
Whsc1l1	3,2564E-09	0,270139092	0,674	0,607	5,5E-05	1
Bmpr1a	3,40704E-09	0,274137276	0,473	0,371	5,7E-05	1
Fam171a2	3,62124E-09	0,284080921	0,644	0,596	6,1E-05	1
Wdr82	4,32043E-09	0,271765996	0,545	0,456	7,3E-05	1
Gopc	4,38429E-09	0,269820088	0,475	0,388	7,4E-05	1
Daam1	6,30789E-09	0,258722279	0,531	0,438	0,00011	1
Pds5b	6,34881E-09	0,263984909	0,596	0,528	0,00011	1
Sbno1	1,37448E-08	0,256721628	0,624	0,535	0,00023	1
Gt(ROSA)26Sor	2,17448E-08	0,297399927	0,495	0,421	0,00037	1
Smc5	2,61009E-08	0,253561992	0,455	0,369	0,00044	1
Pknox1	2,72939E-08	0,260320802	0,463	0,369	0,00046	1
Dnmt1	3,50942E-08	0,252328299	0,596	0,527	0,00059	1
Trim3	6,04013E-08	0,250932252	0,449	0,366	0,00101	1
Rnpc3	6,31457E-08	0,271482368	0,421	0,334	0,00106	1
Nsmf	8,32962E-07	0,282363497	0,598	0,551	0,01399	1
Ubr5	2,36293E-06	0,260677684	0,767	0,755	0,03968	1
Chic2	2,85615E-06	0,252675528	0,571	0,529	0,04796	1
B2m	4,71789E-06	0,301977807	0,684	0,661	0,07922	1
Tekt2	9,1266E-06	0,25785218	0,563	0,508	0,15324	1
Atp11b	1,8382E-05	0,311036496	0,489	0,434	0,30865	1
Gm42418	3,11126E-05	0,596645155	1	1	0,52241	1
Ndrg2	4,12983E-05	0,252369228	0,573	0,534	0,69344	1
Sst	0	6,30850147	1	0,674	0	2
Calcb	0	2,687517687	0,977	0,199	0	2
Gfra2	0	1,909638847	0,907	0,097	0	2
Fxyd7	0	1,858877337	0,997	0,565	0	2
Ly6e	0	1,691949962	0,855	0,225	0	2
Dmkn	0	1,666028396	0,735	0,047	0	2
Slc18a3	0	1,598437158	0,862	0,259	0	2
Pcdh7	0	1,583962872	0,799	0,064	0	2
Tshz2	0	1,560727927	0,992	0,735	0	2
Trp53i11	0	1,462923174	0,987	0,411	0	2
Mcam	0	1,429817299	0,934	0,468	0	2
Atp1b1	0	1,393199402	0,993	0,773	0	2
Hoxa5	0	1,382306753	0,996	0,898	0	2
Fam19a5	0	1,380172361	0,76	0,116	0	2
Ddah1	0	1,330323341	0,758	0,158	0	2
Slc10a4	0	1,328921238	0,974	0,676	0	2
Smarca2	0	1,278030378	0,997	0,906	0	2
Timp3	0	1,244599613	0,821	0,196	0	2
Serping1	0	1,225874602	0,695	0,184	0	2
Bcl2	0	1,185631597	0,778	0,226	0	2
Itga6	0	1,16865942	0,772	0,261	0	2
Chgb	0	1,160130107	0,908	0,638	0	2

Sphkap	0	1,134070083	0,596	0,03	0	2
Vipr2	0	1,122057942	0,617	0,028	0	2
Csrp2	0	1,118849471	0,688	0,207	0	2
Slc5a7	0	1,108207765	0,848	0,444	0	2
Bche	0	1,092339758	0,966	0,656	0	2
Scube1	0	1,073877411	0,992	0,841	0	2
Nrp2	0	1,065949542	0,599	0,062	0	2
Ly6h	0	1,041207003	0,727	0,317	0	2
Pak3	0	1,036336365	0,902	0,555	0	2
Avpr1a	0	1,013103665	0,576	0,013	0	2
Nell1	0	0,995996752	0,602	0,066	0	2
Nfix	0	0,974368227	0,928	0,625	0	2
Igfbp5	0	0,969763544	0,495	0,008	0	2
Rspo2	0	0,965927147	0,539	0,026	0	2
Adamts9	0	0,953197798	0,597	0,058	0	2
Prnp	0	0,910392219	0,998	0,964	0	2
Rprm	0	0,892215103	0,555	0,075	0	2
Rab3b	0	0,885570512	0,727	0,255	0	2
Sparc	0	0,879866347	0,815	0,469	0	2
Bnc2	0	0,871628733	0,515	0,019	0	2
Sez6l	0	0,856814318	0,71	0,368	0	2
Tcaf1	0	0,852097208	0,979	0,882	0	2
Rasd2	0	0,825807165	0,529	0,059	0	2
Parm1	0	0,820008857	0,96	0,845	0	2
Casz1	0	0,81649894	0,705	0,12	0	2
Nrxn3	0	0,799879173	0,786	0,198	0	2
Tcf7l2	0	0,797546942	0,986	0,73	0	2
Ryr2	0	0,797227495	0,528	0,103	0	2
Ntm	0	0,79479251	0,338	0,018	0	2
Krt19	0	0,787326997	0,602	0,161	0	2
Tspan3	0	0,779011323	0,967	0,855	0	2
Spock2	0	0,769755305	0,998	0,963	0	2
Lrrtm1	0	0,769439791	0,541	0,119	0	2
Caln1	0	0,760555275	0,445	0,008	0	2
Pla2g7	0	0,745975377	0,433	0,023	0	2
Wbscr17	0	0,73683079	0,469	0,047	0	2
Vwc2	0	0,730756866	0,479	0,082	0	2
Sez6	0	0,725115976	0,576	0,203	0	2
Ache	0	0,694342692	0,991	0,942	0	2
Piezo1	0	0,681315524	0,448	0,047	0	2
Aqp1	0	0,67828527	0,43	0,055	0	2
Kif21a	0	0,670560687	0,992	0,963	0	2
Emb	0	0,669158067	0,448	0,067	0	2
Gpsm3	0	0,654681779	0,422	0,068	0	2
Tox	0	0,624361366	0,39	0,03	0	2
St3gal6	0	0,613234417	0,441	0,092	0	2

Higd1a	0	0,612669626	0,433	0,084	0	2
Cnih3	0	0,611964999	0,374	0,008	0	2
Meg3	0	0,597392747	1	1	0	2
Zfp503	0	0,58942975	0,395	0,044	0	2
Fat4	0	0,576051453	0,349	0,017	0	2
Sema5a	0	0,566683227	0,325	0,008	0	2
Prmt8	0	0,530789873	0,324	0,004	0	2
Ptma	0	0,450839681	1	1	0	2
Ctnna1	4,1933E-301	0,772649555	0,698	0,356	7E-297	2
Flrt1	1,4979E-298	0,505846164	0,341	0,033	3E-294	2
Pth1r	2,7912E-298	0,62001392	0,433	0,086	5E-294	2
Ntrk3	4,8038E-297	0,598182213	0,917	0,553	8E-293	2
Nlgn1	3,4455E-294	0,727591039	0,616	0,26	6E-290	2
Wscd1	8,1976E-288	0,541236843	0,303	0,02	1E-283	2
Dio2	4,129E-285	0,532718003	0,3	0,02	7E-281	2
Lynx1	1,2545E-281	0,53829989	0,485	0,12	2E-277	2
Prima1	8,8677E-278	0,517414794	0,333	0,04	1E-273	2
Gdf10	2,7803E-277	0,543366294	0,343	0,044	5E-273	2
Ahi1	3,1281E-276	0,50215633	1	0,999	5E-272	2
Ppp3ca	7,2951E-276	0,634743962	0,995	0,943	1E-271	2
Ednrb	1,9347E-274	0,550984666	0,326	0,037	3E-270	2
Ebf3	5,875E-274	0,530591763	0,315	0,032	1E-269	2
Slco2a1	2,3975E-269	0,517908532	0,289	0,021	4E-265	2
Cacna1e	9,3352E-268	0,629330902	0,709	0,349	2E-263	2
App	1,1902E-267	0,511899001	0,999	0,99	2E-263	2
Sema3c	7,1509E-262	0,614655628	0,437	0,116	1E-257	2
Lrrtm4	1,0257E-260	0,411977646	0,273	0,016	2E-256	2
Zbtb20	1,9278E-258	0,687924807	0,981	0,885	3E-254	2
Bcl2l11	1,7531E-256	0,492738051	0,336	0,049	3E-252	2
Kcnb1	3,5026E-256	0,736353129	0,721	0,419	6E-252	2
Sncb	4,785E-256	0,534344012	0,421	0,103	8E-252	2
Brinp1	4,2129E-252	0,619970564	0,542	0,209	7E-248	2
Plcxd3	3,5202E-251	0,684816631	0,585	0,265	6E-247	2
Adamts5	6,4277E-251	0,670465958	0,458	0,136	1E-246	2
Chat	1,2813E-250	0,448302665	0,32	0,042	2E-246	2
Myrip	7,222E-249	0,680109999	0,629	0,305	1E-244	2
Ccser2	3,3927E-248	0,557815183	0,925	0,734	6E-244	2
Rph3a	4,0831E-248	0,6284975	0,833	0,512	7E-244	2
Kif22	9,0848E-248	0,803735603	0,876	0,64	2E-243	2
Ptprt	1,4817E-243	0,515622633	0,368	0,068	2E-239	2
Rtp4	2,3961E-243	0,517612746	0,369	0,076	4E-239	2
Tmem132c	1,9714E-241	0,510555977	0,345	0,062	3E-237	2
Ctgf	1,4921E-239	0,556246458	0,363	0,074	3E-235	2
Sulf2	4,3496E-239	0,405020453	0,463	0,112	7E-235	2
Snap25	2,3114E-238	0,50603567	1	0,999	4E-234	2
Tle3	2,5109E-237	0,5008266	0,386	0,089	4E-233	2

Cdh2	2,5601E-237	0,554561126	0,976	0,889	4E-233	2
Galntl6	2,7686E-237	0,390304097	0,262	0,02	5E-233	2
Pid1	7,1306E-233	0,639541614	0,687	0,392	1E-228	2
Hoxb5	6,7303E-232	0,567487392	0,99	0,933	1E-227	2
Ptprs	1,1446E-229	0,627860199	0,888	0,67	2E-225	2
Adcy1	1,4035E-224	0,544437259	0,517	0,185	2E-220	2
Galnt10	3,1204E-223	0,409163401	0,282	0,035	5E-219	2
Zcchc12	1,0033E-222	0,63362282	0,956	0,832	2E-218	2
Ass1	8,3788E-222	0,736648567	0,682	0,411	1E-217	2
Atxn1	2,3235E-220	0,526751824	0,382	0,098	4E-216	2
Kcnc4	6,6834E-220	0,533851702	0,442	0,141	1E-215	2
Cdk14	1,7713E-219	0,406327958	0,268	0,03	3E-215	2
Camk2b	4,2772E-219	0,674477461	0,853	0,64	7E-215	2
Snhg11	6,6095E-217	0,436437641	1	0,99	1E-212	2
Cdc14a	8,2673E-217	0,488458471	0,347	0,076	1E-212	2
Satb2	1,3183E-214	0,631420524	0,577	0,28	2E-210	2
Smg6	1,9705E-214	0,645803894	0,669	0,396	3E-210	2
Plce1	6,3027E-212	0,6613858	0,635	0,353	1E-207	2
6330403A02Rik	8,2039E-210	0,388985518	0,988	0,897	1E-205	2
Scarb2	9,1969E-210	0,632522966	0,657	0,38	2E-205	2
mt-Nd2	6,1703E-209	0,365440039	1	1	1E-204	2
Vamp1	1,5286E-208	0,599372802	0,908	0,73	3E-204	2
Nfib	3,8283E-204	0,562882731	0,957	0,823	6E-200	2
Dbt	4,444E-204	0,495127834	0,389	0,113	7E-200	2
Tac1	4,0656E-203	0,493829752	0,299	0,053	7E-199	2
Slc35g2	5,4235E-201	0,386209023	0,282	0,044	9E-197	2
Kcnn2	2,1441E-198	0,475913085	0,397	0,121	4E-194	2
Mast4	4,6337E-198	0,546586965	0,452	0,165	8E-194	2
Gm2990	1,0521E-197	0,388394262	0,299	0,056	2E-193	2
Kndc1	1,1979E-195	0,475706022	0,433	0,149	2E-191	2
Enah	1,2081E-195	0,593612932	0,946	0,844	2E-191	2
Chrm2	2,0556E-194	0,397652754	0,28	0,047	3E-190	2
Cd47	4,4873E-193	0,520567675	0,973	0,904	8E-189	2
Chd3	2,1277E-188	0,477573097	0,984	0,929	4E-184	2
Ina	2,553E-187	0,591320894	0,847	0,673	4E-183	2
Cpe	9,6513E-184	0,534801126	0,97	0,916	2E-179	2
Mxra7	1,478E-182	0,570332009	0,553	0,28	2E-178	2
Cadm3	4,2407E-182	0,527126822	0,868	0,679	7E-178	2
Ppm1h	5,4614E-180	0,573742513	0,812	0,615	9E-176	2
Grb10	3,5675E-179	0,397480477	0,305	0,068	6E-175	2
Bend5	4,3074E-179	0,598522341	0,574	0,313	7E-175	2
1810041L15Rik	5,1151E-178	0,580158545	0,851	0,673	9E-174	2
Slit3	6,7781E-177	0,549908019	0,542	0,272	1E-172	2
Slc7a14	3,6862E-176	0,58265635	0,919	0,779	6E-172	2
Celf4	7,6916E-175	0,4463182	0,99	0,965	1E-170	2
Vat1l	2,7396E-174	0,618816832	0,913	0,781	5E-170	2

Prph	7,1197E-174	0,491600373	0,999	0,951	1E-169	2
Hivep3	1,7271E-172	0,550185788	0,71	0,462	3E-168	2
Svil	3,7834E-172	0,546456584	0,656	0,41	6E-168	2
Maz	5,574E-170	0,461053751	0,949	0,81	9E-166	2
Cadm2	5,7187E-168	0,428805886	0,332	0,095	1E-163	2
Filip1	7,2947E-168	0,534589801	0,897	0,761	1E-163	2
Phox2b	8,954E-168	0,353334126	0,999	0,989	2E-163	2
Gsg1l	1,2955E-167	0,421667925	0,264	0,052	2E-163	2
Eml6	8,383E-165	0,554553439	0,681	0,443	1E-160	2
Gpc6	6,1304E-164	0,564717685	0,647	0,401	1E-159	2
Gcgr	5,3535E-162	0,494219167	0,472	0,208	9E-158	2
Ppp2r2c	2,3944E-161	0,532751885	0,895	0,736	4E-157	2
Sel1l3	1,4724E-160	0,578243782	0,731	0,518	2E-156	2
Cachd1	2,275E-158	0,393638994	0,327	0,093	4E-154	2
Plod2	5,1457E-158	0,534566599	0,917	0,779	9E-154	2
Igsf3	9,1347E-158	0,542536254	0,675	0,431	2E-153	2
Vsnl1	2,3442E-156	0,368475625	0,271	0,062	4E-152	2
Pcdh17	5,2373E-150	0,590987598	0,906	0,797	9E-146	2
Arhgap20	1,827E-149	0,452287927	0,445	0,195	3E-145	2
Ank	7,8603E-148	0,434783667	0,406	0,165	1E-143	2
Epha5	1,6576E-147	0,523831419	0,356	0,127	3E-143	2
Xylt1	5,928E-146	0,448995678	0,416	0,172	1E-141	2
6330403K07Rik	6,2053E-146	0,528599425	0,97	0,914	1E-141	2
Nrcam	3,4991E-144	0,4409304	0,397	0,162	6E-140	2
Hdac9	8,9346E-144	0,351077763	0,267	0,066	2E-139	2
Sorl1	9,4244E-144	0,513084708	0,714	0,505	2E-139	2
Scg2	2,1532E-143	0,343772863	1	1	4E-139	2
Itpr1	3,1611E-142	0,451416408	0,402	0,167	5E-138	2
Necab1	4,9061E-142	0,481447892	0,927	0,831	8E-138	2
Adgrb2	6,445E-141	0,454412893	0,47	0,229	1E-136	2
Scml4	1,1448E-140	0,385605764	0,362	0,133	2E-136	2
Dip2b	5,092E-140	0,493421485	0,703	0,501	9E-136	2
Cacng4	9,9094E-139	0,480433019	0,552	0,3	2E-134	2
Parvb	2,0554E-137	0,511452364	0,725	0,513	3E-133	2
Pou2f2	4,8518E-137	0,439712678	0,37	0,143	8E-133	2
Faim2	1,0688E-134	0,481245359	0,546	0,308	2E-130	2
Dpysl5	2,3789E-134	0,481329387	0,651	0,426	4E-130	2
Snca	1,1066E-133	0,396377398	0,992	0,932	2E-129	2
Unc5a	2,7941E-132	0,399810841	0,411	0,177	5E-128	2
Nrxn2	9,2075E-131	0,398205474	0,985	0,944	2E-126	2
Rbfox3	1,0131E-130	0,372348907	0,436	0,188	2E-126	2
Kcnma1	1,2162E-130	0,507732377	0,643	0,429	2E-126	2
Rhoc	2,0495E-130	0,524591471	0,533	0,31	3E-126	2
Rin2	4,7472E-128	0,386830471	0,355	0,138	8E-124	2
Fam171b	1,0468E-127	0,418281455	0,87	0,727	2E-123	2
Ptpn5	1,7812E-127	0,318367653	0,259	0,07	3E-123	2

Arhgap5	4,7312E-125	0,472442427	0,541	0,315	8E-121	2
Map1lc3a	8,3217E-125	0,425986647	0,99	0,975	1E-120	2
Zwint	2,7773E-124	0,38263571	0,995	0,983	5E-120	2
Reep1	2,4924E-122	0,436175166	0,843	0,704	4E-118	2
mt-Nd3	4,3578E-119	0,346322887	1	0,999	7E-115	2
Pcsk1n	4,6921E-119	0,31301057	1	1	8E-115	2
Begain	1,1588E-117	0,460937403	0,65	0,443	2E-113	2
Neat1	2,23E-117	0,401136373	0,423	0,194	4E-113	2
Ptprj	3,3007E-116	0,496125465	0,53	0,322	6E-112	2
Myt1l	4,3487E-116	0,469258542	0,695	0,508	7E-112	2
Syt11	4,5404E-116	0,349366548	0,998	0,987	8E-112	2
Dst	3,9843E-114	0,388198335	0,998	0,997	7E-110	2
Clstn1	7,4067E-114	0,440218889	0,92	0,817	1E-109	2
Adam19	1,2163E-113	0,436798838	0,504	0,287	2E-109	2
Fgf13	5,1679E-113	0,275203084	0,997	0,989	9E-109	2
Nhsl2	1,5612E-112	0,383592327	0,38	0,17	3E-108	2
Calb2	1,2686E-111	0,32655113	1	0,962	2E-107	2
Grik4	5,9967E-110	0,388748243	0,439	0,224	1E-105	2
Prkacb	2,1275E-109	0,388419678	0,966	0,894	4E-105	2
Myh10	1,9909E-108	0,451408797	0,673	0,489	3E-104	2
Mt1	6,4264E-108	0,389343619	0,28	0,1	1E-103	2
Pdlim2	7,1691E-108	0,56242105	0,412	0,21	1E-103	2
Tril	1,1217E-107	0,442549965	0,52	0,315	2E-103	2
mt-Nd1	1,5273E-107	0,265029345	1	1	3E-103	2
Slitrk3	1,8061E-107	0,322272327	0,394	0,177	3E-103	2
Tln2	3,7673E-107	0,357969805	0,885	0,753	6E-103	2
Id2	7,45E-106	0,414504114	0,929	0,853	1E-101	2
Syt17	2,5231E-104	0,430433125	0,846	0,713	4E-100	2
Magi3	2,6034E-103	0,384549868	0,447	0,237	4E-99	2
Cxxc4	6,9601E-103	0,432483563	0,708	0,541	1,2E-98	2
Ablim1	1,0636E-102	0,437495332	0,619	0,426	1,8E-98	2
Sap30	1,0665E-102	0,302481996	0,278	0,1	1,8E-98	2
Dnm3	1,0944E-101	0,410012049	0,791	0,656	1,8E-97	2
Mapre2	8,8367E-101	0,415611431	0,887	0,774	1,5E-96	2
Ctdspl	4,7681E-100	0,371645272	0,402	0,205	8E-96	2
Prkce	5,0064E-100	0,400918116	0,881	0,772	8,4E-96	2
Creg2	9,7674E-100	0,360034284	0,383	0,186	1,6E-95	2
Gaa	1,2344E-99	0,387689426	0,918	0,807	2,1E-95	2
Oprl1	4,1804E-99	0,37481687	0,389	0,195	7E-95	2
Ubash3b	2,70721E-98	0,358340484	0,372	0,179	4,5E-94	2
Psd3	2,98955E-98	0,250137524	0,372	0,162	5E-94	2
Elavl4	8,85535E-98	0,341969689	0,999	0,996	1,5E-93	2
Plppr4	1,93219E-97	0,430229267	0,699	0,511	3,2E-93	2
Tmem59l	2,01354E-97	0,395885055	0,511	0,306	3,4E-93	2
Hs2st1	2,24245E-97	0,431011515	0,533	0,345	3,8E-93	2
Ckmt1	3,64465E-97	0,462414717	0,615	0,452	6,1E-93	2

Tns3	1,26488E-96	0,332819815	0,276	0,104	2,1E-92	2
AY036118	1,83535E-96	0,401025932	0,912	0,803	3,1E-92	2
Raly	2,92555E-96	0,423498051	0,688	0,545	4,9E-92	2
Nos1ap	3,52901E-96	0,292012712	0,325	0,136	5,9E-92	2
Dnm1	3,77406E-96	0,431830657	0,722	0,571	6,3E-92	2
Rgs9	1,91243E-94	0,373359716	0,827	0,701	3,2E-90	2
Traf3	1,91986E-94	0,383569287	0,46	0,263	3,2E-90	2
Cpt1a	2,83278E-94	0,422362706	0,599	0,422	4,8E-90	2
Sept3	4,62234E-94	0,441383234	0,615	0,442	7,8E-90	2
Ypel3	5,6505E-94	0,339407101	0,953	0,912	9,5E-90	2
Adrbk2	1,64929E-93	0,361563036	0,973	0,922	2,8E-89	2
Prkcdbp	2,46081E-93	0,396043646	0,449	0,256	4,1E-89	2
Txnip	3,72734E-93	0,424271286	0,382	0,194	6,3E-89	2
Lars2	4,83615E-93	0,377110611	0,997	0,984	8,1E-89	2
Flot2	5,71827E-93	0,339631175	0,957	0,898	9,6E-89	2
Lrrn2	2,66936E-91	0,398012511	0,644	0,462	4,5E-87	2
Cntln	3,62112E-91	0,373901079	0,433	0,242	6,1E-87	2
Atp2b4	1,64245E-89	0,38687274	0,819	0,678	2,8E-85	2
Timp2	5,67308E-89	0,33156867	0,985	0,965	9,5E-85	2
Tmem229b	6,47969E-89	0,399655697	0,65	0,472	1,1E-84	2
Rnf32	1,1327E-88	0,277097732	0,272	0,107	1,9E-84	2
Ddx5	2,32636E-87	0,253638934	1	0,999	3,9E-83	2
Nlrp6	1,44246E-86	0,320790168	0,316	0,145	2,4E-82	2
Adgre1	4,73997E-86	0,446001191	0,572	0,397	8E-82	2
Pitpnm2	1,90766E-85	0,382914855	0,754	0,599	3,2E-81	2
Ifit1	2,05877E-84	0,414864681	0,46	0,281	3,5E-80	2
Ifitm3	2,3038E-84	0,527908208	0,412	0,234	3,9E-80	2
Cyth3	1,00866E-83	0,277405559	0,998	0,993	1,7E-79	2
Stat3	3,35649E-83	0,402749717	0,815	0,685	5,6E-79	2
Spata13	4,45172E-83	0,337722817	0,37	0,188	7,5E-79	2
Tspan9	7,1825E-83	0,370209008	0,575	0,391	1,2E-78	2
Dync1i1	3,67457E-82	0,415372074	0,647	0,486	6,2E-78	2
Bub3	9,59575E-81	0,34095988	0,876	0,79	1,6E-76	2
Scrn1	2,21342E-80	0,313813098	0,346	0,175	3,7E-76	2
Csmd2	8,07642E-80	0,262835473	0,268	0,109	1,4E-75	2
Nfia	4,61547E-79	0,351855397	0,64	0,454	7,7E-75	2
Nap1l1	1,03784E-78	0,389440397	0,792	0,692	1,7E-74	2
H2afy	1,45909E-78	0,29500328	0,976	0,947	2,4E-74	2
Arhgdig	3,70741E-78	0,382948744	0,738	0,621	6,2E-74	2
Gas1	5,07097E-77	0,292069114	0,264	0,111	8,5E-73	2
Ssbp3	8,4264E-77	0,310847035	0,976	0,932	1,4E-72	2
Susd2	1,19819E-76	0,307351872	0,367	0,19	2E-72	2
Sema6d	3,03685E-76	0,365746742	0,757	0,632	5,1E-72	2
Frmd4b	2,03839E-75	0,264777122	0,321	0,154	3,4E-71	2
Cabp1	2,10431E-75	0,342866644	0,582	0,416	3,5E-71	2
Gtf2i	1,13273E-74	0,362370851	0,8	0,684	1,9E-70	2

Gnaz	1,85737E-74	0,358968603	0,707	0,573	3,1E-70	2
Rab11fip4	3,46372E-74	0,323913774	0,384	0,217	5,8E-70	2
Sgip1	1,05463E-73	0,322304083	0,889	0,817	1,8E-69	2
Cbx3	1,86171E-72	0,295044856	0,952	0,916	3,1E-68	2
Tigar	2,36791E-71	0,365662047	0,509	0,348	4E-67	2
Pxdc1	5,02099E-71	0,258786434	0,296	0,139	8,4E-67	2
Stxbp5l	1,18583E-70	0,340829985	0,524	0,361	2E-66	2
Zdhhc17	1,33572E-70	0,367711645	0,67	0,536	2,2E-66	2
Phox2a	1,40192E-70	0,304438006	0,948	0,87	2,4E-66	2
Nbl1	5,76112E-70	0,354398422	0,658	0,513	9,7E-66	2
Dek	8,97797E-70	0,372057723	0,697	0,577	1,5E-65	2
Stox2	1,29086E-69	0,367889609	0,548	0,384	2,2E-65	2
Asap1	1,84215E-69	0,317537135	0,511	0,343	3,1E-65	2
Fndc1	8,82775E-69	0,311094838	0,368	0,209	1,5E-64	2
Nfasc	1,08656E-68	0,347146186	0,767	0,643	1,8E-64	2
Cited2	4,29947E-67	0,318209166	0,911	0,848	7,2E-63	2
Jph4	4,43727E-67	0,284648897	0,934	0,882	7,5E-63	2
Ctsz	9,48599E-67	0,3022265	0,36	0,201	1,6E-62	2
Dlgap3	4,87611E-66	0,31980498	0,548	0,385	8,2E-62	2
Abcd3	5,0455E-66	0,343714299	0,648	0,519	8,5E-62	2
Rgl1	1,05932E-65	0,250359464	0,28	0,133	1,8E-61	2
Rab6b	1,84176E-65	0,347239929	0,946	0,902	3,1E-61	2
Fam110b	6,01475E-65	0,267622326	0,322	0,168	1E-60	2
Tnpo1	8,37591E-65	0,348145516	0,707	0,568	1,4E-60	2
Eno2	3,05453E-64	0,321512623	0,88	0,802	5,1E-60	2
Khdrbs3	4,04511E-64	0,296533298	0,424	0,266	6,8E-60	2
Zfand5	1,12567E-63	0,323871783	0,83	0,755	1,9E-59	2
Dgkh	2,16068E-63	0,264206522	0,299	0,152	3,6E-59	2
Dip2c	3,29378E-63	0,333214646	0,505	0,359	5,5E-59	2
Epb41l4b	3,48269E-63	0,270891815	0,323	0,172	5,8E-59	2
Sox11	4,37307E-63	0,346593924	0,434	0,277	7,3E-59	2
Cd81	1,60402E-62	0,279071677	0,994	0,99	2,7E-58	2
Grina	1,38921E-61	0,302966134	0,938	0,911	2,3E-57	2
Arhgef28	1,57908E-60	0,316675921	0,795	0,708	2,7E-56	2
Cacna2d2	1,92705E-60	0,266073319	0,345	0,195	3,2E-56	2
Vamp4	2,42256E-60	0,311273131	0,752	0,629	4,1E-56	2
Irf2bpl	3,89411E-60	0,31920851	0,477	0,329	6,5E-56	2
Sacs	3,68421E-59	0,354560178	0,582	0,442	6,2E-55	2
Tmem158	3,24812E-58	0,350287826	0,775	0,679	5,5E-54	2
B4galt6	1,62926E-57	0,337123231	0,766	0,656	2,7E-53	2
Gria2	4,00687E-57	0,300936376	0,95	0,921	6,7E-53	2
Slc36a1	2,15108E-56	0,325530941	0,977	0,954	3,6E-52	2
Dcx	3,55286E-56	0,353005344	0,584	0,456	6E-52	2
Impact	6,48123E-56	0,296823215	0,928	0,869	1,1E-51	2
Epb41l2	6,64305E-56	0,323605376	0,475	0,336	1,1E-51	2
Sdc3	1,11275E-55	0,294423208	0,88	0,785	1,9E-51	2

Gm12216	2,23335E-55	0,318739793	0,559	0,427	3,8E-51	2
Napb	4,89596E-55	0,295187494	0,845	0,758	8,2E-51	2
Ankrd10	1,53378E-54	0,307029571	0,524	0,386	2,6E-50	2
Hacd3	3,31788E-54	0,319309169	0,596	0,472	5,6E-50	2
Chd6	8,55628E-54	0,300958769	0,696	0,585	1,4E-49	2
Apba2	1,27599E-53	0,321703415	0,785	0,671	2,1E-49	2
Fndc3b	1,43172E-53	0,319380309	0,433	0,295	2,4E-49	2
Synm	7,86057E-53	0,330168303	0,546	0,422	1,3E-48	2
Tnrc6c	1,23804E-52	0,288376257	0,89	0,821	2,1E-48	2
Anxa2	1,39156E-52	0,253196136	0,962	0,915	2,3E-48	2
Ncs1	2,0385E-52	0,317228014	0,651	0,534	3,4E-48	2
Zmynd8	2,35295E-52	0,276233623	0,832	0,738	4E-48	2
Mapre3	3,16598E-52	0,284298791	0,795	0,697	5,3E-48	2
Nt5dc2	3,26585E-52	0,263785819	0,382	0,238	5,5E-48	2
Cd109	5,93697E-52	0,261764393	0,325	0,192	1E-47	2
Msi1	9,85535E-51	0,259213598	0,36	0,222	1,7E-46	2
Hdac11	1,14121E-50	0,301414138	0,523	0,391	1,9E-46	2
Gpatch8	2,12426E-50	0,275359171	0,885	0,821	3,6E-46	2
S100a1	4,07297E-50	0,397510036	0,87	0,806	6,8E-46	2
Tspan7	7,07638E-50	0,276955076	0,851	0,768	1,2E-45	2
Sema4d	3,45462E-49	0,302329454	0,552	0,432	5,8E-45	2
Adam9	3,93331E-49	0,272211899	0,363	0,231	6,6E-45	2
Scn7a	5,86338E-49	0,288253505	0,445	0,311	9,8E-45	2
Twsg1	6,75832E-49	0,318128925	0,535	0,411	1,1E-44	2
Snap91	9,46652E-49	0,292452381	0,71	0,613	1,6E-44	2
Rbfox1	1,88124E-48	0,283948007	0,735	0,622	3,2E-44	2
Caly	1,93684E-48	0,29235239	0,81	0,724	3,3E-44	2
Hk1	4,01167E-48	0,251483874	0,906	0,856	6,7E-44	2
Hoxb6	1,08778E-47	0,305959672	0,428	0,292	1,8E-43	2
Plekha6	6,4704E-47	0,285536095	0,828	0,753	1,1E-42	2
Inpp4a	3,06348E-46	0,299077025	0,662	0,553	5,1E-42	2
Dner	1,42161E-45	0,300477509	0,621	0,511	2,4E-41	2
Arhgef1	1,28012E-44	0,276585385	0,635	0,527	2,1E-40	2
Tub	1,58967E-44	0,283740602	0,822	0,727	2,7E-40	2
Gtf2ird1	1,06872E-43	0,26668884	0,577	0,454	1,8E-39	2
Dusp26	1,79357E-43	0,300349674	0,701	0,622	3E-39	2
Raph1	2,9962E-43	0,297584281	0,556	0,44	5E-39	2
Phip	1,49623E-42	0,301990769	0,7	0,606	2,5E-38	2
Ifitm2	2,1215E-42	0,423092559	0,972	0,938	3,6E-38	2
Samd14	9,295E-42	0,260179268	0,781	0,685	1,6E-37	2
Hmgb1	1,27549E-41	0,266953683	0,985	0,969	2,1E-37	2
Fbrsl1	1,53344E-41	0,270868516	0,472	0,348	2,6E-37	2
PISD	5,99023E-41	0,290465804	0,781	0,699	1E-36	2
Il11ra1	2,66562E-40	0,277590476	0,592	0,481	4,5E-36	2
Hmgb2	1,19656E-39	0,258547789	0,456	0,337	2E-35	2
Sep15	1,99185E-39	0,27165355	0,858	0,807	3,3E-35	2

Acp1	7,89866E-39	0,291627339	0,561	0,459	1,3E-34	2
Magi1	1,09077E-38	0,258072266	0,465	0,344	1,8E-34	2
Ldoc1l	1,47223E-38	0,282905225	0,557	0,448	2,5E-34	2
Dlgap2	2,26886E-38	0,254190942	0,411	0,295	3,8E-34	2
Ube2e2	5,50483E-38	0,267149427	0,502	0,396	9,2E-34	2
A830039N20Rik	1,62644E-37	0,255792583	0,437	0,325	2,7E-33	2
Ptprf	1,82912E-37	0,27194139	0,494	0,387	3,1E-33	2
Gm42418	3,98916E-37	0,386581749	1	1	6,7E-33	2
9330182L06Rik	3,99562E-37	0,261003816	0,499	0,389	6,7E-33	2
Ube2e3	4,65074E-36	0,254758446	0,523	0,421	7,8E-32	2
Hsd17b12	8,35428E-36	0,260058168	0,68	0,611	1,4E-31	2
Anp32a	2,30265E-35	0,252153221	0,628	0,537	3,9E-31	2
Unc5c	1,84027E-33	0,25277311	0,481	0,37	3,1E-29	2
H2afz	1,345E-32	0,318516842	0,774	0,695	2,3E-28	2
Oxtr	6,69025E-32	0,261712537	0,528	0,434	1,1E-27	2
Atf5	2,3251E-31	0,286520752	0,622	0,55	3,9E-27	2
Hmgb3	8,77903E-31	0,251932466	0,528	0,438	1,5E-26	2
Ifi27	1,3528E-30	0,312319265	0,844	0,758	2,3E-26	2
Vip	0	5,897767308	1	0,867	0	3
Scgn	0	3,919731395	1	0,764	0	3
Dbh	0	3,751542685	0,922	0,469	0	3
Etv1	0	3,260163619	0,999	0,286	0	3
Npy	0	3,258145911	0,998	0,74	0	3
Cd24a	0	2,870426335	0,991	0,318	0	3
Moxd1	0	2,19963245	0,926	0,129	0	3
Alcam	0	2,146673619	0,963	0,324	0	3
Gfra1	0	2,087308273	0,91	0,078	0	3
Thy1	0	2,001399438	0,928	0,204	0	3
Th	0	1,968088006	0,582	0,084	0	3
Cntnap5a	0	1,745315219	0,982	0,405	0	3
F2r	0	1,724987514	0,931	0,279	0	3
Fibcd1	0	1,695540896	0,823	0,046	0	3
Igfbp7	0	1,612348605	0,8	0,033	0	3
Spock3	0	1,578974074	0,925	0,338	0	3
Gm13889	0	1,463546505	0,788	0,06	0	3
Fxyd5	0	1,458977726	0,767	0,15	0	3
S100a4	0	1,394452831	0,901	0,637	0	3
Kcnd2	0	1,356927876	0,765	0,084	0	3
Gal	0	1,345543676	0,422	0,062	0	3
Crabp1	0	1,327992961	0,699	0,307	0	3
Ngfr	0	1,30857393	0,93	0,245	0	3
Id3	0	1,301082798	0,785	0,171	0	3
Nsg1	0	1,293363891	0,999	0,977	0	3
Eef1e1	0	1,283485777	0,831	0,295	0	3
Tmod1	0	1,253472013	0,9	0,416	0	3
Tmem130	0	1,250027652	0,82	0,348	0	3

Tspan13	0	1,23710385	0,935	0,605	0	3
Camk4	0	1,216937578	0,853	0,35	0	3
Csrp1	0	1,201588232	0,996	0,945	0	3
Ndst4	0	1,184758356	0,683	0,048	0	3
Tgfb1	0	1,164511525	0,689	0,039	0	3
Auts2	0	1,159767538	0,924	0,471	0	3
Pxylp1	0	1,11339888	0,881	0,48	0	3
Chl1	0	1,103170775	0,956	0,614	0	3
Resp18	0	1,09651672	0,971	0,901	0	3
Gpr149	0	1,074591116	0,706	0,082	0	3
Lamc3	0	1,066119268	0,619	0,016	0	3
Ntsr1	0	1,065703033	0,641	0,079	0	3
Ptprz1	0	1,059517142	0,618	0,033	0	3
Npr1	0	1,057659994	0,634	0,109	0	3
Kcnt2	0	1,057604702	0,663	0,042	0	3
Entpd3	0	1,045714068	0,72	0,138	0	3
Socs2	0	1,036682774	0,683	0,099	0	3
Phlda3	0	1,033095974	0,863	0,484	0	3
A730017C20Rik	0	0,998032016	0,891	0,558	0	3
Fbn1	0	0,981883314	0,633	0,036	0	3
Adcyap1r1	0	0,97890712	0,701	0,167	0	3
Cd9	0	0,978620662	0,999	0,988	0	3
Slc18a2	0	0,976360217	0,513	0,015	0	3
Thsd7a	0	0,97523771	0,556	0,011	0	3
Fam155a	0	0,9742789	0,688	0,142	0	3
Gria3	0	0,971002223	0,575	0,014	0	3
Phactr1	0	0,959746437	0,906	0,542	0	3
Camk2a	0	0,942306955	0,878	0,586	0	3
Hpca	0	0,941860709	0,679	0,108	0	3
Asic2	0	0,93857749	0,897	0,659	0	3
Enpp1	0	0,930326332	0,63	0,058	0	3
Kcnj3	0	0,929080026	0,556	0,028	0	3
Mpc1	0	0,925298162	0,926	0,751	0	3
Ptger4	0	0,916207043	0,722	0,237	0	3
Hap1	0	0,909298101	0,953	0,788	0	3
Tm4sf4	0	0,907691512	0,591	0,134	0	3
Rimbp2	0	0,906059614	0,584	0,045	0	3
Ascl1	0	0,902269676	0,549	0,014	0	3
Pdlim5	0	0,900058753	0,648	0,143	0	3
Ppa1	0	0,887411757	0,77	0,425	0	3
Cidea	0	0,886144573	0,907	0,682	0	3
Tmem108	0	0,874234152	0,584	0,052	0	3
Insm1	0	0,872652692	0,531	0,025	0	3
Bglap	0	0,870966009	0,441	0,043	0	3
Lrrc3	0	0,863043669	0,668	0,24	0	3
Clmn	0	0,861355741	0,748	0,274	0	3

Pde3a	0	0,856071534	0,691	0,246	0	3
Spock1	0	0,85502125	0,546	0,067	0	3
Gabre	0	0,85111472	0,385	0,012	0	3
Gng5	0	0,831770834	0,752	0,346	0	3
Spats2l	0	0,819396262	0,762	0,327	0	3
Prokr1	0	0,813575799	0,483	0,007	0	3
Galnt6	0	0,812634028	0,448	0,009	0	3
Pcsk1	0	0,810767493	0,821	0,494	0	3
Sertm1	0	0,802613047	0,503	0,021	0	3
Slit2	0	0,801164466	0,663	0,21	0	3
Stxbp5	0	0,797897795	0,862	0,535	0	3
Alpl	0	0,796410903	0,459	0,043	0	3
Ttc39b	0	0,795691092	0,586	0,134	0	3
Tbx3	0	0,784610018	0,994	0,902	0	3
Kcnab2	0	0,774810964	0,618	0,201	0	3
Nrp1	0	0,772327528	0,927	0,672	0	3
Sh3bgrl	0	0,770895094	0,742	0,323	0	3
Rit2	0	0,756048942	0,782	0,412	0	3
Kif5c	0	0,753796985	0,99	0,933	0	3
Ptpre	0	0,75223065	0,507	0,029	0	3
1500009L16Rik	0	0,745914585	0,712	0,326	0	3
Wls	0	0,741027472	0,62	0,184	0	3
Sv2b	0	0,740613163	0,495	0,029	0	3
Nrsn2	0	0,735352555	0,429	0,064	0	3
B3glct	0	0,724700472	0,528	0,101	0	3
Basp1	0	0,721456753	1	0,998	0	3
Zfhx4	0	0,721437259	0,63	0,215	0	3
Tspan12	0	0,719264476	0,461	0,025	0	3
Clec14a	0	0,716574014	0,505	0,086	0	3
Smpd3	0	0,710532101	1	0,997	0	3
Bex2	0	0,709623737	1	0,998	0	3
Ptprg	0	0,691144795	0,612	0,215	0	3
Nfe2l2	0	0,673765125	0,503	0,089	0	3
Slco3a1	0	0,669842801	0,527	0,086	0	3
Mtch1	0	0,669152792	0,99	0,949	0	3
Man1a	0	0,661445549	0,516	0,084	0	3
Pcolce	0	0,661011233	0,455	0,065	0	3
F2rl2	0	0,647138096	0,429	0,016	0	3
Tes	0	0,644183015	0,524	0,113	0	3
Nav3	0	0,638839868	0,599	0,201	0	3
Synpo2	0	0,636735553	0,402	0,005	0	3
Kcnq4	0	0,632907197	0,564	0,169	0	3
Arhgap22	0	0,631681034	0,598	0,2	0	3
Tceal6	0	0,626602959	0,468	0,101	0	3
Pwwp2b	0	0,622524091	0,535	0,147	0	3
Kcnj5	0	0,621971502	0,398	0,003	0	3

Cdh11	0	0,618910982	0,407	0,007	0	3
Dpysl3	0	0,612479291	0,996	0,987	0	3
Myl1	0	0,601746538	0,988	0,892	0	3
Cpm	0	0,592084854	0,371	0,013	0	3
Rtn4rl1	0	0,580624005	0,331	0,004	0	3
Ncam1	0	0,57196425	1	0,999	0	3
Npy1r	0	0,565504019	0,353	0,028	0	3
Cpne5	0	0,565304875	0,437	0,073	0	3
Kcnq5	0	0,554455681	0,384	0,015	0	3
Trpm2	0	0,553365765	0,416	0,059	0	3
Ptgfrn	0	0,551131127	0,342	0,006	0	3
Ets1	0	0,532277357	0,425	0,062	0	3
Dach1	0	0,525831598	0,348	0,004	0	3
Qpct	0	0,512609392	0,344	0,009	0	3
Gm28905	0	0,507377672	0,325	0,008	0	3
Kcnv1	0	0,504586047	0,332	0,004	0	3
Rerg	0	0,470070442	0,307	0,004	0	3
Epb41l5	9,6793E-301	0,552225939	0,436	0,084	2E-296	3
Ece1	1,9421E-298	0,808322543	0,861	0,633	3E-294	3
Atp7a	1,7692E-297	0,554714189	0,417	0,073	3E-293	3
Lrrc4c	3,5844E-296	0,459577156	0,328	0,024	6E-292	3
Wipi1	4,0292E-296	0,622539321	0,575	0,191	7E-292	3
Ccdc109b	5,5464E-296	0,458031192	0,298	0,01	9E-292	3
Kcnk2	1,1981E-295	0,619718526	0,984	0,901	2E-291	3
Sh3kbp1	1,835E-294	0,627135476	0,612	0,23	3E-290	3
Etl4	4,1337E-294	0,441266749	0,306	0,014	7E-290	3
Spint1	7,0425E-290	0,438225294	0,284	0,006	1E-285	3
Rftn1	2,3463E-286	0,458161771	0,353	0,04	4E-282	3
Htr2c	1,6439E-284	0,465265316	0,332	0,03	3E-280	3
Vcan	3,8386E-281	0,60115438	0,378	0,058	6E-277	3
Adgra1	4,811E-280	0,468175419	0,346	0,04	8E-276	3
Rassf5	3,6433E-277	0,504402186	0,412	0,079	6E-273	3
Cntn3	1,4121E-273	0,412618709	0,273	0,007	2E-269	3
Ccdc80	7,8691E-273	0,600910407	0,525	0,165	1E-268	3
Kcnc2	8,7608E-269	0,446580127	0,339	0,041	1E-264	3
Adra2a	2,6454E-264	0,485540746	0,457	0,106	4E-260	3
Plekha5	2,5996E-261	0,721350955	0,808	0,495	4E-257	3
Myo1b	2,7878E-261	0,492513942	0,463	0,117	5E-257	3
Ptbp3	1,3068E-260	0,675035254	0,942	0,786	2E-256	3
Diablo	1,8429E-260	0,510499618	0,994	0,965	3E-256	3
Bloc1s5	8,2384E-259	0,525211897	0,475	0,136	1E-254	3
Enpp2	2,4614E-258	0,472892452	0,368	0,061	4E-254	3
Dlk1	1,1442E-256	0,509223708	0,289	0,021	2E-252	3
Cst3	2,8892E-256	0,72885025	0,968	0,91	5E-252	3
Cd1d1	1,1021E-254	0,408926101	0,291	0,022	2E-250	3
Actn1	1,2164E-254	0,625549526	0,805	0,477	2E-250	3

9530059O14Rik	3,1714E-254	0,532366877	0,91	0,636	5E-250	3
Cox7c	8,9483E-254	0,818183577	0,978	0,942	2E-249	3
Chst8	6,0023E-252	0,458281726	0,369	0,064	1E-247	3
Abat	3,3239E-250	0,420668968	0,325	0,041	6E-246	3
Slc2a13	4,7489E-249	0,596372255	0,584	0,234	8E-245	3
Cntnap2	7,123E-249	0,535382739	0,527	0,174	1E-244	3
Calm2	1,9741E-247	0,512915625	1	1	3E-243	3
Akap12	6,5852E-245	0,618696377	0,966	0,903	1E-240	3
Tagln2	1,9181E-244	0,54858325	0,988	0,954	3E-240	3
Atp6v0e	3,3233E-244	0,659911369	0,717	0,404	6E-240	3
Myo16	1,1379E-240	0,362982456	0,255	0,011	2E-236	3
Rprml	5,3884E-239	0,472176175	0,301	0,034	9E-235	3
Pbxip1	1,048E-236	0,473086891	0,366	0,071	2E-232	3
Fzd3	1,7227E-236	0,573565755	0,663	0,325	3E-232	3
Sobp	1,8947E-236	0,567550882	0,618	0,268	3E-232	3
Lst1	2,7124E-234	0,69263606	0,655	0,336	5E-230	3
AW551984	8,3679E-234	0,6073472	0,881	0,65	1E-229	3
Mtss1	8,7442E-234	0,589842314	0,64	0,3	1E-229	3
Serpini1	1,9889E-232	0,602471763	0,87	0,64	3E-228	3
Gpr153	5,2518E-232	0,485067982	0,406	0,099	9E-228	3
Parva	4,3759E-230	0,610496656	0,933	0,783	7E-226	3
Lix1	7,7688E-229	0,676406143	0,873	0,673	1E-224	3
Hs3st5	4,0867E-228	0,406280498	0,37	0,073	7E-224	3
Tbx3os1	1,6458E-226	0,452418961	0,409	0,102	3E-222	3
Arvcf	2,1627E-226	0,635851258	0,82	0,56	4E-222	3
Mrpl16	3,7615E-224	0,531589754	0,503	0,183	6E-220	3
Isoc1	9,842E-224	0,569287191	0,574	0,241	2E-219	3
Vstm2l	6,9546E-223	0,581578497	0,845	0,623	1E-218	3
Sort1	1,5164E-222	0,410904188	0,338	0,061	3E-218	3
Rasl10b	1,968E-221	0,553937864	0,609	0,275	3E-217	3
Lnx1	3,0226E-220	0,423511308	0,36	0,077	5E-216	3
Cit	3,0281E-220	0,422824667	0,343	0,065	5E-216	3
Map7	1,1712E-219	0,529263315	0,582	0,248	2E-215	3
Astn2	6,4557E-217	0,517310276	0,53	0,2	1E-212	3
Ptprn	8,5382E-217	0,484794691	1	0,999	1E-212	3
Sh3bgrl3	7,2246E-216	0,736074436	0,757	0,52	1E-211	3
Ncoa7	5,9777E-214	0,655760847	0,884	0,718	1E-209	3
Ugcg	1,6262E-213	0,683851555	0,883	0,696	3E-209	3
Slc29a4	1,2035E-211	0,471635822	0,554	0,214	2E-207	3
B3galt2	2,366E-210	0,36153299	0,273	0,032	4E-206	3
Bambi	5,0471E-210	0,387249086	0,313	0,053	8E-206	3
Lmna	1,71E-209	0,527667654	0,941	0,821	3E-205	3
Mvp	1,9001E-209	0,460925728	0,489	0,173	3E-205	3
Pvrl1	5,2226E-209	0,429796541	0,356	0,079	9E-205	3
Syne1	4,871E-206	0,58785554	0,802	0,545	8E-202	3
Bmyc	7,1772E-206	0,534364421	0,665	0,349	1E-201	3

Wscd2	8,987E-206	0,445050992	0,422	0,125	2E-201	3
Rnf152	6,4914E-205	0,384079676	0,277	0,035	1E-200	3
Tmem200a	2,2678E-204	0,364411441	0,261	0,028	4E-200	3
Chga	4,8884E-203	0,580360753	0,804	0,563	8E-199	3
Sept9	3,2027E-202	0,381752486	0,271	0,034	5E-198	3
Ap3s1	5,3017E-202	0,558832007	0,763	0,49	9E-198	3
Tgfb1i1	5,3085E-202	0,512498795	0,668	0,346	9E-198	3
Tnni1	2,302E-199	0,410953139	0,365	0,091	4E-195	3
Acot7	4,0569E-196	0,576434056	0,844	0,66	7E-192	3
Fam89a	1,6897E-194	0,449599435	0,41	0,126	3E-190	3
Nxn	2,3589E-194	0,353987004	0,284	0,045	4E-190	3
Cald1	4,6868E-193	0,457871935	0,481	0,18	8E-189	3
Lgals1	4,7777E-193	0,812255351	0,94	0,905	8E-189	3
Adora2a	8,9661E-191	0,401015797	0,357	0,092	2E-186	3
Rpl14	1,3579E-190	0,547463229	0,985	0,984	2E-186	3
Lrrn1	2,2921E-190	0,387812383	0,349	0,083	4E-186	3
Tcp11l1	3,9789E-189	0,463340782	0,582	0,267	7E-185	3
Tmsb4x	1,1863E-188	0,453078839	1	1	2E-184	3
Ncam2	3,1373E-188	0,519367709	0,877	0,693	5E-184	3
Tubb2b	2,6755E-186	0,473269675	0,992	0,979	4E-182	3
Slc9b2	2,7455E-186	0,335197414	0,273	0,042	5E-182	3
A830018L16Rik	6,4031E-186	0,445310131	0,509	0,203	1E-181	3
Calm1	6,5175E-186	0,325977722	1	1	1E-181	3
Omg	1,0621E-185	0,411123668	0,39	0,117	2E-181	3
H2afj	1,0799E-185	0,523545256	0,576	0,282	2E-181	3
Man2a1	7,9357E-184	0,642456381	0,917	0,785	1E-179	3
Nell2	1,5369E-183	0,407330524	0,352	0,092	3E-179	3
Garnl3	2,5349E-183	0,533144434	0,851	0,629	4E-179	3
Prkar1a	9,8681E-182	0,406074961	0,999	0,99	2E-177	3
Tanc1	4,7908E-181	0,321069691	0,252	0,033	8E-177	3
Kcnb2	4,2598E-180	0,532478888	0,692	0,398	7E-176	3
Gnas	6,4801E-180	0,323488235	1	1	1E-175	3
Fhod3	1,0355E-179	0,48558249	0,953	0,865	2E-175	3
Sdc2	1,5253E-179	0,403262041	0,393	0,123	3E-175	3
Pam	3,7434E-179	0,462905085	0,996	0,983	6E-175	3
Crem	1,009E-178	0,453097786	0,54	0,24	2E-174	3
Cd63	1,0714E-178	0,502450246	0,534	0,239	2E-174	3
Ucp2	1,1068E-178	0,521131493	0,63	0,325	2E-174	3
Slc22a23	8,0708E-176	0,363457548	0,315	0,072	1E-171	3
Irs3	2,8322E-175	0,40778764	0,395	0,121	5E-171	3
Gstm5	1,0746E-174	0,559494531	0,64	0,354	2E-170	3
Magt1	5,1289E-172	0,458215141	0,501	0,213	9E-168	3
Fstl5	1,1139E-170	0,404076258	0,427	0,149	2E-166	3
Myl6	6,4283E-170	0,447736667	0,973	0,932	1E-165	3
Suox	9,0675E-170	0,390877369	0,436	0,16	2E-165	3
Nol3	3,756E-169	0,43966464	0,477	0,198	6E-165	3

Nrep	4,31E-168	0,469943875	0,549	0,257	7E-164	3
Mpped2	4,5583E-168	0,338894565	0,305	0,07	8E-164	3
Mfap2	1,949E-166	0,457358395	0,456	0,183	3E-162	3
Spint2	5,3921E-166	0,417483058	0,45	0,177	9E-162	3
Slc35b4	6,5456E-166	0,512274058	0,676	0,397	1E-161	3
Kcnq3	1,4017E-165	0,518873554	0,735	0,459	2E-161	3
Cadm1	2,1427E-165	0,38087148	1	0,994	4E-161	3
Ccdc184	1,3603E-163	0,433189572	0,537	0,251	2E-159	3
Ephb6	2,2413E-163	0,458473739	0,573	0,286	4E-159	3
Nr2f2	5,1051E-163	0,318554604	0,26	0,047	9E-159	3
Carhsp1	6,4335E-163	0,455633876	0,589	0,302	1E-158	3
Slc4a8	1,144E-162	0,476038874	0,693	0,412	2E-158	3
Prmt2	1,6264E-162	0,49167954	0,82	0,615	3E-158	3
Cgref1	2,4021E-160	0,348983796	0,326	0,088	4E-156	3
Arhgef4	4,5347E-160	0,372551368	0,356	0,111	8E-156	3
Fitm2	4,1098E-159	0,498943638	0,606	0,33	7E-155	3
Prdx6	2,1026E-158	0,367993859	0,369	0,119	4E-154	3
Chrna5	3,3417E-158	0,35844933	0,374	0,121	6E-154	3
Grn	3,4204E-157	0,48245444	0,835	0,631	6E-153	3
Ier3	7,5328E-156	0,41826842	0,447	0,181	1E-151	3
Fam107b	1,0879E-155	0,294972111	0,251	0,046	2E-151	3
Sub1	1,8051E-154	0,408221218	0,978	0,934	3E-150	3
Nmt2	3,3534E-154	0,453558743	0,896	0,74	6E-150	3
Creb5	3,5299E-154	0,400495767	0,383	0,131	6E-150	3
1810058I24Rik	6,3285E-154	0,418959028	0,551	0,272	1E-149	3
Efna5	2,049E-153	0,56641683	0,771	0,536	3E-149	3
Hand2	3,6904E-152	0,355937243	1	0,982	6E-148	3
Acyp2	2,7405E-151	0,502757741	0,695	0,438	5E-147	3
Ndfip1	2,7904E-151	0,493584908	0,665	0,399	5E-147	3
Uchl1	5,419E-151	0,342487455	1	1	9E-147	3
Chst15	1,1962E-150	0,326119529	0,361	0,108	2E-146	3
Sntg1	2,2789E-150	0,332324453	0,359	0,113	4E-146	3
Rexo2	5,5016E-150	0,510813067	0,736	0,507	9E-146	3
Ramp2	7,9644E-150	0,415351536	0,347	0,113	1E-145	3
Atp1b3	1,6382E-148	0,459581431	0,96	0,895	3E-144	3
Olfm1	3,0765E-148	0,473699927	0,849	0,675	5E-144	3
Arhgap42	3,7483E-148	0,337785886	0,35	0,112	6E-144	3
Cygb	4,7014E-147	0,432141285	0,554	0,278	8E-143	3
Alad	9,4123E-147	0,400390249	0,429	0,175	2E-142	3
Polr1d	1,6639E-146	0,474770563	0,649	0,392	3E-142	3
Plcb1	3,1937E-146	0,295819447	0,254	0,052	5E-142	3
Apbb1	5,5679E-145	0,43167623	0,966	0,922	9E-141	3
Sept6	1,508E-144	0,458259673	0,709	0,453	3E-140	3
Frmpd4	1,5267E-144	0,336401029	0,316	0,092	3E-140	3
Hspb1	2,0079E-144	0,357965773	0,444	0,173	3E-140	3
Qdpr	5,0114E-144	0,471473493	0,691	0,442	8E-140	3

Mest	3,2007E-143	0,345567297	0,388	0,142	5E-139	3
Man1c1	3,447E-143	0,300042226	0,293	0,075	6E-139	3
Dynll1	8,7397E-143	0,388597012	0,995	0,984	1E-138	3
R3hdm1	1,4949E-142	0,471749943	0,92	0,806	3E-138	3
Hpcal4	2,0724E-142	0,505684961	0,718	0,474	3E-138	3
Mid1ip1	1,396E-141	0,451541722	0,72	0,472	2E-137	3
Rgs7	2,0512E-141	0,3423076	0,351	0,118	3E-137	3
Mab21l1	4,416E-141	0,434910436	0,563	0,294	7E-137	3
Shc1	3,6558E-139	0,383253451	0,473	0,213	6E-135	3
Stard4	1,3661E-138	0,47362277	0,558	0,3	2E-134	3
Clvs1	3,5424E-138	0,370288524	0,458	0,201	6E-134	3
Ldha	2,1991E-136	0,431468278	0,987	0,964	4E-132	3
Plekhb2	3,1162E-136	0,577479511	0,8	0,623	5E-132	3
Hspa2	4,2938E-136	0,343642776	0,39	0,15	7E-132	3
Ckb	1,6106E-135	0,530186872	0,82	0,662	3E-131	3
Sec61b	7,0527E-135	0,487092991	0,73	0,508	1E-130	3
Etv6	7,7887E-133	0,34591779	0,389	0,153	1E-128	3
Slc25a3	2,5802E-132	0,419475643	0,979	0,944	4E-128	3
Dstn	4,407E-132	0,559293978	0,961	0,942	7E-128	3
Syt7	5,0288E-132	0,556325073	0,899	0,775	8E-128	3
Lrrc8c	5,815E-132	0,456393902	0,794	0,583	1E-127	3
Gm38112	2,4732E-131	0,381771653	0,508	0,25	4E-127	3
Lxn	2,8935E-131	0,328313668	0,358	0,13	5E-127	3
Asl	7,7295E-130	0,441174092	0,86	0,697	1E-125	3
Tpm4	3,5781E-129	0,393344973	0,612	0,352	6E-125	3
Arpc3	3,9273E-129	0,464299828	0,853	0,722	7E-125	3
Rab8b	1,1908E-128	0,421629842	0,701	0,456	2E-124	3
Rnf217	2,7227E-127	0,434274152	0,561	0,313	5E-123	3
Klhl32	3,3771E-127	0,320312214	0,358	0,134	6E-123	3
Rgs10	4,1788E-127	0,357928266	0,416	0,179	7E-123	3
Gapdh	9,4237E-127	0,639969229	0,945	0,923	2E-122	3
Acsl5	1,8039E-126	0,354504262	0,467	0,223	3E-122	3
Ablim2	4,4126E-126	0,374134105	0,517	0,264	7E-122	3
Abcg2	2,9645E-125	0,322108339	0,363	0,14	5E-121	3
Ssr4	2,1604E-124	0,470542919	0,687	0,456	4E-120	3
Ier5	6,0273E-124	0,304002358	0,305	0,099	1E-119	3
Pnp	1,2138E-123	0,345362345	0,427	0,19	2E-119	3
Gabrg2	3,0489E-123	0,28836503	0,263	0,072	5E-119	3
Ttc9b	8,5257E-123	0,485570156	0,704	0,489	1E-118	3
Tuba1a	1,2119E-122	0,646978389	0,992	0,994	2E-118	3
Dmd	5,9567E-122	0,317824341	0,324	0,113	1E-117	3
Gnai1	8,4617E-122	0,3557843	0,498	0,251	1E-117	3
Trim9	9,3442E-122	0,294735647	0,314	0,105	2E-117	3
Hunk	1,2213E-121	0,37871355	0,57	0,317	2E-117	3
Serpinb6a	4,2058E-121	0,440789643	0,895	0,808	7E-117	3
Golim4	3,4373E-120	0,36661821	0,534	0,285	6E-116	3

Pitpnc1	4,1044E-120	0,432483849	0,663	0,411	7E-116	3
Prr13	4,7876E-120	0,407734161	0,656	0,42	8E-116	3
Sdcbp	2,3964E-119	0,414256248	0,924	0,831	4E-115	3
Hist3h2ba	3,088E-119	0,460766583	0,612	0,376	5E-115	3
Glrx5	7,6482E-119	0,389024363	0,535	0,291	1E-114	3
Ank2	1,1196E-118	0,300759124	1	0,996	2E-114	3
Sh3gl2	1,5821E-118	0,366835656	0,55	0,304	3E-114	3
Prkar1b	1,7971E-118	0,441434716	0,886	0,768	3E-114	3
Arl4a	3,102E-118	0,378156245	0,562	0,319	5E-114	3
Scg5	4,3919E-117	0,452904072	0,899	0,797	7E-113	3
Romo1	6,962E-117	0,465503355	0,91	0,836	1E-112	3
Tubb2a	1,3931E-116	0,353261325	0,997	0,994	2E-112	3
Capn5	1,6485E-116	0,408653822	0,636	0,401	3E-112	3
Palmd	4,8975E-116	0,401230713	0,568	0,319	8E-112	3
Erbb2ip	4,9476E-116	0,356024558	0,458	0,225	8E-112	3
Ywhaq	4,1194E-115	0,375430556	0,971	0,937	7E-111	3
Hadh	4,7868E-115	0,279342848	0,304	0,103	8E-111	3
Fam96b	7,5315E-115	0,363367839	0,574	0,331	1E-110	3
Rap2b	1,4087E-114	0,292974317	0,294	0,098	2E-110	3
Anks1b	2,3456E-114	0,386649138	0,658	0,407	4E-110	3
Anapc5	1,1039E-113	0,366856529	0,905	0,803	2E-109	3
Stx3	6,1424E-113	0,337166476	0,475	0,235	1E-108	3
Dlgap1	4,1314E-112	0,417590991	0,699	0,474	7E-108	3
Lrrk2	9,4264E-112	0,275213683	0,289	0,096	2E-107	3
Gsn	1,8658E-111	0,313848395	0,403	0,18	3E-107	3
Cib2	2,6871E-111	0,307627999	0,355	0,145	5E-107	3
Elk3	3,439E-111	0,381073242	0,643	0,396	6E-107	3
Cdr2	3,134E-110	0,276040455	0,305	0,108	5E-106	3
Astn1	4,802E-110	0,311453777	0,414	0,191	8E-106	3
Hsd17b11	5,9593E-110	0,305605141	0,384	0,167	1E-105	3
Pcbd1	2,323E-109	0,392818809	0,665	0,437	4E-105	3
Adk	2,5273E-109	0,284830747	0,328	0,126	4E-105	3
Grin3a	5,5865E-109	0,261365292	0,48	0,226	9E-105	3
Cdkn1a	9,6614E-109	0,387982582	0,571	0,331	2E-104	3
Cpne8	1,3894E-108	0,306846998	0,395	0,175	2E-104	3
Cox7a2l	3,5646E-108	0,487486194	0,721	0,544	6E-104	3
Rabac1	8,153E-108	0,412207984	0,935	0,873	1E-103	3
Crip1	1,522E-107	0,569597706	0,989	0,987	3E-103	3
Pak1	1,802E-107	0,387492225	0,708	0,488	3E-103	3
Slc7a1	2,0576E-107	0,360779045	0,539	0,306	3E-103	3
Slc7a8	3,345E-107	0,452849875	0,591	0,368	6E-103	3
Actg1	1,3051E-106	0,286442827	1	1	2E-102	3
Eml4	1,7898E-106	0,321016667	0,489	0,256	3E-102	3
Fam110a	1,9911E-106	0,27167901	0,262	0,082	3E-102	3
Tmem159	2,7144E-106	0,281597533	0,335	0,133	5E-102	3
Tmem55a	4,2452E-106	0,317898531	0,428	0,207	7E-102	3

Sec61g	1,3426E-105	0,522461347	0,902	0,847	2E-101	3
Ost4	4,1597E-105	0,366715803	0,613	0,378	7E-101	3
Ralyl	1,9768E-104	0,300331557	0,411	0,192	3E-100	3
Pfn2	2,092E-104	0,366613321	0,916	0,83	4E-100	3
Tmem56	3,7727E-103	0,384000057	0,561	0,328	6E-99	3
Tmem256	1,9414E-102	0,470025593	0,628	0,419	3,3E-98	3
Kctd1	3,1887E-102	0,289595633	0,397	0,181	5,4E-98	3
Zbtb8os	1,0324E-101	0,250193168	0,257	0,082	1,7E-97	3
Acer3	1,0431E-101	0,293566232	0,357	0,154	1,8E-97	3
Rpl41	6,6311E-101	0,366277612	0,998	0,999	1,1E-96	3
Slc3a2	9,5148E-101	0,417848279	0,742	0,564	1,6E-96	3
Them4	1,1682E-100	0,357603913	0,622	0,397	2E-96	3
Syt16	1,1733E-100	0,264162851	0,319	0,125	2E-96	3
Fstl1	7,7648E-100	0,390948832	0,73	0,524	1,3E-95	3
Pfkl	1,7797E-99	0,309948378	0,48	0,257	3E-95	3
Hspa8	2,7679E-99	0,370878995	0,995	0,995	4,6E-95	3
Cpne3	2,8341E-99	0,390543511	0,781	0,61	4,8E-95	3
Gnai2	5,3261E-99	0,317041373	0,979	0,947	8,9E-95	3
Idnk	3,40403E-98	0,326827862	0,484	0,266	5,7E-94	3
Tmed3	1,22545E-97	0,3477465	0,492	0,274	2,1E-93	3
Tlk1	1,3823E-97	0,358638736	0,705	0,491	2,3E-93	3
Tspan6	2,32617E-96	0,273131601	0,357	0,158	3,9E-92	3
Sec24d	3,03302E-96	0,282114404	0,314	0,127	5,1E-92	3
Ninj1	3,89458E-96	0,284896159	0,344	0,15	6,5E-92	3
Vcl	4,85454E-96	0,268077921	0,327	0,136	8,2E-92	3
Gria4	8,2136E-96	0,295612779	0,407	0,196	1,4E-91	3
Cntn1	1,5097E-95	0,349184151	0,806	0,651	2,5E-91	3
Nbea	2,79442E-95	0,38118849	0,746	0,546	4,7E-91	3
Ints3	1,46221E-94	0,3512083	0,616	0,397	2,5E-90	3
Rgs17	2,31944E-94	0,321663389	0,597	0,363	3,9E-90	3
Add3	5,47429E-94	0,362094907	0,754	0,556	9,2E-90	3
Dnajc12	6,28011E-94	0,311036553	0,513	0,293	1,1E-89	3
Slc25a4	1,43676E-93	0,361377508	0,985	0,977	2,4E-89	3
Tyro3	3,55361E-93	0,358791325	0,561	0,345	6E-89	3
Gpx3	7,97819E-93	0,45656896	0,432	0,229	1,3E-88	3
Rpl22	9,80649E-93	0,420711845	0,754	0,604	1,6E-88	3
Phlda1	2,46826E-92	0,259770472	0,31	0,125	4,1E-88	3
Gm1673	4,24404E-92	0,403847187	0,842	0,722	7,1E-88	3
Creb3l2	6,63364E-92	0,297845869	0,407	0,202	1,1E-87	3
Wrap73	1,23189E-91	0,255851704	0,329	0,141	2,1E-87	3
S100a6	1,43076E-91	0,396632469	0,999	0,999	2,4E-87	3
Litaf	7,46513E-91	0,2783237	0,341	0,151	1,3E-86	3
Gm20342	1,15733E-90	0,312839551	0,464	0,249	1,9E-86	3
Eps15	2,61714E-90	0,30254333	0,471	0,26	4,4E-86	3
Fst	2,82825E-90	0,309933223	0,434	0,22	4,7E-86	3
Me2	8,99924E-90	0,264762786	0,332	0,146	1,5E-85	3

Cpq	1,00385E-89	0,261699964	0,306	0,126	1,7E-85	3
Mllt11	1,06787E-89	0,333433276	0,959	0,919	1,8E-85	3
Eml1	3,26563E-89	0,308559498	0,452	0,245	5,5E-85	3
Rnase4	1,51547E-88	0,275147198	0,366	0,172	2,5E-84	3
Nrsn1	3,61629E-88	0,325296454	0,926	0,839	6,1E-84	3
Maged2	6,24727E-88	0,315621584	0,485	0,276	1E-83	3
Neurl1a	1,28341E-87	0,41615843	0,808	0,672	2,2E-83	3
Nek1	3,08893E-87	0,318807451	0,661	0,442	5,2E-83	3
Rps4x	4,82185E-87	0,492172005	0,908	0,869	8,1E-83	3
Polr2g	6,70697E-87	0,318566711	0,598	0,378	1,1E-82	3
Ttl	6,10436E-86	0,324781621	0,601	0,392	1E-81	3
Tpst1	1,277E-85	0,25213366	0,318	0,139	2,1E-81	3
Ebp	1,3686E-85	0,295379525	0,508	0,297	2,3E-81	3
Lrrc8b	7,30548E-85	0,337378708	0,532	0,325	1,2E-80	3
Tcof1	8,96536E-85	0,311443936	0,511	0,304	1,5E-80	3
Tmem255b	9,78423E-85	0,348056916	0,838	0,695	1,6E-80	3
Rgag4	1,07055E-84	0,269020644	0,349	0,164	1,8E-80	3
Trappc1	1,91311E-84	0,298653861	0,419	0,22	3,2E-80	3
Gabarapl2	4,3343E-84	0,367611026	0,912	0,84	7,3E-80	3
2610001J05Rik	5,36E-84	0,266631274	0,434	0,229	9E-80	3
Plpp1	1,67555E-83	0,35342362	0,606	0,409	2,8E-79	3
2610524H06Rik	3,55928E-83	0,283811546	0,428	0,229	6E-79	3
Cox20	7,51492E-83	0,298268425	0,394	0,204	1,3E-78	3
Arrdc3	7,99677E-83	0,284082016	0,408	0,212	1,3E-78	3
Gadd45g	1,07689E-82	0,280101525	0,378	0,188	1,8E-78	3
Dhrs7	1,54303E-82	0,306888543	0,529	0,324	2,6E-78	3
Nnat	2,57654E-82	0,757204829	0,585	0,425	4,3E-78	3
Gabrb3	2,68625E-82	0,378252269	0,686	0,495	4,5E-78	3
Enox2	3,20063E-82	0,251803681	0,332	0,152	5,4E-78	3
Prelid1	5,42735E-82	0,323777276	0,666	0,465	9,1E-78	3
Tbpl1	5,89622E-82	0,295629168	0,527	0,319	9,9E-78	3
1810043G02Rik	6,18358E-82	0,258743513	0,382	0,192	1E-77	3
Rras	6,85243E-82	0,283887364	0,414	0,22	1,2E-77	3
Tmem258	1,164E-81	0,35993253	0,605	0,398	2E-77	3
Cox7a2	4,49008E-81	0,399988221	0,891	0,821	7,5E-77	3
Psme2	4,66454E-81	0,386510088	0,66	0,477	7,8E-77	3
Rps2	1,4143E-80	0,398161696	0,881	0,818	2,4E-76	3
Brk1	1,626E-80	0,325880282	0,655	0,451	2,7E-76	3
Tnfaip1	3,38662E-80	0,292544916	0,474	0,276	5,7E-76	3
Rps23	4,57422E-80	0,411414831	0,924	0,89	7,7E-76	3
Adgrl1	5,71931E-80	0,366992342	0,927	0,836	9,6E-76	3
Hipk2	6,44145E-80	0,257137418	0,335	0,155	1,1E-75	3
Ret	7,45742E-80	0,31232297	0,982	0,955	1,3E-75	3
Prdx2	9,37601E-80	0,412714454	0,866	0,799	1,6E-75	3
A830010M20Rik	1,50871E-79	0,300656972	0,529	0,33	2,5E-75	3
Cyb5a	2,09371E-79	0,341446119	0,709	0,525	3,5E-75	3

Gpx1	2,91226E-79	0,30191357	0,444	0,249	4,9E-75	3
Ndufa1	8,42009E-79	0,423514353	0,677	0,508	1,4E-74	3
Apbb2	1,00324E-78	0,254318226	0,364	0,181	1,7E-74	3
Dclk2	1,33073E-78	0,326436983	0,586	0,383	2,2E-74	3
Ppp1ca	2,0731E-78	0,326227525	0,895	0,812	3,5E-74	3
Pdcd10	2,17913E-78	0,33731593	0,703	0,521	3,7E-74	3
Nrip1	9,21507E-78	0,356957908	0,912	0,839	1,5E-73	3
Capns1	1,65691E-77	0,271074254	0,974	0,951	2,8E-73	3
Pgls	2,58391E-77	0,345277683	0,664	0,476	4,3E-73	3
Tmem176a	3,18774E-77	0,300933145	0,628	0,424	5,4E-73	3
Cnn3	7,87909E-77	0,26960599	0,407	0,217	1,3E-72	3
Ubc	1,02251E-76	0,297643297	0,979	0,981	1,7E-72	3
Cox4i1	1,22447E-76	0,434052839	0,906	0,875	2,1E-72	3
Bola2	7,42345E-76	0,364371557	0,725	0,568	1,2E-71	3
Dad1	1,08467E-75	0,312880721	0,879	0,781	1,8E-71	3
Rock2	1,13288E-75	0,307876551	0,664	0,462	1,9E-71	3
Ppat	1,41813E-75	0,25275071	0,416	0,227	2,4E-71	3
Mrpl23	1,79244E-75	0,311971711	0,532	0,333	3E-71	3
Taf13	1,81361E-75	0,254214992	0,439	0,245	3E-71	3
Cd151	4,2438E-75	0,324883766	0,738	0,566	7,1E-71	3
Hotairm1	7,33479E-75	0,31405882	0,538	0,343	1,2E-70	3
Gm10076	8,41504E-75	0,502112903	0,872	0,83	1,4E-70	3
Ftl1	1,50755E-74	0,359526351	0,949	0,915	2,5E-70	3
Uqcr11	2,29303E-74	0,402911397	0,867	0,8	3,9E-70	3
Mmd	2,6267E-74	0,391449538	0,866	0,781	4,4E-70	3
Rps16	2,76444E-74	0,359197074	0,936	0,916	4,6E-70	3
Ppp1r14b	2,97697E-74	0,31166557	0,67	0,479	5E-70	3
Lsamp	5,79514E-74	0,336304562	0,817	0,674	9,7E-70	3
Rplp2	6,92078E-74	0,347462475	0,95	0,92	1,2E-69	3
Ndufb6	1,10982E-73	0,324720416	0,645	0,45	1,9E-69	3
Rps21	1,70904E-73	0,508721093	0,954	0,952	2,9E-69	3
Ndufb11	1,98222E-73	0,347964863	0,701	0,536	3,3E-69	3
Sfxn3	2,54653E-73	0,256546277	0,448	0,257	4,3E-69	3
Cetn2	2,94916E-73	0,348988393	0,776	0,636	5E-69	3
Atpif1	4,68106E-73	0,433089005	0,956	0,949	7,9E-69	3
Pla2g12a	6,3515E-73	0,253291361	0,397	0,215	1,1E-68	3
Cops6	8,28626E-73	0,352340415	0,677	0,511	1,4E-68	3
Gpr22	9,7335E-73	0,311829188	0,808	0,648	1,6E-68	3
Rpl19	3,14221E-72	0,43620807	0,94	0,93	5,3E-68	3
Vopp1	4,77028E-72	0,258069809	0,44	0,249	8E-68	3
Ehd3	5,56056E-72	0,420285298	0,732	0,594	9,3E-68	3
Junb	7,38218E-72	0,280062521	0,528	0,33	1,2E-67	3
Tlx2	1,71668E-71	0,256899228	0,985	0,958	2,9E-67	3
Mrps6	1,74857E-71	0,264217867	0,47	0,28	2,9E-67	3
Cox8a	1,84786E-71	0,331400902	0,95	0,918	3,1E-67	3
Clic1	2,23378E-71	0,305260665	0,887	0,806	3,8E-67	3

Vasp	2,5716E-71	0,280300466	0,571	0,379	4,3E-67	3
Mrpl20	3,4348E-71	0,333090152	0,561	0,374	5,8E-67	3
Prdx1	3,47575E-71	0,276889532	0,971	0,946	5,8E-67	3
Rpl10	3,48763E-71	0,352622045	0,906	0,859	5,9E-67	3
Rps12	9,60736E-71	0,386262915	0,861	0,803	1,6E-66	3
Lamp1	1,37362E-70	0,291634855	0,851	0,747	2,3E-66	3
Med28	1,7674E-70	0,297117064	0,613	0,424	3E-66	3
Cox6b1	2,09043E-70	0,358037928	0,9	0,852	3,5E-66	3
Rps7	2,24646E-70	0,410353014	0,831	0,753	3,8E-66	3
Coro1c	7,01859E-70	0,33240262	0,679	0,507	1,2E-65	3
Dync1i2	7,56156E-70	0,279778597	0,999	1	1,3E-65	3
Pde5a	8,64419E-70	0,254246948	0,426	0,241	1,5E-65	3
1500011K16Rik	8,73414E-70	0,271139111	0,479	0,29	1,5E-65	3
Tram1	1,22642E-69	0,274113434	0,465	0,282	2,1E-65	3
Cacna2d1	1,42061E-69	0,299807189	0,947	0,896	2,4E-65	3
Tmem160	1,78464E-69	0,316586055	0,578	0,392	3E-65	3
Ubr5	2,1227E-69	0,296444902	0,826	0,689	3,6E-65	3
Fscn1	2,55223E-69	0,317287218	0,737	0,567	4,3E-65	3
Cdk2ap1	3,13171E-69	0,25592546	0,414	0,236	5,3E-65	3
Snx6	3,80524E-69	0,275639273	0,515	0,319	6,4E-65	3
Dctn2	4,09313E-69	0,338563095	0,791	0,669	6,9E-65	3
Mageh1	4,5548E-69	0,262750895	0,53	0,338	7,6E-65	3
Usp46	5,48099E-69	0,278430974	0,599	0,405	9,2E-65	3
Fau	6,08346E-69	0,386807073	0,93	0,921	1E-64	3
Fbll1	1,35413E-68	0,259887048	0,43	0,249	2,3E-64	3
Eno1	6,06665E-68	0,308017706	0,676	0,501	1E-63	3
Hist1h4d	1,44519E-67	0,270082043	0,507	0,317	2,4E-63	3
Hsp90aa1	1,56889E-67	0,265097712	0,992	0,988	2,6E-63	3
Marveld1	5,77591E-67	0,261201159	0,433	0,254	9,7E-63	3
Inafm1	2,01654E-66	0,253448987	0,461	0,277	3,4E-62	3
Ptpra	2,23949E-66	0,285342035	0,795	0,65	3,8E-62	3
Negr1	2,39234E-66	0,269600791	0,979	0,954	4E-62	3
Fndc3a	2,56799E-66	0,294999061	0,712	0,545	4,3E-62	3
Cox6a1	3,73198E-66	0,338014941	0,885	0,82	6,3E-62	3
Pde10a	1,43072E-65	0,33021755	0,857	0,754	2,4E-61	3
Uqcrh	1,43765E-65	0,372202167	0,815	0,735	2,4E-61	3
Serpinb9	2,98055E-65	0,253468851	0,416	0,243	5E-61	3
Nop10	3,80879E-65	0,289760315	0,672	0,484	6,4E-61	3
Kctd13	4,49684E-65	0,252133457	0,528	0,339	7,6E-61	3
Chchd2	4,72783E-65	0,352591167	0,937	0,907	7,9E-61	3
Mien1	6,00215E-65	0,279381348	0,532	0,345	1E-60	3
D430019H16Rik	1,21602E-64	0,32678903	0,72	0,567	2E-60	3
Slc2a3	1,4375E-64	0,318889006	0,602	0,425	2,4E-60	3
Cyc1	1,59732E-64	0,282585842	0,561	0,373	2,7E-60	3
Atp5g3	1,68986E-64	0,292514247	0,853	0,755	2,8E-60	3
BC031181	3,46402E-64	0,291234764	0,82	0,703	5,8E-60	3

Cox7b	4,33102E-64	0,316699697	0,881	0,813	7,3E-60	3
Dcakd	4,37595E-64	0,254426257	0,467	0,287	7,3E-60	3
Atp2c1	5,93368E-64	0,283021465	0,689	0,511	1E-59	3
Stmn3	7,69745E-64	0,354734019	0,945	0,921	1,3E-59	3
Akirin1	1,26695E-63	0,28493391	0,706	0,542	2,1E-59	3
Rab2a	1,49935E-63	0,266009136	0,937	0,866	2,5E-59	3
Mill2	3,32634E-63	0,270562033	0,445	0,272	5,6E-59	3
Emc10	5,73954E-63	0,301203701	0,833	0,726	9,6E-59	3
Pde6d	8,78483E-63	0,253726745	0,565	0,377	1,5E-58	3
Rps13	1,05876E-62	0,369069031	0,861	0,788	1,8E-58	3
Rps9	2,60054E-62	0,359486926	0,934	0,908	4,4E-58	3
Tmem14c	2,75191E-62	0,257782072	0,429	0,258	4,6E-58	3
Slc48a1	1,25152E-61	0,260069963	0,63	0,447	2,1E-57	3
Sat1	1,28094E-61	0,258781609	0,499	0,321	2,2E-57	3
Scand1	1,60425E-61	0,300738245	0,779	0,643	2,7E-57	3
Prrc1	4,87828E-61	0,262599731	0,593	0,409	8,2E-57	3
Cuedc2	5,68593E-61	0,336217236	0,621	0,459	9,5E-57	3
Txnrd1	5,84409E-61	0,28106441	0,66	0,488	9,8E-57	3
Uqcrfs1	8,46662E-61	0,28407866	0,63	0,46	1,4E-56	3
Fgf1	1,25626E-60	0,302249507	0,738	0,582	2,1E-56	3
Rpl35	1,30065E-60	0,411738222	0,853	0,815	2,2E-56	3
Emd	1,38154E-60	0,2776936	0,62	0,44	2,3E-56	3
Vimp	1,5953E-60	0,260159654	0,519	0,338	2,7E-56	3
Mrpl57	2,16403E-60	0,266385046	0,542	0,365	3,6E-56	3
Doc2b	4,4399E-60	0,275132253	0,536	0,359	7,5E-56	3
Chmp2a	5,65856E-60	0,30232428	0,861	0,805	9,5E-56	3
Gm16286	9,33367E-60	0,256818404	0,524	0,349	1,6E-55	3
Rpl35a	2,25033E-59	0,331696492	0,938	0,915	3,8E-55	3
Myeov2	3,0241E-59	0,318753067	0,597	0,431	5,1E-55	3
Uba52	3,28476E-59	0,385223503	0,555	0,393	5,5E-55	3
Psmd6	4,3238E-59	0,288373116	0,626	0,452	7,3E-55	3
Ufm1	4,53578E-59	0,251732058	0,556	0,379	7,6E-55	3
Sdf2	6,61926E-59	0,277112319	0,603	0,436	1,1E-54	3
Rps8	2,67587E-58	0,33682334	0,978	0,979	4,5E-54	3
Trappc4	3,45021E-58	0,291278918	0,6	0,43	5,8E-54	3
Rps5	4,4439E-58	0,317869699	0,909	0,871	7,5E-54	3
Atp5l	4,87858E-58	0,29156164	0,933	0,888	8,2E-54	3
S100a16	6,26791E-58	0,279792098	0,931	0,892	1,1E-53	3
Imp3	7,02299E-58	0,268237807	0,523	0,355	1,2E-53	3
Ubb	9,07604E-58	0,362819099	0,989	0,986	1,5E-53	3
Rpl39	9,77076E-58	0,428453565	0,94	0,937	1,6E-53	3
Aig1	1,35822E-57	0,263054237	0,655	0,48	2,3E-53	3
Samd9l	2,39201E-57	0,250358475	0,59	0,412	4E-53	3
Ndufa4	2,46167E-57	0,352092066	0,87	0,819	4,1E-53	3
Ndufb8	7,85262E-57	0,308200479	0,671	0,51	1,3E-52	3
Ppib	8,60149E-57	0,303525952	0,682	0,535	1,4E-52	3

Atp5j2	1,02766E-56	0,380308804	0,828	0,77	1,7E-52	3
Rps10	1,02797E-56	0,325284672	0,933	0,914	1,7E-52	3
Ubl5	1,18737E-56	0,322575864	0,819	0,739	2E-52	3
Ywhah	1,20635E-56	0,267730977	0,992	0,988	2E-52	3
Tbc1d9	1,76181E-56	0,256881974	0,589	0,415	3E-52	3
Hint1	2,11217E-56	0,340926754	0,906	0,867	3,5E-52	3
Stmn4	3,48682E-56	0,292233096	0,545	0,384	5,9E-52	3
Tmem208	5,6635E-56	0,251793198	0,466	0,299	9,5E-52	3
Rpl11	6,95371E-56	0,279530205	0,96	0,952	1,2E-51	3
1810037I17Rik	1,06169E-55	0,270132774	0,695	0,538	1,8E-51	3
Sptssa	2,07408E-55	0,28068176	0,684	0,523	3,5E-51	3
Rps24	2,42037E-55	0,359641331	0,969	0,969	4,1E-51	3
Rpl34	2,64267E-55	0,321417578	0,932	0,909	4,4E-51	3
Vapb	3,05127E-55	0,269364804	0,8	0,673	5,1E-51	3
Cenpb	5,61935E-55	0,250802439	0,625	0,458	9,4E-51	3
Tbcb	5,73117E-55	0,303699292	0,768	0,669	9,6E-51	3
Necab2	7,13351E-55	0,289393972	0,555	0,388	1,2E-50	3
Mrpl41	7,2973E-55	0,251966134	0,575	0,406	1,2E-50	3
Ric8	1,12583E-54	0,255254966	0,577	0,402	1,9E-50	3
Pin4	1,50849E-54	0,27448468	0,553	0,382	2,5E-50	3
Atp5o	2,11787E-54	0,293102554	0,845	0,779	3,6E-50	3
Dlg2	2,55487E-54	0,28023206	0,795	0,674	4,3E-50	3
Atp5g2	3,92089E-54	0,272986118	0,884	0,817	6,6E-50	3
Adh5	5,55389E-54	0,279336965	0,477	0,311	9,3E-50	3
Rps28	9,58744E-54	0,365617659	0,852	0,806	1,6E-49	3
Ngfrap1	9,92714E-54	0,25374953	0,961	0,939	1,7E-49	3
Psma4	1,01388E-53	0,275446471	0,825	0,729	1,7E-49	3
Usmg5	1,01771E-53	0,271454899	0,891	0,826	1,7E-49	3
Mapre1	1,27165E-53	0,252058747	0,811	0,7	2,1E-49	3
0610012G03Rik	1,36462E-53	0,257169903	0,552	0,387	2,3E-49	3
Ccdc124	2,29941E-53	0,25058513	0,556	0,387	3,9E-49	3
Eif2s2	4,24301E-53	0,286009977	0,934	0,898	7,1E-49	3
Psmd12	5,84215E-53	0,266636562	0,685	0,531	9,8E-49	3
Edf1	1,0336E-52	0,294869124	0,762	0,661	1,7E-48	3
Eif3h	1,27515E-52	0,272870194	0,626	0,463	2,1E-48	3
Ndufb10	1,63313E-52	0,29756748	0,605	0,443	2,7E-48	3
Cmip	2,13586E-52	0,305556666	0,903	0,816	3,6E-48	3
Mrpl51	2,51648E-52	0,262731389	0,698	0,536	4,2E-48	3
Mab21l2	3,54722E-52	0,278817684	0,722	0,57	6E-48	3
Lamtor2	7,77262E-52	0,264973625	0,613	0,447	1,3E-47	3
Rps27a	1,19976E-51	0,332814757	0,936	0,926	2E-47	3
Cfap36	1,50613E-51	0,256592734	0,655	0,496	2,5E-47	3
Psmb5	1,62269E-51	0,297893484	0,819	0,752	2,7E-47	3
Ndufs4	1,97802E-51	0,283544114	0,643	0,488	3,3E-47	3
Rps27	2,6199E-51	0,424986434	0,877	0,862	4,4E-47	3
Hspa5	3,20829E-51	0,304508496	0,861	0,794	5,4E-47	3

Ndufa12	4,88615E-51	0,274623441	0,668	0,516	8,2E-47	3
Psma7	6,76254E-51	0,2869795	0,906	0,881	1,1E-46	3
1110008P14Rik	6,98952E-51	0,261352261	0,716	0,571	1,2E-46	3
Fkbp1b	1,01003E-50	0,253014975	0,552	0,389	1,7E-46	3
Rpl5	1,14251E-50	0,261883344	0,891	0,837	1,9E-46	3
Clu	1,26584E-50	0,304605864	0,507	0,347	2,1E-46	3
Rpl38	1,96806E-50	0,317430408	0,995	0,994	3,3E-46	3
Rps20	3,254E-50	0,303137978	0,916	0,89	5,5E-46	3
Rpl37a	3,95322E-50	0,315190213	0,988	0,987	6,6E-46	3
Psmb1	5,51757E-50	0,274514046	0,865	0,802	9,3E-46	3
Rpl17	7,81668E-50	0,299098531	0,928	0,912	1,3E-45	3
Fosl2	1,00645E-49	0,283840377	0,485	0,332	1,7E-45	3
Cox5b	1,48186E-49	0,276532401	0,845	0,775	2,5E-45	3
Uqcrq	1,66145E-49	0,278865705	0,89	0,841	2,8E-45	3
Dctn3	1,76688E-49	0,273848086	0,79	0,674	3E-45	3
Ndufs5	2,85251E-49	0,267061994	0,892	0,828	4,8E-45	3
Pgam1	3,5685E-49	0,27149599	0,85	0,776	6E-45	3
Atp5c1	8,08787E-49	0,296966195	0,68	0,548	1,4E-44	3
Rps25	1,19641E-48	0,261815019	0,885	0,835	2E-44	3
Mrps33	2,64505E-48	0,251561379	0,82	0,727	4,4E-44	3
Psmb4	3,13442E-48	0,251683163	0,838	0,747	5,3E-44	3
Ndufb2	6,3017E-48	0,289311868	0,731	0,615	1,1E-43	3
Nme1	7,33518E-48	0,273468898	0,737	0,61	1,2E-43	3
Rwdd1	8,52796E-48	0,252954383	0,636	0,485	1,4E-43	3
Txndc17	1,4386E-47	0,25112663	0,768	0,641	2,4E-43	3
Rpl9	1,73769E-47	0,379487211	0,835	0,809	2,9E-43	3
Atp5k	2,08057E-47	0,307414748	0,962	0,958	3,5E-43	3
Ndufc2	3,30812E-47	0,252301496	0,858	0,776	5,6E-43	3
Ndufb5	3,44326E-47	0,260092103	0,581	0,422	5,8E-43	3
Atp5f1	1,91398E-46	0,302437916	0,736	0,619	3,2E-42	3
Tomm7	2,9164E-46	0,290753524	0,814	0,742	4,9E-42	3
Rps3a1	4,00931E-46	0,360580806	0,856	0,815	6,7E-42	3
Ndufa7	7,20427E-46	0,289145062	0,753	0,66	1,2E-41	3
Ralgds	8,65349E-46	0,285881829	0,698	0,57	1,5E-41	3
Mrpl33	1,1196E-45	0,260563454	0,659	0,522	1,9E-41	3
Btf3	1,34309E-45	0,287423371	0,798	0,721	2,3E-41	3
Mat2a	1,93522E-45	0,279879984	0,738	0,612	3,2E-41	3
Tmsb10	3,85248E-45	0,279088858	0,962	0,948	6,5E-41	3
Rab3a	4,47427E-45	0,259915012	0,92	0,88	7,5E-41	3
Gnb2l1	5,58636E-45	0,277022426	0,844	0,775	9,4E-41	3
Serpinf1	9,25802E-45	0,282879121	0,316	0,188	1,6E-40	3
Rps3	2,20018E-44	0,307130844	0,886	0,854	3,7E-40	3
Sstr1	9,53802E-44	0,257949574	0,719	0,569	1,6E-39	3
Cplx2	2,12392E-43	0,293298805	0,689	0,561	3,6E-39	3
Tubb4b	3,08981E-43	0,321614486	0,815	0,768	5,2E-39	3
Rbm3	3,71683E-43	0,286127857	0,828	0,765	6,2E-39	3

Etnk1	1,15725E-42	0,265645032	0,906	0,849	1,9E-38	3
Rpl18	1,5912E-42	0,263664179	0,894	0,849	2,7E-38	3
Rpl7	7,16102E-42	0,301128563	0,803	0,74	1,2E-37	3
2010107E04Rik	1,36315E-41	0,281705443	0,719	0,609	2,3E-37	3
Ndufa6	4,78262E-41	0,253026768	0,706	0,585	8E-37	3
Fdps	7,5025E-41	0,290114081	0,802	0,729	1,3E-36	3
Rps29	1,14366E-40	0,290843092	0,998	0,996	1,9E-36	3
2410015M20Rik	1,94102E-40	0,250655946	0,627	0,495	3,3E-36	3
Pfdn5	8,17468E-40	0,278976152	0,789	0,729	1,4E-35	3
Rpl22l1	1,54807E-39	0,277406708	0,813	0,755	2,6E-35	3
Atp5h	2,0605E-39	0,264422461	0,887	0,864	3,5E-35	3
Rpl12	6,35129E-39	0,261967571	0,834	0,792	1,1E-34	3
Rplp0	7,79953E-39	0,278642906	0,813	0,751	1,3E-34	3
Tpt1	7,16635E-38	0,259276999	0,962	0,953	1,2E-33	3
Rpl3	1,65909E-37	0,282186028	0,921	0,927	2,8E-33	3
Ndufb9	1,10096E-36	0,268191344	0,694	0,598	1,8E-32	3
Rpl36a	2,27414E-36	0,293682182	0,794	0,742	3,8E-32	3
Gm4076	1,13958E-34	0,251388802	0,406	0,284	1,9E-30	3
Cox6c	3,02123E-34	0,255857408	0,888	0,862	5,1E-30	3
Cryab	3,81971E-34	0,273766102	0,481	0,356	6,4E-30	3
Atp5e	1,35137E-33	0,278141149	0,854	0,841	2,3E-29	3
Pnmal2	2,16247E-33	0,250713099	0,885	0,848	3,6E-29	3
Rpl29	2,55064E-33	0,257330068	0,674	0,571	4,3E-29	3
Pld3	2,66727E-33	0,256121527	0,849	0,811	4,5E-29	3
Polr2l	1,24011E-18	0,31203874	0,398	0,3	2,1E-14	3
