# Supplementary material for: The transcriptomes, connections and development of submucosal neuron classes in the mouse small intestine
Source: Nat Neurosci. 2025 May 29;28(6):1146–59. doi: 10.1038/s41593-025-01962-x (PMC12148937; doi:10.1038/s41593-025-01962-x)
Supplement: Supplementary file 10 — DE genes in subclusters of smENC1–smENC3. [file 41593_2025_1962_MOESM10_ESM.pdf]

Supplementary Table 3. Enriched genes in 9 subclusters of smENC1-3.

p-val: unadjusted p value; avg_logFC: log fold-change of the average expression betwee two groups. Positive values indicate high gene expression; pct.1: the percentage of cells where the gene is detected in the group; pct.2: The percentage of cells where the gene is detected in the rest of the dataset; p_val_adj: adjusted p-value based on Bonferroni correction using all genes in the dataset; Statistical analysis was two-sided but only enriched genes are shown. smENC: Enteric Neuron Class as defined in Figure 1i.
Top 30 genes for each subcluster are highlighted by their specific color (Fig. 1i).

Gene	p_val	avg_log2I	pct.1	pct.2	p_val_adj	cluster	smENC
Pcdh10	0	3,30089	0,99	0,17	0	1	smENC1
Serpine2	0	3,20192	0,966	0,292	0	1	
Adgrg6	0	3,00891	0,96	0,042	0	1	
Cbln2	0	2,9894	0,968	0,116	0	1	
Pde2a	0	2,11437	0,942	0,202	0	1	
Edn1	0	2,10006	0,946	0,193	0	1	
Tbx2	0	1,90347	0,924	0,287	0	1	
Dgkg	0	1,90239	0,893	0,139	0	1	
Nog	0	1,8683	0,845	0,079	0	1	
Cdkn1c	0	1,51017	0,674	0,028	0	1	
Cyp26b1	0	1,49713	0,584	0,021	0	1	
Syt15	0	1,47596	0,734	0,019	0	1	
Otof	0	1,45563	0,66	0,005	0	1	
Islr2	0	1,42421	0,74	0,084	0	1	
Tmeff2	0	1,41276	0,666	0,022	0	1	
Slc35d3	0	1,35718	0,674	0,005	0	1	
Nmu	0	1,34876	0,338	0,01	0	1	
Pkp1	0	1,22601	0,61	0,072	0	1	
Kctd12	0	1,13782	0,569	0,018	0	1	
Phgdh	0	1,06783	0,541	0,05	0	1	
Bcl11a	0	0,98352	0,563	0,042	0	1	
Dapk2	0	0,96439	0,598	0,077	0	1	
Slc25a48	0	0,85552	0,433	0,028	0	1	
Sgcz	0	0,85166	0,479	0,007	0	1	
Ptger3	0	0,78698	0,457	0,005	0	1	
Rgs6	0	0,59274	0,354	0,014	0	1	
C130060K24	0	0,59046	0,358	0,019	0	1	
Thsd7b	0	0,54451	0,326	0,012	0	1	
Gpr85	1,2639E-303	1,87516	0,93	0,324	2,1222E-299	1	
Htr3b	2,3244E-289	0,84517	0,509	0,056	3,9028E-285	1	
Sulf2	1,7805E-283	1,50977	0,859	0,233	2,9896E-279	1	
Avil	1,1971E-272	1,15999	0,61	0,094	2,0101E-268	1	
Nrxn3	2,5003E-270	1,77023	0,978	0,431	4,1982E-266	1	
Zfp804a	3,4762E-264	1,61318	0,827	0,239	5,8369E-260	1	
Efr3a	2,617E-252	1,99915	0,924	0,431	4,3943E-248	1	
6330403A02	2,587E-251	1,86555	1	0,934	4,3438E-247	1	
Lhfpl2	2,1716E-249	1,78743	0,954	0,481	3,6464E-245	1	
Ccbe1	3,5795E-249	1,14245	0,706	0,151	6,0103E-245	1	
Casz1	2,7115E-246	1,58446	0,934	0,349	4,5529E-242	1	

Ntrk3	1,0148E-241	1,74004	1	0,7	1,7039E-237	1	
Tcf7l2	4,8941E-241	1,52283	1	0,836	8,2178E-237	1	
Ptgfr	1,1363E-240	1,17357	0,7	0,152	1,908E-236	1	
Id4	1,2812E-238	1,14959	0,662	0,137	2,1513E-234	1	
Cnr1	2,1765E-237	1,3549	1	0,997	3,6545E-233	1	
Zeb2	1,684E-236	1,72681	0,986	0,63	2,8277E-232	1	
Bmp4	1,9467E-228	0,99777	0,614	0,117	3,2686E-224	1	
Cysltr2	7,9664E-227	1,08144	0,672	0,15	1,3376E-222	1	
Shf	8,682E-224	0,56653	0,382	0,039	1,4578E-219	1	
Esr1	8,1916E-218	0,40731	0,264	0,016	1,3754E-213	1	
Rab3c	1,3596E-216	1,31775	1	0,995	2,2829E-212	1	
Hpcal1	1,6685E-216	1,41122	0,779	0,25	2,8016E-212	1	
Trp53i11	4,3764E-216	1,66888	1	0,65	7,3484E-212	1	
Tubb3	2,3039E-210	1,14671	1	0,999	3,8684E-206	1	
Proser2	6,9736E-210	0,97591	0,581	0,114	1,1709E-205	1	
Pcdh9	1,0999E-207	1,3343	0,781	0,251	1,8469E-203	1	
S100a11	1,9969E-207	1,65315	0,992	0,854	3,3529E-203	1	
Hey1	2,4573E-207	0,84992	0,545	0,098	4,126E-203	1	
Rims1	2,1777E-206	0,83385	0,557	0,103	3,6566E-202	1	
Phox2b	9,1576E-201	1,18842	1	0,993	1,5377E-196	1	
Serpina3n	1,5419E-194	0,82999	0,483	0,08	2,589E-190	1	
Wif1	6,3104E-194	0,88974	0,567	0,112	1,0596E-189	1	
Krt19	1,2742E-193	1,97467	0,863	0,328	2,1395E-189	1	
Pdzd2	3,8689E-192	0,84149	0,543	0,103	6,4962E-188	1	
Fam19a1	6,2597E-192	1,31727	0,915	0,432	1,0511E-187	1	
Fam129a	6,3535E-192	0,44588	0,29	0,024	1,0668E-187	1	
Chst15	1,3241E-187	1,04115	0,72	0,2	2,2234E-183	1	
Snhg11	1,6808E-184	1,23368	1	0,994	2,8223E-180	1	
Gabbr2	2,8974E-183	0,54705	0,368	0,045	4,8651E-179	1	
Timp3	4,6459E-183	1,57709	0,936	0,449	7,8009E-179	1	
Hes1	1,8181E-180	0,96735	0,408	0,06	3,0528E-176	1	
Cpne4	5,3023E-178	1,21054	0,988	0,822	8,9032E-174	1	
Psd3	6,5331E-173	1,19429	0,714	0,228	1,097E-168	1	
Hoxb5	1,5462E-170	1,14141	1	0,956	2,5963E-166	1	
Ngfr	2,4929E-163	1,33544	0,974	0,553	4,1858E-159	1	
Cntn5	1,9111E-161	1,10359	0,714	0,237	3,2089E-157	1	
Fgf13	5,8135E-161	1,0445	1	0,992	9,7614E-157	1	
Dgat2	2,3204E-158	0,64951	0,453	0,085	3,8962E-154	1	
9530059O14	9,4252E-158	1,35081	0,982	0,756	1,5826E-153	1	
Tcerg1l	4,387E-157	0,98628	0,668	0,203	7,3662E-153	1	
Gucy1a3	3,3571E-156	1,25323	0,877	0,493	5,6369E-152	1	
Myl1	1,53E-155	1,09285	1	0,935	2,5691E-151	1	
Nedd4l	1,0781E-154	1,10789	0,863	0,45	1,8102E-150	1	
Hcn1	9,1032E-154	0,49446	0,31	0,038	1,5285E-149	1	
Dlx3	1,1653E-152	0,86465	0,563	0,141	1,9566E-148	1	
Prom1	3,582E-145	0,63328	0,453	0,09	6,0145E-141	1	
Sncg	9,4505E-144	1,2897	1	0,943	1,5868E-139	1	
Krt15	2,3193E-143	0,65011	0,394	0,069	3,8944E-139	1	

Ank2	2,6092E-142	0,79245	1	0,998	4,3811E-138	1	
Tubb5	1,8096E-140	0,77153	1	1	3,0386E-136	1	
Tmem229b	4,708E-140	1,20703	0,911	0,529	7,9052E-136	1	
A330102I10I	7,3557E-140	0,3942	0,274	0,032	1,2351E-135	1	
Rph3a	4,5089E-139	1,19491	0,95	0,638	7,5709E-135	1	
Syt2	4,1697E-138	1,07216	0,934	0,654	7,0014E-134	1	
Iqgap2	1,815E-136	0,65378	0,445	0,093	3,0476E-132	1	
Zfhx3	1,6355E-135	0,73289	0,477	0,109	2,7462E-131	1	
Calb2	2,4735E-134	1,07198	1	0,978	4,1532E-130	1	
Ptpru	6,0903E-133	0,77104	0,517	0,132	1,0226E-128	1	
Bche	1,0161E-132	1,14711	0,986	0,784	1,7061E-128	1	
Nkd1	1,2428E-132	0,60917	0,463	0,102	2,0868E-128	1	
Gse1	1,5253E-131	1,03026	0,988	0,823	2,5612E-127	1	
Adamts14	5,5635E-131	0,68846	0,519	0,132	9,3416E-127	1	
Grin3a	7,8792E-130	1,10066	0,748	0,324	1,323E-125	1	
Cacna1e	4,0592E-129	1,05134	0,885	0,488	6,8158E-125	1	
Hspb1	4,7872E-129	1,17585	0,704	0,28	8,0382E-125	1	
mt-Nd2	1,2932E-128	0,7564	1	1	2,1714E-124	1	
Robo2	6,3444E-128	0,95961	0,757	0,321	1,0653E-123	1	
Rbfox3	1,8403E-126	0,89595	0,718	0,273	3,09E-122	1	
Fmnl2	1,902E-125	0,9934	0,825	0,435	3,1936E-121	1	
Epb41l1	2,6618E-124	0,96101	0,946	0,76	4,4694E-120	1	
mt-Atp6	3,2774E-124	0,57697	1	1	5,5031E-120	1	
Snrpn	3,7847E-124	0,88701	1	0,983	6,3549E-120	1	
Arhgap6	3,9935E-121	0,61551	0,463	0,112	6,7055E-117	1	
Cacna1a	1,2057E-120	1,06616	0,887	0,574	2,0244E-116	1	
Slitrk3	4,9936E-120	0,9012	0,668	0,25	8,3848E-116	1	
Adcy1	2,993E-119	0,91731	0,744	0,309	5,0255E-115	1	
Thbs1	5,948E-119	0,71742	0,443	0,104	9,9873E-115	1	
Tmem63b	2,6239E-118	0,92158	0,972	0,745	4,4058E-114	1	
Lzts1	2,1281E-116	0,64041	0,475	0,122	3,5733E-112	1	
mt-Nd4	3,4711E-116	0,57325	1	1	5,8283E-112	1	
Ccser2	5,3773E-116	0,97681	0,97	0,811	9,029E-112	1	
Apba1	8,2362E-116	0,93344	0,962	0,747	1,3829E-111	1	
Adora1	1,0278E-115	0,50175	0,354	0,066	1,7258E-111	1	
Prkca	3,9438E-111	0,39932	0,288	0,046	6,622E-107	1	
L1cam	6,1985E-111	0,78103	1	0,981	1,0408E-106	1	
Tmeff1	3,2662E-110	1,11901	0,815	0,489	5,4842E-106	1	
Mapk3	1,494E-109	0,86149	1	0,991	2,5086E-105	1	
Sgcd	3,0303E-108	0,64055	0,491	0,136	5,0882E-104	1	
Scube1	1,159E-107	0,88094	0,996	0,904	1,946E-103	1	
Dleu7	4,9747E-107	0,7287	0,431	0,11	8,353E-103	1	
Rab3b	7,3553E-107	1,06013	0,827	0,445	1,235E-102	1	
Zbtb7c	1,7182E-106	0,83801	0,676	0,281	2,8851E-102	1	
Ephb2	2,0249E-106	0,56274	0,437	0,111	3,4001E-102	1	
Nfia	4,3854E-106	0,98995	0,853	0,518	7,3634E-102	1	
Ctbp1	2,6696E-104	0,83674	0,984	0,873	4,4825E-100	1	
Smad6	5,14E-104	0,69222	0,531	0,168	8,6306E-100	1	

Slc35g1	4,571E-102	0,62255	0,467	0,13	7,67522E-98	1	
Tln2	9,3817E-102	0,87616	0,95	0,804	1,57529E-97	1	
Cux2	7,4816E-100	0,87656	0,871	0,578	1,25624E-95	1	
Thra	4,2403E-99	0,65908	1	0,993	7,11985E-95	1	
Maz	6,2314E-99	0,81942	0,978	0,866	1,04631E-94	1	
Maoa	1,68094E-97	0,91358	0,926	0,73	2,82246E-93	1	
Lrrc75b	3,42723E-97	0,86793	0,781	0,426	5,75466E-93	1	
Sbk1	4,18258E-97	0,65715	0,507	0,161	7,02296E-93	1	
Chd3	6,09916E-97	0,73484	0,992	0,951	1,02411E-92	1	
Pvrl3	6,07009E-96	0,3633	0,262	0,043	1,01923E-91	1	
Plcb3	1,04554E-95	0,86034	0,722	0,369	1,75557E-91	1	
Slitrk2	5,34675E-95	0,45267	0,324	0,067	8,97772E-91	1	
Zbtb18	2,19536E-93	0,92304	0,857	0,606	3,68622E-89	1	
Slc1a7	3,63101E-92	0,52682	0,417	0,111	6,09683E-88	1	
Gm10600	8,50113E-92	0,38653	0,29	0,055	1,42742E-87	1	
Lynx1	1,33569E-91	0,90378	0,628	0,263	2,24275E-87	1	
Mcf2l	1,65443E-91	0,74763	0,757	0,397	2,77795E-87	1	
Neat1	2,92167E-91	1,09603	0,642	0,276	4,90578E-87	1	
Fam117a	1,9076E-90	0,53475	0,35	0,082	3,20305E-86	1	
Tuba1b	5,15888E-90	0,70782	0,996	0,993	8,66227E-86	1	
Slc52a3	6,72833E-90	0,47344	0,326	0,071	1,12975E-85	1	
mt-Nd4l	7,9009E-90	0,7142	1	1	1,32664E-85	1	
Ptprt	9,26396E-90	0,64827	0,543	0,182	1,55551E-85	1	
Col5a3	4,069E-89	0,40642	0,308	0,062	6,83226E-85	1	
Ywhab	3,14123E-88	0,67116	0,998	0,993	5,27444E-84	1	
Vamp1	8,18056E-88	0,9537	0,96	0,801	1,3736E-83	1	
Pbx1	1,324E-87	0,82963	0,932	0,756	2,22313E-83	1	
mt-Atp8	1,63323E-86	0,7622	1	1	2,74236E-82	1	
Msn	4,20775E-86	0,72141	0,974	0,874	7,06524E-82	1	
mt-Cytb	1,02938E-85	0,41769	1	1	1,72843E-81	1	
Flot2	1,10383E-85	0,76474	0,986	0,921	1,85344E-81	1	
Calm3	3,85524E-84	0,7341	0,974	0,879	6,47334E-80	1	
P2rx2	5,3208E-83	0,81968	0,998	0,983	8,93416E-79	1	
Lingo1	5,98101E-83	0,54448	0,402	0,113	1,00427E-78	1	
Ptprd	9,2976E-81	0,71259	0,972	0,88	1,56116E-76	1	
Nos1ap	1,23011E-80	0,6087	0,541	0,201	2,06548E-76	1	
Hoxc4	2,63826E-80	0,68456	0,986	0,906	4,4299E-76	1	
Atoh8	5,24074E-80	0,64759	0,505	0,186	8,79973E-76	1	
Nfic	6,67065E-80	0,65704	0,99	0,95	1,12007E-75	1	
Ppp2r1a	4,35883E-79	0,66608	0,996	0,953	7,31892E-75	1	
Sgms1	6,21823E-79	0,47327	0,34	0,086	1,0441E-74	1	
Fam171b	6,90816E-79	0,76099	0,946	0,782	1,15995E-74	1	
Elavl3	8,83948E-79	0,69141	0,988	0,92	1,48424E-74	1	
Tsc22d1	7,21752E-78	0,72224	0,976	0,896	1,21189E-73	1	
Tmem178b	8,1147E-78	0,86188	0,821	0,547	1,36254E-73	1	
Eml2	3,54145E-77	0,77607	0,833	0,609	5,94645E-73	1	
Kif26b	2,43537E-76	0,52297	0,437	0,139	4,08922E-72	1	
5330434G04	3,04007E-76	0,76331	0,942	0,789	5,10458E-72	1	

Cntn1	3,13839E-75	0,79677	0,897	0,716	5,26967E-71	1	
Syt9	2,73008E-73	0,8061	0,765	0,492	4,58408E-69	1	
Actb	4,21429E-73	0,61649	1	1	7,07622E-69	1	
mt-Nd1	2,65318E-72	0,51108	1	1	4,45496E-68	1	
Aatk	7,12826E-71	0,72315	0,861	0,597	1,19691E-66	1	
Mpp2	7,73217E-71	0,8025	0,702	0,409	1,29831E-66	1	
Mex3b	1,17301E-70	0,49586	0,41	0,132	1,9696E-66	1	
Plekhm3	1,54279E-70	0,73446	0,799	0,53	2,59049E-66	1	
Klf6	1,75377E-70	0,81582	0,712	0,422	2,94475E-66	1	
Tmem189	5,5715E-70	0,72575	0,646	0,34	9,35511E-66	1	
Cbx6	3,86508E-69	0,64608	0,998	0,944	6,48985E-65	1	
Pak7	6,58335E-69	0,63811	0,481	0,187	1,10541E-64	1	
Tmem64	6,72996E-69	0,66357	0,966	0,888	1,13003E-64	1	
Smad7	6,73444E-69	0,77074	0,734	0,448	1,13078E-64	1	
Ssbp3	1,24685E-68	0,61001	0,994	0,949	2,09359E-64	1	
Adra2a	3,04714E-68	0,68713	0,579	0,258	5,11646E-64	1	
Kif26a	6,50773E-68	0,69142	0,917	0,701	1,09271E-63	1	
Il10rb	1,24282E-67	0,71589	0,66	0,348	2,08682E-63	1	
Pik3r1	1,82349E-67	0,7035	0,831	0,602	3,06182E-63	1	
Crmp1	1,89133E-67	0,65969	0,982	0,902	3,17573E-63	1	
Mgat4b	4,54892E-67	0,72071	0,763	0,488	7,63808E-63	1	
Gas6	6,04181E-67	0,52381	0,455	0,166	1,01448E-62	1	
Slc4a4	1,60201E-66	0,64598	0,948	0,803	2,68994E-62	1	
Fam102b	5,95119E-66	0,70936	0,676	0,376	9,99264E-62	1	
Jph3	6,90858E-66	0,708	0,853	0,662	1,16002E-61	1	
Rhobtb1	2,11656E-65	0,3191	0,29	0,072	3,55391E-61	1	
Scn3a	3,8903E-65	0,71304	0,952	0,823	6,5322E-61	1	
Medag	7,03449E-65	0,42563	0,268	0,065	1,18116E-60	1	
mt-Nd5	2,16753E-64	0,64169	1	1	3,63951E-60	1	
Flrt3	3,70232E-64	0,38898	0,28	0,07	6,21657E-60	1	
Tacc2	7,89743E-64	0,64435	0,565	0,263	1,32606E-59	1	
Paqr8	8,14751E-64	0,68564	0,759	0,494	1,36805E-59	1	
Cyfip2	1,24173E-63	0,68989	0,841	0,653	2,085E-59	1	
Ano6	2,71791E-63	0,63443	0,942	0,796	4,56364E-59	1	
Dclk3	7,24838E-63	0,39448	0,318	0,089	1,21708E-58	1	
Cxxc5	5,53939E-62	0,58745	0,958	0,847	9,30119E-58	1	
Gnb2	7,78928E-62	0,41613	1	1	1,3079E-57	1	
Mgat4a	7,83882E-62	0,65504	0,6	0,298	1,31622E-57	1	
Cbfa2t3	1,58129E-61	0,4624	0,332	0,098	2,65514E-57	1	
Igf1r	2,04114E-61	0,70215	0,837	0,63	3,42729E-57	1	
Mt3	2,69989E-61	0,8249	0,934	0,724	4,53339E-57	1	
Kcnq1ot1	5,49621E-61	0,95706	0,95	0,851	9,22869E-57	1	
Slc44a1	6,34826E-61	0,74517	0,857	0,649	1,06594E-56	1	
Syne2	1,41743E-60	0,60195	0,487	0,202	2,38E-56	1	
Ppp3ca	1,11618E-59	0,53673	0,996	0,965	1,87418E-55	1	
Nt5dc3	2,04661E-59	0,73274	0,849	0,695	3,43647E-55	1	
Klc1	2,20152E-59	0,47239	1	1	3,69657E-55	1	
Sntg1	4,10527E-59	0,57479	0,505	0,215	6,89316E-55	1	

Pex5l	4,15187E-59	0,64625	0,716	0,437	6,97141E-55	1	
Mtus1	4,63251E-59	0,66214	0,785	0,548	7,77845E-55	1	
Dlgap3	5,89555E-58	0,70314	0,708	0,443	9,89921E-54	1	
Zfp423	6,00839E-58	0,34241	0,27	0,07	1,00887E-53	1	
Cd164	1,67578E-57	0,70153	0,644	0,381	2,8138E-53	1	
Dgki	1,67816E-57	0,60834	0,575	0,285	2,8178E-53	1	
Zmynd11	3,96806E-57	0,60898	0,891	0,714	6,66277E-53	1	
Susd2	8,81452E-57	0,65404	0,541	0,253	1,48005E-52	1	
Plec	1,61715E-56	0,58965	0,958	0,853	2,71536E-52	1	
Cnnm4	2,43184E-56	0,56999	0,457	0,189	4,0833E-52	1	
Akt1	1,08177E-55	0,61335	0,877	0,697	1,81639E-51	1	
Klf13	1,42004E-55	0,65577	0,871	0,714	2,38439E-51	1	
Clock	8,47214E-55	0,72752	0,763	0,537	1,42256E-50	1	
Foxo6	1,26845E-54	0,40111	0,338	0,11	2,12985E-50	1	
Ahnak	5,03991E-54	0,70677	0,744	0,482	8,46251E-50	1	
Atp2b1	6,28948E-54	0,62178	0,885	0,746	1,05607E-49	1	
Galnt1	1,33486E-53	0,63981	0,692	0,438	2,24136E-49	1	
Ank3	1,34682E-53	0,64805	0,718	0,46	2,26145E-49	1	
Atp2b2	1,56741E-53	0,64292	0,73	0,475	2,63183E-49	1	
Pcdh1	1,82749E-53	0,61264	0,847	0,674	3,06854E-49	1	
Wnt9a	2,95491E-53	0,41159	0,358	0,123	4,96159E-49	1	
Tnk2	4,78781E-53	0,65267	0,769	0,539	8,0392E-49	1	
Ap2s1	9,70988E-53	0,56867	0,978	0,938	1,63039E-48	1	
Fxyd7	2,56631E-52	0,59407	0,996	0,745	4,30909E-48	1	
Adgrl2	3,06067E-52	0,64061	0,674	0,406	5,13916E-48	1	
Rbfox1	3,73222E-52	0,62107	0,847	0,662	6,26677E-48	1	
Prkar1b	6,82211E-52	0,57625	0,954	0,817	1,1455E-47	1	
Shoc2	5,03445E-51	0,63946	0,777	0,569	8,45334E-47	1	
Srrm2	5,22259E-51	0,60497	0,99	0,968	8,76924E-47	1	
Frmd4b	1,00575E-50	0,50931	0,483	0,213	1,68875E-46	1	
Map1b	1,0218E-50	0,47031	1	1	1,7157E-46	1	
Jakmip1	1,58305E-50	0,62665	0,706	0,465	2,6581E-46	1	
Nav1	1,59942E-50	0,54797	0,978	0,913	2,68559E-46	1	
Aff2	2,51121E-50	0,41182	0,338	0,117	4,21657E-46	1	
Ppp2r2c	3,08032E-50	0,5635	0,942	0,799	5,17217E-46	1	
Stxbp5	4,66895E-50	0,58349	0,883	0,682	7,83964E-46	1	
App	7,51117E-50	0,4295	0,996	0,994	1,2612E-45	1	
Smarcc2	9,34654E-50	0,61992	0,881	0,722	1,56938E-45	1	
Atxn7l3	2,65019E-49	0,61538	0,732	0,507	4,44993E-45	1	
Gcnt2	2,68627E-49	0,64531	0,807	0,634	4,51052E-45	1	
Map1a	3,80551E-49	0,62578	0,948	0,87	6,38984E-45	1	
Lrrn2	6,07172E-49	0,57576	0,773	0,529	1,0195E-44	1	
Necab1	1,14912E-48	0,52878	0,964	0,868	1,92949E-44	1	
Cntfr	2,73681E-48	0,51942	0,509	0,251	4,59537E-44	1	
Sdc3	3,6694E-48	0,57413	0,952	0,82	6,16129E-44	1	
Thsd4	5,50034E-48	0,38102	0,346	0,12	9,23562E-44	1	
Arl8a	9,4862E-48	0,47156	0,992	0,985	1,59283E-43	1	
Them6	9,70474E-48	0,69485	0,586	0,347	1,62952E-43	1	

Clic5	1,26533E-47	0,46033	0,461	0,203	2,12462E-43	1	
Anp32b	1,5032E-47	0,62404	0,807	0,645	2,52402E-43	1	
Ino80d	1,75865E-47	0,58684	0,775	0,542	2,95295E-43	1	
Cacna1h	4,031E-47	0,4098	0,38	0,145	6,76846E-43	1	
Sh3bgrl2	6,74903E-47	0,37192	0,302	0,1	1,13323E-42	1	
mt-Nd3	6,88896E-47	0,44753	1	1	1,15673E-42	1	
Agrn	1,6672E-46	0,61374	0,485	0,235	2,7994E-42	1	
Klf7	1,66843E-46	0,48613	0,986	0,947	2,80146E-42	1	
Map3k1	2,71132E-46	0,59141	0,708	0,471	4,55258E-42	1	
Clgn	3,8274E-46	0,51516	0,467	0,22	6,42659E-42	1	
Kcnk3	8,17803E-46	0,5132	0,465	0,217	1,37317E-41	1	
Myadm	3,7557E-45	0,49859	0,964	0,831	6,3062E-41	1	
Cadm3	5,15638E-45	0,53216	0,907	0,755	8,65808E-41	1	
Hivep3	5,23987E-45	0,53744	0,771	0,561	8,79826E-41	1	
Plcl1	9,62062E-45	0,33688	0,326	0,115	1,6154E-40	1	
Prkag2	1,1303E-44	0,55997	0,624	0,376	1,89789E-40	1	
Maob	1,10613E-43	0,50625	0,515	0,26	1,8573E-39	1	
Mbp	1,83174E-43	0,47048	0,439	0,196	3,07568E-39	1	
Pnisr	2,27945E-43	0,56143	0,879	0,73	3,82742E-39	1	
Hmbox1	1,62385E-42	0,55646	0,732	0,524	2,7266E-38	1	
2610001J05F	2,70176E-42	0,53403	0,559	0,314	4,53653E-38	1	
Gdpd5	1,11085E-41	0,4808	0,515	0,269	1,86523E-37	1	
Rbms3	1,66468E-41	0,42657	1	0,992	2,79516E-37	1	
Cacnb1	1,73393E-41	0,55261	0,775	0,594	2,91145E-37	1	
Nyap2	5,06169E-41	0,32603	0,298	0,105	8,49908E-37	1	
Ppp3cb	5,08099E-41	0,54098	0,841	0,694	8,5315E-37	1	
Plxna4	5,53215E-41	0,43686	0,996	0,965	9,28904E-37	1	
Nrxn2	5,90586E-41	0,44347	0,996	0,961	9,91654E-37	1	
Arap1	9,8915E-41	0,40673	0,398	0,173	1,66088E-36	1	
Il13ra1	1,78773E-40	0,577	0,612	0,394	3,00178E-36	1	
mt-Co2	2,25763E-40	0,27134	1	1	3,79078E-36	1	
Hs3st5	2,56407E-40	0,46825	0,435	0,204	4,30533E-36	1	
F2r	3,50887E-40	0,53656	0,873	0,579	5,89174E-36	1	
Fryl	4,13427E-40	0,47559	0,523	0,28	6,94184E-36	1	
Zfp704	1,02257E-39	0,52341	0,596	0,353	1,717E-35	1	
Nek1	1,07638E-39	0,56186	0,746	0,536	1,80735E-35	1	
Unc5b	1,43431E-39	0,55167	0,716	0,501	2,40835E-35	1	
B3gnt8	1,75129E-39	0,29103	0,296	0,105	2,94059E-35	1	
Apba2	2,53838E-39	0,57585	0,869	0,713	4,26219E-35	1	
Tcf7	3,18107E-39	0,36465	0,362	0,149	5,34133E-35	1	
1700025G04	3,94915E-39	0,30826	0,272	0,092	6,63102E-35	1	
Tspan9	4,42595E-39	0,54172	0,696	0,46	7,43162E-35	1	
Hoxb6	5,11676E-39	0,53027	0,588	0,339	8,59156E-35	1	
Marcks	5,428E-39	0,44924	0,958	0,892	9,11415E-35	1	
Chd4	6,11703E-39	0,49476	0,932	0,854	1,02711E-34	1	
Ino80dos	7,51415E-39	0,46567	0,465	0,235	1,2617E-34	1	
Cic	8,94884E-39	0,58221	0,67	0,487	1,5026E-34	1	
Fam124a	9,83482E-39	0,29596	0,274	0,093	1,65136E-34	1	

Dclk1	7,04935E-38	0,5105	0,905	0,793	1,18366E-33	1	
Tnks1bp1	8,50317E-38	0,45786	0,527	0,289	1,42777E-33	1	
Asap1	1,20338E-37	0,48743	0,632	0,405	2,0206E-33	1	
Zfp618	1,23818E-37	0,49572	0,561	0,323	2,07902E-33	1	
Glce	1,29126E-37	0,3823	0,368	0,157	2,16816E-33	1	
Ap1s2	1,29343E-37	0,5783	0,638	0,435	2,1718E-33	1	
Spock2	1,40764E-37	0,37083	1	0,978	2,36357E-33	1	
Pcbp4	1,77868E-37	0,47197	0,875	0,75	2,98658E-33	1	
Fus	2,82019E-37	0,4582	0,984	0,941	4,73537E-33	1	
Tub	3,22667E-37	0,52381	0,887	0,762	5,4179E-33	1	
Grem2	5,97771E-37	0,41217	0,276	0,101	1,00372E-32	1	
Mesdc1	1,54855E-36	0,38774	0,388	0,175	2,60018E-32	1	
Fam49a	1,61755E-36	0,57465	0,644	0,432	2,71603E-32	1	
Csnk1d	1,82902E-36	0,50962	0,817	0,64	3,07111E-32	1	
Npc2	6,69033E-36	0,52413	0,823	0,706	1,12337E-31	1	
Nfib	6,78525E-36	0,46306	0,952	0,879	1,13931E-31	1	
Gan	8,22365E-36	0,49392	0,505	0,28	1,38083E-31	1	
Gpc1	9,41291E-36	0,6003	0,612	0,419	1,58052E-31	1	
Ankrd11	1,1499E-35	0,45538	0,948	0,87	1,93079E-31	1	
Man1c1	2,13496E-35	0,38421	0,378	0,169	3,58481E-31	1	
Slc29a4	2,29635E-35	0,52904	0,583	0,367	3,85581E-31	1	
Chl1	3,82272E-35	0,48332	0,92	0,772	6,41872E-31	1	
Tnr	4,80905E-35	0,33402	0,268	0,098	8,07487E-31	1	
Zmiz1	5,81296E-35	0,45272	0,875	0,721	9,76054E-31	1	
Bsn	9,22119E-35	0,51374	0,698	0,515	1,54833E-30	1	
Asap2	1,45583E-34	0,43049	0,485	0,263	2,44449E-30	1	
Srgap3	2,6632E-34	0,47375	0,841	0,67	4,47177E-30	1	
Ano2	2,79944E-34	0,58504	0,636	0,436	4,70054E-30	1	
Gnaq	3,24096E-34	0,44254	0,899	0,8	5,44189E-30	1	
Btbd2	4,67309E-34	0,52846	0,698	0,497	7,84658E-30	1	
Sh3pxd2a	6,2251E-34	0,50259	0,763	0,606	1,04526E-29	1	
Igsf3	7,16102E-34	0,49323	0,74	0,528	1,20241E-29	1	
Rnaseh2b	7,649E-34	0,3473	0,346	0,151	1,28434E-29	1	
Spsb1	9,41255E-34	0,35211	0,304	0,122	1,58046E-29	1	
Dlg4	9,42232E-34	0,50722	0,769	0,607	1,5821E-29	1	
Ebf4	1,55947E-33	0,30535	0,282	0,108	2,6185E-29	1	
Adgrb1	2,69144E-33	0,51483	0,706	0,52	4,5192E-29	1	
Cachd1	7,1155E-33	0,35673	0,398	0,186	1,19476E-28	1	
Ei24	7,59459E-33	0,68242	0,744	0,641	1,27521E-28	1	
Pald1	7,78947E-33	0,31097	0,308	0,126	1,30793E-28	1	
Rybp	2,04434E-32	0,46619	0,658	0,45	3,43265E-28	1	
Pip4k2b	2,19838E-32	0,50202	0,668	0,486	3,6913E-28	1	
Brd3	2,52912E-32	0,49919	0,757	0,618	4,24664E-28	1	
Kcna2	3,00076E-32	0,29651	0,284	0,111	5,03858E-28	1	
Scn5a	4,76148E-32	0,47434	0,803	0,658	7,995E-28	1	
Eif4a1	6,65699E-32	0,37344	0,99	0,977	1,11778E-27	1	
Ubtf	7,27076E-32	0,46592	0,815	0,689	1,22083E-27	1	
Cacng4	1,18523E-31	0,46019	0,628	0,4	1,99011E-27	1	

Robo1	1,63978E-31	0,4997	0,71	0,549	2,75336E-27	1	
Gdi1	2,9948E-31	0,3787	0,992	0,975	5,02857E-27	1	
Rgs9	3,49226E-31	0,44652	0,889	0,75	5,86385E-27	1	
Cacna1b	3,88448E-31	0,45452	0,813	0,668	6,52244E-27	1	
Arid1a	4,05409E-31	0,48739	0,694	0,507	6,80722E-27	1	
Kcnn3	5,08179E-31	0,44715	0,73	0,543	8,53283E-27	1	
Ppp1r9b	6,84823E-31	0,49936	0,724	0,568	1,14989E-26	1	
Arl5a	7,74511E-31	0,51142	0,59	0,389	1,30048E-26	1	
Astn2	8,96539E-31	0,45531	0,567	0,348	1,50538E-26	1	
Sox4	1,39714E-30	0,41525	0,946	0,863	2,34594E-26	1	
Camta1	2,2448E-30	0,42416	0,934	0,864	3,76924E-26	1	
Kcnq3	3,12044E-30	0,48962	0,753	0,583	5,23952E-26	1	
Lars2	4,59824E-30	0,4103	0,996	0,989	7,7209E-26	1	
Hspb8	9,02763E-30	0,43704	0,986	0,959	1,51583E-25	1	
Sgpl1	9,03016E-30	0,48131	0,553	0,363	1,51625E-25	1	
Mtcl1	1,10344E-29	0,50108	0,658	0,492	1,85278E-25	1	
Ppfia2	1,721E-29	0,3475	0,453	0,242	2,88974E-25	1	
Nxpe3	5,16092E-29	0,31514	0,314	0,139	8,6657E-25	1	
Nfat5	8,273E-29	0,45061	0,736	0,567	1,38912E-24	1	
Elavl4	9,16729E-29	0,32639	0,996	0,998	1,53928E-24	1	
Dkk3	1,28001E-28	0,44906	0,501	0,299	2,14927E-24	1	
Soga3	1,59646E-28	0,40919	0,95	0,857	2,68062E-24	1	
Itpk1	1,63019E-28	0,40619	0,423	0,23	2,73725E-24	1	
Faah	1,6696E-28	0,37913	0,384	0,195	2,80342E-24	1	
Spin1	1,97735E-28	0,53594	0,7	0,537	3,32017E-24	1	
Hsp90ab1	2,15547E-28	0,26539	1	1	3,61924E-24	1	
Gpsm3	2,53148E-28	0,36491	0,427	0,215	4,25061E-24	1	
Slc12a7	2,79332E-28	0,43874	0,529	0,334	4,69026E-24	1	
Map4	3,26682E-28	0,37418	0,992	0,976	5,48532E-24	1	
Abca8b	4,74015E-28	0,37299	0,501	0,295	7,95919E-24	1	
Mtpn	4,90883E-28	0,46248	0,863	0,773	8,24242E-24	1	
Klhl21	6,2227E-28	0,38329	0,429	0,232	1,04485E-23	1	
Cpt1a	7,56489E-28	0,44058	0,68	0,491	1,27022E-23	1	
Cdh6	8,03117E-28	0,3843	0,392	0,203	1,34851E-23	1	
Snap47	8,55106E-28	0,43367	0,924	0,882	1,43581E-23	1	
Arhgef11	9,42499E-28	0,42914	0,847	0,739	1,58255E-23	1	
Tnpo1	1,07319E-27	0,46736	0,765	0,622	1,80199E-23	1	
Esyt1	1,12216E-27	0,44883	0,632	0,443	1,88423E-23	1	
Mcu	2,26085E-27	0,30631	0,296	0,13	3,7962E-23	1	
Ptprs	2,56039E-27	0,38073	0,899	0,76	4,29915E-23	1	
Tapt1	3,4684E-27	0,43527	0,457	0,269	5,82379E-23	1	
N4bp2	3,85403E-27	0,40356	0,423	0,23	6,47131E-23	1	
Dusp11	4,99402E-27	0,46127	0,751	0,603	8,38546E-23	1	
Vamp4	5,10908E-27	0,52504	0,775	0,679	8,57865E-23	1	
Klf10	5,55205E-27	0,42316	0,459	0,268	9,32245E-23	1	
Syt7	7,01019E-27	0,3645	0,934	0,829	1,17708E-22	1	
Plxna2	7,04573E-27	0,32906	0,388	0,196	1,18305E-22	1	
Nfkbia	9,17011E-27	0,44148	0,509	0,322	1,53975E-22	1	

Parp1	1,2963E-26	0,41565	0,571	0,387	2,17661E-22	1	
Cbarp	1,55205E-26	0,36734	0,952	0,892	2,60604E-22	1	
Prkaca	2,30891E-26	0,37689	0,905	0,825	3,87689E-22	1	
Fkbp1a	6,33989E-26	0,33423	0,996	0,995	1,06453E-21	1	
Hoxa5	7,58608E-26	0,29407	0,992	0,939	1,27378E-21	1	
Nsfl1c	9,33851E-26	0,44027	0,771	0,639	1,56803E-21	1	
Ier5l	1,19544E-25	0,33716	0,322	0,155	2,00726E-21	1	
Prrc2c	1,50556E-25	0,34268	0,986	0,932	2,52799E-21	1	
Rtn3	1,85713E-25	0,30876	1	0,998	3,11831E-21	1	
Dnmt3a	2,03585E-25	0,3886	0,833	0,707	3,41839E-21	1	
Samd14	2,17067E-25	0,47834	0,837	0,722	3,64478E-21	1	
Cdc42bpb	2,64516E-25	0,48264	0,688	0,569	4,44149E-21	1	
Slc41a3	2,77307E-25	0,31824	0,256	0,109	4,65626E-21	1	
Ddx5	4,5199E-25	0,31506	1	0,999	7,58936E-21	1	
Erf	4,82534E-25	0,39839	0,406	0,228	8,10223E-21	1	
Fam101b	5,86889E-25	0,35433	0,396	0,213	9,85446E-21	1	
Kdsr	7,42311E-25	0,40672	0,563	0,376	1,24641E-20	1	
Manea	1,13815E-24	0,35277	0,328	0,161	1,91107E-20	1	
Irs3	1,17046E-24	0,41536	0,433	0,244	1,96531E-20	1	
Gstm1	1,21785E-24	0,44902	0,604	0,433	2,0449E-20	1	
Clk1	1,58096E-24	0,46526	0,873	0,797	2,65459E-20	1	
Nipal3	2,38892E-24	0,41417	0,789	0,658	4,01124E-20	1	
Sqstm1	3,43737E-24	0,38941	0,952	0,901	5,77169E-20	1	
H1f0	3,52385E-24	0,42773	0,781	0,65	5,9169E-20	1	
Cspg4	3,71305E-24	0,32943	0,284	0,128	6,23459E-20	1	
Adam10	4,5024E-24	0,38255	0,612	0,427	7,55998E-20	1	
PISD	4,91187E-24	0,47804	0,841	0,729	8,24751E-20	1	
Aldh2	7,41669E-24	0,43992	0,487	0,321	1,24534E-19	1	
Ddx3x	7,95114E-24	0,37975	0,849	0,731	1,33508E-19	1	
Ppp4c	9,28345E-24	0,41012	0,499	0,323	1,55878E-19	1	
Mmp24	1,04631E-23	0,30405	0,396	0,215	1,75686E-19	1	
Msl2	1,16271E-23	0,35503	0,433	0,249	1,95231E-19	1	
Hr	1,27555E-23	0,37666	0,592	0,404	2,14178E-19	1	
Plekha6	1,92446E-23	0,3409	0,905	0,779	3,23136E-19	1	
Ppfibp1	1,97671E-23	0,31695	0,362	0,188	3,31909E-19	1	
Osbpl10	1,97902E-23	0,25153	0,252	0,109	3,32296E-19	1	
Srrm4	2,10477E-23	0,39495	0,777	0,679	3,53412E-19	1	
Unc5a	2,92422E-23	0,37329	0,453	0,272	4,91006E-19	1	
Zeb1	4,38927E-23	0,40111	0,779	0,652	7,37002E-19	1	
Myh10	4,87706E-23	0,45429	0,714	0,563	8,18906E-19	1	
Arhgap5	5,49966E-23	0,42007	0,588	0,406	9,23448E-19	1	
Foxn3	7,09681E-23	0,33573	0,427	0,25	1,19162E-18	1	
Larp1	7,93622E-23	0,40336	0,722	0,609	1,33257E-18	1	
Cabp1	7,96113E-23	0,46539	0,634	0,482	1,33675E-18	1	
Jup	1,15413E-22	0,38893	0,767	0,649	1,9379E-18	1	
Ski	1,78788E-22	0,38957	0,751	0,621	3,00202E-18	1	
Alms1	1,9065E-22	0,28868	0,298	0,142	3,2012E-18	1	
Gm20342	3,20209E-22	0,43823	0,517	0,344	5,37664E-18	1	

Id2	3,99185E-22	0,40554	0,938	0,884	6,70271E-18	1	
Rcor3	4,57549E-22	0,39079	0,608	0,44	7,6827E-18	1	
Hpca	5,2174E-22	0,46029	0,557	0,376	8,76053E-18	1	
Spata13	5,989E-22	0,35179	0,443	0,259	1,00561E-17	1	
Fhl1	9,27686E-22	0,41662	0,656	0,521	1,55768E-17	1	
Ddx17	1,23429E-21	0,37374	0,895	0,802	2,07249E-17	1	
Tcf20	1,25217E-21	0,40028	0,602	0,444	2,10251E-17	1	
Magee1	1,62429E-21	0,41655	0,779	0,662	2,72735E-17	1	
Cds2	1,90244E-21	0,31788	0,976	0,915	3,19439E-17	1	
Kmt2a	3,19844E-21	0,34591	0,843	0,712	5,3705E-17	1	
Anxa7	4,34448E-21	0,38604	0,656	0,495	7,29482E-17	1	
Sv2a	4,43776E-21	0,37248	0,865	0,786	7,45144E-17	1	
Zfp652	4,65758E-21	0,35949	0,463	0,291	7,82054E-17	1	
Kcnh1	5,05454E-21	0,34409	0,513	0,335	8,48708E-17	1	
Msl1	5,49756E-21	0,40513	0,734	0,605	9,23096E-17	1	
Dpf1	6,38461E-21	0,2975	0,31	0,158	1,07204E-16	1	
Rnf44	6,97024E-21	0,37746	0,581	0,414	1,17037E-16	1	
Cux1	6,99662E-21	0,39822	0,567	0,422	1,1748E-16	1	
Adam23	1,58615E-20	0,36828	0,509	0,344	2,6633E-16	1	
Gnb1	2,6726E-20	0,26681	0,994	0,989	4,48757E-16	1	
Pitpnm2	2,92622E-20	0,35171	0,795	0,661	4,91342E-16	1	
Hmgcs1	2,94407E-20	0,41189	0,946	0,926	4,94339E-16	1	
H2-K1	3,00517E-20	0,43357	0,817	0,724	5,04598E-16	1	
Lcorl	3,66838E-20	0,32438	0,437	0,265	6,15958E-16	1	
Sgk3	4,20091E-20	0,29603	0,28	0,141	7,05375E-16	1	
Magi1	4,43779E-20	0,37736	0,549	0,389	7,45149E-16	1	
Fam129b	4,79125E-20	0,31914	0,463	0,291	8,04499E-16	1	
Fam168a	5,97915E-20	0,37709	0,656	0,513	1,00396E-15	1	
Nell1	6,19784E-20	0,28362	0,499	0,296	1,04068E-15	1	
Miat	6,67731E-20	0,33017	0,394	0,227	1,12119E-15	1	
Inf2	8,02575E-20	0,25805	0,27	0,131	1,3476E-15	1	
Arhgef1	8,77726E-20	0,38204	0,692	0,568	1,47379E-15	1	
Pth1r	1,25395E-19	0,298	0,41	0,232	2,1055E-15	1	
Pde4a	2,10141E-19	0,30904	0,322	0,172	3,52848E-15	1	
Nt5dc2	2,27284E-19	0,36188	0,457	0,293	3,81632E-15	1	
Erc1	2,70061E-19	0,34949	0,783	0,68	4,53459E-15	1	
Efnb1	3,4978E-19	0,27078	0,288	0,145	5,87316E-15	1	
Map3k5	3,60497E-19	0,27961	0,296	0,152	6,0531E-15	1	
Tbx3	3,62215E-19	0,30109	0,99	0,944	6,08195E-15	1	
Atp8a1	4,11718E-19	0,32547	0,871	0,779	6,91315E-15	1	
Ccdc64	4,66135E-19	0,32867	0,469	0,307	7,82687E-15	1	
Osbpl6	5,11929E-19	0,32253	0,382	0,226	8,5958E-15	1	
Celsr3	5,52789E-19	0,33086	0,483	0,319	9,28188E-15	1	
Adrbk1	5,73365E-19	0,37077	0,777	0,688	9,62736E-15	1	
Adgrl1	5,83788E-19	0,31693	0,934	0,877	9,80239E-15	1	
Tubb4a	5,8533E-19	0,37129	0,869	0,804	9,82828E-15	1	
Dpysl2	6,3359E-19	0,34839	0,984	0,984	1,06386E-14	1	
Myo1b	6,49502E-19	0,30475	0,441	0,276	1,09058E-14	1	

Epb41l3	6,82865E-19	0,36693	0,612	0,471	1,1466E-14	1	
Gigyf1	7,45088E-19	0,3695	0,586	0,435	1,25108E-14	1	
Rab11b	7,93609E-19	0,32612	0,903	0,857	1,33255E-14	1	
Hk1	1,02994E-18	0,30373	0,932	0,875	1,72937E-14	1	
Kmt2d	1,22621E-18	0,35437	0,563	0,403	2,05893E-14	1	
Ywhag	1,24604E-18	0,28488	0,998	0,992	2,09223E-14	1	
Lrrn1	1,90025E-18	0,30423	0,358	0,204	3,19072E-14	1	
Aff4	2,07023E-18	0,28694	0,938	0,894	3,47613E-14	1	
Rbm5	2,07692E-18	0,37711	0,793	0,705	3,48735E-14	1	
Map4k4	2,7453E-18	0,34424	0,767	0,662	4,60963E-14	1	
Ccp110	3,16253E-18	0,30968	0,443	0,277	5,31021E-14	1	
Prune	3,47173E-18	0,33817	0,445	0,288	5,82938E-14	1	
Epb41l4b	4,00852E-18	0,30241	0,39	0,231	6,73071E-14	1	
Smug1	4,5666E-18	0,27825	0,314	0,168	7,66778E-14	1	
Ak1	5,81972E-18	0,36028	0,706	0,603	9,77189E-14	1	
Pds5a	5,93382E-18	0,38702	0,551	0,403	9,96347E-14	1	
Capzb	7,08102E-18	0,34909	0,855	0,79	1,18897E-13	1	
Gnao1	8,11005E-18	0,29149	0,982	0,966	1,36176E-13	1	
Bmf	9,08121E-18	0,257	0,276	0,143	1,52483E-13	1	
Matr3	1,59324E-17	0,30757	0,926	0,879	2,67521E-13	1	
Larp4b	2,0867E-17	0,34867	0,74	0,627	3,50378E-13	1	
Bcor	2,11811E-17	0,3671	0,427	0,283	3,55653E-13	1	
Madd	2,3465E-17	0,347	0,748	0,644	3,94001E-13	1	
Hnrnpc	2,65164E-17	0,32803	0,875	0,808	4,45237E-13	1	
Acvr1b	2,67341E-17	0,30235	0,425	0,266	4,48892E-13	1	
Pfn1	2,7353E-17	0,31083	0,95	0,923	4,59284E-13	1	
Dlx2	2,96965E-17	0,37545	0,453	0,316	4,98634E-13	1	
Clasp2	3,20297E-17	0,32899	0,841	0,755	5,3781E-13	1	
Mafg	3,54276E-17	0,35791	0,761	0,666	5,94865E-13	1	
Vezt	3,98024E-17	0,37659	0,628	0,508	6,68322E-13	1	
Samd1	4,20098E-17	0,32344	0,487	0,332	7,05386E-13	1	
Rgs11	4,28071E-17	0,29598	0,382	0,231	7,18775E-13	1	
Rap1gap	4,39812E-17	0,34682	0,553	0,401	7,38489E-13	1	
Kif5a	5,28632E-17	0,29145	0,988	0,979	8,87626E-13	1	
Krit1	5,57149E-17	0,33128	0,736	0,614	9,3551E-13	1	
Ttc14	5,8357E-17	0,37745	0,75	0,64	9,79872E-13	1	
Gpr149	5,85253E-17	0,2821	0,569	0,375	9,82699E-13	1	
Actg1	7,16902E-17	0,29054	1	1	1,20375E-12	1	
Ptms	7,197E-17	0,2733	0,998	1	1,20845E-12	1	
Htt	7,63168E-17	0,30625	0,614	0,466	1,28144E-12	1	
Fbxl16	8,03581E-17	0,3222	0,763	0,64	1,34929E-12	1	
Mark2	8,63444E-17	0,34024	0,688	0,556	1,44981E-12	1	
Adamts9	1,0176E-16	0,28082	0,467	0,291	1,70865E-12	1	
Appl2	1,06709E-16	0,26717	0,322	0,181	1,79175E-12	1	
Man1a	1,09171E-16	0,32601	0,441	0,286	1,8331E-12	1	
Atf7	1,10284E-16	0,32857	0,586	0,438	1,85178E-12	1	
S100a10	1,37457E-16	0,28641	0,996	0,985	2,30805E-12	1	
Grin1	1,81977E-16	0,39316	0,704	0,609	3,05558E-12	1	

Usf2	2,64431E-16	0,38361	0,688	0,596	4,44006E-12	1	
Tspan17	3,19948E-16	0,39275	0,672	0,58	5,37225E-12	1	
Gm15800	3,813E-16	0,32045	0,829	0,699	6,4024E-12	1	
Nyap1	4,94808E-16	0,31276	0,584	0,44	8,30831E-12	1	
Hnrnph1	5,42219E-16	0,3159	0,795	0,739	9,10439E-12	1	
Shank2	5,86457E-16	0,30094	0,467	0,316	9,84719E-12	1	
Pik3cd	6,02913E-16	0,27242	0,336	0,197	1,01235E-11	1	
Dync1i1	6,12734E-16	0,33991	0,67	0,552	1,02884E-11	1	
Men1	6,98385E-16	0,28754	0,384	0,239	1,17266E-11	1	
Tom1l2	7,45217E-16	0,33255	0,716	0,608	1,25129E-11	1	
Hnrnpa0	8,74085E-16	0,27959	0,903	0,838	1,46768E-11	1	
Atf4	8,82758E-16	0,29738	0,926	0,894	1,48224E-11	1	
Hipk2	9,05611E-16	0,25286	0,382	0,234	1,52061E-11	1	
Numbl	1,02489E-15	0,33558	0,724	0,629	1,72089E-11	1	
Mgll	1,42934E-15	0,35594	0,823	0,764	2,40001E-11	1	
Anxa2	1,61586E-15	0,33894	0,964	0,935	2,71318E-11	1	
Cep170b	1,98789E-15	0,33012	0,706	0,581	3,33786E-11	1	
Nktr	2,22696E-15	0,31274	0,845	0,755	3,73928E-11	1	
Pcsk1n	2,24233E-15	0,29917	1	1	3,76509E-11	1	
Srrm3	2,65985E-15	0,3551	0,642	0,543	4,46616E-11	1	
Nr3c1	2,84047E-15	0,29966	0,425	0,279	4,76943E-11	1	
Kmt2e	3,74449E-15	0,29839	0,847	0,759	6,28738E-11	1	
Scd2	3,88715E-15	0,25963	0,956	0,917	6,52691E-11	1	
Map2k3	4,21737E-15	0,31145	0,396	0,261	7,08139E-11	1	
Celf3	4,33772E-15	0,30944	0,879	0,85	7,28347E-11	1	
Spire2	4,44302E-15	0,29404	0,392	0,253	7,46028E-11	1	
Adrbk2	4,44571E-15	0,27472	0,98	0,943	7,46479E-11	1	
Mpzl1	4,87987E-15	0,26644	0,388	0,244	8,19378E-11	1	
Mbd6	5,04246E-15	0,30857	0,449	0,308	8,4668E-11	1	
Ackr1	5,18822E-15	0,31266	0,445	0,307	8,71154E-11	1	
Boc	6,74522E-15	0,26292	0,318	0,186	1,13259E-10	1	
Cntnap2	8,97626E-15	0,31763	0,483	0,338	1,5072E-10	1	
Plod2	9,15947E-15	0,38845	0,871	0,84	1,53797E-10	1	
Zfp462	9,48928E-15	0,29246	0,481	0,334	1,59335E-10	1	
Kalrn	1,03419E-14	0,27318	0,342	0,21	1,73651E-10	1	
Maea	1,04986E-14	0,36471	0,638	0,533	1,76281E-10	1	
Glul	1,08239E-14	0,33884	0,636	0,526	1,81744E-10	1	
Rsrp1	1,4916E-14	0,25688	0,978	0,957	2,50454E-10	1	
Pabpn1	1,53588E-14	0,35714	0,63	0,516	2,57889E-10	1	
Sult4a1	1,88601E-14	0,2966	0,95	0,942	3,1668E-10	1	
Furin	2,27702E-14	0,33545	0,38	0,246	3,82334E-10	1	
Cntn2	2,29269E-14	0,28561	0,392	0,252	3,84966E-10	1	
Luc7l3	2,79482E-14	0,32474	0,861	0,805	4,69278E-10	1	
Smad1	2,82367E-14	0,32577	0,584	0,465	4,74122E-10	1	
Wnk2	3,47778E-14	0,2705	0,414	0,27	5,83955E-10	1	
Nudt4	4,02478E-14	0,4638	0,525	0,425	6,75802E-10	1	
Dock11	4,77656E-14	0,31197	0,646	0,528	8,02032E-10	1	
Arhgef10l	4,99023E-14	0,27182	0,477	0,335	8,3791E-10	1	

Arl5b	5,06946E-14	0,25285	0,334	0,204	8,51213E-10	1	
Stxbp5l	5,14611E-14	0,33006	0,551	0,427	8,64084E-10	1	
Bmpr2	6,64046E-14	0,29864	0,823	0,744	1,115E-09	1	
Pogz	7,41923E-14	0,25617	0,425	0,285	1,24576E-09	1	
Arhgef25	7,45902E-14	0,33565	0,527	0,401	1,25244E-09	1	
Tet3	9,35765E-14	0,30556	0,521	0,388	1,57124E-09	1	
Ctnna2	1,05424E-13	0,33757	0,626	0,513	1,77018E-09	1	
2700081O15	1,10116E-13	0,30931	0,583	0,455	1,84896E-09	1	
Csnk1e	1,10477E-13	0,29841	0,801	0,734	1,85503E-09	1	
Hnrnpu	1,12681E-13	0,32674	0,692	0,605	1,89202E-09	1	
Ubqln4	1,45244E-13	0,31269	0,451	0,322	2,43879E-09	1	
Copa	1,82923E-13	0,2699	0,831	0,748	3,07145E-09	1	
Clip3	2,01921E-13	0,31047	0,891	0,847	3,39046E-09	1	
Chsy1	2,37788E-13	0,29677	0,451	0,323	3,99271E-09	1	
Zcchc14	2,61111E-13	0,31684	0,479	0,35	4,38431E-09	1	
Magi3	2,70642E-13	0,28258	0,455	0,324	4,54435E-09	1	
Eno2	2,75798E-13	0,31073	0,895	0,834	4,63093E-09	1	
Gatad2b	2,77046E-13	0,28093	0,684	0,552	4,65189E-09	1	
Npr2	2,97972E-13	0,31999	0,583	0,47	5,00324E-09	1	
Gm26917	3,15379E-13	0,4348	0,618	0,523	5,29553E-09	1	
Gtf3c1	3,3092E-13	0,30621	0,628	0,514	5,55647E-09	1	
Tbc1d8	3,329E-13	0,31071	0,481	0,354	5,58972E-09	1	
Tmem151a	3,89159E-13	0,40429	0,696	0,649	6,53437E-09	1	
Ciapin1	4,10394E-13	0,37837	0,738	0,675	6,89093E-09	1	
Ulk1	4,22174E-13	0,2926	0,555	0,428	7,08872E-09	1	
BC037034	4,35214E-13	0,29026	0,425	0,295	7,30767E-09	1	
Diras1	4,38051E-13	0,29193	0,457	0,326	7,35532E-09	1	
Atf7ip	4,42484E-13	0,3112	0,561	0,438	7,42975E-09	1	
Zbtb7a	4,5324E-13	0,28368	0,696	0,601	7,61035E-09	1	
Tspyl1	5,7243E-13	0,35521	0,64	0,536	9,61167E-09	1	
Phf12	6,11962E-13	0,28196	0,443	0,312	1,02754E-08	1	
Tns1	6,52702E-13	0,2554	0,865	0,801	1,09595E-08	1	
Ogt	8,49694E-13	0,32737	0,732	0,649	1,42672E-08	1	
Ankrd10	9,05432E-13	0,27635	0,567	0,441	1,52031E-08	1	
Pfkp	1,19585E-12	0,30198	0,831	0,783	2,00795E-08	1	
Gtf2ird1	1,31594E-12	0,29838	0,606	0,504	2,20959E-08	1	
Hnrnpl	1,3587E-12	0,30405	0,686	0,598	2,2814E-08	1	
Caskin1	1,3907E-12	0,30011	0,521	0,4	2,33513E-08	1	
Arhgap12	1,40836E-12	0,3058	0,535	0,42	2,36477E-08	1	
B3galt1	1,49062E-12	0,29151	0,449	0,328	2,5029E-08	1	
Tulp4	1,55271E-12	0,2583	0,899	0,831	2,60716E-08	1	
Pank3	1,86454E-12	0,27068	0,706	0,61	3,13074E-08	1	
Atxn7	2,08783E-12	0,25275	0,33	0,209	3,50567E-08	1	
Fzr1	2,49475E-12	0,28539	0,453	0,333	4,18893E-08	1	
Usp31	2,50412E-12	0,27841	0,539	0,412	4,20467E-08	1	
Dpysl5	2,57122E-12	0,28855	0,632	0,521	4,31734E-08	1	
1700020I14R	3,02524E-12	0,28667	0,499	0,375	5,07969E-08	1	
Adam19	3,18905E-12	0,29283	0,503	0,378	5,35473E-08	1	

Bbx	3,76891E-12	0,31368	0,497	0,391	6,32837E-08	1	
Litaf	3,94237E-12	0,25063	0,36	0,237	6,61964E-08	1	
Wbp2	4,04293E-12	0,29778	0,775	0,732	6,78848E-08	1	
Rnps1	4,1214E-12	0,30486	0,767	0,702	6,92025E-08	1	
Setd1b	4,26026E-12	0,28753	0,485	0,359	7,15341E-08	1	
Htr3a	4,30446E-12	0,34125	0,738	0,667	7,22761E-08	1	
Ttbk2	4,41402E-12	0,28515	0,757	0,645	7,41157E-08	1	
Srrm1	5,00694E-12	0,29869	0,789	0,73	8,40715E-08	1	
Chfr	5,18484E-12	0,27822	0,447	0,326	8,70587E-08	1	
Tle2	5,6733E-12	0,30752	0,431	0,316	9,52604E-08	1	
Arf1	6,21304E-12	0,26471	0,97	0,953	1,04323E-07	1	
Eif5a2	7,03916E-12	0,27957	0,443	0,317	1,18195E-07	1	
Clic4	7,58431E-12	0,33441	0,318	0,201	1,27348E-07	1	
Sept8	8,66587E-12	0,27652	0,588	0,485	1,45509E-07	1	
Ppp1cb	1,10222E-11	0,29458	0,714	0,624	1,85073E-07	1	
Bean1	1,33384E-11	0,35354	0,577	0,484	2,23965E-07	1	
Cnot6	1,41562E-11	0,31061	0,567	0,466	2,37697E-07	1	
Fry	1,42506E-11	0,31063	0,634	0,542	2,39283E-07	1	
Zcchc7	1,96778E-11	0,29162	0,718	0,654	3,3041E-07	1	
Brd4	2,21325E-11	0,28768	0,67	0,586	3,71627E-07	1	
Ccnt2	2,3519E-11	0,28355	0,573	0,459	3,94907E-07	1	
Brwd1	2,51393E-11	0,32058	0,561	0,457	4,22114E-07	1	
Cpeb2	2,73685E-11	0,25705	0,539	0,418	4,59544E-07	1	
Ube2g1	2,99543E-11	0,26169	0,39	0,274	5,02963E-07	1	
Nat8l	3,2367E-11	0,25255	0,336	0,221	5,43474E-07	1	
Apc2	3,33693E-11	0,27906	0,658	0,552	5,60305E-07	1	
Hn1	3,45356E-11	0,25831	0,972	0,93	5,79887E-07	1	
Ankhd1	3,55739E-11	0,288	0,483	0,377	5,97321E-07	1	
Prpf4b	3,57101E-11	0,26463	0,96	0,936	5,99608E-07	1	
Ablim2	4,33124E-11	0,26547	0,499	0,38	7,27259E-07	1	
Atxn1l	4,37787E-11	0,26805	0,374	0,26	7,35088E-07	1	
Trappc10	4,46526E-11	0,25095	0,614	0,506	7,49762E-07	1	
Rnf38	4,6662E-11	0,25371	0,475	0,351	7,83501E-07	1	
mt-Nd6	5,5957E-11	0,28044	0,429	0,314	9,39573E-07	1	
Arid2	5,9727E-11	0,2775	0,535	0,418	1,00288E-06	1	
Scamp1	6,20296E-11	0,28169	0,785	0,732	1,04154E-06	1	
Camk2d	7,07709E-11	0,26187	0,813	0,77	1,18831E-06	1	
Kmt2c	7,71964E-11	0,26591	0,68	0,601	1,2962E-06	1	
Poldip3	8,23333E-11	0,25927	0,618	0,501	1,38246E-06	1	
Kcnj12	8,63718E-11	0,26022	0,419	0,305	1,45027E-06	1	
Unc13a	8,76496E-11	0,27529	0,632	0,538	1,47172E-06	1	
Dstyk	8,8104E-11	0,2572	0,425	0,309	1,47935E-06	1	
Sppl3	1,02505E-10	0,26398	0,662	0,563	1,72117E-06	1	
Kansl1	1,02583E-10	0,27907	0,588	0,486	1,72247E-06	1	
Arhgap31	1,11196E-10	0,25959	0,525	0,412	1,86709E-06	1	
Srsf2	1,19043E-10	0,28606	0,744	0,666	1,99886E-06	1	
Celf1	1,21592E-10	0,25289	0,584	0,473	2,04166E-06	1	
Mtmr6	1,3195E-10	0,2528	0,791	0,737	2,21558E-06	1	

Zzef1	1,32022E-10	0,26782	0,513	0,414	2,21679E-06	1	
Myo9a	1,79857E-10	0,30521	0,726	0,676	3,01997E-06	1	
Flywch1	2,50907E-10	0,29492	0,835	0,797	4,21297E-06	1	
Parvb	2,75907E-10	0,26223	0,688	0,604	4,63275E-06	1	
Mau2	3,02489E-10	0,25671	0,471	0,365	5,0791E-06	1	
Cbl	4,15081E-10	0,28695	0,61	0,526	6,96962E-06	1	
Arpc4	4,57914E-10	0,26798	0,567	0,474	7,68883E-06	1	
Tomm34	5,30579E-10	0,26262	0,561	0,459	8,90895E-06	1	
Iqgap1	5,63578E-10	0,25472	0,376	0,267	9,46304E-06	1	
Med25	6,8093E-10	0,27379	0,586	0,501	1,14335E-05	1	
Pkia	7,24275E-10	0,27646	0,457	0,348	1,21613E-05	1	
Odf2	9,14462E-10	0,29115	0,469	0,37	1,53547E-05	1	
Mxd4	1,16304E-09	0,25581	0,682	0,623	1,95286E-05	1	
Sorl1	1,3715E-09	0,25176	0,676	0,595	2,30288E-05	1	
Pianp	1,55859E-09	0,27954	0,533	0,447	2,61703E-05	1	
Vps4b	1,84494E-09	0,29085	0,654	0,6	3,09784E-05	1	
Fam178a	1,87369E-09	0,25417	0,513	0,404	3,14611E-05	1	
Leprot	2,16445E-09	0,25997	0,662	0,578	3,63434E-05	1	
Whsc1l1	3,2564E-09	0,27014	0,674	0,607	5,46781E-05	1	
Bmpr1a	3,40704E-09	0,27414	0,473	0,371	5,72076E-05	1	
Fam171a2	3,62124E-09	0,28408	0,644	0,596	6,08043E-05	1	
Wdr82	4,32043E-09	0,27177	0,545	0,456	7,25443E-05	1	
Gopc	4,38429E-09	0,26982	0,475	0,388	7,36166E-05	1	
Daam1	6,30789E-09	0,25872	0,531	0,438	0,000105916	1	
Pds5b	6,34881E-09	0,26398	0,596	0,528	0,000106603	1	
Sbno1	1,37448E-08	0,25672	0,624	0,535	0,000230789	1	
Gt(ROSA)26S	2,17448E-08	0,2974	0,495	0,421	0,000365117	1	
Smc5	2,61009E-08	0,25356	0,455	0,369	0,000438261	1	
Pknox1	2,72939E-08	0,26032	0,463	0,369	0,000458292	1	
Dnmt1	3,50942E-08	0,25233	0,596	0,527	0,000589267	1	
Trim3	6,04013E-08	0,25093	0,449	0,366	0,001014198	1	
Rnpc3	6,31457E-08	0,27148	0,421	0,334	0,00106028	1	
Nsmf	8,32962E-07	0,28236	0,598	0,551	0,013986268	1	
Ubr5	2,36293E-06	0,26068	0,767	0,755	0,039675975	1	
Chic2	2,85615E-06	0,25268	0,571	0,529	0,047957631	1	
B2m	4,71789E-06	0,30198	0,684	0,661	0,079218069	1	
Tekt2	9,1266E-06	0,25785	0,563	0,508	0,15324471	1	
Atp11b	1,8382E-05	0,31104	0,489	0,434	0,308652522	1	
Gm42418	3,11126E-05	0,59665	1	1	0,522411572	1	
Ndrg2	4,12983E-05	0,25237	0,573	0,534	0,693440337	1	
Calcb	0	1,86275	0,997	0,496	0	2	smENC2a
Sst	0	1,84316	1	0,8	0	2	
Ifitm2	0	1,33624	1	0,948	0	2	
Rps27	0	1,28984	1	0,854	0	2	
Csrp2	0	1,2644	0,929	0,363	0	2	
Ifi27	0	1,25377	0,997	0,773	0	2	
Cox6c	0	1,2426	1	0,859	0	2	
Rpl24	0	1,19953	1	0,942	0	2	

Rpl6	0	1,14053	1	0,97	0	2	
Rpl37	0	1,13868	1	0,985	0	2	
Tpt1	0	1,13137	1	0,953	0	2	
Lgals3	0	0,79589	0,416	0,046	0	2	
Rps29	6,0403E-308	1,10027	1	0,997	1,0142E-303	2	
Rps8	6,5776E-305	1,11486	1	0,976	1,1044E-300	2	
Rpl9	1,2161E-302	1,21969	1	0,8	2,042E-298	2	
Rpl35a	5,1176E-302	1,12243	1	0,917	8,593E-298	2	
Rpl37a	5,4216E-302	1,08068	1	0,986	9,1033E-298	2	
Pdlim2	8,8299E-302	1,25619	0,765	0,245	1,4826E-297	2	
Prph	1,4949E-300	0,92055	1	0,969	2,51E-296	2	
Rps21	2,7604E-294	1,24828	1	0,947	4,635E-290	2	
Dmkn	3,5142E-294	1,13088	0,889	0,293	5,9006E-290	2	
Rpl39	2,0751E-293	1,20878	1	0,931	3,4843E-289	2	
Stmn3	1,6099E-290	1,09709	1	0,925	2,7032E-286	2	
Ubb	3,2041E-289	1,10805	1	0,986	5,38E-285	2	
Hint1	1,4389E-287	1,10851	1	0,872	2,416E-283	2	
Rpl13	6,9906E-287	1,02519	1	0,953	1,1738E-282	2	
Rps3a1	3,3248E-286	1,18172	0,999	0,815	5,5826E-282	2	
Rps27a	1,0329E-285	1,07166	1	0,923	1,7344E-281	2	
Rps15a	7,2198E-283	1,04492	1	0,875	1,2123E-278	2	
Atp6v1e1	3,4345E-280	0,98695	1	0,956	5,7668E-276	2	
Ly6h	4,1236E-280	1,13634	0,935	0,45	6,9239E-276	2	
Rpl30	2,7159E-277	1,08626	1	0,796	4,5603E-273	2	
Higd1a	6,3863E-277	0,84961	0,695	0,187	1,0723E-272	2	
Rps3	2,1724E-272	1,07349	0,998	0,854	3,6477E-268	2	
Atp5e	5,3163E-271	1,11244	1	0,829	8,9266E-267	2	
Atp5k	5,887E-269	1,02614	1	0,955	9,8848E-265	2	
Chchd2	2,2553E-268	1,02703	0,999	0,912	3,787E-264	2	
Rpl23	4,6618E-267	0,8139	1	0,991	7,8277E-263	2	
Rpl3	1,0031E-266	1,00717	1	0,915	1,6844E-262	2	
S100a1	1,3994E-266	1,11406	0,996	0,815	2,3498E-262	2	
Fxyd7	4,4913E-266	1,16042	1	0,732	7,5413E-262	2	
Atp5j2	8,1059E-266	1,08955	1	0,774	1,3611E-261	2	
Fth1	1,7357E-265	0,90266	1	0,994	2,9143E-261	2	
Rpl32	2,9517E-265	0,92075	1	0,964	4,9561E-261	2	
Rpl19	4,207E-262	1,0332	1	0,927	7,064E-258	2	
Rpl17	3,789E-261	0,97096	1	0,91	6,3621E-257	2	
Rpl34	5,129E-261	0,97732	1	0,911	8,6122E-257	2	
Rpl36a	5,14E-260	1,05312	0,994	0,74	8,6306E-256	2	
H2afz	2,2774E-259	1,12177	0,978	0,701	3,824E-255	2	
Rps5	4,0372E-258	1,00042	1	0,876	6,7788E-254	2	
Rps24	4,2072E-255	0,96499	1	0,965	7,0642E-251	2	
Slc18a3	2,8478E-254	1,05097	0,972	0,478	4,7818E-250	2	
Ass1	1,1319E-253	1,07615	0,931	0,485	1,9007E-249	2	
Rps20	1,6905E-252	0,98488	1	0,891	2,8385E-248	2	
Rps10	2,743E-252	0,96259	1	0,914	4,6058E-248	2	
Atpif1	6,4442E-252	1,0007	1	0,947	1,082E-247	2	

Aldoa	1,9046E-250	0,75949	1	0,996	3,198E-246	2	
Rpl22l1	4,7874E-250	0,99432	0,994	0,758	8,0386E-246	2	
Uqcr10	5,9367E-249	0,99437	0,993	0,717	9,9684E-245	2	
Rps14	2,1261E-248	0,87604	1	0,95	3,5699E-244	2	
Pfdn5	1,2078E-247	0,98026	0,994	0,73	2,0279E-243	2	
Ndufa3	4,8464E-246	0,98495	0,996	0,751	8,1376E-242	2	
Gap43	6,7449E-245	0,75899	1	0,998	1,1325E-240	2	
Rps4x	1,6479E-243	1,00413	1	0,875	2,767E-239	2	
Map1lc3a	7,8172E-243	0,80583	0,999	0,98	1,3126E-238	2	
Rps13	1,1054E-242	0,97151	0,999	0,803	1,8561E-238	2	
Hmgb1	3,0116E-242	0,83481	1	0,974	5,0568E-238	2	
Rps18	1,71E-241	0,89011	1	0,931	2,8713E-237	2	
Atp5h	4,9943E-240	0,92201	0,999	0,861	8,386E-236	2	
Rps6	1,2171E-239	0,92689	0,997	0,83	2,0436E-235	2	
Rpl21	3,3891E-239	0,8235	1	0,965	5,6906E-235	2	
Rpl38	8,5942E-239	0,91349	1	0,994	1,4431E-234	2	
Rpl18	7,5817E-238	0,94959	0,999	0,855	1,2731E-233	2	
Ndufa4	8,797E-238	0,9803	0,998	0,825	1,4771E-233	2	
Rps9	2,9408E-236	0,92423	1	0,911	4,9379E-232	2	
Rps11	3,3579E-236	0,88026	1	0,931	5,6383E-232	2	
Rpl35	2,1562E-235	1,01306	0,999	0,814	3,6204E-231	2	
Rps7	1,1122E-231	0,99322	0,994	0,767	1,8676E-227	2	
Ddah1	1,0908E-230	0,97314	0,884	0,374	1,8316E-226	2	
Cox4i1	2,8837E-230	0,96405	1	0,877	4,842E-226	2	
Rpl7	5,4392E-230	0,95395	0,991	0,744	9,133E-226	2	
Gm10076	9,6232E-228	1,05333	0,999	0,832	1,6158E-223	2	
Ndufb10	2,5952E-227	0,92359	0,948	0,47	4,3575E-223	2	
Vwc2	3,1796E-227	0,7965	0,683	0,21	5,3388E-223	2	
Ndufb9	4,7044E-227	0,97839	0,975	0,605	7,8991E-223	2	
Rab3b	6,1692E-226	0,88212	0,92	0,413	1,0359E-221	2	
Ndufa5	2,1288E-225	0,9072	0,998	0,789	3,5744E-221	2	
Eef1b2	1,7111E-224	0,88595	0,994	0,799	2,8731E-220	2	
Rpl10a	1,9304E-224	0,89227	0,999	0,85	3,2414E-220	2	
Cox5a	2,4039E-224	0,9497	0,979	0,598	4,0364E-220	2	
Sec62	9,1191E-223	0,82236	1	0,935	1,5312E-218	2	
Rps28	2,2987E-221	0,96753	0,999	0,808	3,8597E-217	2	
Tomm7	3,6881E-221	0,91262	0,987	0,752	6,1927E-217	2	
Ftl1	1,287E-220	0,87121	1	0,923	2,1609E-216	2	
Rhoc	2,9147E-220	0,88422	0,841	0,359	4,8942E-216	2	
Chgb	2,9473E-219	1,04083	0,973	0,734	4,9489E-215	2	
Ndufc1	8,0617E-219	0,88675	0,998	0,8	1,3536E-214	2	
Kif22	2,0942E-217	1,07217	0,975	0,719	3,5163E-213	2	
Psme1	1,7844E-215	0,90399	0,996	0,836	2,9962E-211	2	
Rpl27a	1,967E-215	0,79755	1	0,961	3,3028E-211	2	
S100a6	2,5441E-214	0,84772	1	0,999	4,2718E-210	2	
Tspo	4,5385E-214	0,92904	0,958	0,624	7,6206E-210	2	
Uqcr11	2,5177E-213	0,91046	0,998	0,813	4,2275E-209	2	
Rpl26	5,9463E-212	0,80965	1	0,948	9,9844E-208	2	

Fau	1,5429E-211	0,87734	1	0,917	2,5906E-207	2	
Slc25a4	3,8198E-211	0,76569	1	0,979	6,4139E-207	2	
Nme1	1,9523E-210	0,90576	0,963	0,637	3,2782E-206	2	
Ndufa7	2,2634E-210	0,90189	0,987	0,671	3,8005E-206	2	
Rpl11	6,2988E-210	0,79589	1	0,95	1,0576E-205	2	
Tmsb10	5,0658E-208	0,85719	0,999	0,949	8,506E-204	2	
Oaz1	8,1533E-208	0,80843	0,999	0,922	1,369E-203	2	
Gapdh	8,6322E-207	0,92715	1	0,926	1,4494E-202	2	
Eef1a1	2,2335E-206	0,65614	1	0,999	3,7503E-202	2	
2010107E04	3,1822E-205	0,88123	0,973	0,625	5,3433E-201	2	
Ndufs5	1,7204E-202	0,8098	0,997	0,843	2,8887E-198	2	
Rps26	3,0711E-202	0,79696	1	0,911	5,1567E-198	2	
Naca	6,6365E-201	0,7934	0,994	0,864	1,1143E-196	2	
Rps23	6,6365E-201	0,85581	1	0,895	1,1143E-196	2	
Atp5f1	2,072E-200	0,8607	0,972	0,64	3,4791E-196	2	
Adgre1	4,511E-200	0,92256	0,854	0,43	7,5743E-196	2	
Crip1	1,001E-198	0,94167	1	0,986	1,6808E-194	2	
C1ql3	1,7373E-194	0,47594	0,324	0,047	2,9172E-190	2	
Tmco1	1,2677E-191	0,85881	0,93	0,561	2,1286E-187	2	
Ndufa1	3,5643E-190	0,83706	0,96	0,545	5,9848E-186	2	
Avpr1a	4,7169E-190	0,6427	0,687	0,216	7,9202E-186	2	
Gng3	6,0081E-190	0,81256	0,988	0,797	1,0088E-185	2	
Gnb2l1	1,526E-189	0,82215	0,99	0,787	2,5622E-185	2	
Itm2b	3,192E-189	0,58994	1	1	5,3598E-185	2	
Krt19	1,1096E-188	0,96774	0,796	0,308	1,863E-184	2	
Rprm	1,1162E-186	0,69317	0,708	0,242	1,8742E-182	2	
Psmb5	1,0086E-185	0,83594	0,986	0,76	1,6936E-181	2	
Rps16	4,8986E-185	0,75916	0,999	0,917	8,2252E-181	2	
Rpl8	9,6149E-185	0,68183	1	0,969	1,6144E-180	2	
Cox6a1	1,7049E-184	0,77979	0,998	0,834	2,8626E-180	2	
Cox8a	7,8229E-184	0,75713	1	0,925	1,3135E-179	2	
Bend5	9,9755E-183	0,81129	0,813	0,385	1,675E-178	2	
Cox6b1	1,7727E-182	0,78146	0,999	0,861	2,9766E-178	2	
Rpl10	5,232E-182	0,77806	1	0,867	8,785E-178	2	
Tuba1a	1,5528E-181	0,82747	1	0,992	2,6074E-177	2	
Cryab	4,3094E-181	0,75964	0,838	0,366	7,236E-177	2	
Cox7a2	6,4088E-181	0,77549	0,994	0,838	1,0761E-176	2	
Prdx2	8,0822E-181	0,79817	0,99	0,813	1,3571E-176	2	
Rplp2	1,1756E-180	0,73223	1	0,926	1,974E-176	2	
Sec61g	1,3956E-180	0,79395	0,999	0,859	2,3433E-176	2	
Rplp0	2,1546E-180	0,80969	0,983	0,757	3,6177E-176	2	
Rtn1	1,5744E-177	0,58008	1	0,999	2,6436E-173	2	
Ndufb4	2,5491E-176	0,77954	0,984	0,757	4,2802E-172	2	
6330403K07	1,1163E-175	0,78522	0,992	0,933	1,8744E-171	2	
Mif	1,9301E-175	0,79425	0,979	0,729	3,2408E-171	2	
Atp1b1	1,0279E-174	0,79976	0,996	0,858	1,7259E-170	2	
Mpc2	1,3707E-172	0,75065	0,885	0,449	2,3016E-168	2	
Ppia	8,6839E-172	0,51342	1	1	1,4581E-167	2	

Sep15	1,0228E-171	0,75309	0,984	0,811	1,7174E-167	2	
Ubl5	7,103E-170	0,75364	0,983	0,753	1,1927E-165	2	
Eif2s2	4,2357E-168	0,70606	0,994	0,906	7,1121E-164	2	
Gabarapl2	9,0442E-168	0,70408	0,997	0,86	1,5186E-163	2	
Rps2	1,6997E-167	0,77251	0,988	0,832	2,8539E-163	2	
Uqcrb	2,0094E-166	0,72833	0,983	0,781	3,374E-162	2	
Gabarap	2,9079E-166	0,66447	0,999	0,914	4,8826E-162	2	
Rplp1	3,5905E-166	0,63995	1	0,977	6,0287E-162	2	
Ctsz	4,3388E-166	0,65966	0,632	0,229	7,2853E-162	2	
Rpl7a	2,5887E-165	0,73072	0,989	0,811	4,3467E-161	2	
Atp5l	3,6636E-165	0,7032	0,999	0,899	6,1516E-161	2	
Cox5b	5,7274E-165	0,75179	0,992	0,787	9,6169E-161	2	
Rps17	9,6644E-165	0,69586	0,997	0,905	1,6227E-160	2	
Rpl41	1,0443E-164	0,61345	1	0,998	1,7535E-160	2	
Rps19	1,2495E-164	0,70278	0,999	0,943	2,0981E-160	2	
Bsg	1,6412E-163	0,68442	0,994	0,934	2,7558E-159	2	
Atp5j	1,9862E-163	0,72612	0,988	0,817	3,3349E-159	2	
Uqcrq	2,1169E-163	0,71733	0,996	0,849	3,5545E-159	2	
Ndufb8	2,3319E-163	0,75938	0,928	0,548	3,9155E-159	2	
Rps12	8,4869E-163	0,76834	0,994	0,812	1,425E-158	2	
Gpsm3	6,8852E-162	0,69144	0,578	0,186	1,1561E-157	2	
Fdps	1,0353E-161	0,79044	0,973	0,739	1,7384E-157	2	
Btf3	1,2646E-160	0,74281	0,975	0,732	2,1234E-156	2	
Myeov2	7,0639E-160	0,7504	0,885	0,467	1,1861E-155	2	
Ckmt1	1,1227E-159	0,77804	0,853	0,486	1,8852E-155	2	
Rpl12	2,589E-158	0,71084	0,986	0,791	4,3471E-154	2	
Rpl29	3,6044E-158	0,78753	0,931	0,584	6,0521E-154	2	
Rps15	4,147E-157	0,65858	0,999	0,941	6,9633E-153	2	
Eif3e	6,0119E-157	0,76677	0,877	0,533	1,0095E-152	2	
Rpl28	6,0125E-157	0,6334	0,999	0,962	1,0096E-152	2	
Atp5c1	1,8859E-156	0,75422	0,929	0,574	3,1666E-152	2	
Ramp1	2,4999E-156	0,82105	0,882	0,524	4,1976E-152	2	
Ndufa13	4,3402E-156	0,71721	0,988	0,788	7,2876E-152	2	
Nmt1	5,0734E-156	0,66598	0,996	0,913	8,5188E-152	2	
Ndufb5	2,9039E-154	0,70749	0,863	0,456	4,876E-150	2	
Tubb4b	4,4426E-154	0,7581	0,977	0,768	7,4595E-150	2	
Cisd1	8,7602E-153	0,69786	0,949	0,69	1,4709E-148	2	
Emb	2,6698E-152	0,59727	0,585	0,197	4,4828E-148	2	
Stmn2	3,7763E-152	0,47002	1	1	6,3408E-148	2	
Ndufb2	8,0751E-152	0,73602	0,951	0,637	1,3559E-147	2	
Ndufs6	9,0645E-152	0,72427	0,937	0,628	1,522E-147	2	
Ywhae	1,4141E-150	0,61178	1	0,996	2,3745E-146	2	
Uqcrh	1,9907E-150	0,70628	0,979	0,75	3,3425E-146	2	
Usmg5	7,9801E-150	0,67303	0,997	0,841	1,3399E-145	2	
Aimp1	4,4155E-149	0,72724	0,948	0,647	7,4141E-145	2	
Calb2	2,0096E-148	0,60298	1	0,977	3,3743E-144	2	
Smdt1	1,0823E-147	0,65052	0,996	0,887	1,8172E-143	2	
Rpl36al	3,8804E-147	0,66724	0,992	0,825	6,5157E-143	2	

Atp5g2	1,0952E-146	0,66742	0,994	0,832	1,839E-142	2	
Spcs1	4,6456E-146	0,75028	0,871	0,494	7,8004E-142	2	
Gfra2	1,5714E-145	0,64525	0,929	0,407	2,6385E-141	2	
Ndufa2	2,7241E-145	0,65898	0,996	0,876	4,574E-141	2	
Prima1	4,12E-145	0,52071	0,473	0,136	6,9179E-141	2	
Txn1	8,2742E-145	0,62767	0,993	0,907	1,3893E-140	2	
Atp5b	7,5879E-144	0,64578	0,999	0,914	1,2741E-139	2	
1110004F10	1,7926E-143	0,65136	0,998	0,926	3,01E-139	2	
Nenf	1,8943E-143	0,68626	0,98	0,756	3,1807E-139	2	
Coq7	2,0075E-143	0,64265	0,733	0,341	3,3708E-139	2	
Fkbp3	9,3553E-142	0,69747	0,936	0,629	1,5708E-137	2	
Pcsk2	3,1477E-140	0,55388	1	1	5,2853E-136	2	
Dctn3	3,8961E-140	0,67348	0,95	0,704	6,5419E-136	2	
Ifitm3	4,8842E-140	0,9379	0,641	0,275	8,2011E-136	2	
S100a10	5,9311E-140	0,55843	1	0,984	9,9589E-136	2	
Dync1i2	9,356E-139	0,50657	1	0,999	1,571E-134	2	
Eif3h	6,8778E-138	0,70627	0,869	0,503	1,1548E-133	2	
Aprt	9,3822E-138	0,70431	0,88	0,519	1,5754E-133	2	
Dstn	2,6505E-137	0,63665	1	0,946	4,4505E-133	2	
Slc35g2	4,5597E-137	0,47019	0,43	0,117	7,6562E-133	2	
Cox17	8,6317E-137	0,68845	0,866	0,488	1,4493E-132	2	
Rpl36	1,7847E-136	0,62041	1	0,942	2,9967E-132	2	
Mrpl20	8,8964E-136	0,65086	0,828	0,421	1,4938E-131	2	
Rps25	2,5094E-135	0,64435	0,994	0,844	4,2136E-131	2	
Hras	8,9259E-135	0,65314	0,945	0,713	1,4988E-130	2	
Rasd2	2,3094E-134	0,53687	0,618	0,229	3,8777E-130	2	
Ndufa6	1,0147E-132	0,68413	0,929	0,609	1,7037E-128	2	
Mrpl52	1,2811E-132	0,65596	0,967	0,753	2,151E-128	2	
Gde1	3,3588E-132	0,68304	0,879	0,541	5,6397E-128	2	
Rtp4	6,3881E-132	0,55037	0,513	0,172	1,0726E-127	2	
Cmbl	8,899E-132	0,66344	0,747	0,377	1,4942E-127	2	
Cox7b	1,0854E-131	0,6336	0,992	0,829	1,8225E-127	2	
Vsnl1	1,7507E-131	0,49878	0,427	0,123	2,9395E-127	2	
Pdcd5	1,6846E-130	0,62883	0,971	0,767	2,8287E-126	2	
Ndufab1	6,9161E-130	0,65318	0,942	0,68	1,1613E-125	2	
Tmem158	1,0133E-129	0,73906	0,922	0,698	1,7014E-125	2	
Ndufb7	1,5496E-128	0,63457	0,967	0,76	2,6019E-124	2	
Pla2g7	3,0477E-128	0,52875	0,518	0,171	5,1174E-124	2	
Arhgdig	6,2624E-128	0,66319	0,915	0,644	1,0515E-123	2	
Atp6v0d1	1,2082E-127	0,63892	0,967	0,772	2,0287E-123	2	
Cox7c	1,5131E-127	0,60422	1	0,954	2,5406E-123	2	
Fabp5	7,9205E-127	0,55812	1	0,979	1,3299E-122	2	
Park7	1,3445E-126	0,61667	0,972	0,797	2,2576E-122	2	
Tmem258	2,0841E-126	0,6319	0,841	0,457	3,4994E-122	2	
Serping1	3,6073E-126	0,5973	0,796	0,369	6,057E-122	2	
Cycs	3,6232E-126	0,64341	0,838	0,481	6,0837E-122	2	
Ndufb6	1,1625E-125	0,66288	0,861	0,506	1,9519E-121	2	
Psme2	1,9187E-125	0,63844	0,899	0,526	3,2217E-121	2	

1810022K09	2,7917E-125	0,64005	0,897	0,558	4,6876E-121	2	
Rab3a	5,2625E-125	0,58806	0,993	0,888	8,8362E-121	2	
Ndufb3	1,2417E-124	0,63215	0,924	0,626	2,0849E-120	2	
Ly6e	2,7541E-124	0,59301	0,898	0,462	4,6244E-120	2	
Skp1a	6,4912E-124	0,58546	0,992	0,904	1,0899E-119	2	
Tecr	8,9558E-124	0,5369	1	0,972	1,5038E-119	2	
Acadl	9,6151E-124	0,6523	0,945	0,745	1,6145E-119	2	
Rpl18a	1,2273E-123	0,53797	1	0,975	2,0607E-119	2	
Wscd1	2,9948E-123	0,49367	0,41	0,116	5,0286E-119	2	
Uba52	4,4782E-123	0,66344	0,823	0,43	7,5193E-119	2	
Slirp	6,2665E-123	0,58495	0,79	0,403	1,0522E-118	2	
Coa3	1,5503E-122	0,62261	0,859	0,498	2,6031E-118	2	
Sumo1	2,4468E-122	0,62732	0,908	0,605	4,1084E-118	2	
Pmm1	3,4074E-122	0,65576	0,878	0,584	5,7213E-118	2	
Atox1	3,6643E-122	0,6587	0,91	0,636	6,1528E-118	2	
Bri3	2,9776E-121	0,62227	0,825	0,48	4,9997E-117	2	
Nsa2	2,0193E-120	0,65456	0,943	0,718	3,3906E-116	2	
Psma7	5,2623E-120	0,57684	0,996	0,881	8,8359E-116	2	
Fxr1	8,9739E-120	0,62785	0,947	0,731	1,5068E-115	2	
Pomp	1,5079E-119	0,61959	0,937	0,753	2,532E-115	2	
Atp6v0b	8,5976E-119	0,53644	0,994	0,951	1,4436E-114	2	
Tmem256	8,6338E-119	0,62219	0,863	0,479	1,4497E-114	2	
Fez1	1,5151E-118	0,6477	0,955	0,789	2,544E-114	2	
Sars	3,7723E-117	0,64947	0,921	0,703	6,3341E-113	2	
Rbm3	3,2359E-116	0,60658	0,967	0,775	5,4334E-112	2	
Usp50	1,4116E-115	0,55354	0,734	0,354	2,3702E-111	2	
Use1	2,0226E-115	0,59015	0,776	0,412	3,3961E-111	2	
Tspan3	2,8272E-115	0,55405	0,99	0,895	4,7472E-111	2	
Cuedc2	3,2717E-115	0,60042	0,865	0,499	5,4935E-111	2	
Cpe	4,7089E-115	0,57214	0,997	0,933	7,9068E-111	2	
Dek	5,1037E-115	0,65851	0,874	0,602	8,5696E-111	2	
Ndufb11	1,4248E-114	0,61129	0,915	0,58	2,3923E-110	2	
Edf1	7,4674E-114	0,59059	0,947	0,682	1,2539E-109	2	
Rabac1	1,112E-113	0,5692	0,998	0,892	1,8671E-109	2	
Emc2	2,7163E-113	0,60103	0,756	0,411	4,5609E-109	2	
Eid1	3,016E-113	0,54319	0,988	0,904	5,0642E-109	2	
Mrpl28	4,3238E-113	0,52231	0,631	0,275	7,26E-109	2	
Tmem100	6,0974E-113	0,67632	0,891	0,603	1,0238E-108	2	
Psmb1	2,2939E-112	0,56675	0,981	0,815	3,8517E-108	2	
Nap1l1	2,4075E-112	0,63957	0,917	0,715	4,0424E-108	2	
Tac1	3,4973E-112	0,54275	0,42	0,133	5,8723E-108	2	
Romo1	4,1002E-112	0,56587	0,993	0,857	6,8847E-108	2	
Tonsl	1,2946E-110	0,58953	0,895	0,583	2,1738E-106	2	
Ngfrap1	4,2652E-110	0,51413	0,996	0,944	7,1616E-106	2	
Pcdh7	4,7215E-110	0,49785	0,786	0,349	7,9279E-106	2	
Lrrtm1	5,1654E-110	0,52452	0,625	0,272	8,6732E-106	2	
Dpm3	6,5879E-110	0,59531	0,901	0,582	1,1062E-105	2	
Pebp1	6,7838E-110	0,5022	0,998	0,958	1,1391E-105	2	

Shfm1	8,651E-110	0,52248	0,99	0,922	1,4526E-105	2	
Atp6v1f	1,997E-109	0,55846	0,968	0,798	3,3532E-105	2	
Sncb	7,5431E-109	0,48661	0,542	0,211	1,2666E-104	2	
Psmb6	8,6602E-109	0,61464	0,965	0,754	1,4541E-104	2	
Mt3	1,0265E-108	0,71111	0,918	0,714	1,7236E-104	2	
Ap2a2	3,8469E-108	0,50622	0,999	0,978	6,4594E-104	2	
Fam162a	1,9559E-107	0,58183	0,722	0,377	3,2842E-103	2	
Pmvk	2,3441E-107	0,62732	0,837	0,511	3,9359E-103	2	
Pam16	4,1484E-107	0,54747	0,745	0,389	6,9656E-103	2	
2410015M2(	4,3427E-107	0,58649	0,859	0,523	7,2918E-103	2	
Cetn2	3,0933E-106	0,60295	0,929	0,677	5,194E-102	2	
Gm1673	4,4559E-106	0,56147	0,961	0,758	7,4819E-102	2	
Tmem50a	4,9351E-104	0,52928	0,983	0,904	8,2865E-100	2	
Mrpl23	6,3782E-104	0,51013	0,757	0,39	1,071E-99	2	
Pxmp2	8,717E-104	0,56343	0,655	0,328	1,4637E-99	2	
Tceb2	1,6312E-103	0,50362	0,999	0,954	2,739E-99	2	
Selk	1,993E-103	0,4747	0,998	0,97	3,3464E-99	2	
Lamtor4	2,0561E-103	0,56196	0,729	0,389	3,4524E-99	2	
Casp1	2,4013E-103	0,33307	0,283	0,066	4,032E-99	2	
Prkcdbp	3,0059E-103	0,57866	0,639	0,308	5,0471E-99	2	
Cnih3	4,3186E-103	0,39027	0,435	0,142	7,2514E-99	2	
Pfdn1	2,0888E-102	0,57175	0,906	0,632	3,50726E-98	2	
Uqcc2	2,2878E-102	0,57457	0,942	0,699	3,84148E-98	2	
Cops5	3,7825E-102	0,60256	0,912	0,722	6,35119E-98	2	
Anxa2	7,6412E-102	0,52967	0,987	0,93	1,28304E-97	2	
Ndufv2	8,6524E-102	0,58859	0,873	0,571	1,45282E-97	2	
Bcap31	4,5778E-101	0,56884	0,793	0,475	7,68664E-97	2	
Ddrgk1	5,1947E-101	0,55694	0,737	0,398	8,72249E-97	2	
Tceal5	8,5892E-101	0,58062	0,779	0,447	1,44222E-96	2	
Atp5o	3,9856E-100	0,55389	0,972	0,791	6,69229E-96	2	
Selm	2,3536E-99	0,54788	0,981	0,85	3,952E-95	2	
Nars	2,3562E-99	0,58168	0,939	0,794	3,95623E-95	2	
Vipr2	4,1618E-99	0,48813	0,611	0,256	6,98806E-95	2	
Ddah2	5,7789E-99	0,59866	0,771	0,461	9,7033E-95	2	
Stmn1	1,71746E-98	0,5405	0,983	0,895	2,88379E-94	2	
Cacybp	1,89214E-98	0,58286	0,84	0,535	3,1771E-94	2	
H2-D1	2,72572E-98	0,65148	0,911	0,731	4,57675E-94	2	
Dbi	3,2303E-98	0,55312	0,777	0,451	5,424E-94	2	
Psmb10	5,89478E-98	0,61057	0,896	0,659	9,89793E-94	2	
Nell1	2,78892E-97	0,42343	0,643	0,267	4,68287E-93	2	
Dctn2	3,03943E-97	0,55305	0,947	0,702	5,1035E-93	2	
Scg2	3,32296E-97	0,36978	1	1	5,57958E-93	2	
D8Ertd738e	4,15045E-97	0,54598	0,858	0,532	6,96903E-93	2	
Rdx	1,07407E-96	0,53371	0,977	0,891	1,80347E-92	2	
Ube2m	1,49801E-96	0,55174	0,917	0,688	2,5153E-92	2	
Dnajb6	1,7564E-96	0,54243	0,991	0,933	2,94917E-92	2	
Vdac2	2,13538E-96	0,546	0,902	0,666	3,58551E-92	2	
Sparc	5,93001E-96	0,56969	0,89	0,593	9,95707E-92	2	

Ccl27a	6,05121E-96	0,56125	0,715	0,395	1,01606E-91	2	
Cd9	7,32318E-96	0,41793	1	0,993	1,22963E-91	2	
Ndufs4	1,08374E-95	0,53485	0,847	0,529	1,8197E-91	2	
Rpl31	1,36423E-95	0,51112	0,986	0,871	2,29069E-91	2	
Sarnp	1,4001E-95	0,50519	0,772	0,426	2,35091E-91	2	
Sap30	2,87307E-95	0,42928	0,425	0,151	4,82417E-91	2	
Smap1	9,38782E-95	0,54034	0,874	0,599	1,57631E-90	2	
Nt5dc2	2,49665E-94	0,48662	0,592	0,268	4,19213E-90	2	
Atp5g1	4,92039E-94	0,51591	0,994	0,923	8,26183E-90	2	
Eif3k	5,90524E-94	0,53793	0,927	0,686	9,91548E-90	2	
Aqp1	8,23346E-94	0,42066	0,5	0,191	1,38248E-89	2	
Dnaja1	1,79394E-93	0,44482	0,999	0,993	3,01221E-89	2	
Mxra7	1,87695E-93	0,55485	0,684	0,369	3,15158E-89	2	
Spr	2,55209E-93	0,47933	0,66	0,33	4,28521E-89	2	
Cox7a2l	2,69189E-93	0,53396	0,899	0,597	4,51996E-89	2	
Supt4a	3,43723E-93	0,49108	0,712	0,375	5,77145E-89	2	
Ndufv3	3,72018E-93	0,51432	0,984	0,85	6,24655E-89	2	
Fundc2	5,30408E-93	0,52934	0,758	0,436	8,90608E-89	2	
Atp6v1g1	1,30878E-92	0,48489	0,986	0,918	2,19757E-88	2	
Nol7	2,14428E-92	0,5395	0,833	0,522	3,60047E-88	2	
Rpl13a	2,40979E-92	0,43364	1	0,988	4,04627E-88	2	
Pin4	3,83508E-92	0,50209	0,773	0,428	6,43949E-88	2	
Denr	4,75195E-92	0,5335	0,799	0,483	7,979E-88	2	
Mt1	1,01934E-91	0,5469	0,418	0,152	1,71157E-87	2	
Psmd6	1,72009E-91	0,54432	0,812	0,504	2,8882E-87	2	
Ndufaf2	3,28195E-91	0,50696	0,684	0,357	5,51073E-87	2	
Tbcb	4,21014E-91	0,50893	0,932	0,691	7,06925E-87	2	
Dnajc15	6,81784E-91	0,48014	0,568	0,264	1,14478E-86	2	
Polr2i	7,21696E-91	0,51717	0,671	0,347	1,2118E-86	2	
Lamtor2	7,45204E-91	0,5296	0,817	0,493	1,25127E-86	2	
Tmem14a	9,12362E-91	0,47029	0,553	0,253	1,53195E-86	2	
Calm2	1,21016E-90	0,36084	1	1	2,03198E-86	2	
Paip2	3,31211E-90	0,53021	0,969	0,863	5,56137E-86	2	
Akr1a1	3,37475E-90	0,49712	0,972	0,9	5,66655E-86	2	
Hcfc1r1	3,22399E-89	0,48737	0,98	0,893	5,4134E-85	2	
Srp14	9,83084E-89	0,51033	0,953	0,806	1,6507E-84	2	
Mdh2	1,9266E-88	0,53808	0,93	0,703	3,23495E-84	2	
Mrps24	3,19377E-88	0,51854	0,68	0,368	5,36266E-84	2	
Pdlim7	3,41754E-88	0,54025	0,889	0,633	5,73838E-84	2	
Lamtor5	7,82534E-88	0,49491	0,718	0,4	1,31395E-83	2	
H1fx	1,27366E-87	0,55337	0,904	0,675	2,1386E-83	2	
Tbrg1	1,60844E-87	0,53125	0,777	0,463	2,70072E-83	2	
Ufc1	1,88295E-87	0,50116	0,735	0,419	3,16166E-83	2	
Caly	2,09972E-87	0,53048	0,925	0,743	3,52564E-83	2	
Psmb3	3,28138E-87	0,49502	0,961	0,81	5,50976E-83	2	
Cfl2	3,3787E-87	0,54066	0,891	0,688	5,67318E-83	2	
Map1lc3b	3,52432E-87	0,51511	0,948	0,758	5,91769E-83	2	
Plcxd3	4,21941E-87	0,53261	0,684	0,376	7,08482E-83	2	

Rpl27	4,22492E-87	0,53754	0,771	0,46	7,09406E-83	2	
Acp1	7,57406E-87	0,5449	0,769	0,473	1,27176E-82	2	
Hagh	7,59352E-87	0,50463	0,753	0,442	1,27503E-82	2	
Znhit1	7,71391E-87	0,5472	0,93	0,703	1,29524E-82	2	
Pcdh17	1,64932E-86	0,59956	0,951	0,833	2,76937E-82	2	
Ppib	3,33116E-86	0,51503	0,867	0,575	5,59335E-82	2	
Arl3	5,5861E-86	0,50228	0,78	0,454	9,37962E-82	2	
Dpcd	7,00159E-86	0,46422	0,634	0,32	1,17564E-81	2	
Txnl4a	7,35916E-86	0,4458	0,615	0,301	1,23568E-81	2	
Pgam1	8,29784E-86	0,52172	0,943	0,796	1,39329E-81	2	
Gm2990	8,74221E-86	0,35618	0,396	0,138	1,4679E-81	2	
Cxx1a	1,44576E-85	0,4896	0,945	0,786	2,42758E-81	2	
Ndufc2	2,12224E-85	0,48527	0,965	0,798	3,56346E-81	2	
Minos1	2,373E-85	0,51234	0,912	0,677	3,9845E-81	2	
Ptma	4,9201E-85	0,32094	1	1	8,26133E-81	2	
Glrx3	9,64245E-85	0,50292	0,799	0,479	1,61906E-80	2	
Gm10073	1,66702E-84	0,51923	0,585	0,287	2,79909E-80	2	
Cnpy2	2,44879E-84	0,46757	0,647	0,342	4,11176E-80	2	
C1qbp	5,2606E-84	0,54307	0,768	0,472	8,83308E-80	2	
Gm15417	5,59727E-84	0,33578	0,363	0,121	9,39838E-80	2	
Bola2	7,99648E-84	0,50286	0,895	0,614	1,34269E-79	2	
Timm13	9,48311E-84	0,51638	0,85	0,57	1,59231E-79	2	
Sphkap	2,33051E-83	0,3812	0,59	0,249	3,91316E-79	2	
Idh2	4,14703E-83	0,46391	0,574	0,282	6,96328E-79	2	
Nrcam	7,54626E-83	0,43358	0,529	0,237	1,26709E-78	2	
Sumo2	7,64156E-83	0,43629	0,988	0,94	1,28309E-78	2	
Fam19a5	1,80213E-82	0,40041	0,751	0,365	3,02596E-78	2	
Cd59a	1,97144E-82	0,50533	0,661	0,352	3,31025E-78	2	
Ube2e2	2,15815E-82	0,48721	0,713	0,411	3,62376E-78	2	
Ankra2	1,04135E-81	0,53064	0,888	0,681	1,74853E-77	2	
Rpl22	1,47428E-81	0,5023	0,91	0,649	2,47547E-77	2	
Erh	1,84702E-81	0,52272	0,839	0,574	3,10133E-77	2	
Snrpd2	3,49332E-81	0,50469	0,928	0,721	5,86563E-77	2	
Fabp3	7,67201E-81	0,35206	0,356	0,122	1,28821E-76	2	
Timm8b	9,59636E-81	0,49597	0,898	0,62	1,61133E-76	2	
Ybx1	1,14954E-80	0,4778	0,932	0,736	1,93019E-76	2	
2210013O21	1,24797E-80	0,52914	0,824	0,567	2,09547E-76	2	
Trappc4	1,70392E-80	0,47722	0,793	0,479	2,86105E-76	2	
Aurkaip1	2,2237E-80	0,48752	0,708	0,399	3,73382E-76	2	
Cyc1	3,38673E-80	0,47191	0,749	0,43	5,68665E-76	2	
Tm4sf1	3,7527E-80	0,74508	0,899	0,74	6,30115E-76	2	
Psma1	5,31329E-80	0,48778	0,823	0,523	8,92154E-76	2	
Tmsb4x	5,46706E-80	0,34202	1	1	9,17973E-76	2	
Sirt3	1,01686E-79	0,46775	0,604	0,314	1,7074E-75	2	
Psmd8	1,23679E-79	0,49863	0,87	0,594	2,07669E-75	2	
Eif6	1,70636E-79	0,4415	0,641	0,334	2,86516E-75	2	
Vdac3	1,99025E-79	0,49831	0,837	0,57	3,34184E-75	2	
Txndc15	4,1569E-79	0,43241	0,625	0,323	6,97985E-75	2	

Tspan8	4,65355E-79	0,63787	0,328	0,111	7,81377E-75	2	
Pet100	1,05385E-78	0,49515	0,886	0,619	1,76953E-74	2	
Fuom	1,53709E-78	0,40511	0,475	0,204	2,58093E-74	2	
Ndufs2	1,95923E-78	0,51313	0,762	0,47	3,28974E-74	2	
Mrpl48	2,86244E-78	0,4836	0,803	0,506	4,80632E-74	2	
Slc25a3	3,36105E-78	0,42456	0,998	0,957	5,64354E-74	2	
Blvrb	5,73602E-78	0,41831	0,535	0,251	9,63135E-74	2	
Ndufa12	9,40339E-78	0,48458	0,85	0,559	1,57892E-73	2	
Uchl1	1,27105E-77	0,32418	1	1	2,13422E-73	2	
Ppt1	1,34003E-77	0,51164	0,748	0,463	2,25005E-73	2	
Hspe1	2,03063E-77	0,49159	0,885	0,625	3,40964E-73	2	
Commd1	2,19368E-77	0,47265	0,735	0,432	3,68341E-73	2	
Sms	2,94407E-77	0,53453	0,825	0,578	4,94339E-73	2	
Mocs2	3,15896E-77	0,53252	0,819	0,565	5,3042E-73	2	
Mrps25	3,62081E-77	0,45084	0,637	0,348	6,0797E-73	2	
Anapc13	9,51661E-77	0,44158	0,712	0,407	1,59793E-72	2	
Emc7	1,21751E-76	0,46535	0,859	0,604	2,04433E-72	2	
Ndufa11	1,50093E-76	0,45599	0,983	0,881	2,52022E-72	2	
Prdx5	1,74649E-76	0,4841	0,922	0,702	2,93253E-72	2	
Srp19	2,83926E-76	0,48391	0,838	0,561	4,7674E-72	2	
Psmb4	3,22881E-76	0,48709	0,951	0,772	5,4215E-72	2	
Prdx1	5,40072E-76	0,42173	0,991	0,954	9,06836E-72	2	
Nfu1	6,70428E-76	0,42203	0,656	0,36	1,12572E-71	2	
Mrps18c	7,08788E-76	0,44603	0,671	0,371	1,19013E-71	2	
Tmem208	1,26451E-75	0,43495	0,646	0,348	2,12325E-71	2	
Serbp1	1,33981E-75	0,42289	0,992	0,952	2,24967E-71	2	
Pkig	3,14868E-75	0,40667	0,549	0,263	5,28694E-71	2	
C1d	3,15629E-75	0,45744	0,784	0,479	5,29973E-71	2	
Atp6v1d	3,62289E-75	0,47862	0,898	0,722	6,08319E-71	2	
Mrps26	3,6614E-75	0,44349	0,639	0,347	6,14785E-71	2	
Mrpl17	7,28083E-75	0,4346	0,646	0,345	1,22252E-70	2	
Ccdc124	1,19877E-74	0,46779	0,739	0,437	2,01286E-70	2	
Ppa2	1,58108E-74	0,425	0,595	0,306	2,65479E-70	2	
Naa38	1,74293E-74	0,42808	0,642	0,342	2,92656E-70	2	
Suclg1	2,23221E-74	0,45406	0,688	0,393	3,74811E-70	2	
Dync1i1	3,68849E-74	0,58348	0,79	0,531	6,19334E-70	2	
St13	4,31221E-74	0,4231	0,98	0,914	7,24063E-70	2	
Snrnp27	5,11832E-74	0,4432	0,977	0,882	8,59418E-70	2	
Bcas2	5,28913E-74	0,48045	0,833	0,566	8,88097E-70	2	
Surf1	5,47548E-74	0,42085	0,555	0,274	9,19388E-70	2	
Rpsa	8,25261E-74	0,38084	1	0,992	1,3857E-69	2	
Rpl5	9,39563E-74	0,44265	0,973	0,85	1,57762E-69	2	
Ube2e3	1,79585E-73	0,46927	0,715	0,437	3,01542E-69	2	
Ddx1	2,09414E-73	0,50992	0,87	0,624	3,51627E-69	2	
Trappc2l	2,1568E-73	0,46471	0,718	0,418	3,62148E-69	2	
Eif1	2,69263E-73	0,33224	1	0,998	4,5212E-69	2	
1110065P20	3,95422E-73	0,4428	0,675	0,374	6,63954E-69	2	
Fxyd6	4,57894E-73	0,38615	0,998	0,987	7,6885E-69	2	

Tshz2	5,82852E-73	0,43166	0,983	0,835	9,78667E-69	2	
Tmem160	8,49336E-73	0,44278	0,769	0,448	1,42612E-68	2	
Psmc5	9,00535E-73	0,4645	0,937	0,763	1,51209E-68	2	
Atp5d	9,45216E-73	0,44961	0,973	0,849	1,58711E-68	2	
Mien1	1,00379E-72	0,4511	0,697	0,405	1,68546E-68	2	
Vti1b	1,03259E-72	0,44745	0,746	0,45	1,73382E-68	2	
Cir1	1,17449E-72	0,49093	0,863	0,644	1,97208E-68	2	
Churc1	2,01906E-72	0,43673	0,72	0,42	3,3902E-68	2	
Slc25a5	2,51344E-72	0,46967	0,778	0,491	4,22032E-68	2	
Mrpl27	2,72576E-72	0,4364	0,66	0,367	4,57683E-68	2	
Snrpd1	4,55441E-72	0,45873	0,784	0,509	7,64732E-68	2	
Mrpl51	6,88616E-72	0,48953	0,838	0,588	1,15625E-67	2	
Wif1	1,18224E-71	0,32075	0,336	0,115	1,9851E-67	2	
Cct5	1,24102E-71	0,48126	0,892	0,679	2,0838E-67	2	
Il11ra1	1,2938E-71	0,49739	0,779	0,501	2,17243E-67	2	
Scg5	1,86361E-71	0,43481	0,962	0,833	3,12919E-67	2	
Rpf2	2,04423E-71	0,41967	0,583	0,304	3,43246E-67	2	
Bloc1s1	2,04447E-71	0,36374	0,394	0,156	3,43287E-67	2	
Sdhb	2,60752E-71	0,45933	0,824	0,554	4,37829E-67	2	
Nucb2	3,08453E-71	0,47178	0,752	0,477	5,17924E-67	2	
Chmp5	5,14549E-71	0,44737	0,916	0,708	8,63979E-67	2	
Psmc6	6,16114E-71	0,48231	0,885	0,685	1,03452E-66	2	
Ndufv1	7,02947E-71	0,46915	0,868	0,599	1,18032E-66	2	
St3gal6	8,56967E-71	0,3785	0,494	0,22	1,43893E-66	2	
Sugt1	1,43696E-70	0,44588	0,775	0,488	2,41279E-66	2	
Sdhd	1,46547E-70	0,42671	0,603	0,322	2,46067E-66	2	
Cops6	1,54475E-70	0,44715	0,857	0,56	2,59379E-66	2	
Smim8	1,62307E-70	0,38191	0,482	0,222	2,7253E-66	2	
Bcl2	2,81171E-70	0,37629	0,782	0,438	4,72114E-66	2	
Psma3	2,82636E-70	0,42972	0,961	0,853	4,74575E-66	2	
Tm7sf2	7,91724E-70	0,3849	0,421	0,179	1,32938E-65	2	
Snrpe	1,18494E-69	0,45999	0,829	0,57	1,98964E-65	2	
Grpel1	1,81704E-69	0,39231	0,653	0,355	3,051E-65	2	
Timm17a	3,07041E-69	0,4421	0,759	0,472	5,15553E-65	2	
Eif4ebp1	3,14886E-69	0,33491	0,375	0,146	5,28725E-65	2	
H3f3b	5,85581E-69	0,42265	0,982	0,899	9,8325E-65	2	
Psmc3	6,01354E-69	0,44177	0,935	0,744	1,00973E-64	2	
Hmgn2	6,62282E-69	0,42123	0,596	0,32	1,11204E-64	2	
Vim	9,13537E-69	0,56795	0,815	0,567	1,53392E-64	2	
Ufsp2	1,2503E-68	0,43481	0,614	0,339	2,09938E-64	2	
Tm2d1	1,40803E-68	0,45071	0,776	0,501	2,36423E-64	2	
Slc10a4	1,7267E-68	0,37768	0,973	0,791	2,8993E-64	2	
2810428I15R	1,97974E-68	0,47347	0,861	0,606	3,32417E-64	2	
Thoc7	2,70599E-68	0,47417	0,839	0,589	4,54363E-64	2	
Pop7	5,75949E-68	0,42614	0,682	0,394	9,67075E-64	2	
Nop10	6,36483E-68	0,45485	0,819	0,546	1,06872E-63	2	
Tm2d2	9,23482E-68	0,40456	0,603	0,325	1,55062E-63	2	
Cartpt	9,8206E-68	0,39626	0,436	0,192	1,64898E-63	2	

2700060E02	1,38415E-67	0,46088	0,763	0,491	2,32412E-63	2	
Nedd8	3,01133E-67	0,36707	0,996	0,976	5,05633E-63	2	
Cwc15	3,27372E-67	0,44738	0,766	0,494	5,49691E-63	2	
Hmx3	3,38524E-67	0,48555	0,657	0,392	5,68416E-63	2	
Dad1	3,94743E-67	0,44372	0,958	0,813	6,62812E-63	2	
Ssr4	4,53562E-67	0,45313	0,819	0,538	7,61577E-63	2	
Mrpl33	7,72849E-67	0,45144	0,834	0,559	1,29769E-62	2	
Cct4	9,52917E-67	0,41277	0,741	0,449	1,60004E-62	2	
Dgcr6	1,04716E-66	0,41982	0,692	0,404	1,75828E-62	2	
Swi5	1,09273E-66	0,43547	0,949	0,793	1,8348E-62	2	
Lrpap1	1,11946E-66	0,44494	0,894	0,694	1,87968E-62	2	
Hint2	2,02374E-66	0,39675	0,605	0,327	3,39807E-62	2	
Psmd12	3,25116E-66	0,44897	0,841	0,578	5,45902E-62	2	
Mrpl14	1,06988E-65	0,4075	0,708	0,42	1,79643E-61	2	
Snrpc	1,8202E-65	0,38396	0,554	0,285	3,05631E-61	2	
Arpc5l	2,55217E-65	0,44078	0,757	0,49	4,28535E-61	2	
Mrps18a	4,00739E-65	0,384	0,552	0,286	6,72882E-61	2	
Zmat2	5,70559E-65	0,44964	0,854	0,608	9,58026E-61	2	
Hspb2	6,10128E-65	0,28011	0,269	0,087	1,02447E-60	2	
Eif3m	1,82371E-64	0,41457	0,748	0,462	3,06219E-60	2	
Pfdn4	4,7895E-64	0,4431	0,762	0,498	8,04206E-60	2	
Ldha	5,03959E-64	0,3625	0,998	0,973	8,46198E-60	2	
Gstp1	9,7586E-64	0,32652	0,367	0,148	1,63857E-59	2	
Clta	1,1551E-63	0,37727	0,989	0,949	1,93952E-59	2	
Prdx4	1,23663E-63	0,3742	0,455	0,21	2,07642E-59	2	
Tmbim4	3,71458E-63	0,41751	0,734	0,454	6,23715E-59	2	
Cst3	4,01339E-63	0,37303	0,994	0,932	6,73888E-59	2	
Fkbp2	5,95454E-63	0,42345	0,914	0,729	9,99827E-59	2	
Dbt	9,58893E-63	0,3608	0,459	0,211	1,61008E-58	2	
Ntan1	9,90228E-63	0,40475	0,649	0,381	1,66269E-58	2	
Chchd1	1,19451E-62	0,3945	0,584	0,316	2,0057E-58	2	
Mrpl11	1,45901E-62	0,4041	0,589	0,325	2,44982E-58	2	
Mrps36	3,26901E-62	0,40432	0,627	0,357	5,489E-58	2	
Pdap1	5,52727E-62	0,39883	0,99	0,935	9,28084E-58	2	
Arpc3	8,32483E-62	0,40086	0,95	0,765	1,39782E-57	2	
Eapp	1,03315E-61	0,41625	0,797	0,527	1,73475E-57	2	
Tmem5	1,47385E-61	0,45063	0,785	0,536	2,47474E-57	2	
Mrpl12	1,55001E-61	0,40269	0,672	0,401	2,60262E-57	2	
Mrpl18	1,74934E-61	0,36445	0,431	0,199	2,93731E-57	2	
Tmed10	2,87325E-61	0,44068	0,892	0,719	4,82448E-57	2	
Mrpl41	3,53175E-61	0,41769	0,741	0,458	5,93016E-57	2	
Gpx1	5,53109E-61	0,40072	0,578	0,316	9,28726E-57	2	
Sfr1	8,47496E-61	0,44936	0,877	0,673	1,42303E-56	2	
Etfb	1,61378E-60	0,40649	0,701	0,428	2,7097E-56	2	
H3f3a	2,27899E-60	0,31768	1	0,986	3,82665E-56	2	
Dfna5	2,37425E-60	0,44376	0,654	0,403	3,9866E-56	2	
Eef2	2,54002E-60	0,36929	0,997	0,946	4,26495E-56	2	
Esd	3,04847E-60	0,42237	0,79	0,536	5,11869E-56	2	

Anapc16	4,15486E-60	0,39069	0,552	0,302	6,97643E-56	2	
Rspo2	4,43987E-60	0,31455	0,5	0,228	7,45499E-56	2	
Mrpl13	5,84463E-60	0,40108	0,659	0,389	9,81371E-56	2	
Tcaf1	7,71082E-60	0,37017	0,976	0,92	1,29472E-55	2	
Dtd1	1,12004E-59	0,38818	0,545	0,296	1,88066E-55	2	
Wdr45b	2,42206E-59	0,4191	0,879	0,692	4,06688E-55	2	
Drap1	2,51843E-59	0,42814	0,875	0,686	4,2287E-55	2	
Ap4s1	3,69311E-59	0,34148	0,465	0,222	6,2011E-55	2	
Nudcd3	4,68644E-59	0,35989	0,987	0,938	7,869E-55	2	
Mrps21	6,88152E-59	0,4029	0,695	0,438	1,15548E-54	2	
Mrpl30	8,94389E-59	0,42146	0,722	0,465	1,50177E-54	2	
Txndc17	1,03402E-58	0,39948	0,904	0,679	1,73622E-54	2	
Fam173a	1,26859E-58	0,40342	0,619	0,359	2,13009E-54	2	
Ctxn1	1,51723E-58	0,44046	0,86	0,645	2,54758E-54	2	
Mpp6	1,80342E-58	0,40127	0,521	0,276	3,02812E-54	2	
2700029M09	2,99381E-58	0,38445	0,513	0,268	5,0269E-54	2	
Arhgap20	3,02077E-58	0,36309	0,54	0,28	5,07217E-54	2	
Mrpl34	3,26395E-58	0,36794	0,525	0,274	5,48049E-54	2	
Chchd6	3,36085E-58	0,36672	0,538	0,288	5,6432E-54	2	
Gtf2h5	4,55836E-58	0,42134	0,836	0,606	7,65395E-54	2	
Mgst3	1,03473E-57	0,37444	0,592	0,332	1,73742E-53	2	
Ndufa8	1,21027E-57	0,40595	0,859	0,649	2,03216E-53	2	
Prmt8	1,50971E-57	0,27867	0,33	0,127	2,53495E-53	2	
Nhp2l1	1,55953E-57	0,42778	0,825	0,599	2,61861E-53	2	
Psmb2	1,85286E-57	0,39546	0,892	0,686	3,11113E-53	2	
Uqcrfs1	2,48533E-57	0,40274	0,784	0,514	4,17312E-53	2	
Mrpl42	2,49808E-57	0,3589	0,61	0,34	4,19452E-53	2	
Crip2	4,04808E-57	0,33337	1	0,993	6,79713E-53	2	
Emc9	4,55233E-57	0,38763	0,633	0,375	7,64381E-53	2	
Cebpzos	5,12898E-57	0,35142	0,462	0,228	8,61207E-53	2	
S100a13	5,48113E-57	0,40657	0,981	0,891	9,20336E-53	2	
Emc6	6,17428E-57	0,35532	0,608	0,345	1,03672E-52	2	
Polr2e	6,32765E-57	0,36847	0,629	0,372	1,06248E-52	2	
Mrps34	7,77024E-57	0,35639	0,532	0,282	1,3047E-52	2	
Bex1	8,82677E-57	0,45104	0,902	0,755	1,4821E-52	2	
Rpl4	9,001E-57	0,38958	0,957	0,842	1,51136E-52	2	
0610012G03	1,0486E-56	0,39285	0,706	0,439	1,76071E-52	2	
Caml	1,35707E-56	0,43368	0,729	0,495	2,27865E-52	2	
Hspa8	1,43861E-56	0,35647	1	0,994	2,41557E-52	2	
Rpl15	2,93596E-56	0,4082	0,706	0,454	4,92978E-52	2	
Tomm20	4,44753E-56	0,37686	0,943	0,833	7,46785E-52	2	
Sec11a	7,24108E-56	0,37568	0,615	0,362	1,21585E-51	2	
Id2	9,54484E-56	0,43148	0,958	0,879	1,60267E-51	2	
Uqcrc2	2,08675E-55	0,41281	0,748	0,497	3,50386E-51	2	
Ap2b1	4,18035E-55	0,37228	0,992	0,933	7,01922E-51	2	
Caln1	6,69058E-55	0,2654	0,416	0,18	1,12341E-50	2	
Mrps33	7,67965E-55	0,40414	0,933	0,753	1,28949E-50	2	
Raly	1,28324E-54	0,39157	0,82	0,584	2,15468E-50	2	

Mrpl21	1,38221E-54	0,37346	0,583	0,332	2,32087E-50	2	
Scg3	1,57239E-54	0,38199	0,99	0,951	2,64021E-50	2	
Serp2	1,90884E-54	0,39061	0,706	0,451	3,20513E-50	2	
Mrpl43	2,19394E-54	0,34693	0,627	0,365	3,68384E-50	2	
Lgals1	2,52581E-54	0,36453	0,986	0,914	4,24109E-50	2	
Tceb1	3,36889E-54	0,39559	0,818	0,595	5,6567E-50	2	
Sh3bgrl3	3,55999E-54	0,34894	0,885	0,605	5,97757E-50	2	
Bax	4,96351E-54	0,36557	0,671	0,411	8,33423E-50	2	
Anxa5	5,40387E-54	0,42827	0,857	0,69	9,07364E-50	2	
Cbr1	8,0076E-54	0,37972	0,701	0,439	1,34456E-49	2	
Psma4	8,39823E-54	0,39693	0,911	0,759	1,41015E-49	2	
Ccdc137	8,83183E-54	0,37846	0,582	0,338	1,48295E-49	2	
Pcmt1	1,2681E-53	0,3832	0,905	0,735	2,12927E-49	2	
Rpl23a	1,61771E-53	0,39285	0,865	0,654	2,7163E-49	2	
N6amt2	2,15061E-53	0,34827	0,519	0,28	3,61109E-49	2	
Ckb	3,8227E-53	0,39021	0,91	0,718	6,41869E-49	2	
Slc5a7	3,82551E-53	0,3278	0,866	0,598	6,42341E-49	2	
Hspd1	3,91299E-53	0,42035	0,818	0,626	6,5703E-49	2	
Scrn1	5,81554E-53	0,32849	0,458	0,228	9,76487E-49	2	
Psd3	7,34621E-53	0,30216	0,472	0,231	1,2335E-48	2	
Snrpb2	8,56899E-53	0,34365	0,684	0,416	1,43882E-48	2	
Phyh	9,06429E-53	0,40061	0,642	0,398	1,52198E-48	2	
Ncl	1,12808E-52	0,35795	0,993	0,961	1,89415E-48	2	
Rsph9	1,20479E-52	0,35598	0,596	0,345	2,02296E-48	2	
Ccdc167	1,35624E-52	0,29386	0,343	0,146	2,27727E-48	2	
Commd4	1,43423E-52	0,32166	0,433	0,21	2,40821E-48	2	
Eif3j1	1,63933E-52	0,38701	0,907	0,731	2,7526E-48	2	
Arhgap5	2,25356E-52	0,37501	0,649	0,389	3,78396E-48	2	
Aldoc	2,90026E-52	0,39676	0,614	0,372	4,86983E-48	2	
Tomm22	3,28655E-52	0,34403	0,642	0,382	5,51844E-48	2	
B4gat1	4,27767E-52	0,35568	0,551	0,313	7,18264E-48	2	
Hsd17b10	4,37494E-52	0,3436	0,574	0,324	7,34596E-48	2	
Mrps12	5,18081E-52	0,35737	0,726	0,467	8,6991E-48	2	
Ebpl	5,81962E-52	0,34198	0,466	0,239	9,77173E-48	2	
Abcd3	7,26901E-52	0,39839	0,782	0,553	1,22054E-47	2	
Tmem147	7,32678E-52	0,38328	0,779	0,538	1,23024E-47	2	
Carkd	7,4245E-52	0,32803	0,519	0,275	1,24665E-47	2	
Mrpl54	8,22326E-52	0,38471	0,755	0,506	1,38077E-47	2	
Timm10	1,10798E-51	0,34493	0,543	0,302	1,86042E-47	2	
Apoa1bp	1,27628E-51	0,32937	0,499	0,259	2,143E-47	2	
Serf1	1,27701E-51	0,37989	0,94	0,797	2,14423E-47	2	
Wbp5	1,83539E-51	0,36451	0,983	0,94	3,0818E-47	2	
Ddx24	2,40633E-51	0,40451	0,9	0,777	4,04046E-47	2	
Tceal3	2,44787E-51	0,40097	0,735	0,487	4,11023E-47	2	
Pigp	2,69023E-51	0,35273	0,589	0,34	4,51716E-47	2	
Morf4l1	3,95401E-51	0,28457	1	0,999	6,63918E-47	2	
Npm1	4,18375E-51	0,36191	0,967	0,881	7,02493E-47	2	
Arl6ip5	4,63083E-51	0,38302	0,704	0,468	7,77562E-47	2	

Hmgn1	4,74437E-51	0,39537	0,829	0,593	7,96627E-47	2	
Snhg6	8,2722E-51	0,30258	0,417	0,201	1,38898E-46	2	
Trappc1	8,84019E-51	0,32656	0,535	0,29	1,48436E-46	2	
Zfp639	8,86087E-51	0,35216	0,534	0,303	1,48783E-46	2	
Txnl1	1,07096E-50	0,39042	0,839	0,653	1,79826E-46	2	
Snrpf	1,59563E-50	0,38184	0,928	0,769	2,67923E-46	2	
Sar1b	1,82328E-50	0,34558	0,613	0,369	3,06147E-46	2	
Pnkd	3,1738E-50	0,36752	0,746	0,502	5,32912E-46	2	
Khdrbs3	3,50746E-50	0,33375	0,55	0,312	5,88937E-46	2	
Nabp2	3,6275E-50	0,34327	0,568	0,325	6,09093E-46	2	
Mcts1	4,11165E-50	0,36338	0,586	0,349	6,90387E-46	2	
Abhd17a	4,41197E-50	0,35644	0,715	0,465	7,40814E-46	2	
Rpgrip1	4,68636E-50	0,3357	0,448	0,228	7,86886E-46	2	
Mrps5	4,9098E-50	0,34933	0,728	0,483	8,24404E-46	2	
Nrp2	5,65552E-50	0,25142	0,551	0,275	9,49619E-46	2	
Hacd1	6,31039E-50	0,34044	0,528	0,291	1,05958E-45	2	
Atp5g3	9,80799E-50	0,37526	0,933	0,787	1,64686E-45	2	
Gpx4	9,94753E-50	0,32489	0,998	0,974	1,67029E-45	2	
Rpl14	1,19072E-49	0,32257	1	0,983	1,99934E-45	2	
Eif3g	1,33298E-49	0,3523	0,703	0,447	2,2382E-45	2	
Bnip3	1,33765E-49	0,38344	0,67	0,443	2,24605E-45	2	
Ostc	1,55562E-49	0,33735	0,661	0,406	2,61203E-45	2	
Sra1	3,97123E-49	0,36389	0,669	0,424	6,66809E-45	2	
Slc25a11	4,19297E-49	0,36517	0,663	0,426	7,04042E-45	2	
Rwdd1	4,30003E-49	0,3841	0,772	0,533	7,22018E-45	2	
Manbal	4,48971E-49	0,3782	0,694	0,458	7,53867E-45	2	
Psmc2	6,08082E-49	0,36379	0,917	0,743	1,02103E-44	2	
Evl	9,67992E-49	0,35523	0,839	0,61	1,62536E-44	2	
Ahi1	1,31164E-48	0,28133	1	1	2,20237E-44	2	
Smarca2	1,31812E-48	0,28476	0,993	0,942	2,21325E-44	2	
Tmem176b	1,83353E-48	0,34887	0,981	0,937	3,07868E-44	2	
Praf2	2,80723E-48	0,3368	0,548	0,313	4,71362E-44	2	
Mphosph8	4,16052E-48	0,39784	0,794	0,569	6,98592E-44	2	
Rps27l	4,34406E-48	0,36019	0,826	0,598	7,29412E-44	2	
Kif21a	5,51045E-48	0,324	0,988	0,974	9,25259E-44	2	
Tomm5	5,65726E-48	0,33873	0,62	0,378	9,4991E-44	2	
Psmg4	5,79223E-48	0,34775	0,626	0,385	9,72573E-44	2	
Pdcd4	5,91809E-48	0,4099	0,774	0,552	9,93706E-44	2	
Tmem59l	6,21061E-48	0,36094	0,618	0,372	1,04282E-43	2	
Tmem205	7,69348E-48	0,3473	0,612	0,368	1,29181E-43	2	
Ppp1ca	8,93803E-48	0,35021	0,951	0,84	1,50079E-43	2	
Ube2s	9,90782E-48	0,37656	0,916	0,784	1,66362E-43	2	
Snrpb	1,08126E-47	0,37629	0,864	0,663	1,81555E-43	2	
Rufy3	1,28049E-47	0,28734	1	0,99	2,15008E-43	2	
Vimp	1,47859E-47	0,3592	0,645	0,4	2,4827E-43	2	
Smim20	1,48248E-47	0,27371	0,374	0,174	2,48924E-43	2	
Mycbp	1,69455E-47	0,33819	0,635	0,386	2,84532E-43	2	
Nt5c	2,07617E-47	0,34002	0,604	0,36	3,4861E-43	2	

Fam195b	2,11893E-47	0,36192	0,851	0,608	3,5579E-43	2	
Ubash3b	2,59803E-47	0,34004	0,459	0,243	4,36235E-43	2	
Mrpl36	3,10142E-47	0,32125	0,552	0,315	5,2076E-43	2	
Cetn3	3,21732E-47	0,38135	0,755	0,534	5,40219E-43	2	
Ttc9b	3,44099E-47	0,3527	0,817	0,566	5,77777E-43	2	
Polr2f	3,61258E-47	0,36113	0,751	0,511	6,06588E-43	2	
Mrpl57	3,85243E-47	0,33239	0,676	0,424	6,46861E-43	2	
Fam103a1	4,85389E-47	0,3837	0,792	0,58	8,15017E-43	2	
Abhd14a	4,91984E-47	0,31186	0,466	0,247	8,26091E-43	2	
Commd3	6,0348E-47	0,32695	0,529	0,299	1,0133E-42	2	
Phf5a	7,30395E-47	0,33808	0,619	0,378	1,22641E-42	2	
Emp3	9,11527E-47	0,31388	0,313	0,134	1,53054E-42	2	
Borcs7	9,12651E-47	0,3424	0,564	0,338	1,53243E-42	2	
Cxx1b	1,26402E-46	0,3833	0,796	0,603	2,12242E-42	2	
Cope	2,39313E-46	0,36357	0,812	0,578	4,01831E-42	2	
Ndufs7	2,72933E-46	0,37465	0,838	0,597	4,58282E-42	2	
Snx6	2,74581E-46	0,35336	0,625	0,388	4,61049E-42	2	
Psmd14	4,25896E-46	0,35827	0,673	0,441	7,15122E-42	2	
Atp5a1	4,83044E-46	0,33625	0,961	0,869	8,11079E-42	2	
Psma6	5,98621E-46	0,36421	0,819	0,581	1,00514E-41	2	
Lcmt1	5,99599E-46	0,34154	0,601	0,365	1,00679E-41	2	
Mrps15	7,30055E-46	0,31012	0,569	0,335	1,22584E-41	2	
Sema3c	9,89684E-46	0,26044	0,468	0,236	1,66178E-41	2	
Utp11l	1,21535E-45	0,35335	0,542	0,32	2,0407E-41	2	
Ndufs8	1,3603E-45	0,33972	0,722	0,474	2,28409E-41	2	
BC029214	1,37497E-45	0,31962	0,542	0,309	2,30872E-41	2	
Wbp11	2,02062E-45	0,35914	0,758	0,529	3,39282E-41	2	
Mrps16	2,97598E-45	0,32913	0,581	0,352	4,99696E-41	2	
Glrx2	3,14532E-45	0,33234	0,64	0,409	5,2813E-41	2	
Xaf1	3,42004E-45	0,40057	0,635	0,42	5,74258E-41	2	
Rala	3,45128E-45	0,35854	0,715	0,488	5,79504E-41	2	
Nsrp1	3,74238E-45	0,31876	0,642	0,392	6,28383E-41	2	
Arl2	4,44497E-45	0,36963	0,767	0,555	7,46355E-41	2	
Cox20	4,92931E-45	0,32963	0,496	0,272	8,27681E-41	2	
Metap2	5,32519E-45	0,30547	0,991	0,968	8,94153E-41	2	
Rbm22	6,52335E-45	0,34576	0,644	0,418	1,09534E-40	2	
Hmx2	7,33677E-45	0,3673	0,506	0,29	1,23192E-40	2	
Snrpg	1,03167E-44	0,33244	0,602	0,369	1,73227E-40	2	
Zc3h15	1,20984E-44	0,33388	0,977	0,899	2,03144E-40	2	
Glo1	1,72331E-44	0,35502	0,661	0,432	2,89361E-40	2	
Brinp1	1,77027E-44	0,30245	0,58	0,333	2,97246E-40	2	
Tmem126a	2,34831E-44	0,30205	0,552	0,32	3,94305E-40	2	
Chchd4	2,71979E-44	0,32905	0,555	0,332	4,56679E-40	2	
Nlgn1	2,78341E-44	0,29585	0,647	0,394	4,67362E-40	2	
Bad	3,98102E-44	0,32566	0,57	0,343	6,68453E-40	2	
Nap1l5	5,09506E-44	0,35036	0,978	0,94	8,55511E-40	2	
Hmgb3	5,12531E-44	0,38113	0,672	0,455	8,60591E-40	2	
Mrfap1	5,51403E-44	0,36032	0,911	0,772	9,2586E-40	2	

Dtymk	5,88807E-44	0,31104	0,491	0,276	9,88666E-40	2	
Rgs9	7,38781E-44	0,37457	0,884	0,743	1,24049E-39	2	
2210016L21R	8,39377E-44	0,34835	0,846	0,659	1,4094E-39	2	
Sucla2	8,71015E-44	0,3604	0,724	0,499	1,46252E-39	2	
Rheb	9,57295E-44	0,34839	0,874	0,734	1,60739E-39	2	
Eef1g	1,02881E-43	0,34014	0,951	0,857	1,72747E-39	2	
Rnf187	1,10655E-43	0,31927	0,968	0,911	1,858E-39	2	
Borcs8	1,20812E-43	0,29151	0,428	0,223	2,02855E-39	2	
Mrpl15	1,46291E-43	0,31801	0,525	0,306	2,45637E-39	2	
Zcchc10	1,74808E-43	0,31291	0,574	0,348	2,9352E-39	2	
Erlec1	3,04783E-43	0,31398	0,558	0,337	5,1176E-39	2	
Rps19bp1	3,05882E-43	0,28118	0,466	0,25	5,13607E-39	2	
Pafah1b1	3,6054E-43	0,27771	0,997	0,988	6,05382E-39	2	
Scp2	3,67772E-43	0,34812	0,854	0,664	6,17526E-39	2	
Elof1	4,46595E-43	0,32745	0,63	0,401	7,49878E-39	2	
Rer1	6,14818E-43	0,34165	0,72	0,479	1,03234E-38	2	
1700023F06	8,59425E-43	0,34398	0,588	0,368	1,44306E-38	2	
Guk1	1,40701E-42	0,35543	0,735	0,514	2,36251E-38	2	
Marc2	1,40853E-42	0,29706	0,435	0,232	2,36506E-38	2	
Smpdl3a	1,67626E-42	0,30156	0,499	0,284	2,81462E-38	2	
Lhfpl5	1,70403E-42	0,32585	0,601	0,373	2,86124E-38	2	
Pts	1,78349E-42	0,29467	0,435	0,231	2,99466E-38	2	
Emc10	2,12869E-42	0,3268	0,935	0,759	3,57429E-38	2	
Psma2	2,13742E-42	0,32494	0,939	0,816	3,58895E-38	2	
Mrps18b	2,63695E-42	0,29628	0,41	0,214	4,4277E-38	2	
Hsbp1	2,75428E-42	0,35121	0,895	0,717	4,62472E-38	2	
Pin1	4,06223E-42	0,3184	0,703	0,467	6,82089E-38	2	
Cox14	4,06925E-42	0,34427	0,732	0,5	6,83268E-38	2	
Zcrb1	4,20339E-42	0,34472	0,764	0,537	7,05791E-38	2	
Gm561	4,54811E-42	0,31637	0,524	0,304	7,63674E-38	2	
Atraid	5,03503E-42	0,31246	0,604	0,38	8,45432E-38	2	
Cdc37	5,63339E-42	0,33631	0,806	0,592	9,45902E-38	2	
0610011F06	7,23455E-42	0,2967	0,499	0,283	1,21475E-37	2	
Tmem14c	1,12009E-41	0,31767	0,537	0,318	1,88074E-37	2	
Tpi1	1,31034E-41	0,28425	0,969	0,874	2,20018E-37	2	
Pdcl3	1,31036E-41	0,32406	0,522	0,316	2,20023E-37	2	
Cdk5rap3	1,36663E-41	0,27847	0,436	0,232	2,29471E-37	2	
Adh5	1,90469E-41	0,29905	0,596	0,367	3,19817E-37	2	
Pdzd11	2,1021E-41	0,28427	0,44	0,236	3,52963E-37	2	
Psmb7	2,31812E-41	0,35872	0,885	0,684	3,89236E-37	2	
Grina	4,40167E-41	0,30157	0,975	0,917	7,39085E-37	2	
Sdhaf4	4,7146E-41	0,33934	0,659	0,447	7,91628E-37	2	
Ssna1	5,35289E-41	0,2951	0,6	0,377	8,98804E-37	2	
Plpp1	5,70679E-41	0,34126	0,712	0,48	9,58228E-37	2	
Egfl7	6,27483E-41	0,26683	0,369	0,183	1,05361E-36	2	
1110032A03	7,00313E-41	0,30444	0,435	0,238	1,1759E-36	2	
Ednrb	7,67177E-41	0,28283	0,326	0,149	1,28817E-36	2	
Med28	8,2335E-41	0,31946	0,722	0,491	1,38249E-36	2	

Rab14	9,23819E-41	0,31894	0,956	0,899	1,55118E-36	2	
Cript	9,61362E-41	0,34163	0,786	0,57	1,61422E-36	2	
Snf8	1,21264E-40	0,31462	0,599	0,381	2,03614E-36	2	
Emc4	1,33394E-40	0,3296	0,643	0,422	2,23982E-36	2	
Sssca1	1,61646E-40	0,29287	0,524	0,307	2,71419E-36	2	
Myl12b	1,84395E-40	0,33718	0,931	0,829	3,09617E-36	2	
Psmd4	2,66786E-40	0,33789	0,906	0,772	4,47961E-36	2	
Lysmd2	3,7033E-40	0,31433	0,604	0,39	6,21822E-36	2	
Agpat4	4,05209E-40	0,32597	0,538	0,329	6,80387E-36	2	
Polr2c	4,12846E-40	0,2961	0,61	0,389	6,9321E-36	2	
Mcee	4,32793E-40	0,33008	0,625	0,407	7,26702E-36	2	
Nudc	5,80916E-40	0,33166	0,861	0,69	9,75416E-36	2	
Bola1	5,90042E-40	0,29347	0,441	0,243	9,90739E-36	2	
Rbm39	6,53335E-40	0,27676	0,99	0,966	1,09701E-35	2	
Rab24	7,2777E-40	0,29408	0,53	0,314	1,222E-35	2	
Ola1	9,4715E-40	0,3193	0,691	0,468	1,59036E-35	2	
Pafah1b3	1,07485E-39	0,34322	0,761	0,547	1,80478E-35	2	
Yif1b	1,19459E-39	0,32589	0,691	0,458	2,00583E-35	2	
Arl6ip4	1,22404E-39	0,3184	0,54	0,334	2,05528E-35	2	
Mmgt2	1,41475E-39	0,33012	0,657	0,447	2,3755E-35	2	
Psma5	1,87854E-39	0,33254	0,776	0,568	3,15426E-35	2	
Flywch2	1,9764E-39	0,31895	0,531	0,319	3,31858E-35	2	
Ktn1	2,68684E-39	0,29628	0,98	0,942	4,51148E-35	2	
Yipf1	2,78078E-39	0,26878	0,431	0,233	4,6692E-35	2	
Dnm1l	2,80368E-39	0,33039	0,969	0,919	4,70765E-35	2	
Cntln	2,94292E-39	0,30079	0,521	0,305	4,94145E-35	2	
Hdhd2	4,63141E-39	0,29806	0,549	0,339	7,7766E-35	2	
Vps29	4,8751E-39	0,33245	0,752	0,537	8,18578E-35	2	
Tbcc	4,89019E-39	0,26956	0,498	0,285	8,21112E-35	2	
Hist3h2ba	5,4981E-39	0,29877	0,713	0,464	9,23185E-35	2	
Tmem199	6,31494E-39	0,30077	0,472	0,273	1,06034E-34	2	
B3gnt2	7,61247E-39	0,29033	0,482	0,277	1,27821E-34	2	
Clybl	7,89897E-39	0,28208	0,408	0,217	1,32632E-34	2	
Sap30l	9,48022E-39	0,33949	0,716	0,505	1,59182E-34	2	
Coprs	9,71881E-39	0,30108	0,544	0,332	1,63189E-34	2	
Eif3f	9,95867E-39	0,35011	0,847	0,672	1,67216E-34	2	
Riiad1	1,05674E-38	0,29356	0,499	0,29	1,77437E-34	2	
Jtb	1,13441E-38	0,26895	0,427	0,231	1,90478E-34	2	
Cbx3	1,87885E-38	0,31104	0,966	0,928	3,15478E-34	2	
Ech1	2,5867E-38	0,28817	0,608	0,384	4,34332E-34	2	
Cct7	2,76219E-38	0,27334	0,991	0,924	4,63799E-34	2	
Pop5	2,77022E-38	0,30108	0,612	0,393	4,65147E-34	2	
Lsm12	2,96784E-38	0,30498	0,645	0,429	4,98329E-34	2	
Trappc6b	3,35634E-38	0,3095	0,867	0,719	5,63562E-34	2	
Sec11c	3,38876E-38	0,29585	0,407	0,219	5,69006E-34	2	
Fdx1	3,41574E-38	0,27408	0,449	0,249	5,73536E-34	2	
Psmc4	5,3402E-38	0,31994	0,749	0,531	8,96673E-34	2	
Polr2g	5,48634E-38	0,30486	0,68	0,462	9,21211E-34	2	

Zfp330	5,84609E-38	0,29877	0,519	0,314	9,81616E-34	2	
Tsr3	6,78646E-38	0,25299	0,397	0,207	1,13952E-33	2	
Polr2k	7,04897E-38	0,33066	0,754	0,541	1,18359E-33	2	
Morn2	1,09726E-37	0,29682	0,483	0,284	1,8424E-33	2	
Rpp21	1,15217E-37	0,29885	0,551	0,341	1,93461E-33	2	
1110008F13	1,36167E-37	0,27914	0,441	0,246	2,28638E-33	2	
Fam92a	1,39821E-37	0,31485	0,646	0,437	2,34773E-33	2	
Hspa5	1,50381E-37	0,31896	0,927	0,814	2,52504E-33	2	
Mllt11	1,61585E-37	0,30499	0,978	0,934	2,71317E-33	2	
Irak1bp1	1,81953E-37	0,27112	0,424	0,232	3,05517E-33	2	
Gcgr	1,83002E-37	0,28546	0,524	0,304	3,07279E-33	2	
Sgip1	2,02783E-37	0,33566	0,935	0,839	3,40493E-33	2	
Cyb5a	2,09433E-37	0,32356	0,794	0,593	3,51659E-33	2	
Cmpk1	2,14369E-37	0,31456	0,894	0,765	3,59947E-33	2	
Serf2	2,38887E-37	0,29487	0,96	0,882	4,01115E-33	2	
Frg1	2,81014E-37	0,32624	0,816	0,626	4,71851E-33	2	
Akr1b3	3,14149E-37	0,32609	0,881	0,71	5,27488E-33	2	
Mrps28	3,39534E-37	0,25768	0,365	0,186	5,70111E-33	2	
Zfp771	4,07896E-37	0,27119	0,549	0,332	6,84898E-33	2	
Ifi35	5,50862E-37	0,30404	0,629	0,41	9,24953E-33	2	
Rabggtb	5,76858E-37	0,29853	0,619	0,404	9,68602E-33	2	
Brk1	6,05467E-37	0,31791	0,742	0,527	1,01664E-32	2	
H2afv	6,76203E-37	0,28097	0,508	0,305	1,13541E-32	2	
Fra10ac1	7,70927E-37	0,31156	0,45	0,262	1,29446E-32	2	
Apopt1	8,52179E-37	0,32459	0,573	0,378	1,43089E-32	2	
Ndufa9	1,14066E-36	0,28949	0,596	0,378	1,91528E-32	2	
Polr2j	1,27324E-36	0,31384	0,678	0,479	2,1379E-32	2	
Ift27	1,71278E-36	0,27052	0,456	0,261	2,87593E-32	2	
Erp29	1,99101E-36	0,31278	0,924	0,78	3,34311E-32	2	
Ctsl	2,01598E-36	0,29818	0,943	0,88	3,38503E-32	2	
Clu	2,04256E-36	0,33332	0,619	0,401	3,42966E-32	2	
Krtcap2	2,18258E-36	0,308	0,841	0,63	3,66478E-32	2	
Ift57	2,9433E-36	0,31296	0,611	0,407	4,9421E-32	2	
Uqcc3	3,35396E-36	0,29548	0,51	0,309	5,63163E-32	2	
Tm2d3	3,40718E-36	0,27635	0,506	0,304	5,72099E-32	2	
Chrac1	3,54546E-36	0,3073	0,551	0,344	5,95318E-32	2	
Wdr83os	3,97692E-36	0,28617	0,569	0,356	6,67764E-32	2	
Zcchc18	4,44398E-36	0,28602	0,973	0,958	7,46189E-32	2	
Glrx5	4,59338E-36	0,28264	0,611	0,385	7,71274E-32	2	
Ctnnbip1	4,65134E-36	0,30218	0,695	0,471	7,81006E-32	2	
Exoc6	5,12562E-36	0,29372	0,458	0,269	8,60643E-32	2	
Wdr61	6,43366E-36	0,30962	0,71	0,499	1,08028E-31	2	
Tusc3	6,53963E-36	0,30627	0,902	0,764	1,09807E-31	2	
Eif3i	7,1558E-36	0,32431	0,8	0,597	1,20153E-31	2	
Bex4	7,63886E-36	0,3062	0,715	0,501	1,28264E-31	2	
Irf2	8,78587E-36	0,31808	0,711	0,507	1,47524E-31	2	
Fam133b	8,98761E-36	0,32423	0,785	0,596	1,50911E-31	2	
Rnh1	9,47528E-36	0,30085	0,555	0,351	1,59099E-31	2	

Ndufa10	1,15491E-35	0,31305	0,795	0,618	1,9392E-31	2	
Gm8730	1,3103E-35	0,34732	0,614	0,407	2,20013E-31	2	
Snw1	1,74195E-35	0,31471	0,693	0,487	2,92491E-31	2	
Bud31	2,39548E-35	0,27523	0,49	0,292	4,02224E-31	2	
Got1	2,63719E-35	0,30984	0,522	0,333	4,4281E-31	2	
Banf1	2,77222E-35	0,2961	0,669	0,455	4,65484E-31	2	
Mrpl55	3,14053E-35	0,27877	0,483	0,288	5,27326E-31	2	
Ldhb	3,86429E-35	0,29259	0,5	0,302	6,48853E-31	2	
Syf2	4,14662E-35	0,32629	0,769	0,579	6,9626E-31	2	
Cnih1	4,85319E-35	0,28832	0,576	0,37	8,14899E-31	2	
Phax	5,6621E-35	0,27945	0,646	0,428	9,50723E-31	2	
Eno1	5,83756E-35	0,30344	0,782	0,563	9,80185E-31	2	
Ndufs3	8,39957E-35	0,28897	0,739	0,51	1,41037E-30	2	
Hoxb2	8,64965E-35	0,29944	0,764	0,557	1,45236E-30	2	
Acbd6	9,53071E-35	0,28356	0,603	0,397	1,6003E-30	2	
Hsd17b12	9,82071E-35	0,34114	0,782	0,625	1,649E-30	2	
Pgls	1,09456E-34	0,29265	0,766	0,543	1,83787E-30	2	
Fndc1	1,22618E-34	0,27604	0,45	0,26	2,05888E-30	2	
Svil	1,23408E-34	0,30643	0,707	0,499	2,07214E-30	2	
E530001K10	1,28962E-34	0,30255	0,681	0,475	2,1654E-30	2	
Tbca	1,41534E-34	0,27796	0,932	0,834	2,3765E-30	2	
Hprt	1,4402E-34	0,32496	0,771	0,598	2,41824E-30	2	
Slc24a5	1,47538E-34	0,26057	0,359	0,189	2,47731E-30	2	
Nudt2	1,65201E-34	0,2541	0,344	0,178	2,7739E-30	2	
Spcs2	1,72242E-34	0,31734	0,817	0,654	2,89212E-30	2	
Atp1a1	2,01626E-34	0,28193	1	0,997	3,3855E-30	2	
Hypk	2,21982E-34	0,28286	0,541	0,342	3,72731E-30	2	
Rab6b	3,12759E-34	0,25203	0,966	0,916	5,25153E-30	2	
Ntm	3,3891E-34	0,31879	0,306	0,145	5,69064E-30	2	
Prkrip1	3,83262E-34	0,28717	0,449	0,266	6,43535E-30	2	
Rom1	4,15625E-34	0,27976	0,436	0,253	6,97876E-30	2	
Nt5m	4,31103E-34	0,26111	0,399	0,221	7,23865E-30	2	
Ube2v2	4,95139E-34	0,29906	0,82	0,657	8,31388E-30	2	
Ap1s1	4,96328E-34	0,31019	0,773	0,582	8,33385E-30	2	
Dynll1	5,59812E-34	0,25295	0,999	0,988	9,39981E-30	2	
Ppdpf	5,9033E-34	0,28341	0,493	0,302	9,91223E-30	2	
Tcea1	8,88938E-34	0,28663	0,734	0,524	1,49262E-29	2	
Chmp2a	9,18529E-34	0,27839	0,956	0,817	1,5423E-29	2	
Txndc9	9,27773E-34	0,2875	0,687	0,477	1,55782E-29	2	
Cstb	9,58216E-34	0,29333	0,61	0,405	1,60894E-29	2	
Dnajc19	1,19139E-33	0,27503	0,492	0,301	2,00047E-29	2	
Rab4a	1,36082E-33	0,27549	0,572	0,367	2,28496E-29	2	
Ppid	1,57418E-33	0,2783	0,629	0,421	2,64321E-29	2	
Otulin	1,66332E-33	0,28127	0,514	0,32	2,79288E-29	2	
Yaf2	1,84282E-33	0,29446	0,68	0,476	3,09429E-29	2	
Asap1	2,06261E-33	0,30488	0,593	0,398	3,46332E-29	2	
Trnt1	2,36292E-33	0,25635	0,433	0,251	3,96757E-29	2	
Lyrm2	2,71233E-33	0,28707	0,508	0,319	4,55427E-29	2	

Pigyl	2,77017E-33	0,26799	0,439	0,257	4,65139E-29	2	
Fnta	3,14396E-33	0,25164	0,539	0,334	5,27902E-29	2	
Txn2	4,16685E-33	0,29409	0,639	0,439	6,99656E-29	2	
Mrpl35	4,3074E-33	0,29172	0,49	0,303	7,23256E-29	2	
Mast4	4,50337E-33	0,25005	0,471	0,274	7,56161E-29	2	
Gm13305	5,79093E-33	0,39466	0,364	0,202	9,72356E-29	2	
Rbm8a	5,82802E-33	0,29463	0,882	0,726	9,78583E-29	2	
Tmed3	6,07843E-33	0,2596	0,567	0,358	1,02063E-28	2	
Malsu1	6,35932E-33	0,26283	0,426	0,245	1,06779E-28	2	
Csnk2b	6,64354E-33	0,28822	0,773	0,568	1,11552E-28	2	
Echs1	6,91536E-33	0,28619	0,551	0,361	1,16116E-28	2	
Fam174a	7,84937E-33	0,29218	0,678	0,471	1,31799E-28	2	
Nmi	9,31898E-33	0,25685	0,431	0,248	1,56475E-28	2	
Taf10	1,01398E-32	0,28058	0,565	0,366	1,70258E-28	2	
Adrm1	1,12912E-32	0,2842	0,635	0,432	1,8959E-28	2	
Jagn1	1,17072E-32	0,25434	0,43	0,248	1,96575E-28	2	
Sri	1,73616E-32	0,29284	0,667	0,473	2,91519E-28	2	
Slc35b1	1,75251E-32	0,28601	0,584	0,386	2,94265E-28	2	
Mrps14	1,86575E-32	0,28845	0,571	0,378	3,13278E-28	2	
Cystm1	1,90667E-32	0,30026	0,877	0,744	3,20148E-28	2	
Hmgb2	2,8315E-32	0,30037	0,563	0,37	4,75438E-28	2	
Snca	3,13382E-32	0,25931	0,996	0,954	5,26199E-28	2	
Camk1	4,56912E-32	0,30073	0,672	0,483	7,672E-28	2	
Vta1	4,5708E-32	0,28206	0,613	0,416	7,67482E-28	2	
Rsu1	5,03625E-32	0,30619	0,787	0,625	8,45637E-28	2	
Bbip1	5,16336E-32	0,28374	0,633	0,432	8,6698E-28	2	
Psmb8	5,47045E-32	0,31203	0,83	0,649	9,18543E-28	2	
Ifit1	7,98697E-32	0,31272	0,532	0,341	1,34109E-27	2	
Card19	8,40844E-32	0,29813	0,662	0,464	1,41186E-27	2	
Smn1	8,68435E-32	0,28068	0,484	0,303	1,45819E-27	2	
C1qtnf4	1,16765E-31	0,26888	0,401	0,231	1,96059E-27	2	
Wdr43	1,23681E-31	0,30155	0,816	0,644	2,07673E-27	2	
Med19	4,23495E-31	0,25175	0,54	0,347	7,1109E-27	2	
S100a16	4,57357E-31	0,27953	0,978	0,903	7,67948E-27	2	
Ctnna1	5,27447E-31	0,25376	0,715	0,486	8,85636E-27	2	
Cops4	5,42004E-31	0,25462	0,506	0,314	9,10078E-27	2	
Camk2n2	6,55146E-31	0,29288	0,809	0,607	1,10006E-26	2	
Hotairm1	6,74207E-31	0,25741	0,622	0,416	1,13206E-26	2	
Pdia6	6,86344E-31	0,28548	0,695	0,491	1,15244E-26	2	
Immt	7,8455E-31	0,2975	0,703	0,514	1,31734E-26	2	
Tmem242	9,14119E-31	0,26176	0,519	0,332	1,5349E-26	2	
Psmd7	1,31943E-30	0,29006	0,853	0,716	2,21546E-26	2	
Vapa	1,49214E-30	0,27065	0,888	0,751	2,50545E-26	2	
Nr2c2ap	1,98803E-30	0,2506	0,364	0,202	3,3381E-26	2	
Aqr	2,39452E-30	0,28226	0,618	0,427	4,02064E-26	2	
Mitd1	3,35188E-30	0,28062	0,521	0,344	5,62814E-26	2	
Acat1	3,75062E-30	0,29055	0,727	0,537	6,29767E-26	2	
Wdr70	4,2733E-30	0,29703	0,616	0,435	7,1753E-26	2	

Nt5c3b	4,5385E-30	0,25059	0,451	0,272	7,6206E-26	2	
Zfp580	5,53427E-30	0,25803	0,448	0,273	9,2926E-26	2	
Naa10	5,79663E-30	0,25355	0,5	0,318	9,73312E-26	2	
Stard3nl	7,83151E-30	0,29707	0,779	0,606	1,31499E-25	2	
Vbp1	1,39576E-29	0,26876	0,726	0,532	2,34362E-25	2	
Uchl3	2,04161E-29	0,26438	0,5	0,32	3,42806E-25	2	
Ppp1r11	3,15594E-29	0,28589	0,838	0,685	5,29913E-25	2	
Ssu72	3,22979E-29	0,26918	0,869	0,744	5,42314E-25	2	
Eid2	3,29207E-29	0,27954	0,625	0,44	5,52772E-25	2	
Ube2e1	3,97045E-29	0,27531	0,692	0,507	6,66679E-25	2	
Nipsnap1	3,97716E-29	0,25765	0,573	0,383	6,67805E-25	2	
Snapc5	4,46957E-29	0,25295	0,402	0,235	7,50486E-25	2	
Nol12	5,66498E-29	0,27289	0,583	0,404	9,51206E-25	2	
Dynlrb1	6,43746E-29	0,25298	0,992	0,969	1,08091E-24	2	
Zcchc17	6,83102E-29	0,28126	0,697	0,512	1,147E-24	2	
Cnbp	6,97058E-29	0,30488	0,823	0,673	1,17043E-24	2	
Arpp19	8,76446E-29	0,28091	0,917	0,799	1,47164E-24	2	
Cebpz	9,08627E-29	0,28561	0,718	0,535	1,52568E-24	2	
Acyp2	1,5079E-28	0,25006	0,757	0,54	2,53191E-24	2	
Cdk2ap2	1,72073E-28	0,26128	0,635	0,438	2,88928E-24	2	
Wbp4	1,73788E-28	0,271	0,909	0,824	2,91807E-24	2	
Fam32a	1,85659E-28	0,27925	0,725	0,54	3,1174E-24	2	
N6amt1	1,99686E-28	0,25393	0,42	0,253	3,35292E-24	2	
Rbx1	2,27611E-28	0,2514	0,969	0,924	3,82181E-24	2	
Ranbp1	2,32111E-28	0,27753	0,84	0,67	3,89738E-24	2	
Cuta	2,60494E-28	0,25647	0,647	0,445	4,37395E-24	2	
Uqcrc1	3,1273E-28	0,27675	0,836	0,656	5,25105E-24	2	
Dnajc7	3,49601E-28	0,27719	0,756	0,571	5,87015E-24	2	
Dnpep	4,57821E-28	0,26236	0,544	0,362	7,68727E-24	2	
Oas1c	4,74325E-28	0,2635	0,288	0,149	7,9644E-24	2	
Tma7	4,90397E-28	0,25583	0,957	0,878	8,23425E-24	2	
Ube2b	5,70367E-28	0,25477	0,977	0,924	9,57702E-24	2	
Stip1	1,77262E-27	0,25483	0,576	0,393	2,97641E-23	2	
Pfdn6	2,24514E-27	0,25649	0,705	0,507	3,76981E-23	2	
Mrps10	3,87406E-27	0,25541	0,524	0,351	6,50493E-23	2	
Llph	3,93853E-27	0,29157	0,722	0,539	6,61319E-23	2	
Tmx2	4,92901E-27	0,28157	0,706	0,525	8,2763E-23	2	
Snrpd3	5,38359E-27	0,27867	0,764	0,609	9,03959E-23	2	
Mea1	5,67671E-27	0,25315	0,532	0,357	9,53176E-23	2	
Cdc123	6,17649E-27	0,25442	0,604	0,419	1,0371E-22	2	
Idh3b	7,15901E-27	0,27709	0,561	0,389	1,20207E-22	2	
Eif3d	9,92525E-27	0,25348	0,634	0,448	1,66655E-22	2	
Rab18	1,60294E-26	0,27733	0,735	0,571	2,69149E-22	2	
Ube2a	2,17542E-26	0,25251	0,541	0,368	3,65274E-22	2	
Podxl2	2,43987E-26	0,25883	0,79	0,604	4,09679E-22	2	
Smc6	2,78656E-26	0,26432	0,688	0,505	4,67891E-22	2	
Tmem255b	4,4079E-26	0,25622	0,887	0,75	7,40131E-22	2	
Dhrs7	4,89347E-26	0,25469	0,586	0,404	8,21663E-22	2	

Emd	6,31973E-26	0,25227	0,7	0,507	1,06115E-21	2	
Cisd3	7,84483E-26	0,25472	0,572	0,397	1,31723E-21	2	
Sept7	1,01821E-25	0,26072	0,949	0,908	1,70968E-21	2	
Gpatch11	1,46253E-25	0,2523	0,457	0,299	2,45573E-21	2	
Ube2n	2,14757E-25	0,27086	0,759	0,601	3,60598E-21	2	
Mesdc2	2,42293E-25	0,25927	0,683	0,496	4,06835E-21	2	
Higd2a	2,5849E-25	0,26286	0,749	0,594	4,34031E-21	2	
Pdhb	4,24302E-25	0,25586	0,655	0,476	7,12446E-21	2	
Svip	1,20646E-24	0,26316	0,725	0,558	2,02577E-20	2	
Nagk	2,16263E-24	0,26352	0,874	0,747	3,63128E-20	2	
Napg	3,72542E-24	0,25506	0,875	0,754	6,25536E-20	2	
Cfap36	5,29354E-24	0,2546	0,726	0,555	8,88839E-20	2	
Bag1	9,20141E-24	0,25768	0,825	0,683	1,54501E-19	2	
Ap1s2	1,33863E-23	0,26458	0,595	0,429	2,24769E-19	2	
Map7d2	2,52774E-23	0,27144	0,793	0,645	4,24433E-19	2	
Smc3	2,78576E-23	0,25207	0,737	0,574	4,67756E-19	2	
Ncbp2	3,95998E-23	0,25715	0,604	0,442	6,6492E-19	2	
Nub1	4,76414E-23	0,25872	0,705	0,541	7,99947E-19	2	
Msmo1	4,80917E-23	0,27129	0,818	0,686	8,07508E-19	2	
Ehd4	4,81821E-23	0,25224	0,539	0,381	8,09026E-19	2	
Kifap3	5,12602E-23	0,25209	0,912	0,858	8,6071E-19	2	
Hoxb6	8,33066E-23	0,25446	0,496	0,336	1,3988E-18	2	
Atf5	1,23196E-22	0,25706	0,728	0,564	2,06859E-18	2	
Palmd	1,28261E-22	0,29196	0,588	0,423	2,15363E-18	2	
Xrn2	2,88701E-22	0,25517	0,659	0,502	4,84757E-18	2	
Itgb1	4,32521E-22	0,25035	0,981	0,972	7,26245E-18	2	
B2m	3,47734E-18	0,27888	0,787	0,647	5,8388E-14	2	
Oas1d	1,13164E-07	0,32985	0,506	0,426	0,001900135	2	
Sst	0	1,76285	1	0,798	0	3	smENC2b
Slc18a3	7,1869E-265	1,16479	0,933	0,479	1,2068E-260	3	
Dmkn	1,2134E-253	1,11385	0,816	0,298	2,0375E-249	3	
Serping1	1,7447E-230	1,09179	0,812	0,363	2,9296E-226	3	
Ly6e	6,2328E-212	1,03511	0,904	0,458	1,0465E-207	3	
Calcb	4,1564E-199	1,02943	0,979	0,494	6,9791E-195	3	
Gfra2	8,1843E-195	0,86011	0,903	0,406	1,3742E-190	3	
Chgb	2,0248E-189	0,91515	0,961	0,734	3,3999E-185	3	
Fxyd7	2,0636E-188	0,91125	1	0,729	3,4649E-184	3	
Vipr2	1,8543E-180	0,75933	0,678	0,244	3,1135E-176	3	
Smarca2	1,3854E-172	0,7592	0,997	0,941	2,3263E-168	3	
Ache	9,9993E-172	0,74185	0,997	0,96	1,679E-167	3	
Trp53i11	1,2846E-164	0,79404	0,997	0,629	2,157E-160	3	
Pcdh7	2,5726E-159	0,69486	0,809	0,342	4,3197E-155	3	
Krt19	4,3847E-159	0,85204	0,733	0,311	7,3623E-155	3	
Csrp2	6,7126E-159	0,71383	0,796	0,376	1,1271E-154	3	
Cd81	5,0893E-155	0,63608	1	0,991	8,5454E-151	3	
Ly6h	7,3436E-147	0,75948	0,829	0,459	1,2331E-142	3	
Slc10a4	3,3821E-143	0,72884	0,973	0,79	5,6788E-139	3	
Tshz2	8,7791E-141	0,73898	0,993	0,833	1,4741E-136	3	

Nrp2	3,1067E-139	0,64309	0,653	0,259	5,2164E-135	3	
Sphkap	5,1452E-132	0,60919	0,622	0,242	8,6393E-128	3	
Rab3b	3,0387E-129	0,65755	0,811	0,423	5,1023E-125	3	
Sparc	2,4297E-127	0,74735	0,877	0,592	4,0797E-123	3	
Fam19a5	8,8752E-127	0,63172	0,763	0,36	1,4902E-122	3	
Pla2g7	2,7373E-124	0,55475	0,494	0,171	4,5963E-120	3	
Ifitm2	2,9108E-124	0,62889	1	0,947	4,8875E-120	3	
Nell1	3,1812E-122	0,59864	0,64	0,264	5,3416E-118	3	
Ddah1	5,3124E-117	0,60584	0,762	0,385	8,9201E-113	3	
Nisch	1,292E-116	0,48952	1	0,988	2,1695E-112	3	
Rspo2	5,3319E-115	0,52075	0,563	0,218	8,9527E-111	3	
Piezo1	3,4779E-114	0,47521	0,517	0,191	5,8398E-110	3	
Wbscr17	4,0335E-113	0,49853	0,525	0,2	6,7727E-109	3	
Wscd1	7,1675E-111	0,50725	0,385	0,117	1,2035E-106	3	
Prph	5,3854E-109	0,48416	1	0,969	9,0426E-105	3	
Bcl2	1,0565E-107	0,55619	0,798	0,433	1,7739E-103	3	
Kif22	1,805E-105	0,68588	0,932	0,722	3,0308E-101	3	
Rasd2	4,2335E-103	0,52352	0,556	0,234	7,1085E-99	3	
Pth1r	3,4819E-102	0,50287	0,52	0,206	5,84646E-98	3	
Adamts9	5,0394E-102	0,47162	0,616	0,261	8,46167E-98	3	
Galnt10	1,4783E-101	0,39868	0,373	0,117	2,48213E-97	3	
Aqp1	8,1018E-101	0,53232	0,484	0,191	1,36037E-96	3	
Cct7	1,2113E-97	0,55069	0,991	0,924	2,03389E-93	3	
Tpi1	4,81695E-97	0,64111	0,954	0,875	8,08814E-93	3	
S100a1	6,04884E-97	0,57338	0,962	0,818	1,01566E-92	3	
Map1lc3a	8,74436E-91	0,44993	0,999	0,979	1,46826E-86	3	
Samd14	6,09049E-90	0,56381	0,889	0,707	1,02265E-85	3	
Avpr1a	1,55036E-88	0,46942	0,538	0,232	2,60321E-84	3	
Scube1	1,21707E-87	0,48253	0,995	0,898	2,04359E-83	3	
Ahi1	1,13319E-86	0,42051	1	1	1,90274E-82	3	
Rpl36	8,89389E-86	0,47818	0,998	0,941	1,49337E-81	3	
Emb	3,26973E-85	0,45001	0,486	0,207	5,49021E-81	3	
Rtp4	7,69346E-85	0,41957	0,446	0,178	1,29181E-80	3	
Rpl31	1,02009E-84	0,5004	0,978	0,871	1,71284E-80	3	
Itga6	1,37593E-83	0,50419	0,768	0,456	2,31032E-79	3	
Higd1a	2,37985E-83	0,39626	0,496	0,208	3,996E-79	3	
Brinp1	1,66874E-82	0,50561	0,611	0,326	2,80199E-78	3	
Ap2a2	2,32942E-82	0,42677	0,997	0,978	3,91133E-78	3	
Lrrtm1	4,43199E-82	0,47095	0,573	0,276	7,44175E-78	3	
Rpl6	8,24837E-81	0,55304	1	0,97	1,38498E-76	3	
Lynx1	5,35578E-80	0,40709	0,547	0,251	8,99289E-76	3	
Atp1b1	3,7647E-79	0,4582	0,989	0,857	6,3213E-75	3	
Igfbp5	4,56818E-78	0,3635	0,481	0,195	7,67043E-74	3	
Ass1	5,71194E-78	0,5308	0,774	0,502	9,59091E-74	3	
Tceb2	2,56854E-77	0,44275	0,999	0,954	4,31284E-73	3	
Bnc2	3,5708E-77	0,36091	0,495	0,21	5,99572E-73	3	
Tcf7l2	3,73897E-77	0,41478	0,981	0,828	6,27811E-73	3	
Mcam	9,0295E-77	0,43237	0,922	0,646	1,51614E-72	3	

Rplp1	1,30725E-75	0,42069	1	0,977	2,195E-71	3	
Hoxa5	8,61733E-75	0,37208	0,99	0,936	1,44694E-70	3	
Ncl	1,08539E-74	0,47447	0,998	0,96	1,82247E-70	3	
Tcaf1	1,17533E-74	0,45763	0,98	0,919	1,97349E-70	3	
Cnih3	1,86827E-74	0,36535	0,383	0,146	3,13701E-70	3	
Sez6l	1,89452E-74	0,54796	0,743	0,494	3,18108E-70	3	
Rprm	2,19263E-74	0,4476	0,54	0,26	3,68164E-70	3	
Spock2	2,57024E-74	0,39991	0,999	0,977	4,31568E-70	3	
Gcgr	1,51493E-72	0,47414	0,564	0,297	2,54372E-68	3	
Rpl8	1,436E-71	0,42533	1	0,968	2,41118E-67	3	
Parm1	1,7338E-71	0,49244	0,963	0,889	2,91122E-67	3	
Ntm	5,17246E-71	0,48759	0,362	0,136	8,68508E-67	3	
Ifi27	6,6294E-71	0,4332	0,967	0,775	1,11314E-66	3	
Prima1	1,04388E-70	0,39442	0,372	0,146	1,75278E-66	3	
Rpl38	1,93754E-70	0,49582	1	0,994	3,25332E-66	3	
Nap1l1	3,20462E-70	0,48783	0,887	0,717	5,38088E-66	3	
Rhoc	4,21007E-69	0,47864	0,642	0,381	7,06914E-65	3	
Atp5k	3,23537E-68	0,49145	1	0,955	5,43251E-64	3	
Caln1	4,7343E-68	0,33078	0,425	0,177	7,94937E-64	3	
P2rx2	7,43623E-68	0,4422	0,999	0,982	1,24862E-63	3	
Gaa	7,84281E-68	0,48676	0,949	0,845	1,31689E-63	3	
Pcsk1n	1,53434E-67	0,30672	1	1	2,57631E-63	3	
Tle3	7,48876E-67	0,3685	0,437	0,195	1,25744E-62	3	
Nmbr	1,0231E-65	0,27603	0,279	0,092	1,71789E-61	3	
Bche	1,03077E-65	0,39628	0,96	0,775	1,73077E-61	3	
Rpl37	3,22438E-65	0,44074	1	0,985	5,41406E-61	3	
Eef2	2,36843E-64	0,41779	0,993	0,946	3,97683E-60	3	
St3gal6	3,60072E-64	0,3725	0,469	0,221	6,04598E-60	3	
Nbl1	1,41479E-63	0,49196	0,766	0,554	2,37558E-59	3	
Fat4	2,63705E-63	0,3242	0,359	0,142	4,42788E-59	3	
Hmgb1	3,40213E-63	0,38727	0,999	0,974	5,71252E-59	3	
Mt1	1,32326E-62	0,36565	0,372	0,156	2,22188E-58	3	
Timp3	1,37826E-62	0,37017	0,775	0,44	2,31424E-58	3	
Ptprt	2,2797E-62	0,37915	0,407	0,177	3,82784E-58	3	
Gria1	2,76528E-62	0,30568	0,272	0,092	4,64318E-58	3	
Tox	3,08182E-62	0,33977	0,392	0,166	5,17468E-58	3	
Atp1a1	1,84952E-61	0,32597	1	0,997	3,10553E-57	3	
Nrxn3	3,00804E-61	0,26764	0,77	0,423	5,05081E-57	3	
Ctnna1	3,17288E-61	0,47224	0,718	0,483	5,32759E-57	3	
Mrpl52	4,09762E-61	0,43932	0,915	0,758	6,88032E-57	3	
Tmem59l	3,77154E-60	0,45689	0,611	0,371	6,3328E-56	3	
Ednrb	5,26639E-60	0,32613	0,354	0,144	8,8428E-56	3	
Ryr2	6,40966E-60	0,3567	0,524	0,266	1,07625E-55	3	
Gtf2i	1,34989E-58	0,44089	0,887	0,716	2,2666E-54	3	
Dio2	1,55586E-58	0,3284	0,32	0,124	2,61245E-54	3	
Kndc1	5,23065E-57	0,36286	0,486	0,25	8,78279E-53	3	
Psme1	7,22703E-57	0,39336	0,979	0,837	1,21349E-52	3	
Tmem229b	1,45197E-56	0,44154	0,752	0,526	2,438E-52	3	

Flot2	2,17601E-56	0,36835	0,977	0,918	3,65375E-52	3	
Sncb	2,58958E-56	0,3444	0,452	0,22	4,34817E-52	3	
Prmt8	5,03245E-56	0,26557	0,323	0,126	8,44999E-52	3	
B3gnt2	5,98797E-56	0,38126	0,507	0,272	1,00544E-51	3	
Psmb10	8,68671E-56	0,42318	0,863	0,661	1,45859E-51	3	
Rab11fip4	1,03198E-55	0,39672	0,49	0,266	1,7328E-51	3	
Grina	1,65741E-55	0,39297	0,975	0,916	2,78295E-51	3	
Gpsm3	3,53469E-55	0,3603	0,428	0,202	5,93509E-51	3	
Casz1	9,40299E-55	0,26819	0,66	0,348	1,57886E-50	3	
Ppp2r1a	1,99128E-54	0,35987	0,995	0,951	3,34356E-50	3	
Ckmt1	2,02114E-54	0,43934	0,718	0,501	3,39369E-50	3	
Cacna1e	1,17335E-53	0,35411	0,735	0,482	1,97018E-49	3	
Hdac11	2,63194E-53	0,44708	0,636	0,426	4,41929E-49	3	
Faim2	1,24916E-52	0,43606	0,617	0,389	2,09746E-48	3	
Clstn1	1,35829E-52	0,42198	0,94	0,854	2,2807E-48	3	
Slco2a1	3,11819E-52	0,30319	0,303	0,121	5,23575E-48	3	
Atp6v1e1	7,79857E-52	0,33762	0,999	0,956	1,30946E-47	3	
Dusp26	9,82764E-52	0,46196	0,784	0,641	1,65016E-47	3	
Sod1	2,66536E-51	0,37966	0,979	0,921	4,4754E-47	3	
Mxra7	3,79868E-51	0,39304	0,612	0,376	6,37836E-47	3	
Atp9a	4,51825E-51	0,36003	0,98	0,912	7,5866E-47	3	
Plce1	7,65752E-51	0,43157	0,67	0,456	1,28577E-46	3	
Smg6	7,83477E-51	0,42923	0,695	0,496	1,31554E-46	3	
Rps15	7,98291E-51	0,34406	0,995	0,941	1,34041E-46	3	
Pdap1	1,62463E-50	0,36462	0,994	0,934	2,72791E-46	3	
Slc22a17	1,93317E-50	0,38058	0,974	0,926	3,24599E-46	3	
Rps17	5,61478E-50	0,34042	0,992	0,905	9,42777E-46	3	
Calr	1,21357E-49	0,37443	0,98	0,937	2,0377E-45	3	
Bend5	3,70227E-49	0,41398	0,624	0,406	6,21648E-45	3	
Chrm2	6,17665E-49	0,27105	0,312	0,131	1,03712E-44	3	
Begain	6,73306E-49	0,38394	0,729	0,511	1,13055E-44	3	
Kcnc4	7,53E-49	0,32497	0,476	0,251	1,26436E-44	3	
Ndufa13	1,15544E-48	0,33967	0,96	0,79	1,94009E-44	3	
Dek	1,28036E-48	0,39869	0,794	0,61	2,14985E-44	3	
Gsg1l	1,42566E-48	0,2845	0,305	0,127	2,39383E-44	3	
Zc3h15	5,82932E-48	0,33793	0,975	0,898	9,78801E-44	3	
Kif21a	7,89939E-48	0,3361	0,989	0,974	1,32639E-43	3	
Ccser2	9,99056E-48	0,32129	0,933	0,806	1,67752E-43	3	
Akr1b3	1,17551E-47	0,3944	0,856	0,711	1,9738E-43	3	
Ndufa5	1,97715E-47	0,36433	0,951	0,793	3,31984E-43	3	
Sez6	2,35933E-47	0,38248	0,565	0,346	3,96155E-43	3	
Scarb2	2,72552E-47	0,39026	0,688	0,481	4,57641E-43	3	
Sema5a	2,86921E-47	0,25354	0,31	0,131	4,81769E-43	3	
Ptov1	3,51939E-47	0,33485	0,981	0,937	5,9094E-43	3	
Dpysl5	3,59075E-47	0,3739	0,718	0,503	6,02922E-43	3	
Rps29	4,19596E-47	0,40814	1	0,996	7,04543E-43	3	
Susd2	1,84398E-46	0,38993	0,451	0,246	3,09622E-42	3	
Atp5a1	4,58356E-46	0,38525	0,943	0,871	7,69626E-42	3	

Rpl21	6,79862E-46	0,3188	0,999	0,965	1,14156E-41	3	
Pkm	8,75798E-46	0,36533	0,991	0,952	1,47055E-41	3	
Ctgf	1,20432E-45	0,30393	0,378	0,182	2,02217E-41	3	
Cspg4	3,24298E-45	0,28566	0,282	0,119	5,44529E-41	3	
Rpl26	2,35571E-44	0,31055	0,996	0,948	3,95547E-40	3	
Gars	2,52716E-44	0,38404	0,802	0,639	4,24336E-40	3	
Pak3	7,01058E-44	0,2922	0,89	0,689	1,17715E-39	3	
Gdf10	7,86938E-44	0,28633	0,341	0,158	1,32135E-39	3	
Prkcdbp	1,03425E-43	0,3487	0,531	0,319	1,73661E-39	3	
Sez6l2	1,10317E-43	0,34938	0,903	0,795	1,85233E-39	3	
Rpl18a	1,53799E-42	0,30475	0,999	0,975	2,58243E-38	3	
Rpl13	1,99571E-42	0,2992	0,999	0,953	3,35099E-38	3	
Slit3	2,22656E-42	0,34479	0,584	0,369	3,73861E-38	3	
Dynlrb1	2,24853E-42	0,30197	0,993	0,969	3,77551E-38	3	
Vcp	4,09171E-42	0,27603	0,999	0,993	6,87038E-38	3	
Hspa8	6,09333E-42	0,36869	1	0,994	1,02313E-37	3	
Atp5e	6,65855E-42	0,30995	0,984	0,829	1,11804E-37	3	
Nfix	1,22923E-41	0,29764	0,919	0,741	2,06401E-37	3	
Dynll2	6,24108E-41	0,28118	0,996	0,972	1,04794E-36	3	
Cyth3	1,38203E-40	0,28669	1	0,995	2,32057E-36	3	
Rpl32	3,67767E-40	0,28559	1	0,964	6,17518E-36	3	
Tspan8	6,84441E-40	0,30613	0,27	0,117	1,14924E-35	3	
Skp1a	8,66068E-40	0,3146	0,962	0,908	1,45421E-35	3	
Eif4a2	2,68782E-39	0,28036	0,997	0,979	4,51312E-35	3	
Epha5	2,91198E-39	0,33711	0,395	0,209	4,8895E-35	3	
Filip1	3,74703E-39	0,35554	0,912	0,81	6,29164E-35	3	
Dnm1	6,40101E-39	0,35331	0,784	0,621	1,07479E-34	3	
Dip2b	9,42148E-39	0,36791	0,74	0,573	1,58196E-34	3	
Vwc2	1,70023E-38	0,29834	0,435	0,239	2,85486E-34	3	
Rpl24	1,88338E-38	0,2842	0,999	0,941	3,16239E-34	3	
Rgs9	2,40941E-38	0,3435	0,865	0,744	4,04563E-34	3	
Calb2	2,70542E-38	0,26029	1	0,977	4,54268E-34	3	
S100a6	5,60571E-38	0,27855	1	0,999	9,41254E-34	3	
Tmem132c	1,36449E-37	0,25469	0,344	0,17	2,29112E-33	3	
Pid1	1,40667E-37	0,32446	0,694	0,503	2,36194E-33	3	
Rps11	1,94238E-37	0,27804	0,998	0,931	3,26145E-33	3	
Rrp1	2,34273E-37	0,32696	0,916	0,809	3,93368E-33	3	
Eno2	2,35607E-37	0,35562	0,895	0,83	3,95607E-33	3	
Rpl28	2,50947E-37	0,28915	0,998	0,962	4,21365E-33	3	
Ap2a1	4,95345E-37	0,33712	0,836	0,709	8,31733E-33	3	
Atf5	7,16173E-37	0,40799	0,709	0,566	1,20253E-32	3	
Uqcc2	1,25245E-36	0,33948	0,861	0,708	2,103E-32	3	
Ywhae	2,41629E-36	0,25043	1	0,996	4,05719E-32	3	
Sgip1	3,00337E-36	0,3156	0,912	0,841	5,04296E-32	3	
Eif4g1	1,08901E-35	0,30602	0,923	0,85	1,82856E-31	3	
Tecr	1,2211E-35	0,27808	0,997	0,972	2,05036E-31	3	
Rpl18	1,38668E-35	0,26755	0,984	0,856	2,32838E-31	3	
Got2	2,06877E-35	0,34372	0,85	0,751	3,47367E-31	3	

Myh10	2,29383E-35	0,33681	0,731	0,551	3,85157E-31	3	
Rpl36al	2,56572E-35	0,29065	0,959	0,828	4,30811E-31	3	
Ndufs6	3,25035E-35	0,32092	0,817	0,641	5,45767E-31	3	
Cpe	3,26425E-35	0,30088	0,971	0,936	5,481E-31	3	
Uqcr10	4,07161E-35	0,27286	0,927	0,723	6,83664E-31	3	
Chd3	7,34452E-35	0,25823	0,993	0,949	1,23322E-30	3	
Psmb6	1,01196E-34	0,29104	0,922	0,758	1,69918E-30	3	
Ifitm3	1,05356E-34	0,42872	0,474	0,293	1,76903E-30	3	
Por	1,19636E-34	0,33781	0,727	0,577	2,0088E-30	3	
Raly	1,2335E-34	0,35409	0,743	0,592	2,07116E-30	3	
Slc5a7	1,6506E-34	0,27907	0,797	0,604	2,77152E-30	3	
Ndufa11	2,90748E-34	0,29512	0,966	0,882	4,88195E-30	3	
Uqcrq	5,89199E-34	0,27114	0,982	0,85	9,89323E-30	3	
Cdh2	6,00078E-34	0,28122	0,971	0,923	1,00759E-29	3	
Arhgap20	7,20462E-34	0,30277	0,471	0,287	1,20973E-29	3	
Aldoa	8,56852E-34	0,25257	1	0,996	1,43874E-29	3	
Ptprs	9,23463E-34	0,2669	0,897	0,751	1,55059E-29	3	
Rpl27a	1,39709E-33	0,25429	0,999	0,961	2,34586E-29	3	
Cplx1	2,01499E-33	0,31738	0,509	0,333	3,38337E-29	3	
Nfib	2,12652E-33	0,26372	0,961	0,873	3,57064E-29	3	
Metap2	3,67769E-33	0,2771	0,992	0,968	6,17521E-29	3	
Nlrp6	5,86049E-33	0,28774	0,365	0,203	9,84035E-29	3	
Psmd13	6,38776E-33	0,34199	0,784	0,641	1,07257E-28	3	
Ubb	7,62004E-33	0,26996	1	0,986	1,27948E-28	3	
Hmgb2	7,96613E-33	0,35275	0,541	0,371	1,33759E-28	3	
Fam163a	9,07877E-33	0,32666	0,749	0,596	1,52442E-28	3	
Pdlim2	1,03891E-32	0,28564	0,471	0,279	1,74444E-28	3	
Rps25	1,3394E-32	0,27625	0,96	0,847	2,24899E-28	3	
Tkt	2,32689E-32	0,30678	0,885	0,768	3,90709E-28	3	
Atp5b	9,11618E-32	0,3122	0,976	0,916	1,5307E-27	3	
Arhgdig	1,02029E-31	0,34314	0,781	0,66	1,71317E-27	3	
Eri3	1,34801E-31	0,3182	0,661	0,504	2,26344E-27	3	
Tln2	1,9513E-31	0,26092	0,904	0,801	3,27642E-27	3	
Uqcrc1	2,39771E-31	0,32215	0,802	0,659	4,026E-27	3	
Parvb	2,54005E-31	0,3102	0,751	0,59	4,265E-27	3	
Pfkm	3,3565E-31	0,31744	0,534	0,361	5,6359E-27	3	
Tmem5	3,3794E-31	0,31684	0,702	0,544	5,67435E-27	3	
Aco2	6,08196E-31	0,3478	0,795	0,682	1,02122E-26	3	
Maz	1,08667E-30	0,26693	0,954	0,862	1,82462E-26	3	
Ddx1	1,18749E-30	0,28824	0,813	0,629	1,99392E-26	3	
Pebp1	1,34545E-30	0,26952	0,994	0,958	2,25915E-26	3	
Kcnb1	2,36741E-30	0,2537	0,716	0,534	3,97513E-26	3	
Myrip	2,56555E-30	0,27186	0,613	0,43	4,30781E-26	3	
Tmsb10	2,99331E-30	0,25164	0,998	0,949	5,02607E-26	3	
Adamts5	3,58211E-30	0,29272	0,438	0,261	6,01471E-26	3	
Nrcam	4,46963E-30	0,26576	0,417	0,249	7,50496E-26	3	
Ndufb7	4,9447E-30	0,27062	0,905	0,767	8,30264E-26	3	
Ccl27a	6,66905E-30	0,29654	0,581	0,409	1,1198E-25	3	

Caly	8,72493E-30	0,31766	0,876	0,748	1,465E-25	3	
Rph3a	1,06158E-29	0,26225	0,817	0,636	1,7825E-25	3	
Atp5j	1,36531E-29	0,26777	0,936	0,822	2,29249E-25	3	
Znhit1	2,48699E-29	0,29545	0,856	0,71	4,1759E-25	3	
Dync1i1	4,27783E-29	0,33772	0,694	0,541	7,1829E-25	3	
Psap	5,26711E-29	0,25533	0,999	0,999	8,844E-25	3	
Arrb1	6,67817E-29	0,32432	0,605	0,458	1,12133E-24	3	
St13	7,25486E-29	0,28246	0,957	0,916	1,21816E-24	3	
Svil	7,31031E-29	0,28256	0,673	0,502	1,22747E-24	3	
Pcdh17	1,19058E-28	0,30508	0,908	0,838	1,9991E-24	3	
Adgrb2	1,20513E-28	0,28369	0,482	0,319	2,02353E-24	3	
Otub1	2,56429E-28	0,31841	0,711	0,587	4,30569E-24	3	
Acadl	2,7977E-28	0,29115	0,855	0,755	4,69762E-24	3	
Ndufa2	4,84659E-28	0,26293	0,972	0,878	8,1379E-24	3	
Eml6	6,32247E-28	0,27774	0,694	0,532	1,06161E-23	3	
Dbn1	7,56975E-28	0,29717	0,752	0,622	1,27104E-23	3	
Podxl2	1,89182E-27	0,30396	0,737	0,609	3,17656E-23	3	
Atp6v0e2	4,7272E-27	0,29034	0,918	0,874	7,93744E-23	3	
Cadm3	5,02083E-27	0,25808	0,862	0,752	8,43047E-23	3	
Eef1g	5,38929E-27	0,29843	0,926	0,86	9,04916E-23	3	
Nt5dc2	1,12361E-26	0,26535	0,444	0,285	1,88665E-22	3	
Pomp	1,18737E-26	0,26931	0,882	0,758	1,99372E-22	3	
Fdft1	1,41468E-26	0,29182	0,853	0,736	2,37538E-22	3	
Mapk8ip1	2,97039E-26	0,31115	0,753	0,654	4,98759E-22	3	
Gabarapl1	3,29616E-26	0,25229	0,984	0,958	5,53458E-22	3	
Mdh2	3,30001E-26	0,26588	0,857	0,71	5,54104E-22	3	
Ddb1	3,45536E-26	0,34065	0,798	0,723	5,80189E-22	3	
Abcf1	7,10583E-26	0,2778	0,734	0,584	1,19314E-21	3	
Scrn1	7,46458E-26	0,25471	0,383	0,235	1,25338E-21	3	
Pdcd5	8,22279E-26	0,28051	0,874	0,778	1,38069E-21	3	
Plppr3	9,51426E-26	0,2906	0,616	0,469	1,59754E-21	3	
Eif3c	2,60376E-25	0,27487	0,899	0,824	4,37198E-21	3	
Tmem179	3,45273E-25	0,29083	0,871	0,803	5,79749E-21	3	
Rangap1	3,48276E-25	0,29098	0,734	0,614	5,8479E-21	3	
Eif3e	7,01423E-25	0,26233	0,72	0,55	1,17776E-20	3	
Il11ra1	9,22383E-25	0,29942	0,66	0,515	1,54877E-20	3	
1810041L15I	9,64994E-25	0,25256	0,854	0,74	1,62032E-20	3	
Gpc6	1,01185E-24	0,26694	0,649	0,494	1,699E-20	3	
Ctsd	1,17234E-24	0,26611	0,911	0,842	1,96848E-20	3	
Ifit1	1,4656E-24	0,32527	0,493	0,345	2,46089E-20	3	
Ddx24	1,62573E-24	0,27468	0,872	0,78	2,72977E-20	3	
Adgre1	4,90382E-24	0,26264	0,623	0,457	8,23401E-20	3	
Sf3b2	2,63127E-23	0,25172	0,912	0,858	4,41817E-19	3	
Ndufv1	3,48755E-23	0,263	0,748	0,612	5,85594E-19	3	
Gltscr2	2,69314E-22	0,27845	0,647	0,517	4,52205E-18	3	
Tceal5	3,97475E-22	0,27027	0,615	0,465	6,674E-18	3	
Fasn	4,98609E-22	0,28005	0,509	0,375	8,37214E-18	3	
Slc27a4	1,99221E-21	0,26633	0,526	0,394	3,34512E-17	3	

Ppp2r4	2,63525E-21	0,25013	0,861	0,801	4,42485E-17	3	
Tekt2	3,11071E-21	0,27252	0,632	0,496	5,2232E-17	3	
Nub1	4,63461E-21	0,26132	0,668	0,545	7,78197E-17	3	
Ufsp1	9,25281E-21	0,26113	0,473	0,339	1,55364E-16	3	
Nudc	1,04635E-20	0,253	0,793	0,698	1,75692E-16	3	
Ppp5c	1,38166E-20	0,27743	0,643	0,521	2,31994E-16	3	
Rsph9	1,92094E-20	0,26146	0,488	0,357	3,22546E-16	3	
Svip	2,02257E-20	0,25451	0,692	0,561	3,3961E-16	3	
6330403K07	2,78001E-20	0,25534	0,965	0,936	4,66792E-16	3	
AY036118	3,37375E-20	0,25288	0,918	0,844	5,66486E-16	3	
Gnaz	7,83377E-20	0,25238	0,734	0,621	1,31537E-15	3	
Tbrg1	2,15977E-19	0,25753	0,613	0,481	3,62646E-15	3	
Nrbp1	2,18798E-19	0,25481	0,691	0,579	3,67384E-15	3	
Kpna3	1,60678E-18	0,25004	0,685	0,566	2,69794E-14	3	
Ndrg3	3,24191E-18	0,25271	0,676	0,577	5,44349E-14	3	
Trp53inp2	9,57587E-16	0,26578	0,614	0,528	1,60788E-11	3	
Gm42418	0	1,66103	1	1	0	4	smENC2c
Trp53i11	0	1,29058	0,998	0,623	0	4	
Mcam	7,67E-291	1,20386	0,97	0,636	1,2879E-286	4	
Hoxa5	1,9615E-263	0,96045	0,998	0,934	3,2936E-259	4	
Timp3	3,3846E-232	1,08026	0,884	0,419	5,6831E-228	4	
Ly6e	3,3486E-229	1,17758	0,871	0,456	5,6227E-225	4	
Scube1	4,553E-228	0,90128	1	0,896	7,6449E-224	4	
Pcsk1n	3,7106E-220	0,63133	1	1	6,2304E-216	4	
Sst	1,1314E-219	1,25193	1	0,795	1,8998E-215	4	
Prnp	4,2978E-219	0,8325	0,999	0,976	7,2165E-215	4	
Fxyd7	7,9282E-214	0,96815	0,999	0,725	1,3312E-209	4	
Nfix	1,7391E-210	0,87363	0,98	0,729	2,9201E-206	4	
Igfbp5	5,3395E-206	0,79744	0,585	0,176	8,9656E-202	4	
Smarca2	4,8879E-195	0,78027	0,999	0,94	8,2073E-191	4	
Bche	4,3138E-189	0,87677	0,985	0,768	7,2433E-185	4	
Tshz2	7,7627E-185	0,81249	0,993	0,83	1,3034E-180	4	
Ebf3	1,678E-181	0,58603	0,456	0,116	2,8175E-177	4	
Sphkap	3,1236E-177	0,78026	0,636	0,234	5,2449E-173	4	
Rspo2	1,2028E-174	0,71266	0,603	0,207	2,0196E-170	4	
Vamp1	8,7399E-174	0,85273	0,963	0,788	1,4675E-169	4	
Apba2	8,26E-168	0,79195	0,933	0,691	1,3869E-163	4	
Vat1l	1,9766E-167	0,786	0,975	0,821	3,319E-163	4	
Pcdh7	3,9916E-165	0,71332	0,782	0,339	6,7023E-161	4	
Camk2b	2,3138E-164	0,77326	0,927	0,708	3,8851E-160	4	
Lars2	3,903E-161	0,77514	0,999	0,988	6,5535E-157	4	
Gfra2	2,523E-160	0,71616	0,873	0,403	4,2363E-156	4	
Fam19a5	2,5361E-160	0,7745	0,757	0,355	4,2584E-156	4	
Hoxb5	2,6334E-160	0,66649	0,999	0,953	4,4218E-156	4	
Atp1b1	3,0597E-150	0,69344	0,991	0,855	5,1375E-146	4	
Slc10a4	3,869E-150	0,74431	0,965	0,788	6,4965E-146	4	
Sez6l	4,0566E-147	0,82937	0,79	0,483	6,8114E-143	4	
Dst	4,715E-147	0,52572	1	0,997	7,9169E-143	4	

Rph3a	5,8917E-144	0,77508	0,887	0,623	9,8927E-140	4	
Bnc2	2,3918E-139	0,52846	0,555	0,197	4,016E-135	4	
Casz1	5,1092E-134	0,53392	0,753	0,33	8,5788E-130	4	
Nrxn2	2,71E-133	0,5722	0,996	0,958	4,5504E-129	4	
Tox	4,3218E-131	0,50674	0,463	0,153	7,2568E-127	4	
Ina	2,0721E-128	0,66807	0,91	0,728	3,4792E-124	4	
Chd3	7,4321E-124	0,5484	0,996	0,948	1,2479E-119	4	
Calcb	4,7658E-123	0,58285	0,95	0,492	8,0023E-119	4	
Vipr2	4,5607E-121	0,54157	0,607	0,248	7,6579E-117	4	
Gsg1l	4,7351E-120	0,52483	0,378	0,114	7,9508E-116	4	
Satb2	9,2923E-120	0,64147	0,691	0,374	1,5603E-115	4	
Smarcc2	2,6646E-119	0,61241	0,901	0,706	4,4742E-115	4	
Piezo1	1,6078E-117	0,49884	0,502	0,188	2,6996E-113	4	
Nrp2	5,1816E-117	0,56817	0,606	0,261	8,7004E-113	4	
Snap25	2,4306E-115	0,46625	1	0,999	4,0812E-111	4	
Spock2	1,6046E-114	0,50139	0,996	0,977	2,6943E-110	4	
Itga6	2,3095E-113	0,64079	0,77	0,451	3,8779E-109	4	
Fat4	5,1723E-113	0,42047	0,407	0,132	8,6848E-109	4	
Slco2a1	9,9536E-112	0,4382	0,364	0,109	1,6713E-107	4	
Tcf7l2	4,7604E-111	0,48539	0,989	0,824	7,9932E-107	4	
Atxn1	1,4727E-107	0,48764	0,481	0,189	2,4728E-103	4	
Ctbp1	1,1788E-106	0,52071	0,967	0,867	1,9793E-102	4	
Wbscr17	1,5493E-105	0,4902	0,497	0,199	2,6015E-101	4	
Enah	1,3266E-104	0,49347	0,978	0,877	2,2275E-100	4	
Pak3	1,9503E-104	0,55672	0,897	0,685	3,2747E-100	4	
Slc7a14	3,2695E-103	0,56598	0,955	0,826	5,4899E-99	4	
Sulf2	1,4991E-102	0,43911	0,552	0,229	2,51721E-98	4	
Slc36a1	3,0791E-102	0,51464	1	0,959	5,17012E-98	4	
Ntm	3,5071E-102	0,55578	0,389	0,129	5,88871E-98	4	
Ppp2r2c	1,2986E-101	0,55691	0,938	0,789	2,18042E-97	4	
Caln1	3,6179E-101	0,41378	0,457	0,169	6,07477E-97	4	
6330403A02	1,08282E-98	0,33261	0,996	0,93	1,81817E-94	4	
Dmkn	1,22794E-94	0,53597	0,664	0,313	2,06183E-90	4	
Adamts9	1,22819E-94	0,46921	0,576	0,262	2,06226E-90	4	
Ache	9,74755E-94	0,50946	0,994	0,96	1,63671E-89	4	
App	1,40441E-93	0,42079	0,999	0,994	2,35814E-89	4	
Slc5a7	3,37216E-93	0,57117	0,836	0,596	5,66219E-89	4	
Zfp503	4,69131E-93	0,38913	0,438	0,168	7,87717E-89	4	
Gria1	1,38615E-92	0,35369	0,297	0,086	2,32749E-88	4	
Sel1l3	3,63276E-92	0,56545	0,81	0,585	6,09976E-88	4	
L1cam	4,28043E-92	0,42164	0,999	0,979	7,18727E-88	4	
Serping1	2,00793E-91	0,60094	0,678	0,376	3,37152E-87	4	
Adamtsl1	1,69768E-90	0,30699	0,28	0,078	2,85058E-86	4	
Maz	1,01764E-89	0,49466	0,963	0,859	1,70871E-85	4	
Zcchc12	2,67254E-89	0,55901	0,972	0,876	4,48747E-85	4	
Flywch1	6,54596E-89	0,51331	0,913	0,783	1,09913E-84	4	
Avpr1a	2,95758E-88	0,39884	0,534	0,228	4,96608E-84	4	
Abca2	3,35893E-87	0,55898	0,793	0,579	5,63998E-83	4	

Ctnna1	6,48167E-87	0,58074	0,729	0,478	1,08834E-82	4	
Tmem28	3,0016E-86	0,31117	0,286	0,084	5,03998E-82	4	
Pth1r	1,03398E-83	0,47236	0,472	0,209	1,73615E-79	4	
Dio2	2,01608E-83	0,37357	0,342	0,118	3,3852E-79	4	
Dlgap3	2,93215E-82	0,54852	0,677	0,427	4,92338E-78	4	
Nrxn3	2,97183E-82	0,41044	0,761	0,42	4,98999E-78	4	
Ryr2	6,57529E-82	0,43718	0,538	0,26	1,10406E-77	4	
Tmem63b	8,00831E-82	0,46211	0,901	0,738	1,34467E-77	4	
Bcl2	8,229E-82	0,47632	0,741	0,436	1,38173E-77	4	
Gtf2i	1,34863E-81	0,51192	0,877	0,716	2,26448E-77	4	
Spata13	2,96604E-81	0,45307	0,501	0,236	4,98029E-77	4	
Tmem229b	1,46118E-80	0,57516	0,754	0,522	2,45346E-76	4	
Adamts5	6,36253E-77	0,47763	0,511	0,248	1,06833E-72	4	
Ctgf	9,83052E-77	0,46946	0,41	0,175	1,65064E-72	4	
Adcy1	1,68069E-76	0,44287	0,576	0,3	2,82205E-72	4	
Kcnc4	1,75368E-74	0,4463	0,497	0,245	2,9446E-70	4	
Ppp2r1a	3,2546E-73	0,39407	0,994	0,95	5,4648E-69	4	
Flot2	5,0225E-73	0,43342	0,975	0,917	8,43327E-69	4	
Tmem132c	9,31996E-73	0,37769	0,39	0,161	1,56491E-68	4	
Ppm1h	2,4693E-72	0,46473	0,847	0,683	4,14621E-68	4	
Prmt8	3,00749E-72	0,33471	0,331	0,122	5,04988E-68	4	
Gdf10	3,73384E-72	0,3869	0,374	0,151	6,26948E-68	4	
Nell1	4,54927E-72	0,45771	0,546	0,273	7,63868E-68	4	
Rprm	1,68507E-71	0,4418	0,522	0,258	2,8294E-67	4	
Fam19a2	1,70661E-71	0,3262	0,291	0,1	2,86557E-67	4	
Kif21a	1,96625E-71	0,39702	0,993	0,973	3,30152E-67	4	
Ssbp3	2,45859E-71	0,35513	0,993	0,946	4,12822E-67	4	
Tub	3,55113E-71	0,4867	0,894	0,751	5,96271E-67	4	
Pdzrn3	9,41475E-71	0,30878	0,296	0,103	1,58083E-66	4	
Eef1a2	1,35471E-70	0,38884	0,997	0,958	2,2747E-66	4	
Sez6	1,37316E-70	0,45331	0,593	0,339	2,30567E-66	4	
Pla2g7	2,96655E-70	0,36926	0,417	0,178	4,98113E-66	4	
Hdac9	1,93444E-69	0,32576	0,338	0,13	3,24812E-65	4	
Sema5a	3,07533E-69	0,32459	0,333	0,125	5,16379E-65	4	
Myrip	7,56484E-69	0,51234	0,658	0,421	1,27021E-64	4	
Lynx1	2,57153E-67	0,38548	0,51	0,252	4,31786E-63	4	
Cdh2	5,77027E-67	0,38056	0,985	0,92	9,68886E-63	4	
Nmbr	2,07257E-66	0,27502	0,27	0,091	3,48005E-62	4	
Syt6	9,4073E-66	0,32907	0,252	0,082	1,57958E-61	4	
Phox2b	1,10481E-65	0,32425	1	0,992	1,85509E-61	4	
Stat3	3,37341E-65	0,48131	0,866	0,726	5,6643E-61	4	
Cacng4	3,8139E-65	0,47454	0,621	0,383	6,40391E-61	4	
Begain	1,91308E-64	0,49126	0,716	0,51	3,21225E-60	4	
Foxo3	7,35381E-64	0,45097	0,612	0,381	1,23478E-59	4	
Ptprt	1,13258E-63	0,35195	0,4	0,175	1,90171E-59	4	
Tspan3	3,42991E-63	0,46296	0,957	0,898	5,75916E-59	4	
Mapre3	6,8478E-62	0,4719	0,836	0,728	1,14981E-57	4	
Tnrc6c	8,84367E-62	0,39604	0,937	0,84	1,48494E-57	4	

Igsf3	9,06211E-62	0,43731	0,737	0,512	1,52162E-57	4	
Nedd9	1,33306E-61	0,32013	0,293	0,111	2,23834E-57	4	
Lrrn2	4,63517E-61	0,46796	0,722	0,518	7,78292E-57	4	
Pid1	5,43206E-61	0,45063	0,718	0,497	9,12097E-57	4	
Snrpn	6,997E-61	0,36902	0,992	0,983	1,17487E-56	4	
Cadm3	2,31528E-60	0,4221	0,88	0,747	3,88759E-56	4	
Dnm1	1,07069E-59	0,52241	0,764	0,621	1,7978E-55	4	
Rbm20	9,96914E-59	0,29443	0,282	0,107	1,67392E-54	4	
Nfasc	2,84411E-58	0,40476	0,833	0,679	4,77555E-54	4	
Setbp1	1,31372E-57	0,28016	0,286	0,11	2,20586E-53	4	
Chd5	1,55884E-57	0,35047	0,993	0,961	2,61745E-53	4	
Ddah1	2,06275E-57	0,36599	0,673	0,393	3,46356E-53	4	
Med25	6,30629E-57	0,46154	0,679	0,48	1,05889E-52	4	
Slc18a3	1,29561E-56	0,45712	0,758	0,498	2,17546E-52	4	
Rere	2,10513E-56	0,4485	0,744	0,574	3,53472E-52	4	
Ppp3ca	2,35595E-56	0,29346	0,997	0,962	3,95588E-52	4	
Plce1	3,10069E-56	0,42771	0,671	0,453	5,20636E-52	4	
Slit3	9,56033E-56	0,4213	0,586	0,366	1,60528E-51	4	
Oprl1	1,99684E-55	0,3874	0,467	0,255	3,35289E-51	4	
Galnt10	6,43917E-55	0,28928	0,303	0,124	1,0812E-50	4	
Myh10	1,01762E-54	0,47724	0,726	0,55	1,70868E-50	4	
Kcnb1	1,05289E-54	0,40604	0,733	0,529	1,76792E-50	4	
Rasd2	1,3773E-53	0,32816	0,47	0,242	2,31263E-49	4	
Cited2	6,7739E-53	0,40197	0,94	0,867	1,1374E-48	4	
Lrrtm1	1,11382E-52	0,36073	0,508	0,281	1,87022E-48	4	
Traf3	1,22222E-52	0,37468	0,544	0,323	2,05223E-48	4	
Rbms3	2,23086E-52	0,25909	1	0,992	3,74584E-48	4	
Tcaf1	4,36123E-52	0,35652	0,966	0,92	7,32294E-48	4	
Rin2	4,88869E-52	0,37211	0,408	0,211	8,20859E-48	4	
Cdc14a	6,71374E-52	0,31678	0,367	0,174	1,1273E-47	4	
Lhfpl2	1,21006E-51	0,32539	0,711	0,48	2,03181E-47	4	
Ptprj	2,59072E-51	0,42622	0,596	0,39	4,35008E-47	4	
Hk1	3,13908E-51	0,35696	0,925	0,872	5,27082E-47	4	
Impact	8,50903E-51	0,39788	0,935	0,889	1,42875E-46	4	
Gse1	1,91379E-50	0,28584	0,962	0,814	3,21344E-46	4	
Akt1	2,14549E-50	0,36465	0,84	0,689	3,60249E-46	4	
Fndc3b	3,29738E-50	0,37788	0,542	0,33	5,53662E-46	4	
Ankrd11	5,5126E-50	0,37798	0,938	0,866	9,25621E-46	4	
Dpysl4	2,31851E-49	0,33442	0,405	0,209	3,89302E-45	4	
Dcx	3,48274E-49	0,40962	0,678	0,49	5,84788E-45	4	
1810041L15I	8,22643E-49	0,38076	0,857	0,738	1,3813E-44	4	
Cacna1e	1,00519E-48	0,34368	0,706	0,483	1,68782E-44	4	
Ldoc1l	1,96321E-48	0,42213	0,661	0,473	3,29642E-44	4	
P2rx2	2,40179E-48	0,33314	0,999	0,982	4,03284E-44	4	
Nhsl2	2,56381E-48	0,33908	0,438	0,239	4,30489E-44	4	
Rbfox3	1,02416E-47	0,29517	0,492	0,272	1,71967E-43	4	
Brd4	1,1324E-46	0,41152	0,732	0,57	1,90141E-42	4	
Plxna4	1,28502E-46	0,30082	0,996	0,963	2,15767E-42	4	

Dner	1,64467E-46	0,42545	0,707	0,54	2,76156E-42	4	
Chrm2	2,59962E-46	0,28158	0,297	0,131	4,36501E-42	4	
Gm15800	4,8513E-46	0,4036	0,812	0,692	8,14582E-42	4	
AY036118	7,13076E-46	0,40356	0,932	0,84	1,19733E-41	4	
Gdi1	7,43843E-46	0,32491	0,993	0,973	1,24899E-41	4	
Bcl2l11	8,57557E-46	0,28512	0,334	0,156	1,43992E-41	4	
Xylt1	9,15385E-46	0,30403	0,462	0,256	1,53702E-41	4	
Sdc3	1,32168E-45	0,32333	0,927	0,814	2,21924E-41	4	
Ldlrad3	5,79714E-45	0,25492	0,315	0,145	9,73398E-41	4	
Sema3c	1,31225E-44	0,34434	0,43	0,236	2,2034E-40	4	
Scarb2	2,1795E-44	0,38653	0,66	0,482	3,6596E-40	4	
Rltpr	2,58937E-44	0,40077	0,571	0,381	4,34782E-40	4	
Ubqln2	1,70225E-43	0,35523	0,884	0,806	2,85825E-39	4	
Brinp1	1,68704E-42	0,35344	0,533	0,334	2,83271E-38	4	
Map1a	1,98805E-42	0,33985	0,936	0,866	3,33814E-38	4	
Faim2	1,94612E-41	0,39777	0,573	0,392	3,26774E-37	4	
Parvb	2,88812E-41	0,35299	0,75	0,588	4,84944E-37	4	
Plekha6	7,6004E-41	0,32575	0,879	0,773	1,27618E-36	4	
Grin1	2,09671E-40	0,36228	0,748	0,595	3,52058E-36	4	
Mapk8ip2	8,00324E-40	0,3522	0,902	0,836	1,34382E-35	4	
Pitpnm2	9,44706E-40	0,33155	0,8	0,649	1,58626E-35	4	
Prkce	1,27745E-39	0,2955	0,897	0,81	2,14497E-35	4	
Clasp2	1,99157E-39	0,33811	0,85	0,747	3,34405E-35	4	
Hivep3	5,57248E-39	0,3407	0,726	0,552	9,35675E-35	4	
Magi3	6,38233E-39	0,34055	0,492	0,308	1,07166E-34	4	
Ednrb	2,36061E-38	0,25801	0,307	0,147	3,96371E-34	4	
B4galt6	3,5302E-38	0,33679	0,805	0,691	5,92756E-34	4	
Rgl1	4,16654E-38	0,27068	0,343	0,178	6,99604E-34	4	
Grb10	4,99241E-38	0,2621	0,313	0,155	8,38276E-34	4	
Gtf3c1	1,65308E-37	0,34228	0,677	0,498	2,77569E-33	4	
Gpc6	1,71446E-37	0,32177	0,668	0,489	2,87875E-33	4	
Scml4	1,25945E-36	0,27965	0,382	0,215	2,11474E-32	4	
Magee1	4,42688E-36	0,33262	0,779	0,653	7,43318E-32	4	
Gnaz	5,43208E-36	0,35775	0,734	0,619	9,121E-32	4	
Cabp1	9,67707E-36	0,34493	0,634	0,47	1,62488E-31	4	
Hs2st1	1,44629E-35	0,33436	0,579	0,408	2,42847E-31	4	
Tom1l2	1,65267E-35	0,34865	0,73	0,598	2,775E-31	4	
Dbt	1,94697E-35	0,27	0,385	0,216	3,26916E-31	4	
Mapk3	2,56078E-35	0,26847	0,998	0,99	4,29981E-31	4	
Ap2s1	2,72954E-35	0,27158	0,974	0,935	4,58318E-31	4	
Mapre2	3,02094E-35	0,28033	0,902	0,814	5,07247E-31	4	
Clip3	4,51579E-35	0,32641	0,89	0,844	7,58246E-31	4	
Nfia	4,87235E-35	0,31654	0,686	0,516	8,18116E-31	4	
Stom	1,20152E-34	0,34037	0,884	0,805	2,01747E-30	4	
Snap91	1,85046E-34	0,33751	0,75	0,643	3,10711E-30	4	
Sema4d	2,41307E-34	0,33841	0,622	0,466	4,05179E-30	4	
Ccser2	3,64808E-34	0,25806	0,907	0,807	6,12548E-30	4	
Srebf1	3,83647E-34	0,31178	0,472	0,303	6,44181E-30	4	

Pou2f2	4,0259E-34	0,26364	0,393	0,224	6,7599E-30	4	
Twsg1	5,73922E-34	0,34776	0,607	0,447	9,63672E-30	4	
Ptprs	8,12141E-34	0,2582	0,88	0,752	1,36367E-29	4	
Snap47	9,33772E-34	0,3103	0,925	0,878	1,5679E-29	4	
Lonrf2	2,33877E-33	0,3333	0,633	0,466	3,92702E-29	4	
Zbtb18	3,32794E-33	0,30779	0,74	0,604	5,58794E-29	4	
Abr	6,25415E-33	0,33427	0,728	0,595	1,05013E-28	4	
Sema6d	6,38567E-33	0,30507	0,79	0,674	1,07222E-28	4	
Gaa	1,04733E-32	0,33946	0,91	0,849	1,75857E-28	4	
Ank	2,15563E-32	0,28721	0,417	0,253	3,61953E-28	4	
Chgb	2,22292E-32	0,26998	0,859	0,745	3,73251E-28	4	
Fam49a	7,94127E-32	0,331	0,584	0,424	1,33342E-27	4	
Rab3b	9,83459E-32	0,25954	0,646	0,442	1,65133E-27	4	
U2af2	1,22489E-31	0,32338	0,746	0,622	2,05671E-27	4	
Lrrc58	2,16555E-31	0,2545	0,913	0,819	3,63618E-27	4	
Fis1	2,21629E-31	0,28421	0,973	0,956	3,72136E-27	4	
Hipk1	2,28127E-31	0,30353	0,685	0,535	3,83049E-27	4	
Vamp4	4,23287E-31	0,34815	0,774	0,671	7,10742E-27	4	
Cacna2d2	4,53295E-31	0,25495	0,404	0,242	7,61127E-27	4	
Myt1l	5,28633E-31	0,29675	0,707	0,576	8,87628E-27	4	
Cadm2	6,90606E-31	0,25519	0,331	0,183	1,1596E-26	4	
Gtpbp1	7,74039E-31	0,28521	0,382	0,231	1,29969E-26	4	
Tspan9	2,04906E-30	0,30214	0,617	0,454	3,44057E-26	4	
Sv2a	2,56402E-30	0,28572	0,859	0,78	4,30525E-26	4	
Ciapin1	3,85249E-30	0,33418	0,758	0,667	6,46871E-26	4	
Aff4	8,17415E-30	0,25607	0,933	0,892	1,37252E-25	4	
Ly6h	1,58902E-29	0,25627	0,671	0,478	2,66812E-25	4	
Ogdh	1,83336E-29	0,31843	0,793	0,714	3,0784E-25	4	
March6	2,27288E-29	0,29987	0,772	0,679	3,81639E-25	4	
Tnpo1	2,51787E-29	0,27111	0,752	0,613	4,22776E-25	4	
Fbxl16	2,56548E-29	0,2957	0,749	0,633	4,30769E-25	4	
Strip1	2,95499E-29	0,31217	0,607	0,455	4,96172E-25	4	
Matr3	3,67138E-29	0,26591	0,928	0,875	6,16461E-25	4	
Zdhhc17	5,98668E-29	0,31473	0,707	0,581	1,00522E-24	4	
Grik4	9,24946E-29	0,26107	0,462	0,301	1,55308E-24	4	
Rnps1	1,41831E-28	0,29145	0,777	0,696	2,38149E-24	4	
Ppp1r9b	1,5771E-28	0,31344	0,703	0,559	2,64811E-24	4	
Csnk1d	1,64573E-28	0,30774	0,74	0,638	2,76335E-24	4	
Hacd3	3,32018E-28	0,30333	0,65	0,511	5,57491E-24	4	
Sparc	6,3704E-28	0,32715	0,741	0,608	1,06965E-23	4	
Inpp4a	8,78134E-28	0,30577	0,705	0,587	1,47448E-23	4	
Epb41l2	8,81284E-28	0,31235	0,526	0,38	1,47976E-23	4	
Fam163a	1,32024E-27	0,32859	0,717	0,598	2,21681E-23	4	
Filip1	1,50448E-27	0,28864	0,882	0,813	2,52618E-23	4	
Zdhhc8	1,86216E-27	0,29294	0,694	0,547	3,12675E-23	4	
Dnajc6	1,86815E-27	0,30272	0,623	0,491	3,13681E-23	4	
Tigar	4,24069E-27	0,31787	0,546	0,402	7,12055E-23	4	
Morn4	4,24593E-27	0,315	0,629	0,5	7,12935E-23	4	

Txnip	4,90426E-27	0,33638	0,403	0,261	8,23474E-23	4	
Tspan7	9,34664E-27	0,2883	0,864	0,797	1,56939E-22	4	
Per1	5,81794E-26	0,25146	0,409	0,265	9,76891E-22	4	
Chd6	7,2834E-26	0,28909	0,723	0,622	1,22296E-21	4	
Hr	1,27891E-25	0,25188	0,547	0,396	2,14742E-21	4	
Dlg4	1,64003E-25	0,27881	0,715	0,602	2,75378E-21	4	
Prpf8	1,70049E-25	0,29269	0,703	0,585	2,85529E-21	4	
Ddx3x	1,81547E-25	0,29034	0,807	0,728	3,04835E-21	4	
Mpp2	2,89631E-25	0,28004	0,556	0,408	4,8632E-21	4	
Cpt1a	5,8071E-25	0,31825	0,611	0,486	9,75071E-21	4	
Ilf3	8,47665E-25	0,28923	0,615	0,49	1,42331E-20	4	
Wdr6	9,80982E-25	0,26536	0,879	0,84	1,64717E-20	4	
Adam9	1,02088E-24	0,28029	0,407	0,273	1,71415E-20	4	
Adam19	4,10151E-24	0,2646	0,512	0,367	6,88684E-20	4	
Eml6	6,09526E-24	0,26366	0,661	0,535	1,02346E-19	4	
Sod1	9,78138E-24	0,2549	0,958	0,924	1,64239E-19	4	
Btbd2	1,05946E-23	0,28738	0,61	0,494	1,77893E-19	4	
Stox2	1,84431E-23	0,25059	0,581	0,44	3,09678E-19	4	
Zbtb44	2,70213E-23	0,26331	0,574	0,439	4,53715E-19	4	
Gcgr	5,12249E-23	0,25061	0,454	0,309	8,60117E-19	4	
Epha5	6,32626E-23	0,29139	0,343	0,214	1,06224E-18	4	
Synj1	7,666E-22	0,26436	0,725	0,624	1,2872E-17	4	
Git1	1,10112E-21	0,25903	0,488	0,359	1,84888E-17	4	
Dip2b	1,17724E-21	0,25095	0,694	0,577	1,97671E-17	4	
Tspyl1	2,26358E-21	0,27606	0,637	0,528	3,80077E-17	4	
Nsf	2,72333E-21	0,27422	0,677	0,572	4,57274E-17	4	
Ankrd10	4,94544E-20	0,25383	0,553	0,433	8,30388E-16	4	
Got2	5,68804E-20	0,27614	0,79	0,758	9,5508E-16	4	
Nipal3	7,70342E-20	0,25658	0,735	0,656	1,29348E-15	4	
Cdc42bpb	8,02405E-20	0,25456	0,664	0,563	1,34732E-15	4	
Fam171a2	9,02376E-20	0,27686	0,671	0,588	1,51518E-15	4	
Sumo3	1,57804E-18	0,25423	0,777	0,711	2,64969E-14	4	
Atn1.1	1,83597E-18	0,25856	0,605	0,493	3,08278E-14	4	
Cxxc4	7,34534E-18	0,25328	0,7	0,604	1,23336E-13	4	
Raly	1,00521E-14	0,26314	0,655	0,603	1,68785E-10	4	
Meg3	0	1,22497	1	1	0	5	smENC2d
Zbtb20	2,2609E-265	1,28467	0,998	0,921	3,7963E-261	5	
Snhg11	3,385E-265	1,00554	1	0,994	5,6837E-261	5	
Gfra2	1,8843E-263	1,26144	0,934	0,411	3,1639E-259	5	
mt-Nd2	3,5802E-263	0,82469	1	1	6,0115E-259	5	
mt-Atp8	3,6859E-255	0,96831	1	1	6,1891E-251	5	
Ppp3ca	3,7605E-247	1,07486	1	0,963	6,3143E-243	5	
Elavl4	4,2935E-247	0,9474	1	0,997	7,2092E-243	5	
6330403A02	3,9995E-245	1,07612	1	0,931	6,7156E-241	5	
mt-Nd4l	5,0397E-245	0,87876	1	1	8,4622E-241	5	
Hoxa5	3,1846E-233	1,22727	1	0,936	5,3472E-229	5	
mt-Nd1	2,8478E-231	0,76596	1	1	4,7817E-227	5	
Ntrk3	5,5435E-220	1,17539	0,978	0,689	9,308E-216	5	

Scube1	2,0961E-212	0,99414	1	0,899	3,5195E-208	5	
Pcdh7	4,8191E-212	1,14279	0,822	0,349	8,0917E-208	5	
Mcam	1,3161E-202	1,11947	0,976	0,646	2,2098E-198	5	
mt-Nd3	5,0534E-200	0,79137	1	1	8,4852E-196	5	
Pak3	1,4859E-193	1,09285	0,953	0,685	2,4949E-189	5	
Dst	5,5726E-191	0,75999	1	0,997	9,357E-187	5	
Tshz2	1,3987E-188	1,01955	0,999	0,835	2,3486E-184	5	
Enah	1,9097E-188	0,95595	0,994	0,879	3,2065E-184	5	
Gsk3b	1,8959E-187	0,81558	0,999	0,984	3,1834E-183	5	
Adrbk2	8,1555E-186	0,87306	0,999	0,939	1,3694E-181	5	
Itga6	1,5055E-183	1,0463	0,832	0,454	2,5279E-179	5	
Plod2	1,5121E-180	0,96989	0,976	0,827	2,539E-176	5	
Slc5a7	1,735E-178	1,09572	0,904	0,596	2,9133E-174	5	
Bche	4,744E-176	0,98646	0,989	0,775	7,9656E-172	5	
Prnp	4,1809E-171	0,88767	1	0,977	7,0201E-167	5	
Nrxn3	1,9352E-170	0,91263	0,854	0,42	3,2494E-166	5	
Cd47	2,0485E-169	0,87822	0,999	0,928	3,4396E-165	5	
Rbms1	3,0725E-166	0,93135	0,965	0,794	5,159E-162	5	
Rbms3	4,1132E-166	0,7997	1	0,992	6,9064E-162	5	
Ptprs	7,7034E-166	0,91627	0,958	0,747	1,2935E-161	5	
Tcf4	2,9849E-165	0,71234	1	0,991	5,012E-161	5	
Bcl2	6,0538E-165	1,034	0,798	0,44	1,0165E-160	5	
Syt17	2,5004E-160	0,92118	0,963	0,752	4,1984E-156	5	
Nfix	1,3374E-159	0,90258	0,963	0,739	2,2456E-155	5	
Nfib	3,5411E-158	0,85992	0,987	0,872	5,9459E-154	5	
Casz1	3,1156E-157	0,89703	0,767	0,342	5,2313E-153	5	
Snap25	4,1328E-157	0,70571	1	0,999	6,9394E-153	5	
Kcnb1	6,3122E-157	0,98663	0,819	0,526	1,0599E-152	5	
Atp1b1	4,2925E-155	0,83659	1	0,858	7,2075E-151	5	
Bnc2	1,1079E-154	0,77532	0,588	0,205	1,8603E-150	5	
Spock2	5,6263E-154	0,71125	1	0,977	9,4471E-150	5	
Celf4	5,6436E-154	0,72178	1	0,974	9,4761E-150	5	
Timp3	2,8004E-153	1,00742	0,86	0,436	4,7021E-149	5	
Fam19a5	3,128E-150	0,93169	0,769	0,367	5,2523E-146	5	
Slc36a1	1,5367E-149	0,78183	1	0,961	2,5804E-145	5	
Gria2	5,2064E-148	0,82096	0,994	0,928	8,742E-144	5	
Slc10a4	2,6504E-147	0,80576	0,988	0,791	4,4503E-143	5	
mt-Atp6	7,2935E-145	0,39169	1	1	1,2247E-140	5	
mt-Nd4	3,0143E-144	0,43339	1	1	5,0612E-140	5	
Adamts9	6,5063E-144	0,85383	0,646	0,264	1,0925E-139	5	
Slc7a14	8,941E-143	0,84667	0,981	0,827	1,5013E-138	5	
Pura	3,9658E-142	0,73656	0,998	0,965	6,659E-138	5	
Mycbp2	4,1147E-142	0,68725	1	0,961	6,909E-138	5	
Smarca2	3,4661E-141	0,72469	1	0,942	5,82E-137	5	
mt-Nd5	4,7192E-141	0,61808	1	1	7,924E-137	5	
Parm1	1,4636E-137	0,95284	0,994	0,886	2,4575E-133	5	
Ssbp3	1,892E-137	0,71554	1	0,946	3,1768E-133	5	
Prkce	8,3021E-137	0,80465	0,963	0,806	1,394E-132	5	

Gse1	1,8271E-134	0,78572	0,98	0,817	3,0679E-130	5	
Syn2	3,4573E-134	0,52381	1	1	5,8052E-130	5	
Rbfox1	2,0243E-133	0,83175	0,881	0,65	3,3991E-129	5	
Ryr2	1,8095E-132	0,8028	0,614	0,26	3,0384E-128	5	
Nlgn1	8,799E-132	0,87677	0,709	0,389	1,4774E-127	5	
Mapt	1,1185E-131	0,67097	0,999	0,971	1,8781E-127	5	
Cnr1	1,9314E-131	0,56894	1	0,997	3,243E-127	5	
Ppm1h	4,2899E-131	0,77743	0,912	0,681	7,2032E-127	5	
Prkacb	2,3918E-130	0,73852	0,99	0,92	4,0161E-126	5	
Nktr	6,4615E-130	0,84063	0,924	0,742	1,0849E-125	5	
Hoxb5	1,8952E-128	0,71692	1	0,954	3,1823E-124	5	
Peg3	1,352E-127	0,67123	1	0,979	2,2701E-123	5	
Syt11	1,2324E-126	0,64621	1	0,991	2,0693E-122	5	
Nsg2	2,1303E-126	0,61561	1	0,997	3,577E-122	5	
Ddx5	3,7253E-126	0,55269	1	0,999	6,2551E-122	5	
Elavl3	7,4466E-126	0,75379	0,994	0,916	1,2504E-121	5	
Kcnma1	4,4573E-125	0,89623	0,75	0,501	7,4842E-121	5	
Sdc3	7,9537E-125	0,75275	0,974	0,812	1,3355E-120	5	
Kmt2e	1,0929E-124	0,74833	0,923	0,747	1,8351E-120	5	
Kcnq1ot1	9,4619E-124	0,90026	0,972	0,844	1,5887E-119	5	
Ogt	1,8247E-123	0,81006	0,857	0,631	3,0638E-119	5	
Tcaf1	2,1959E-122	0,69105	0,995	0,918	3,6872E-118	5	
Mapre2	5,6211E-121	0,78753	0,952	0,811	9,4384E-117	5	
Atp2b4	6,2916E-121	0,75793	0,935	0,721	1,0564E-116	5	
Fus	8,1453E-120	0,6751	0,992	0,938	1,3677E-115	5	
Tcf7l2	8,4808E-120	0,68774	0,99	0,829	1,424E-115	5	
1810041L15I	1,1473E-118	0,76877	0,93	0,734	1,9264E-114	5	
B4galt6	2,042E-118	0,7868	0,907	0,683	3,4287E-114	5	
Plekha6	2,9811E-118	0,69594	0,946	0,769	5,0055E-114	5	
Klf7	2,2276E-117	0,68332	0,992	0,944	3,7403E-113	5	
Phox2b	8,2367E-117	0,6034	0,999	0,993	1,383E-112	5	
Tnpo1	1,3796E-116	0,78353	0,836	0,608	2,3164E-112	5	
Tulp4	1,1709E-115	0,68127	0,963	0,821	1,966E-111	5	
Rtn3	1,9291E-115	0,56794	1	0,997	3,2392E-111	5	
Camk2b	3,9391E-115	0,74837	0,931	0,715	6,6142E-111	5	
mt-Co3	7,1798E-115	0,3274	1	1	1,2056E-110	5	
Ywhag	1,0247E-113	0,57135	0,999	0,991	1,7206E-109	5	
Caln1	1,4835E-113	0,67528	0,485	0,175	2,4909E-109	5	
Ccser2	2,2797E-113	0,66421	0,97	0,804	3,8278E-109	5	
Calcb	8,024E-113	0,60201	0,987	0,502	1,3473E-108	5	
Thra	1,8795E-112	0,56659	1	0,993	3,1559E-108	5	
Igfbp5	4,3944E-112	0,7094	0,522	0,196	7,3786E-108	5	
mt-Cytb	2,1209E-111	0,31023	1	1	3,5612E-107	5	
Lrrc58	8,2854E-111	0,7213	0,969	0,816	1,3912E-106	5	
Scn3a	1,8205E-110	0,67761	0,968	0,816	3,0568E-106	5	
Wnk1	9,282E-110	0,70734	0,947	0,798	1,5585E-105	5	
Zcchc12	8,6293E-109	0,75515	0,992	0,876	1,4489E-104	5	
Rab6b	1,0402E-108	0,77454	0,995	0,913	1,7466E-104	5	

Ppp2r2c	1,0526E-107	0,7226	0,952	0,792	1,7675E-103	5	
Kif1b	4,0581E-107	0,55459	1	0,992	6,814E-103	5	
Snrnp70	5,3348E-107	0,68724	0,93	0,772	8,9577E-103	5	
Zmiz1	5,707E-107	0,73226	0,909	0,71	9,5826E-103	5	
Camta1	7,1643E-107	0,7057	0,974	0,857	1,2029E-102	5	
Ddx17	9,6925E-107	0,70305	0,939	0,793	1,6275E-102	5	
Scd2	2,1812E-106	0,75902	0,992	0,911	3,6625E-102	5	
Flrt1	1,5218E-105	0,58459	0,427	0,144	2,5553E-101	5	
Scg2	1,5892E-105	0,50392	1	1	2,6685E-101	5	
Ddah1	1,7279E-105	0,74707	0,726	0,396	2,9014E-101	5	
Nfasc	1,1521E-104	0,69266	0,893	0,677	1,9345E-100	5	
App	1,0973E-103	0,53328	1	0,994	1,8425E-99	5	
Arhgap26	4,6458E-103	0,6643	0,966	0,815	7,8008E-99	5	
Tnrc6c	7,697E-103	0,66568	0,954	0,841	1,2924E-98	5	
Rbfox2	2,6063E-102	0,66061	0,957	0,844	4,37627E-98	5	
Trp53i11	7,9113E-102	0,60746	0,988	0,636	1,32839E-97	5	
Avpr1a	9,6134E-101	0,66628	0,554	0,236	1,61419E-96	5	
Celf3	1,3298E-100	0,65688	0,964	0,839	2,23283E-96	5	
Luc7l2	4,1207E-100	0,68604	0,942	0,795	6,91904E-96	5	
Ptprd	1,4008E-99	0,6589	0,968	0,877	2,3521E-95	5	
Igsf3	1,5951E-99	0,7566	0,748	0,518	2,67837E-95	5	
Tns1	1,41829E-98	0,65688	0,936	0,79	2,38145E-94	5	
Clk1	2,03225E-98	0,66212	0,941	0,786	3,41236E-94	5	
Ago2	4,06823E-98	0,68824	0,893	0,718	6,83096E-94	5	
Taok1	4,66045E-98	0,6639	0,93	0,743	7,82535E-94	5	
Tmod2	2,11015E-96	0,63863	0,984	0,903	3,54315E-92	5	
Frmd4a	3,76919E-96	0,59273	0,993	0,923	6,32885E-92	5	
Nrp2	2,31937E-95	0,76238	0,58	0,274	3,89445E-91	5	
Kmt2a	2,65359E-95	0,66654	0,887	0,702	4,45565E-91	5	
Msi2	6,60936E-95	0,70123	0,911	0,775	1,10978E-90	5	
Cbarp	6,12007E-94	0,62007	0,983	0,886	1,02762E-89	5	
Pnisr	7,36817E-94	0,71837	0,887	0,723	1,23719E-89	5	
Cbx6	8,904E-94	0,54987	0,998	0,942	1,49507E-89	5	
Nav1	1,20694E-93	0,60032	0,988	0,909	2,02657E-89	5	
Dnmt3a	1,18003E-92	0,65498	0,898	0,694	1,98139E-88	5	
Timp2	1,29638E-92	0,60556	0,999	0,971	2,17675E-88	5	
Pitpnm2	2,48363E-92	0,6643	0,853	0,648	4,17027E-88	5	
Dpysl2	2,66269E-91	0,59339	0,999	0,982	4,47092E-87	5	
Zwint	1,11537E-90	0,57263	1	0,987	1,87281E-86	5	
Pbx1	1,40093E-90	0,63364	0,915	0,75	2,3523E-86	5	
Slc6a6	2,16823E-90	0,71901	0,767	0,567	3,64068E-86	5	
Cacna1e	2,79808E-90	0,69408	0,746	0,486	4,69826E-86	5	
Phip	4,56414E-90	0,72108	0,826	0,629	7,66364E-86	5	
Myt1l	4,666E-90	0,73087	0,775	0,573	7,83468E-86	5	
Map2	5,5644E-90	0,63407	0,96	0,836	9,34318E-86	5	
Eml6	8,14211E-90	0,7044	0,748	0,529	1,36714E-85	5	
Nfia	1,92427E-89	0,71869	0,747	0,515	3,23104E-85	5	
Plppr4	3,39375E-89	0,74097	0,776	0,576	5,69845E-85	5	

Napb	9,57893E-89	0,63366	0,936	0,782	1,6084E-84	5	
BC005537	2,46105E-88	0,64796	0,897	0,721	4,13235E-84	5	
Tspan3	4,78903E-88	0,57611	0,984	0,897	8,04127E-84	5	
Fam171b	5,25914E-88	0,61628	0,942	0,775	8,83062E-84	5	
Nell1	1,04295E-87	0,65349	0,584	0,277	1,75122E-83	5	
Syt1	7,35794E-87	0,39421	1	1	1,23547E-82	5	
Unc80	2,11703E-86	0,65913	0,83	0,64	3,55471E-82	5	
Mafg	6,94445E-86	0,66042	0,833	0,654	1,16604E-81	5	
Syt4	9,65837E-86	0,72285	0,998	0,961	1,62174E-81	5	
Ddx6	1,25217E-85	0,61407	0,857	0,678	2,10251E-81	5	
Parvb	4,42843E-85	0,67073	0,799	0,588	7,43577E-81	5	
Stox2	5,21885E-85	0,73151	0,671	0,435	8,76298E-81	5	
Adgrb3	1,04377E-84	0,63259	0,895	0,737	1,75259E-80	5	
Necab1	3,35335E-84	0,63026	0,971	0,863	5,63061E-80	5	
Zcchc7	4,99534E-84	0,66181	0,823	0,639	8,38768E-80	5	
Sema5a	8,60853E-84	0,54032	0,369	0,127	1,44546E-79	5	
Usp22	1,64415E-83	0,60411	0,952	0,823	2,7607E-79	5	
Sorl1	3,13037E-83	0,67235	0,777	0,58	5,25621E-79	5	
Klf13	5,01079E-83	0,61208	0,888	0,705	8,41361E-79	5	
Mbnl1	6,82243E-83	0,60091	0,912	0,76	1,14555E-78	5	
Hivep3	1,18379E-82	0,68224	0,755	0,554	1,98771E-78	5	
Jph4	7,57704E-82	0,54477	0,978	0,897	1,27226E-77	5	
Reep1	5,79388E-81	0,59323	0,905	0,752	9,72851E-77	5	
Nfic	1,69158E-80	0,49895	0,993	0,948	2,84034E-76	5	
Nedd4	2,21544E-80	0,48145	0,998	0,988	3,71994E-76	5	
Vat1l	2,98957E-80	0,65486	0,964	0,827	5,01978E-76	5	
Purb	7,61949E-80	0,60882	0,936	0,798	1,27939E-75	5	
Adcy1	2,57641E-79	0,60968	0,591	0,307	4,32605E-75	5	
Kcnn2	1,00088E-78	0,56289	0,484	0,219	1,68058E-74	5	
Tub	7,33515E-78	0,5993	0,922	0,753	1,23165E-73	5	
Arglu1	1,42655E-77	0,56847	0,951	0,854	2,39531E-73	5	
Vipr2	1,97716E-77	0,54281	0,566	0,264	3,31985E-73	5	
4930402H24	2,6459E-77	0,62013	0,781	0,593	4,44272E-73	5	
Tnrc6a	2,80816E-77	0,57436	0,949	0,846	4,71517E-73	5	
Usp34	5,43546E-77	0,59973	0,898	0,741	9,12669E-73	5	
Cdc14a	5,53742E-77	0,54981	0,422	0,174	9,29788E-73	5	
Cds2	6,01142E-77	0,53194	0,994	0,91	1,00938E-72	5	
Cadm3	6,92937E-77	0,55713	0,917	0,748	1,16351E-72	5	
Itpr1	1,5896E-76	0,62653	0,503	0,247	2,66909E-72	5	
Akap13	1,81705E-76	0,57561	0,961	0,876	3,05101E-72	5	
Sel1l3	3,42089E-76	0,68575	0,777	0,596	5,74402E-72	5	
Arpp21	7,53801E-76	0,58867	0,97	0,885	1,26571E-71	5	
Sept3	8,7742E-76	0,7152	0,696	0,501	1,47328E-71	5	
Gpatch8	3,10651E-75	0,55697	0,935	0,84	5,21614E-71	5	
Plxna4	9,82146E-75	0,49427	1	0,964	1,64912E-70	5	
Serinc1	1,1468E-74	0,46771	0,999	0,988	1,92559E-70	5	
Lrrfip1	3,45922E-74	0,51659	0,978	0,914	5,80838E-70	5	
Rtn1	3,95937E-74	0,43655	1	0,999	6,64818E-70	5	

Ablim1	2,42551E-73	0,68174	0,687	0,494	4,07267E-69	5	
Kif21a	2,53561E-73	0,45225	0,998	0,973	4,25755E-69	5	
Gnaq	2,58266E-73	0,58316	0,923	0,793	4,33654E-69	5	
Cdc42bpa	3,43456E-73	0,55861	0,864	0,701	5,76697E-69	5	
Kmt2c	5,01599E-73	0,66157	0,762	0,589	8,42235E-69	5	
Id2	5,69213E-73	0,59226	0,97	0,879	9,55766E-69	5	
Rbm5	8,44474E-73	0,64254	0,845	0,695	1,41796E-68	5	
Sacs	9,82426E-73	0,72115	0,684	0,486	1,64959E-68	5	
Tmem64	3,91581E-72	0,57881	0,98	0,883	6,57504E-68	5	
Csmd2	2,56455E-71	0,46339	0,392	0,158	4,30614E-67	5	
Dnm3	4,6816E-71	0,57244	0,856	0,702	7,86087E-67	5	
Plcxd3	5,71212E-71	0,65005	0,621	0,386	9,59122E-67	5	
Cyth3	6,82511E-71	0,41242	1	0,995	1,146E-66	5	
PISD	1,33951E-70	0,64982	0,871	0,721	2,24916E-66	5	
Mast4	3,78845E-70	0,61512	0,522	0,27	6,36118E-66	5	
Tmem30a	8,83575E-70	0,59394	0,964	0,855	1,48361E-65	5	
Ptprf	2,12978E-69	0,62945	0,628	0,414	3,57611E-65	5	
Luc7l3	4,30065E-69	0,57114	0,905	0,798	7,22122E-65	5	
Atxn1	4,96788E-69	0,53335	0,449	0,202	8,34157E-65	5	
Chat	7,43304E-69	0,45388	0,372	0,145	1,24808E-64	5	
5330434G04	1,15128E-68	0,56133	0,928	0,784	1,93311E-64	5	
Fat4	1,39947E-68	0,46677	0,372	0,144	2,34984E-64	5	
Stat3	1,78723E-68	0,56355	0,911	0,725	3,00094E-64	5	
Atxn7l3b	2,03272E-68	0,5402	0,964	0,874	3,41314E-64	5	
Synj1	4,2645E-68	0,62047	0,786	0,621	7,16052E-64	5	
Myrip	4,37183E-68	0,62459	0,653	0,429	7,34074E-64	5	
Etnk1	6,9536E-68	0,53557	0,964	0,867	1,16758E-63	5	
Myo9a	1,38173E-67	0,55772	0,818	0,663	2,32006E-63	5	
Ptprj	1,53729E-67	0,64322	0,615	0,394	2,58127E-63	5	
Snca	3,68458E-67	0,49517	0,996	0,955	6,18678E-63	5	
Mfsd6	3,83624E-67	0,57304	0,791	0,645	6,44144E-63	5	
Cdk14	5,35224E-67	0,43792	0,325	0,117	8,98695E-63	5	
Hnrnph1	5,80063E-67	0,51117	0,881	0,727	9,73984E-63	5	
Prrc2b	7,26206E-67	0,4838	0,981	0,882	1,21937E-62	5	
Xylt1	7,92168E-67	0,59847	0,497	0,259	1,33013E-62	5	
Arl8a	3,45382E-66	0,42498	1	0,983	5,79931E-62	5	
Zmynd11	4,69018E-66	0,54205	0,85	0,711	7,87528E-62	5	
Dclk1	5,03248E-66	0,54738	0,925	0,785	8,45004E-62	5	
Ash1l	5,92181E-66	0,53796	0,865	0,707	9,94331E-62	5	
Sfpq	8,58412E-66	0,56312	0,897	0,789	1,44136E-61	5	
Ndrg4	2,38123E-65	0,55338	0,957	0,852	3,99832E-61	5	
Ina	3,4588E-65	0,56456	0,894	0,736	5,80766E-61	5	
Gnao1	6,48473E-65	0,4434	0,998	0,964	1,08885E-60	5	
Clstn1	1,21687E-64	0,5218	0,971	0,852	2,04325E-60	5	
Csnk1a1	1,98038E-64	0,45888	0,981	0,936	3,32525E-60	5	
Ttc14	2,61215E-64	0,59815	0,791	0,63	4,38606E-60	5	
Ttbk2	2,78955E-64	0,58841	0,803	0,635	4,68393E-60	5	
Pcdh17	3,25987E-64	0,62422	0,963	0,833	5,47364E-60	5	

Zfp503	4,97623E-64	0,46342	0,413	0,18	8,35558E-60	5	
Xiap	7,73236E-64	0,58843	0,77	0,617	1,29834E-59	5	
Sphkap	1,04472E-63	0,54887	0,522	0,26	1,75419E-59	5	
Sox11	1,10718E-63	0,68164	0,544	0,326	1,85907E-59	5	
Brd2	2,11335E-63	0,51852	0,91	0,782	3,54853E-59	5	
Usp9x	4,99513E-63	0,47008	0,972	0,884	8,38732E-59	5	
Pclo	6,47531E-63	0,5398	0,921	0,812	1,08727E-58	5	
Bicd1	6,61462E-63	0,5758	0,783	0,649	1,11066E-58	5	
Gm26917	1,03788E-62	0,71822	0,7	0,51	1,7427E-58	5	
Pgm2l1	1,19613E-62	0,59118	0,791	0,643	2,00842E-58	5	
Sh3pxd2a	1,70149E-62	0,56027	0,765	0,599	2,85697E-58	5	
Rab6a	4,14765E-62	0,49378	0,976	0,9	6,96432E-58	5	
Ttc3	5,98614E-62	0,41332	1	0,991	1,00513E-57	5	
Miat	6,14072E-62	0,56007	0,44	0,214	1,03109E-57	5	
Cpe	1,02298E-61	0,50753	0,995	0,934	1,71769E-57	5	
Zeb2	1,93019E-61	0,49598	0,817	0,633	3,24098E-57	5	
Zfand5	6,06438E-61	0,53344	0,889	0,778	1,01827E-56	5	
Ids	1,84315E-60	0,50004	0,969	0,877	3,09483E-56	5	
Neat1	2,12353E-60	0,67478	0,507	0,275	3,56563E-56	5	
Larp1	2,70941E-60	0,59084	0,753	0,601	4,54938E-56	5	
Nrxn2	1,05921E-58	0,41877	0,993	0,96	1,77853E-54	5	
Matr3	1,88343E-58	0,47289	0,965	0,873	3,16246E-54	5	
Nrxn1	2,03402E-58	0,38536	0,999	0,988	3,41532E-54	5	
Adgrl1	2,65398E-58	0,40792	0,972	0,87	4,4563E-54	5	
Chd3	2,73463E-58	0,4335	0,994	0,949	4,59172E-54	5	
Tle3	3,03516E-58	0,44507	0,428	0,2	5,09634E-54	5	
Zdhhc2	3,67739E-58	0,5905	0,776	0,634	6,17471E-54	5	
Inpp4a	4,12821E-58	0,56868	0,735	0,588	6,93167E-54	5	
Tmem178b	4,88341E-58	0,62988	0,712	0,547	8,19974E-54	5	
Cdh2	8,39668E-58	0,4548	0,988	0,922	1,40989E-53	5	
Zbtb18	1,26355E-57	0,51914	0,78	0,604	2,12162E-53	5	
Sez6	1,56149E-57	0,55725	0,574	0,349	2,62189E-53	5	
Sema3c	1,97512E-57	0,58072	0,458	0,239	3,31642E-53	5	
Cers4	2,82772E-57	0,52603	0,892	0,788	4,74803E-53	5	
Igf1r	4,09114E-57	0,51809	0,779	0,628	6,86943E-53	5	
Whsc1	5,39908E-57	0,5537	0,77	0,633	9,0656E-53	5	
Cnih3	2,90404E-56	0,40039	0,363	0,152	4,87617E-52	5	
H2afy	3,82916E-56	0,42898	0,996	0,956	6,42955E-52	5	
Rspo2	4,76505E-56	0,47525	0,471	0,234	8,001E-52	5	
Fbrsl1	4,83342E-56	0,55101	0,585	0,384	8,11579E-52	5	
Atp2a2	5,91181E-56	0,46796	0,948	0,838	9,92652E-52	5	
Zcchc18	8,88021E-56	0,43111	0,994	0,955	1,49108E-51	5	
Huwe1	9,5067E-56	0,4768	0,923	0,808	1,59627E-51	5	
Scarb2	1,13463E-55	0,5885	0,664	0,487	1,90516E-51	5	
Macf1	1,64073E-55	0,49614	0,928	0,817	2,75494E-51	5	
Sema6d	2,17285E-55	0,52145	0,822	0,674	3,64843E-51	5	
Pou2f2	2,18463E-55	0,56557	0,438	0,225	3,66822E-51	5	
Nfat5	6,91797E-55	0,56748	0,721	0,561	1,1616E-50	5	

Hmbox1	9,84773E-55	0,54294	0,69	0,519	1,65353E-50	5	
Adam19	1,14709E-54	0,56212	0,573	0,365	1,92608E-50	5	
Srcin1	1,41138E-54	0,53598	0,756	0,595	2,36984E-50	5	
Smg6	1,89212E-54	0,59496	0,662	0,504	3,17707E-50	5	
Fem1b	2,68432E-54	0,61355	0,74	0,603	4,50725E-50	5	
Arhgef12	3,075E-54	0,54491	0,769	0,623	5,16323E-50	5	
Gls	4,47398E-54	0,51645	0,819	0,688	7,51226E-50	5	
Srrm2	6,16896E-54	0,42809	0,99	0,967	1,03583E-49	5	
Tnrc6b	6,34618E-54	0,54713	0,705	0,562	1,06559E-49	5	
Bptf	5,33975E-53	0,47685	0,899	0,771	8,96598E-49	5	
Arl2bp	6,82779E-53	0,48776	0,836	0,707	1,14645E-48	5	
Satb2	1,43985E-52	0,54857	0,596	0,394	2,41765E-48	5	
Arfgef1	1,67446E-52	0,49002	0,821	0,688	2,81159E-48	5	
Lrrtm4	3,19073E-52	0,32635	0,298	0,114	5,35755E-48	5	
Ppp3r1	4,31675E-52	0,52041	0,87	0,761	7,24826E-48	5	
Wbscr17	1,51888E-51	0,41326	0,438	0,215	2,55035E-47	5	
Setd7	2,88671E-51	0,58145	0,611	0,44	4,84708E-47	5	
Dio2	3,89375E-51	0,43406	0,314	0,128	6,53799E-47	5	
Tmem28	5,61567E-51	0,31619	0,262	0,093	9,42928E-47	5	
Csnk1e	9,83036E-51	0,49272	0,844	0,727	1,65062E-46	5	
Nptn	1,62611E-50	0,43136	0,982	0,91	2,7304E-46	5	
Rb1cc1	2,05831E-50	0,46564	0,862	0,753	3,4561E-46	5	
Unc5c	3,00203E-50	0,57297	0,585	0,402	5,0407E-46	5	
Nt5dc3	3,01788E-50	0,49567	0,832	0,69	5,06732E-46	5	
Magi1	4,9703E-50	0,55217	0,57	0,38	8,34562E-46	5	
Ncor1	9,89841E-50	0,48742	0,876	0,765	1,66204E-45	5	
Rab3c	1,67455E-49	0,32311	0,999	0,995	2,81174E-45	5	
Psap	1,70224E-49	0,34881	1	0,999	2,85822E-45	5	
Rasd2	1,95045E-49	0,42917	0,475	0,249	3,27499E-45	5	
Hs2st1	2,16333E-49	0,5387	0,593	0,412	3,63245E-45	5	
Ctdspl	2,59687E-49	0,50341	0,475	0,274	4,3604E-45	5	
Galntl6	4,20196E-49	0,32874	0,286	0,112	7,05551E-45	5	
Zmynd8	4,74851E-49	0,48565	0,874	0,77	7,97322E-45	5	
Mirg	7,40686E-49	0,56415	0,633	0,469	1,24369E-44	5	
Soga3	7,98339E-49	0,46514	0,936	0,854	1,34049E-44	5	
Plppr5	8,79215E-49	0,50951	0,911	0,793	1,47629E-44	5	
Traf3	1,18923E-48	0,51541	0,531	0,332	1,99684E-44	5	
Epb41l4b	1,35841E-48	0,46904	0,419	0,221	2,2809E-44	5	
Fndc3b	2,08473E-48	0,53634	0,528	0,339	3,50047E-44	5	
Pum2	2,45128E-48	0,53118	0,702	0,566	4,11594E-44	5	
St3gal6	2,95022E-48	0,42228	0,44	0,229	4,95371E-44	5	
Pank3	3,43035E-48	0,53694	0,726	0,604	5,7599E-44	5	
Cxxc4	4,39372E-48	0,49964	0,734	0,604	7,3775E-44	5	
Cpeb4	4,76285E-48	0,51735	0,712	0,572	7,99731E-44	5	
Syngr3	4,93932E-48	0,43994	0,93	0,813	8,29361E-44	5	
Zbtb7a	9,69633E-48	0,53192	0,732	0,593	1,62811E-43	5	
Pabpn1	9,87676E-48	0,54006	0,652	0,508	1,65841E-43	5	
Adamts5	1,04952E-47	0,56942	0,467	0,261	1,76225E-43	5	

Gigyf1	1,46595E-47	0,5653	0,598	0,427	2,46147E-43	5	
Gnb1	2,02343E-47	0,37496	0,999	0,989	3,39755E-43	5	
Map4	3,79057E-47	0,37244	0,995	0,974	6,36475E-43	5	
Sulf2	6,31771E-47	0,37315	0,479	0,248	1,06081E-42	5	
Ptpn5	6,43102E-47	0,34697	0,32	0,137	1,07983E-42	5	
Ncald	8,22716E-47	0,44232	0,933	0,795	1,38142E-42	5	
Slit3	1,01591E-46	0,50232	0,562	0,375	1,70582E-42	5	
Thsd4	1,09055E-46	0,3332	0,29	0,117	1,83115E-42	5	
Glg1	1,21348E-46	0,54095	0,753	0,631	2,03755E-42	5	
Gas1	2,49501E-46	0,41683	0,348	0,162	4,18938E-42	5	
Arhgap5	3,71286E-46	0,54728	0,581	0,399	6,23426E-42	5	
Tet2	4,27371E-46	0,51976	0,481	0,285	7,17599E-42	5	
Ptprt	4,56194E-46	0,42171	0,385	0,184	7,65996E-42	5	
Prrc2a	7,69921E-46	0,4272	0,836	0,721	1,29277E-41	5	
Senp6	9,09259E-46	0,42677	0,884	0,796	1,52674E-41	5	
Prmt8	1,02353E-45	0,33883	0,31	0,131	1,71861E-41	5	
Cacng4	1,27656E-45	0,52486	0,58	0,395	2,14347E-41	5	
Cacna1b	1,34094E-45	0,49249	0,786	0,665	2,25158E-41	5	
Rgs7bp	2,35067E-45	0,52565	0,711	0,599	3,94701E-41	5	
Piezo1	2,49459E-45	0,40945	0,412	0,208	4,18866E-41	5	
Gpc6	3,5034E-45	0,57373	0,65	0,497	5,88255E-41	5	
Rc3h2	3,66116E-45	0,51734	0,734	0,612	6,14746E-41	5	
Ubn2	4,37797E-45	0,53075	0,685	0,553	7,35105E-41	5	
Paqr8	5,33943E-45	0,54304	0,639	0,496	8,96543E-41	5	
Sstr1	6,87766E-45	0,53477	0,768	0,628	1,15483E-40	5	
Tmem132c	9,96741E-45	0,4219	0,357	0,172	1,67363E-40	5	
Rab11b	1,02166E-44	0,39465	0,937	0,851	1,71546E-40	5	
Dusp3	1,59975E-44	0,42246	0,863	0,743	2,68614E-40	5	
Lrrc75b	1,99072E-44	0,51635	0,59	0,432	3,34261E-40	5	
Srgap3	2,10542E-44	0,44987	0,788	0,668	3,53522E-40	5	
Arhgef28	2,63543E-44	0,46772	0,848	0,736	4,42516E-40	5	
Stxbp1	3,25158E-44	0,36615	0,989	0,956	5,45972E-40	5	
Gdf10	3,4585E-44	0,42112	0,345	0,161	5,80717E-40	5	
Mbnl2	6,07795E-44	0,41936	0,919	0,815	1,02055E-39	5	
Ppp3cb	1,50546E-43	0,42507	0,813	0,69	2,52782E-39	5	
Mef2a	1,53648E-43	0,46805	0,703	0,58	2,5799E-39	5	
Tox	1,55961E-43	0,38919	0,362	0,174	2,61874E-39	5	
Tia1	2,05508E-43	0,55145	0,667	0,546	3,45068E-39	5	
Fnbp1l	2,32397E-43	0,48981	0,699	0,582	3,90217E-39	5	
Plce1	3,03628E-43	0,54637	0,625	0,465	5,09822E-39	5	
Dock10	3,53813E-43	0,52081	0,668	0,538	5,94088E-39	5	
Dcx	4,83987E-43	0,56065	0,633	0,501	8,12662E-39	5	
Abcc5	5,28473E-43	0,4281	0,83	0,714	8,8736E-39	5	
Pid1	6,93986E-43	0,49373	0,652	0,511	1,16527E-38	5	
Ebf3	6,99199E-43	0,38356	0,319	0,142	1,17402E-38	5	
Rc3h1	7,9612E-43	0,46693	0,767	0,649	1,33677E-38	5	
Bmpr2	8,56816E-43	0,41031	0,862	0,736	1,43868E-38	5	
Grik4	9,35125E-43	0,46901	0,493	0,302	1,57017E-38	5	

Cuedc1	1,14719E-42	0,48727	0,691	0,576	1,92625E-38	5	
Scoc	1,31736E-42	0,39338	0,939	0,86	2,21199E-38	5	
Adgrb2	1,37051E-42	0,4941	0,504	0,32	2,30123E-38	5	
Dgkh	1,79526E-42	0,41493	0,384	0,201	3,01443E-38	5	
Hivep2	2,15513E-42	0,48398	0,767	0,666	3,61868E-38	5	
Bcl2l11	2,25195E-42	0,36459	0,343	0,161	3,78125E-38	5	
Bub3	2,56257E-42	0,47495	0,906	0,821	4,30281E-38	5	
Calm3	3,086E-42	0,35889	0,961	0,876	5,18171E-38	5	
Ssbp2	4,29295E-42	0,39947	0,943	0,86	7,2083E-38	5	
Tns3	5,90799E-42	0,40088	0,343	0,164	9,9201E-38	5	
Eml5	7,11603E-42	0,50368	0,692	0,563	1,19485E-37	5	
Add2	1,03767E-41	0,41596	0,845	0,737	1,74234E-37	5	
Ube3a	1,26618E-41	0,39167	0,959	0,875	2,12604E-37	5	
Raph1	1,5588E-41	0,54859	0,625	0,478	2,61738E-37	5	
Tril	1,56283E-41	0,55619	0,55	0,392	2,62414E-37	5	
Sgsm2	1,92621E-41	0,50565	0,609	0,459	3,2343E-37	5	
Kndc1	1,99341E-41	0,39924	0,452	0,258	3,34714E-37	5	
Ptp4a2	2,10896E-41	0,54943	0,706	0,592	3,54116E-37	5	
March6	2,97158E-41	0,42396	0,819	0,677	4,98958E-37	5	
Ahi1	5,76626E-41	0,29964	1	1	9,68214E-37	5	
Ankrd12	7,29837E-41	0,42692	0,921	0,826	1,22547E-36	5	
Tln2	8,03863E-41	0,38049	0,919	0,801	1,34977E-36	5	
Gatad2b	1,44964E-40	0,48345	0,676	0,548	2,43409E-36	5	
Napg	1,77457E-40	0,40049	0,868	0,756	2,97969E-36	5	
Zdhhc17	2,51071E-40	0,50071	0,696	0,586	4,21573E-36	5	
Gnb4	2,64494E-40	0,4307	0,977	0,911	4,44111E-36	5	
Sez6l	2,86974E-40	0,44969	0,685	0,505	4,81858E-36	5	
Krit1	3,3304E-40	0,50523	0,706	0,612	5,59208E-36	5	
Npy2r	4,27656E-40	0,50948	0,782	0,651	7,18077E-36	5	
Aqp1	4,72007E-40	0,41593	0,392	0,206	7,92548E-36	5	
Dpysl5	7,66686E-40	0,50385	0,649	0,514	1,28734E-35	5	
Cpt1a	7,97853E-40	0,48445	0,627	0,489	1,33967E-35	5	
Vwc2	1,2378E-39	0,36338	0,443	0,241	2,0784E-35	5	
Aatk	1,61781E-39	0,43823	0,736	0,599	2,71647E-35	5	
Eno2	1,77108E-39	0,41434	0,942	0,826	2,97382E-35	5	
Akap11	4,18793E-39	0,35917	0,972	0,903	7,03196E-35	5	
Slc4a4	6,31824E-39	0,44091	0,894	0,803	1,0609E-34	5	
Srsf5	8,97395E-39	0,42008	0,8	0,684	1,50682E-34	5	
Ednrb	9,47345E-39	0,405	0,319	0,151	1,59069E-34	5	
Mapk1	1,09149E-38	0,40883	0,857	0,756	1,83272E-34	5	
Gtf2ird1	1,22884E-38	0,45942	0,641	0,495	2,06335E-34	5	
Pcdh15	1,84714E-38	0,52111	0,656	0,521	3,10153E-34	5	
Pacsin1	2,64115E-38	0,43455	0,77	0,664	4,43476E-34	5	
Unc5a	3,09136E-38	0,41315	0,448	0,265	5,19071E-34	5	
Ret	4,48679E-38	0,35253	0,994	0,965	7,53377E-34	5	
Man2a1	7,08578E-38	0,34298	0,952	0,838	1,18977E-33	5	
Mkln1	8,51811E-38	0,42336	0,8	0,698	1,43028E-33	5	
Fgf13	9,89373E-38	0,29712	0,999	0,992	1,66126E-33	5	

Slc18a3	1,01456E-37	0,27421	0,795	0,502	1,70354E-33	5	
Cspp1	1,047E-37	0,52323	0,638	0,52	1,75802E-33	5	
Mdm4	1,48374E-37	0,4696	0,574	0,424	2,49134E-33	5	
Rufy2	1,54329E-37	0,47044	0,706	0,596	2,59134E-33	5	
Polr2m	1,69364E-37	0,42515	0,882	0,794	2,84379E-33	5	
Dpyd	3,06291E-37	0,43945	0,767	0,656	5,14293E-33	5	
Ncs1	3,62717E-37	0,4603	0,692	0,575	6,09038E-33	5	
Akap9	4,39199E-37	0,41381	0,886	0,793	7,37458E-33	5	
Cadm1	6,1286E-37	0,30619	0,999	0,996	1,02905E-32	5	
Zeb1	6,21163E-37	0,43099	0,758	0,649	1,04299E-32	5	
Ano6	6,85566E-37	0,35811	0,899	0,795	1,15113E-32	5	
Myo5a	7,18349E-37	0,37777	0,946	0,855	1,20618E-32	5	
Rbm20	7,61124E-37	0,31526	0,262	0,114	1,278E-32	5	
Prune2	9,78827E-37	0,39475	0,94	0,824	1,64355E-32	5	
Ube2d3	1,13569E-36	0,39418	0,864	0,752	1,90693E-32	5	
Crmp1	1,50295E-36	0,36323	0,966	0,9	2,52361E-32	5	
Vamp1	1,7518E-36	0,32585	0,942	0,796	2,94145E-32	5	
Chd6	2,30148E-36	0,42365	0,732	0,624	3,86442E-32	5	
Maz	2,52313E-36	0,34674	0,955	0,864	4,23659E-32	5	
Hnrnpa0	2,70483E-36	0,38405	0,921	0,833	4,54168E-32	5	
Cacna1a	2,72567E-36	0,43997	0,697	0,581	4,57668E-32	5	
Klc1	2,94264E-36	0,29531	1	1	4,94098E-32	5	
Trip12	3,10817E-36	0,39446	0,758	0,656	5,21893E-32	5	
Cxxc5	3,32033E-36	0,35571	0,94	0,844	5,57517E-32	5	
Clasp2	3,81517E-36	0,39868	0,86	0,749	6,40605E-32	5	
Nr2c2	3,89247E-36	0,47067	0,615	0,496	6,53585E-32	5	
Flrt2	3,89984E-36	0,31123	0,253	0,108	6,54822E-32	5	
Ank	4,19334E-36	0,39468	0,431	0,257	7,04104E-32	5	
Ube2b	7,17521E-36	0,34383	0,974	0,925	1,20479E-31	5	
Hoxc4	8,05328E-36	0,36889	0,969	0,905	1,35223E-31	5	
Ankrd17	8,14587E-36	0,40854	0,816	0,715	1,36777E-31	5	
AY036118	9,65187E-36	0,38872	0,922	0,844	1,62064E-31	5	
Trib2	1,09516E-35	0,41257	0,851	0,735	1,83888E-31	5	
Ywhaz	1,49857E-35	0,34707	1	0,991	2,51625E-31	5	
Frmd4b	1,78163E-35	0,36553	0,385	0,212	2,99153E-31	5	
Svil	1,94146E-35	0,45657	0,633	0,509	3,2599E-31	5	
Maml3	2,23834E-35	0,48711	0,606	0,485	3,75839E-31	5	
Slco2a1	2,51107E-35	0,35881	0,278	0,127	4,21634E-31	5	
Impact	2,58855E-35	0,40448	0,957	0,888	4,34644E-31	5	
Grb10	2,9797E-35	0,32177	0,323	0,159	5,00321E-31	5	
Fam168a	6,79472E-35	0,48355	0,62	0,511	1,1409E-30	5	
Nhsl2	8,32374E-35	0,42014	0,419	0,248	1,39764E-30	5	
Tspan9	8,87075E-35	0,46376	0,598	0,461	1,48949E-30	5	
Araf	1,29829E-34	0,42799	0,75	0,649	2,17996E-30	5	
Pkd2	2,22161E-34	0,37685	0,39	0,224	3,7303E-30	5	
Hmcn1	2,55066E-34	0,31297	0,262	0,117	4,28282E-30	5	
Tnik	3,93897E-34	0,43118	0,693	0,589	6,61393E-30	5	
Ypel3	5,59626E-34	0,35884	0,964	0,927	9,39667E-30	5	

Klhdc2	6,28117E-34	0,36936	0,847	0,755	1,05467E-29	5	
Chuk	6,34267E-34	0,4292	0,502	0,339	1,065E-29	5	
Abr	8,30988E-34	0,42528	0,708	0,602	1,39531E-29	5	
Ubash3b	8,71894E-34	0,3835	0,418	0,249	1,464E-29	5	
Csde1	1,01439E-33	0,35491	0,877	0,779	1,70327E-29	5	
Ccdc88a	1,19259E-33	0,45475	0,703	0,619	2,00248E-29	5	
Pcbp3	1,29534E-33	0,32339	0,988	0,957	2,175E-29	5	
Snap91	1,51088E-33	0,4174	0,745	0,647	2,53692E-29	5	
Rev3l	1,7201E-33	0,50642	0,543	0,412	2,88822E-29	5	
Jph3	1,75117E-33	0,37808	0,792	0,66	2,94039E-29	5	
Tra2a	3,74015E-33	0,44023	0,726	0,635	6,28009E-29	5	
Sox4	6,04944E-33	0,4311	0,949	0,859	1,01576E-28	5	
Cpsf6	8,201E-33	0,46292	0,581	0,456	1,37703E-28	5	
Scn2a1	8,56182E-33	0,48961	0,591	0,478	1,43762E-28	5	
Rsrp1	1,09275E-32	0,35839	0,978	0,956	1,83484E-28	5	
Vamp2	1,18148E-32	0,30172	0,993	0,978	1,98382E-28	5	
Ppm1a	1,19042E-32	0,35136	0,875	0,768	1,99883E-28	5	
Ubl3	1,26864E-32	0,38996	0,8	0,701	2,13017E-28	5	
2410089E03	1,73005E-32	0,44759	0,626	0,513	2,90492E-28	5	
Apba1	1,73419E-32	0,33776	0,876	0,748	2,91187E-28	5	
Cadm2	1,79854E-32	0,36001	0,344	0,186	3,01993E-28	5	
Msi1	1,94182E-32	0,41242	0,426	0,269	3,2605E-28	5	
Ubqln2	2,35443E-32	0,35308	0,906	0,806	3,95332E-28	5	
Msl1	2,49933E-32	0,42263	0,706	0,602	4,19663E-28	5	
Pam	2,57373E-32	0,2995	1	0,988	4,32155E-28	5	
Setbp1	2,57734E-32	0,27244	0,26	0,119	4,32761E-28	5	
Nos1ap	2,96282E-32	0,37716	0,366	0,205	4,97488E-28	5	
Rin2	3,14248E-32	0,36347	0,384	0,22	5,27654E-28	5	
Cep170b	3,39946E-32	0,43279	0,677	0,578	5,70804E-28	5	
Scn5a	4,63934E-32	0,41304	0,756	0,657	7,78992E-28	5	
Hdgfrp3	4,7909E-32	0,39471	0,8	0,699	8,0444E-28	5	
Azin1	6,44602E-32	0,37068	0,823	0,715	1,08235E-27	5	
Mecp2	6,68403E-32	0,39872	0,714	0,614	1,12232E-27	5	
Clcn3	7,07981E-32	0,42074	0,64	0,532	1,18877E-27	5	
Snx10	9,81973E-32	0,45925	0,682	0,588	1,64883E-27	5	
Erc1	1,16857E-31	0,3608	0,794	0,674	1,96215E-27	5	
Scml4	1,1749E-31	0,33178	0,385	0,22	1,97278E-27	5	
Hdac9	1,37619E-31	0,31689	0,288	0,142	2,31076E-27	5	
Atp8a1	1,54421E-31	0,33097	0,895	0,773	2,59289E-27	5	
Plxna2	1,70724E-31	0,36761	0,347	0,192	2,86663E-27	5	
Zmat3	1,72816E-31	0,41882	0,706	0,597	2,90176E-27	5	
Shank1	1,81233E-31	0,39362	0,438	0,275	3,04308E-27	5	
Sgip1	1,83251E-31	0,33199	0,931	0,84	3,07696E-27	5	
Atf4	2,49793E-31	0,36029	0,951	0,89	4,19427E-27	5	
Zdhhc21	2,78176E-31	0,43765	0,751	0,656	4,67086E-27	5	
Clasp1	2,90723E-31	0,47267	0,649	0,555	4,88154E-27	5	
Wdfy3	3,30359E-31	0,44628	0,638	0,545	5,54706E-27	5	
Lhfpl2	3,39377E-31	0,328	0,656	0,493	5,69849E-27	5	

Arhgef11	3,51784E-31	0,36545	0,832	0,736	5,9068E-27	5	
Smg1	3,64896E-31	0,43691	0,562	0,44	6,12696E-27	5	
Rph3a	3,90158E-31	0,2998	0,834	0,637	6,55114E-27	5	
C2cd5	6,19516E-31	0,44633	0,56	0,428	1,04023E-26	5	
Kif26a	7,75658E-31	0,37341	0,827	0,701	1,30241E-26	5	
Snrk	1,42804E-30	0,39865	0,807	0,701	2,39782E-26	5	
Rcn2	1,46825E-30	0,40683	0,748	0,648	2,46534E-26	5	
Cacna2d1	1,67008E-30	0,31416	0,968	0,915	2,80422E-26	5	
Sv2a	2,19904E-30	0,35026	0,878	0,781	3,69241E-26	5	
Mtpn	2,45639E-30	0,36901	0,852	0,77	4,12453E-26	5	
Ccnl2	2,67628E-30	0,41108	0,554	0,415	4,49374E-26	5	
Cux2	2,89012E-30	0,42019	0,692	0,585	4,8528E-26	5	
Ccnt2	2,91401E-30	0,45544	0,566	0,455	4,89291E-26	5	
Arf1	3,07388E-30	0,32104	0,983	0,951	5,16134E-26	5	
Ddx3x	4,08796E-30	0,34821	0,857	0,725	6,86409E-26	5	
Lynx1	4,95226E-30	0,30778	0,446	0,267	8,31534E-26	5	
D17Wsu92e	6,41269E-30	0,33728	0,822	0,704	1,07675E-25	5	
Zfp280d	6,65455E-30	0,43936	0,55	0,432	1,11736E-25	5	
Helz	6,67095E-30	0,44425	0,563	0,452	1,12012E-25	5	
Atp6v1b2	6,97456E-30	0,32437	0,951	0,86	1,1711E-25	5	
Aff4	7,48656E-30	0,33784	0,958	0,89	1,25707E-25	5	
Sh3glb1	8,20906E-30	0,34649	0,895	0,816	1,37838E-25	5	
BC005561	8,53879E-30	0,44534	0,625	0,511	1,43375E-25	5	
Pbrm1	1,26596E-29	0,366	0,854	0,756	2,12567E-25	5	
Hoxb3	1,49445E-29	0,41272	0,401	0,251	2,50933E-25	5	
Cmip	1,7531E-29	0,3122	0,934	0,85	2,94363E-25	5	
Xkr6	2,61719E-29	0,4173	0,497	0,356	4,39452E-25	5	
Disp2	3,19457E-29	0,34161	0,832	0,724	5,364E-25	5	
Dmkn	3,99657E-29	0,25739	0,564	0,335	6,71064E-25	5	
Leprotl1	5,21154E-29	0,39083	0,744	0,654	8,75069E-25	5	
Nsd1	5,34571E-29	0,3828	0,763	0,684	8,97598E-25	5	
Zranb2	5,57539E-29	0,33789	0,859	0,777	9,36163E-25	5	
Arhgap21	5,58414E-29	0,41571	0,639	0,546	9,37633E-25	5	
Ccdc50	1,03981E-28	0,37625	0,801	0,71	1,74595E-24	5	
Nufip2	1,17129E-28	0,40566	0,679	0,599	1,96671E-24	5	
Ccbe1	1,18003E-28	0,2612	0,324	0,169	1,98139E-24	5	
Dnajc5	1,33914E-28	0,35005	0,826	0,727	2,24854E-24	5	
Ppp1cb	1,36925E-28	0,39132	0,728	0,618	2,29911E-24	5	
Dock3	1,37145E-28	0,40584	0,519	0,387	2,30281E-24	5	
Grb2	1,92814E-28	0,33586	0,864	0,77	3,23754E-24	5	
AI314180	1,93022E-28	0,40506	0,653	0,558	3,24103E-24	5	
Filip1	2,21403E-28	0,35564	0,898	0,814	3,71758E-24	5	
Birc6	2,49851E-28	0,3531	0,751	0,668	4,19525E-24	5	
Plec	3,10353E-28	0,3142	0,947	0,85	5,21114E-24	5	
Kcnc4	3,62398E-28	0,33634	0,421	0,262	6,08503E-24	5	
Tshz1	4,26547E-28	0,38062	0,455	0,31	7,16215E-24	5	
Herc1	4,41425E-28	0,35478	0,822	0,725	7,41197E-24	5	
Stxbp5l	6,08327E-28	0,41528	0,546	0,422	1,02144E-23	5	

Epb41l2	6,1563E-28	0,41162	0,521	0,385	1,0337E-23	5	
Arf3	6,18069E-28	0,3162	0,895	0,772	1,0378E-23	5	
Rnf11	6,25604E-28	0,40315	0,682	0,594	1,05045E-23	5	
Mapk10	6,67312E-28	0,31183	0,96	0,886	1,12048E-23	5	
Larp4b	6,97417E-28	0,40694	0,717	0,625	1,17103E-23	5	
Aak1	7,55039E-28	0,42747	0,549	0,43	1,26779E-23	5	
Pum1	9,81856E-28	0,44193	0,622	0,544	1,64863E-23	5	
Ptbp2	9,8944E-28	0,44515	0,647	0,57	1,66137E-23	5	
Erc2	1,14928E-27	0,43821	0,6	0,506	1,92976E-23	5	
Fubp1	1,22227E-27	0,41378	0,71	0,634	2,05232E-23	5	
Hipk1	1,35509E-27	0,40947	0,649	0,544	2,27533E-23	5	
Tom1l2	1,63626E-27	0,37455	0,714	0,604	2,74745E-23	5	
Lrrtm1	1,68196E-27	0,3434	0,455	0,294	2,82418E-23	5	
Mtmr6	1,83027E-27	0,33895	0,813	0,732	3,07321E-23	5	
Gm21092	2,06072E-27	0,40603	0,56	0,433	3,46016E-23	5	
Slc38a1	2,20401E-27	0,39247	0,626	0,527	3,70075E-23	5	
Rprm	2,51468E-27	0,33739	0,448	0,275	4,2224E-23	5	
Son	2,73301E-27	0,28806	0,99	0,98	4,589E-23	5	
Emb	2,93303E-27	0,2817	0,386	0,223	4,92485E-23	5	
Brwd1	3,09983E-27	0,41857	0,568	0,452	5,20493E-23	5	
Syt2	3,21366E-27	0,33094	0,768	0,66	5,39606E-23	5	
Scn8a	3,52444E-27	0,34747	0,381	0,235	5,91789E-23	5	
Pcmtd1	4,08856E-27	0,36197	0,727	0,641	6,8651E-23	5	
Pirt	4,7552E-27	0,32702	0,999	0,983	7,98446E-23	5	
Rapgef6	5,33287E-27	0,40981	0,634	0,538	8,95442E-23	5	
Ip6k1	5,61836E-27	0,32138	0,819	0,697	9,43378E-23	5	
Xpr1	7,41238E-27	0,42159	0,55	0,439	1,24461E-22	5	
Pja2	7,64646E-27	0,35779	0,917	0,82	1,28392E-22	5	
Cbx5	8,515E-27	0,3409	0,815	0,721	1,42975E-22	5	
Tspan7	8,57809E-27	0,3113	0,909	0,794	1,44035E-22	5	
Spata13	1,05007E-26	0,38937	0,401	0,256	1,76318E-22	5	
Frrs1l	1,18261E-26	0,41631	0,471	0,339	1,98572E-22	5	
Dynll2	1,40409E-26	0,29616	0,995	0,972	2,3576E-22	5	
Zcchc14	1,46696E-26	0,43018	0,473	0,345	2,46317E-22	5	
Ep300	1,59428E-26	0,37262	0,567	0,446	2,67696E-22	5	
Sult4a1	2,10996E-26	0,28739	0,987	0,937	3,54283E-22	5	
Hacd3	2,70018E-26	0,41911	0,614	0,519	4,53388E-22	5	
Unc79	3,15302E-26	0,41253	0,519	0,39	5,29424E-22	5	
Mllt6	3,28631E-26	0,4073	0,525	0,407	5,51804E-22	5	
Sumo3	3,85934E-26	0,34975	0,807	0,71	6,48022E-22	5	
Ndst1	4,69337E-26	0,30006	0,329	0,188	7,88064E-22	5	
Rbfox3	4,97371E-26	0,36867	0,439	0,285	8,35136E-22	5	
Gm16586	5,66746E-26	0,31084	0,307	0,172	9,51624E-22	5	
Kdm6b	5,83605E-26	0,38985	0,477	0,344	9,79931E-22	5	
Gm38393	9,94916E-26	0,30859	0,321	0,183	1,67056E-21	5	
Tmcc3	1,25019E-25	0,4401	0,575	0,482	2,0992E-21	5	
Hdac7	1,44056E-25	0,40012	0,515	0,39	2,41884E-21	5	
Klf9	1,48099E-25	0,38626	0,436	0,302	2,48674E-21	5	

Camk2d	1,96804E-25	0,33634	0,863	0,763	3,30453E-21	5	
Baz1b	2,15636E-25	0,39304	0,661	0,592	3,62075E-21	5	
Mdga2	2,37282E-25	0,29871	0,307	0,172	3,9842E-21	5	
Adrbk1	2,53633E-25	0,32576	0,776	0,684	4,25876E-21	5	
Smug1	2,60624E-25	0,29315	0,297	0,164	4,37613E-21	5	
Gabbr1	2,69212E-25	0,31293	0,917	0,86	4,52034E-21	5	
Epn2	2,70084E-25	0,37609	0,697	0,621	4,53498E-21	5	
Cdkn1b	3,10797E-25	0,34314	0,793	0,696	5,2186E-21	5	
Hnrnpa3	4,07098E-25	0,3126	0,875	0,789	6,83559E-21	5	
Ugcg	4,39029E-25	0,26612	0,901	0,775	7,37174E-21	5	
Capza2	4,41561E-25	0,31944	0,883	0,771	7,41425E-21	5	
Arhgef1	6,10149E-25	0,38806	0,643	0,568	1,0245E-20	5	
Epm2aip1	6,73375E-25	0,36407	0,742	0,65	1,13066E-20	5	
Cadps	6,75331E-25	0,31499	0,903	0,824	1,13395E-20	5	
Fbxl16	8,59573E-25	0,32293	0,735	0,638	1,44331E-20	5	
Rgs9	9,25536E-25	0,30474	0,851	0,748	1,55407E-20	5	
Tollip	1,01598E-24	0,36721	0,664	0,579	1,70594E-20	5	
Dbt	1,1637E-24	0,32589	0,366	0,224	1,95396E-20	5	
Zfp148	1,22172E-24	0,39514	0,534	0,423	2,05138E-20	5	
Atp6ap2	1,2577E-24	0,32061	0,845	0,742	2,11181E-20	5	
Txnip	1,25809E-24	0,40489	0,407	0,265	2,11245E-20	5	
Arpc5	1,39984E-24	0,31098	0,953	0,886	2,35047E-20	5	
Chp1	1,55655E-24	0,33965	0,775	0,672	2,6136E-20	5	
Flot2	1,5962E-24	0,27709	0,98	0,919	2,68018E-20	5	
Rap1gap2	2,07358E-24	0,35938	0,801	0,712	3,48176E-20	5	
Cdipt	2,07403E-24	0,33084	0,834	0,718	3,48251E-20	5	
Mff	2,10329E-24	0,36457	0,718	0,65	3,53164E-20	5	
Nudt3	2,20672E-24	0,31537	0,83	0,725	3,70531E-20	5	
Zfp618	2,35012E-24	0,35989	0,457	0,324	3,94609E-20	5	
Fsd1l	2,67222E-24	0,37047	0,677	0,603	4,48692E-20	5	
Usf2	3,1422E-24	0,36735	0,682	0,593	5,27608E-20	5	
Git2	3,28614E-24	0,41147	0,54	0,44	5,51776E-20	5	
Dner	3,47405E-24	0,38212	0,644	0,552	5,83327E-20	5	
Camsap2	5,21469E-24	0,34623	0,742	0,651	8,75598E-20	5	
Prickle2	5,54951E-24	0,26204	0,3	0,167	9,31819E-20	5	
Prrc2c	6,39696E-24	0,28682	0,977	0,931	1,07411E-19	5	
Mark2	9,16018E-24	0,34555	0,641	0,556	1,53809E-19	5	
Mbd6	1,00541E-23	0,34369	0,438	0,303	1,68819E-19	5	
Twsg1	1,03511E-23	0,42146	0,56	0,457	1,73805E-19	5	
Picalm	1,07582E-23	0,3678	0,687	0,604	1,8064E-19	5	
Kmt2d	1,10779E-23	0,38747	0,523	0,4	1,86009E-19	5	
Apc	1,28343E-23	0,30015	0,841	0,75	2,155E-19	5	
Phf14	1,52561E-23	0,38335	0,646	0,573	2,56166E-19	5	
Hdgf	1,72406E-23	0,31199	0,789	0,708	2,89487E-19	5	
Ctgf	1,78141E-23	0,31269	0,329	0,191	2,99117E-19	5	
Zfp451	2,18058E-23	0,3596	0,604	0,511	3,66141E-19	5	
Cachd1	2,18388E-23	0,31341	0,319	0,186	3,66695E-19	5	
Arid2	2,26939E-23	0,39983	0,526	0,414	3,81053E-19	5	

Zdhhc8	2,73439E-23	0,35046	0,658	0,556	4,59131E-19	5	
Ntm	2,96991E-23	0,3565	0,279	0,15	4,98677E-19	5	
Pcm1	3,30179E-23	0,30515	0,887	0,801	5,54403E-19	5	
Ctnna1	3,56614E-23	0,35894	0,617	0,499	5,98791E-19	5	
Dip2b	3,70566E-23	0,36571	0,656	0,585	6,22217E-19	5	
Rnf44	4,78295E-23	0,38973	0,521	0,413	8,03105E-19	5	
Irf2bpl	5,04849E-23	0,37837	0,504	0,384	8,47692E-19	5	
Med13l	5,75198E-23	0,29701	0,379	0,242	9,65814E-19	5	
Atp2b1	5,76863E-23	0,28976	0,835	0,746	9,6861E-19	5	
March4	6,05332E-23	0,35517	0,56	0,446	1,01641E-18	5	
Crebbp	9,18429E-23	0,38751	0,616	0,559	1,54213E-18	5	
Atrx	1,12684E-22	0,32406	0,911	0,852	1,89208E-18	5	
Tmcc1	1,24112E-22	0,40508	0,548	0,452	2,08396E-18	5	
Fam178a	1,27002E-22	0,36133	0,516	0,399	2,1325E-18	5	
Vamp4	1,28393E-22	0,33838	0,768	0,675	2,15585E-18	5	
Atp6v0a1	1,69147E-22	0,27846	0,939	0,872	2,84014E-18	5	
Pde4b	1,71234E-22	0,40997	0,507	0,404	2,87519E-18	5	
Braf	2,14761E-22	0,39189	0,596	0,523	3,60605E-18	5	
Spred2	2,28965E-22	0,36867	0,437	0,312	3,84456E-18	5	
Ankrd10	2,33625E-22	0,38903	0,543	0,438	3,92281E-18	5	
Csnk1d	2,84323E-22	0,32737	0,744	0,64	4,77407E-18	5	
Srsf2	3,2722E-22	0,33601	0,729	0,665	5,49435E-18	5	
Gnaz	3,89988E-22	0,31737	0,715	0,625	6,54829E-18	5	
A830039N20	4,79648E-22	0,37275	0,48	0,364	8,05377E-18	5	
R3hdm2	5,10792E-22	0,32766	0,746	0,685	8,57671E-18	5	
Mllt10	5,1823E-22	0,32443	0,408	0,282	8,7016E-18	5	
Tac1	5,47696E-22	0,29391	0,277	0,152	9,19637E-18	5	
Kcnq2	6,97174E-22	0,32465	0,722	0,632	1,17062E-17	5	
Tmem158	7,01104E-22	0,30074	0,826	0,711	1,17722E-17	5	
Trp53inp2	7,33569E-22	0,39922	0,611	0,529	1,23174E-17	5	
Rnpc3	8,66038E-22	0,37309	0,444	0,327	1,45416E-17	5	
Nf1	8,72415E-22	0,36812	0,598	0,511	1,46487E-17	5	
B830012L14I	9,96613E-22	0,29464	0,314	0,189	1,67341E-17	5	
Ptprr	1,04825E-21	0,29736	0,868	0,8	1,76011E-17	5	
Ctbp1	1,30953E-21	0,27707	0,947	0,872	2,19883E-17	5	
Zc3h7a	1,63613E-21	0,38564	0,499	0,39	2,74722E-17	5	
Arf5	1,87814E-21	0,26679	0,969	0,941	3,15358E-17	5	
4932438A13	2,48104E-21	0,32532	0,736	0,677	4,16592E-17	5	
Slc36a4	2,51555E-21	0,38726	0,484	0,365	4,22387E-17	5	
Atxn2	2,74265E-21	0,36854	0,621	0,57	4,60518E-17	5	
Magi3	2,79303E-21	0,32742	0,451	0,319	4,68978E-17	5	
Plcb4	3,35626E-21	0,25917	0,99	0,949	5,63549E-17	5	
Tspan2	3,59206E-21	0,36162	0,61	0,534	6,03143E-17	5	
Lpgat1	3,9243E-21	0,40946	0,549	0,479	6,5893E-17	5	
B4galt5	4,0324E-21	0,26916	0,309	0,185	6,7708E-17	5	
Cacnb1	4,94724E-21	0,30488	0,686	0,596	8,30691E-17	5	
Mtus1	4,99879E-21	0,38026	0,619	0,556	8,39346E-17	5	
Dopey1	6,30658E-21	0,36643	0,564	0,48	1,05894E-16	5	

Tet3	6,71713E-21	0,36053	0,492	0,385	1,12787E-16	5	
Utrn	6,71882E-21	0,31202	0,775	0,689	1,12816E-16	5	
Rock1	6,88333E-21	0,3765	0,522	0,428	1,15578E-16	5	
Mat2a	8,12464E-21	0,30218	0,753	0,665	1,36421E-16	5	
Marcksl1	9,13497E-21	0,35573	0,71	0,651	1,53385E-16	5	
Sept11	9,44284E-21	0,39585	0,522	0,429	1,58555E-16	5	
Mga	9,85016E-21	0,37151	0,582	0,506	1,65394E-16	5	
Spred1	1,30682E-20	0,36502	0,432	0,317	2,19428E-16	5	
Ldlr	1,32621E-20	0,28342	0,767	0,64	2,22684E-16	5	
Tanc2	1,38665E-20	0,37982	0,542	0,452	2,32833E-16	5	
Tmeff1	1,46397E-20	0,37298	0,586	0,5	2,45816E-16	5	
Acvr1b	1,55444E-20	0,31099	0,389	0,264	2,61006E-16	5	
Rmst	1,83285E-20	0,38884	0,374	0,25	3,07754E-16	5	
Atp6v1a	2,06947E-20	0,36107	0,73	0,657	3,47484E-16	5	
Nrip1	2,18392E-20	0,25326	0,948	0,867	3,66702E-16	5	
Ccnl1	2,35349E-20	0,36889	0,523	0,429	3,95175E-16	5	
Kif3a	2,62377E-20	0,32712	0,684	0,62	4,40557E-16	5	
Arl6ip1	2,6244E-20	0,29428	0,909	0,862	4,40663E-16	5	
Ankrd11	2,96235E-20	0,26392	0,937	0,868	4,97409E-16	5	
Slitrk3	3,11299E-20	0,30536	0,394	0,262	5,22702E-16	5	
Celf6	3,40091E-20	0,26029	0,977	0,948	5,71048E-16	5	
Whsc1l1	3,95375E-20	0,36296	0,661	0,605	6,63875E-16	5	
Guf1	4,25413E-20	0,28628	0,351	0,226	7,14311E-16	5	
Fam63b	4,51465E-20	0,34897	0,668	0,604	7,58055E-16	5	
Mef2d	4,64686E-20	0,35597	0,519	0,415	7,80254E-16	5	
Scai	5,80613E-20	0,33736	0,424	0,309	9,74907E-16	5	
Bace1	6,40695E-20	0,32226	0,67	0,594	1,07579E-15	5	
Ulk1	6,6927E-20	0,34739	0,529	0,425	1,12377E-15	5	
Impad1	7,73064E-20	0,40975	0,594	0,528	1,29805E-15	5	
Nbeal1	7,98393E-20	0,35716	0,613	0,548	1,34058E-15	5	
Creg2	8,30917E-20	0,30897	0,389	0,263	1,39519E-15	5	
Ski	1,3726E-19	0,31916	0,694	0,622	2,30473E-15	5	
Pcnp	1,42593E-19	0,35751	0,621	0,559	2,39428E-15	5	
Man2a2	1,53382E-19	0,37301	0,526	0,439	2,57544E-15	5	
Rac1	1,70546E-19	0,27341	0,929	0,857	2,86364E-15	5	
Kcnt1	1,77469E-19	0,35635	0,445	0,336	2,97988E-15	5	
Apba2	2,97602E-19	0,29312	0,817	0,712	4,99704E-15	5	
Slc38a2	3,12853E-19	0,36523	0,644	0,593	5,25311E-15	5	
Klhl24	3,69592E-19	0,33032	0,448	0,337	6,20582E-15	5	
Nrcam	4,14745E-19	0,33201	0,374	0,257	6,96398E-15	5	
Nek7	4,38297E-19	0,29943	0,813	0,742	7,35945E-15	5	
Setd5	5,6417E-19	0,38283	0,617	0,57	9,47298E-15	5	
Zzef1	6,70575E-19	0,33148	0,513	0,41	1,12596E-14	5	
Fam110b	9,13053E-19	0,27731	0,348	0,226	1,53311E-14	5	
G3bp2	1,11009E-18	0,25467	0,88	0,801	1,86396E-14	5	
Tacc1	1,53276E-18	0,36204	0,534	0,455	2,57366E-14	5	
Clcn4	1,61032E-18	0,32339	0,722	0,656	2,70389E-14	5	
Scn2b	1,73986E-18	0,39886	0,478	0,381	2,9214E-14	5	

Slc25a36	1,79908E-18	0,36379	0,481	0,388	3,02083E-14	5	
Hdac4	1,98041E-18	0,34148	0,542	0,465	3,3253E-14	5	
Cbl	2,20846E-18	0,33916	0,599	0,523	3,70823E-14	5	
Cntln	2,25747E-18	0,32564	0,433	0,317	3,79052E-14	5	
Fam134b	2,81807E-18	0,32299	0,632	0,574	4,73183E-14	5	
Mgat4a	2,92106E-18	0,30565	0,421	0,305	4,90476E-14	5	
Reep5	3,53884E-18	0,25409	0,995	0,975	5,94206E-14	5	
Cyb5b	3,56356E-18	0,33691	0,637	0,575	5,98358E-14	5	
Pten	4,03455E-18	0,29001	0,692	0,628	6,77441E-14	5	
Spaca6	4,16881E-18	0,28266	0,341	0,224	6,99985E-14	5	
Phc3	4,49834E-18	0,32474	0,409	0,301	7,55316E-14	5	
Scn7a	4,5379E-18	0,33495	0,467	0,361	7,61959E-14	5	
Arid1a	4,55739E-18	0,33531	0,581	0,511	7,65232E-14	5	
Arrb1	4,87242E-18	0,35991	0,544	0,467	8,18129E-14	5	
B230219D22	6,22191E-18	0,28814	0,858	0,804	1,04472E-13	5	
Kidins220	6,84209E-18	0,28869	0,767	0,689	1,14886E-13	5	
Tnk2	6,87002E-18	0,3116	0,628	0,545	1,15354E-13	5	
R3hdm1	7,91957E-18	0,26648	0,924	0,854	1,32978E-13	5	
Mapk8	8,25905E-18	0,32376	0,514	0,419	1,38678E-13	5	
Gatsl2	8,55937E-18	0,32954	0,461	0,36	1,4372E-13	5	
Ewsr1	8,59139E-18	0,33516	0,655	0,613	1,44258E-13	5	
Fmnl2	8,73885E-18	0,32685	0,542	0,45	1,46734E-13	5	
Cyfip2	8,94155E-18	0,29165	0,728	0,657	1,50138E-13	5	
Zyg11b	9,10727E-18	0,36813	0,58	0,526	1,5292E-13	5	
Osbpl3	1,0381E-17	0,40191	0,535	0,462	1,74307E-13	5	
Zbtb44	1,27972E-17	0,34813	0,531	0,448	2,14878E-13	5	
Taf1d	1,37996E-17	0,3144	0,734	0,692	2,31709E-13	5	
Gm15800	1,51908E-17	0,25241	0,792	0,698	2,55069E-13	5	
Synm	1,55349E-17	0,38449	0,543	0,471	2,60846E-13	5	
Fam168b	2,26855E-17	0,31703	0,627	0,564	3,80913E-13	5	
Fam49a	2,46802E-17	0,3353	0,528	0,435	4,14405E-13	5	
Itpripl2	2,48937E-17	0,35001	0,481	0,385	4,17991E-13	5	
Esyt2	2,74424E-17	0,34056	0,534	0,458	4,60786E-13	5	
Spin1	2,75521E-17	0,36776	0,6	0,541	4,62627E-13	5	
Syne2	2,76431E-17	0,28159	0,321	0,207	4,64156E-13	5	
Ylpm1	3,70119E-17	0,36144	0,556	0,49	6,21467E-13	5	
Tead1	5,3324E-17	0,27808	0,391	0,276	8,95363E-13	5	
Grina	5,9993E-17	0,25848	0,976	0,917	1,00734E-12	5	
2700081O15	6,33262E-17	0,33175	0,535	0,454	1,06331E-12	5	
Tra2b	6,85404E-17	0,30673	0,645	0,599	1,15086E-12	5	
Hook3	9,13179E-17	0,33379	0,592	0,526	1,53332E-12	5	
Uggt2	9,4434E-17	0,31157	0,392	0,284	1,58564E-12	5	
Hoxa3	1,00118E-16	0,30896	0,426	0,322	1,68108E-12	5	
Spag9	1,55337E-16	0,25116	0,942	0,884	2,60826E-12	5	
Epha5	1,97255E-16	0,36008	0,329	0,22	3,31211E-12	5	
Map6	2,00048E-16	0,28253	0,782	0,734	3,359E-12	5	
Ept1	2,09188E-16	0,32915	0,527	0,452	3,51247E-12	5	
Kat6b	2,24821E-16	0,34472	0,467	0,381	3,77498E-12	5	

Cd99l2	2,41183E-16	0,3493	0,552	0,494	4,04971E-12	5	
Maged1	2,44963E-16	0,26842	0,99	0,962	4,11317E-12	5	
Adam23	2,76228E-16	0,33111	0,44	0,344	4,63815E-12	5	
Tcf20	2,92134E-16	0,30588	0,531	0,445	4,90522E-12	5	
Chka	3,10462E-16	0,36688	0,549	0,487	5,21297E-12	5	
Pcbp2	3,22633E-16	0,29196	0,877	0,827	5,41733E-12	5	
Sema4d	3,85816E-16	0,32582	0,55	0,479	6,47824E-12	5	
Inpp5f	3,90834E-16	0,26519	0,786	0,714	6,5625E-12	5	
Lonrf2	4,10616E-16	0,35395	0,552	0,481	6,89466E-12	5	
Eif4g3	4,13956E-16	0,2568	0,889	0,84	6,95074E-12	5	
Hivep1	4,54998E-16	0,29306	0,479	0,379	7,63988E-12	5	
Lrrn2	4,76674E-16	0,29649	0,616	0,536	8,00384E-12	5	
Trio	4,9983E-16	0,30621	0,434	0,336	8,39264E-12	5	
Hmgcr	5,43985E-16	0,25717	0,934	0,851	9,13404E-12	5	
Acvr2a	5,84615E-16	0,26083	0,294	0,191	9,81627E-12	5	
9330182L06R	5,94921E-16	0,34884	0,508	0,431	9,98932E-12	5	
Lims1	6,30088E-16	0,32821	0,639	0,59	1,05798E-11	5	
Mvb12b	7,02841E-16	0,33021	0,483	0,398	1,18014E-11	5	
Rcor3	7,39701E-16	0,35819	0,52	0,442	1,24203E-11	5	
Sbf2	7,74628E-16	0,3078	0,455	0,356	1,30068E-11	5	
Myadm	7,97438E-16	0,25372	0,912	0,831	1,33898E-11	5	
Nsf	9,17906E-16	0,33066	0,626	0,581	1,54126E-11	5	
Cep170	9,45553E-16	0,32743	0,656	0,597	1,58768E-11	5	
Rnf157	1,00963E-15	0,32932	0,509	0,434	1,69527E-11	5	
Atxn7l3	1,16343E-15	0,27532	0,585	0,513	1,95351E-11	5	
Sv2c	1,18217E-15	0,34611	0,587	0,509	1,98498E-11	5	
Vezt	1,18537E-15	0,31911	0,568	0,51	1,99035E-11	5	
Uhrf2	1,21811E-15	0,31239	0,486	0,402	2,04533E-11	5	
Scamp1	1,24302E-15	0,27329	0,817	0,726	2,08715E-11	5	
Tardbp	1,27061E-15	0,31753	0,671	0,625	2,13348E-11	5	
Tmed8	1,38008E-15	0,30731	0,395	0,295	2,31729E-11	5	
Selt	1,38365E-15	0,33086	0,605	0,562	2,32329E-11	5	
Strbp	1,39102E-15	0,25059	0,799	0,724	2,33566E-11	5	
A230050P20	1,41084E-15	0,27283	0,389	0,284	2,36894E-11	5	
Lrp8	1,48502E-15	0,25918	0,374	0,266	2,4935E-11	5	
Mib1	1,52321E-15	0,34165	0,517	0,441	2,55763E-11	5	
Leng8	1,55859E-15	0,32688	0,381	0,284	2,61704E-11	5	
Rabgap1l	1,58981E-15	0,29723	0,373	0,274	2,66946E-11	5	
Tmem245	1,69834E-15	0,33789	0,498	0,416	2,85168E-11	5	
Xrn1	1,72874E-15	0,33182	0,498	0,418	2,90272E-11	5	
Smc5	1,75009E-15	0,32494	0,454	0,365	2,93858E-11	5	
Add3	2,06984E-15	0,2581	0,717	0,645	3,47547E-11	5	
Pik3ca	2,21841E-15	0,31924	0,604	0,552	3,72494E-11	5	
Wdr13	2,88651E-15	0,31062	0,473	0,383	4,84674E-11	5	
Map4k4	2,90357E-15	0,31146	0,699	0,665	4,87539E-11	5	
Chn1	3,05123E-15	0,2707	0,763	0,708	5,12332E-11	5	
D5Ertd579e	3,36201E-15	0,33065	0,491	0,419	5,64515E-11	5	
Trim35	4,50428E-15	0,26629	0,726	0,675	7,56313E-11	5	

Rif1	5,19811E-15	0,31275	0,449	0,358	8,72815E-11	5	
Ivns1abp	6,19495E-15	0,3206	0,43	0,34	1,04019E-10	5	
Zbtb4	6,37379E-15	0,31699	0,532	0,466	1,07022E-10	5	
Ppfibp2	1,05466E-14	0,29747	0,662	0,613	1,77088E-10	5	
Trpm7	1,20497E-14	0,28763	0,463	0,376	2,02326E-10	5	
Oprl1	1,35186E-14	0,26703	0,378	0,272	2,26991E-10	5	
Slc25a51	1,4146E-14	0,29797	0,637	0,589	2,37526E-10	5	
Arhgap20	1,52538E-14	0,27673	0,401	0,298	2,56127E-10	5	
Cacna2d2	1,82102E-14	0,25866	0,353	0,253	3,05767E-10	5	
Ttc7b	2,32414E-14	0,31907	0,431	0,34	3,90247E-10	5	
Mgea5	2,41514E-14	0,31105	0,61	0,567	4,05526E-10	5	
Flna	2,86273E-14	0,26813	0,347	0,247	4,8068E-10	5	
Map9	2,90504E-14	0,25234	0,799	0,729	4,87785E-10	5	
Coro2a	3,07892E-14	0,31588	0,588	0,555	5,16981E-10	5	
Bcr	3,10329E-14	0,30633	0,463	0,381	5,21074E-10	5	
Usp31	3,35784E-14	0,30794	0,486	0,413	5,63815E-10	5	
Dgki	3,38078E-14	0,27904	0,391	0,293	5,67667E-10	5	
Lhfpl4	3,58706E-14	0,30934	0,578	0,532	6,02304E-10	5	
Tbl1xr1	3,86373E-14	0,31654	0,46	0,378	6,48759E-10	5	
Rnf10	4,27751E-14	0,25066	0,697	0,658	7,18236E-10	5	
Appbp2	4,40358E-14	0,35387	0,489	0,42	7,39405E-10	5	
Cited2	4,91003E-14	0,26075	0,93	0,87	8,24443E-10	5	
Cntn2	5,78199E-14	0,2628	0,351	0,251	9,70854E-10	5	
Nipbl	6,03503E-14	0,33855	0,526	0,476	1,01334E-09	5	
Arhgef7	6,20586E-14	0,30522	0,456	0,372	1,04203E-09	5	
Klf3	7,86856E-14	0,28324	0,391	0,295	1,32121E-09	5	
Trim25	9,263E-14	0,31675	0,54	0,48	1,55535E-09	5	
Rai1	1,08502E-13	0,30803	0,472	0,403	1,82185E-09	5	
Fam19a1	1,30599E-13	0,30587	0,532	0,453	2,19289E-09	5	
Tsc1	1,40427E-13	0,28533	0,381	0,286	2,35791E-09	5	
Camkk2	1,41554E-13	0,31503	0,515	0,448	2,37684E-09	5	
Gcnt2	1,4566E-13	0,26691	0,691	0,64	2,44577E-09	5	
Tapbp	1,52845E-13	0,25262	0,806	0,722	2,56642E-09	5	
Col4a3bp	1,53046E-13	0,32073	0,496	0,433	2,5698E-09	5	
Actr2	1,54875E-13	0,28925	0,64	0,611	2,6005E-09	5	
Dlgap2	1,57359E-13	0,31483	0,424	0,339	2,64221E-09	5	
Adam9	1,70307E-13	0,26808	0,378	0,281	2,85963E-09	5	
Rcan3	1,76351E-13	0,30482	0,602	0,56	2,96112E-09	5	
Sbno1	2,24165E-13	0,32854	0,573	0,537	3,76396E-09	5	
Smim13	2,27494E-13	0,33042	0,549	0,512	3,81984E-09	5	
Wdr37	2,46043E-13	0,31733	0,478	0,402	4,13131E-09	5	
S100pbp	2,97803E-13	0,32132	0,478	0,413	5,00041E-09	5	
Atf7ip	3,02602E-13	0,30355	0,504	0,439	5,081E-09	5	
Hnrnpl	3,17673E-13	0,304	0,643	0,599	5,33405E-09	5	
Gdap1	3,83284E-13	0,30248	0,649	0,621	6,43572E-09	5	
Khdrbs1	3,99383E-13	0,29225	0,657	0,626	6,70604E-09	5	
Hjurp	4,04937E-13	0,29253	0,467	0,394	6,7993E-09	5	
Cyth1	7,67281E-13	0,28288	0,469	0,394	1,28834E-08	5	

March7	7,69005E-13	0,32045	0,497	0,448	1,29124E-08	5	
Begain	7,75933E-13	0,26639	0,602	0,529	1,30287E-08	5	
Robo2	8,10645E-13	0,27101	0,433	0,338	1,36115E-08	5	
Far1	8,24509E-13	0,2875	0,611	0,569	1,38443E-08	5	
Cry2	8,40788E-13	0,27324	0,425	0,337	1,41177E-08	5	
Vezf1	8,61522E-13	0,28672	0,581	0,539	1,44658E-08	5	
Usp32	9,38299E-13	0,29867	0,514	0,459	1,5755E-08	5	
Ldoc1l	1,195E-12	0,32404	0,546	0,492	2,00652E-08	5	
Hoxb2	1,20582E-12	0,26361	0,623	0,575	2,02469E-08	5	
H2-K1	1,35387E-12	0,27472	0,789	0,723	2,27328E-08	5	
Hnrnpu	1,44829E-12	0,26188	0,646	0,606	2,43182E-08	5	
Kazn	1,48517E-12	0,29848	0,452	0,38	2,49374E-08	5	
Cpeb1	1,72854E-12	0,252	0,345	0,254	2,9024E-08	5	
N4bp1	1,73124E-12	0,30768	0,529	0,483	2,90692E-08	5	
Rab27b	1,73358E-12	0,29046	0,456	0,368	2,91085E-08	5	
Glud1	1,91126E-12	0,27817	0,641	0,605	3,20919E-08	5	
Socs7	2,78442E-12	0,27237	0,391	0,308	4,67532E-08	5	
Nus1	2,81505E-12	0,30706	0,495	0,433	4,72674E-08	5	
Srrm3	2,85917E-12	0,29306	0,582	0,545	4,80083E-08	5	
Rbm33	2,87811E-12	0,27761	0,41	0,328	4,83263E-08	5	
Golga7b	2,99524E-12	0,31987	0,535	0,497	5,0293E-08	5	
Rbm26	3,07151E-12	0,30526	0,519	0,479	5,15737E-08	5	
Sppl3	3,08408E-12	0,25596	0,614	0,564	5,17848E-08	5	
Akap6	3,09121E-12	0,2699	0,723	0,677	5,19045E-08	5	
Gapvd1	3,1542E-12	0,29071	0,616	0,577	5,29622E-08	5	
Dmtn	3,209E-12	0,3127	0,544	0,511	5,38823E-08	5	
Dgcr2	3,25404E-12	0,27109	0,523	0,457	5,46386E-08	5	
Nras	3,39952E-12	0,32717	0,511	0,462	5,70813E-08	5	
Rere	3,96828E-12	0,27326	0,647	0,59	6,66315E-08	5	
Celf5	3,9832E-12	0,31056	0,508	0,447	6,6882E-08	5	
Cpne2	4,69474E-12	0,29779	0,523	0,466	7,88294E-08	5	
Zkscan3	4,72555E-12	0,29647	0,38	0,299	7,93467E-08	5	
Aph1a	4,73826E-12	0,29066	0,426	0,352	7,95601E-08	5	
Ppp2cb	6,50879E-12	0,27818	0,651	0,63	1,09289E-07	5	
Dip2c	6,83777E-12	0,30671	0,481	0,419	1,14813E-07	5	
Hnrnpr	6,84843E-12	0,26465	0,667	0,637	1,14992E-07	5	
Galnt11	7,65648E-12	0,3028	0,586	0,561	1,2856E-07	5	
Gmfb	7,69828E-12	0,29709	0,591	0,56	1,29262E-07	5	
Nlgn3	7,87488E-12	0,28094	0,424	0,348	1,32227E-07	5	
Adamts1	8,81244E-12	0,28562	0,696	0,613	1,4797E-07	5	
Snph	9,37712E-12	0,26441	0,398	0,316	1,57451E-07	5	
Dvl1	9,49867E-12	0,28238	0,462	0,397	1,59492E-07	5	
Tmem65	9,94424E-12	0,27226	0,634	0,604	1,66974E-07	5	
Tmem50b	1,00316E-11	0,27824	0,458	0,385	1,68441E-07	5	
Gm6483	1,00826E-11	0,27057	0,402	0,325	1,69296E-07	5	
Fto	1,06862E-11	0,27595	0,59	0,555	1,79433E-07	5	
Pip4k2b	1,12624E-11	0,2747	0,551	0,491	1,89107E-07	5	
Mllt4	1,30209E-11	0,28725	0,602	0,577	2,18635E-07	5	

Ssh2	1,32638E-11	0,28343	0,366	0,288	2,22713E-07	5	
March8	1,5635E-11	0,27435	0,42	0,344	2,62528E-07	5	
Arhgap12	1,80009E-11	0,27964	0,491	0,42	3,02253E-07	5	
Il6st	1,8276E-11	0,30346	0,474	0,41	3,06872E-07	5	
Evl	1,91583E-11	0,26383	0,668	0,631	3,21686E-07	5	
Kcnn3	2,05988E-11	0,31069	0,582	0,551	3,45874E-07	5	
Kcnh1	2,19959E-11	0,26973	0,42	0,338	3,69334E-07	5	
Dgkd	2,20202E-11	0,26536	0,434	0,36	3,69741E-07	5	
Fbxl20	2,31992E-11	0,2604	0,433	0,358	3,89538E-07	5	
Cep350	2,8124E-11	0,31491	0,486	0,434	4,72229E-07	5	
Med13	3,08171E-11	0,28557	0,444	0,373	5,17449E-07	5	
Dmxl1	3,50264E-11	0,28517	0,458	0,396	5,88128E-07	5	
Ppp1r2	3,92425E-11	0,25081	0,69	0,668	6,5892E-07	5	
Ubr3	4,35266E-11	0,28576	0,581	0,563	7,30855E-07	5	
Dennd5a	4,38714E-11	0,26478	0,558	0,519	7,36644E-07	5	
Crebrf	4,60945E-11	0,27423	0,499	0,445	7,73972E-07	5	
Clcn6	4,80629E-11	0,27279	0,546	0,502	8,07025E-07	5	
Dnajb14	4,86596E-11	0,26579	0,58	0,546	8,17044E-07	5	
Setd1b	5,20139E-11	0,28582	0,434	0,359	8,73365E-07	5	
Celf1	5,45791E-11	0,29643	0,525	0,475	9,16438E-07	5	
Mdn1	5,45987E-11	0,25298	0,356	0,273	9,16767E-07	5	
Stat2	5,51573E-11	0,27874	0,342	0,262	9,26146E-07	5	
Fam193a	6,6393E-11	0,28033	0,535	0,494	1,1148E-06	5	
Tmtc2	7,06095E-11	0,27927	0,505	0,455	1,1856E-06	5	
Megf8	7,34368E-11	0,29977	0,397	0,326	1,23308E-06	5	
Pan3	7,3828E-11	0,27475	0,425	0,352	1,23965E-06	5	
Osbpl5	7,58022E-11	0,25564	0,44	0,366	1,27279E-06	5	
Zdhhc20	7,61801E-11	0,28334	0,431	0,365	1,27914E-06	5	
Ece2	8,11599E-11	0,28103	0,451	0,391	1,36276E-06	5	
Trim56	1,42874E-10	0,27627	0,472	0,412	2,39899E-06	5	
Dab2ip	1,53917E-10	0,26607	0,572	0,531	2,58442E-06	5	
Ncoa2	1,64483E-10	0,27582	0,484	0,43	2,76183E-06	5	
Pkn2	1,83128E-10	0,28462	0,514	0,479	3,07491E-06	5	
Pacs2	1,96599E-10	0,27152	0,466	0,406	3,3011E-06	5	
Slc12a6	2,44233E-10	0,26551	0,418	0,347	4,10091E-06	5	
Srgap1	3,3514E-10	0,25598	0,349	0,273	5,62734E-06	5	
Thoc2	3,4723E-10	0,29417	0,55	0,524	5,83034E-06	5	
Dcbld2	3,68603E-10	0,25952	0,402	0,329	6,18921E-06	5	
Cnot6l	3,86058E-10	0,29956	0,438	0,377	6,48229E-06	5	
Bcl2l1	4,13435E-10	0,28738	0,552	0,519	6,94199E-06	5	
Spop	4,3116E-10	0,28286	0,596	0,579	7,23961E-06	5	
Atf2	4,62336E-10	0,28102	0,501	0,455	7,76308E-06	5	
Surf4	5,31949E-10	0,26353	0,585	0,552	8,93196E-06	5	
Brsk2	5,90955E-10	0,27438	0,561	0,529	9,92272E-06	5	
Npepps	6,56538E-10	0,27977	0,49	0,444	1,10239E-05	5	
Gprin3	6,99322E-10	0,26817	0,371	0,3	1,17423E-05	5	
Pitpnc1	7,08223E-10	0,27699	0,563	0,531	1,18918E-05	5	
Rbbp6	7,28172E-10	0,26219	0,608	0,59	1,22267E-05	5	

Wdr82	7,63286E-10	0,27468	0,505	0,457	1,28163E-05	5	
Pdpk1	1,01977E-09	0,30364	0,501	0,458	1,7123E-05	5	
Clip1	1,11325E-09	0,26202	0,502	0,455	1,86925E-05	5	
Nbl1	1,16861E-09	0,25146	0,617	0,574	1,96222E-05	5	
Tigar	1,27926E-09	0,28106	0,472	0,415	2,148E-05	5	
Suv420h1	1,35346E-09	0,2529	0,412	0,346	2,27259E-05	5	
Usp12	1,36129E-09	0,2705	0,513	0,492	2,28575E-05	5	
Dusp8	1,4475E-09	0,25984	0,546	0,52	2,4305E-05	5	
Prkar2a	1,51048E-09	0,25665	0,581	0,563	2,53625E-05	5	
Etv5	1,84439E-09	0,26799	0,389	0,322	3,09692E-05	5	
Dmxl2	2,10473E-09	0,25706	0,613	0,587	3,53405E-05	5	
Dtx3	3,03834E-09	0,26778	0,579	0,557	5,10167E-05	5	
Arhgap23	3,08194E-09	0,27044	0,413	0,354	5,17489E-05	5	
Gucy1a3	3,62819E-09	0,25931	0,552	0,512	6,0921E-05	5	
Zfp704	4,21268E-09	0,25954	0,42	0,361	7,07351E-05	5	
Rmnd5a	4,38821E-09	0,28969	0,517	0,492	7,36824E-05	5	
Rgmb	4,65515E-09	0,25422	0,396	0,329	7,81647E-05	5	
Fgfr1	6,1885E-09	0,25308	0,585	0,552	0,000103911	5	
Chd9	6,57873E-09	0,27886	0,543	0,514	0,000110463	5	
Zranb1	7,62738E-09	0,26523	0,523	0,502	0,000128071	5	
Agap1	8,13045E-09	0,27735	0,484	0,449	0,000136518	5	
Dnajc6	8,63535E-09	0,26863	0,533	0,505	0,000144996	5	
Bsn	8,67425E-09	0,26084	0,545	0,524	0,000145649	5	
Elavl2	1,15275E-08	0,25716	0,413	0,348	0,000193558	5	
Pcgf3	1,16141E-08	0,26064	0,467	0,423	0,000195013	5	
Setd2	1,32552E-08	0,27148	0,507	0,469	0,000222568	5	
Chic2	1,39326E-08	0,27769	0,545	0,53	0,000233943	5	
Lss	1,50083E-08	0,28601	0,421	0,368	0,000252004	5	
Ube2h	1,50914E-08	0,26565	0,539	0,517	0,000253399	5	
Kdm2a	1,66118E-08	0,26257	0,556	0,536	0,000278928	5	
Usp7	2,0494E-08	0,25576	0,554	0,535	0,000344114	5	
Tet1	2,25549E-08	0,25327	0,384	0,322	0,00037872	5	
Zfp207	2,42593E-08	0,25341	0,602	0,594	0,000407337	5	
Mgat4b	2,87818E-08	0,25736	0,529	0,501	0,000483276	5	
Herc3	2,95462E-08	0,28172	0,46	0,422	0,000496111	5	
Ttyh3	3,41908E-08	0,25183	0,473	0,431	0,000574098	5	
Cab39	3,72132E-08	0,25923	0,493	0,471	0,000624847	5	
Snx27	3,85799E-08	0,26087	0,442	0,399	0,000647795	5	
Dzip3	4,04391E-08	0,25579	0,515	0,482	0,000679013	5	
Uhmk1	4,0554E-08	0,25432	0,55	0,522	0,000680942	5	
Adam22	4,26881E-08	0,27113	0,471	0,435	0,000716776	5	
Safb2	4,99225E-08	0,25268	0,412	0,359	0,000838249	5	
Zfp638	5,17233E-08	0,25498	0,626	0,611	0,000868487	5	
Map3k2	5,28341E-08	0,26311	0,413	0,367	0,000887137	5	
Jmjd1c	6,43407E-08	0,25148	0,487	0,459	0,001080345	5	
Tab2	7,18466E-08	0,26113	0,433	0,391	0,001206377	5	
Mbd5	7,75701E-08	0,25184	0,449	0,404	0,00130248	5	
Vgf	8,14069E-08	0,26588	0,556	0,532	0,001366902	5	

Znrf1	8,44009E-08	0,2595	0,54	0,535	0,001417175	5	
Slc1a4	8,63031E-08	0,25547	0,422	0,366	0,001449116	5	
Rad21	1,07041E-07	0,25922	0,566	0,553	0,00179732	5	
Trpc4	1,2413E-07	0,25147	0,374	0,323	0,00208426	5	
G3bp1	1,28216E-07	0,26788	0,495	0,484	0,002152869	5	
Fam73a	7,63232E-07	0,25808	0,458	0,427	0,012815432	5	
Xist	2,94432E-06	0,76231	0,337	0,296	0,049438111	5	
Etv1	0	1,65925	1	0,595	0	6	smENC3a
Camk2a	0	1,49452	0,989	0,701	0	6	
Tcf4	7,4007E-273	1,04992	1	0,991	1,2427E-268	6	
Adgrl1	4,6943E-251	1,07933	0,995	0,869	7,8822E-247	6	
Syt7	2,8263E-245	1,18044	0,991	0,819	4,7456E-241	6	
F2r	1,1121E-241	1,49537	0,966	0,558	1,8674E-237	6	
Plekha5	9,1364E-227	1,19135	0,937	0,617	1,5341E-222	6	
Man2a1	1,6966E-225	1,18771	0,991	0,834	2,8487E-221	6	
Fam155a	8,1601E-220	1,05618	0,836	0,363	1,3702E-215	6	
Cntnap5a	2,1765E-219	1,10651	0,992	0,654	3,6545E-215	6	
Cd24a	8,9558E-212	1,25555	0,997	0,61	1,5038E-207	6	
Moxd1	1,8447E-208	1,14596	0,944	0,473	3,0974E-204	6	
Kcnd2	1,1352E-206	1,16539	0,817	0,374	1,906E-202	6	
Ncam1	4,5994E-206	0,74854	1	0,999	7,7228E-202	6	
Ptbp3	1,65E-204	1,04557	0,984	0,849	2,7706E-200	6	
Asic2	2,1703E-202	1,06001	0,979	0,754	3,6442E-198	6	
Ugcg	4,5823E-202	1,14294	0,97	0,768	7,6941E-198	6	
Etnk1	2,7398E-201	0,99286	0,987	0,865	4,6003E-197	6	
Gria3	3,6471E-197	0,93523	0,703	0,244	6,1238E-193	6	
Tbx3	2,525E-196	0,87513	1	0,941	4,2397E-192	6	
Pxylp1	3,1879E-195	1,05426	0,953	0,646	5,3527E-191	6	
Kcnt2	6,3643E-194	0,95522	0,758	0,301	1,0686E-189	6	
Auts2	9,4251E-191	0,99088	0,963	0,663	1,5826E-186	6	
Rimbp2	1,8055E-188	0,89964	0,716	0,265	3,0317E-184	6	
Efna5	3,8076E-184	1,09229	0,915	0,623	6,3934E-180	6	
9530059O14	7,1354E-184	0,98841	0,976	0,748	1,1981E-179	6	
Ptger4	1,4526E-183	0,98733	0,847	0,434	2,4391E-179	6	
Lrrc3	7,3865E-183	1,22217	0,79	0,413	1,2403E-178	6	
Cmip	2,314E-182	0,91513	0,985	0,845	3,8855E-178	6	
Alcam	1,4285E-180	1,09916	0,975	0,6	2,3986E-176	6	
Pam	1,7313E-175	0,81596	1	0,988	2,907E-171	6	
R3hdm1	8,8082E-175	0,91335	0,986	0,848	1,479E-170	6	
Stxbp5	1,429E-174	0,97471	0,938	0,669	2,3994E-170	6	
Arl8a	4,7381E-173	0,76072	0,999	0,984	7,9557E-169	6	
Vip	1,9823E-172	1,26743	1	0,925	3,3285E-168	6	
Spock3	1,1692E-170	0,9771	0,947	0,59	1,9632E-166	6	
Clmn	5,7004E-170	0,98147	0,841	0,47	9,5715E-166	6	
Gpr149	1,985E-169	0,90771	0,784	0,345	3,333E-165	6	
Pde3a	7,4395E-169	1,00682	0,813	0,427	1,2492E-164	6	
Kcnj3	9,4484E-168	0,78418	0,677	0,245	1,5865E-163	6	
Nav1	9,0302E-166	0,83424	0,992	0,909	1,5163E-161	6	

Kcnq3	2,2608E-162	0,9532	0,888	0,562	3,7961E-158	6	
Ptprn	1,3704E-161	0,65495	1	0,999	2,301E-157	6	
Nrxn1	1,4818E-160	0,69388	0,996	0,988	2,4881E-156	6	
Prokr1	6,5276E-158	0,78349	0,598	0,202	1,0961E-153	6	
Adcyap1r1	2,4549E-155	0,87967	0,784	0,39	4,122E-151	6	
Gsk3b	5,9637E-152	0,68891	0,996	0,984	1,0014E-147	6	
Hap1	1,0491E-150	0,85383	0,982	0,856	1,7616E-146	6	
Gnaq	6,0987E-150	0,92737	0,952	0,791	1,024E-145	6	
Garnl3	1,735E-149	0,83219	0,943	0,716	2,9132E-145	6	
Rbms3	3,7285E-147	0,7666	1	0,992	6,2605E-143	6	
Nbea	3,2703E-146	0,83482	0,889	0,618	5,4912E-142	6	
Chl1	5,4349E-146	0,87666	0,973	0,76	9,1257E-142	6	
mt-Co2	1,2264E-144	0,39292	1	1	2,0592E-140	6	
Elavl3	1,9128E-143	0,78127	0,989	0,917	3,2118E-139	6	
Gfra1	2,1088E-143	0,85051	0,899	0,44	3,5409E-139	6	
Ret	6,3743E-142	0,71015	0,997	0,965	1,0703E-137	6	
Camk2d	8,1918E-141	0,8234	0,944	0,755	1,3755E-136	6	
Nrip1	9,9054E-141	0,8389	0,979	0,864	1,6632E-136	6	
Cadm1	3,0608E-140	0,62652	1	0,996	5,1394E-136	6	
Ank2	9,1312E-140	0,58595	1	0,998	1,5332E-135	6	
Enpp1	1,4994E-139	0,72764	0,701	0,299	2,5177E-135	6	
Nsg2	2,3601E-139	0,66779	1	0,997	3,9628E-135	6	
mt-Atp6	4,3918E-139	0,3897	1	1	7,3743E-135	6	
Wnk1	2,0316E-138	0,78374	0,962	0,797	3,4113E-134	6	
Msi2	3,1288E-138	0,86069	0,948	0,772	5,2536E-134	6	
Pitpnc1	7,0013E-138	0,89609	0,83	0,503	1,1756E-133	6	
Frmd4a	6,4633E-137	0,70751	0,992	0,924	1,0853E-132	6	
Gm13889	1,9885E-136	0,87041	0,785	0,376	3,3389E-132	6	
Ndst4	1,1348E-135	0,78712	0,727	0,319	1,9055E-131	6	
Atp8a1	1,292E-135	0,81706	0,936	0,769	2,1693E-131	6	
mt-Atp8	1,6924E-135	0,70356	1	1	2,8418E-131	6	
Eml5	2,3295E-135	0,87499	0,846	0,547	3,9114E-131	6	
Klf7	1,691E-134	0,72409	0,994	0,944	2,8393E-130	6	
Celf3	1,1921E-132	0,80081	0,971	0,839	2,0016E-128	6	
Syne1	9,8965E-131	0,81154	0,89	0,648	1,6617E-126	6	
mt-Co3	1,8203E-130	0,35543	1	1	3,0565E-126	6	
Cacna2d1	1,8789E-130	0,74342	0,982	0,914	3,1548E-126	6	
Ngfr	6,2997E-130	0,82573	0,936	0,541	1,0578E-125	6	
Zfhx4	3,3775E-129	0,83955	0,736	0,384	5,6712E-125	6	
Ttc39b	4,2339E-129	0,80767	0,683	0,32	7,1091E-125	6	
Arpp21	6,7189E-129	0,80243	0,977	0,885	1,1282E-124	6	
Gabrb3	7,1358E-128	0,87809	0,824	0,564	1,1982E-123	6	
Mapt	7,5249E-127	0,67545	0,996	0,971	1,2635E-122	6	
Peg3	3,7278E-126	0,77666	0,997	0,98	6,2594E-122	6	
Actn1	6,582E-126	0,78699	0,878	0,612	1,1052E-121	6	
Sstr1	7,8208E-126	0,86718	0,862	0,619	1,3132E-121	6	
D430019H16	8,4365E-123	0,78922	0,866	0,618	1,4166E-118	6	
Pdlim5	3,6193E-121	0,74703	0,717	0,355	6,0772E-117	6	

Wipi1	4,6393E-121	0,74605	0,694	0,345	7,7898E-117	6	
Bmpr2	4,8852E-121	0,78042	0,913	0,731	8,2027E-117	6	
Camk4	5,0782E-121	0,83234	0,87	0,567	8,5268E-117	6	
Ids	2,2171E-120	0,82395	0,982	0,876	3,7227E-116	6	
Ece1	2,4989E-119	0,73376	0,941	0,724	4,1959E-115	6	
Cbx6	7,3261E-119	0,65848	0,997	0,942	1,2301E-114	6	
Tns1	9,0878E-119	0,72933	0,947	0,79	1,5259E-114	6	
Meg3	1,0584E-118	0,55404	1	1	1,7771E-114	6	
Tspan13	7,2977E-118	0,7545	0,953	0,746	1,2254E-113	6	
Prkar1a	1,1764E-117	0,59459	0,999	0,994	1,9752E-113	6	
Arhgap26	1,6008E-116	0,70272	0,961	0,816	2,688E-112	6	
Elavl4	1,8887E-116	0,56088	1	0,997	3,1714E-112	6	
Ago2	1,6146E-115	0,73071	0,914	0,716	2,711E-111	6	
Dpysl2	8,1833E-115	0,67065	0,996	0,982	1,3741E-110	6	
Kcnq1ot1	2,5323E-113	0,8381	0,972	0,845	4,2519E-109	6	
Ube3a	1,1213E-112	0,65813	0,97	0,874	1,8829E-108	6	
Zmat3	1,3421E-112	0,79333	0,823	0,585	2,2536E-108	6	
mt-Nd4l	2,0116E-111	0,55425	1	1	3,3776E-107	6	
Slc7a8	2,9692E-109	0,78572	0,758	0,447	4,9856E-105	6	
Phactr1	4,315E-108	0,71258	0,923	0,698	7,2454E-104	6	
Rbfox2	1,3583E-106	0,67122	0,946	0,846	2,2806E-102	6	
Socs2	1,5869E-105	0,70782	0,697	0,351	2,6646E-101	6	
Tspan12	1,8779E-105	0,63931	0,525	0,207	3,1532E-101	6	
Fibcd1	2,9307E-105	0,62075	0,798	0,386	4,9209E-101	6	
Insm1	5,7478E-105	0,70423	0,573	0,24	9,6511E-101	6	
Dpysl3	7,0397E-105	0,59644	1	0,991	1,182E-100	6	
Gse1	9,2837E-105	0,69384	0,968	0,819	1,5588E-100	6	
Dlg2	1,0349E-104	0,67842	0,894	0,716	1,7377E-100	6	
Tmod2	4,9035E-104	0,65114	0,98	0,904	8,2334E-100	6	
Cds2	6,2671E-104	0,63352	0,99	0,911	1,0523E-99	6	
Dlgap1	6,7081E-104	0,7299	0,812	0,56	1,1264E-99	6	
Lsamp	1,6979E-103	0,69519	0,908	0,726	2,851E-99	6	
Eef1e1	2,2342E-103	0,73244	0,84	0,526	3,7515E-99	6	
Mapk10	2,8238E-103	0,59066	0,973	0,885	4,7414E-99	6	
Rbms1	6,5306E-103	0,68617	0,941	0,797	1,09656E-98	6	
Taok1	7,8043E-103	0,68227	0,918	0,746	1,31041E-98	6	
Nrp1	9,3161E-103	0,68739	0,953	0,78	1,56427E-98	6	
Mycbp2	2,3517E-102	0,60537	0,992	0,962	3,9487E-98	6	
Tulp4	4,396E-102	0,67339	0,948	0,823	7,38126E-98	6	
Npy	5,4424E-102	0,87489	0,999	0,852	9,13826E-98	6	
Kmt2a	1,3334E-101	0,70395	0,886	0,703	2,23884E-97	6	
Rnf217	1,4955E-101	0,73091	0,703	0,406	2,51114E-97	6	
Serpini1	1,5331E-101	0,6811	0,915	0,735	2,57418E-97	6	
Hpcal4	3,918E-101	0,7408	0,824	0,569	6,57864E-97	6	
Pirt	1,0366E-100	0,59549	0,994	0,984	1,74063E-96	6	
Ptprd	2,2275E-100	0,60997	0,971	0,877	3,74027E-96	6	
Scn3a	2,9192E-100	0,65011	0,952	0,818	4,90169E-96	6	
Ptpre	7,9851E-100	0,60184	0,554	0,232	1,34078E-95	6	

Ubl3	1,2841E-99	0,68986	0,884	0,692	2,15616E-95	6	
Apba1	2,8401E-99	0,6771	0,927	0,743	4,76885E-95	6	
Epb41l5	8,2588E-99	0,61287	0,535	0,226	1,38674E-94	6	
Scd2	1,19577E-98	0,69562	0,989	0,912	2,00782E-94	6	
Kif1b	1,65394E-98	0,54735	1	0,992	2,77713E-94	6	
Nbeal1	3,84804E-98	0,65747	0,808	0,528	6,46124E-94	6	
BC005537	9,23935E-98	0,71283	0,883	0,723	1,55138E-93	6	
Lrrc8c	2,42799E-97	0,67964	0,876	0,666	4,07685E-93	6	
Pura	7,08805E-97	0,55603	0,995	0,965	1,19015E-92	6	
4930402H24	7,39384E-97	0,64245	0,841	0,588	1,2415E-92	6	
Entpd3	3,13116E-96	0,63735	0,726	0,39	5,25753E-92	6	
Serinc1	4,44163E-96	0,54002	0,997	0,988	7,45794E-92	6	
Slc4a4	1,95244E-95	0,67857	0,949	0,797	3,27834E-91	6	
Rnf152	3,52155E-95	0,51635	0,396	0,128	5,91303E-91	6	
Arvcf	4,67869E-95	0,65683	0,889	0,665	7,856E-91	6	
Plekhb2	7,43103E-95	0,72444	0,894	0,69	1,24774E-90	6	
Nfic	1,84366E-94	0,56008	0,992	0,948	3,09569E-90	6	
Kcnq4	6,27507E-94	0,61074	0,657	0,331	1,05365E-89	6	
Slc18a2	9,99636E-94	0,7323	0,54	0,228	1,67849E-89	6	
Slit2	2,08137E-93	0,69961	0,699	0,403	3,49482E-89	6	
Rab3c	3,51574E-93	0,53562	1	0,995	5,90329E-89	6	
Tmem108	4,88487E-93	0,59408	0,606	0,281	8,20219E-89	6	
Ywhag	1,73506E-92	0,5001	0,999	0,991	2,91334E-88	6	
Kif26a	1,71792E-91	0,62491	0,886	0,696	2,88456E-87	6	
Gria2	1,74586E-91	0,60134	0,989	0,928	2,93147E-87	6	
Atp6v1b2	4,92464E-91	0,61706	0,946	0,861	8,26897E-87	6	
Ubr5	6,36251E-91	0,62011	0,91	0,74	1,06833E-86	6	
Stxbp1	6,93244E-91	0,527	0,994	0,956	1,16403E-86	6	
Fus	2,53855E-90	0,59532	0,99	0,939	4,26249E-86	6	
Ptprg	3,07134E-90	0,66122	0,681	0,38	5,15708E-86	6	
Dst	1,6959E-89	0,43805	1	0,997	2,84759E-85	6	
Kcnb2	1,381E-88	0,73186	0,771	0,517	2,31884E-84	6	
Nav3	3,40578E-88	0,62285	0,672	0,367	5,71865E-84	6	
Atp7a	7,16177E-88	0,57153	0,503	0,214	1,20253E-83	6	
mt-Nd5	7,91293E-88	0,71831	1	1	1,32866E-83	6	
Diablo	9,58891E-88	0,49829	0,997	0,977	1,61007E-83	6	
Kif1a	4,92869E-87	0,51951	0,996	0,977	8,27577E-83	6	
Tmem30a	9,57691E-87	0,7333	0,947	0,858	1,60806E-82	6	
Gnao1	2,89393E-86	0,52944	0,994	0,965	4,85919E-82	6	
Tmem56	8,53989E-86	0,74464	0,674	0,417	1,43393E-81	6	
Man1a	2,5385E-85	0,60101	0,568	0,266	4,2624E-81	6	
Trim2	2,78666E-85	0,64753	0,816	0,614	4,67908E-81	6	
Tmem130	1,01222E-84	0,634	0,83	0,552	1,69962E-80	6	
mt-Co1	1,34467E-84	0,30812	1	1	2,25784E-80	6	
Mbnl1	2,67836E-84	0,64233	0,902	0,762	4,49724E-80	6	
Npy1r	6,55999E-84	0,55361	0,427	0,161	1,10149E-79	6	
Pik3r1	1,32569E-83	0,63189	0,819	0,595	2,22597E-79	6	
Plxna4	1,9888E-83	0,54904	0,999	0,964	3,33939E-79	6	

Cdh11	2,0011E-83	0,50718	0,451	0,176	3,36005E-79	6	
Adrbk2	2,08452E-83	0,52313	0,985	0,941	3,50012E-79	6	
Anks1b	2,25705E-83	0,68162	0,76	0,505	3,78981E-79	6	
Cacna1b	4,30612E-83	0,59476	0,862	0,657	7,23041E-79	6	
Syt4	4,53404E-83	0,61908	0,991	0,962	7,61311E-79	6	
Tmem64	5,47743E-83	0,60856	0,972	0,885	9,19715E-79	6	
Usp22	7,80427E-83	0,59358	0,931	0,826	1,31042E-78	6	
Sobp	1,24944E-82	0,67142	0,679	0,413	2,09793E-78	6	
Herc1	1,38046E-82	0,58639	0,885	0,719	2,31793E-78	6	
Sv2b	4,27783E-82	0,53167	0,523	0,228	7,18291E-78	6	
Rassf5	7,46042E-82	0,51535	0,5	0,214	1,25268E-77	6	
Srcin1	1,6442E-81	0,67687	0,812	0,59	2,76077E-77	6	
Kcnq5	5,49415E-81	0,45547	0,437	0,169	9,22523E-77	6	
Cacna1a	6,21414E-81	0,61798	0,817	0,569	1,04342E-76	6	
Arhgef12	8,51988E-81	0,65755	0,818	0,619	1,43057E-76	6	
Mafg	2,73701E-80	0,58448	0,878	0,65	4,59571E-76	6	
Ehd3	4,4649E-80	0,64499	0,852	0,641	7,49701E-76	6	
Thsd7a	5,70844E-80	0,57666	0,548	0,248	9,58504E-76	6	
Ptprz1	6,61529E-80	0,62125	0,595	0,289	1,11077E-75	6	
Adgrb3	2,03918E-79	0,60268	0,913	0,736	3,42398E-75	6	
Add3	3,28771E-79	0,6175	0,841	0,632	5,52039E-75	6	
Ppp3r1	6,41649E-79	0,61627	0,908	0,757	1,07739E-74	6	
Gm20342	1,58422E-78	0,64648	0,612	0,327	2,66006E-74	6	
Trps1	1,10801E-77	0,40522	0,345	0,113	1,86046E-73	6	
Dusp3	1,61249E-77	0,61584	0,88	0,742	2,70754E-73	6	
Zbtb20	1,71487E-77	0,54329	0,987	0,922	2,87944E-73	6	
mt-Nd1	2,00727E-77	0,39224	1	1	3,3704E-73	6	
Zbtb7a	2,15138E-77	0,60809	0,806	0,586	3,61237E-73	6	
Mmd	3,14056E-77	0,62186	0,938	0,81	5,27332E-73	6	
Ogt	1,82127E-76	0,62844	0,854	0,633	3,0581E-72	6	
Thy1	2,04715E-76	0,53972	0,888	0,523	3,43736E-72	6	
Plekhm3	3,48412E-75	0,61482	0,758	0,524	5,85018E-71	6	
Ttbk2	1,70724E-74	0,60752	0,848	0,631	2,86662E-70	6	
C2cd5	1,84369E-74	0,58825	0,681	0,416	3,09574E-70	6	
Lrrc8b	3,52033E-74	0,60946	0,663	0,401	5,91099E-70	6	
Disp2	3,84117E-74	0,60738	0,879	0,72	6,4497E-70	6	
Fam63b	4,88547E-74	0,6325	0,787	0,592	8,20319E-70	6	
Kcnj5	5,0869E-74	0,46074	0,431	0,171	8,54142E-70	6	
Mtss1	5,34295E-74	0,58945	0,706	0,44	8,97135E-70	6	
Lhfpl4	6,0849E-74	0,61469	0,737	0,516	1,02172E-69	6	
B3glct	1,062E-73	0,55847	0,563	0,282	1,7832E-69	6	
Slc22a23	1,41685E-73	0,40615	0,423	0,166	2,37904E-69	6	
Sdcbp	1,70852E-73	0,56529	0,96	0,868	2,86877E-69	6	
Cntn3	2,25429E-73	0,38968	0,341	0,115	3,78517E-69	6	
Gna12	3,56399E-73	0,58474	0,692	0,437	5,9843E-69	6	
Csnk1a1	4,1291E-73	0,51894	0,976	0,937	6,93317E-69	6	
Gm26917	8,02637E-73	0,77513	0,753	0,506	1,34771E-68	6	
Grn	1,64329E-72	0,56688	0,884	0,714	2,75925E-68	6	

Tmod1	1,69982E-72	0,52722	0,886	0,627	2,85417E-68	6	
Smg1	2,32846E-72	0,5578	0,689	0,427	3,90971E-68	6	
Unc80	2,68359E-72	0,54757	0,851	0,639	4,50601E-68	6	
Olfm1	3,52992E-72	0,58653	0,89	0,746	5,92709E-68	6	
Aatk	4,02169E-72	0,54224	0,827	0,59	6,75283E-68	6	
Doc2b	7,7524E-72	0,60478	0,678	0,421	1,30171E-67	6	
Rit2	8,74457E-72	0,61266	0,804	0,571	1,4683E-67	6	
Hipk2	1,1863E-71	0,47115	0,482	0,218	1,99192E-67	6	
Ralgapa1	2,19201E-71	0,57712	0,813	0,608	3,6806E-67	6	
Sntg1	4,36559E-71	0,46792	0,472	0,208	7,33025E-67	6	
Atp2b2	5,3185E-71	0,57571	0,716	0,467	8,9303E-67	6	
Usp34	2,47516E-70	0,51126	0,898	0,741	4,15605E-66	6	
Kmt2e	3,61253E-70	0,53037	0,912	0,749	6,06581E-66	6	
Ccnl1	8,62947E-70	0,5655	0,678	0,414	1,44897E-65	6	
Igf1r	1,1763E-69	0,60794	0,822	0,624	1,97512E-65	6	
Dpyd	1,33318E-69	0,5794	0,86	0,647	2,23855E-65	6	
Ttc14	3,65538E-69	0,53771	0,843	0,626	6,13774E-65	6	
Ap3s1	7,17302E-69	0,57299	0,803	0,604	1,20442E-64	6	
Lancl3	1,01766E-68	0,3666	0,299	0,096	1,70875E-64	6	
Epb41l1	1,30953E-68	0,50958	0,919	0,756	2,19883E-64	6	
Slc36a1	1,82546E-68	0,47133	0,999	0,961	3,06513E-64	6	
Cbx5	2,23591E-68	0,56038	0,86	0,717	3,75431E-64	6	
Akap11	2,24633E-68	0,47733	0,976	0,903	3,77182E-64	6	
Cnr1	2,59224E-68	0,33963	1	0,997	4,35263E-64	6	
Cntn1	2,66888E-68	0,58136	0,861	0,713	4,48132E-64	6	
Mat2a	2,76565E-68	0,64776	0,823	0,658	4,6438E-64	6	
Rapgef6	3,55581E-68	0,56381	0,758	0,526	5,97056E-64	6	
Arf3	4,41456E-68	0,53601	0,923	0,77	7,41248E-64	6	
Kcnc2	7,83461E-68	0,4113	0,405	0,163	1,31551E-63	6	
Sertm1	8,65196E-68	0,48348	0,499	0,23	1,45275E-63	6	
Gan	8,87051E-68	0,50274	0,53	0,268	1,48945E-63	6	
Syt17	1,32653E-67	0,55224	0,9	0,76	2,22737E-63	6	
Ino80d	3,70444E-67	0,56922	0,765	0,534	6,22012E-63	6	
Whsc1	4,18766E-67	0,53315	0,833	0,627	7,03149E-63	6	
Ascl1	5,28707E-67	0,49933	0,524	0,249	8,87752E-63	6	
Fbn1	5,9264E-67	0,46993	0,585	0,3	9,95101E-63	6	
Gls	7,71991E-67	0,5211	0,854	0,685	1,29625E-62	6	
Cxxc5	8,42221E-67	0,53327	0,944	0,844	1,41417E-62	6	
Stard4	1,01683E-66	0,68905	0,639	0,403	1,70736E-62	6	
Map7	1,54332E-66	0,54713	0,645	0,386	2,59138E-62	6	
Syt1	1,79889E-66	0,32802	1	1	3,02051E-62	6	
Apc	2,06974E-66	0,56141	0,871	0,748	3,47531E-62	6	
Kif5c	2,77077E-66	0,47494	0,994	0,957	4,6524E-62	6	
Nktr	5,22233E-66	0,54131	0,905	0,745	8,76882E-62	6	
Adora2a	5,98357E-66	0,41498	0,451	0,197	1,0047E-61	6	
Tbx3os1	6,48405E-66	0,4664	0,485	0,227	1,08874E-61	6	
Aplp2	8,40616E-66	0,42329	1	0,99	1,41148E-61	6	
Nrep	8,51386E-66	0,55745	0,636	0,374	1,42956E-61	6	

Pcdh15	1,03614E-65	0,61485	0,729	0,514	1,73978E-61	6	
Ubn2	1,54323E-65	0,58593	0,753	0,546	2,59123E-61	6	
Bicd1	4,16164E-65	0,53687	0,824	0,646	6,9878E-61	6	
Kcnk2	4,63222E-65	0,47851	0,986	0,936	7,77795E-61	6	
Soga3	5,67797E-65	0,47365	0,951	0,853	9,53388E-61	6	
Camsap2	6,36862E-65	0,54717	0,832	0,642	1,06936E-60	6	
Atp1b3	7,01425E-65	0,47694	0,975	0,922	1,17776E-60	6	
Ccdc50	1,00323E-64	0,5566	0,862	0,704	1,68453E-60	6	
Slc2a13	2,26911E-64	0,58817	0,628	0,381	3,81006E-60	6	
RP23-54G8.4	2,33993E-64	0,44489	0,424	0,185	3,92897E-60	6	
Usp9x	2,57113E-64	0,45079	0,967	0,885	4,31718E-60	6	
Ttyh3	2,82377E-64	0,56505	0,672	0,41	4,74139E-60	6	
Arrdc3	3,8875E-64	0,50226	0,54	0,283	6,52751E-60	6	
Rasl10b	6,7029E-64	0,49965	0,678	0,413	1,12548E-59	6	
Zbtb4	1,78698E-63	0,58599	0,679	0,451	3,00051E-59	6	
Ski	4,74424E-63	0,51542	0,813	0,61	7,96606E-59	6	
Pde4a	5,18129E-63	0,43958	0,386	0,16	8,6999E-59	6	
Spock1	9,32923E-63	0,52453	0,544	0,275	1,56647E-58	6	
Unc5b	1,42961E-62	0,5914	0,713	0,493	2,40047E-58	6	
Ralgds	2,17985E-62	0,52157	0,811	0,613	3,66018E-58	6	
Ets1	7,21957E-62	0,42022	0,463	0,216	1,21224E-57	6	
Slc35b4	9,76096E-62	0,53603	0,742	0,511	1,63896E-57	6	
Insr	6,18321E-61	0,50426	0,578	0,324	1,03822E-56	6	
Stx3	9,60298E-61	0,51976	0,578	0,328	1,61244E-56	6	
Kcnn3	1,22597E-60	0,63499	0,739	0,535	2,05853E-56	6	
Wscd2	1,36292E-60	0,46365	0,497	0,246	2,28848E-56	6	
Atp2c1	3,33903E-60	0,48676	0,78	0,578	5,60656E-56	6	
Fry	4,01446E-60	0,54561	0,732	0,528	6,74068E-56	6	
Zmiz1	4,76752E-60	0,52655	0,865	0,716	8,00515E-56	6	
Basp1	4,80775E-60	0,39386	1	0,999	8,0727E-56	6	
Hdac7	1,07301E-59	0,51309	0,634	0,378	1,8017E-55	6	
Gabre	1,83006E-59	0,45861	0,409	0,172	3,07285E-55	6	
Nt5dc3	2,35663E-59	0,51498	0,86	0,688	3,95701E-55	6	
Lrrc58	1,14642E-58	0,50658	0,933	0,821	1,92496E-54	6	
Cdc42bpa	1,32232E-58	0,46478	0,864	0,702	2,22031E-54	6	
Parva	1,66131E-58	0,48195	0,956	0,846	2,78951E-54	6	
Lims1	2,37524E-58	0,53797	0,765	0,577	3,98827E-54	6	
Map2	3,05649E-58	0,47742	0,941	0,839	5,13214E-54	6	
Nek1	3,19291E-58	0,52443	0,744	0,528	5,36121E-54	6	
Cbarp	3,45085E-58	0,47548	0,97	0,888	5,79432E-54	6	
Prkar1b	8,97899E-58	0,43565	0,937	0,814	1,50766E-53	6	
Celf1	1,15966E-57	0,56265	0,673	0,46	1,94718E-53	6	
Rc3h2	1,91515E-57	0,51142	0,789	0,607	3,21572E-53	6	
Purb	2,16434E-57	0,52573	0,898	0,803	3,63414E-53	6	
Fzd3	4,17969E-57	0,51641	0,689	0,469	7,01813E-53	6	
Fosl2	7,31461E-57	0,54374	0,622	0,384	1,2282E-52	6	
Ocrl	1,44208E-56	0,56189	0,683	0,482	2,42139E-52	6	
Mgll	2,19281E-56	0,48609	0,885	0,755	3,68195E-52	6	

Prrc2b	2,43315E-56	0,43352	0,965	0,884	4,0855E-52	6	
Rap1gap2	4,2155E-56	0,50597	0,855	0,707	7,07824E-52	6	
Lyst	4,22747E-56	0,50716	0,721	0,511	7,09834E-52	6	
Scgn	8,86219E-56	0,56374	1	0,866	1,48805E-51	6	
Mib1	9,94063E-56	0,49658	0,655	0,427	1,66913E-51	6	
Ywhaz	3,70586E-55	0,43528	0,996	0,992	6,22251E-51	6	
Nras	4,02289E-55	0,50945	0,662	0,447	6,75484E-51	6	
Cpeb2	4,43042E-55	0,50741	0,629	0,404	7,43912E-51	6	
Tlk1	4,50997E-55	0,49891	0,77	0,577	7,57268E-51	6	
Glg1	5,75496E-55	0,54354	0,795	0,627	9,66315E-51	6	
Rc3h1	6,89816E-55	0,49681	0,807	0,645	1,15827E-50	6	
Tcp11l1	7,10179E-55	0,47612	0,631	0,399	1,19246E-50	6	
Lix1	1,01329E-54	0,50305	0,902	0,757	1,70142E-50	6	
Nfat5	1,01916E-54	0,50027	0,761	0,557	1,71127E-50	6	
Gnb4	1,0644E-54	0,43732	0,967	0,912	1,78723E-50	6	
Fsd1l	1,15221E-54	0,50247	0,773	0,593	1,93468E-50	6	
Ldlr	1,47573E-54	0,52565	0,818	0,635	2,47789E-50	6	
Mab21l1	2,67679E-54	0,52075	0,63	0,403	4,4946E-50	6	
Ptpra	4,64018E-54	0,47631	0,848	0,707	7,79132E-50	6	
Arl2bp	5,61007E-54	0,47882	0,86	0,706	9,41987E-50	6	
Scn2a1	8,91758E-54	0,48331	0,698	0,467	1,49735E-49	6	
Gng4	1,06664E-53	0,52668	0,842	0,672	1,79099E-49	6	
Erbb2ip	1,52791E-53	0,43486	0,556	0,316	2,56551E-49	6	
Synpo2	1,69275E-53	0,39408	0,399	0,178	2,84229E-49	6	
Klhl29	2,05267E-53	0,39005	0,438	0,21	3,44664E-49	6	
Prrc2a	3,96417E-53	0,44404	0,861	0,719	6,65624E-49	6	
Cbl	1,13482E-52	0,50671	0,718	0,511	1,90548E-48	6	
Osbpl3	1,60562E-52	0,51347	0,65	0,45	2,696E-48	6	
Maged1	1,71766E-52	0,43784	0,996	0,961	2,88412E-48	6	
Slc5a3	1,81368E-52	0,44913	0,576	0,333	3,04536E-48	6	
Syt11	4,0932E-52	0,39527	0,999	0,991	6,87289E-48	6	
Slc7a1	7,11194E-52	0,48518	0,624	0,398	1,19417E-47	6	
Htr2c	7,75324E-52	0,3848	0,364	0,158	1,30185E-47	6	
Cpeb4	8,72916E-52	0,50338	0,751	0,569	1,46571E-47	6	
Pnisr	1,06576E-51	0,44529	0,879	0,724	1,78951E-47	6	
Col4a1	1,26079E-51	0,37215	0,375	0,167	2,117E-47	6	
Baz2a	1,28498E-51	0,46044	0,62	0,386	2,15761E-47	6	
Atp2a2	1,55815E-51	0,44924	0,933	0,84	2,61629E-47	6	
Dusp11	1,99593E-51	0,48822	0,792	0,593	3,35136E-47	6	
Abcc5	2,07336E-51	0,47729	0,848	0,713	3,48137E-47	6	
Lrch3	2,27057E-51	0,44536	0,553	0,322	3,81251E-47	6	
Ddx17	2,81868E-51	0,45971	0,917	0,796	4,73285E-47	6	
Rtn3	3,1114E-51	0,34823	1	0,997	5,22436E-47	6	
Wwc2	3,50669E-51	0,51636	0,72	0,522	5,88809E-47	6	
Lasp1	3,61983E-51	0,46561	0,583	0,353	6,07806E-47	6	
Camta1	4,62831E-51	0,48998	0,941	0,861	7,7714E-47	6	
Znrf1	5,85811E-51	0,47775	0,716	0,516	9,83635E-47	6	
mt-Nd4	7,09526E-51	0,25349	1	1	1,19136E-46	6	

Ncald	8,06194E-51	0,4687	0,912	0,798	1,35368E-46	6	
Etl4	9,95529E-51	0,34664	0,335	0,138	1,67159E-46	6	
Syn2	1,15938E-50	0,2944	1	1	1,94672E-46	6	
A830018L16	1,46641E-50	0,44823	0,562	0,331	2,46225E-46	6	
Cit	1,55636E-50	0,37465	0,394	0,18	2,61328E-46	6	
Sh3pxd2a	2,27262E-50	0,46118	0,784	0,598	3,81596E-46	6	
Zzef1	4,48408E-50	0,47263	0,619	0,399	7,52922E-46	6	
Pum2	4,67559E-50	0,51503	0,744	0,562	7,85079E-46	6	
Mbnl2	5,78099E-50	0,43416	0,917	0,815	9,70685E-46	6	
Slc2a3	6,69501E-50	0,4735	0,694	0,492	1,12416E-45	6	
Kcnab2	7,4097E-50	0,47316	0,616	0,382	1,24416E-45	6	
Cyb5b	9,12401E-50	0,49742	0,756	0,563	1,53201E-45	6	
Pank3	1,29644E-49	0,50739	0,764	0,6	2,17686E-45	6	
Cpm	1,52948E-49	0,40107	0,374	0,168	2,56815E-45	6	
B4galt6	2,31736E-49	0,50004	0,841	0,691	3,89108E-45	6	
Mga	3,4321E-49	0,47952	0,699	0,494	5,76285E-45	6	
Cpne3	3,93616E-49	0,4977	0,823	0,68	6,6092E-45	6	
Kcnv1	5,22911E-49	0,34039	0,343	0,145	8,78019E-45	6	
Kcnq2	5,80078E-49	0,45739	0,802	0,624	9,74009E-45	6	
Trim35	6,74845E-49	0,47663	0,814	0,666	1,13313E-44	6	
Camkk2	7,30897E-49	0,48018	0,652	0,434	1,22725E-44	6	
Cntnap2	7,97142E-49	0,45467	0,554	0,325	1,33848E-44	6	
Wdfy3	9,53325E-49	0,44805	0,737	0,535	1,60073E-44	6	
Arl6ip1	1,28663E-48	0,44354	0,936	0,859	2,16038E-44	6	
Kmt2d	1,40297E-48	0,44993	0,616	0,391	2,35572E-44	6	
Uhmk1	1,59061E-48	0,53533	0,687	0,507	2,67079E-44	6	
Rb1cc1	1,62324E-48	0,44867	0,88	0,751	2,72557E-44	6	
Grin3a	1,74916E-48	0,4705	0,557	0,328	2,93702E-44	6	
Akap13	3,38754E-48	0,41463	0,966	0,876	5,68802E-44	6	
Dtna	5,16891E-48	0,42106	0,492	0,27	8,67912E-44	6	
Ablim2	5,86144E-48	0,45294	0,587	0,366	9,84194E-44	6	
Pten	5,88626E-48	0,48099	0,766	0,621	9,88363E-44	6	
Xkr6	6,09214E-48	0,45542	0,571	0,349	1,02293E-43	6	
Bex2	6,15823E-48	0,36165	1	0,999	1,03403E-43	6	
Creb5	6,28746E-48	0,47143	0,447	0,234	1,05573E-43	6	
Pde10a	1,14932E-47	0,46444	0,913	0,793	1,92982E-43	6	
Hdac4	1,73449E-47	0,46213	0,664	0,453	2,91239E-43	6	
Cplx2	3,27096E-47	0,50995	0,785	0,606	5,49227E-43	6	
Tnrc6b	3,41455E-47	0,51125	0,73	0,56	5,73338E-43	6	
Utrn	3,64575E-47	0,4653	0,836	0,683	6,12157E-43	6	
Atp2b4	6,33382E-47	0,45226	0,867	0,729	1,06351E-42	6	
Srsf5	7,7167E-47	0,44781	0,827	0,681	1,29571E-42	6	
Lrrc4c	1,22178E-46	0,34647	0,347	0,154	2,0515E-42	6	
Cldnd1	1,26764E-46	0,45715	0,779	0,619	2,1285E-42	6	
Krit1	2,49171E-46	0,45547	0,773	0,605	4,18382E-42	6	
Mapk1ip1l	3,05297E-46	0,48909	0,622	0,426	5,12625E-42	6	
Clk1	3,53209E-46	0,41561	0,928	0,788	5,93074E-42	6	
Gpr22	4,15013E-46	0,4617	0,866	0,711	6,96849E-42	6	

Mkln1	4,30753E-46	0,47147	0,843	0,694	7,23278E-42	6	
Scarb1	5,58261E-46	0,36333	0,389	0,184	9,37376E-42	6	
Ddx6	5,92385E-46	0,43856	0,819	0,683	9,94674E-42	6	
Luc7l2	1,20784E-45	0,43795	0,904	0,8	2,02809E-41	6	
Agap1	1,26963E-45	0,45097	0,639	0,433	2,13183E-41	6	
Tbc1d9	1,43847E-45	0,46699	0,665	0,483	2,41534E-41	6	
Tia1	1,49362E-45	0,48663	0,72	0,542	2,50794E-41	6	
Rbm5	2,40617E-45	0,43371	0,847	0,696	4,04019E-41	6	
Helz	2,53412E-45	0,47566	0,649	0,444	4,25504E-41	6	
Pcgf3	3,29973E-45	0,4327	0,62	0,407	5,54058E-41	6	
Ptgfrn	4,0556E-45	0,3478	0,345	0,152	6,80975E-41	6	
Tnrc6c	4,41717E-45	0,40468	0,942	0,843	7,41686E-41	6	
Sdc3	4,79462E-45	0,38474	0,937	0,817	8,05065E-41	6	
Rgs7bp	5,47531E-45	0,49551	0,741	0,596	9,1936E-41	6	
Lrp11	5,79347E-45	0,43905	0,893	0,792	9,72782E-41	6	
Etv6	5,80896E-45	0,39201	0,461	0,248	9,75382E-41	6	
Azin1	6,27618E-45	0,46276	0,832	0,714	1,05383E-40	6	
Prune2	6,49806E-45	0,40054	0,939	0,824	1,09109E-40	6	
Lnpep	6,77935E-45	0,45767	0,657	0,46	1,13832E-40	6	
Dnmt3a	8,18109E-45	0,42608	0,852	0,7	1,37369E-40	6	
Fem1b	9,18781E-45	0,4683	0,763	0,601	1,54273E-40	6	
Rock2	9,40476E-45	0,46275	0,72	0,544	1,57915E-40	6	
Mef2a	9,68825E-45	0,44016	0,753	0,575	1,62675E-40	6	
Nr2f2	1,0316E-44	0,32803	0,314	0,134	1,73216E-40	6	
Cdon	1,33103E-44	0,4147	0,437	0,23	2,23494E-40	6	
Neurl1a	2,06301E-44	0,39588	0,891	0,722	3,464E-40	6	
Myo1b	2,0635E-44	0,36669	0,489	0,265	3,46483E-40	6	
Sox4	2,25778E-44	0,48174	0,942	0,86	3,79104E-40	6	
Cep170b	2,76235E-44	0,41885	0,756	0,57	4,63826E-40	6	
Pip4k2b	3,42542E-44	0,45174	0,674	0,478	5,75163E-40	6	
Slc4a8	3,95981E-44	0,45557	0,716	0,531	6,64892E-40	6	
Ash1l	5,87788E-44	0,42292	0,83	0,712	9,86955E-40	6	
Plppr5	6,24382E-44	0,45994	0,876	0,797	1,0484E-39	6	
Mllt1	9,50359E-44	0,38938	0,462	0,252	1,59575E-39	6	
Clvs1	9,72839E-44	0,41303	0,518	0,307	1,63349E-39	6	
9330159F19	1,34522E-43	0,43157	0,585	0,375	2,25877E-39	6	
Adra2a	2,0901E-43	0,40113	0,472	0,257	3,50949E-39	6	
Ttc3	5,57494E-43	0,33358	0,997	0,991	9,36089E-39	6	
Tmtc2	5,67083E-43	0,50188	0,635	0,441	9,52189E-39	6	
Fstl1	6,09902E-43	0,47822	0,77	0,609	1,02409E-38	6	
Rgag4	6,33619E-43	0,35198	0,444	0,234	1,06391E-38	6	
Man2a2	8,0137E-43	0,40024	0,638	0,428	1,34558E-38	6	
Npy2r	8,32864E-43	0,48568	0,798	0,65	1,39846E-38	6	
Cdyl2	8,35672E-43	0,35509	0,463	0,252	1,40318E-38	6	
Isoc1	8,46343E-43	0,42021	0,601	0,382	1,42109E-38	6	
Slco3a1	9,06899E-43	0,38546	0,503	0,28	1,52277E-38	6	
Rnf165	1,32847E-42	0,41171	0,5	0,288	2,23064E-38	6	
Man1c1	1,38827E-42	0,32243	0,352	0,163	2,33104E-38	6	

Arid1a	1,52567E-42	0,43935	0,689	0,5	2,56175E-38	6	
Ube2d3	2,26338E-42	0,42543	0,855	0,754	3,80045E-38	6	
Ppp2r5c	2,36117E-42	0,43665	0,686	0,5	3,96465E-38	6	
Ywhah	3,56904E-42	0,43275	0,997	0,989	5,99278E-38	6	
Celf6	4,35051E-42	0,3633	0,979	0,948	7,30495E-38	6	
Frmpd4	6,03229E-42	0,34064	0,375	0,183	1,01288E-37	6	
Huwe1	8,44405E-42	0,38766	0,903	0,811	1,41784E-37	6	
Mtpn	9,32114E-42	0,42697	0,888	0,767	1,56511E-37	6	
Tmem200a	1,60516E-41	0,27814	0,299	0,125	2,69523E-37	6	
Pcnp	1,75647E-41	0,46595	0,711	0,55	2,94928E-37	6	
Fbxl20	1,82142E-41	0,40053	0,556	0,346	3,05835E-37	6	
Atp11c	2,4702E-41	0,4046	0,492	0,287	4,14771E-37	6	
Ptpn1	2,69005E-41	0,43366	0,712	0,554	4,51686E-37	6	
Chst15	2,98966E-41	0,3659	0,418	0,212	5,01994E-37	6	
Dmd	3,19803E-41	0,36963	0,389	0,198	5,36982E-37	6	
Plekha6	4,04517E-41	0,38418	0,914	0,773	6,79224E-37	6	
Hdgfrp3	4,88141E-41	0,43193	0,813	0,698	8,19637E-37	6	
Adrbk1	5,06024E-41	0,38581	0,832	0,679	8,49665E-37	6	
Ppm1a	5,44956E-41	0,40298	0,87	0,769	9,15036E-37	6	
Slc6a6	6,14276E-41	0,45146	0,744	0,571	1,03143E-36	6	
Jmjd1c	8,75235E-41	0,40666	0,644	0,442	1,46961E-36	6	
Calm1	8,85911E-41	0,26118	1	1	1,48753E-36	6	
Ccnt2	1,03678E-40	0,42318	0,636	0,448	1,74085E-36	6	
Dmtn	1,11322E-40	0,41891	0,684	0,497	1,8692E-36	6	
Fam168b	1,32736E-40	0,42033	0,726	0,554	2,22877E-36	6	
Tes	1,90991E-40	0,37969	0,504	0,293	3,20693E-36	6	
Spry2	3,12067E-40	0,29131	0,293	0,126	5,23992E-36	6	
Gnb1	3,43717E-40	0,33266	0,997	0,989	5,77136E-36	6	
Agrn	3,82859E-40	0,34911	0,432	0,231	6,42859E-36	6	
Larp4b	5,17923E-40	0,41543	0,775	0,619	8,69645E-36	6	
Rab6b	5,96459E-40	0,40535	0,968	0,917	1,00152E-35	6	
Scn5a	6,02195E-40	0,39541	0,809	0,652	1,01115E-35	6	
Fndc3a	6,03288E-40	0,43763	0,763	0,612	1,01298E-35	6	
Shc1	6,59366E-40	0,38516	0,529	0,32	1,10714E-35	6	
Ptp4a1	6,9485E-40	0,45338	0,764	0,61	1,16672E-35	6	
Whsc1l1	8,09329E-40	0,409	0,755	0,596	1,35894E-35	6	
Gatad2b	8,38275E-40	0,46781	0,699	0,546	1,40755E-35	6	
Nlk	8,39848E-40	0,35917	0,451	0,251	1,41019E-35	6	
Unc13a	8,72253E-40	0,41855	0,702	0,527	1,4646E-35	6	
Tmem184b	1,33113E-39	0,37465	0,496	0,296	2,2351E-35	6	
Celf5	1,63481E-39	0,43652	0,619	0,436	2,74501E-35	6	
Tmem178b	2,05773E-39	0,421	0,723	0,547	3,45513E-35	6	
Brd2	2,36161E-39	0,36162	0,903	0,784	3,96538E-35	6	
Pcdh1	3,64377E-39	0,38523	0,823	0,67	6,11826E-35	6	
Gigyf1	4,05906E-39	0,41688	0,628	0,425	6,81557E-35	6	
Chp1	4,33465E-39	0,42178	0,806	0,67	7,27832E-35	6	
Zyg11b	4,45962E-39	0,44741	0,684	0,515	7,48815E-35	6	
Cpne4	5,93097E-39	0,35374	0,928	0,822	9,95869E-35	6	

Plcb4	9,03391E-39	0,33634	0,999	0,949	1,51688E-34	6	
Mirg	9,16353E-39	0,42243	0,654	0,467	1,53865E-34	6	
Zmynd11	1,03407E-38	0,37743	0,847	0,712	1,73631E-34	6	
Prkacb	1,23605E-38	0,36384	0,968	0,922	2,07545E-34	6	
Rbfox1	1,4227E-38	0,40327	0,807	0,659	2,38886E-34	6	
Git2	1,45593E-38	0,40499	0,631	0,431	2,44465E-34	6	
Myo9a	1,60778E-38	0,39718	0,804	0,666	2,69962E-34	6	
Fam8a1	1,80295E-38	0,43266	0,644	0,469	3,02733E-34	6	
Oprd1	1,98859E-38	0,28783	0,317	0,145	3,33904E-34	6	
Zdhhc2	2,08155E-38	0,45953	0,766	0,636	3,49513E-34	6	
Fam60a	2,91456E-38	0,28546	0,303	0,136	4,89384E-34	6	
Clec14a	3,60885E-38	0,40174	0,473	0,271	6,05963E-34	6	
Cdr2l	4,19964E-38	0,36361	0,52	0,319	7,05161E-34	6	
Magt1	4,65411E-38	0,45353	0,525	0,336	7,81472E-34	6	
Bptf	4,85186E-38	0,36508	0,884	0,773	8,14676E-34	6	
Napb	5,01221E-38	0,40299	0,885	0,788	8,41601E-34	6	
Acer3	5,03571E-38	0,39466	0,419	0,235	8,45547E-34	6	
Atf7	5,13039E-38	0,40162	0,625	0,428	8,61444E-34	6	
Zfhx2	5,16273E-38	0,35185	0,452	0,254	8,66874E-34	6	
Lats2	5,57758E-38	0,32227	0,386	0,2	9,36531E-34	6	
Tnrc6a	6,47741E-38	0,38719	0,933	0,848	1,08762E-33	6	
Mark2	7,13437E-38	0,38143	0,735	0,546	1,19793E-33	6	
Zfp950	1,11433E-37	0,4037	0,573	0,379	1,87107E-33	6	
Syt14	1,15415E-37	0,40075	0,461	0,274	1,93793E-33	6	
Med13	1,25909E-37	0,39738	0,557	0,362	2,11414E-33	6	
Zmym5	1,31076E-37	0,40198	0,572	0,38	2,2009E-33	6	
Rab10	1,35283E-37	0,39232	0,77	0,638	2,27154E-33	6	
B830012L14I	1,76418E-37	0,34512	0,366	0,184	2,96224E-33	6	
Abr	2,26838E-37	0,36857	0,761	0,597	3,80884E-33	6	
Unc79	2,33706E-37	0,39371	0,577	0,385	3,92415E-33	6	
Ncam2	2,60944E-37	0,41173	0,871	0,773	4,3815E-33	6	
Hook3	2,6206E-37	0,4242	0,676	0,518	4,40024E-33	6	
Mid1ip1	2,80949E-37	0,41625	0,744	0,577	4,71741E-33	6	
Pik3ca	3,01576E-37	0,43552	0,706	0,541	5,06376E-33	6	
D17Wsu92e	3,18086E-37	0,39628	0,831	0,704	5,34098E-33	6	
Wsb2	3,4071E-37	0,39664	0,756	0,616	5,72087E-33	6	
Irak1	3,68513E-37	0,41852	0,574	0,393	6,18771E-33	6	
Coro2b	3,8316E-37	0,39661	0,782	0,634	6,43364E-33	6	
A730017C20	4,59528E-37	0,37276	0,9	0,701	7,71594E-33	6	
Lrrk2	5,43966E-37	0,29987	0,35	0,173	9,13373E-33	6	
Csde1	6,14341E-37	0,38094	0,869	0,78	1,03154E-32	6	
Ptpn4	6,62072E-37	0,33929	0,423	0,232	1,11168E-32	6	
Sfpq	6,81423E-37	0,37925	0,872	0,792	1,14418E-32	6	
Rtn1	9,38365E-37	0,28154	1	0,999	1,57561E-32	6	
Kidins220	1,03325E-36	0,4026	0,809	0,685	1,73494E-32	6	
Tmem245	1,19074E-36	0,42442	0,596	0,406	1,99936E-32	6	
Nsd1	1,25232E-36	0,38706	0,818	0,678	2,10277E-32	6	
Iqsec3	1,29681E-36	0,41183	0,66	0,486	2,17748E-32	6	

Gramd1b	1,59754E-36	0,38864	0,557	0,366	2,68242E-32	6	
Pbx1	1,98642E-36	0,39349	0,886	0,754	3,3354E-32	6	
Orai2	2,04777E-36	0,4009	0,639	0,451	3,43841E-32	6	
Ubr2	3,59641E-36	0,40014	0,626	0,453	6,03873E-32	6	
Rac1	3,66567E-36	0,35976	0,931	0,858	6,15503E-32	6	
Smad1	4,17496E-36	0,399	0,638	0,455	7,01017E-32	6	
Zdhhc20	6,091E-36	0,37102	0,554	0,353	1,02274E-31	6	
Arf1	7,11146E-36	0,32316	0,976	0,952	1,19409E-31	6	
Cygb	8,86426E-36	0,39446	0,592	0,394	1,4884E-31	6	
Cpne5	1,0105E-35	0,36452	0,418	0,233	1,69672E-31	6	
Fnbp1l	1,03481E-35	0,42413	0,729	0,579	1,73754E-31	6	
Sgsm1	1,17453E-35	0,40777	0,583	0,409	1,97216E-31	6	
Pafah1b1	1,36812E-35	0,3097	0,996	0,989	2,29721E-31	6	
Klf13	1,39568E-35	0,37133	0,837	0,711	2,34348E-31	6	
Atp11a	1,59062E-35	0,35436	0,479	0,286	2,6708E-31	6	
Mbd6	1,71068E-35	0,36524	0,494	0,298	2,8724E-31	6	
Trrap	1,74214E-35	0,38991	0,568	0,374	2,92523E-31	6	
Lamc3	2,04813E-35	0,3027	0,51	0,29	3,43901E-31	6	
Zfp871	2,31045E-35	0,38556	0,466	0,284	3,87947E-31	6	
Pde5a	2,39756E-35	0,38687	0,501	0,313	4,02574E-31	6	
Impad1	2,55903E-35	0,43928	0,682	0,519	4,29686E-31	6	
Snrk	2,86096E-35	0,3941	0,821	0,7	4,80383E-31	6	
Msl1	2,98741E-35	0,40262	0,726	0,601	5,01617E-31	6	
2410089E03	2,9893E-35	0,39512	0,679	0,508	5,01933E-31	6	
Tm9sf3	3,59569E-35	0,37502	0,884	0,804	6,03752E-31	6	
Pbxip1	3,64182E-35	0,31915	0,379	0,197	6,11498E-31	6	
Sgsm2	4,13147E-35	0,40597	0,63	0,458	6,93715E-31	6	
mt-Nd3	6,67897E-35	0,28859	1	1	1,12147E-30	6	
Braf	7,41333E-35	0,39778	0,679	0,515	1,24477E-30	6	
Rcor3	8,63447E-35	0,36765	0,624	0,432	1,44981E-30	6	
Slc36a4	9,2649E-35	0,41884	0,542	0,359	1,55567E-30	6	
March8	1,11706E-34	0,35565	0,529	0,333	1,87566E-30	6	
Cuedc1	1,16978E-34	0,39153	0,717	0,573	1,96417E-30	6	
Ip6k1	1,23289E-34	0,36599	0,845	0,695	2,07014E-30	6	
Gna11	1,34765E-34	0,35468	0,938	0,864	2,26284E-30	6	
Atp6v0a1	1,73947E-34	0,32612	0,944	0,871	2,92074E-30	6	
Psap	2,47027E-34	0,29445	1	0,999	4,14783E-30	6	
Celf4	3,05768E-34	0,28939	0,995	0,975	5,13415E-30	6	
Grik5	3,68735E-34	0,37054	0,766	0,61	6,19143E-30	6	
Sbf2	3,78324E-34	0,35098	0,542	0,348	6,35244E-30	6	
Asxl2	3,93488E-34	0,34644	0,496	0,307	6,60705E-30	6	
Pvrl1	4,0322E-34	0,30381	0,376	0,197	6,77046E-30	6	
Nptxr	7,04249E-34	0,39976	0,605	0,423	1,1825E-29	6	
Eps15	1,29397E-33	0,38191	0,528	0,346	2,1727E-29	6	
Arid2	1,45353E-33	0,37387	0,592	0,407	2,44062E-29	6	
Rtn4rl1	1,88614E-33	0,35554	0,308	0,149	3,16701E-29	6	
Appbp2	1,94703E-33	0,38177	0,592	0,409	3,26926E-29	6	
Trim9	2,09946E-33	0,30884	0,362	0,19	3,5252E-29	6	

Zfp462	2,7906E-33	0,35755	0,516	0,325	4,68569E-29	6	
Pabpn1	2,9772E-33	0,39445	0,676	0,507	4,99902E-29	6	
Rai1	3,09021E-33	0,37157	0,577	0,393	5,18878E-29	6	
Tspan2	3,46996E-33	0,432	0,681	0,527	5,8264E-29	6	
Cux2	3,72034E-33	0,36339	0,74	0,581	6,24682E-29	6	
Vezt	3,80731E-33	0,40732	0,66	0,5	6,39285E-29	6	
Prkar2a	4,08602E-33	0,41461	0,705	0,55	6,86084E-29	6	
Nrsn1	4,52897E-33	0,3798	0,933	0,876	7,60459E-29	6	
Slc38a1	4,56271E-33	0,38561	0,686	0,522	7,66125E-29	6	
Slc29a4	4,76729E-33	0,39012	0,545	0,362	8,00475E-29	6	
Phip	4,93905E-33	0,38564	0,79	0,634	8,29316E-29	6	
Spen	5,37121E-33	0,33762	0,612	0,422	9,01879E-29	6	
Vstm2l	5,58245E-33	0,37016	0,827	0,721	9,37349E-29	6	
Nmnat2	5,73742E-33	0,30173	0,433	0,247	9,6337E-29	6	
Ss18	6,92871E-33	0,28234	0,336	0,17	1,1634E-28	6	
Lrrn1	7,7779E-33	0,31136	0,369	0,197	1,30599E-28	6	
Atp6v1a	8,96777E-33	0,43789	0,763	0,654	1,50578E-28	6	
Slc25a22	9,06737E-33	0,40335	0,697	0,541	1,5225E-28	6	
Hs3st5	1,037E-32	0,30544	0,378	0,201	1,74123E-28	6	
Slc44a1	1,14617E-32	0,33854	0,802	0,647	1,92454E-28	6	
Pgm2l1	1,40068E-32	0,44001	0,766	0,646	2,35189E-28	6	
Sh3kbp1	1,78505E-32	0,37393	0,585	0,399	2,99728E-28	6	
Pdpk1	1,80888E-32	0,39761	0,61	0,447	3,03729E-28	6	
Adgrl2	2,00472E-32	0,38859	0,585	0,405	3,36613E-28	6	
Rnf38	2,19087E-32	0,36587	0,52	0,341	3,6787E-28	6	
Megf8	2,27615E-32	0,362	0,497	0,316	3,82189E-28	6	
Far1	2,52731E-32	0,38443	0,713	0,559	4,24361E-28	6	
Dock10	2,54138E-32	0,44199	0,686	0,537	4,26723E-28	6	
Snx4	2,62088E-32	0,37044	0,655	0,485	4,40072E-28	6	
Rerg	3,60724E-32	0,25271	0,292	0,137	6,05692E-28	6	
Tmppe	3,79539E-32	0,25288	0,27	0,123	6,37285E-28	6	
Negr1	3,91394E-32	0,33243	0,991	0,964	6,5719E-28	6	
Mapre2	3,98074E-32	0,34314	0,903	0,817	6,68407E-28	6	
Hook1	4,04017E-32	0,33434	0,444	0,261	6,78386E-28	6	
Prrc1	4,18544E-32	0,3725	0,654	0,482	7,02777E-28	6	
Mxd4	4,34754E-32	0,36224	0,766	0,612	7,29996E-28	6	
Apbb2	4,55441E-32	0,33211	0,432	0,253	7,64731E-28	6	
Setd7	5,49041E-32	0,37341	0,612	0,441	9,21894E-28	6	
Aak1	5,9118E-32	0,36641	0,605	0,424	9,92651E-28	6	
Nufip2	6,09841E-32	0,34894	0,741	0,593	1,02398E-27	6	
Pum1	6,66969E-32	0,38973	0,683	0,538	1,11991E-27	6	
Mfsd6	6,95304E-32	0,36796	0,778	0,647	1,16749E-27	6	
D5Ertd579e	7,18181E-32	0,35015	0,591	0,409	1,2059E-27	6	
Nol4l	8,70878E-32	0,30431	0,549	0,371	1,46229E-27	6	
Grb2	9,17267E-32	0,32723	0,862	0,771	1,54018E-27	6	
Slc25a51	1,02804E-31	0,37785	0,736	0,579	1,72618E-27	6	
Fam120a	1,30123E-31	0,39511	0,604	0,435	2,18489E-27	6	
Synj1	1,54048E-31	0,35942	0,761	0,624	2,58663E-27	6	

Dmxl2	1,56984E-31	0,3855	0,729	0,575	2,63592E-27	6	
4932438A13	1,66068E-31	0,37877	0,784	0,672	2,78845E-27	6	
Syngr1	1,79883E-31	0,34898	0,883	0,757	3,02042E-27	6	
Clcn4	1,93285E-31	0,36631	0,775	0,651	3,24545E-27	6	
Gdap1	2,06158E-31	0,3861	0,737	0,612	3,4616E-27	6	
Zwint	2,09156E-31	0,29097	0,996	0,988	3,51194E-27	6	
Cnnm4	2,09723E-31	0,27873	0,357	0,189	3,52145E-27	6	
Pclo	2,46814E-31	0,34478	0,888	0,816	4,14426E-27	6	
Mob1b	2,56402E-31	0,31366	0,394	0,223	4,30525E-27	6	
Phc2	4,46498E-31	0,36638	0,655	0,502	7,49715E-27	6	
Prkce	4,63541E-31	0,35548	0,914	0,812	7,78332E-27	6	
Mgat5	4,68689E-31	0,39457	0,633	0,468	7,86976E-27	6	
Omg	4,94892E-31	0,32022	0,407	0,234	8,30974E-27	6	
Fstl5	4,9864E-31	0,34434	0,444	0,268	8,37266E-27	6	
Cmtm4	5,09537E-31	0,39136	0,577	0,41	8,55564E-27	6	
Zcchc7	6,13652E-31	0,35346	0,798	0,643	1,03038E-26	6	
Kdm6b	6,37569E-31	0,33842	0,527	0,34	1,07054E-26	6	
Cnot6	6,59108E-31	0,37564	0,626	0,456	1,10671E-26	6	
Epm2aip1	6,5984E-31	0,35503	0,765	0,649	1,10794E-26	6	
Lmbrd1	8,32902E-31	0,38469	0,659	0,519	1,39853E-26	6	
AW551984	9,61083E-31	0,32442	0,894	0,749	1,61375E-26	6	
Atxn7	1,25041E-30	0,28429	0,369	0,2	2,09956E-26	6	
Kdm7a	1,25286E-30	0,31728	0,441	0,261	2,10367E-26	6	
Spats2l	1,31234E-30	0,36198	0,703	0,522	2,20355E-26	6	
Rasal2	1,55789E-30	0,34208	0,494	0,315	2,61585E-26	6	
Ndrg4	1,56763E-30	0,35053	0,917	0,857	2,6322E-26	6	
Sgpp2	1,59223E-30	0,39025	0,583	0,413	2,67352E-26	6	
G3bp2	2,25679E-30	0,35682	0,891	0,8	3,78938E-26	6	
Larp1	2,73211E-30	0,33009	0,754	0,602	4,58749E-26	6	
Pgrmc1	2,77612E-30	0,36284	0,884	0,799	4,66139E-26	6	
Dnajb14	3,39875E-30	0,3778	0,679	0,536	5,70684E-26	6	
Cbfb	4,47296E-30	0,36867	0,576	0,411	7,51054E-26	6	
Nptn	5,84387E-30	0,2922	0,962	0,912	9,81244E-26	6	
Csnk1g1	6,29597E-30	0,38355	0,545	0,378	1,05716E-25	6	
Gm37494	6,94418E-30	0,30325	0,378	0,212	1,166E-25	6	
Xiap	7,35385E-30	0,38151	0,758	0,619	1,23478E-25	6	
Ep300	7,6773E-30	0,33408	0,624	0,441	1,2891E-25	6	
Gpr153	7,86825E-30	0,29529	0,408	0,232	1,32116E-25	6	
Ddr1	8,76972E-30	0,32776	0,605	0,428	1,47252E-25	6	
Mprip	1,14473E-29	0,33472	0,865	0,79	1,92211E-25	6	
Tollip	1,4684E-29	0,36508	0,713	0,574	2,46559E-25	6	
Adgrb1	1,6201E-29	0,37478	0,696	0,514	2,72031E-25	6	
Ppm1l	2,02334E-29	0,37233	0,465	0,296	3,39739E-25	6	
Cadps	2,0385E-29	0,32076	0,895	0,825	3,42284E-25	6	
Pcsk1	2,51234E-29	0,31218	0,822	0,636	4,21846E-25	6	
Fgfr1	2,87225E-29	0,3766	0,689	0,541	4,8228E-25	6	
Eif4g3	2,95122E-29	0,34681	0,908	0,838	4,95539E-25	6	
Plec	3,07685E-29	0,30778	0,933	0,852	5,16633E-25	6	

Dhcr24	3,30663E-29	0,36855	0,697	0,547	5,55216E-25	6	
Setd5	3,33763E-29	0,36709	0,699	0,561	5,60422E-25	6	
Chuk	4,1054E-29	0,3585	0,513	0,338	6,89337E-25	6	
Abcc1	4,12114E-29	0,3592	0,58	0,418	6,9198E-25	6	
Cbx7	5,1187E-29	0,2602	0,395	0,225	8,59482E-25	6	
Nedd4	5,21439E-29	0,26446	0,997	0,988	8,75548E-25	6	
Cers4	5,29768E-29	0,35004	0,888	0,789	8,89534E-25	6	
Wls	7,52252E-29	0,35728	0,561	0,379	1,26311E-24	6	
Clasp1	7,9338E-29	0,32685	0,711	0,549	1,33216E-24	6	
Tgoln1	8,62402E-29	0,34842	0,83	0,739	1,44806E-24	6	
Extl3	1,09551E-28	0,3156	0,496	0,319	1,83947E-24	6	
Mkl2	1,26551E-28	0,25434	0,298	0,151	2,12491E-24	6	
Dach1	1,3361E-28	0,25335	0,309	0,157	2,24345E-24	6	
Nfasc	1,73556E-28	0,33406	0,821	0,686	2,91418E-24	6	
Ankrd6	1,85351E-28	0,29545	0,351	0,192	3,11223E-24	6	
Zfp207	1,88512E-28	0,34496	0,729	0,581	3,1653E-24	6	
Pcnx	2,18541E-28	0,35987	0,51	0,344	3,66953E-24	6	
Dpp6	2,32181E-28	0,31992	0,867	0,756	3,89855E-24	6	
Gm21092	2,3272E-28	0,3531	0,587	0,431	3,90759E-24	6	
Dnajc5	2,39849E-28	0,34883	0,814	0,729	4,02731E-24	6	
March7	2,58385E-28	0,37307	0,591	0,438	4,33855E-24	6	
Zfp451	2,71516E-28	0,37415	0,65	0,507	4,55902E-24	6	
Gabbr1	2,84428E-28	0,3059	0,92	0,86	4,77584E-24	6	
Vcan	3,16048E-28	0,38175	0,359	0,198	5,30676E-24	6	
Tm4sf4	3,48918E-28	0,34698	0,523	0,34	5,85867E-24	6	
Dvl1	3,62106E-28	0,33147	0,556	0,387	6,08012E-24	6	
Ppp3cb	4,08033E-28	0,3363	0,811	0,691	6,85129E-24	6	
Ppm1f	4,45653E-28	0,27836	0,369	0,209	7,48297E-24	6	
Prdm2	4,56576E-28	0,35523	0,545	0,385	7,66637E-24	6	
Camsap1	5,03804E-28	0,3516	0,612	0,456	8,45937E-24	6	
Kat6b	5,13545E-28	0,33398	0,545	0,373	8,62294E-24	6	
Crabp1	5,89986E-28	0,31444	0,696	0,478	9,90646E-24	6	
Clic4	6,23798E-28	0,27118	0,35	0,193	1,04742E-23	6	
Smc5	6,8912E-28	0,35512	0,528	0,358	1,1571E-23	6	
Pja2	7,46348E-28	0,34582	0,89	0,824	1,25319E-23	6	
Mecp2	7,94853E-28	0,34461	0,737	0,612	1,33464E-23	6	
Sh3pxd2b	8,65822E-28	0,27327	0,312	0,164	1,4538E-23	6	
Syngr3	8,87338E-28	0,32047	0,895	0,817	1,48993E-23	6	
Ddi2	1,01854E-27	0,31162	0,423	0,26	1,71023E-23	6	
Alg2	1,08976E-27	0,39068	0,739	0,618	1,82982E-23	6	
Arfgef1	1,09367E-27	0,3221	0,803	0,691	1,83638E-23	6	
Syt2	1,1506E-27	0,29936	0,798	0,657	1,93198E-23	6	
Ssbp2	1,66345E-27	0,32783	0,903	0,865	2,7931E-23	6	
Zfp365	1,66958E-27	0,33338	0,423	0,265	2,80339E-23	6	
Rhoq	1,88201E-27	0,34166	0,547	0,385	3,16008E-23	6	
Unc5c	1,92619E-27	0,391	0,561	0,405	3,23427E-23	6	
Fitm2	1,94616E-27	0,37447	0,609	0,45	3,2678E-23	6	
Ccdc88a	1,95114E-27	0,3551	0,746	0,615	3,27616E-23	6	

Pfn2	2,00392E-27	0,31457	0,941	0,865	3,36477E-23	6	
Lpin2	2,12999E-27	0,38306	0,537	0,379	3,57646E-23	6	
Creb3l2	2,16665E-27	0,3445	0,448	0,287	3,63802E-23	6	
Map4	2,64661E-27	0,25912	0,992	0,975	4,44393E-23	6	
Phc3	2,81067E-27	0,31677	0,465	0,296	4,7194E-23	6	
Enah	2,81289E-27	0,27375	0,957	0,883	4,72313E-23	6	
Rock1	3,32799E-27	0,32589	0,59	0,422	5,58803E-23	6	
R3hdm4	3,43453E-27	0,3309	0,583	0,416	5,76692E-23	6	
Abhd2	3,4501E-27	0,29144	0,42	0,254	5,79306E-23	6	
Mapre1	3,50456E-27	0,35339	0,831	0,746	5,88451E-23	6	
Ap1ar	5,0189E-27	0,36702	0,491	0,34	8,42723E-23	6	
Rnf44	5,51059E-27	0,33196	0,58	0,408	9,25284E-23	6	
Micall1	8,08784E-27	0,27688	0,342	0,191	1,35803E-22	6	
Tab2	8,21835E-27	0,3422	0,545	0,38	1,37994E-22	6	
Eml4	9,4001E-27	0,32121	0,518	0,354	1,57837E-22	6	
5330434G04	9,60936E-27	0,32304	0,889	0,789	1,61351E-22	6	
Ankfy1	9,60939E-27	0,30818	0,597	0,425	1,61351E-22	6	
Tmcc1	1,01538E-26	0,33821	0,598	0,447	1,70493E-22	6	
Vamp2	1,0198E-26	0,26871	0,992	0,978	1,71234E-22	6	
Ubr3	1,08853E-26	0,34221	0,691	0,552	1,82775E-22	6	
Ncoa7	1,10736E-26	0,29824	0,913	0,787	1,85937E-22	6	
Adcy5	1,1591E-26	0,29741	0,431	0,269	1,94624E-22	6	
Me1	1,17232E-26	0,37252	0,683	0,546	1,96844E-22	6	
Iqgap1	1,2276E-26	0,31893	0,417	0,259	2,06126E-22	6	
Cpeb1	1,35049E-26	0,2843	0,413	0,248	2,2676E-22	6	
Rasa2	1,35519E-26	0,37419	0,624	0,49	2,27551E-22	6	
Selt	1,43466E-26	0,33843	0,691	0,553	2,40894E-22	6	
Ago3	1,4623E-26	0,33225	0,468	0,306	2,45536E-22	6	
Ankrd17	1,55988E-26	0,3584	0,807	0,717	2,6192E-22	6	
Zcchc11	1,58644E-26	0,34184	0,712	0,565	2,6638E-22	6	
Klhl32	1,65766E-26	0,28741	0,385	0,229	2,78337E-22	6	
Golim4	1,6654E-26	0,33905	0,553	0,391	2,79637E-22	6	
Aff4	1,81214E-26	0,28251	0,957	0,891	3,04277E-22	6	
L1cam	2,18831E-26	0,26611	0,992	0,981	3,67439E-22	6	
Kif5b	2,48606E-26	0,32306	0,895	0,817	4,17434E-22	6	
Nrg1	2,67109E-26	0,2606	0,354	0,2	4,48503E-22	6	
Golph3	2,67738E-26	0,31273	0,492	0,328	4,49558E-22	6	
Mpped2	2,84476E-26	0,26319	0,318	0,171	4,77663E-22	6	
Tmbim6	3,33899E-26	0,33814	0,821	0,741	5,60651E-22	6	
Dopey1	3,62957E-26	0,32906	0,631	0,474	6,09441E-22	6	
Pnmal2	3,68508E-26	0,31258	0,922	0,86	6,18763E-22	6	
Il6st	3,70761E-26	0,33478	0,561	0,401	6,22544E-22	6	
Add2	3,81408E-26	0,30733	0,837	0,739	6,40422E-22	6	
Dclk2	4,1407E-26	0,33196	0,634	0,466	6,95265E-22	6	
Esyt2	4,52165E-26	0,36181	0,6	0,451	7,5923E-22	6	
Slc7a14	4,89171E-26	0,26744	0,926	0,834	8,21367E-22	6	
Foxp1	5,01878E-26	0,37452	0,638	0,514	8,42704E-22	6	
Srgap3	6,01449E-26	0,31285	0,784	0,669	1,00989E-21	6	

Tnfaip1	6,26418E-26	0,36749	0,511	0,358	1,05182E-21	6	
Myo5a	6,39844E-26	0,30172	0,917	0,858	1,07436E-21	6	
Sh3gl2	7,05539E-26	0,32785	0,564	0,41	1,18467E-21	6	
Sidt2	8,5657E-26	0,29089	0,458	0,293	1,43827E-21	6	
Vasp	1,03109E-25	0,34703	0,596	0,46	1,73131E-21	6	
Zdhhc8	1,06212E-25	0,33252	0,703	0,551	1,78341E-21	6	
Irf2bp2	1,08353E-25	0,37069	0,628	0,48	1,81936E-21	6	
Abca5	1,17006E-25	0,355	0,609	0,472	1,96464E-21	6	
2010111I01R	1,23466E-25	0,34848	0,726	0,598	2,07312E-21	6	
Pbrm1	1,31417E-25	0,32138	0,851	0,757	2,20663E-21	6	
Scoc	1,39087E-25	0,29575	0,926	0,862	2,3354E-21	6	
Brwd1	1,53181E-25	0,29133	0,614	0,448	2,57205E-21	6	
St8sia3	1,71877E-25	0,35606	0,626	0,48	2,88599E-21	6	
Tet1	1,77432E-25	0,30553	0,475	0,313	2,97926E-21	6	
Gapvd1	2,34776E-25	0,32318	0,702	0,569	3,94212E-21	6	
Tyro3	2,69153E-25	0,34354	0,595	0,435	4,51935E-21	6	
Tmx3	2,72077E-25	0,31841	0,684	0,554	4,56844E-21	6	
Thoc2	2,97709E-25	0,33235	0,653	0,513	4,99882E-21	6	
Zcchc14	3,00723E-25	0,29964	0,508	0,342	5,04945E-21	6	
Kif3c	3,02649E-25	0,33754	0,678	0,551	5,08177E-21	6	
Smurf2	3,11376E-25	0,25751	0,35	0,2	5,22832E-21	6	
Setd1b	3,32515E-25	0,30726	0,516	0,351	5,58326E-21	6	
Cpd	4,14575E-25	0,29564	0,4	0,245	6,96112E-21	6	
Cd47	4,20788E-25	0,29285	0,973	0,931	7,06545E-21	6	
Srrm2	4,34658E-25	0,30037	0,976	0,969	7,29834E-21	6	
Spaca6	4,35938E-25	0,28338	0,375	0,221	7,31984E-21	6	
Zfp704	4,97842E-25	0,31225	0,514	0,352	8,35926E-21	6	
Atl3	5,18128E-25	0,36347	0,534	0,39	8,69989E-21	6	
Flna	5,68028E-25	0,31252	0,396	0,243	9,53776E-21	6	
Atl1	6,15497E-25	0,31843	0,816	0,719	1,03348E-20	6	
Rbm33	7,61673E-25	0,31756	0,479	0,321	1,27893E-20	6	
Picalm	9,53098E-25	0,31709	0,731	0,6	1,60035E-20	6	
Ylpm1	1,04109E-24	0,2963	0,638	0,482	1,74809E-20	6	
Arpc5	1,21176E-24	0,29533	0,939	0,888	2,03467E-20	6	
Ddx3x	2,03286E-24	0,31279	0,841	0,727	3,41338E-20	6	
Rgs7	2,11301E-24	0,27709	0,366	0,218	3,54796E-20	6	
Fgf1	2,11913E-24	0,32093	0,784	0,645	3,55822E-20	6	
Chka	2,14914E-24	0,33843	0,625	0,479	3,60862E-20	6	
Atxn7l3b	2,22071E-24	0,30397	0,928	0,878	3,72879E-20	6	
Cpsf6	3,32716E-24	0,32095	0,611	0,453	5,58663E-20	6	
Fam78b	3,57686E-24	0,33015	0,52	0,368	6,0059E-20	6	
March6	3,59806E-24	0,31264	0,799	0,68	6,04149E-20	6	
Slc9a6	3,88962E-24	0,31913	0,595	0,442	6,53105E-20	6	
Tbcel	4,31954E-24	0,31828	0,487	0,333	7,25294E-20	6	
Crebbp	4,88078E-24	0,33189	0,681	0,553	8,19531E-20	6	
Sptssa	6,06282E-24	0,3451	0,721	0,589	1,01801E-19	6	
D1Ertd622e	6,27242E-24	0,28836	0,364	0,219	1,0532E-19	6	
Mbd5	6,3954E-24	0,31592	0,545	0,394	1,07385E-19	6	

Nf1	7,02665E-24	0,30658	0,649	0,506	1,17985E-19	6	
Blcap	7,58276E-24	0,31442	0,494	0,343	1,27322E-19	6	
Cab39	7,95466E-24	0,33218	0,611	0,458	1,33567E-19	6	
Marveld1	8,29354E-24	0,28395	0,485	0,326	1,39257E-19	6	
Fam178a	8,48951E-24	0,32737	0,553	0,396	1,42547E-19	6	
Efr3b	8,59069E-24	0,345	0,564	0,425	1,44246E-19	6	
Pan3	8,64753E-24	0,29024	0,509	0,344	1,45201E-19	6	
Pdcd6ip	1,10337E-23	0,31019	0,593	0,45	1,85266E-19	6	
Ncor1	1,10952E-23	0,2969	0,855	0,768	1,86299E-19	6	
Nr4a3	1,23514E-23	0,33055	0,605	0,444	2,07392E-19	6	
Snrnp70	1,30506E-23	0,33093	0,864	0,78	2,19132E-19	6	
Canx	1,38027E-23	0,26777	0,982	0,964	2,31761E-19	6	
Sppl3	1,39076E-23	0,33854	0,682	0,557	2,33523E-19	6	
Ralb	1,47762E-23	0,25256	0,362	0,216	2,48107E-19	6	
Tex261	1,60242E-23	0,31678	0,604	0,469	2,69063E-19	6	
Oxct1	1,68059E-23	0,35214	0,777	0,691	2,82187E-19	6	
Sult4a1	1,75929E-23	0,25944	0,977	0,939	2,95402E-19	6	
Arhgap23	1,77128E-23	0,29881	0,501	0,345	2,97416E-19	6	
Secisbp2l	1,84431E-23	0,29481	0,568	0,409	3,09677E-19	6	
Fam53c	1,86473E-23	0,29942	0,534	0,377	3,13106E-19	6	
Id3	1,87957E-23	0,25782	0,664	0,45	3,15598E-19	6	
Tmem65	1,92451E-23	0,35918	0,711	0,596	3,23144E-19	6	
Evi5	1,97248E-23	0,31696	0,694	0,575	3,31198E-19	6	
Mllt10	2,2109E-23	0,30309	0,431	0,28	3,71232E-19	6	
Sh3bgrl	2,37413E-23	0,28963	0,676	0,512	3,9864E-19	6	
Trove2	2,43828E-23	0,33463	0,463	0,32	4,09412E-19	6	
Bicd2	2,77E-23	0,30013	0,476	0,322	4,6511E-19	6	
Tle2	2,94492E-23	0,29906	0,462	0,308	4,94481E-19	6	
Sbno1	3,43103E-23	0,32936	0,653	0,529	5,76104E-19	6	
Bean1	4,13123E-23	0,31993	0,617	0,476	6,93675E-19	6	
Alkbh5	4,33184E-23	0,28984	0,462	0,31	7,27359E-19	6	
Myadm	4,55358E-23	0,27642	0,913	0,831	7,64592E-19	6	
Scd1	4,65515E-23	0,31082	0,359	0,219	7,81646E-19	6	
Ndfip2	4,67048E-23	0,3313	0,706	0,609	7,84221E-19	6	
Rab6a	5,53939E-23	0,28209	0,952	0,903	9,30119E-19	6	
Syn3	6,04244E-23	0,27206	0,383	0,234	1,01459E-18	6	
Xpo7	6,11359E-23	0,2622	0,431	0,275	1,02653E-18	6	
Celf2	7,8751E-23	0,33744	0,475	0,33	1,32231E-18	6	
Dynll2	7,93453E-23	0,25899	0,991	0,973	1,33229E-18	6	
Smim13	8,61176E-23	0,3464	0,628	0,504	1,446E-18	6	
Xrn1	8,61229E-23	0,30676	0,556	0,412	1,44609E-18	6	
Luc7l3	9,08879E-23	0,29383	0,891	0,8	1,5261E-18	6	
Macf1	1,00048E-22	0,27573	0,903	0,821	1,6799E-18	6	
Klhl24	1,12372E-22	0,28662	0,489	0,333	1,88684E-18	6	
Samd4b	1,27003E-22	0,28516	0,516	0,363	2,13251E-18	6	
Ptprr	1,31296E-22	0,26661	0,885	0,798	2,20459E-18	6	
Kmt2c	1,35884E-22	0,31176	0,726	0,593	2,28162E-18	6	
Marcks	1,36696E-22	0,30377	0,932	0,892	2,29526E-18	6	

Gmfb	1,56805E-22	0,36313	0,653	0,553	2,6329E-18	6	
Zfp638	1,66759E-22	0,30125	0,716	0,602	2,80006E-18	6	
Abl2	1,9294E-22	0,29278	0,457	0,31	3,23965E-18	6	
Srrm3	2,06027E-22	0,32802	0,663	0,537	3,4594E-18	6	
Ncoa2	2,19572E-22	0,27768	0,572	0,421	3,68683E-18	6	
Tbpl1	2,49714E-22	0,30979	0,554	0,406	4,19294E-18	6	
Cspp1	2,66688E-22	0,35283	0,648	0,52	4,47796E-18	6	
Tub	2,86682E-22	0,25853	0,87	0,759	4,81367E-18	6	
Rubcn	3,25879E-22	0,316	0,523	0,381	5,47184E-18	6	
Ssr3	3,5035E-22	0,32533	0,593	0,461	5,88272E-18	6	
Mdm4	4,01501E-22	0,31127	0,574	0,425	6,74161E-18	6	
Ero1l	4,25806E-22	0,27777	0,456	0,309	7,14971E-18	6	
Capn5	4,8628E-22	0,31039	0,634	0,503	8,16513E-18	6	
Klhl9	4,96822E-22	0,29769	0,622	0,48	8,34214E-18	6	
Capza2	5,40451E-22	0,29318	0,86	0,774	9,07471E-18	6	
Msn	6,17061E-22	0,2587	0,946	0,873	1,03611E-17	6	
Atp11b	6,36744E-22	0,30871	0,563	0,424	1,06916E-17	6	
Surf4	7,01677E-22	0,30903	0,663	0,544	1,17819E-17	6	
Adgra1	7,1408E-22	0,2513	0,309	0,177	1,19901E-17	6	
Mapk14	7,27742E-22	0,27246	0,375	0,234	1,22195E-17	6	
Map4k4	7,53578E-22	0,31392	0,761	0,658	1,26533E-17	6	
Jph4	9,08658E-22	0,28761	0,937	0,902	1,52573E-17	6	
Fbxl17	1,00458E-21	0,28449	0,468	0,318	1,6868E-17	6	
Tmem8b	1,19472E-21	0,29329	0,444	0,301	2,00605E-17	6	
Ei24	1,26957E-21	0,30471	0,74	0,637	2,13174E-17	6	
Slc15a2	1,66782E-21	0,26159	0,312	0,178	2,80044E-17	6	
Pdzd8	1,78686E-21	0,33179	0,606	0,482	3,00032E-17	6	
Leprotl1	1,91731E-21	0,30885	0,746	0,654	3,21935E-17	6	
Tet3	1,95349E-21	0,28707	0,532	0,382	3,28011E-17	6	
Ankrd12	2,38942E-21	0,28512	0,888	0,83	4,01207E-17	6	
Adamts1	2,45526E-21	0,45855	0,725	0,611	4,12263E-17	6	
Ikbkb	2,71561E-21	0,2937	0,394	0,255	4,55978E-17	6	
N4bp2	2,84516E-21	0,25109	0,369	0,229	4,77732E-17	6	
Gng2	2,87521E-21	0,30381	0,867	0,795	4,82777E-17	6	
Mllt6	2,90868E-21	0,27366	0,557	0,404	4,88397E-17	6	
Samd8	2,94655E-21	0,29865	0,482	0,343	4,94756E-17	6	
Phf20l1	2,97381E-21	0,31653	0,659	0,549	4,99332E-17	6	
Aebp2	3,23724E-21	0,27976	0,503	0,357	5,43566E-17	6	
Astn1	3,45164E-21	0,27723	0,427	0,286	5,79564E-17	6	
Nr4a1	3,53741E-21	0,34589	0,418	0,277	5,93966E-17	6	
Fam160b1	4,18523E-21	0,27201	0,438	0,294	7,02742E-17	6	
Rnf13	4,65111E-21	0,30836	0,596	0,472	7,80968E-17	6	
Usp31	4,81462E-21	0,30892	0,548	0,407	8,08423E-17	6	
Rab3d	5,09321E-21	0,30443	0,689	0,553	8,55201E-17	6	
Pcmtd1	5,19717E-21	0,30588	0,731	0,641	8,72657E-17	6	
Pou2f1	5,8312E-21	0,2681	0,38	0,241	9,79117E-17	6	
Fam189b	5,83999E-21	0,28253	0,622	0,491	9,80592E-17	6	
Slc25a36	6,14048E-21	0,31978	0,524	0,384	1,03105E-16	6	

Hipk1	6,15831E-21	0,28422	0,674	0,542	1,03404E-16	6	
Atf4	6,19013E-21	0,25679	0,944	0,891	1,03938E-16	6	
Tmx4	6,52225E-21	0,26567	0,898	0,835	1,09515E-16	6	
Aftph	8,4421E-21	0,30582	0,532	0,392	1,41751E-16	6	
4833439L19I	9,33231E-21	0,3355	0,673	0,58	1,56699E-16	6	
Pds5a	9,73636E-21	0,30356	0,534	0,399	1,63483E-16	6	
Epn2	9,97364E-21	0,29856	0,727	0,619	1,67467E-16	6	
Tsc1	1,04525E-20	0,2593	0,427	0,282	1,75507E-16	6	
Pcmtd2	1,1101E-20	0,268	0,452	0,306	1,86396E-16	6	
Nek7	1,27872E-20	0,30029	0,832	0,74	2,1471E-16	6	
Pacsin1	1,29964E-20	0,28466	0,768	0,665	2,18222E-16	6	
Ykt6	1,34484E-20	0,30503	0,573	0,438	2,25812E-16	6	
Map3k2	1,37285E-20	0,30321	0,495	0,359	2,30515E-16	6	
Trip12	1,68404E-20	0,26979	0,76	0,656	2,82767E-16	6	
Wdr26	1,76108E-20	0,34048	0,605	0,498	2,95702E-16	6	
Plekha1	1,77832E-20	0,28391	0,619	0,488	2,98598E-16	6	
Baz2b	1,7875E-20	0,31149	0,679	0,562	3,00139E-16	6	
Zdhhc9	1,86296E-20	0,28138	0,438	0,3	3,1281E-16	6	
Klf6	1,87052E-20	0,30549	0,563	0,426	3,14078E-16	6	
Synrg	2,22662E-20	0,2978	0,739	0,65	3,73871E-16	6	
Pxdn	2,31983E-20	0,25376	0,362	0,226	3,89523E-16	6	
Plxnd1	2,35046E-20	0,27134	0,362	0,228	3,94666E-16	6	
Nav2	2,38392E-20	0,27021	0,308	0,18	4,00284E-16	6	
Rnf157	3,04836E-20	0,2791	0,572	0,428	5,11849E-16	6	
Fam73a	3,10293E-20	0,30498	0,542	0,418	5,21012E-16	6	
Pgap1	3,2709E-20	0,25344	0,39	0,252	5,49217E-16	6	
Hnrnph1	3,61947E-20	0,29894	0,824	0,733	6,07746E-16	6	
Safb2	3,7707E-20	0,29562	0,49	0,351	6,33138E-16	6	
Serinc5	4,00372E-20	0,27693	0,447	0,306	6,72264E-16	6	
Itfg1	4,18746E-20	0,30189	0,638	0,515	7,03116E-16	6	
Bbx	4,48109E-20	0,30028	0,523	0,384	7,5242E-16	6	
Zfp280d	5,25968E-20	0,3037	0,571	0,43	8,83153E-16	6	
Rabgap1l	5,52814E-20	0,27354	0,405	0,271	9,28229E-16	6	
Rnf11	5,60227E-20	0,34276	0,684	0,595	9,40677E-16	6	
Actr2	5,64757E-20	0,33949	0,689	0,606	9,48283E-16	6	
Gclc	6,41886E-20	0,26575	0,37	0,236	1,07779E-15	6	
Klhdc10	6,74385E-20	0,28648	0,711	0,591	1,13236E-15	6	
Csrnp3	8,94484E-20	0,26433	0,375	0,24	1,50193E-15	6	
mt-Nd6	1,08834E-19	0,28246	0,444	0,308	1,82744E-15	6	
Clock	1,2027E-19	0,27635	0,66	0,539	2,01945E-15	6	
Astn2	1,41481E-19	0,25693	0,5	0,347	2,37561E-15	6	
Frrs1l	1,45991E-19	0,30011	0,472	0,34	2,45134E-15	6	
Phf21a	1,48357E-19	0,26264	0,504	0,359	2,49106E-15	6	
Psme4	1,54074E-19	0,29164	0,548	0,416	2,58705E-15	6	
Ttl	1,76524E-19	0,31055	0,604	0,482	2,96402E-15	6	
Adipor1	1,98587E-19	0,31903	0,677	0,592	3,33448E-15	6	
Zranb1	1,99247E-19	0,29011	0,616	0,492	3,34555E-15	6	
Mex3d	2,01715E-19	0,25739	0,385	0,252	3,387E-15	6	

Trpm7	2,15382E-19	0,27475	0,508	0,372	3,61648E-15	6	
Mob4	2,35257E-19	0,3109	0,614	0,505	3,9502E-15	6	
Ints3	2,52158E-19	0,2856	0,628	0,491	4,23399E-15	6	
Zc2hc1a	2,80496E-19	0,29439	0,558	0,44	4,70981E-15	6	
Usp29	3,00481E-19	0,28958	0,797	0,708	5,04537E-15	6	
Ccnt1	3,28948E-19	0,27762	0,497	0,36	5,52337E-15	6	
Stard7	3,58956E-19	0,26905	0,604	0,468	6,02724E-15	6	
Nf2	4,19829E-19	0,2629	0,495	0,355	7,04935E-15	6	
Nr2c2	4,38313E-19	0,30605	0,612	0,497	7,35972E-15	6	
Pom121	4,39274E-19	0,25656	0,439	0,302	7,37585E-15	6	
Rnasel	4,49303E-19	0,27556	0,516	0,372	7,54425E-15	6	
Dgke	4,78067E-19	0,26031	0,398	0,266	8,02722E-15	6	
Cdipt	5,61066E-19	0,2891	0,814	0,721	9,42085E-15	6	
Dpp9	5,64566E-19	0,26899	0,501	0,362	9,47963E-15	6	
Fam193a	5,78851E-19	0,29302	0,607	0,487	9,71948E-15	6	
Ppp2r5b	5,97192E-19	0,30069	0,697	0,594	1,00274E-14	6	
Pld5	6,38436E-19	0,34935	0,537	0,413	1,072E-14	6	
Coro2a	6,73058E-19	0,28805	0,668	0,547	1,13013E-14	6	
Rcan3	7,0764E-19	0,3189	0,645	0,555	1,1882E-14	6	
Usp7	7,36517E-19	0,29756	0,639	0,526	1,23669E-14	6	
Ssh2	7,63679E-19	0,26111	0,418	0,283	1,28229E-14	6	
Slc12a6	7,67525E-19	0,26806	0,479	0,341	1,28875E-14	6	
Dcaf8	8,61237E-19	0,27688	0,636	0,521	1,4461E-14	6	
Atp13a3	9,07366E-19	0,25245	0,475	0,334	1,52356E-14	6	
Akt3	1,1523E-18	0,27439	0,578	0,437	1,93483E-14	6	
Creb1	1,15846E-18	0,29177	0,578	0,453	1,94517E-14	6	
Stat2	1,16394E-18	0,26479	0,388	0,257	1,95437E-14	6	
Cep170	1,24221E-18	0,29469	0,692	0,593	2,0858E-14	6	
Arxes2	1,28511E-18	0,31908	0,514	0,39	2,15783E-14	6	
Ppip5k2	1,32766E-18	0,25052	0,386	0,254	2,22928E-14	6	
Sos2	1,7869E-18	0,25858	0,508	0,373	3,00039E-14	6	
Irs2	2,05845E-18	0,28536	0,48	0,349	3,45634E-14	6	
Pex5l	2,18701E-18	0,26473	0,582	0,441	3,6722E-14	6	
Fam219a	2,46911E-18	0,25248	0,568	0,43	4,14588E-14	6	
Rab8b	2,69587E-18	0,29692	0,668	0,566	4,52664E-14	6	
Dynlt3	3,54582E-18	0,26012	0,923	0,88	5,95379E-14	6	
Tra2a	4,05835E-18	0,27017	0,739	0,634	6,81437E-14	6	
Paxbp1	4,21432E-18	0,26604	0,457	0,322	7,07626E-14	6	
Rmnd5a	4,35025E-18	0,26615	0,605	0,483	7,30451E-14	6	
Fktn	4,49208E-18	0,26738	0,396	0,266	7,54266E-14	6	
Ube2j1	4,54654E-18	0,25567	0,716	0,6	7,63409E-14	6	
Cnot6l	4,81224E-18	0,29956	0,497	0,371	8,08024E-14	6	
Tnpo2	4,96404E-18	0,27584	0,48	0,35	8,33513E-14	6	
Lypla2	5,09724E-18	0,27391	0,581	0,453	8,55877E-14	6	
Slc41a2	5,22035E-18	0,26492	0,391	0,265	8,76549E-14	6	
Usp24	5,40817E-18	0,2519	0,447	0,313	9,08086E-14	6	
Kdm2a	5,42985E-18	0,27431	0,655	0,525	9,11727E-14	6	
Eif4e3	6,2555E-18	0,28035	0,485	0,359	1,05036E-13	6	

Aldh3a2	6,39453E-18	0,25299	0,434	0,302	1,0737E-13	6	
Rfx7	6,55535E-18	0,25384	0,409	0,276	1,10071E-13	6	
Actn4	8,16885E-18	0,2536	0,804	0,695	1,37163E-13	6	
Gria4	8,36803E-18	0,28163	0,412	0,287	1,40508E-13	6	
Bsn	8,41628E-18	0,27332	0,635	0,515	1,41318E-13	6	
Lrrc55	8,53745E-18	0,274	0,415	0,289	1,43352E-13	6	
Mapk1	9,16394E-18	0,26192	0,83	0,759	1,53872E-13	6	
Bace1	1,03479E-17	0,2592	0,705	0,591	1,73752E-13	6	
Birc6	1,03649E-17	0,28248	0,751	0,668	1,74038E-13	6	
Clcn6	1,05229E-17	0,28598	0,62	0,494	1,76691E-13	6	
Crk	1,33273E-17	0,30986	0,545	0,434	2,23778E-13	6	
Cep350	1,49479E-17	0,25107	0,567	0,426	2,50991E-13	6	
Atrn	1,53088E-17	0,25247	0,527	0,389	2,57051E-13	6	
Bcl2l2	1,57625E-17	0,28447	0,487	0,36	2,64669E-13	6	
Ncdn	1,77623E-17	0,29271	0,665	0,579	2,98247E-13	6	
Coro1c	1,86903E-17	0,26401	0,701	0,58	3,13829E-13	6	
Trim56	1,90309E-17	0,2776	0,527	0,407	3,19548E-13	6	
Nalcn	1,96753E-17	0,289	0,525	0,41	3,30367E-13	6	
Zc3h7b	2,08196E-17	0,25313	0,633	0,505	3,49582E-13	6	
Srsf2	2,36444E-17	0,27978	0,758	0,662	3,97013E-13	6	
Npepps	2,53997E-17	0,28688	0,558	0,437	4,26486E-13	6	
Gpr45	2,57246E-17	0,2586	0,422	0,294	4,31942E-13	6	
Csnk1e	2,95234E-17	0,25339	0,814	0,73	4,95727E-13	6	
Gm4076	2,98475E-17	0,36973	0,452	0,332	5,0117E-13	6	
Ccnl2	3,01293E-17	0,30243	0,533	0,418	5,05901E-13	6	
Azin2	3,26789E-17	0,26778	0,696	0,598	5,48712E-13	6	
Exoc2	3,56012E-17	0,25845	0,441	0,316	5,97779E-13	6	
Kdelr2	3,72457E-17	0,2739	0,622	0,512	6,25393E-13	6	
Hunk	4,51431E-17	0,27917	0,554	0,428	7,57998E-13	6	
Csnk1g2	4,7257E-17	0,27333	0,761	0,685	7,93492E-13	6	
Dgcr2	4,76826E-17	0,26055	0,581	0,451	8,00638E-13	6	
Arhgap21	4,99443E-17	0,26227	0,655	0,545	8,38614E-13	6	
Scamp1	5,16826E-17	0,28656	0,811	0,727	8,67803E-13	6	
Map2k4	5,63288E-17	0,27364	0,544	0,424	9,45816E-13	6	
Nploc4	5,63438E-17	0,25456	0,467	0,342	9,46069E-13	6	
Csnk2a1	6,24441E-17	0,27733	0,566	0,452	1,0485E-12	6	
Tardbp	7,54599E-17	0,27885	0,725	0,62	1,26705E-12	6	
Mapk9	7,83655E-17	0,27431	0,587	0,473	1,31584E-12	6	
Tm9sf2	8,0756E-17	0,27927	0,665	0,575	1,35597E-12	6	
Wdr1	8,60393E-17	0,27916	0,662	0,555	1,44469E-12	6	
Qdpr	8,8493E-17	0,26302	0,669	0,553	1,48589E-12	6	
Mef2d	1,06575E-16	0,26877	0,535	0,414	1,78951E-12	6	
Icmt	1,0832E-16	0,27377	0,562	0,438	1,8188E-12	6	
Ank3	1,15337E-16	0,31928	0,573	0,465	1,93662E-12	6	
Rgs17	1,17832E-16	0,29201	0,582	0,466	1,97852E-12	6	
Zfp9	1,2428E-16	0,26866	0,422	0,302	2,08679E-12	6	
Lrrc8a	1,24652E-16	0,25116	0,534	0,399	2,09303E-12	6	
Tra2b	1,35226E-16	0,27393	0,681	0,595	2,27059E-12	6	

Lcorl	1,3627E-16	0,26257	0,388	0,263	2,28811E-12	6	
Plppr4	1,45584E-16	0,26675	0,688	0,586	2,4445E-12	6	
Ulk2	1,60476E-16	0,26752	0,547	0,427	2,69456E-12	6	
Ube4a	1,86677E-16	0,26329	0,535	0,413	3,13449E-12	6	
Atf2	1,95101E-16	0,26966	0,563	0,448	3,27595E-12	6	
Strn3	1,9646E-16	0,27852	0,634	0,532	3,29876E-12	6	
Rhob	2,13521E-16	0,28032	0,543	0,431	3,58523E-12	6	
Tcf12	2,38665E-16	0,27192	0,54	0,42	4,00742E-12	6	
Them4	2,48327E-16	0,30007	0,597	0,498	4,16966E-12	6	
1700020I14F	2,65172E-16	0,25739	0,494	0,371	4,4525E-12	6	
Cdk16	2,79019E-16	0,26232	0,624	0,516	4,68502E-12	6	
R3hdm2	3,78445E-16	0,25328	0,775	0,682	6,35448E-12	6	
Akap6	4,07307E-16	0,25037	0,766	0,673	6,83909E-12	6	
Slc43a2	4,496E-16	0,25517	0,509	0,383	7,54923E-12	6	
Eif4h	5,23662E-16	0,25633	0,794	0,728	8,79281E-12	6	
Ube2d2a	5,6739E-16	0,28846	0,703	0,629	9,52704E-12	6	
Enpp4	5,83975E-16	0,25122	0,508	0,38	9,80553E-12	6	
Daam1	6,34084E-16	0,28246	0,54	0,433	1,06469E-11	6	
Acaca	6,36566E-16	0,26558	0,61	0,496	1,06886E-11	6	
Rusc1	7,04952E-16	0,27316	0,559	0,45	1,18369E-11	6	
Eif5a2	7,39194E-16	0,25176	0,434	0,313	1,24118E-11	6	
Arih1	7,81648E-16	0,25714	0,523	0,396	1,31246E-11	6	
Pip5k1c	1,06673E-15	0,28075	0,587	0,479	1,79115E-11	6	
Tnrc18	1,07378E-15	0,28226	0,457	0,339	1,80298E-11	6	
Hipk3	1,19933E-15	0,25778	0,514	0,39	2,01379E-11	6	
9530068E07	1,68636E-15	0,27276	0,612	0,515	2,83157E-11	6	
Pcgf1	1,72216E-15	0,27648	0,588	0,477	2,89168E-11	6	
Slc48a1	1,91976E-15	0,26032	0,63	0,526	3,22346E-11	6	
Sc5d	2,00277E-15	0,29588	0,558	0,456	3,36286E-11	6	
Exoc5	2,23893E-15	0,26311	0,501	0,383	3,75939E-11	6	
Dock7	2,3519E-15	0,25826	0,529	0,416	3,94907E-11	6	
Samhd1	2,60901E-15	0,26087	0,424	0,311	4,38079E-11	6	
Ppp1r12b	2,86296E-15	0,25853	0,49	0,373	4,8072E-11	6	
Elavl2	3,01074E-15	0,30856	0,453	0,344	5,05534E-11	6	
Galnt1	3,34385E-15	0,27336	0,549	0,443	5,61465E-11	6	
Dixdc1	4,81203E-15	0,26663	0,614	0,513	8,07988E-11	6	
Atp2b1	5,61066E-15	0,27095	0,806	0,749	9,42086E-11	6	
Abi2	6,2471E-15	0,25857	0,561	0,455	1,04895E-10	6	
Fam134b	6,91026E-15	0,26405	0,66	0,571	1,1603E-10	6	
Skil	7,22107E-15	0,25249	0,576	0,464	1,21249E-10	6	
Trim25	8,99305E-15	0,25468	0,583	0,476	1,51002E-10	6	
Phtf1	9,45497E-15	0,25059	0,455	0,343	1,58758E-10	6	
Ssr1	1,13684E-14	0,25454	0,735	0,659	1,90886E-10	6	
Tsn	2,03534E-14	0,25017	0,813	0,759	3,41754E-10	6	
Slc29a1	2,61809E-14	0,2583	0,747	0,662	4,39603E-10	6	
Brsk2	2,74649E-14	0,25763	0,622	0,523	4,61163E-10	6	
Trp53inp2	4,05289E-14	0,25223	0,634	0,527	6,80521E-10	6	
Sike1	4,22249E-14	0,26973	0,467	0,365	7,08998E-10	6	

Fubp1	4,5322E-14	0,26228	0,721	0,633	7,61002E-10	6	
Sacs	4,55744E-14	0,26396	0,605	0,495	7,65239E-10	6	
5730455P16	7,68821E-14	0,25783	0,448	0,348	1,29093E-09	6	
Dusp1	1,01648E-13	0,25893	0,351	0,241	1,70677E-09	6	
Gatad1	1,02714E-13	0,25941	0,652	0,57	1,72467E-09	6	
Usp32	1,16799E-13	0,2552	0,549	0,455	1,96118E-09	6	
Mff	1,73003E-13	0,25679	0,698	0,652	2,90489E-09	6	
Cep120	2,428E-13	0,26162	0,501	0,407	4,07685E-09	6	
Kdm5a	3,45067E-13	0,26091	0,548	0,441	5,79402E-09	6	
Serpinb9	5,0121E-13	0,25081	0,419	0,318	8,41582E-09	6	
Zdhhc21	7,44071E-13	0,28021	0,72	0,659	1,24937E-08	6	
Hsd17b7	5,32345E-12	0,25136	0,48	0,381	8,93861E-08	6	
Setx	1,0693E-11	0,25378	0,516	0,427	1,79546E-07	6	
Egr1	1,63452E-10	0,34229	0,463	0,383	2,74453E-06	6	
Fst	4,31063E-10	0,26336	0,41	0,316	7,23799E-06	6	
Xist	0,000161773	0,75656	0,316	0,299	1	6	
Nnat	0,000175149	0,2548	0,542	0,499	1	6	
Dbh	0	2,62626	0,949	0,642	0	7	smENC3b
Scgn	0	1,52646	1	0,857	0	7	
Cd24a	0	1,38975	0,987	0,584	0	7	
Fibcd1	0	1,37733	0,915	0,334	0	7	
Npr1	0	1,15876	0,788	0,287	0	7	
Ntsr1	2,9454E-290	1,0126	0,734	0,283	4,9456E-286	7	
Hap1	1,758E-280	0,93031	0,991	0,846	2,9519E-276	7	
Thy1	7,9516E-266	1,08479	0,914	0,491	1,3352E-261	7	
Akap12	7,1171E-256	0,81022	0,992	0,923	1,195E-251	7	
Gfra1	5,7821E-253	1,04587	0,873	0,412	9,7088E-249	7	
Etv1	6,6024E-253	1,00736	0,999	0,566	1,1086E-248	7	
Lamc3	4,0548E-250	0,80631	0,688	0,241	6,8084E-246	7	
Ptgfrn	4,393E-248	0,58912	0,488	0,111	7,3763E-244	7	
Gabre	1,7487E-247	0,97544	0,514	0,135	2,9362E-243	7	
Asic2	8,5247E-244	0,86358	0,958	0,741	1,4314E-239	7	
Pxylp1	1,0908E-242	0,9181	0,929	0,628	1,8315E-238	7	
Moxd1	4,6652E-242	0,96286	0,911	0,445	7,8334E-238	7	
F2r	2,0869E-240	0,96662	0,932	0,535	3,5041E-236	7	
Galnt6	2,0577E-237	0,78478	0,557	0,161	3,4551E-233	7	
Ece1	8,1146E-237	0,86556	0,945	0,707	1,3625E-232	7	
Pwwp2b	1,4948E-230	0,73157	0,685	0,271	2,5099E-226	7	
Kcnj3	3,0766E-230	0,7469	0,641	0,22	5,166E-226	7	
Npy	1,8352E-227	1,10485	0,999	0,841	3,0814E-223	7	
Alcam	3,1074E-226	0,92677	0,95	0,578	5,2177E-222	7	
Th	2,9287E-220	1,50103	0,668	0,264	4,9176E-216	7	
Stxbp5	6,0964E-215	0,76884	0,941	0,649	1,0236E-210	7	
Tmem130	2,4795E-214	0,97458	0,857	0,527	4,1634E-210	7	
Gm42418	3,2267E-209	0,81388	1	1	5,418E-205	7	
Tmod1	9,9885E-199	0,84197	0,886	0,609	1,6772E-194	7	
Ptprn	1,9492E-198	0,57142	1	0,999	3,2729E-194	7	
Prkar1b	1,0959E-197	0,75866	0,955	0,802	1,8402E-193	7	

Synpo2	1,9116E-193	0,54498	0,495	0,144	3,2098E-189	7	
Thsd7a	4,2877E-191	0,68228	0,599	0,217	7,1994E-187	7	
Nrsn2	4,1374E-189	0,81996	0,536	0,187	6,9471E-185	7	
Pdlim5	8,5752E-188	0,72208	0,714	0,329	1,4399E-183	7	
Gpr153	1,4614E-185	0,57662	0,557	0,191	2,4539E-181	7	
Kcnab2	8,7146E-184	0,74305	0,719	0,346	1,4633E-179	7	
Prokr1	2,1328E-182	0,5845	0,546	0,183	3,5812E-178	7	
Spock3	1,338E-181	0,7985	0,913	0,571	2,2466E-177	7	
Ngfr	3,3963E-181	0,72576	0,939	0,512	5,7027E-177	7	
Spock1	5,2977E-181	0,71267	0,615	0,243	8,8954E-177	7	
Fbn1	4,2286E-179	0,6185	0,658	0,266	7,1002E-175	7	
Adcyap1r1	1,6411E-176	0,65912	0,751	0,367	2,7556E-172	7	
Cntnap5a	5,8794E-173	0,66964	0,978	0,632	9,872E-169	7	
Rimbp2	2,7063E-171	0,5612	0,63	0,248	4,5441E-167	7	
Sod1	2,1581E-170	0,70922	0,977	0,919	3,6237E-166	7	
Parva	1,7317E-163	0,68729	0,96	0,837	2,9077E-159	7	
Pvrl1	3,7319E-156	0,49021	0,486	0,164	6,2662E-152	7	
Tgfb1	1,3087E-155	0,52673	0,7	0,292	2,1975E-151	7	
Gpr149	3,4331E-155	0,59708	0,714	0,326	5,7645E-151	7	
Chl1	4,0551E-155	0,6579	0,955	0,748	6,809E-151	7	
Slco3a1	2,4231E-153	0,53667	0,604	0,245	4,0686E-149	7	
Ncam1	1,9563E-152	0,43542	1	0,999	3,2849E-148	7	
Fam155a	2,4697E-152	0,56606	0,724	0,35	4,1469E-148	7	
Slc18a2	2,9199E-151	0,62308	0,544	0,205	4,9028E-147	7	
Camk4	6,7788E-150	0,70121	0,862	0,546	1,1382E-145	7	
Kcnd2	1,7083E-147	0,56474	0,744	0,355	2,8684E-143	7	
Alpl	1,3962E-146	0,63956	0,517	0,196	2,3444E-142	7	
Entpd3	4,8154E-146	0,62825	0,718	0,367	8,0855E-142	7	
Vip	2,9124E-145	0,8224	1	0,919	4,8902E-141	7	
Syp	2,9698E-143	0,57	0,993	0,971	4,9865E-139	7	
Insm1	2,8213E-142	0,51053	0,559	0,219	4,7372E-138	7	
Csrp1	1,0011E-139	0,63588	0,99	0,966	1,6809E-135	7	
Eef1a2	1,0792E-136	0,52519	0,998	0,957	1,8121E-132	7	
Ehd3	1,3052E-136	0,69321	0,858	0,625	2,1916E-132	7	
Ascl1	1,0493E-133	0,51423	0,554	0,224	1,7619E-129	7	
Chst8	4,9461E-133	0,45661	0,463	0,166	8,305E-129	7	
Grin1	1,549E-131	0,60884	0,85	0,571	2,601E-127	7	
Msn	3,1258E-131	0,55281	0,969	0,863	5,2486E-127	7	
Fxyd5	1,6266E-130	0,58042	0,767	0,392	2,7313E-126	7	
Actb	3,9826E-130	0,41931	1	1	6,6871E-126	7	
Kcnj5	4,4173E-128	0,40967	0,439	0,151	7,417E-124	7	
Pnmal2	6,1068E-128	0,59987	0,956	0,849	1,0254E-123	7	
Astn2	8,4734E-127	0,56363	0,628	0,312	1,4228E-122	7	
Dclk2	3,2277E-126	0,55601	0,743	0,434	5,4196E-122	7	
Neurl1a	2,1767E-124	0,63396	0,883	0,712	3,6549E-120	7	
Ptger4	4,0736E-123	0,57144	0,748	0,423	6,8399E-119	7	
Kcnt2	9,8066E-122	0,44749	0,642	0,29	1,6466E-117	7	
Tmem108	1,5772E-121	0,47227	0,589	0,261	2,6483E-117	7	

Rasl10b	1,8738E-119	0,53885	0,697	0,39	3,1463E-115	7	
Sertm1	4,6297E-119	0,49554	0,508	0,209	7,7738E-115	7	
Ptpro	4,3102E-117	0,31836	0,251	0,057	7,2372E-113	7	
Hpca	2,8821E-116	0,49807	0,677	0,333	4,8394E-112	7	
Arhgap22	7,016E-116	0,51724	0,651	0,347	1,1781E-111	7	
Gm13889	8,9935E-116	0,46574	0,733	0,356	1,5101E-111	7	
Arvcf	1,4881E-113	0,56525	0,867	0,653	2,4987E-109	7	
Tspan12	1,2049E-112	0,44324	0,478	0,193	2,0231E-108	7	
Enpp1	2,8639E-111	0,45498	0,602	0,288	4,8088E-107	7	
Dach1	4,6618E-111	0,37229	0,385	0,132	7,8277E-107	7	
Myo16	6,7392E-111	0,31785	0,321	0,094	1,1316E-106	7	
Rab5b	3,2983E-109	0,53973	0,876	0,663	5,5381E-105	7	
Auts2	3,5274E-109	0,53447	0,917	0,65	5,9228E-105	7	
Ank2	3,9474E-109	0,3661	1	0,997	6,6282E-105	7	
Apbb1	1,0339E-108	0,50901	0,978	0,937	1,736E-104	7	
Man2a1	4,1326E-108	0,50667	0,957	0,829	6,939E-104	7	
Socs2	1,903E-107	0,45164	0,664	0,332	3,1954E-103	7	
Ttc39b	3,148E-106	0,47778	0,608	0,308	5,2859E-102	7	
Ptprg	3,3468E-105	0,50918	0,656	0,362	5,6196E-101	7	
F2rl2	5,638E-105	0,38579	0,447	0,175	9,4667E-101	7	
Map1b	4,3568E-104	0,32962	1	1	7,3154E-100	7	
Cpm	1,3743E-103	0,38425	0,401	0,148	2,3075E-99	7	
Tbx3	1,6552E-103	0,44759	0,997	0,938	2,7793E-99	7	
Cntnap2	1,9398E-103	0,46502	0,597	0,3	3,2571E-99	7	
Ptprz1	3,5237E-103	0,43455	0,583	0,269	5,9167E-99	7	
Rassf5	8,7381E-103	0,39253	0,472	0,199	1,46721E-98	7	
Pde3a	1,2302E-102	0,49251	0,718	0,416	2,06565E-98	7	
Pbxip1	9,3585E-102	0,42259	0,433	0,174	1,57139E-97	7	
Gria3	4,2944E-101	0,39063	0,543	0,24	7,21072E-97	7	
Syt7	8,6326E-101	0,48603	0,949	0,814	1,4495E-96	7	
Enpp2	1,2898E-100	0,39623	0,428	0,17	2,16564E-96	7	
Chst15	1,5876E-100	0,38629	0,464	0,189	2,66581E-96	7	
Tubb3	1,95403E-98	0,3491	1	0,999	3,28102E-94	7	
Mtss1	4,24812E-98	0,50094	0,698	0,423	7,13302E-94	7	
Ppp6r1	8,04153E-98	0,49265	0,795	0,537	1,35025E-93	7	
Ugcg	4,44151E-96	0,47905	0,918	0,763	7,45773E-92	7	
Nrp1	8,8571E-96	0,48322	0,941	0,77	1,4872E-91	7	
Atp1b3	8,07754E-95	0,50664	0,974	0,918	1,3563E-90	7	
Sh3kbp1	1,34711E-94	0,48082	0,647	0,374	2,26193E-90	7	
Sv2b	1,57163E-94	0,39907	0,483	0,214	2,63892E-90	7	
Spats2l	1,3652E-93	0,48691	0,769	0,496	2,29231E-89	7	
Plekhb2	3,22828E-93	0,64352	0,832	0,686	5,4206E-89	7	
Ap2s1	1,09296E-92	0,42263	0,985	0,932	1,83519E-88	7	
Clmn	1,98471E-92	0,43579	0,758	0,458	3,33252E-88	7	
Fscn1	4,00328E-92	0,50167	0,83	0,617	6,72191E-88	7	
Aplp2	7,63103E-92	0,42105	0,998	0,989	1,28133E-87	7	
Sept9	3,43745E-91	0,32772	0,332	0,116	5,77182E-87	7	
Ptpru	6,59308E-91	0,30488	0,343	0,12	1,10704E-86	7	

Man1a	5,48191E-90	0,38784	0,532	0,251	9,20468E-86	7	
Igfbp7	9,64473E-90	0,35893	0,716	0,35	1,61945E-85	7	
Lmna	8,8051E-89	0,44503	0,963	0,864	1,47846E-84	7	
Rnf128	2,50746E-88	0,28422	0,256	0,075	4,21028E-84	7	
Ccdc80	8,0329E-88	0,46212	0,563	0,299	1,3488E-83	7	
Trpm2	4,69402E-87	0,37241	0,441	0,195	7,88174E-83	7	
Tanc1	6,10682E-87	0,29248	0,315	0,108	1,0254E-82	7	
Slit2	7,80886E-87	0,44452	0,665	0,387	1,31119E-82	7	
Pld3	1,15825E-86	0,51719	0,922	0,813	1,94482E-82	7	
Ctsd	5,13554E-86	0,47708	0,919	0,837	8,62309E-82	7	
Cald1	3,60406E-85	0,41315	0,546	0,286	6,05158E-81	7	
Mmd	4,05272E-85	0,54299	0,908	0,807	6,80493E-81	7	
AW551984	1,1794E-84	0,50524	0,88	0,741	1,98034E-80	7	
Hspb8	1,25991E-84	0,42048	0,985	0,957	2,11552E-80	7	
Etl4	3,24019E-84	0,29209	0,337	0,123	5,44061E-80	7	
Slc35b4	4,79082E-84	0,47675	0,741	0,495	8,04427E-80	7	
Ptbp3	6,29287E-84	0,38741	0,962	0,844	1,05664E-79	7	
Capn5	5,07094E-83	0,47106	0,717	0,478	8,51461E-79	7	
Fitm2	6,14389E-83	0,49818	0,67	0,427	1,03162E-78	7	
Vcan	6,33212E-83	0,42673	0,41	0,177	1,06323E-78	7	
Mvp	1,46521E-82	0,41638	0,544	0,287	2,46024E-78	7	
Nrep	2,9468E-82	0,43135	0,622	0,358	4,94797E-78	7	
Sdcbp	3,31502E-82	0,46416	0,939	0,865	5,56624E-78	7	
Tspan13	3,35248E-82	0,48355	0,899	0,741	5,62915E-78	7	
Spint1	1,44451E-81	0,30369	0,311	0,11	2,42547E-77	7	
Slc22a23	1,65191E-81	0,32375	0,38	0,155	2,77372E-77	7	
Zfhx4	1,94298E-81	0,3999	0,658	0,373	3,26246E-77	7	
Irs3	5,54024E-81	0,40896	0,46	0,217	9,30262E-77	7	
Sort1	7,84956E-81	0,3306	0,387	0,161	1,31802E-76	7	
Gm26888	8,01001E-81	0,27343	0,253	0,078	1,34496E-76	7	
Gng4	1,58599E-80	0,51149	0,833	0,661	2,66303E-76	7	
Ralgds	2,29427E-80	0,48629	0,803	0,601	3,8523E-76	7	
Psap	2,53946E-80	0,3374	1	0,999	4,26401E-76	7	
Tes	4,33064E-80	0,36978	0,537	0,272	7,27157E-76	7	
Hpcal4	1,10801E-79	0,45592	0,784	0,558	1,86046E-75	7	
Nsg1	2,12684E-79	0,38037	0,998	0,986	3,57117E-75	7	
Kcnq5	2,15107E-79	0,30187	0,386	0,159	3,61186E-75	7	
Maged1	2,47489E-79	0,37374	0,987	0,96	4,15559E-75	7	
Saraf	2,89982E-79	0,47922	0,963	0,921	4,86909E-75	7	
Slc7a8	3,36506E-79	0,46861	0,686	0,438	5,65027E-75	7	
Trim62	5,90229E-79	0,34206	0,43	0,196	9,91054E-75	7	
Ptp4a1	2,10978E-78	0,49045	0,788	0,594	3,54254E-74	7	
Lrrc4c	8,44808E-78	0,29719	0,351	0,139	1,41852E-73	7	
Cdh11	2,2863E-77	0,30329	0,395	0,166	3,83892E-73	7	
Arhgef4	7,0796E-77	0,3662	0,421	0,196	1,18874E-72	7	
Ddb1	8,18673E-77	0,5423	0,818	0,716	1,37463E-72	7	
Kif5c	1,89458E-76	0,37396	0,982	0,957	3,18119E-72	7	
Phlda3	2,20329E-76	0,43253	0,85	0,635	3,69955E-72	7	

Reep2	4,86712E-76	0,46143	0,81	0,641	8,17238E-72	7	
Kdelr2	6,60743E-76	0,46758	0,715	0,487	1,10945E-71	7	
R3hdm1	6,75133E-76	0,35537	0,948	0,845	1,13362E-71	7	
Adgrb1	6,10898E-75	0,48758	0,728	0,494	1,02576E-70	7	
Sec24d	7,59171E-75	0,32846	0,407	0,183	1,27472E-70	7	
Pcsk1	8,25651E-75	0,51347	0,829	0,621	1,38635E-70	7	
Efna5	9,59003E-75	0,40107	0,83	0,618	1,61026E-70	7	
Frmpd4	9,63732E-75	0,30075	0,388	0,167	1,6182E-70	7	
Ephb6	1,68619E-74	0,42371	0,632	0,388	2,83128E-70	7	
Camk2a	2,79829E-73	0,35615	0,886	0,7	4,69861E-69	7	
Tmem200a	3,09435E-73	0,30396	0,299	0,112	5,19572E-69	7	
Adgra1	3,23582E-73	0,29982	0,37	0,156	5,43327E-69	7	
Map7	4,62605E-73	0,3849	0,628	0,371	7,7676E-69	7	
Ints3	6,73255E-73	0,43298	0,695	0,468	1,13046E-68	7	
L1cam	2,61538E-72	0,34643	0,993	0,98	4,39149E-68	7	
Spsb1	5,05356E-72	0,26494	0,289	0,105	8,48543E-68	7	
Kcnc2	1,3473E-71	0,28964	0,362	0,153	2,26224E-67	7	
Nr4a3	1,55561E-70	0,43568	0,665	0,421	2,61202E-66	7	
Pcolce	1,66385E-70	0,36145	0,449	0,22	2,79378E-66	7	
Ndst4	1,78687E-70	0,31619	0,598	0,313	3,00034E-66	7	
Stom	6,36715E-70	0,42222	0,901	0,799	1,06911E-65	7	
Tyro3	2,83821E-69	0,41238	0,643	0,415	4,76564E-65	7	
Ppp2r1a	3,23071E-69	0,32499	0,997	0,948	5,42469E-65	7	
Eef1e1	3,62582E-69	0,42763	0,766	0,517	6,08812E-65	7	
Slc8a2	1,6627E-68	0,36544	0,507	0,276	2,79184E-64	7	
Coro1c	2,7915E-68	0,45666	0,746	0,562	4,68721E-64	7	
Eml1	4,02647E-68	0,39027	0,537	0,311	6,76085E-64	7	
Cntn3	5,65699E-68	0,25698	0,289	0,108	9,49865E-64	7	
Arf3	9,2573E-68	0,43455	0,886	0,766	1,55439E-63	7	
Strip1	1,78839E-67	0,33737	0,693	0,434	3,00288E-63	7	
Kcnb2	3,37001E-67	0,38763	0,738	0,505	5,65858E-63	7	
Ets1	4,54149E-67	0,29826	0,427	0,204	7,62561E-63	7	
Nfe2l1	5,48238E-67	0,31607	0,989	0,976	9,20547E-63	7	
Cgref1	7,52604E-67	0,30271	0,378	0,172	1,2637E-62	7	
Kcnq4	8,616E-67	0,34947	0,565	0,325	1,44671E-62	7	
Pkdcc	1,21634E-66	0,25861	0,299	0,116	2,04235E-62	7	
Sept6	1,31078E-66	0,41663	0,751	0,546	2,20092E-62	7	
Lrrc8c	2,40982E-66	0,39165	0,842	0,658	4,04633E-62	7	
Dync1h1	3,35625E-66	0,3276	0,992	0,972	5,63548E-62	7	
Mapk8ip2	1,42319E-65	0,38165	0,918	0,831	2,38969E-61	7	
Nav3	2,64976E-65	0,34756	0,602	0,357	4,44921E-61	7	
Adra2a	4,09559E-65	0,31595	0,473	0,241	6,8769E-61	7	
Dlk1	7,11236E-65	0,35058	0,305	0,123	1,19424E-60	7	
Cpne5	1,06505E-64	0,32117	0,437	0,216	1,78833E-60	7	
Rftn1	1,45791E-64	0,28028	0,361	0,161	2,44798E-60	7	
Nomo1	1,50836E-64	0,39305	0,625	0,401	2,53269E-60	7	
Plxna4	2,24443E-64	0,34226	0,998	0,961	3,76863E-60	7	
Itm2c	3,77053E-64	0,37702	0,943	0,877	6,3311E-60	7	

Gm28905	4,49807E-64	0,28828	0,322	0,134	7,5527E-60	7	
Mab21l1	1,0066E-63	0,40279	0,615	0,39	1,69018E-59	7	
Tsc22d1	1,00999E-63	0,35004	0,959	0,89	1,69588E-59	7	
Prkar1a	1,30814E-63	0,27917	0,998	0,993	2,19649E-59	7	
Sun2	2,84877E-63	0,35165	0,536	0,309	4,78337E-59	7	
Hs3st5	3,24941E-63	0,26406	0,398	0,185	5,45608E-59	7	
Hunk	3,70648E-63	0,3703	0,631	0,405	6,22354E-59	7	
Slc2a13	4,61791E-63	0,36328	0,606	0,367	7,75394E-59	7	
Cplx2	1,0362E-62	0,44616	0,776	0,595	1,73989E-58	7	
Dact3	3,27095E-62	0,32777	0,572	0,335	5,49226E-58	7	
Nynrin	6,74574E-62	0,38115	0,553	0,335	1,13268E-57	7	
Slc20a2	1,24016E-61	0,27386	0,339	0,15	2,08236E-57	7	
Htr2c	1,66567E-61	0,25892	0,34	0,147	2,79683E-57	7	
Syne1	1,82805E-61	0,37019	0,824	0,642	3,06947E-57	7	
Vars	6,22402E-61	0,33865	0,563	0,331	1,04508E-56	7	
Vcp	1,45967E-60	0,29107	0,998	0,993	2,45093E-56	7	
Iqsec3	1,78249E-60	0,40739	0,682	0,47	2,99298E-56	7	
Plcb1	2,01131E-60	0,25235	0,299	0,123	3,3772E-56	7	
Vopp1	6,1624E-60	0,33027	0,531	0,307	1,03473E-55	7	
Fosl2	1,23789E-59	0,41209	0,589	0,373	2,07854E-55	7	
Akt1	2,61358E-59	0,37504	0,859	0,68	4,38846E-55	7	
Gpx3	6,2771E-59	0,57393	0,499	0,297	1,05399E-54	7	
Ptpre	1,00663E-58	0,25308	0,453	0,227	1,69023E-54	7	
Csdc2	2,4471E-58	0,34153	0,481	0,27	4,10893E-54	7	
Lrrn1	2,72102E-58	0,27296	0,382	0,182	4,56887E-54	7	
Slc44a1	4,00893E-58	0,39785	0,808	0,635	6,7314E-54	7	
Cygb	4,47232E-58	0,36618	0,599	0,379	7,50947E-54	7	
Elmo2	5,55634E-58	0,36238	0,623	0,411	9,32965E-54	7	
Gdi1	5,87808E-58	0,3125	0,992	0,973	9,86988E-54	7	
Pde10a	7,10009E-58	0,39988	0,896	0,787	1,19218E-53	7	
Ipo5	7,57595E-58	0,36492	0,655	0,446	1,27208E-53	7	
Phactr1	4,31914E-57	0,34807	0,873	0,691	7,25227E-53	7	
Npr2	4,52364E-57	0,35848	0,659	0,443	7,59564E-53	7	
Fam163a	5,83905E-57	0,362	0,79	0,581	9,80435E-53	7	
Akt2	6,0384E-57	0,3286	0,541	0,325	1,01391E-52	7	
Myo1b	8,74781E-57	0,29157	0,47	0,252	1,46884E-52	7	
Ucp2	9,47244E-57	0,42707	0,643	0,442	1,59052E-52	7	
Adora2a	1,33765E-55	0,27207	0,385	0,191	2,24605E-51	7	
Slc29a4	1,85466E-55	0,32097	0,572	0,344	3,11415E-51	7	
Tagln2	1,10032E-54	0,33274	0,983	0,968	1,84754E-50	7	
Prrt1	2,78108E-54	0,32079	0,476	0,27	4,66971E-50	7	
Abca2	2,96302E-54	0,3635	0,767	0,577	4,97521E-50	7	
Epb41l1	4,08677E-54	0,32306	0,896	0,748	6,86209E-50	7	
Eif4a1	6,18997E-54	0,29576	0,989	0,975	1,03936E-49	7	
Dync1li1	6,8931E-54	0,34409	0,877	0,775	1,15742E-49	7	
Slc25a1	1,78283E-53	0,3563	0,907	0,833	2,99355E-49	7	
Ret	1,85112E-53	0,30732	0,987	0,964	3,10821E-49	7	
Mfap2	2,45893E-53	0,33733	0,49	0,284	4,12879E-49	7	

Plekhm3	6,43417E-53	0,31296	0,734	0,511	1,08036E-48	7	
Ubqln4	8,23603E-53	0,29535	0,508	0,297	1,38291E-48	7	
Syngr1	3,13715E-52	0,3352	0,875	0,75	5,26759E-48	7	
Cmip	5,05805E-52	0,31504	0,945	0,842	8,49298E-48	7	
Mapk3	7,87594E-52	0,28864	0,995	0,99	1,32245E-47	7	
Kif1a	1,34978E-51	0,25347	0,993	0,977	2,26641E-47	7	
Cds2	1,49191E-51	0,28023	0,982	0,907	2,50506E-47	7	
Slc36a1	1,64125E-51	0,27559	0,998	0,958	2,75583E-47	7	
Epb41l5	2,01514E-51	0,27013	0,423	0,224	3,38363E-47	7	
Carhsp1	2,57225E-51	0,3478	0,61	0,411	4,31907E-47	7	
Fhod3	2,72448E-51	0,3595	0,947	0,901	4,57468E-47	7	
Wls	3,13573E-51	0,32109	0,583	0,362	5,2652E-47	7	
Rangap1	3,80119E-51	0,3917	0,766	0,603	6,38258E-47	7	
Creb3l2	4,20486E-51	0,28558	0,477	0,27	7,06038E-47	7	
Unc45a	1,07991E-50	0,33759	0,616	0,417	1,81327E-46	7	
Wscd2	1,48888E-50	0,2749	0,437	0,239	2,49998E-46	7	
Npy1r	1,56652E-50	0,2634	0,335	0,159	2,63034E-46	7	
Tmem63b	2,0072E-50	0,31484	0,884	0,736	3,37029E-46	7	
Rhog	3,48782E-50	0,26703	0,367	0,187	5,85639E-46	7	
Prune2	3,65733E-50	0,34247	0,921	0,819	6,14103E-46	7	
Mapkapk2	8,96179E-50	0,27341	0,429	0,236	1,50477E-45	7	
Pfkl	9,98565E-50	0,29668	0,54	0,333	1,67669E-45	7	
Got2	1,16484E-49	0,33938	0,86	0,745	1,95588E-45	7	
Ppme1	1,63461E-49	0,32905	0,845	0,699	2,74468E-45	7	
Rap1gap2	2,473E-49	0,33078	0,849	0,697	4,15242E-45	7	
Ids	2,72899E-49	0,36552	0,938	0,876	4,58224E-45	7	
Kcnq3	5,35077E-49	0,29195	0,763	0,562	8,98448E-45	7	
Arhgap1	7,22008E-49	0,30821	0,55	0,354	1,21232E-44	7	
Dazap2	8,49711E-49	0,36938	0,571	0,388	1,42675E-44	7	
Shisa5	1,40432E-48	0,38303	0,846	0,747	2,358E-44	7	
Chrna3	1,45685E-48	0,34779	0,965	0,943	2,44619E-44	7	
A730017C20	1,69542E-48	0,35062	0,869	0,693	2,84678E-44	7	
Shc1	3,42087E-48	0,30792	0,509	0,308	5,74398E-44	7	
Elovl5	7,63313E-48	0,33797	0,614	0,417	1,28168E-43	7	
Sigmar1	8,62474E-48	0,35503	0,659	0,475	1,44818E-43	7	
Jup	8,97649E-48	0,36718	0,79	0,631	1,50724E-43	7	
Nell2	1,73724E-47	0,26759	0,37	0,191	2,91699E-43	7	
Sec61a1	3,86568E-47	0,34429	0,716	0,546	6,49087E-43	7	
Myadm	5,64365E-47	0,31001	0,919	0,824	9,47626E-43	7	
Fstl1	6,65528E-47	0,34653	0,765	0,598	1,11749E-42	7	
Slc2a3	6,81558E-47	0,39463	0,662	0,483	1,1444E-42	7	
Drg2	1,5103E-46	0,301	0,543	0,346	2,53595E-42	7	
Mlf2	3,86033E-46	0,31405	0,962	0,943	6,48188E-42	7	
Chpf2	5,02402E-46	0,27634	0,448	0,258	8,43583E-42	7	
Lrp11	8,55951E-46	0,33801	0,866	0,79	1,43723E-41	7	
Magee1	1,10679E-45	0,32497	0,807	0,643	1,85842E-41	7	
Ensa	1,50549E-45	0,31475	0,883	0,79	2,52787E-41	7	
Nek1	2,71084E-45	0,29443	0,715	0,518	4,55177E-41	7	

Syvn1	5,45229E-45	0,3019	0,523	0,329	9,15495E-41	7	
Sh3gl1	1,36791E-44	0,28332	0,499	0,309	2,29686E-40	7	
Ctdnep1	1,66106E-44	0,29886	0,625	0,433	2,78909E-40	7	
Klhdc10	1,88079E-44	0,32826	0,751	0,575	3,15804E-40	7	
Tecpr1	2,3872E-44	0,30894	0,64	0,45	4,00834E-40	7	
Gm2a	2,69733E-44	0,26604	0,406	0,229	4,52909E-40	7	
Gm4076	3,85509E-44	0,3759	0,496	0,315	6,47307E-40	7	
Dpp6	4,98253E-44	0,31379	0,866	0,749	8,36616E-40	7	
Rgs7	8,1351E-44	0,26414	0,376	0,205	1,36597E-39	7	
Atp8a1	1,09919E-43	0,26084	0,895	0,764	1,84565E-39	7	
Sncg	1,34943E-43	0,44373	0,958	0,944	2,26583E-39	7	
Foxo3	1,44655E-43	0,29204	0,583	0,378	2,4289E-39	7	
Aldh3a2	2,64394E-43	0,29128	0,465	0,286	4,43944E-39	7	
Clec14a	2,91862E-43	0,2757	0,453	0,26	4,90065E-39	7	
Kctd10	3,70589E-43	0,29174	0,56	0,37	6,22256E-39	7	
S100a4	9,9108E-43	0,30814	0,893	0,742	1,66412E-38	7	
Enpp5	1,13901E-42	0,34271	0,782	0,648	1,91251E-38	7	
Eps15	1,63656E-42	0,25938	0,526	0,333	2,74794E-38	7	
Csnk1g2	1,85981E-42	0,31348	0,819	0,669	3,12281E-38	7	
Ccne1	1,86337E-42	0,25556	0,372	0,202	3,12879E-38	7	
Dock11	2,58275E-42	0,31253	0,682	0,508	4,3367E-38	7	
Magt1	3,56356E-42	0,28766	0,513	0,324	5,98357E-38	7	
Fhl1	3,59402E-42	0,30385	0,683	0,501	6,03473E-38	7	
Nudt3	5,2478E-42	0,28746	0,844	0,715	8,81158E-38	7	
Fgf1	7,61444E-42	0,38544	0,752	0,641	1,27854E-37	7	
Calr	1,88679E-41	0,3534	0,962	0,938	3,1681E-37	7	
Alad	1,99257E-41	0,28987	0,449	0,271	3,34572E-37	7	
Cdkn1a	2,00001E-41	0,33304	0,606	0,418	3,35822E-37	7	
Cnn3	2,62306E-41	0,28339	0,462	0,281	4,40437E-37	7	
Sept8	3,47437E-41	0,30173	0,645	0,462	5,83381E-37	7	
Esyt1	3,82283E-41	0,2975	0,608	0,426	6,41892E-37	7	
Cidea	5,04124E-41	0,31886	0,891	0,774	8,46474E-37	7	
Slc35a4	7,45649E-41	0,25206	0,48	0,293	1,25202E-36	7	
Garnl3	8,52366E-41	0,2728	0,855	0,716	1,43121E-36	7	
Rit2	1,00258E-40	0,31513	0,741	0,565	1,68343E-36	7	
Tpm1	1,10732E-40	0,27758	0,97	0,934	1,8593E-36	7	
Unc13a	1,12562E-40	0,29256	0,701	0,514	1,89002E-36	7	
Unc5b	1,45074E-40	0,28846	0,675	0,484	2,43594E-36	7	
Icmt	1,55611E-40	0,29083	0,608	0,421	2,61286E-36	7	
Sec24c	1,62679E-40	0,26244	0,476	0,293	2,73154E-36	7	
Slc25a22	2,07078E-40	0,29892	0,708	0,528	3,47704E-36	7	
Ccdc184	3,52416E-40	0,27848	0,547	0,361	5,91741E-36	7	
Capn2	4,08643E-40	0,29412	0,526	0,35	6,86152E-36	7	
Slc22a17	1,19084E-39	0,27257	0,965	0,925	1,99954E-35	7	
Gns	1,39815E-39	0,29445	0,528	0,35	2,34763E-35	7	
Itga3	1,7884E-39	0,27682	0,512	0,335	3,00291E-35	7	
Bscl2	2,04414E-39	0,30913	0,792	0,649	3,43231E-35	7	
Tcof1	2,83421E-39	0,3119	0,55	0,378	4,75892E-35	7	

Rltpr	4,25563E-39	0,29533	0,554	0,378	7,14564E-35	7	
Arhgef10l	8,48348E-39	0,26499	0,499	0,315	1,42446E-34	7	
Rnf181	9,2385E-39	0,27497	0,495	0,318	1,55124E-34	7	
Nr1h2	1,11222E-38	0,29588	0,569	0,393	1,86754E-34	7	
Hspbp1	1,25601E-38	0,30326	0,641	0,469	2,10897E-34	7	
Cntn1	1,2836E-38	0,28942	0,831	0,708	2,15529E-34	7	
Hspb1	1,31885E-38	0,25149	0,462	0,276	2,21448E-34	7	
Psmd3	2,7849E-38	0,28894	0,814	0,671	4,67612E-34	7	
Ssr1	3,11594E-38	0,31636	0,779	0,645	5,23198E-34	7	
Parp10	3,44753E-38	0,25561	0,431	0,262	5,78875E-34	7	
B630019K06	4,16941E-38	0,25317	0,391	0,227	7,00086E-34	7	
Slc7a1	4,28368E-38	0,27304	0,572	0,391	7,19272E-34	7	
Fastk	4,80135E-38	0,26834	0,554	0,375	8,06195E-34	7	
Scap	4,92879E-38	0,27479	0,556	0,385	8,27594E-34	7	
Fbll1	8,99135E-38	0,27712	0,483	0,31	1,50974E-33	7	
Ablim2	1,49206E-37	0,25167	0,544	0,358	2,50532E-33	7	
Vapb	1,78741E-37	0,327	0,81	0,721	3,00124E-33	7	
Grn	2,55263E-37	0,29729	0,827	0,712	4,28612E-33	7	
Rab11a	5,23071E-37	0,28036	0,879	0,785	8,78289E-33	7	
Trim8	6,92828E-37	0,25268	0,513	0,333	1,16333E-32	7	
Sgpl1	7,30785E-37	0,25022	0,525	0,346	1,22706E-32	7	
Chrnb4	8,18407E-37	0,32445	0,776	0,648	1,37419E-32	7	
Pofut2	9,0659E-37	0,26789	0,537	0,365	1,52225E-32	7	
Lrrc8b	1,11909E-36	0,27708	0,574	0,398	1,87906E-32	7	
AI837181	1,19746E-36	0,26322	0,632	0,453	2,01066E-32	7	
Nr4a1	1,19836E-36	0,29304	0,43	0,265	2,01217E-32	7	
Gtf3c1	3,9811E-36	0,29234	0,661	0,495	6,68466E-32	7	
Wasf2	4,097E-36	0,28616	0,618	0,45	6,87927E-32	7	
Scamp1	4,29615E-36	0,29516	0,828	0,718	7,21366E-32	7	
Per1	4,32553E-36	0,25459	0,425	0,257	7,26301E-32	7	
Bag6	4,4079E-36	0,30046	0,703	0,545	7,40131E-32	7	
Crabp1	6,17125E-36	0,27243	0,667	0,467	1,03622E-31	7	
Prpf19	7,28395E-36	0,29507	0,652	0,495	1,22305E-31	7	
Rab3gap2	8,17112E-36	0,26241	0,665	0,486	1,37201E-31	7	
Znfx1	1,18545E-35	0,25536	0,556	0,378	1,99048E-31	7	
Flii	1,21259E-35	0,26683	0,612	0,442	2,03605E-31	7	
Slc4a8	1,56331E-35	0,29735	0,683	0,524	2,62495E-31	7	
Qsox1	4,02494E-35	0,26037	0,587	0,41	6,75827E-31	7	
Clip2	7,73943E-35	0,29017	0,664	0,509	1,29953E-30	7	
Kcnq2	9,16543E-35	0,2834	0,772	0,617	1,53897E-30	7	
Eif3b	1,03282E-34	0,32068	0,627	0,483	1,7342E-30	7	
Arhgef40	1,42367E-34	0,26296	0,474	0,306	2,39048E-30	7	
U2af2	1,82438E-34	0,27831	0,761	0,616	3,06332E-30	7	
Serpini1	2,60091E-34	0,28439	0,851	0,734	4,36718E-30	7	
Slc3a2	3,01521E-34	0,32874	0,765	0,629	5,06284E-30	7	
Poldip2	3,94147E-34	0,26018	0,58	0,406	6,61812E-30	7	
Rab3d	4,82632E-34	0,31252	0,685	0,544	8,10388E-30	7	
Hsph1	4,86596E-34	0,2661	0,556	0,39	8,17044E-30	7	

Prkaca	7,70423E-34	0,25844	0,896	0,818	1,29362E-29	7	
Atf4	8,48094E-34	0,25613	0,944	0,887	1,42403E-29	7	
Add1	1,16836E-33	0,25207	0,914	0,844	1,9618E-29	7	
Gm38112	1,37446E-33	0,26983	0,52	0,349	2,30786E-29	7	
5330417C22	1,61755E-33	0,2686	0,525	0,361	2,71604E-29	7	
Madd	2,45109E-33	0,27774	0,774	0,627	4,11563E-29	7	
Tgfb1i1	7,06008E-33	0,25433	0,652	0,476	1,18546E-28	7	
Chga	8,54134E-33	0,27264	0,802	0,658	1,43418E-28	7	
Coro2b	1,08086E-32	0,2524	0,78	0,624	1,81487E-28	7	
Prrc1	1,27471E-32	0,25219	0,642	0,472	2,14036E-28	7	
Nbas	1,42329E-32	0,28158	0,65	0,503	2,38984E-28	7	
Fam171a1	1,63976E-32	0,25402	0,552	0,39	2,75333E-28	7	
Gm15800	3,33955E-32	0,26638	0,833	0,684	5,60744E-28	7	
Elk3	5,7191E-32	0,28617	0,655	0,491	9,60294E-28	7	
Mill2	9,21425E-32	0,27479	0,488	0,332	1,54716E-27	7	
Fam69b	1,08335E-31	0,28504	0,674	0,537	1,81905E-27	7	
Hsp90b1	1,43853E-31	0,25535	0,988	0,987	2,41543E-27	7	
Arpc4	3,56074E-31	0,26046	0,609	0,456	5,97884E-27	7	
Erbb2ip	3,84552E-31	0,25088	0,472	0,314	6,45701E-27	7	
Mxd4	5,60967E-31	0,27339	0,748	0,604	9,41919E-27	7	
Syn1	7,00859E-31	0,27451	0,823	0,734	1,17681E-26	7	
Clptm1l	1,2492E-30	0,28335	0,695	0,552	2,09754E-26	7	
Txnrd1	2,17559E-30	0,27266	0,688	0,551	3,65303E-26	7	
Sdhc	2,17674E-30	0,2712	0,712	0,573	3,65497E-26	7	
Cct8	4,04261E-30	0,27126	0,903	0,857	6,78794E-26	7	
Ddx54	6,46769E-30	0,26602	0,626	0,475	1,08599E-25	7	
Kars	1,01666E-29	0,25382	0,519	0,367	1,70707E-25	7	
Slc38a10	1,89057E-29	0,26464	0,655	0,511	3,17445E-25	7	
Mab21l2	8,36432E-29	0,29223	0,755	0,623	1,40445E-24	7	
Disp2	2,59978E-28	0,254	0,813	0,721	4,36528E-24	7	
Nrbp1	4,62933E-28	0,267	0,698	0,572	7,77312E-24	7	
Mvk	9,02547E-28	0,25506	0,599	0,444	1,51547E-23	7	
Map2k1	9,36508E-27	0,25313	0,676	0,548	1,57249E-22	7	
Ldlr	2,33762E-26	0,29007	0,745	0,636	3,92509E-22	7	
Htt	5,18566E-26	0,26202	0,607	0,451	8,70724E-22	7	
Acly	5,9654E-26	0,28177	0,875	0,82	1,00165E-21	7	
Usp5	9,48132E-26	0,25715	0,705	0,589	1,59201E-21	7	
Egr1	3,86025E-21	0,31202	0,494	0,372	6,48174E-17	7	
Paip2b	5,39237E-12	0,38285	0,417	0,52	9,05433E-08	7	
Vip	0	1,8037	1	0,913	0	8	smENC3c
S100a4	0	1,49459	0,97	0,708	0	8	
Rps21	0	1,44674	1	0,94	0	8	
Cd9	0	1,42858	1	0,992	0	8	
Tuba1a	0	1,39319	1	0,991	0	8	
Gapdh	0	1,3899	1	0,916	0	8	
Igfbp7	0	1,36947	0,938	0,258	0	8	
Rps27	0	1,36753	0,999	0,833	0	8	
Npy	0	1,36332	0,997	0,829	0	8	

Scgn	0	1,35037	0,999	0,845	0	8	
Rpl39	0	1,34804	1	0,921	0	8	
Gm10076	0	1,34349	1	0,808	0	8	
Cox7c	0	1,34231	1	0,948	0	8	
Crip1	0	1,34072	1	0,984	0	8	
Rpl9	0	1,25846	0,997	0,772	0	8	
Lgals1	0	1,25766	0,997	0,9	0	8	
Rps4x	0	1,25565	0,999	0,857	0	8	
Resp18	0	1,25032	0,993	0,919	0	8	
Fxyd5	0	1,23521	0,886	0,329	0	8	
Atpif1	0	1,22379	1	0,939	0	8	
Rpl19	0	1,21748	1	0,916	0	8	
Rps3a1	0	1,18739	0,992	0,791	0	8	
Rpl35	0	1,18456	0,999	0,787	0	8	
Atp5j2	0	1,17737	0,998	0,742	0	8	
Sec61g	0	1,17439	0,999	0,839	0	8	
Sh3bgrl3	0	1,16427	0,961	0,544	0	8	
Ubb	0	1,16023	1	0,984	0	8	
Mpc1	0	1,15966	0,983	0,795	0	8	
Id3	0	1,15766	0,905	0,348	0	8	
Cox4i1	0	1,14749	1	0,86	0	8	
Rps28	0	1,14692	0,999	0,781	0	8	
Rps7	0	1,13809	0,995	0,734	0	8	
Rps24	0	1,13254	1	0,96	0	8	
Dstn	0	1,12261	1	0,938	0	8	
Cox6c	0	1,11219	0,999	0,84	0	8	
Rps29	0	1,10986	1	0,996	0	8	
Ppa1	0	1,10822	0,931	0,499	0	8	
Hint1	0	1,10798	1	0,854	0	8	
Rps27a	0	1,09791	1	0,912	0	8	
Rps8	0	1,09293	1	0,973	0	8	
Atp5e	0	1,09222	0,998	0,805	0	8	
Stmn3	0	1,09074	1	0,914	0	8	
Rpl35a	0	1,08991	1	0,905	0	8	
Ndufa1	0	1,08519	0,962	0,486	0	8	
Rps23	0	1,08039	0,999	0,88	0	8	
Rpl37a	0	1,07564	1	0,984	0	8	
Rps13	0	1,07007	0,995	0,776	0	8	
Fau	0	1,06628	1	0,905	0	8	
Uqcr11	0	1,0657	0,999	0,786	0	8	
Rps3	0	1,06543	0,998	0,834	0	8	
Bglap	0	1,05926	0,633	0,125	0	8	
Nsg1	0	1,05902	1	0,985	0	8	
Rpl36a	0	1,0496	0,984	0,707	0	8	
Chchd2	0	1,04425	0,999	0,9	0	8	
Tpt1	0	1,04185	1	0,946	0	8	
Prdx2	0	1,03978	0,994	0,786	0	8	
Rps9	0	1,03661	1	0,898	0	8	

Tmem256	0	1,0331	0,923	0,407	0	8	
Rps20	0	1,03024	1	0,875	0	8	
Atp5k	0	1,02619	1	0,949	0	8	
Ndufa4	0	1,02124	0,993	0,802	0	8	
Rpl3	0	1,01421	1	0,902	0	8	
Rps10	0	1,01419	1	0,901	0	8	
Rps12	0	1,01319	0,995	0,786	0	8	
Rpl34	0	1,00889	1	0,898	0	8	
Rpl38	0	1,00397	1	0,993	0	8	
Cox7a2	0	1,00101	0,997	0,815	0	8	
Rpl30	0	0,99991	0,996	0,768	0	8	
Rpl6	0	0,993	1	0,966	0	8	
Rpl37	0	0,99147	1	0,983	0	8	
Pfdn5	0	0,98512	0,987	0,694	0	8	
Rps5	0	0,98218	0,998	0,859	0	8	
S100a6	0	0,98034	1	0,999	0	8	
Rpl7	0	0,97836	0,982	0,711	0	8	
Uqcrh	0	0,97517	0,982	0,716	0	8	
Cox6b1	0	0,97481	0,998	0,841	0	8	
Rps2	0	0,97406	0,993	0,808	0	8	
Rpl22l1	0	0,97318	0,988	0,726	0	8	
Tubb4b	0	0,97066	0,983	0,736	0	8	
Ftl1	0	0,96994	0,999	0,912	0	8	
Rpl17	0	0,96807	0,999	0,898	0	8	
Lst1	0	0,96634	0,883	0,381	0	8	
Rpl24	0	0,96417	1	0,933	0	8	
Etv1	0	0,95976	0,999	0,531	0	8	
Cox7a2l	0	0,95968	0,936	0,544	0	8	
Romo1	0	0,95882	0,993	0,838	0	8	
Rpl18	0	0,95667	0,999	0,835	0	8	
Cidea	0	0,95595	0,984	0,738	0	8	
Rps16	0	0,94848	1	0,904	0	8	
Tmsb10	0	0,94801	1	0,942	0	8	
Tomm7	0	0,947	0,989	0,717	0	8	
Ndufb9	0	0,94557	0,953	0,558	0	8	
Ndufa7	0	0,94274	0,977	0,629	0	8	
Rpl10	0	0,93528	0,998	0,849	0	8	
Tgfb1	0	0,93364	0,787	0,235	0	8	
Cst3	0	0,93239	0,996	0,922	0	8	
Ckb	0	0,92921	0,95	0,68	0	8	
Thy1	0	0,92633	0,955	0,446	0	8	
Rpl13	0	0,92448	1	0,946	0	8	
Ndufb10	0	0,92362	0,914	0,412	0	8	
Fth1	0	0,91755	1	0,993	0	8	
Atp5h	0	0,91703	0,997	0,842	0	8	
Atp5f1	0	0,91595	0,944	0,601	0	8	
Uba52	0	0,91533	0,825	0,373	0	8	
Rplp2	0	0,90981	0,999	0,916	0	8	

2010107E04	0	0,90876	0,949	0,582	0	8	
Cox6a1	0	0,90635	0,991	0,813	0	8	
Cox5a	0	0,90399	0,944	0,553	0	8	
Ndufa3	0	0,89944	0,991	0,718	0	8	
Rpl14	0	0,89827	1	0,98	0	8	
Tm4sf4	0	0,89503	0,714	0,257	0	8	
Psme2	0	0,89218	0,917	0,468	0	8	
Rps15a	0	0,89046	0,997	0,859	0	8	
Gnb2l1	0	0,89042	0,989	0,758	0	8	
Rps11	0	0,88597	1	0,922	0	8	
Cox8a	0	0,88516	1	0,915	0	8	
Ndufc1	0	0,88455	0,992	0,773	0	8	
Psmb5	0	0,88294	0,984	0,728	0	8	
Rplp0	0	0,88167	0,978	0,726	0	8	
Rpl29	0	0,87694	0,925	0,536	0	8	
Rpl22	0	0,87527	0,94	0,603	0	8	
Gng5	0	0,8737	0,882	0,449	0	8	
Gstm5	0	0,87032	0,866	0,389	0	8	
Atp5c1	0	0,87019	0,925	0,524	0	8	
Slc25a4	0	0,86989	0,999	0,976	0	8	
Rpl10a	0	0,86925	0,995	0,83	0	8	
Ttc9b	0	0,86808	0,903	0,507	0	8	
Phlda3	0	0,86773	0,939	0,593	0	8	
Ubl5	0	0,86615	0,978	0,722	0	8	
Myeov2	0	0,86481	0,879	0,409	0	8	
Rpl41	0	0,86424	1	0,998	0	8	
Ndufb8	0	0,86197	0,928	0,493	0	8	
Btf3	0	0,85639	0,972	0,699	0	8	
Cuedc2	0	0,85462	0,885	0,441	0	8	
Gfra1	0	0,85168	0,938	0,356	0	8	
Fdps	0	0,85154	0,964	0,709	0	8	
Gabarapl2	0	0,84582	0,993	0,842	0	8	
Ssr4	0	0,84467	0,892	0,478	0	8	
Gm1673	0	0,84353	0,977	0,725	0	8	
Rabac1	0	0,84284	0,995	0,878	0	8	
Gm13889	0	0,84208	0,826	0,3	0	8	
Rpl11	0	0,83773	1	0,943	0	8	
Cox5b	0	0,83719	0,991	0,758	0	8	
Rps18	0	0,83562	1	0,921	0	8	
Eif2s2	0	0,83256	0,998	0,892	0	8	
Mrpl20	0	0,83227	0,843	0,359	0	8	
Nme1	0	0,83075	0,937	0,598	0	8	
Arpc3	0	0,8302	0,975	0,732	0	8	
Tmsb4x	0	0,82981	1	1	0	8	
Ndufs5	0	0,82876	0,996	0,821	0	8	
Tspo	0	0,82871	0,944	0,581	0	8	
Tmem258	0	0,82794	0,875	0,393	0	8	
Ndufb2	0	0,82613	0,937	0,597	0	8	

Calm2	0	0,82376	1	1	0	8	
Rpl32	0	0,82261	1	0,959	0	8	
Rps6	0	0,82171	0,995	0,807	0	8	
Ndufb11	0	0,81825	0,92	0,531	0	8	
Atp5l	0	0,81524	0,999	0,885	0	8	
Uqcrq	0	0,81495	0,996	0,828	0	8	
Uqcr10	0	0,80232	0,976	0,682	0	8	
Rpl12	0	0,80148	0,985	0,763	0	8	
Rpl27a	0	0,80071	1	0,956	0	8	
Csrp1	0	0,79904	0,999	0,962	0	8	
Acot7	0	0,79805	0,96	0,691	0	8	
Rpl26	0	0,78982	0,999	0,94	0	8	
Ndufb6	0	0,78584	0,897	0,446	0	8	
Ndufa5	0	0,78414	0,993	0,76	0	8	
Rpl7a	0	0,78216	0,987	0,786	0	8	
Rps26	0	0,77832	0,997	0,9	0	8	
Cox7b	0	0,77746	0,989	0,806	0	8	
Psma7	0	0,77713	0,996	0,864	0	8	
Rps19	0	0,77182	0,999	0,935	0	8	
Rps25	0	0,76573	0,994	0,822	0	8	
Ldha	0	0,75862	0,999	0,969	0	8	
Usmg5	0	0,75855	0,991	0,821	0	8	
Eef1b2	0	0,75562	0,986	0,773	0	8	
Slc25a3	0	0,74845	0,998	0,951	0	8	
Naca	0	0,73772	0,994	0,846	0	8	
Dync1i2	0	0,7323	1	0,999	0	8	
Rps14	0	0,72792	1	0,943	0	8	
Nfe2l2	0	0,71414	0,64	0,193	0	8	
Hspa8	0	0,69089	1	0,994	0	8	
Smpd3	0	0,65302	1	0,998	0	8	
Basp1	0	0,65158	1	0,999	0	8	
Eef1a1	0	0,64778	1	0,998	0	8	
Tubb2a	0	0,6421	1	0,994	0	8	
Uchl1	0	0,63814	1	1	0	8	
Bex2	0	0,60643	1	0,998	0	8	
Stmn2	0	0,52685	1	1	0	8	
Uqcrb	7,6666E-307	0,7565	0,98	0,753	1,2873E-302	8	
Edf1	1,1841E-306	0,76791	0,952	0,643	1,9882E-302	8	
Dynll1	1,2889E-306	0,63234	1	0,986	2,1641E-302	8	
Mpc2	5,6945E-305	0,76939	0,85	0,397	9,5617E-301	8	
Rpl36al	8,1125E-305	0,75447	0,991	0,801	1,3622E-300	8	
Hist3h2ba	2,291E-304	0,80188	0,83	0,396	3,8468E-300	8	
Mrpl23	4,0205E-303	0,71356	0,787	0,329	6,7508E-299	8	
Atp6v0e	7,2569E-303	0,80753	0,86	0,472	1,2185E-298	8	
Oaz1	1,6564E-301	0,72726	0,998	0,912	2,7812E-297	8	
Bola2	1,6713E-301	0,78118	0,927	0,565	2,8062E-297	8	
Acyp2	8,2114E-299	0,77831	0,874	0,476	1,3788E-294	8	
Cetn2	3,7604E-298	0,78051	0,949	0,636	6,314E-294	8	

Rpl23	4,694E-297	0,60749	1	0,99	7,8818E-293	8	
Alcam	5,0174E-297	0,85195	0,966	0,543	8,4247E-293	8	
Moxd1	3,9562E-296	0,81111	0,926	0,403	6,6429E-292	8	
Sec62	9,4113E-296	0,69098	0,999	0,926	1,5802E-291	8	
Ndufa13	1,9318E-295	0,73781	0,987	0,76	3,2437E-291	8	
Eif3h	2,014E-295	0,7622	0,869	0,451	3,3818E-291	8	
Ndufb4	3,5008E-294	0,74691	0,983	0,725	5,8783E-290	8	
Rbm3	2,3672E-292	0,78096	0,966	0,748	3,9748E-288	8	
Ndufa6	1,8299E-291	0,76645	0,925	0,565	3,0726E-287	8	
Rab3a	4,58E-290	0,72027	0,992	0,873	7,6903E-286	8	
Ndst4	8,8756E-290	0,73679	0,717	0,257	1,4903E-285	8	
Dctn3	1,9366E-289	0,75006	0,952	0,668	3,2517E-285	8	
Tbcb	3,6947E-289	0,7439	0,942	0,654	6,2038E-285	8	
Dctn2	5,4417E-289	0,75684	0,954	0,666	9,1371E-285	8	
Cntnap5a	2,1376E-287	0,77834	0,979	0,604	3,5892E-283	8	
Serpinb6a	1,0717E-286	0,72594	0,978	0,814	1,7994E-282	8	
Atp5b	4,9704E-286	0,70837	0,996	0,902	8,3458E-282	8	
Gabarap	3,2523E-285	0,6604	0,996	0,902	5,461E-281	8	
Sec61b	9,981E-284	0,76177	0,902	0,536	1,6759E-279	8	
Ndufb5	7,6066E-283	0,73601	0,839	0,404	1,2772E-278	8	
Ccdc109b	8,5601E-282	0,51497	0,414	0,076	1,4373E-277	8	
Nenf	3,3616E-281	0,72355	0,97	0,726	5,6445E-277	8	
Rps17	1,2011E-279	0,69111	0,999	0,891	2,0168E-275	8	
Gng3	3,7935E-279	0,72496	0,98	0,772	6,3696E-275	8	
Ncoa7	1,3834E-278	0,82086	0,956	0,755	2,3229E-274	8	
Cops6	1,4477E-276	0,74452	0,885	0,51	2,4309E-272	8	
Mtch1	2,9707E-276	0,63275	0,998	0,961	4,9882E-272	8	
Tubb2b	6,3607E-276	0,64443	0,999	0,982	1,068E-271	8	
1110004F10	1,0391E-275	0,68756	0,995	0,916	1,7447E-271	8	
Scg5	2,1151E-275	0,76465	0,967	0,813	3,5514E-271	8	
Atp5g2	1,7679E-274	0,69349	0,985	0,812	2,9684E-270	8	
Pcolce	3,0596E-274	0,60291	0,573	0,166	5,1374E-270	8	
Mif	3,3189E-272	0,71707	0,967	0,697	5,5728E-268	8	
Polr1d	4,3782E-270	0,72142	0,831	0,429	7,3514E-266	8	
Cd24a	5,337E-270	0,73396	0,991	0,55	8,9613E-266	8	
Cox8b	6,2478E-270	0,98378	0,306	0,036	1,0491E-265	8	
Rprml	1,3413E-269	0,59341	0,428	0,09	2,2522E-265	8	
Ngfrap1	3,5381E-269	0,63452	0,994	0,937	5,9408E-265	8	
Psmb1	5,2834E-269	0,68576	0,986	0,79	8,8714E-265	8	
Gnas	4,6218E-268	0,43078	1	1	7,7605E-264	8	
Ndufa12	6,9E-267	0,70898	0,893	0,505	1,1586E-262	8	
Cox17	5,6645E-266	0,71533	0,851	0,438	9,5113E-262	8	
H2afj	1,7538E-265	0,68659	0,75	0,334	2,9448E-261	8	
Cox20	2,8032E-264	0,61015	0,614	0,207	4,7068E-260	8	
Ndufa2	4,3196E-264	0,64832	0,99	0,861	7,253E-260	8	
Spcs1	4,8695E-264	0,73445	0,852	0,445	8,1764E-260	8	
Hpca	7,6427E-264	0,67063	0,74	0,287	1,2833E-259	8	
Atp6v1e1	3,7869E-261	0,62192	1	0,95	6,3586E-257	8	

Tceal6	8,5331E-261	0,6426	0,591	0,193	1,4328E-256	8	
Eef1e1	1,6452E-260	0,76963	0,863	0,47	2,7625E-256	8	
Aimp1	6,9072E-257	0,70996	0,933	0,608	1,1598E-252	8	
Cryab	1,4198E-256	0,78413	0,743	0,325	2,384E-252	8	
Rplp1	5,2406E-256	0,60475	1	0,974	8,7996E-252	8	
Mrpl16	9,9039E-256	0,6381	0,655	0,25	1,663E-251	8	
Ndufs4	2,6177E-254	0,71359	0,858	0,481	4,3953E-250	8	
Glrx5	2,9734E-254	0,6481	0,732	0,319	4,9927E-250	8	
Ptprz1	4,2459E-254	0,63302	0,652	0,224	7,1293E-250	8	
1810022K09	3,1651E-251	0,66314	0,899	0,509	5,3145E-247	8	
Atp5o	1,163E-249	0,67701	0,979	0,763	1,9527E-245	8	
Apoa1bp	2,3198E-249	0,56106	0,591	0,199	3,8952E-245	8	
Gal	5,3796E-249	1,43072	0,52	0,159	9,0329E-245	8	
Lamtor4	1,2448E-248	0,62247	0,757	0,333	2,0901E-244	8	
Rps15	1,4115E-248	0,62529	0,999	0,933	2,3701E-244	8	
Atp6v0d1	2,4612E-247	0,6891	0,954	0,748	4,1325E-243	8	
Tmem160	1,0332E-246	0,66549	0,808	0,392	1,7348E-242	8	
Atp6v1f	1,5148E-246	0,65573	0,967	0,774	2,5435E-242	8	
Pin4	6,1397E-246	0,65707	0,793	0,373	1,0309E-241	8	
Dpm3	7,3022E-246	0,69173	0,896	0,538	1,2261E-241	8	
Mrpl28	7,8215E-245	0,54743	0,634	0,223	1,3133E-240	8	
Gm10073	1,0354E-244	0,62582	0,634	0,231	1,7385E-240	8	
Rpl36	4,1438E-244	0,62526	0,999	0,934	6,9579E-240	8	
Sh3bgrl	3,8514E-243	0,69009	0,83	0,442	6,4669E-239	8	
Prdx1	8,9653E-243	0,58352	0,997	0,947	1,5054E-238	8	
Aldoa	1,1063E-241	0,52668	1	0,995	1,8576E-237	8	
2410015M2(	2,0925E-241	0,68068	0,857	0,476	3,5134E-237	8	
Ndufv3	5,0816E-241	0,63391	0,984	0,831	8,5326E-237	8	
Ppib	6,9137E-241	0,69299	0,873	0,532	1,1609E-236	8	
Cyc1	1,1029E-240	0,63952	0,788	0,374	1,8519E-236	8	
Trappc1	5,5303E-240	0,57223	0,627	0,23	9,286E-236	8	
Myl6	2,0917E-239	0,58163	0,996	0,94	3,5121E-235	8	
Ndfip1	2,666E-239	0,69111	0,822	0,446	4,4766E-235	8	
Nmt1	1,4303E-236	0,60123	0,997	0,901	2,4016E-232	8	
Myl1	3,7961E-236	0,63669	0,995	0,923	6,374E-232	8	
Psmd6	4,2985E-236	0,66188	0,836	0,453	7,2176E-232	8	
Gpx1	2,0394E-235	0,59864	0,655	0,257	3,4244E-231	8	
Gap43	3,1676E-235	0,51543	1	0,997	5,3187E-231	8	
Ndufb7	4,2134E-235	0,64694	0,966	0,731	7,0748E-231	8	
Ndufc2	1,229E-234	0,62642	0,969	0,773	2,0637E-230	8	
Bloc1s5	6,1248E-234	0,56528	0,599	0,217	1,0284E-229	8	
Psme1	7,7253E-233	0,62516	0,986	0,816	1,2972E-228	8	
Lamtor2	7,8085E-233	0,64965	0,833	0,442	1,3111E-228	8	
Tspan13	2,8483E-232	0,67206	0,949	0,714	4,7826E-228	8	
Tceb2	1,1479E-231	0,57465	0,999	0,948	1,9275E-227	8	
Ywhae	2,9443E-231	0,55033	1	0,995	4,9438E-227	8	
Polr2i	1,1115E-230	0,58346	0,698	0,293	1,8663E-226	8	
Cela1	1,3817E-230	0,41352	0,349	0,065	2,3201E-226	8	

1500009L16[illegible]	1,4328E-228	0,68615	0,806	0,432	2,4057E-224	8	
Akr1a1	2,4716E-228	0,62329	0,975	0,889	4,15E-224	8	
Bsg	1,3119E-226	0,58616	0,992	0,926	2,2028E-222	8	
Ubc	2,0426E-226	0,56033	0,999	0,975	3,4297E-222	8	
Fkbp3	3,3314E-226	0,65868	0,904	0,595	5,5937E-222	8	
Atp5j	8,752E-226	0,60556	0,985	0,794	1,4695E-221	8	
Ddt	9,1665E-225	0,46063	0,461	0,125	1,5391E-220	8	
Nop10	6,0432E-224	0,64161	0,865	0,495	1,0147E-219	8	
D8Ertd738e	1,0528E-223	0,64316	0,858	0,485	1,7678E-219	8	
Usp50	1,3664E-222	0,59506	0,704	0,308	2,2943E-218	8	
Pfdn1	2,082E-222	0,64924	0,9	0,595	3,4959E-218	8	
Bmyc	5,5672E-222	0,62466	0,796	0,421	9,3479E-218	8	
Pam16	1,2585E-221	0,59106	0,74	0,34	2,1131E-217	8	
Ndufaf2	1,3791E-221	0,58224	0,709	0,303	2,3156E-217	8	
Tonsl	1,5578E-221	0,64752	0,89	0,54	2,6157E-217	8	
Mien1	1,5918E-220	0,60064	0,744	0,35	2,6728E-216	8	
Trappc4	7,8124E-220	0,64114	0,803	0,431	1,3118E-215	8	
Ube2m	2,3557E-219	0,62894	0,927	0,653	3,9554E-215	8	
Pgls	1,3011E-217	0,64571	0,854	0,487	2,1848E-213	8	
Rgs10	1,6529E-217	0,53818	0,584	0,213	2,7753E-213	8	
Cd63	1,6957E-217	0,62969	0,678	0,3	2,8472E-213	8	
Fam89a	1,9968E-217	0,54247	0,544	0,186	3,3528E-213	8	
Ccdc124	2,9504E-217	0,59596	0,775	0,384	4,954E-213	8	
Rpl5	1,0695E-216	0,6019	0,978	0,832	1,7958E-212	8	
Mdh2	1,142E-216	0,62589	0,947	0,665	1,9176E-212	8	
Tmem50a	4,3347E-216	0,58726	0,981	0,893	7,2783E-212	8	
Naa38	4,6143E-216	0,55903	0,679	0,289	7,7478E-212	8	
Ppp1ca	5,0802E-216	0,60353	0,973	0,818	8,5302E-212	8	
Tmem208	5,4734E-216	0,57623	0,681	0,296	9,1905E-212	8	
Ifitm2	3,0685E-215	0,57553	0,999	0,94	5,1523E-211	8	
Ndufab1	8,1838E-215	0,61511	0,934	0,645	1,3741E-210	8	
Pgam1	8,6439E-215	0,63837	0,947	0,774	1,4514E-210	8	
Rexo2	1,6667E-214	0,67707	0,859	0,551	2,7986E-210	8	
Psmc3	2,8137E-214	0,62304	0,949	0,712	4,7244E-210	8	
Glrx3	5,5672E-213	0,61703	0,812	0,43	9,3478E-209	8	
Hcfc1r1	2,4087E-212	0,58455	0,989	0,878	4,0444E-208	8	
Lamtor5	2,9995E-212	0,57466	0,741	0,348	5,0365E-208	8	
Dad1	2,2962E-211	0,59683	0,967	0,79	3,8556E-207	8	
S100a10	2,7675E-211	0,4978	1	0,982	4,647E-207	8	
Smdt1	4,4213E-211	0,55743	0,985	0,874	7,4238E-207	8	
Park7	4,9818E-211	0,58502	0,966	0,774	8,3649E-207	8	
Rpl21	1,3483E-210	0,539	1	0,96	2,2639E-206	8	
Sub1	1,7049E-209	0,52366	0,993	0,945	2,8627E-205	8	
Lxn	2,0282E-209	0,4716	0,512	0,165	3,4056E-205	8	
Hsd17b10	3,4696E-209	0,53164	0,649	0,267	5,8258E-205	8	
Prdx4	1,0665E-207	0,49308	0,505	0,161	1,7907E-203	8	
Spock3	2,5858E-206	0,59736	0,921	0,541	4,3418E-202	8	
Ost4	3,3284E-206	0,59172	0,784	0,411	5,5887E-202	8	

Pet100	1,4299E-205	0,64073	0,889	0,581	2,401E-201	8	
Cd1d1	1,5712E-204	0,42453	0,379	0,09	2,6382E-200	8	
Vdac2	3,4493E-203	0,64595	0,893	0,635	5,7917E-199	8	
Lix1	5,0208E-203	0,70269	0,927	0,727	8,4305E-199	8	
Cops5	6,298E-203	0,63252	0,92	0,692	1,0575E-198	8	
Hagh	3,9945E-202	0,58997	0,767	0,394	6,7072E-198	8	
Mrpl51	1,4546E-201	0,61664	0,874	0,543	2,4425E-197	8	
Slirp	1,7544E-201	0,5784	0,746	0,36	2,9458E-197	8	
Qpct	5,6429E-199	0,43754	0,407	0,107	9,4749E-195	8	
Chchd1	8,9422E-199	0,5096	0,637	0,264	1,5015E-194	8	
A730017C20	1,1077E-198	0,73258	0,899	0,67	1,8599E-194	8	
Srp14	1,8268E-198	0,58437	0,955	0,784	3,0674E-194	8	
Asl	3,1256E-198	0,60612	0,952	0,727	5,2482E-194	8	
Psma4	3,7224E-198	0,59511	0,934	0,731	6,2503E-194	8	
Mrps24	5,1744E-198	0,53144	0,697	0,318	8,6884E-194	8	
Eif6	7,9602E-198	0,5324	0,657	0,286	1,3366E-193	8	
Selk	8,4345E-198	0,4837	0,998	0,966	1,4162E-193	8	
Atp5d	1,8706E-197	0,57441	0,972	0,832	3,1409E-193	8	
Dgcr6	3,5888E-197	0,54757	0,733	0,352	6,0259E-193	8	
Sdhb	1,4478E-196	0,60899	0,854	0,507	2,4309E-192	8	
Coa3	1,642E-196	0,59478	0,813	0,459	2,757E-192	8	
Nol7	1,7292E-196	0,60071	0,831	0,478	2,9035E-192	8	
Fbn1	5,3026E-196	0,47907	0,636	0,241	8,9036E-192	8	
Prdx6	1,9223E-195	0,46989	0,501	0,168	3,2278E-191	8	
Use1	2,3015E-195	0,56213	0,749	0,367	3,8645E-191	8	
Mrpl17	3,0509E-195	0,53271	0,668	0,296	5,1228E-191	8	
Aprt	1,0564E-194	0,62108	0,825	0,483	1,7737E-190	8	
S100a13	5,701E-192	0,59731	0,987	0,877	9,5725E-188	8	
Hsp90aa1	9,9022E-192	0,47993	1	0,987	1,6627E-187	8	
Psmd12	1,1533E-191	0,59055	0,865	0,533	1,9364E-187	8	
Rwdd1	1,3499E-191	0,58155	0,834	0,481	2,2665E-187	8	
Lamc3	1,5078E-191	0,48329	0,614	0,225	2,5317E-187	8	
Fam162a	1,6271E-191	0,54462	0,708	0,332	2,7321E-187	8	
Chmp2a	2,6783E-191	0,57053	0,969	0,794	4,4972E-187	8	
Fabp5	3,8941E-191	0,50353	1	0,976	6,5386E-187	8	
Psmb4	4,4708E-191	0,57447	0,959	0,744	7,507E-187	8	
Ppia	4,8557E-191	0,42234	1	1	8,1532E-187	8	
Aurkaip1	1,216E-190	0,55625	0,726	0,35	2,0418E-186	8	
Atox1	1,9435E-190	0,60278	0,89	0,602	3,2634E-186	8	
Kcnd2	6,9367E-190	0,47222	0,756	0,321	1,1647E-185	8	
Uqcrfs1	1,1222E-189	0,58092	0,816	0,466	1,8844E-185	8	
Idnk	3,4242E-189	0,5181	0,651	0,294	5,7495E-185	8	
Swi5	4,9746E-189	0,55627	0,954	0,769	8,3528E-185	8	
Arl3	7,9468E-189	0,56856	0,768	0,411	1,3344E-184	8	
Ndufv2	3,4216E-188	0,58529	0,866	0,53	5,7452E-184	8	
Socs2	5,0782E-188	0,53027	0,69	0,298	8,5267E-184	8	
Psmb6	6,0829E-187	0,57959	0,958	0,726	1,0214E-182	8	
Mrpl52	2,3343E-186	0,55576	0,962	0,724	3,9195E-182	8	

Hddc2	3,4947E-186	0,46602	0,507	0,18	5,8679E-182	8	
Mrps18c	4,8201E-186	0,5136	0,697	0,321	8,0934E-182	8	
Brk1	7,1133E-186	0,57178	0,821	0,474	1,1944E-181	8	
Rpl4	3,7334E-185	0,53254	0,971	0,821	6,2688E-181	8	
Nt5c	5,1646E-185	0,52349	0,667	0,307	8,6719E-181	8	
Smim4	1,1348E-184	0,4437	0,474	0,158	1,9055E-180	8	
Plpp1	3,2433E-184	0,59765	0,773	0,43	5,4459E-180	8	
Mrps33	4,3279E-184	0,56029	0,931	0,728	7,2669E-180	8	
Maged2	5,9848E-184	0,51287	0,66	0,299	1,0049E-179	8	
Trappc2l	9,6219E-184	0,54061	0,732	0,372	1,6156E-179	8	
Bambi	7,7228E-183	0,42352	0,405	0,116	1,2967E-178	8	
Snapc5	1,0964E-182	0,45347	0,508	0,182	1,8409E-178	8	
Commd4	2,383E-182	0,43495	0,485	0,164	4,0014E-178	8	
Ldhb	2,701E-182	0,48567	0,597	0,247	4,5353E-178	8	
Ascl1	3,6458E-181	0,46246	0,55	0,198	6,1216E-177	8	
Tmem14c	7,0439E-181	0,51442	0,611	0,265	1,1827E-176	8	
Bax	6,307E-180	0,52275	0,721	0,36	1,059E-175	8	
Ap2b1	1,5317E-179	0,48733	0,992	0,925	2,5719E-175	8	
Ywhaq	2,317E-179	0,52407	0,991	0,943	3,8905E-175	8	
Snrpd2	3,188E-179	0,57558	0,923	0,693	5,3531E-175	8	
Tmod1	1,4899E-178	0,53808	0,917	0,577	2,5016E-174	8	
Vimp	9,368E-178	0,52393	0,707	0,348	1,573E-173	8	
Pdcd5	6,9068E-177	0,53678	0,951	0,743	1,1597E-172	8	
Ngfr	7,9993E-177	0,51916	0,919	0,483	1,3432E-172	8	
Mrpl42	9,0535E-177	0,48599	0,644	0,292	1,5202E-172	8	
Sumo1	6,4646E-176	0,57625	0,875	0,571	1,0855E-171	8	
Fxr1	1,0703E-175	0,57542	0,931	0,705	1,7971E-171	8	
Dhrs7	1,0853E-175	0,51774	0,699	0,347	1,8223E-171	8	
Txndc17	2,0506E-175	0,56291	0,908	0,646	3,4431E-171	8	
Calm1	2,2369E-175	0,3541	1	1	3,7561E-171	8	
Psma6	4,7368E-175	0,57109	0,85	0,538	7,9535E-171	8	
Psmg4	7,6143E-175	0,50738	0,689	0,333	1,2785E-170	8	
Atp5g3	8,0437E-175	0,53928	0,952	0,76	1,3506E-170	8	
Ptpre	1,0151E-174	0,43982	0,522	0,189	1,7044E-170	8	
Rpl28	1,3366E-174	0,47401	0,999	0,957	2,2444E-170	8	
Fam96b	2,5341E-174	0,51259	0,725	0,373	4,255E-170	8	
Dbh	3,7602E-174	0,26223	0,899	0,631	6,3137E-170	8	
Tomm22	4,2358E-174	0,52454	0,678	0,334	7,1123E-170	8	
Ddx1	8,4488E-174	0,59998	0,874	0,588	1,4186E-169	8	
Chga	1,0734E-173	0,59281	0,881	0,624	1,8024E-169	8	
Ufc1	1,9594E-173	0,5311	0,725	0,377	3,2901E-169	8	
Riiad1	2,298E-173	0,48432	0,577	0,238	3,8586E-169	8	
Timm8b	2,4363E-173	0,554	0,883	0,584	4,0908E-169	8	
Mrpl41	3,5108E-173	0,5293	0,76	0,412	5,895E-169	8	
Hotairm1	4,5266E-173	0,55742	0,704	0,363	7,6006E-169	8	
Ifi27	5,1655E-173	0,60319	0,929	0,76	8,6733E-169	8	
Eno1	7,0992E-173	0,56093	0,828	0,518	1,192E-168	8	
Ndufa11	7,4521E-173	0,5135	0,99	0,864	1,2513E-168	8	

0610012G03	8,6802E-173	0,5322	0,737	0,392	1,4575E-168	8	
Atp5g1	5,9031E-172	0,5037	0,994	0,913	9,9119E-168	8	
Uqcc3	6,789E-172	0,47022	0,596	0,256	1,1399E-167	8	
Timm13	6,8867E-172	0,55295	0,845	0,532	1,1563E-167	8	
Stmn1	1,1808E-171	0,56831	0,975	0,885	1,9827E-167	8	
Crabp1	2,1601E-171	1,11414	0,722	0,436	3,6271E-167	8	
Nabp2	2,5636E-171	0,48174	0,619	0,276	4,3045E-167	8	
Rpl8	6,1778E-171	0,48465	1	0,964	1,0373E-166	8	
Adh5	8,6119E-171	0,55542	0,661	0,316	1,446E-166	8	
Sugt1	5,1374E-170	0,5484	0,78	0,445	8,6262E-166	8	
Pmvk	1,0312E-169	0,55266	0,815	0,471	1,7314E-165	8	
Snx6	1,7625E-169	0,51073	0,689	0,337	2,9595E-165	8	
Cacybp	2,4145E-169	0,55616	0,821	0,497	4,0542E-165	8	
Cops4	2,4587E-169	0,4618	0,601	0,26	4,1285E-165	8	
Commd3	3,661E-169	0,47421	0,585	0,25	6,1471E-165	8	
Hspe1	5,293E-169	0,54013	0,88	0,59	8,8875E-165	8	
Suclg1	1,0919E-168	0,4883	0,701	0,348	1,8334E-164	8	
Crip2	1,1633E-168	0,44959	1	0,992	1,9533E-164	8	
Pdcd10	1,4414E-168	0,56627	0,84	0,545	2,4203E-164	8	
Hras	2,0954E-168	0,53547	0,921	0,687	3,5184E-164	8	
Mrpl33	2,5432E-168	0,57464	0,834	0,52	4,2702E-164	8	
Taf10	8,1757E-168	0,47095	0,662	0,311	1,3728E-163	8	
Vti1b	2,614E-167	0,53684	0,749	0,407	4,3891E-163	8	
Ndufb3	2,7863E-167	0,53733	0,889	0,593	4,6785E-163	8	
Ndufs6	2,8983E-167	0,53104	0,902	0,593	4,8665E-163	8	
Mrps34	3,212E-167	0,45327	0,572	0,235	5,3932E-163	8	
1810058I24R	5,5642E-167	0,50186	0,676	0,333	9,3429E-163	8	
Supt4a	1,6392E-166	0,48592	0,686	0,335	2,7524E-162	8	
Sarnp	1,7222E-166	0,51732	0,739	0,386	2,8918E-162	8	
Dbi	3,0066E-166	0,54747	0,752	0,412	5,0484E-162	8	
Ninj1	9,3672E-166	0,43245	0,483	0,177	1,5729E-161	8	
Mrpl14	1,6921E-165	0,51859	0,717	0,377	2,8412E-161	8	
Ddrgk1	3,2166E-165	0,50318	0,708	0,358	5,4011E-161	8	
Map1lc3b	9,3724E-165	0,53015	0,944	0,733	1,5737E-160	8	
Rpl13a	2,4726E-164	0,43681	1	0,987	4,1518E-160	8	
Clec14a	4,8277E-164	0,48359	0,546	0,218	8,1062E-160	8	
Phactr1	5,1864E-164	0,53464	0,921	0,663	8,7084E-160	8	
Cnpy2	7,485E-164	0,47365	0,648	0,298	1,2568E-159	8	
Polr2g	8,0731E-164	0,5161	0,748	0,412	1,3556E-159	8	
Cyb5a	2,0508E-163	0,55507	0,849	0,549	3,4436E-159	8	
Clic1	9,4695E-163	0,52502	0,952	0,815	1,59E-158	8	
Kcnt2	7,2656E-162	0,41024	0,636	0,263	1,22E-157	8	
Tmed3	7,5948E-162	0,50468	0,641	0,307	1,2752E-157	8	
Pop7	1,2594E-161	0,47297	0,696	0,349	2,1147E-157	8	
Dpysl3	1,4721E-161	0,4387	1	0,989	2,4718E-157	8	
Rerg	4,7501E-161	0,38049	0,351	0,095	7,9759E-157	8	
Mycbp	6,9361E-161	0,48599	0,684	0,337	1,1646E-156	8	
Bglap2	9,2819E-161	0,3395	0,25	0,046	1,5585E-156	8	

Snx17	3,1284E-160	0,45682	0,597	0,266	5,2528E-156	8	
Ufsp2	4,6796E-160	0,48744	0,628	0,296	7,8575E-156	8	
F2rl2	5,2964E-160	0,41722	0,449	0,152	8,8932E-156	8	
Sec11c	6,3815E-160	0,42254	0,473	0,173	1,0715E-155	8	
Mrpl34	9,2518E-160	0,42693	0,555	0,231	1,5535E-155	8	
Sms	1,2798E-159	0,57785	0,828	0,542	2,1488E-155	8	
Rtn4rl1	2,4388E-159	0,38716	0,373	0,105	4,0949E-155	8	
Tmco1	2,5688E-159	0,54777	0,842	0,533	4,3133E-155	8	
Mllt11	6,0358E-159	0,49909	0,982	0,926	1,0135E-154	8	
Snrpg	7,023E-159	0,48702	0,657	0,32	1,1792E-154	8	
Nsa2	1,1448E-158	0,52925	0,923	0,692	1,9223E-154	8	
Scand1	4,4004E-158	0,53417	0,906	0,654	7,3887E-154	8	
Eif3k	1,4854E-157	0,51884	0,919	0,654	2,4941E-153	8	
Tmbim4	5,7619E-157	0,51886	0,746	0,411	9,6748E-153	8	
Hsbp1	3,4536E-156	0,52596	0,912	0,687	5,799E-152	8	
Cycs	4,3027E-156	0,52789	0,775	0,448	7,2247E-152	8	
Psmc6	4,9564E-156	0,52394	0,896	0,654	8,3223E-152	8	
Tma7	9,5184E-156	0,47628	0,972	0,862	1,5982E-151	8	
Mrpl54	3,9325E-155	0,50438	0,8	0,458	6,6031E-151	8	
Akr7a5	4,5617E-155	0,38139	0,424	0,142	7,6596E-151	8	
Hsp90ab1	5,5137E-155	0,29546	1	1	9,2581E-151	8	
Ier3	2,1818E-154	0,47584	0,557	0,242	3,6635E-150	8	
Enpp1	2,564E-154	0,40775	0,615	0,259	4,3051E-150	8	
Mrpl57	3,0732E-154	0,49568	0,71	0,378	5,1602E-150	8	
Sdhd	5,8099E-154	0,46534	0,607	0,281	9,7553E-150	8	
Jtb	7,0064E-154	0,40493	0,487	0,186	1,1765E-149	8	
Rpl18a	2,8703E-153	0,43435	1	0,971	4,8196E-149	8	
Fibcd1	3,7635E-153	0,3842	0,769	0,328	6,3193E-149	8	
Txnl4a	6,8228E-153	0,43048	0,594	0,263	1,1456E-148	8	
Tmem255b	8,1104E-153	0,54202	0,926	0,72	1,3618E-148	8	
Nol3	1,0801E-152	0,47471	0,582	0,265	1,8135E-148	8	
Wls	1,6513E-152	0,49261	0,663	0,322	2,7728E-148	8	
Zbtb8os	2,3181E-152	0,35452	0,369	0,111	3,8923E-148	8	
Mrps21	4,2629E-152	0,4849	0,728	0,393	7,1579E-148	8	
Sfr1	1,083E-151	0,5353	0,878	0,643	1,8185E-147	8	
Snrpe	2,1506E-151	0,50636	0,839	0,53	3,611E-147	8	
Mrpl21	4,1096E-151	0,44876	0,615	0,288	6,9005E-147	8	
Yif1b	4,4135E-151	0,49814	0,743	0,41	7,4108E-147	8	
Psmd8	9,2109E-151	0,52239	0,848	0,561	1,5466E-146	8	
Ybx1	1,1962E-150	0,54565	0,914	0,713	2,0086E-146	8	
Gm561	1,4921E-150	0,43988	0,575	0,259	2,5055E-146	8	
Mrps18a	2,717E-150	0,43005	0,563	0,245	4,562E-146	8	
Carkd	3,2743E-150	0,42803	0,545	0,233	5,498E-146	8	
Hint2	4,0575E-150	0,4377	0,613	0,285	6,813E-146	8	
Entpd3	5,0958E-150	0,44984	0,714	0,34	8,5563E-146	8	
Chrac1	3,8346E-149	0,44007	0,622	0,294	6,4386E-145	8	
Psma1	2,7554E-148	0,50935	0,801	0,486	4,6265E-144	8	
Grpel1	4,3297E-148	0,46014	0,641	0,316	7,2699E-144	8	

Hsd17b11	4,417E-148	0,40701	0,509	0,206	7,4165E-144	8	
Smim11	2,4742E-147	0,36872	0,426	0,149	4,1545E-143	8	
Mrpl11	2,5711E-147	0,42544	0,605	0,282	4,3171E-143	8	
Uqcc2	2,8227E-147	0,50539	0,918	0,672	4,7396E-143	8	
B3glct	2,9573E-147	0,46886	0,553	0,24	4,9655E-143	8	
Ten1	3,2734E-147	0,47472	0,657	0,336	5,4964E-143	8	
Txn1	3,3263E-147	0,4363	0,99	0,896	5,5852E-143	8	
Gm8730	7,1341E-147	0,57134	0,665	0,363	1,1979E-142	8	
Vps29	1,3827E-146	0,5028	0,797	0,494	2,3217E-142	8	
Eif3j1	3,0918E-146	0,4975	0,922	0,702	5,1914E-142	8	
Prdx5	4,3981E-146	0,5109	0,902	0,676	7,3849E-142	8	
Gria3	4,8556E-146	0,38411	0,544	0,215	8,1531E-142	8	
Ostc	7,8423E-146	0,48969	0,681	0,364	1,3168E-141	8	
Grcc10	5,9017E-145	0,38641	0,426	0,152	9,9095E-141	8	
Tmem108	7,7504E-145	0,41764	0,567	0,24	1,3014E-140	8	
Psmb2	1,6327E-144	0,49607	0,899	0,654	2,7414E-140	8	
Emd	3,3054E-144	0,49745	0,775	0,458	5,55E-140	8	
Vdac3	5,7288E-144	0,50843	0,83	0,534	9,6192E-140	8	
Tm4sf1	8,3961E-144	0,51043	0,908	0,715	1,4098E-139	8	
Kif5c	1,6676E-143	0,42515	0,994	0,952	2,8001E-139	8	
Nars	2,2448E-143	0,50304	0,937	0,774	3,7693E-139	8	
Ssna1	1,533E-142	0,45518	0,65	0,331	2,574E-138	8	
Atp6v0b	2,4E-142	0,4191	0,992	0,945	4,0298E-138	8	
Mrpl30	4,1187E-142	0,47131	0,748	0,421	6,9157E-138	8	
Ndufs2	4,9721E-142	0,44722	0,765	0,428	8,3486E-138	8	
1500011K16	6,6426E-142	0,45829	0,628	0,313	1,1154E-137	8	
Denr	4,0945E-141	0,47389	0,771	0,446	6,8751E-137	8	
Eif3m	4,384E-141	0,47545	0,744	0,422	7,3612E-137	8	
Ndufv1	5,7425E-141	0,50441	0,858	0,563	9,6422E-137	8	
Dnlz	6,421E-141	0,42563	0,562	0,257	1,0781E-136	8	
Minos1	6,9702E-141	0,49272	0,896	0,648	1,1704E-136	8	
Eapp	7,6722E-141	0,49951	0,795	0,49	1,2882E-136	8	
Emc7	7,8361E-141	0,4637	0,855	0,569	1,3158E-136	8	
Sars	9,7027E-141	0,49178	0,914	0,674	1,6292E-136	8	
Nhp2	5,7257E-140	0,38971	0,472	0,188	9,6141E-136	8	
Psmb3	7,1615E-140	0,46388	0,949	0,792	1,2025E-135	8	
Mrps36	9,7034E-140	0,42927	0,637	0,316	1,6293E-135	8	
Mrps6	1,3804E-139	0,42792	0,618	0,303	2,3178E-135	8	
Bad	2,3993E-139	0,42004	0,616	0,297	4,0287E-135	8	
Erh	3,6493E-139	0,49104	0,826	0,54	6,1275E-135	8	
Arpc5l	5,405E-139	0,49727	0,758	0,452	9,0755E-135	8	
Eif1	2,7695E-138	0,34244	1	0,998	4,6503E-134	8	
Coq7	2,6295E-137	0,41997	0,64	0,311	4,4152E-133	8	
2610524H06	5,8013E-137	0,43328	0,562	0,26	9,7409E-133	8	
Pop5	5,8833E-137	0,44382	0,665	0,347	9,8786E-133	8	
Pdcd4	8,246E-137	0,55548	0,799	0,513	1,3846E-132	8	
Rtn1	1,3502E-136	0,36385	1	0,999	2,2671E-132	8	
0610011F06	1,4299E-136	0,39867	0,541	0,241	2,401E-132	8	

Esd	1,5393E-136	0,48807	0,801	0,497	2,5847E-132	8	
Marc2	2,2544E-136	0,38913	0,476	0,192	3,7853E-132	8	
Alpl	3,314E-136	0,41827	0,474	0,182	5,5645E-132	8	
Ramp1	4,4027E-136	0,53305	0,796	0,497	7,3926E-132	8	
Pcbd1	8,8556E-136	0,50935	0,773	0,485	1,4869E-131	8	
Cope	1,1075E-135	0,48582	0,825	0,541	1,8596E-131	8	
Pcsk2	1,9123E-135	0,37521	1	1	3,211E-131	8	
Txndc15	2,768E-135	0,41892	0,598	0,288	4,6477E-131	8	
Sertm1	8,1969E-135	0,35946	0,491	0,19	1,3763E-130	8	
Nudt2	1,1851E-134	0,34351	0,398	0,139	1,9899E-130	8	
Cbr1	1,4965E-134	0,44988	0,719	0,397	2,5128E-130	8	
Pnp	2,7929E-134	0,40093	0,538	0,241	4,6895E-130	8	
Ramp2	4,5157E-134	0,4647	0,433	0,169	7,5823E-130	8	
Prr13	1,2094E-133	0,48252	0,771	0,469	2,0307E-129	8	
C1d	4,7275E-133	0,46766	0,756	0,443	7,938E-129	8	
Dnajc19	6,4701E-133	0,40311	0,556	0,255	1,0864E-128	8	
Wdr83os	8,4104E-133	0,41357	0,625	0,31	1,4122E-128	8	
Rps27rt	1,0181E-132	0,31777	0,328	0,098	1,7094E-128	8	
Prelid1	1,0198E-132	0,47348	0,791	0,499	1,7124E-128	8	
Thoc7	1,3936E-132	0,50184	0,823	0,558	2,34E-128	8	
Rab24	2,5932E-132	0,4009	0,573	0,272	4,3542E-128	8	
H3f3b	2,599E-132	0,4546	0,978	0,889	4,364E-128	8	
Mcee	3,5218E-132	0,43458	0,674	0,362	5,9135E-128	8	
Ywhah	4,0332E-132	0,41064	0,998	0,988	6,7721E-128	8	
Rpsa	5,2403E-132	0,41339	1	0,991	8,7991E-128	8	
Qdpr	6,5672E-132	0,50763	0,785	0,501	1,1027E-127	8	
Sv2b	1,0049E-131	0,35895	0,485	0,192	1,6874E-127	8	
1110008F13	1,9007E-131	0,39701	0,486	0,205	3,1914E-127	8	
Med28	2,2815E-131	0,46499	0,748	0,451	3,8309E-127	8	
Isoc1	5,0087E-131	0,50123	0,636	0,338	8,4101E-127	8	
Prmt2	5,5959E-131	0,47414	0,894	0,664	9,396E-127	8	
Emc10	6,2145E-131	0,46992	0,937	0,734	1,0435E-126	8	
Chchd10	1,0561E-130	0,46778	0,535	0,249	1,7733E-126	8	
Thsd7a	1,6968E-130	0,35002	0,522	0,207	2,8492E-126	8	
Mrpl55	5,9082E-130	0,3893	0,538	0,245	9,9205E-126	8	
Rdx	5,9115E-130	0,44782	0,976	0,879	9,9261E-126	8	
Trpm2	7,1257E-130	0,37674	0,446	0,173	1,1965E-125	8	
B230118H07	1,244E-129	0,35016	0,396	0,143	2,0888E-125	8	
Psmc5	1,3415E-129	0,43986	0,941	0,738	2,2526E-125	8	
Cct4	1,8654E-129	0,44365	0,727	0,411	3,1322E-125	8	
Ech1	1,9772E-129	0,43147	0,647	0,341	3,32E-125	8	
Cisd1	3,6785E-129	0,45752	0,905	0,665	6,1766E-125	8	
Gtf2h5	1,492E-128	0,47409	0,841	0,572	2,5053E-124	8	
Dpcd	1,9908E-128	0,41467	0,589	0,288	3,3427E-124	8	
Zc3h15	2,8491E-128	0,4659	0,983	0,886	4,7839E-124	8	
Gm16286	3,373E-128	0,44597	0,668	0,369	5,6637E-124	8	
Mgst3	4,2979E-128	0,40651	0,596	0,294	7,2165E-124	8	
Rpl23a	2,1235E-127	0,47017	0,871	0,622	3,5656E-123	8	

Pafah1b3	2,8178E-127	0,46982	0,792	0,507	4,7314E-123	8	
Spats2l	2,925E-127	0,46949	0,779	0,472	4,9114E-123	8	
Sdf2l1	3,6263E-127	0,34676	0,333	0,107	6,0889E-123	8	
Arl4a	3,7904E-127	0,44635	0,673	0,371	6,3644E-123	8	
Uchl3	3,8283E-127	0,40103	0,574	0,273	6,4282E-123	8	
Chmp5	8,2237E-127	0,4698	0,892	0,685	1,3808E-122	8	
H1fx	1,348E-126	0,48646	0,886	0,648	2,2635E-122	8	
Tbcc	2,9263E-126	0,39212	0,531	0,245	4,9136E-122	8	
Trnp1	3,8059E-126	0,38874	0,536	0,247	6,3905E-122	8	
Fam98c	3,8935E-126	0,39576	0,536	0,247	6,5375E-122	8	
Pfn2	5,3211E-126	0,43947	0,956	0,848	8,9346E-122	8	
Camk4	6,397E-126	0,43208	0,841	0,526	1,0741E-121	8	
Itm2b	1,1845E-125	0,3352	1	1	1,9889E-121	8	
Mrpl43	1,6111E-125	0,41972	0,629	0,327	2,7053E-121	8	
Ranbp1	2,3591E-125	0,46087	0,867	0,638	3,9611E-121	8	
Zcrb1	3,4799E-125	0,46795	0,781	0,5	5,8431E-121	8	
Fam173a	3,7077E-125	0,42274	0,623	0,321	6,2257E-121	8	
Fam195b	4,5769E-125	0,47563	0,851	0,574	7,6851E-121	8	
Bud31	8,9867E-125	0,38846	0,54	0,25	1,509E-120	8	
Tpd52l1	1,3036E-124	0,28474	0,265	0,068	2,1888E-120	8	
Nmt2	1,6808E-124	0,45546	0,939	0,782	2,8222E-120	8	
Ccdc80	1,7514E-124	0,41891	0,577	0,274	2,9407E-120	8	
Smim12	5,3267E-124	0,38563	0,551	0,262	8,944E-120	8	
Serbp1	1,548E-123	0,41402	0,991	0,947	2,5993E-119	8	
Gpr149	1,5596E-123	0,33855	0,667	0,307	2,6187E-119	8	
S100a1	1,5736E-123	0,43003	0,97	0,797	2,6423E-119	8	
Cyb561d2	1,9256E-123	0,35812	0,433	0,172	3,2333E-119	8	
Snw1	2,4729E-123	0,42339	0,753	0,441	4,1523E-119	8	
Svbp	3,5081E-123	0,39203	0,56	0,268	5,8905E-119	8	
Gm28905	4,8194E-123	0,32238	0,345	0,112	8,0923E-119	8	
Wbp11	8,99E-123	0,47792	0,768	0,493	1,5095E-118	8	
Aes	1,0523E-122	0,35717	0,998	0,987	1,7669E-118	8	
Pebp1	2,3746E-122	0,40128	0,995	0,953	3,9873E-118	8	
Cript	2,4804E-122	0,45565	0,804	0,534	4,1648E-118	8	
Guk1	2,5619E-122	0,45433	0,762	0,475	4,3018E-118	8	
Pak1	3,3408E-122	0,46427	0,816	0,533	5,6095E-118	8	
Auts2	6,8541E-122	0,43658	0,912	0,63	1,1509E-117	8	
Cfap36	7,7767E-122	0,4572	0,791	0,513	1,3058E-117	8	
Krtcap2	1,6285E-121	0,46381	0,849	0,598	2,7344E-117	8	
Adk	1,6483E-121	0,34839	0,424	0,168	2,7677E-117	8	
Rpp21	3,5244E-121	0,40759	0,592	0,299	5,9178E-117	8	
Imp3	3,7242E-121	0,436	0,665	0,372	6,2533E-117	8	
Sdf2	6,3987E-121	0,44688	0,741	0,455	1,0744E-116	8	
Cib2	1,0561E-120	0,37016	0,452	0,19	1,7734E-116	8	
Arid5b	1,2457E-120	0,29664	0,295	0,087	2,0916E-116	8	
Nxn	1,9852E-120	0,32398	0,338	0,112	3,3333E-116	8	
Mrpl12	2,5717E-120	0,40304	0,669	0,364	4,3182E-116	8	
Mrps16	3,648E-120	0,3949	0,606	0,313	6,1253E-116	8	

Tlx2	8,0541E-120	0,38043	0,995	0,965	1,3524E-115	8	
Sat1	9,5139E-120	0,4238	0,638	0,343	1,5975E-115	8	
Alad	1,2755E-119	0,38562	0,519	0,237	2,1416E-115	8	
Palmd	1,9209E-119	0,49775	0,667	0,377	3,2253E-115	8	
Ntan1	3,957E-119	0,40737	0,646	0,343	6,6442E-115	8	
Tnni1	3,9646E-119	0,34663	0,424	0,168	6,657E-115	8	
H3f3a	4,4637E-119	0,4101	1	0,984	7,4951E-115	8	
Stmn4	1,0674E-118	0,48329	0,682	0,401	1,7923E-114	8	
Eif2s1	1,2947E-118	0,3909	0,564	0,281	2,1739E-114	8	
Tomm5	1,4678E-118	0,39668	0,637	0,339	2,4646E-114	8	
Kcnv1	1,7153E-118	0,30328	0,344	0,114	2,8801E-114	8	
Lhfpl5	1,8654E-118	0,4119	0,631	0,332	3,1322E-114	8	
Rpl27	1,9618E-118	0,4262	0,727	0,429	3,294E-114	8	
Eif3l	4,6143E-118	0,41473	0,655	0,361	7,7478E-114	8	
Capns1	7,023E-118	0,37531	0,994	0,953	1,1792E-113	8	
Emc6	7,955E-118	0,38271	0,603	0,308	1,3357E-113	8	
Gadd45g	1,032E-117	0,40349	0,491	0,222	1,7328E-113	8	
Tmem261	1,1682E-117	0,34323	0,454	0,191	1,9615E-113	8	
Echs1	1,5563E-117	0,38083	0,614	0,316	2,6131E-113	8	
Srp19	1,7074E-117	0,44225	0,812	0,529	2,8669E-113	8	
Atraid	2,027E-117	0,4125	0,634	0,34	3,4036E-113	8	
Lsm5	6,8327E-117	0,3897	0,574	0,289	1,1473E-112	8	
Abhd17a	6,9558E-117	0,42139	0,721	0,428	1,168E-112	8	
Ccdc107	7,1876E-117	0,37424	0,521	0,245	1,2069E-112	8	
Eif3f	7,3232E-117	0,44578	0,867	0,641	1,2296E-112	8	
Rpgrip1	9,7408E-117	0,35102	0,459	0,194	1,6356E-112	8	
Mkks	1,0608E-116	0,37413	0,549	0,267	1,7812E-112	8	
Metap2	1,3422E-116	0,36845	0,998	0,963	2,2537E-112	8	
Rpf2	1,4689E-116	0,36014	0,561	0,27	2,4664E-112	8	
Yipf1	1,6896E-116	0,34954	0,46	0,196	2,8369E-112	8	
Dnpep	2,9344E-116	0,39213	0,611	0,318	4,9271E-112	8	
Arpp19	3,8888E-116	0,42806	0,928	0,779	6,5296E-112	8	
Psmb7	1,0134E-115	0,43237	0,886	0,655	1,7016E-111	8	
Hspa5	1,31E-115	0,49252	0,928	0,798	2,1997E-111	8	
Anapc13	2,1858E-115	0,40863	0,671	0,374	3,6702E-111	8	
Cct5	2,2389E-115	0,43568	0,884	0,65	3,7593E-111	8	
Fh1	2,3232E-115	0,37272	0,551	0,269	3,9009E-111	8	
2700029M09	2,3412E-115	0,35742	0,511	0,234	3,9311E-111	8	
Fkbp2	3,4287E-115	0,45363	0,902	0,706	5,7571E-111	8	
Abcb1a	3,5362E-115	0,31749	0,295	0,091	5,9376E-111	8	
Slc18a2	4,8154E-115	0,33371	0,479	0,196	8,0855E-111	8	
Tmem159	5,6669E-115	0,34035	0,428	0,176	9,5152E-111	8	
Ric8	5,8625E-115	0,44294	0,712	0,424	9,8437E-111	8	
Mrps14	6,6062E-115	0,38085	0,623	0,336	1,1093E-110	8	
Pkig	7,6246E-115	0,35867	0,509	0,234	1,2803E-110	8	
Psmc4	7,6837E-115	0,44651	0,77	0,494	1,2902E-110	8	
Taldo1	1,0267E-114	0,44924	0,849	0,584	1,724E-110	8	
Kcnk2	1,3015E-114	0,40478	0,985	0,929	2,1854E-110	8	

Snrpc	1,3093E-114	0,36743	0,532	0,252	2,1985E-110	8	
Ift27	2,5621E-114	0,35215	0,495	0,222	4,3019E-110	8	
Phyh	4,7009E-114	0,40202	0,654	0,36	7,8932E-110	8	
Bag1	7,1279E-114	0,44322	0,873	0,649	1,1968E-109	8	
Creb3	9,4003E-114	0,35863	0,537	0,258	1,5784E-109	8	
Aplp1	1,2146E-113	0,38536	0,983	0,933	2,0394E-109	8	
Insm1	1,7548E-113	0,32252	0,494	0,21	2,9465E-109	8	
Uqcrc2	1,9032E-113	0,44026	0,745	0,462	3,1956E-109	8	
Alyref	6,5181E-113	0,35857	0,521	0,248	1,0945E-108	8	
Tmem205	8,5803E-113	0,38008	0,623	0,33	1,4407E-108	8	
Ndufs8	9,5788E-113	0,4179	0,736	0,434	1,6084E-108	8	
Bbip1	9,8626E-113	0,41891	0,681	0,39	1,656E-108	8	
Ntsr1	1,4307E-112	0,32685	0,609	0,282	2,4023E-108	8	
Commd1	2,6841E-112	0,41009	0,694	0,4	4,5068E-108	8	
Psma3	3,54E-112	0,4078	0,958	0,838	5,944E-108	8	
Dnajc15	4,5447E-112	0,35581	0,51	0,237	7,6311E-108	8	
Txndc9	6,6447E-112	0,42295	0,722	0,437	1,1157E-107	8	
Ndufs3	7,5274E-112	0,42331	0,76	0,472	1,2639E-107	8	
Rps19bp1	8,774E-112	0,35039	0,479	0,215	1,4733E-107	8	
Taco1os	9,9671E-112	0,34198	0,401	0,16	1,6736E-107	8	
Mrps26	1,8437E-111	0,35843	0,608	0,314	3,0958E-107	8	
Timm17b	1,8596E-111	0,35218	0,479	0,219	3,1225E-107	8	
Osgep	3,6884E-111	0,31355	0,363	0,135	6,1933E-107	8	
Cdh11	4,6177E-111	0,30284	0,393	0,149	7,7536E-107	8	
1110065P20	5,8976E-111	0,39895	0,635	0,342	9,9027E-107	8	
Lcmt1	6,2666E-111	0,38524	0,614	0,327	1,0522E-106	8	
Comtd1	1,5569E-110	0,29686	0,322	0,109	2,6142E-106	8	
Spint1	2,1502E-110	0,29929	0,302	0,096	3,6104E-106	8	
BC031181	2,6709E-110	0,43284	0,89	0,724	4,4847E-106	8	
Sec11a	3,0875E-110	0,37343	0,614	0,326	5,1842E-106	8	
Ict1	4,6572E-110	0,38694	0,595	0,316	7,8199E-106	8	
Churc1	1,1068E-109	0,38552	0,689	0,386	1,8584E-105	8	
Cda	1,61E-109	0,27035	0,264	0,076	2,7034E-105	8	
Npm1	2,4909E-109	0,39882	0,963	0,87	4,1825E-105	8	
Uqcrc1	3,4394E-109	0,421	0,874	0,62	5,7751E-105	8	
Drap1	3,4474E-109	0,40559	0,887	0,655	5,7886E-105	8	
Pdap1	6,0054E-109	0,3924	0,991	0,927	1,0084E-104	8	
Pigyl	6,0376E-109	0,32865	0,482	0,219	1,0138E-104	8	
Pomp	7,9273E-109	0,40425	0,93	0,728	1,3311E-104	8	
Tmem126a	8,5056E-109	0,36661	0,563	0,284	1,4282E-104	8	
Lsm2	1,5567E-108	0,31097	0,384	0,15	2,6138E-104	8	
Yif1a	2,3847E-108	0,35015	0,497	0,233	4,0041E-104	8	
B9d2	2,6232E-108	0,34155	0,46	0,207	4,4047E-104	8	
Mrpl18	3,851E-108	0,30972	0,416	0,17	6,4663E-104	8	
Psmd10	4,4322E-108	0,31183	0,369	0,142	7,442E-104	8	
Rps12-ps3	4,4859E-108	0,34474	0,434	0,187	7,5323E-104	8	
Mrps12	4,696E-108	0,39686	0,722	0,431	7,8851E-104	8	
Apip	6,413E-108	0,30762	0,373	0,143	1,0768E-103	8	

Abhd11	1,7603E-107	0,30974	0,388	0,155	2,9558E-103	8	
Morn2	5,3272E-107	0,35884	0,514	0,248	8,9449E-103	8	
Ube2a	1,8458E-106	0,36713	0,607	0,325	3,0993E-102	8	
Cwc15	2,5791E-106	0,40254	0,744	0,462	4,3306E-102	8	
Tceb1	2,6587E-106	0,42741	0,815	0,564	4,4643E-102	8	
Cebpzos	3,6211E-106	0,31935	0,452	0,198	6,0803E-102	8	
Mrps28	3,7249E-106	0,30937	0,389	0,154	6,2544E-102	8	
Ssr2	3,7895E-106	0,41295	0,732	0,444	6,3629E-102	8	
2700060E02	6,5656E-106	0,39784	0,742	0,458	1,1024E-101	8	
Gipc1	8,0569E-106	0,36172	0,533	0,267	1,3528E-101	8	
Ccs	8,2809E-106	0,32827	0,446	0,196	1,3905E-101	8	
Bcas2	1,0166E-105	0,42007	0,802	0,537	1,7071E-101	8	
Mrpl27	1,2829E-105	0,37007	0,621	0,336	2,154E-101	8	
Vim	1,4998E-105	0,66838	0,757	0,548	2,5183E-101	8	
H2afz	1,8129E-105	0,39016	0,896	0,684	3,044E-101	8	
Skp1a	2,2105E-105	0,38758	0,977	0,896	3,7117E-101	8	
Adrm1	2,3308E-105	0,38837	0,674	0,392	3,9136E-101	8	
Cd151	4,8125E-105	0,41718	0,843	0,596	8,0806E-101	8	
Glod4	5,3393E-105	0,325	0,397	0,164	8,9651E-101	8	
Shfm1	6,5917E-105	0,35917	0,978	0,915	1,1068E-100	8	
Zfp580	7,0663E-105	0,3481	0,495	0,235	1,1865E-100	8	
Ebp	8,1165E-105	0,37002	0,619	0,338	1,3628E-100	8	
Tmem53	1,2332E-104	0,27162	0,271	0,082	2,0706E-100	8	
Mocs2	2,1601E-104	0,43147	0,795	0,535	3,6271E-100	8	
Tmem120a	3,1718E-104	0,27092	0,28	0,088	5,3257E-100	8	
C1qbp	3,2221E-104	0,41402	0,727	0,442	5,4102E-100	8	
Hmgb1	3,2381E-104	0,34916	0,998	0,97	5,437E-100	8	
Ppp1r14b	3,7543E-104	0,42978	0,778	0,514	6,3039E-100	8	
Hspa2	3,8645E-104	0,34761	0,463	0,212	6,4888E-100	8	
Cdc37	5,0779E-104	0,4216	0,809	0,561	8,5263E-100	8	
Gnai1	5,191E-104	0,37945	0,582	0,312	8,7162E-100	8	
Snhg6	5,2187E-104	0,32059	0,411	0,171	8,7628E-100	8	
Ndufs7	9,8325E-104	0,39776	0,828	0,565	1,651E-99	8	
Wbp5	1,6565E-103	0,37042	0,985	0,933	2,7814E-99	8	
Eef2	1,997E-103	0,36818	0,992	0,94	3,3532E-99	8	
Tagln2	3,1129E-103	0,39082	0,993	0,964	5,2268E-99	8	
Znhit1	3,4041E-103	0,40749	0,884	0,683	5,7159E-99	8	
Rab8b	4,8127E-103	0,42028	0,782	0,517	8,0809E-99	8	
Ssb	5,7658E-103	0,38794	0,951	0,816	9,6814E-99	8	
Zfp771	7,0072E-103	0,35383	0,568	0,296	1,17658E-98	8	
Ppp2r3c	1,0358E-102	0,36888	0,553	0,288	1,73924E-98	8	
Snrnp27	1,1223E-102	0,37941	0,973	0,87	1,88438E-98	8	
Tbx3os2	1,3293E-102	0,26149	0,271	0,083	2,23208E-98	8	
Nelfe	1,7687E-102	0,35046	0,531	0,268	2,96981E-98	8	
Rpp25l	2,1398E-102	0,30565	0,408	0,171	3,59292E-98	8	
Tsr3	2,2268E-102	0,30683	0,414	0,175	3,73909E-98	8	
Pdzd11	4,4557E-102	0,31697	0,452	0,203	7,48158E-98	8	
Timm17a	6,554E-102	0,40531	0,716	0,444	1,10049E-97	8	

Sra1	8,5509E-102	0,38374	0,668	0,389	1,43578E-97	8	
Snrpb2	9,8567E-102	0,38192	0,661	0,384	1,65504E-97	8	
Ssu72	1,1528E-101	0,40002	0,907	0,716	1,93558E-97	8	
1810037I17F	1,5905E-101	0,41143	0,81	0,559	2,67057E-97	8	
Commd6	3,82E-101	0,32416	0,469	0,22	6,41413E-97	8	
Ptrhd1	3,9799E-101	0,31491	0,385	0,158	6,68265E-97	8	
Polr2f	6,8637E-101	0,40117	0,752	0,477	1,15249E-96	8	
1110008P14	7,4473E-101	0,41177	0,833	0,588	1,25047E-96	8	
Rnh1	1,0891E-100	0,36116	0,587	0,313	1,82868E-96	8	
Tm2d3	1,5918E-100	0,33858	0,534	0,268	2,67286E-96	8	
Chchd7	1,7913E-100	0,31547	0,413	0,178	3,00776E-96	8	
Zfpl1	3,947E-100	0,27996	0,348	0,132	6,62738E-96	8	
Mrps7	5,7027E-100	0,34282	0,512	0,254	9,57546E-96	8	
Polr2k	6,0452E-100	0,40128	0,772	0,506	1,01504E-95	8	
Fbxl15	7,4789E-100	0,30408	0,365	0,145	1,25578E-95	8	
Elof1	7,8447E-100	0,36099	0,645	0,364	1,3172E-95	8	
2810428I15F	1,0559E-99	0,41243	0,831	0,578	1,77294E-95	8	
Gtf3c6	1,0562E-99	0,32284	0,424	0,187	1,77355E-95	8	
Rab2a	1,0725E-99	0,38486	0,967	0,882	1,80076E-95	8	
Tmem242	1,3593E-99	0,35567	0,558	0,294	2,28239E-95	8	
Surf1	1,3781E-99	0,34162	0,505	0,248	2,31389E-95	8	
Th	1,5231E-99	0,64381	0,529	0,27	2,55749E-95	8	
Rpap3	1,6133E-99	0,37403	0,633	0,358	2,70882E-95	8	
Apopt1	1,7342E-99	0,34989	0,617	0,338	2,91196E-95	8	
Galnt6	2,202E-99	0,30273	0,415	0,169	3,69744E-95	8	
Eef1g	3,0834E-99	0,36959	0,963	0,841	5,17729E-95	8	
Med30	3,251E-99	0,30448	0,394	0,164	5,45882E-95	8	
Thyn1	4,0172E-99	0,29087	0,355	0,138	6,74534E-95	8	
Bcap31	4,2337E-99	0,389	0,726	0,448	7,10882E-95	8	
Timm10b	4,76E-99	0,31418	0,426	0,188	7,99248E-95	8	
Ube2s	6,4674E-99	0,3849	0,922	0,763	1,08594E-94	8	
Immp1l	6,7942E-99	0,30166	0,36	0,142	1,14082E-94	8	
Rer1	1,36292E-98	0,39136	0,715	0,446	2,28848E-94	8	
Mrpl13	1,77324E-98	0,36706	0,631	0,358	2,97745E-94	8	
Lyrm2	1,85236E-98	0,346	0,546	0,282	3,1103E-94	8	
Nsmce1	2,27227E-98	0,35542	0,615	0,341	3,81538E-94	8	
Chl1	3,67275E-98	0,33592	0,949	0,734	6,16691E-94	8	
Lsm6	6,39835E-98	0,36346	0,57	0,307	1,07435E-93	8	
Atg12	8,09014E-98	0,39979	0,816	0,574	1,35842E-93	8	
Fsd1	8,87482E-98	0,31941	0,448	0,206	1,49017E-93	8	
Map7d2	9,25772E-98	0,43649	0,834	0,612	1,55446E-93	8	
Emc2	1,42885E-97	0,37081	0,662	0,388	2,39919E-93	8	
Psmd2	1,59919E-97	0,39733	0,905	0,743	2,6852E-93	8	
Erp29	1,91806E-97	0,38703	0,922	0,76	3,22061E-93	8	
Nucb2	3,77514E-97	0,43034	0,709	0,45	6,33884E-93	8	
Slc25a5	4,15594E-97	0,38655	0,736	0,462	6,97823E-93	8	
Blvrb	4,76647E-97	0,33119	0,476	0,227	8,00338E-93	8	
Bola3	5,89965E-97	0,36234	0,616	0,345	9,90611E-93	8	

Dnajb11	1,74353E-96	0,34583	0,56	0,296	2,92757E-92	8	
Fam213a	1,90025E-96	0,2697	0,31	0,111	3,19071E-92	8	
Gpx4	2,00537E-96	0,33783	1	0,97	3,36722E-92	8	
Nedd8	2,71355E-96	0,31688	0,998	0,973	4,55632E-92	8	
Tes	4,29674E-96	0,33959	0,517	0,256	7,21466E-92	8	
Slc50a1	7,02579E-96	0,34576	0,581	0,315	1,1797E-91	8	
Caml	7,56862E-96	0,38609	0,732	0,461	1,27085E-91	8	
Rufy3	8,18094E-96	0,3028	0,999	0,989	1,37366E-91	8	
Arl2	9,90728E-96	0,4141	0,771	0,524	1,66353E-91	8	
Eif3i	1,11648E-95	0,39702	0,811	0,565	1,87469E-91	8	
Cstb	1,4071E-95	0,37796	0,633	0,369	2,36266E-91	8	
Ethe1	2,15237E-95	0,25107	0,287	0,098	3,61404E-91	8	
Tgfb1i1	3,09272E-95	0,37883	0,717	0,443	5,19298E-91	8	
Ndufaf3	3,95604E-95	0,35094	0,546	0,29	6,64259E-91	8	
Ebpl	5,06733E-95	0,32859	0,451	0,21	8,50856E-91	8	
Ift74	5,59208E-95	0,34164	0,527	0,272	9,38966E-91	8	
Dnajb6	6,8389E-95	0,3606	0,983	0,927	1,14832E-90	8	
Ppdpf	7,7336E-95	0,34376	0,521	0,267	1,29855E-90	8	
Nsrp1	8,43939E-95	0,36445	0,63	0,36	1,41706E-90	8	
Hspb11	1,42182E-94	0,31527	0,415	0,186	2,38738E-90	8	
Zfp637	3,24415E-94	0,32856	0,488	0,241	5,44724E-90	8	
Serf2	3,51015E-94	0,3631	0,969	0,868	5,89388E-90	8	
Gm5914	3,52107E-94	0,34514	0,501	0,251	5,91222E-90	8	
Polr2c	3,92038E-94	0,35932	0,619	0,355	6,5827E-90	8	
Tbrg1	3,96823E-94	0,37046	0,713	0,436	6,66305E-90	8	
Larp7	4,27811E-94	0,34007	0,506	0,255	7,18338E-90	8	
Psma5	4,76189E-94	0,39058	0,788	0,536	7,9957E-90	8	
Slc25a11	5,31495E-94	0,36796	0,661	0,393	8,92434E-90	8	
Rtcb	5,40399E-94	0,34512	0,649	0,371	9,07384E-90	8	
Acot9	5,93604E-94	0,32478	0,482	0,236	9,96721E-90	8	
Borcs8	7,59775E-94	0,29715	0,43	0,194	1,27574E-89	8	
Tmem199	8,11542E-94	0,3291	0,486	0,241	1,36266E-89	8	
Tex264	9,35254E-94	0,36094	0,573	0,317	1,57038E-89	8	
Crem	9,95849E-94	0,36425	0,59	0,329	1,67213E-89	8	
Pard6a	1,06194E-93	0,30709	0,424	0,191	1,7831E-89	8	
Sar1b	1,36077E-93	0,3519	0,601	0,338	2,28486E-89	8	
Sdc2	1,60602E-93	0,32423	0,438	0,204	2,69667E-89	8	
Arhgap22	1,65745E-93	0,33814	0,61	0,334	2,78302E-89	8	
Ctnnbl1	1,97102E-93	0,32152	0,486	0,239	3,30954E-89	8	
Mrpl15	2,23144E-93	0,32714	0,53	0,274	3,74682E-89	8	
Snrpb	2,30062E-93	0,38599	0,856	0,636	3,86298E-89	8	
Slc9b2	3,49218E-93	0,26686	0,308	0,112	5,86372E-89	8	
Rit2	3,70057E-93	0,38841	0,797	0,535	6,21362E-89	8	
1110001J03F	4,2499E-93	0,38422	0,7	0,438	7,13601E-89	8	
Rpl31	4,43112E-93	0,36247	0,972	0,858	7,44029E-89	8	
Dnajc2	5,37152E-93	0,32689	0,519	0,264	9,01932E-89	8	
Pdia3	9,19773E-93	0,40162	0,917	0,785	1,54439E-88	8	
Pdia6	1,3561E-92	0,39382	0,712	0,457	2,27703E-88	8	

Glrx2	1,50826E-92	0,35467	0,646	0,374	2,53252E-88	8	
Mea1	1,52445E-92	0,32986	0,584	0,318	2,5597E-88	8	
Znhit2	1,66791E-92	0,2997	0,423	0,192	2,80058E-88	8	
Hacd1	1,83486E-92	0,32079	0,514	0,261	3,08091E-88	8	
Tceal3	1,84425E-92	0,42128	0,714	0,458	3,09668E-88	8	
Acads	2,03479E-92	0,30856	0,386	0,168	3,41661E-88	8	
Cdk2ap1	3,50801E-92	0,34769	0,515	0,269	5,89031E-88	8	
Sf3b5	3,53949E-92	0,383	0,69	0,429	5,94316E-88	8	
Cpne5	4,70367E-92	0,2918	0,44	0,197	7,89794E-88	8	
Ufm1	4,79905E-92	0,37501	0,668	0,409	8,05809E-88	8	
Dach1	5,75381E-92	0,27036	0,333	0,126	9,66123E-88	8	
Pcmt1	7,86242E-92	0,39297	0,887	0,716	1,32018E-87	8	
Cmbl	7,93753E-92	0,39301	0,622	0,359	1,33279E-87	8	
Lmna	8,09583E-92	0,37804	0,956	0,858	1,35937E-87	8	
Ap1s1	1,18382E-91	0,3835	0,796	0,548	1,98776E-87	8	
S100a16	1,2051E-91	0,41224	0,954	0,899	2,02348E-87	8	
Ostf1	1,35875E-91	0,26917	0,324	0,123	2,28147E-87	8	
Lias	4,06404E-91	0,34446	0,581	0,321	6,82393E-87	8	
Polr2j	4,26696E-91	0,35962	0,71	0,441	7,16464E-87	8	
Ncl	7,18852E-91	0,34931	0,996	0,955	1,20702E-86	8	
Vamp8	7,90101E-91	0,30963	0,362	0,152	1,32666E-86	8	
Asns	9,78821E-91	0,32856	0,55	0,292	1,64354E-86	8	
Lsm4	1,1764E-90	0,34813	0,651	0,383	1,97529E-86	8	
2310039H08	1,53969E-90	0,26233	0,302	0,111	2,58529E-86	8	
Pex16	2,5486E-90	0,27026	0,337	0,133	4,27935E-86	8	
Abat	3,94294E-90	0,2677	0,342	0,134	6,62059E-86	8	
Ankra2	4,24373E-90	0,39742	0,862	0,659	7,12564E-86	8	
Rftn1	8,10367E-90	0,27744	0,358	0,146	1,36069E-85	8	
Rbm22	1,84736E-89	0,36254	0,645	0,385	3,10191E-85	8	
Serf1	2,04287E-89	0,37627	0,928	0,78	3,43019E-85	8	
Tmem147	2,50758E-89	0,3696	0,769	0,507	4,21047E-85	8	
Manbal	2,94516E-89	0,37028	0,689	0,426	4,94522E-85	8	
Mrpl40	3,03545E-89	0,30741	0,439	0,208	5,09682E-85	8	
Dph3	4,28827E-89	0,33209	0,562	0,308	7,20043E-85	8	
Gsn	8,23737E-89	0,31441	0,477	0,236	1,38314E-84	8	
Pop4	9,26484E-89	0,3096	0,497	0,252	1,55566E-84	8	
Emc4	1,11598E-88	0,35522	0,65	0,388	1,87385E-84	8	
Pcsk1	2,61758E-88	0,44446	0,811	0,61	4,39517E-84	8	
Mecr	6,22073E-88	0,25186	0,279	0,098	1,04452E-83	8	
Lamp1	9,02516E-88	0,35885	0,911	0,766	1,51542E-83	8	
Nipsnap1	1,08661E-87	0,34288	0,603	0,348	1,82453E-83	8	
Ak6	1,14244E-87	0,26701	0,323	0,126	1,91827E-83	8	
Diablo	1,56165E-87	0,30953	0,997	0,974	2,62217E-83	8	
Stx8	2,29429E-87	0,28401	0,404	0,183	3,85235E-83	8	
Fez1	2,5461E-87	0,39425	0,913	0,777	4,27515E-83	8	
Chchd4	3,30424E-87	0,32437	0,554	0,3	5,54816E-83	8	
Ndufa8	3,64891E-87	0,38031	0,844	0,624	6,12689E-83	8	
Pold4	3,67521E-87	0,27751	0,33	0,132	6,17105E-83	8	

Zmat2	5,30441E-87	0,37452	0,817	0,583	8,90664E-83	8	
Snf8	6,02495E-87	0,33872	0,603	0,349	1,01165E-82	8	
Fuom	8,3196E-87	0,28008	0,409	0,184	1,39694E-82	8	
Sumo2	8,72404E-87	0,34652	0,986	0,934	1,46485E-82	8	
Jagn1	9,0947E-87	0,30591	0,447	0,217	1,52709E-82	8	
Psmc2	9,33203E-87	0,35391	0,908	0,721	1,56694E-82	8	
Utp3	1,01703E-86	0,30095	0,487	0,247	1,70769E-82	8	
Eif3g	1,08246E-86	0,34039	0,687	0,415	1,81756E-82	8	
Ppp2r2d	1,88249E-86	0,35617	0,648	0,393	3,16089E-82	8	
Babam1	1,92526E-86	0,32202	0,531	0,284	3,23271E-82	8	
Npr1	1,98328E-86	0,26243	0,593	0,301	3,33013E-82	8	
Chchd6	2,6487E-86	0,30394	0,508	0,26	4,44743E-82	8	
Ndufa9	2,85386E-86	0,34036	0,602	0,345	4,79192E-82	8	
Fdx1	3,07304E-86	0,29608	0,452	0,22	5,15994E-82	8	
Tmem130	3,89407E-86	0,33531	0,785	0,52	6,53853E-82	8	
Pts	4,2213E-86	0,30176	0,428	0,204	7,08799E-82	8	
Vstm2l	6,00601E-86	0,39021	0,871	0,692	1,00847E-81	8	
Mfap2	6,36665E-86	0,35283	0,503	0,264	1,06902E-81	8	
Psmd14	6,90553E-86	0,35414	0,666	0,41	1,15951E-81	8	
Fnta	8,16967E-86	0,32642	0,55	0,301	1,37177E-81	8	
Cox16	1,12667E-85	0,30468	0,475	0,237	1,89179E-81	8	
Rpl15	1,14927E-85	0,37018	0,68	0,426	1,92974E-81	8	
H2-D1	1,32145E-85	0,38305	0,887	0,712	2,21884E-81	8	
Rfc3	1,35392E-85	0,26591	0,33	0,132	2,27337E-81	8	
Npdc1	1,85341E-85	0,34804	0,974	0,892	3,11206E-81	8	
Malsu1	1,9711E-85	0,29398	0,444	0,214	3,30968E-81	8	
Galk1	2,07886E-85	0,27334	0,353	0,148	3,49061E-81	8	
Mrpl48	2,77462E-85	0,35043	0,745	0,48	4,65887E-81	8	
Saysd1	2,79714E-85	0,26352	0,327	0,131	4,69667E-81	8	
Tmem176b	3,65399E-85	0,32922	0,981	0,931	6,13542E-81	8	
Pgf	4,25821E-85	0,35054	0,517	0,276	7,14996E-81	8	
Atp6v1g1	4,31851E-85	0,33215	0,967	0,914	7,25121E-81	8	
Tmed10	5,10865E-85	0,3673	0,878	0,698	8,57793E-81	8	
Mid1ip1	5,37631E-85	0,38315	0,773	0,542	9,02736E-81	8	
Pigp	5,55419E-85	0,32822	0,561	0,312	9,32604E-81	8	
Ccdc137	8,2648E-85	0,31894	0,561	0,309	1,38774E-80	8	
Timm22	1,20937E-84	0,3186	0,528	0,286	2,03066E-80	8	
Gtf3a	1,43356E-84	0,30839	0,494	0,254	2,40709E-80	8	
Gnptg	2,89331E-84	0,32234	0,562	0,315	4,85816E-80	8	
Snrnp25	3,01774E-84	0,25102	0,288	0,107	5,0671E-80	8	
Dctpp1	3,82492E-84	0,2563	0,293	0,11	6,42242E-80	8	
Gamt	4,08017E-84	0,27521	0,374	0,163	6,85101E-80	8	
Smap1	4,55033E-84	0,38457	0,797	0,582	7,64046E-80	8	
Hspb1	5,00407E-84	0,33159	0,496	0,252	8,40233E-80	8	
Aamdc	9,60818E-84	0,29208	0,423	0,201	1,61331E-79	8	
Lnx1	1,2107E-83	0,28659	0,38	0,169	2,03289E-79	8	
Snrpa1	1,44608E-83	0,25846	0,356	0,151	2,42812E-79	8	
Plekhb1	1,56093E-83	0,26954	0,272	0,098	2,62095E-79	8	

Ddx21	2,70095E-83	0,37421	0,715	0,478	4,53517E-79	8	
Atp7a	3,26772E-83	0,27852	0,418	0,191	5,48684E-79	8	
Hist1h4d	3,77E-83	0,35592	0,603	0,356	6,33021E-79	8	
Lrrc3	3,809E-83	0,25035	0,668	0,387	6,39569E-79	8	
Hmg20b	6,34314E-83	0,31782	0,552	0,309	1,06508E-78	8	
Ift43	8,42442E-83	0,27634	0,386	0,174	1,41454E-78	8	
Rab4a	8,61102E-83	0,32439	0,586	0,334	1,44588E-78	8	
Fam213b	1,324E-82	0,25693	0,333	0,136	2,22312E-78	8	
2300009A05	1,47862E-82	0,28149	0,462	0,229	2,48275E-78	8	
Spsb2	1,48377E-82	0,2571	0,288	0,109	2,49141E-78	8	
Cgref1	2,19929E-82	0,27434	0,365	0,159	3,69283E-78	8	
Flywch2	3,16839E-82	0,32317	0,53	0,29	5,32005E-78	8	
Ap3s1	3,44966E-82	0,35688	0,798	0,573	5,79233E-78	8	
Serpinf1	3,717E-82	0,43501	0,415	0,204	6,24121E-78	8	
Arl6ip4	4,80252E-82	0,30586	0,549	0,303	8,06391E-78	8	
Pin1	5,00882E-82	0,35402	0,687	0,438	8,41031E-78	8	
Fam174a	5,91543E-82	0,35291	0,691	0,438	9,9326E-78	8	
Timm10	8,56799E-82	0,30731	0,515	0,275	1,43865E-77	8	
1700023F06	9,70686E-82	0,33089	0,584	0,338	1,62988E-77	8	
Cox19	1,18829E-81	0,30595	0,503	0,268	1,99526E-77	8	
1700123O20	1,22723E-81	0,2833	0,423	0,204	2,06064E-77	8	
Triap1	1,73424E-81	0,29339	0,448	0,223	2,91196E-77	8	
Rps27l	2,52499E-81	0,3593	0,801	0,573	4,23971E-77	8	
Alg5	2,5742E-81	0,27353	0,386	0,176	4,32233E-77	8	
Cpm	2,84764E-81	0,25918	0,346	0,143	4,78148E-77	8	
Sucla2	3,41062E-81	0,351	0,72	0,468	5,72677E-77	8	
Pkm	3,77011E-81	0,31271	0,993	0,946	6,3304E-77	8	
Tmem59	3,96607E-81	0,36738	0,926	0,829	6,65942E-77	8	
Kcnq5	5,08164E-81	0,25124	0,355	0,149	8,53258E-77	8	
Ddah2	6,13012E-81	0,36437	0,693	0,438	1,02931E-76	8	
Gde1	7,24509E-81	0,35897	0,773	0,523	1,21652E-76	8	
Ccdc184	1,07317E-80	0,32565	0,585	0,336	1,80195E-76	8	
Ap4s1	1,20911E-80	0,28341	0,419	0,2	2,03022E-76	8	
Fibp	1,56883E-80	0,29223	0,496	0,26	2,63422E-76	8	
Kcnj5	2,21918E-80	0,25025	0,358	0,151	3,72622E-76	8	
Morf4l1	3,13495E-80	0,27083	0,999	0,999	5,2639E-76	8	
Cpne8	3,79545E-80	0,31294	0,456	0,233	6,37294E-76	8	
Erlec1	6,6831E-80	0,32113	0,548	0,308	1,12216E-75	8	
N6amt2	1,43184E-79	0,28838	0,489	0,254	2,4042E-75	8	
Slit2	2,90021E-79	0,29253	0,645	0,371	4,86974E-75	8	
Adgra1	3,48071E-79	0,26349	0,344	0,146	5,84446E-75	8	
Dtd1	4,02696E-79	0,29778	0,508	0,271	6,76167E-75	8	
Zcchc9	8,55499E-79	0,34687	0,775	0,54	1,43647E-74	8	
Mtfr1l	8,5867E-79	0,28849	0,46	0,235	1,44179E-74	8	
Endog	9,00158E-79	0,25339	0,32	0,132	1,51146E-74	8	
Rnf7	1,1754E-78	0,36097	0,833	0,648	1,97362E-74	8	
Anapc5	2,58948E-78	0,33487	0,937	0,829	4,34799E-74	8	
Psmd7	2,76132E-78	0,35048	0,879	0,689	4,63654E-74	8	

Fundc2	3,75625E-78	0,33256	0,669	0,415	6,30712E-74	8	
Praf2	3,77224E-78	0,29778	0,525	0,286	6,33397E-74	8	
Mettl9	4,15231E-78	0,28695	0,499	0,266	6,97215E-74	8	
Spint2	5,06069E-78	0,31377	0,488	0,26	8,4974E-74	8	
Tceal8	6,31748E-78	0,34548	0,691	0,447	1,06077E-73	8	
Haghl	7,0423E-78	0,30628	0,501	0,273	1,18247E-73	8	
Dph6	7,83204E-78	0,29323	0,496	0,264	1,31508E-73	8	
Wdr45b	8,02891E-78	0,35921	0,861	0,67	1,34813E-73	8	
Hist3h2a	8,77071E-78	0,29336	0,414	0,202	1,47269E-73	8	
Gnai2	8,97961E-78	0,32037	0,987	0,955	1,50777E-73	8	
Acaa1a	1,02427E-77	0,27623	0,409	0,196	1,71986E-73	8	
Hexa	1,03624E-77	0,28611	0,42	0,205	1,73994E-73	8	
Tm2d2	1,32085E-77	0,32038	0,539	0,303	2,21784E-73	8	
Acbd6	1,35038E-77	0,30963	0,611	0,365	2,26743E-73	8	
Chchd3	2,03946E-77	0,26475	0,375	0,172	3,42446E-73	8	
Tbc1d7	2,98209E-77	0,26494	0,373	0,171	5,00723E-73	8	
Fam132a	4,05571E-77	0,28277	0,387	0,183	6,80994E-73	8	
Fhod3	4,61424E-77	0,32871	0,969	0,89	7,74777E-73	8	
2310036O22	4,80851E-77	0,33522	0,91	0,782	8,07397E-73	8	
Mrpl1	6,04072E-77	0,27366	0,389	0,183	1,0143E-72	8	
Smim8	6,28422E-77	0,27606	0,417	0,203	1,05518E-72	8	
Fcf1	9,00602E-77	0,26937	0,426	0,21	1,5122E-72	8	
Psmc1	1,29131E-76	0,35331	0,833	0,603	2,16824E-72	8	
Mrfap1	1,48625E-76	0,35435	0,901	0,755	2,49556E-72	8	
Mrps18b	2,35524E-76	0,2724	0,398	0,19	3,95468E-72	8	
Eid1	2,59243E-76	0,33874	0,972	0,897	4,35295E-72	8	
1110032A03	4,38906E-76	0,28189	0,426	0,212	7,36966E-72	8	
Mvp	4,6968E-76	0,28648	0,512	0,275	7,8864E-72	8	
Dcun1d5	5,09569E-76	0,32918	0,593	0,363	8,55617E-72	8	
Mdh1	5,94123E-76	0,30618	0,974	0,929	9,97593E-72	8	
Atp5a1	1,44944E-75	0,32063	0,956	0,858	2,43376E-71	8	
Nagk	1,75875E-75	0,3436	0,88	0,727	2,95312E-71	8	
Cystm1	1,89388E-75	0,35872	0,874	0,726	3,18002E-71	8	
Acsl5	1,10177E-74	0,30007	0,52	0,292	1,84999E-70	8	
Rbm8a	1,44373E-74	0,33678	0,877	0,705	2,42416E-70	8	
4921524J17R	2,11931E-74	0,31893	0,614	0,372	3,55854E-70	8	
Mrps15	2,55624E-74	0,29148	0,545	0,308	4,29218E-70	8	
BC029214	2,63378E-74	0,29988	0,513	0,284	4,42238E-70	8	
Gspt1	2,77709E-74	0,31349	0,557	0,326	4,66301E-70	8	
Cxx1a	3,05112E-74	0,32127	0,927	0,769	5,12314E-70	8	
Psmb8	3,44322E-74	0,35395	0,836	0,621	5,78151E-70	8	
B4gat1	4,36905E-74	0,28165	0,524	0,287	7,33608E-70	8	
Cuta	4,4984E-74	0,3087	0,661	0,413	7,55326E-70	8	
Alkbh7	4,52977E-74	0,254	0,394	0,188	7,60593E-70	8	
Mrpl35	4,98717E-74	0,2799	0,503	0,273	8,37396E-70	8	
Ercc1	6,90819E-74	0,26134	0,397	0,192	1,15995E-69	8	
Crot	7,30111E-74	0,27797	0,448	0,231	1,22593E-69	8	
Lrpap1	9,96674E-74	0,34262	0,856	0,675	1,67352E-69	8	

Slc35b1	1,05069E-73	0,30908	0,593	0,355	1,76422E-69	8	
Inafm1	1,1157E-73	0,29636	0,551	0,315	1,87337E-69	8	
Dpy30	1,718E-73	0,29987	0,56	0,323	2,88469E-69	8	
Exoc7	2,17933E-73	0,28409	0,512	0,282	3,65932E-69	8	
Nhp2l1	2,51973E-73	0,34933	0,796	0,575	4,23088E-69	8	
Gemin7	3,4734E-73	0,29988	0,557	0,323	5,83218E-69	8	
Acadl	3,72117E-73	0,3337	0,895	0,73	6,24822E-69	8	
Eci2	4,28674E-73	0,27729	0,46	0,241	7,19787E-69	8	
Fam104a	4,37907E-73	0,29844	0,593	0,354	7,3529E-69	8	
Xaf1	6,01293E-73	0,30989	0,633	0,39	1,00963E-68	8	
Ucp2	6,24123E-73	0,33602	0,67	0,419	1,04797E-68	8	
Coq2	6,48124E-73	0,29372	0,533	0,303	1,08827E-68	8	
Ctnnbip1	9,36836E-73	0,32942	0,68	0,444	1,57304E-68	8	
Acot13	1,11784E-72	0,26404	0,426	0,214	1,87696E-68	8	
Eif2b5	1,15559E-72	0,27999	0,498	0,272	1,94035E-68	8	
Cdc123	1,71354E-72	0,31763	0,619	0,389	2,87721E-68	8	
Mphosph8	1,90208E-72	0,34029	0,773	0,542	3,19378E-68	8	
Mrps25	2,09475E-72	0,28627	0,564	0,327	3,51729E-68	8	
Llph	2,34961E-72	0,34837	0,742	0,508	3,94523E-68	8	
Eif3j2	2,84844E-72	0,2707	0,314	0,136	4,78282E-68	8	
Ssbp1	3,10026E-72	0,31135	0,636	0,397	5,20565E-68	8	
Ino80b	3,23661E-72	0,31259	0,609	0,376	5,43458E-68	8	
Eif3e	3,81985E-72	0,32401	0,76	0,517	6,41392E-68	8	
Atp1a1	4,42211E-72	0,32714	1	0,996	7,42516E-68	8	
Wdr61	4,46963E-72	0,32734	0,708	0,469	7,50496E-68	8	
AI413582	4,77475E-72	0,26707	0,432	0,22	8,01728E-68	8	
Pdcd6	6,11755E-72	0,32643	0,592	0,366	1,0272E-67	8	
Immt	9,53334E-72	0,34546	0,707	0,486	1,60074E-67	8	
Leo1	1,27454E-71	0,27777	0,48	0,261	2,14008E-67	8	
Naa10	1,32643E-71	0,29258	0,514	0,288	2,22722E-67	8	
Hmgn3	1,42047E-71	0,31577	0,949	0,851	2,38511E-67	8	
Camk2n2	2,38372E-71	0,33213	0,808	0,578	4,00251E-67	8	
Map2k2	2,87951E-71	0,34012	0,797	0,579	4,83498E-67	8	
Mydgf	3,06105E-71	0,31195	0,615	0,381	5,1398E-67	8	
Alkbh6	4,21273E-71	0,27508	0,383	0,187	7,0736E-67	8	
Sssca1	4,79146E-71	0,28732	0,505	0,281	8,04535E-67	8	
Sap18	6,33288E-71	0,28635	0,543	0,313	1,06335E-66	8	
Emc9	7,9746E-71	0,28851	0,59	0,35	1,33902E-66	8	
Arxes1	8,32522E-71	0,27718	0,46	0,247	1,39789E-66	8	
Cops7a	8,46636E-71	0,30491	0,567	0,339	1,42159E-66	8	
Apbb1	1,2337E-70	0,29424	0,986	0,932	2,07151E-66	8	
Dlk1	1,49931E-70	0,26972	0,284	0,114	2,51749E-66	8	
Mrpl22	1,76444E-70	0,27301	0,425	0,219	2,96267E-66	8	
Magoh	2,62059E-70	0,28637	0,53	0,306	4,40023E-66	8	
Ydjc	2,70786E-70	0,27532	0,425	0,219	4,54676E-66	8	
Asna1	3,49215E-70	0,28331	0,503	0,283	5,86367E-66	8	
Slc7a7	4,40474E-70	0,2515	0,383	0,185	7,396E-66	8	
Fstl5	4,52412E-70	0,28453	0,452	0,237	7,59645E-66	8	

2210013O21	4,83225E-70	0,31027	0,774	0,544	8,11383E-66	8	
Sae1	4,88473E-70	0,28463	0,56	0,33	8,20195E-66	8	
Polr2e	5,69584E-70	0,29312	0,584	0,348	9,56389E-66	8	
Syf2	6,63392E-70	0,32083	0,778	0,549	1,1139E-65	8	
Mrps5	9,85573E-70	0,30972	0,701	0,456	1,65488E-65	8	
St13	1,06541E-69	0,30013	0,966	0,908	1,78894E-65	8	
Mrps31	1,17349E-69	0,26046	0,403	0,202	1,9704E-65	8	
Parl	1,39161E-69	0,28546	0,504	0,285	2,33666E-65	8	
Bzw2	1,81383E-69	0,287	0,489	0,273	3,04559E-65	8	
Egfl7	1,91282E-69	0,252	0,349	0,162	3,21182E-65	8	
Ier3ip1	2,02427E-69	0,33682	0,78	0,581	3,39895E-65	8	
Suox	2,27032E-69	0,27062	0,462	0,248	3,81209E-65	8	
Pdhb	2,2835E-69	0,31219	0,679	0,444	3,83423E-65	8	
Dynlrb1	3,34683E-69	0,27796	0,997	0,965	5,61966E-65	8	
Cyth2	3,40165E-69	0,30361	0,59	0,363	5,71171E-65	8	
Ttc33	4,14271E-69	0,2838	0,536	0,311	6,95602E-65	8	
Med19	6,02807E-69	0,28894	0,544	0,319	1,01217E-64	8	
Rars	8,96984E-69	0,28993	0,543	0,316	1,50613E-64	8	
Phax	2,18478E-68	0,30118	0,632	0,401	3,66846E-64	8	
Csnk2b	2,20534E-68	0,3162	0,771	0,539	3,70298E-64	8	
Bri3	2,36241E-68	0,30066	0,714	0,462	3,96672E-64	8	
Nfu1	2,89686E-68	0,28522	0,573	0,342	4,86412E-64	8	
Fkbp1b	3,12615E-68	0,32691	0,645	0,419	5,24913E-64	8	
Mrpl36	3,26507E-68	0,28176	0,512	0,292	5,48237E-64	8	
Acadm	3,79242E-68	0,2722	0,527	0,302	6,36785E-64	8	
Bola1	4,1921E-68	0,2696	0,423	0,22	7,03896E-64	8	
Btg3	4,68621E-68	0,27907	0,472	0,259	7,86861E-64	8	
Lsm12	6,87557E-68	0,29788	0,631	0,402	1,15448E-63	8	
Psmb10	7,4218E-68	0,31455	0,842	0,64	1,24619E-63	8	
Smyd2	7,95039E-68	0,27892	0,525	0,301	1,33495E-63	8	
Trnt1	8,31557E-68	0,26644	0,432	0,226	1,39627E-63	8	
Pgp	9,76244E-68	0,32103	0,654	0,427	1,63921E-63	8	
Eif2b2	1,01992E-67	0,26492	0,404	0,205	1,71255E-63	8	
Yipf3	1,23473E-67	0,30172	0,592	0,367	2,07324E-63	8	
Psmd4	1,63639E-67	0,31106	0,907	0,752	2,74766E-63	8	
Msl3	1,69689E-67	0,25609	0,41	0,208	2,84925E-63	8	
Hist1h2bc	1,87512E-67	0,26083	0,347	0,165	3,14852E-63	8	
Psmg2	2,20581E-67	0,29002	0,555	0,329	3,70377E-63	8	
Taf13	2,28541E-67	0,26204	0,514	0,291	3,83743E-63	8	
Clu	2,41034E-67	0,426	0,591	0,378	4,0472E-63	8	
Eid2	2,53235E-67	0,28712	0,645	0,408	4,25207E-63	8	
Rsl1d1	4,04489E-67	0,29364	0,616	0,388	6,79177E-63	8	
Psenen	4,20722E-67	0,29798	0,717	0,476	7,06434E-63	8	
Sf3b6	4,94449E-67	0,31655	0,739	0,519	8,30229E-63	8	
Arpc2	4,9563E-67	0,32603	0,856	0,698	8,32213E-63	8	
Trappc5	8,84215E-67	0,2739	0,52	0,3	1,48469E-62	8	
Rbfa	1,14816E-66	0,27818	0,498	0,281	1,92788E-62	8	
Top1	1,62671E-66	0,31693	0,921	0,814	2,7314E-62	8	

1810043H04	2,10386E-66	0,28474	0,551	0,327	3,53259E-62	8	
Psma2	3,25989E-66	0,30046	0,925	0,802	5,47369E-62	8	
1810043G02	4,68447E-66	0,26562	0,444	0,24	7,8657E-62	8	
Pmpcb	4,705E-66	0,26652	0,446	0,241	7,90017E-62	8	
Snrpd1	4,73151E-66	0,30037	0,721	0,487	7,94467E-62	8	
Phb2	8,67529E-66	0,29248	0,615	0,391	1,45667E-61	8	
Ddost	1,0417E-65	0,29471	0,61	0,383	1,74912E-61	8	
E530001K10	1,06245E-65	0,31392	0,678	0,447	1,78396E-61	8	
Lsm7	2,21694E-65	0,25831	0,396	0,201	3,72246E-61	8	
Tubg1	2,67338E-65	0,26077	0,479	0,266	4,48888E-61	8	
Commd7	4,0933E-65	0,29396	0,563	0,341	6,87307E-61	8	
Mrpl24	4,1697E-65	0,27149	0,478	0,268	7,00135E-61	8	
Ufd1l	4,45644E-65	0,27215	0,515	0,298	7,48281E-61	8	
Timm50	4,95408E-65	0,26956	0,468	0,259	8,31839E-61	8	
C1qtnf4	5,06649E-65	0,25395	0,406	0,206	8,50714E-61	8	
Rala	9,43672E-65	0,30355	0,691	0,462	1,58452E-60	8	
Commd9	1,00825E-64	0,26238	0,553	0,325	1,69295E-60	8	
Gpi1	1,38622E-64	0,30056	0,957	0,882	2,3276E-60	8	
Dnajb2	1,63059E-64	0,3106	0,774	0,562	2,73793E-60	8	
2310033P09	2,5323E-64	0,25894	0,48	0,267	4,25199E-60	8	
Utp11l	2,69527E-64	0,26338	0,514	0,296	4,52563E-60	8	
Cd59a	2,74385E-64	0,328	0,553	0,339	4,6072E-60	8	
Ppt2	5,4978E-64	0,25255	0,378	0,19	9,23136E-60	8	
Slc3a2	9,07648E-64	0,3369	0,798	0,609	1,52403E-59	8	
Hypk	9,36666E-64	0,26835	0,534	0,315	1,57276E-59	8	
Faim	1,12301E-63	0,25451	0,474	0,264	1,88565E-59	8	
Abcg2	1,38372E-63	0,2519	0,4	0,206	2,3234E-59	8	
Sec13	1,65762E-63	0,27432	0,575	0,354	2,78331E-59	8	
0610009B22	1,76821E-63	0,25318	0,473	0,262	2,969E-59	8	
Cox14	2,30365E-63	0,30467	0,701	0,476	3,86806E-59	8	
Ifi35	3,72926E-63	0,30098	0,609	0,385	6,2618E-59	8	
Snrpf	4,60033E-63	0,3137	0,908	0,752	7,72442E-59	8	
Ppat	4,66748E-63	0,25801	0,484	0,273	7,83717E-59	8	
Exosc4	6,67723E-63	0,28036	0,556	0,338	1,12117E-58	8	
5033428I22F	7,14753E-63	0,3013	0,533	0,324	1,20014E-58	8	
Apex1	7,15888E-63	0,27536	0,483	0,274	1,20205E-58	8	
Gorasp2	9,55508E-63	0,26306	0,474	0,267	1,60439E-58	8	
Mrps17	1,06149E-62	0,28942	0,615	0,392	1,78234E-58	8	
Sbds	1,06861E-62	0,30114	0,693	0,473	1,7943E-58	8	
Nmi	1,96038E-62	0,25193	0,423	0,224	3,29168E-58	8	
Pde6d	2,45683E-62	0,29151	0,639	0,421	4,12527E-58	8	
Tmem167	2,93147E-62	0,27883	0,669	0,438	4,92223E-58	8	
Med29	2,93932E-62	0,27403	0,544	0,33	4,93542E-58	8	
Xpa	3,56491E-62	0,25866	0,452	0,249	5,98585E-58	8	
Tcp1	4,12183E-62	0,31575	0,798	0,603	6,92097E-58	8	
Mesdc2	5,14311E-62	0,30418	0,685	0,469	8,63579E-58	8	
Mcts1	7,4136E-62	0,26503	0,547	0,326	1,24482E-57	8	
Mageh1	8,13917E-62	0,29946	0,596	0,386	1,36665E-57	8	

9130401M01	9,92326E-62	0,25229	0,424	0,228	1,66621E-57	8	
Ifngr2	1,46886E-61	0,25539	0,456	0,253	2,46636E-57	8	
Ginm1	1,79515E-61	0,2725	0,492	0,288	3,01424E-57	8	
Tcea1	2,0258E-61	0,30325	0,716	0,499	3,40152E-57	8	
Sptssa	2,28855E-61	0,32998	0,757	0,558	3,8427E-57	8	
Prkcsh	2,67527E-61	0,26731	0,572	0,351	4,49205E-57	8	
Eif3d	2,68203E-61	0,28828	0,639	0,42	4,5034E-57	8	
Tpi1	3,48597E-61	0,26617	0,969	0,861	5,85329E-57	8	
Anapc16	4,7159E-61	0,25506	0,494	0,282	7,91846E-57	8	
Atg101	5,48436E-61	0,25373	0,463	0,26	9,20879E-57	8	
Pmm1	6,74479E-61	0,29936	0,787	0,567	1,13252E-56	8	
Cetn3	6,85626E-61	0,29239	0,737	0,507	1,15123E-56	8	
Pcbd2	7,29141E-61	0,26186	0,408	0,219	1,2243E-56	8	
Idh3g	1,17259E-60	0,29171	0,635	0,413	1,9689E-56	8	
Ttc1	2,49273E-60	0,27468	0,536	0,326	4,18554E-56	8	
Zcchc17	3,11748E-60	0,28712	0,705	0,484	5,23456E-56	8	
Gpatch11	3,20733E-60	0,25536	0,477	0,271	5,38543E-56	8	
Fzd3	3,64981E-60	0,28792	0,664	0,441	6,12839E-56	8	
Frg1	6,37623E-60	0,30648	0,806	0,602	1,07063E-55	8	
2610001J05R	6,50448E-60	0,26219	0,49	0,284	1,09217E-55	8	
Cdk4	9,57119E-60	0,25072	0,426	0,232	1,6071E-55	8	
Gm26735	1,18904E-59	0,27171	0,474	0,269	1,99652E-55	8	
Gtf2b	1,56165E-59	0,27559	0,589	0,374	2,62216E-55	8	
Ddx18	2,05346E-59	0,2641	0,521	0,314	3,44797E-55	8	
Cir1	2,89843E-59	0,30885	0,815	0,626	4,86675E-55	8	
Nradd	3,09995E-59	0,26085	0,454	0,255	5,20513E-55	8	
Suds3	3,45465E-59	0,27052	0,486	0,289	5,80071E-55	8	
Manf	3,71927E-59	0,31231	0,773	0,575	6,24502E-55	8	
Nudcd3	4,14114E-59	0,26651	0,983	0,932	6,95339E-55	8	
2410004B18	4,37235E-59	0,25736	0,458	0,261	7,34162E-55	8	
Samm50	6,32587E-59	0,25928	0,554	0,335	1,06218E-54	8	
Ppp1r11	7,47137E-59	0,30379	0,837	0,663	1,25452E-54	8	
Copb2	1,22938E-58	0,26751	0,65	0,424	2,06425E-54	8	
Them4	1,72066E-58	0,28821	0,674	0,46	2,88915E-54	8	
Gtf2a2	1,85924E-58	0,2719	0,545	0,339	3,12184E-54	8	
Mtx2	2,15584E-58	0,28617	0,628	0,416	3,61987E-54	8	
Etfb	3,63993E-58	0,26492	0,633	0,408	6,1118E-54	8	
Ncam2	4,71118E-58	0,30164	0,882	0,755	7,91055E-54	8	
Dnttip1	5,09673E-58	0,25277	0,451	0,256	8,55792E-54	8	
Dnajc12	6,00654E-58	0,25992	0,569	0,352	1,00856E-53	8	
Enho	6,67477E-58	0,26225	0,458	0,263	1,12076E-53	8	
Selm	8,50755E-58	0,27422	0,961	0,837	1,4285E-53	8	
Drg1	1,20732E-57	0,25568	0,543	0,334	2,02722E-53	8	
Atg3	1,32347E-57	0,25431	0,548	0,333	2,22224E-53	8	
Zfp639	1,62098E-57	0,25427	0,484	0,284	2,72179E-53	8	
2810004N23	2,4368E-57	0,28612	0,633	0,422	4,09163E-53	8	
Srek1ip1	2,67482E-57	0,25428	0,492	0,29	4,49129E-53	8	
Stard10	3,3529E-57	0,30562	0,86	0,71	5,62985E-53	8	

Hmox2	3,40214E-57	0,27997	0,709	0,489	5,71254E-53	8	
Eif4a3	6,84665E-57	0,25596	0,559	0,348	1,14962E-52	8	
Phf5a	8,33919E-57	0,26809	0,569	0,358	1,40023E-52	8	
Anxa5	1,565E-56	0,29829	0,835	0,673	2,62779E-52	8	
Tsc22d4	3,13048E-56	0,30102	0,683	0,472	5,25639E-52	8	
Rbm39	3,49388E-56	0,25936	0,988	0,963	5,86657E-52	8	
March5	3,92078E-56	0,25351	0,53	0,323	6,58339E-52	8	
Banf1	4,70935E-56	0,27308	0,645	0,431	7,90748E-52	8	
Cisd3	9,67868E-56	0,28221	0,574	0,371	1,62515E-51	8	
Ktn1	1,74696E-55	0,25999	0,978	0,937	2,93331E-51	8	
Ift22	2,3385E-55	0,2546	0,573	0,362	3,92658E-51	8	
Fst	2,61565E-55	0,28225	0,482	0,28	4,39194E-51	8	
Eny2	2,88091E-55	0,26915	0,616	0,405	4,83734E-51	8	
Yeats4	4,8338E-55	0,26858	0,654	0,446	8,11643E-51	8	
Gps1	6,52999E-55	0,28474	0,645	0,439	1,09645E-50	8	
Vat1	1,07413E-54	0,25311	0,986	0,923	1,80357E-50	8	
Smim19	3,04515E-54	0,25103	0,49	0,294	5,11311E-50	8	
Xrn2	3,61174E-54	0,29753	0,678	0,475	6,06447E-50	8	
Spcs2	5,87967E-54	0,28596	0,809	0,633	9,87256E-50	8	
Vamp7	6,7946E-54	0,27998	0,607	0,406	1,14088E-49	8	
Tmem55b	1,54895E-53	0,25586	0,636	0,425	2,60084E-49	8	
Ergic3	1,66517E-53	0,28452	0,792	0,599	2,79598E-49	8	
Higd2a	2,2385E-53	0,28667	0,756	0,57	3,75866E-49	8	
Cfl2	5,78818E-53	0,30237	0,823	0,678	9,71893E-49	8	
Letm1	7,23261E-53	0,27837	0,636	0,427	1,21443E-48	8	
Sod2	7,50561E-53	0,27963	0,71	0,508	1,26027E-48	8	
Gstm1	1,18775E-52	0,27529	0,605	0,397	1,99435E-48	8	
Tmem100	2,23887E-52	0,32727	0,778	0,594	3,75929E-48	8	
Nnat	3,17931E-52	0,66504	0,622	0,469	5,33837E-48	8	
Nsdhl	3,48047E-52	0,26201	0,568	0,368	5,84406E-48	8	
Cwf19l2	3,85248E-52	0,25876	0,57	0,367	6,4687E-48	8	
Paip2	4,25492E-52	0,25764	0,943	0,855	7,14443E-48	8	
Ubxn1	4,86932E-52	0,28118	0,841	0,673	8,17607E-48	8	
Hspd1	8,52736E-52	0,28491	0,787	0,607	1,43183E-47	8	
Tmem176a	1,0134E-51	0,27249	0,688	0,478	1,7016E-47	8	
Eif1a	1,33834E-51	0,27237	0,632	0,431	2,24721E-47	8	
Tpr	4,2536E-51	0,30743	0,842	0,699	7,14221E-47	8	
Arl6ip5	6,36731E-51	0,26647	0,652	0,449	1,06914E-46	8	
Lamp2	6,89205E-51	0,26283	0,682	0,479	1,15724E-46	8	
Rrp1	9,76633E-51	0,26136	0,92	0,793	1,63986E-46	8	
Gnl3l	1,10969E-50	0,27517	0,967	0,93	1,86328E-46	8	
Hprt	1,14666E-50	0,27953	0,763	0,575	1,92536E-46	8	
Lamtor1	1,19863E-50	0,28708	0,791	0,612	2,01262E-46	8	
Iscu	1,2067E-50	0,27095	0,809	0,63	2,02617E-46	8	
Eif1ax	1,45482E-50	0,28377	0,89	0,772	2,44279E-46	8	
Myl12b	2,13939E-50	0,27223	0,916	0,818	3,59226E-46	8	
Atp6v1d	3,87971E-50	0,27015	0,86	0,708	6,51441E-46	8	
Gdi2	8,31251E-50	0,27378	0,772	0,584	1,39575E-45	8	

Psmd13	1,6892E-49	0,26087	0,814	0,614	2,83634E-45	8	
Anapc11	3,14377E-49	0,26642	0,842	0,672	5,27871E-45	8	
Xbp1	9,84963E-49	0,27487	0,699	0,508	1,65385E-44	8	
Srp9	1,84151E-48	0,26891	0,793	0,613	3,09208E-44	8	
Ctxn1	2,16759E-48	0,29396	0,792	0,634	3,63961E-44	8	
Stard3nl	4,12176E-48	0,2798	0,76	0,586	6,92084E-44	8	
Olfm1	9,19823E-48	0,27268	0,861	0,732	1,54448E-43	8	
Sf3b2	4,04352E-47	0,25639	0,935	0,844	6,78947E-43	8	
Tceal5	5,20328E-47	0,28388	0,634	0,44	8,73682E-43	8	
Cnbp	5,7946E-47	0,27274	0,808	0,656	9,72971E-43	8	
Vbp1	7,08468E-47	0,26133	0,707	0,51	1,18959E-42	8	
Ubxn6	1,85843E-46	0,25756	0,882	0,739	3,12048E-42	8	
Mt3	9,79556E-46	0,41365	0,812	0,715	1,64477E-41	8	
Jkamp	1,25999E-45	0,25398	0,765	0,576	2,11565E-41	8	
Cebpz	3,27161E-45	0,25162	0,709	0,512	5,49336E-41	8	
Usp46	5,4767E-45	0,26092	0,649	0,458	9,19593E-41	8	
Rsrc2	3,46079E-44	0,26948	0,922	0,837	5,81102E-40	8	
Fermt2	1,39369E-42	0,28474	0,729	0,557	2,34015E-38	8	
Ube2n	2,63225E-42	0,25017	0,756	0,579	4,41981E-38	8	
Necab2	3,77292E-40	0,28381	0,605	0,432	6,33511E-36	8	
Id1	6,98832E-39	0,25918	0,612	0,433	1,17341E-34	8	
Htr3a	4,8304E-25	0,29267	0,735	0,653	8,11073E-21	8	
Cartpt	2,64854E-22	0,46817	0,297	0,196	4,44717E-18	8	
S100b	6,40201E-12	0,35164	0,796	0,849	1,07496E-07	8	
Paip2b	1,1631E-105	4,23111	1	0,495	1,953E-101	9	smENC3d
Igfbp7	1,11367E-42	0,90612	0,93	0,398	1,86997E-38	9	
Fxyd5	6,11273E-41	0,93278	0,951	0,442	1,02639E-36	9	
Vip	2,29966E-36	1,2238	1	0,931	3,86136E-32	9	
Tgfb1	5,45378E-36	0,69523	0,853	0,347	9,15744E-32	9	
Nsg1	1,74595E-34	0,71776	1	0,988	2,93163E-30	9	
Scgn	5,04593E-34	1,07923	1	0,877	8,47262E-30	9	
Cox7c	6,65426E-34	0,84002	1	0,959	1,11732E-29	9	
Cd9	1,57589E-32	0,84071	1	0,993	2,64607E-28	9	
Npy	7,42132E-32	1,06779	1	0,864	1,24611E-27	9	
Gfra1	1,03924E-31	0,80993	0,951	0,476	1,74499E-27	9	
Gapdh	1,54331E-30	0,86136	1	0,933	2,59138E-26	9	
Moxd1	1,67271E-30	0,82746	0,965	0,51	2,80865E-26	9	
Pcolce	1,79532E-30	0,64174	0,657	0,248	3,01452E-26	9	
Tuba1a	1,89965E-30	0,84409	1	0,993	3,1897E-26	9	
Cst3	7,22603E-30	0,75043	1	0,937	1,21332E-25	9	
Rps4x	1,73755E-29	0,81889	1	0,886	2,91752E-25	9	
Cidea	2,19761E-29	0,72308	0,986	0,789	3,69001E-25	9	
Csrp1	3,53332E-29	0,64677	1	0,97	5,9328E-25	9	
Id3	3,56974E-29	0,82804	0,881	0,463	5,99394E-25	9	
Nfe2l2	4,40954E-29	0,55566	0,713	0,284	7,40405E-25	9	
Sh3bgrl3	6,08928E-29	0,7632	0,944	0,63	1,02245E-24	9	
Bglap	4,46341E-28	0,7084	0,608	0,23	7,49452E-24	9	
Resp18	7,12191E-28	0,90318	1	0,934	1,19584E-23	9	

Sec61g	1,16754E-27	0,75431	1	0,872	1,96042E-23	9	
Thy1	1,20982E-27	0,81184	0,944	0,551	2,0314E-23	9	
Trappc1	3,17745E-27	0,59669	0,713	0,31	5,33526E-23	9	
Cox7a2l	4,40934E-27	0,74801	0,93	0,625	7,40372E-23	9	
Dstn	6,05947E-27	0,70006	1	0,951	1,01745E-22	9	
Calm2	6,09979E-27	0,49889	1	1	1,02422E-22	9	
Rpl14	7,0733E-27	0,57309	1	0,984	1,18768E-22	9	
Crip1	1,30146E-26	0,74523	1	0,988	2,18528E-22	9	
Cd24a	4,97149E-26	0,76979	1	0,64	8,34764E-22	9	
Eef1e1	5,77761E-26	0,65984	0,951	0,549	9,70118E-22	9	
Phlda3	6,83366E-26	0,73056	0,951	0,664	1,14744E-21	9	
Alcam	1,34602E-25	0,71661	0,979	0,63	2,26011E-21	9	
S100a4	2,26199E-25	0,93247	0,951	0,763	3,7981E-21	9	
Lgals1	2,97811E-25	0,76259	1	0,92	5,00054E-21	9	
Slc25a4	4,63454E-25	0,59576	1	0,981	7,78186E-21	9	
Fau	5,92474E-25	0,66625	1	0,924	9,94823E-21	9	
Sertm1	1,66438E-24	0,5083	0,629	0,249	2,79467E-20	9	
Cox4i1	1,69605E-24	0,70517	1	0,889	2,84783E-20	9	
Atpif1	1,82287E-24	0,71688	1	0,952	3,06078E-20	9	
Dbh	1,8877E-24	1,04418	0,944	0,686	3,16963E-20	9	
Gng5	2,03827E-24	0,62896	0,902	0,538	3,42247E-20	9	
Rps21	2,80499E-24	0,79931	1	0,952	4,70985E-20	9	
Rpl19	6,5848E-24	0,70094	1	0,933	1,10565E-19	9	
Spock3	7,79859E-24	0,65664	0,965	0,618	1,30946E-19	9	
Uqcrh	9,71372E-24	0,65665	0,972	0,771	1,63103E-19	9	
Rpl41	1,82348E-23	0,5127	1	0,998	3,06181E-19	9	
Psmb5	1,88555E-23	0,63071	0,979	0,781	3,16603E-19	9	
Atp6v0e	1,92028E-23	0,63276	0,895	0,551	3,22435E-19	9	
Rps3	1,92302E-23	0,68257	0,993	0,868	3,22894E-19	9	
Rps9	2,87207E-23	0,63943	1	0,919	4,8225E-19	9	
Hpca	3,96911E-23	0,61211	0,79	0,38	6,66454E-19	9	
Tmsb4x	4,14045E-23	0,50396	1	1	6,95224E-19	9	
Ndst4	9,07264E-23	0,51801	0,769	0,35	1,52339E-18	9	
Ldha	9,81563E-23	0,53549	1	0,975	1,64814E-18	9	
Rps12	1,00845E-22	0,63654	0,986	0,829	1,69328E-18	9	
Rps8	1,09497E-22	0,64335	1	0,978	1,83857E-18	9	
Rpl7	1,18885E-22	0,66701	0,965	0,767	1,99619E-18	9	
Cela1	1,20006E-22	0,36491	0,399	0,122	2,01502E-18	9	
Rps27	1,49883E-22	0,74448	0,986	0,868	2,51668E-18	9	
Prdx2	1,63836E-22	0,65533	0,979	0,829	2,75096E-18	9	
Ppa1	1,77549E-22	0,65406	0,902	0,588	2,98123E-18	9	
Ndufb9	2,18718E-22	0,71104	0,923	0,64	3,6725E-18	9	
Gm10076	2,55829E-22	0,71423	0,986	0,848	4,29562E-18	9	
Th	3,19691E-22	0,93349	0,685	0,321	5,36793E-18	9	
Cox7a2	3,30337E-22	0,61091	0,993	0,853	5,5467E-18	9	
Rpl35	3,45559E-22	0,69142	0,979	0,831	5,80227E-18	9	
Ttc9b	3,9677E-22	0,61965	0,902	0,588	6,66216E-18	9	
Atp5b	5,18006E-22	0,55707	1	0,922	8,69784E-18	9	

Rpl24	5,33251E-22	0,63081	1	0,947	8,95382E-18	9	
Rps24	5,79509E-22	0,64279	1	0,968	9,73053E-18	9	
Jtb	6,00644E-22	0,43913	0,594	0,246	1,00854E-17	9	
Ubl5	6,34431E-22	0,61378	0,972	0,775	1,06527E-17	9	
Rpl9	6,70585E-22	0,6851	0,993	0,819	1,12598E-17	9	
Cops6	7,67039E-22	0,59696	0,888	0,587	1,28794E-17	9	
Cox6b1	8,26828E-22	0,57694	1	0,873	1,38833E-17	9	
Fth1	8,30024E-22	0,59521	1	0,994	1,39369E-17	9	
Uqcr11	8,31291E-22	0,62824	0,979	0,83	1,39582E-17	9	
Chchd2	1,27435E-21	0,6272	0,993	0,921	2,13977E-17	9	
Hspa8	1,30876E-21	0,52245	1	0,995	2,19754E-17	9	
Cox5a	1,3505E-21	0,65104	0,923	0,634	2,26763E-17	9	
Rpl22l1	2,21358E-21	0,63079	0,972	0,78	3,71683E-17	9	
Carkd	2,34342E-21	0,47145	0,657	0,295	3,93483E-17	9	
Myeov2	2,61659E-21	0,66364	0,846	0,506	4,39352E-17	9	
Rps16	2,79586E-21	0,56101	1	0,924	4,69452E-17	9	
Ndufa7	3,13097E-21	0,63638	0,93	0,702	5,25721E-17	9	
Cox7b	3,45662E-21	0,55707	0,993	0,844	5,80401E-17	9	
Ubb	3,49865E-21	0,65496	1	0,987	5,87458E-17	9	
Pfdn5	3,66645E-21	0,61793	0,944	0,755	6,15634E-17	9	
Gm13889	3,88939E-21	0,53121	0,825	0,408	6,53068E-17	9	
Lamc3	3,94185E-21	0,53763	0,671	0,304	6,61875E-17	9	
Rpl3	4,05714E-21	0,6126	1	0,922	6,81234E-17	9	
Tomm7	4,27285E-21	0,60462	0,979	0,773	7,17454E-17	9	
Rplp2	4,47971E-21	0,56494	0,993	0,933	7,52188E-17	9	
Rps23	4,55594E-21	0,61213	1	0,905	7,64987E-17	9	
Atp5f1	5,21228E-21	0,62546	0,951	0,671	8,75194E-17	9	
Rpl37a	6,10474E-21	0,61802	1	0,988	1,02505E-16	9	
F2rl2	7,47943E-21	0,40028	0,545	0,212	1,25587E-16	9	
Acot7	8,01452E-21	0,57452	0,923	0,747	1,34572E-16	9	
Thsd7a	8,30304E-21	0,4754	0,643	0,27	1,39416E-16	9	
Ndufb10	1,05197E-20	0,62416	0,874	0,516	1,76637E-16	9	
Atp5j2	1,05965E-20	0,65479	0,979	0,795	1,77927E-16	9	
Tspan13	1,25511E-20	0,63845	0,965	0,762	2,10745E-16	9	
Cuedc2	1,25977E-20	0,56207	0,888	0,532	2,11528E-16	9	
Arpc3	1,27222E-20	0,57183	0,958	0,782	2,13618E-16	9	
Uchl1	1,32326E-20	0,38322	1	1	2,22189E-16	9	
Rpl11	1,353E-20	0,52996	1	0,955	2,27181E-16	9	
Ntsr1	1,35585E-20	0,50998	0,734	0,347	2,2766E-16	9	
Nme1	1,3775E-20	0,62543	0,916	0,668	2,31295E-16	9	
Rabac1	1,43558E-20	0,56605	0,986	0,902	2,41048E-16	9	
Gm1673	1,64699E-20	0,61514	1	0,776	2,76546E-16	9	
Atp5h	1,76645E-20	0,59737	0,993	0,874	2,96604E-16	9	
Rps2	1,84002E-20	0,60018	0,979	0,847	3,08957E-16	9	
Rps13	1,88121E-20	0,6359	0,972	0,821	3,15874E-16	9	
Ndufa1	1,9612E-20	0,63876	0,902	0,585	3,29306E-16	9	
Etv1	2,10779E-20	0,70379	1	0,627	3,53918E-16	9	
Agrp	2,68335E-20	0,35218	0,287	0,077	4,50561E-16	9	

Rpl34	2,73326E-20	0,61152	1	0,919	4,58941E-16	9	
Ascl1	4,14184E-20	0,41748	0,636	0,269	6,95456E-16	9	
Rps27a	4,15483E-20	0,62466	1	0,93	6,97638E-16	9	
Hint1	4,60027E-20	0,60912	0,986	0,884	7,72431E-16	9	
Abcb1a	5,05207E-20	0,34157	0,399	0,131	8,48294E-16	9	
Rps3a1	5,94389E-20	0,71531	0,972	0,833	9,98039E-16	9	
Cox6a1	6,69128E-20	0,58263	0,979	0,85	1,12353E-15	9	
Ddt	6,77189E-20	0,37649	0,503	0,193	1,13707E-15	9	
Gnas	8,45007E-20	0,34484	1	1	1,41885E-15	9	
Tubb4b	8,48028E-20	0,60238	0,965	0,788	1,42392E-15	9	
Atp5e	9,75669E-20	0,6015	0,986	0,845	1,63825E-15	9	
Ftl1	1,01334E-19	0,55439	1	0,93	1,7015E-15	9	
Dync1i2	1,0248E-19	0,46538	1	0,999	1,72074E-15	9	
Rpl10	1,18842E-19	0,56418	0,993	0,88	1,99548E-15	9	
Rpl35a	1,46722E-19	0,56736	1	0,925	2,46362E-15	9	
Tpt1	1,49512E-19	0,58933	1	0,957	2,51045E-15	9	
Romo1	1,61522E-19	0,53571	0,993	0,87	2,71211E-15	9	
Glrx5	1,70604E-19	0,53449	0,762	0,404	2,86462E-15	9	
Psma7	1,82628E-19	0,54114	1	0,891	3,06651E-15	9	
Cox6c	2,01038E-19	0,60187	0,993	0,873	3,37563E-15	9	
Basp1	2,22409E-19	0,46539	1	0,999	3,73448E-15	9	
Psme2	2,25671E-19	0,55261	0,909	0,56	3,78924E-15	9	
Rpl22	2,35112E-19	0,61307	0,895	0,673	3,94776E-15	9	
Lnx1	2,45337E-19	0,41492	0,517	0,21	4,11945E-15	9	
Rps10	2,66034E-19	0,57367	1	0,922	4,46698E-15	9	
Atp5l	2,76149E-19	0,52247	1	0,908	4,63682E-15	9	
Rpl17	3,41924E-19	0,56036	0,993	0,919	5,74125E-15	9	
Gng3	4,02374E-19	0,60848	0,979	0,815	6,75626E-15	9	
H2afj	4,53854E-19	0,54527	0,755	0,42	7,62067E-15	9	
Tmem256	4,81721E-19	0,60188	0,832	0,515	8,08858E-15	9	
Ssr4	4,84609E-19	0,5608	0,895	0,563	8,13707E-15	9	
Ndufb11	4,93259E-19	0,55608	0,916	0,611	8,28232E-15	9	
Ncoa7	5,20475E-19	0,62984	0,958	0,796	8,73929E-15	9	
Rpl39	6,00702E-19	0,66498	0,993	0,937	1,00864E-14	9	
Mpc1	6,02782E-19	0,64442	0,965	0,834	1,01213E-14	9	
Tmem160	6,69692E-19	0,53117	0,818	0,477	1,12448E-14	9	
Mtch1	6,88193E-19	0,48885	1	0,969	1,15555E-14	9	
Pnp	9,45834E-19	0,44341	0,629	0,3	1,58815E-14	9	
Rps20	9,66999E-19	0,58276	0,986	0,901	1,62369E-14	9	
Sh3bgrl	1,06484E-18	0,54288	0,86	0,522	1,78797E-14	9	
Ndufb2	1,19911E-18	0,54184	0,944	0,667	2,01343E-14	9	
Rps7	1,28776E-18	0,63262	0,958	0,788	2,16228E-14	9	
Rps5	1,37158E-18	0,59227	0,993	0,887	2,30302E-14	9	
Lst1	1,41133E-18	0,52769	0,832	0,485	2,36977E-14	9	
A730017C20	1,57975E-18	0,63742	0,937	0,716	2,65256E-14	9	
Eef1a1	1,63674E-18	0,44951	1	0,999	2,74824E-14	9	
Rpl36a	1,85082E-18	0,64491	0,93	0,765	3,10771E-14	9	
Spint1	1,90985E-18	0,38937	0,392	0,137	3,20684E-14	9	

Wls	2,44918E-18	0,51819	0,734	0,391	4,11242E-14	9	
Tmem130	2,57581E-18	0,56222	0,888	0,573	4,32504E-14	9	
Ndfip1	2,75621E-18	0,5206	0,881	0,523	4,62794E-14	9	
Ppp1ca	3,1193E-18	0,51248	0,979	0,85	5,23762E-14	9	
Ccdc109b	3,13971E-18	0,39528	0,406	0,146	5,27188E-14	9	
Rps19	3,18989E-18	0,48327	1	0,948	5,35615E-14	9	
Kcnd2	3,33339E-18	0,44449	0,797	0,409	5,59709E-14	9	
Cox8a	3,50843E-18	0,49892	1	0,932	5,891E-14	9	
Gstm5	3,92586E-18	0,53799	0,846	0,487	6,59191E-14	9	
Tmem258	4,07018E-18	0,50749	0,86	0,493	6,83423E-14	9	
Isoc1	5,09222E-18	0,49722	0,748	0,397	8,55035E-14	9	
Rps28	5,77115E-18	0,62126	0,965	0,826	9,69033E-14	9	
Ninj1	8,12623E-18	0,4217	0,545	0,239	1,36448E-13	9	
Rab3a	1,03608E-17	0,50632	0,986	0,898	1,73969E-13	9	
Rpl27a	1,33082E-17	0,48	1	0,965	2,23457E-13	9	
Ndufa4	1,64468E-17	0,5973	0,958	0,842	2,76159E-13	9	
Qpct	1,72191E-17	0,39904	0,434	0,168	2,89126E-13	9	
Ndufa3	1,79195E-17	0,53817	0,972	0,774	3,00887E-13	9	
Atp5o	1,99029E-17	0,51275	0,965	0,808	3,3419E-13	9	
Cntnap5a	2,03759E-17	0,51989	0,986	0,681	3,42132E-13	9	
Clec14a	2,0769E-17	0,43973	0,615	0,284	3,48733E-13	9	
S100a6	2,21859E-17	0,51036	1	0,999	3,72524E-13	9	
Slc25a3	2,35351E-17	0,47528	0,993	0,961	3,95178E-13	9	
Stmn3	2,69733E-17	0,56712	0,986	0,932	4,52909E-13	9	
Rplp0	2,82913E-17	0,55194	0,972	0,778	4,75039E-13	9	
Eif3h	3,24147E-17	0,49493	0,86	0,537	5,44275E-13	9	
Atp5k	3,54254E-17	0,55563	1	0,959	5,94828E-13	9	
Mpc2	3,61867E-17	0,48105	0,86	0,49	6,07611E-13	9	
Uqcrq	3,67501E-17	0,48678	1	0,863	6,17071E-13	9	
Pam16	4,59538E-17	0,47108	0,755	0,422	7,71611E-13	9	
Rpl13	5,2981E-17	0,51277	1	0,957	8,89604E-13	9	
Hspa2	5,54507E-17	0,38268	0,573	0,262	9,31072E-13	9	
2010107E04	5,67379E-17	0,57954	0,909	0,659	9,52686E-13	9	
Atp5d	5,704E-17	0,47644	0,979	0,861	9,57759E-13	9	
Rps29	6,32151E-17	0,59119	1	0,997	1,06144E-12	9	
Ndufc1	6,49162E-17	0,53324	0,965	0,819	1,09001E-12	9	
Rpl30	7,03487E-17	0,61275	0,951	0,816	1,18122E-12	9	
Rpl38	7,53993E-17	0,54523	1	0,994	1,26603E-12	9	
Rpl12	8,57426E-17	0,53321	0,972	0,809	1,4397E-12	9	
Marc2	8,58859E-17	0,36719	0,552	0,249	1,44211E-12	9	
Rpl4	8,61401E-17	0,47666	0,986	0,852	1,44638E-12	9	
Tm4sf4	8,62998E-17	0,62943	0,678	0,351	1,44906E-12	9	
Psmd6	8,64298E-17	0,4737	0,867	0,531	1,45124E-12	9	
Gabarapl2	9,82885E-17	0,50687	0,993	0,873	1,65036E-12	9	
Rps18	1,01282E-16	0,49559	1	0,937	1,70063E-12	9	
Rexo2	1,07265E-16	0,53709	0,881	0,614	1,80109E-12	9	
Mrpl16	1,07806E-16	0,44911	0,657	0,334	1,81018E-12	9	
Atp5c1	1,18104E-16	0,5335	0,888	0,607	1,98308E-12	9	

Naca	1,25413E-16	0,49146	0,972	0,877	2,10581E-12	9	
Rpl18	1,27713E-16	0,52271	0,993	0,869	2,14444E-12	9	
Rpl6	1,4239E-16	0,55976	1	0,973	2,39086E-12	9	
Bex2	1,82911E-16	0,41371	1	0,999	3,07126E-12	9	
Rps11	1,93015E-16	0,47739	1	0,938	3,24091E-12	9	
Tbcc	2,1464E-16	0,407	0,608	0,303	3,60402E-12	9	
Ckb	2,28641E-16	0,57653	0,923	0,736	3,83912E-12	9	
Aimp1	2,75781E-16	0,49796	0,923	0,675	4,63064E-12	9	
Cox5b	2,76237E-16	0,50065	0,972	0,806	4,63829E-12	9	
Chchd1	2,78988E-16	0,41993	0,664	0,34	4,68449E-12	9	
Bambi	3,59647E-16	0,34672	0,441	0,175	6,03884E-12	9	
Mien1	3,65042E-16	0,47601	0,755	0,431	6,12942E-12	9	
Chga	3,89394E-16	0,50953	0,916	0,676	6,53831E-12	9	
Prdx4	3,98631E-16	0,42762	0,517	0,231	6,69341E-12	9	
Edf1	4,0458E-16	0,48975	0,958	0,706	6,7933E-12	9	
Ngfr	4,82533E-16	0,48765	0,951	0,572	8,10221E-12	9	
Tmed3	4,89365E-16	0,4551	0,692	0,375	8,21693E-12	9	
Rpl7a	4,9662E-16	0,50085	0,986	0,827	8,33874E-12	9	
Nrsn2	5,00342E-16	0,40304	0,538	0,237	8,40125E-12	9	
Polr1d	5,48191E-16	0,47595	0,825	0,512	9,20468E-12	9	
Atp5g2	6,22071E-16	0,49374	0,993	0,847	1,04452E-11	9	
Rpl37	6,35403E-16	0,51616	1	0,987	1,0669E-11	9	
Tbcb	6,89789E-16	0,46963	0,951	0,713	1,15822E-11	9	
Fbn1	6,97428E-16	0,44054	0,65	0,322	1,17105E-11	9	
Rps6	6,9933E-16	0,50627	0,979	0,846	1,17425E-11	9	
Psmb1	8,38853E-16	0,46442	0,979	0,831	1,40852E-11	9	
Qdpr	8,48782E-16	0,47071	0,853	0,559	1,42519E-11	9	
Ndufa5	8,61085E-16	0,47116	0,993	0,808	1,44585E-11	9	
Uba52	9,19917E-16	0,60285	0,776	0,467	1,54463E-11	9	
Bola2	9,28514E-16	0,49612	0,93	0,64	1,55907E-11	9	
Glrx3	9,55736E-16	0,52217	0,776	0,509	1,60478E-11	9	
Tmem108	1,00398E-15	0,34589	0,65	0,306	1,68578E-11	9	
Ndufb8	1,01403E-15	0,4938	0,888	0,583	1,70266E-11	9	
Scg5	1,01725E-15	0,54796	0,951	0,845	1,70806E-11	9	
Enpp1	1,10118E-15	0,40963	0,671	0,331	1,84899E-11	9	
Tceal6	1,30677E-15	0,44201	0,566	0,275	2,19419E-11	9	
Dctn2	1,31173E-15	0,50665	0,923	0,725	2,20252E-11	9	
1110065P20	1,44411E-15	0,45389	0,699	0,401	2,42481E-11	9	
Lxn	1,4964E-15	0,3553	0,524	0,236	2,51261E-11	9	
Cox17	1,65733E-15	0,47523	0,846	0,523	2,78283E-11	9	
Fibcd1	1,66286E-15	0,43653	0,804	0,418	2,7921E-11	9	
1500009L16I	1,67084E-15	0,50813	0,79	0,51	2,80551E-11	9	
Chst8	1,71606E-15	0,38093	0,476	0,208	2,88143E-11	9	
Ndufb4	1,76615E-15	0,47327	0,979	0,778	2,96555E-11	9	
Rpl26	1,92201E-15	0,45009	1	0,953	3,22724E-11	9	
Ndufs5	1,93202E-15	0,47986	0,979	0,857	3,24405E-11	9	
Socs2	2,0336E-15	0,5152	0,692	0,378	3,41463E-11	9	
1110004F10	2,04979E-15	0,44621	0,993	0,932	3,4418E-11	9	

Rps15a	2,05423E-15	0,48558	0,986	0,887	3,44926E-11	9	
Trappc4	2,22584E-15	0,51879	0,818	0,507	3,73742E-11	9	
Ndufs4	2,55274E-15	0,44176	0,881	0,558	4,28631E-11	9	
Entpd3	2,67575E-15	0,40499	0,769	0,416	4,49285E-11	9	
Fdps	2,91675E-15	0,5398	0,937	0,762	4,89751E-11	9	
F2r	2,94979E-15	0,36484	0,923	0,591	4,953E-11	9	
Sec61b	3,15062E-15	0,5191	0,888	0,612	5,2902E-11	9	
Taco1os	3,93337E-15	0,33701	0,483	0,208	6,60452E-11	9	
Ict1	4,21028E-15	0,3505	0,706	0,371	7,06948E-11	9	
Pcsk1	4,4217E-15	0,52738	0,881	0,65	7,42448E-11	9	
Rps17	4,75151E-15	0,43847	1	0,913	7,97827E-11	9	
Usmg5	5,08572E-15	0,44391	0,979	0,856	8,53943E-11	9	
Prokr1	5,63943E-15	0,26819	0,545	0,234	9,46916E-11	9	
Gm28905	5,79202E-15	0,31078	0,406	0,159	9,72539E-11	9	
Ywhaq	5,94459E-15	0,40559	0,993	0,953	9,98155E-11	9	
Rpl10a	6,12329E-15	0,48242	0,979	0,864	1,02816E-10	9	
Atp6v0d1	6,36205E-15	0,48119	0,937	0,791	1,06825E-10	9	
Gnb2l1	6,49728E-15	0,45982	0,986	0,806	1,09096E-10	9	
Lamtor2	6,57829E-15	0,46993	0,832	0,523	1,10456E-10	9	
Nenf	6,61017E-15	0,49716	0,979	0,776	1,10991E-10	9	
Gal	7,04963E-15	0,6981	0,51	0,233	1,1837E-10	9	
Mrpl20	7,31735E-15	0,44077	0,804	0,459	1,22866E-10	9	
Cd1d1	7,5016E-15	0,31941	0,385	0,149	1,25959E-10	9	
Palmd	7,51424E-15	0,51522	0,727	0,436	1,26172E-10	9	
Fam213a	7,78376E-15	0,32646	0,385	0,151	1,30697E-10	9	
Camk4	7,90737E-15	0,55881	0,839	0,591	1,32773E-10	9	
Rpl32	8,16814E-15	0,43756	1	0,967	1,37151E-10	9	
Idnk	8,48593E-15	0,4148	0,678	0,367	1,42487E-10	9	
Ndufv3	8,96064E-15	0,43934	0,979	0,862	1,50458E-10	9	
Pfdn1	9,09177E-15	0,46997	0,895	0,658	1,5266E-10	9	
Dtd1	9,68241E-15	0,40584	0,608	0,318	1,62577E-10	9	
Sv2b	9,83727E-15	0,30768	0,559	0,251	1,65178E-10	9	
Trpm2	1,09681E-14	0,37851	0,497	0,228	1,84165E-10	9	
Ndufa6	1,11782E-14	0,4534	0,916	0,639	1,87692E-10	9	
Atp5g3	1,21334E-14	0,47004	0,958	0,8	2,03731E-10	9	
Coa3	1,21543E-14	0,42632	0,853	0,531	2,04083E-10	9	
Akr1a1	1,55672E-14	0,41273	0,986	0,907	2,61388E-10	9	
Psmd10	1,58998E-14	0,34948	0,441	0,187	2,66973E-10	9	
Eef1b2	1,60873E-14	0,49105	0,965	0,818	2,70122E-10	9	
Nabp2	1,80796E-14	0,4037	0,657	0,346	3,03574E-10	9	
Rpl36al	2,65466E-14	0,45507	0,965	0,841	4,45745E-10	9	
Dach1	2,73668E-14	0,26575	0,42	0,167	4,59516E-10	9	
Uqcr10	2,80686E-14	0,47006	0,951	0,743	4,71301E-10	9	
Smdt1	3,31735E-14	0,41041	0,993	0,897	5,57016E-10	9	
Kcnt2	3,35912E-14	0,37141	0,671	0,339	5,64029E-10	9	
Rps25	3,48949E-14	0,42696	0,986	0,858	5,85921E-10	9	
Ucp2	3,51332E-14	0,47145	0,776	0,468	5,89921E-10	9	
Ptpre	3,57352E-14	0,35267	0,545	0,258	6,0003E-10	9	

Med30	3,69292E-14	0,31959	0,476	0,21	6,20078E-10	9	
Oaz1	3,9188E-14	0,45625	0,979	0,93	6,58005E-10	9	
Cnksr2	4,44532E-14	0,28644	0,35	0,132	7,46414E-10	9	
Bsg	4,47877E-14	0,44971	0,993	0,939	7,5203E-10	9	
Adh5	5,13385E-14	0,36429	0,692	0,387	8,62025E-10	9	
Smpd3	6,31153E-14	0,35413	1	0,999	1,05977E-09	9	
Acyp2	6,46078E-14	0,44178	0,86	0,558	1,08483E-09	9	
Serpinb6a	7,3031E-14	0,47987	0,958	0,848	1,22626E-09	9	
Cd63	7,41407E-14	0,47912	0,657	0,378	1,2449E-09	9	
Uqcrb	7,974E-14	0,46396	0,958	0,8	1,33891E-09	9	
Emc10	8,02092E-14	0,46128	0,93	0,776	1,34679E-09	9	
Cgref1	9,75373E-14	0,31819	0,455	0,2	1,63775E-09	9	
Pcbd1	9,83835E-14	0,44931	0,811	0,544	1,65196E-09	9	
Rps26	1,00581E-13	0,40801	1	0,92	1,68885E-09	9	
Dctn3	1,08882E-13	0,42995	0,944	0,727	1,82823E-09	9	
Eif2s2	1,09502E-13	0,43699	0,993	0,914	1,83866E-09	9	
Hsd17b10	1,13763E-13	0,37118	0,65	0,346	1,9102E-09	9	
Cd151	1,18915E-13	0,48265	0,874	0,646	1,9967E-09	9	
Tes	1,21164E-13	0,40341	0,601	0,308	2,03446E-09	9	
Dynll1	1,26141E-13	0,39868	1	0,989	2,11804E-09	9	
2610524H06	1,28287E-13	0,38702	0,601	0,322	2,15407E-09	9	
Apoa1bp	1,36253E-13	0,31993	0,573	0,28	2,28782E-09	9	
Tesc	1,69343E-13	0,26084	0,259	0,084	2,84344E-09	9	
Dpm3	1,80596E-13	0,49651	0,846	0,612	3,03239E-09	9	
Slirp	1,94868E-13	0,41573	0,741	0,439	3,27202E-09	9	
Ngfrap1	2,01567E-13	0,42052	0,993	0,949	3,38451E-09	9	
Sdhb	2,08785E-13	0,41683	0,874	0,578	3,50571E-09	9	
Ndufb7	2,13275E-13	0,42303	0,951	0,78	3,58111E-09	9	
Tonsl	2,33111E-13	0,41061	0,888	0,612	3,91416E-09	9	
Tmem14c	2,43227E-13	0,36975	0,629	0,336	4,08402E-09	9	
Ptprz1	2,54322E-13	0,38387	0,622	0,313	4,27033E-09	9	
Rimbp2	2,65811E-13	0,33492	0,608	0,303	4,46323E-09	9	
Dhrs7	2,95336E-13	0,38822	0,713	0,419	4,95899E-09	9	
Cfap36	3,01258E-13	0,45176	0,832	0,569	5,05842E-09	9	
Ndufa11	3,05988E-13	0,3868	0,993	0,89	5,13784E-09	9	
Itga1	3,09118E-13	0,2722	0,273	0,093	5,1904E-09	9	
Fhod3	3,15852E-13	0,38723	0,958	0,907	5,30347E-09	9	
Sertad1	3,19774E-13	0,28879	0,406	0,171	5,36932E-09	9	
Cyb561d2	3,19971E-13	0,32286	0,483	0,225	5,37263E-09	9	
Cpne5	3,27692E-13	0,36696	0,503	0,246	5,50228E-09	9	
Rpl5	3,97661E-13	0,39211	0,972	0,862	6,67713E-09	9	
Rpl29	4,09039E-13	0,51993	0,86	0,617	6,86817E-09	9	
Cox20	4,12021E-13	0,39381	0,559	0,292	6,91824E-09	9	
Ndufb5	4,89099E-13	0,43767	0,769	0,495	8,21246E-09	9	
Pop5	4,91271E-13	0,36595	0,713	0,412	8,24893E-09	9	
Hsp90aa1	5,81937E-13	0,36357	1	0,99	9,7713E-09	9	
Gadd45g	5,96571E-13	0,42741	0,545	0,276	1,0017E-08	9	
Tmsb10	6,0545E-13	0,43466	1	0,954	1,01661E-08	9	

1810058I24R	6,12235E-13	0,3699	0,699	0,403	1,028E-08	9	
Gpr149	6,3528E-13	0,35232	0,713	0,381	1,0667E-08	9	
Rpl36	6,48874E-13	0,39019	1	0,947	1,08952E-08	9	
Ufc1	6,76213E-13	0,35231	0,762	0,448	1,13543E-08	9	
Ubc	6,98995E-13	0,36214	1	0,98	1,17368E-08	9	
Btf3	7,70818E-13	0,51316	0,888	0,756	1,29428E-08	9	
Bglap2	8,06726E-13	0,3479	0,259	0,088	1,35457E-08	9	
Slc39a7	8,73622E-13	0,35591	0,58	0,308	1,4669E-08	9	
Mrps34	8,78499E-13	0,36695	0,573	0,305	1,47509E-08	9	
Ndufa13	9,10928E-13	0,41602	1	0,807	1,52954E-08	9	
Cetn2	9,45424E-13	0,45144	0,895	0,701	1,58746E-08	9	
Ndufa12	9,57385E-13	0,44427	0,86	0,585	1,60754E-08	9	
A830010M2(	1,02894E-12	0,38959	0,72	0,422	1,72769E-08	9	
Rbm3	1,04609E-12	0,44677	0,937	0,793	1,75649E-08	9	
Tubb2a	1,06573E-12	0,36869	1	0,995	1,78947E-08	9	
Myl1	1,11191E-12	0,40986	0,993	0,938	1,867E-08	9	
Mrpl51	1,27419E-12	0,40459	0,895	0,61	2,13949E-08	9	
Pgls	1,28776E-12	0,42467	0,846	0,563	2,16227E-08	9	
Cdh11	1,38385E-12	0,27376	0,448	0,198	2,32363E-08	9	
Tmem255b	1,4398E-12	0,41673	0,909	0,763	2,41756E-08	9	
Tmem50a	1,47767E-12	0,39702	0,986	0,911	2,48116E-08	9	
B3glct	1,5127E-12	0,34742	0,587	0,304	2,53998E-08	9	
Cenpf	1,58374E-12	0,25311	0,406	0,174	2,65926E-08	9	
Trappc2l	1,65596E-12	0,41554	0,734	0,446	2,78052E-08	9	
Mrpl57	1,69049E-12	0,39689	0,706	0,447	2,8385E-08	9	
Nelfe	1,7079E-12	0,33922	0,601	0,321	2,86774E-08	9	
Ndufv2	1,71275E-12	0,41594	0,853	0,599	2,87587E-08	9	
Kcnq5	1,72685E-12	0,28092	0,434	0,19	2,89956E-08	9	
Pdcd2	1,73152E-12	0,25831	0,385	0,161	2,9074E-08	9	
Mdh2	1,76255E-12	0,44508	0,923	0,724	2,9595E-08	9	
Ywhae	1,84159E-12	0,36475	1	0,996	3,09221E-08	9	
Dnajb11	1,94758E-12	0,35233	0,629	0,349	3,27019E-08	9	
Ddrgk1	1,99216E-12	0,43955	0,706	0,43	3,34503E-08	9	
Tmbim4	1,99226E-12	0,39217	0,769	0,48	3,3452E-08	9	
Pin4	2,00344E-12	0,40619	0,748	0,461	3,36398E-08	9	
1810022K09	2,08791E-12	0,42426	0,839	0,59	3,50581E-08	9	
Gm561	2,09359E-12	0,3159	0,608	0,323	3,51534E-08	9	
Mrpl17	2,20507E-12	0,36131	0,664	0,372	3,70253E-08	9	
Hagh	2,23766E-12	0,41875	0,762	0,471	3,75725E-08	9	
Ndufa2	2,31759E-12	0,40181	0,986	0,887	3,89147E-08	9	
Fam89a	2,4912E-12	0,39333	0,51	0,26	4,18297E-08	9	
Ndufaf2	2,50551E-12	0,39258	0,678	0,387	4,207E-08	9	
Timm13	2,60419E-12	0,43376	0,86	0,596	4,37269E-08	9	
Acsl5	2,7504E-12	0,35703	0,608	0,337	4,6182E-08	9	
2700029M09	2,88619E-12	0,3687	0,552	0,29	4,8462E-08	9	
Aprt	3,27547E-12	0,41947	0,825	0,554	5,49985E-08	9	
Cit	3,32301E-12	0,29527	0,434	0,197	5,57967E-08	9	
Nol3	3,42346E-12	0,35525	0,608	0,33	5,74833E-08	9	

Crabp1	3,61399E-12	0,75502	0,727	0,495	6,06825E-08	9	
Sec62	3,66699E-12	0,38674	1	0,941	6,15724E-08	9	
Pard6a	3,81407E-12	0,35979	0,476	0,238	6,40421E-08	9	
Swi5	4,29333E-12	0,3678	0,965	0,807	7,20894E-08	9	
Rpl23	4,41333E-12	0,34688	1	0,992	7,41042E-08	9	
Psma4	4,45838E-12	0,41492	0,951	0,773	7,48607E-08	9	
Use1	4,62409E-12	0,37363	0,727	0,446	7,76431E-08	9	
Hint2	4,62652E-12	0,35455	0,622	0,352	7,7684E-08	9	
Ndufb6	4,77142E-12	0,40773	0,832	0,54	8,01168E-08	9	
Hddc2	4,80123E-12	0,29302	0,51	0,247	8,06175E-08	9	
Tmod1	4,90458E-12	0,4848	0,867	0,648	8,23529E-08	9	
Maged2	5,13841E-12	0,40065	0,65	0,373	8,62791E-08	9	
Dad1	5,32661E-12	0,40016	0,965	0,827	8,94391E-08	9	
Asl	5,39258E-12	0,40291	0,951	0,773	9,05468E-08	9	
Thoc7	5,4009E-12	0,46111	0,846	0,612	9,06864E-08	9	
Kif5c	5,60166E-12	0,32796	1	0,96	9,40574E-08	9	
Atp6v1f	5,87805E-12	0,38186	0,958	0,814	9,86983E-08	9	
Rps14	6,00899E-12	0,36898	0,993	0,955	1,00897E-07	9	
Myl6	6,13861E-12	0,3648	1	0,951	1,03073E-07	9	
Prkag1	6,20751E-12	0,3565	0,503	0,266	1,0423E-07	9	
Mid1ip1	6,42146E-12	0,39286	0,839	0,589	1,07823E-07	9	
Cyc1	6,45637E-12	0,3737	0,748	0,46	1,08409E-07	9	
Eif3m	6,47129E-12	0,36526	0,797	0,488	1,08659E-07	9	
Eif3l	6,59866E-12	0,38663	0,706	0,421	1,10798E-07	9	
Ube2m	6,63773E-12	0,42186	0,916	0,709	1,11454E-07	9	
Tmem159	6,64119E-12	0,26586	0,483	0,227	1,11512E-07	9	
Atp6v1e1	6,69359E-12	0,37096	1	0,96	1,12392E-07	9	
Ier3	6,81084E-12	0,34761	0,58	0,306	1,14361E-07	9	
Chchd7	6,82879E-12	0,31025	0,469	0,225	1,14662E-07	9	
2410015M2(	7,68518E-12	0,44495	0,811	0,555	1,29042E-07	9	
Smim4	8,05774E-12	0,30739	0,469	0,223	1,35298E-07	9	
Mgst3	8,54726E-12	0,34303	0,629	0,356	1,43517E-07	9	
Rps15	8,74309E-12	0,36289	0,993	0,946	1,46805E-07	9	
D8Ertd738e	9,06903E-12	0,4387	0,818	0,563	1,52278E-07	9	
Ebp	9,18228E-12	0,39495	0,664	0,395	1,5418E-07	9	
Pbxip1	9,27186E-12	0,27699	0,455	0,21	1,55684E-07	9	
Ufsp2	9,42132E-12	0,36747	0,636	0,364	1,58193E-07	9	
Srp14	9,68943E-12	0,41239	0,937	0,82	1,62695E-07	9	
Etl4	9,79268E-12	0,26198	0,364	0,153	1,64429E-07	9	
Eif3j1	9,82168E-12	0,42305	0,951	0,747	1,64916E-07	9	
Ap3s1	9,98711E-12	0,41357	0,867	0,618	1,67694E-07	9	
Taf13	1,11999E-11	0,33155	0,601	0,335	1,88057E-07	9	
Zbtb8os	1,13661E-11	0,27591	0,378	0,164	1,90848E-07	9	
Snx6	1,19755E-11	0,40094	0,699	0,409	2,01081E-07	9	
Slco3a1	1,21057E-11	0,31313	0,566	0,297	2,03266E-07	9	
Park7	1,21546E-11	0,36829	0,951	0,814	2,04087E-07	9	
Timm17a	1,24074E-11	0,41656	0,769	0,499	2,08333E-07	9	
Lsm2	1,27573E-11	0,29103	0,427	0,197	2,14208E-07	9	

Nxn	1,2788E-11	0,2645	0,371	0,158	2,14723E-07	9	
Morn2	1,28877E-11	0,32858	0,559	0,302	2,16397E-07	9	
Bloc1s5	1,32462E-11	0,31397	0,559	0,296	2,22417E-07	9	
Mrps18c	1,34125E-11	0,32527	0,699	0,399	2,25209E-07	9	
Zmat2	1,34966E-11	0,43011	0,853	0,631	2,26622E-07	9	
Abcg2	1,43661E-11	0,32046	0,49	0,244	2,41222E-07	9	
Manf	1,4521E-11	0,39272	0,86	0,615	2,43821E-07	9	
Rplp1	1,47795E-11	0,35395	1	0,979	2,48162E-07	9	
Dpcd	1,56296E-11	0,308	0,636	0,349	2,62437E-07	9	
Gabrg2	1,73301E-11	0,28453	0,371	0,161	2,90989E-07	9	
Fam162a	1,86266E-11	0,38654	0,678	0,41	3,12759E-07	9	
Tomm5	1,94959E-11	0,32008	0,699	0,399	3,27356E-07	9	
Rit2	1,95826E-11	0,37575	0,853	0,588	3,28812E-07	9	
Ppib	2,06898E-11	0,40156	0,853	0,603	3,47403E-07	9	
Myo16	2,09203E-11	0,27676	0,315	0,126	3,51272E-07	9	
Inafm1	2,09662E-11	0,32815	0,636	0,362	3,52043E-07	9	
0610012G03	2,10428E-11	0,39343	0,734	0,463	3,5333E-07	9	
Cct4	2,34416E-11	0,37916	0,755	0,476	3,93607E-07	9	
Chl1	2,39441E-11	0,37795	0,965	0,778	4,02046E-07	9	
Bmyc	2,5013E-11	0,40126	0,776	0,498	4,19993E-07	9	
Pfn2	2,6059E-11	0,38675	0,951	0,871	4,37557E-07	9	
Sub1	2,64952E-11	0,32561	0,979	0,955	4,44881E-07	9	
Gm10073	2,6553E-11	0,3136	0,594	0,314	4,45852E-07	9	
Comtd1	2,82415E-11	0,25787	0,357	0,152	4,74203E-07	9	
Dnajc15	2,87137E-11	0,32307	0,552	0,292	4,82132E-07	9	
Borcs8	2,9202E-11	0,30257	0,483	0,241	4,90331E-07	9	
Sugt1	2,98471E-11	0,39404	0,797	0,514	5,01162E-07	9	
Scfd1	3,01843E-11	0,30663	0,601	0,336	5,06824E-07	9	
Creb3	3,34751E-11	0,30586	0,573	0,315	5,6208E-07	9	
Cope	3,48026E-11	0,39832	0,853	0,599	5,84371E-07	9	
Mrpl23	3,64397E-11	0,36886	0,706	0,425	6,11858E-07	9	
Rap2b	3,6465E-11	0,26565	0,413	0,19	6,12283E-07	9	
Nars	3,65063E-11	0,38648	0,944	0,807	6,12978E-07	9	
Rer1	4,12615E-11	0,34679	0,797	0,5	6,92822E-07	9	
Triap1	4,13819E-11	0,29917	0,517	0,268	6,94843E-07	9	
Srp19	4,15385E-11	0,40156	0,818	0,587	6,97472E-07	9	
Mrpl54	4,3348E-11	0,34533	0,797	0,529	7,27856E-07	9	
Dlk1	4,39454E-11	0,3316	0,35	0,148	7,37888E-07	9	
1110008F13	4,42618E-11	0,33688	0,503	0,263	7,432E-07	9	
Psmc3	4,47491E-11	0,38981	0,93	0,761	7,51382E-07	9	
Sms	4,52943E-11	0,37735	0,846	0,6	7,60536E-07	9	
Nudt16l1	4,54042E-11	0,27721	0,49	0,243	7,62382E-07	9	
Vimp	5,04344E-11	0,4162	0,678	0,422	8,46844E-07	9	
Smim8	5,14674E-11	0,2843	0,49	0,246	8,64188E-07	9	
Grcc10	5,31363E-11	0,31397	0,434	0,208	8,92212E-07	9	
Mrpl55	5,53306E-11	0,3282	0,559	0,305	9,29056E-07	9	
Gm5914	5,59603E-11	0,32683	0,559	0,302	9,3963E-07	9	
Ets1	5,62514E-11	0,2805	0,483	0,235	9,44518E-07	9	

Mrpl11	5,77524E-11	0,32072	0,622	0,348	9,69721E-07	9	
Arpc5l	5,82431E-11	0,38363	0,762	0,515	9,77959E-07	9	
Pet100	5,8298E-11	0,40608	0,86	0,645	9,78881E-07	9	
Ramp1	5,8749E-11	0,39421	0,818	0,558	9,86455E-07	9	
Psmc4	6,19212E-11	0,36291	0,839	0,55	1,03972E-06	9	
Mfap2	6,38241E-11	0,37131	0,559	0,312	1,07167E-06	9	
Kcnj3	6,65556E-11	0,25705	0,559	0,281	1,11753E-06	9	
Mrps6	6,69189E-11	0,2873	0,65	0,368	1,12364E-06	9	
Akr7a5	6,74072E-11	0,26949	0,427	0,2	1,13183E-06	9	
Aldoa	7,03875E-11	0,3241	1	0,996	1,18188E-06	9	
Ndufc2	7,17217E-11	0,38753	0,923	0,814	1,20428E-06	9	
D10Jhu81e	7,55472E-11	0,27649	0,427	0,203	1,26851E-06	9	
Supt4a	7,56623E-11	0,39583	0,643	0,408	1,27045E-06	9	
Fam213b	7,69207E-11	0,27913	0,385	0,176	1,29158E-06	9	
Tspo	7,80487E-11	0,44038	0,874	0,657	1,31052E-06	9	
Commd7	8,13095E-11	0,29337	0,643	0,385	1,36527E-06	9	
Tmco1	8,17446E-11	0,38175	0,839	0,597	1,37257E-06	9	
Sdhd	8,75455E-11	0,28742	0,622	0,348	1,46998E-06	9	
Psmb7	9,22944E-11	0,39835	0,909	0,702	1,54971E-06	9	
Diablo	9,28062E-11	0,31813	1	0,979	1,55831E-06	9	
Tubb2b	9,47555E-11	0,35639	1	0,985	1,59104E-06	9	
Clu	9,53886E-11	0,44603	0,664	0,421	1,60167E-06	9	
Hcfc1r1	9,77009E-11	0,3514	0,979	0,901	1,6405E-06	9	
Hspa5	9,78822E-11	0,50629	0,93	0,825	1,64354E-06	9	
1810043G02	9,92602E-11	0,28699	0,531	0,28	1,66668E-06	9	
Ldhb	1,01865E-10	0,34222	0,573	0,319	1,71042E-06	9	
Alad	1,02293E-10	0,33835	0,538	0,295	1,7176E-06	9	
Cpm	1,04668E-10	0,32195	0,392	0,184	1,75748E-06	9	
Insm1	1,10673E-10	0,27803	0,524	0,268	1,8583E-06	9	
Gpx1	1,13802E-10	0,38398	0,58	0,34	1,91085E-06	9	
Psmd12	1,15176E-10	0,37268	0,832	0,602	1,93391E-06	9	
Med28	1,16347E-10	0,35754	0,769	0,512	1,95358E-06	9	
Rab24	1,18455E-10	0,32362	0,587	0,333	1,98897E-06	9	
Fh1	1,20436E-10	0,32931	0,573	0,326	2,02224E-06	9	
Mrpl15	1,23413E-10	0,32096	0,573	0,326	2,07222E-06	9	
Psmb4	1,31015E-10	0,37627	0,951	0,788	2,19987E-06	9	
Phactr1	1,31729E-10	0,40467	0,909	0,716	2,21185E-06	9	
Rpn2	1,33234E-10	0,33879	0,713	0,447	2,23714E-06	9	
Mrps16	1,33436E-10	0,31786	0,636	0,373	2,24052E-06	9	
Pdia3	1,35124E-10	0,37036	0,958	0,812	2,26887E-06	9	
Nubp2	1,39173E-10	0,27211	0,42	0,201	2,33685E-06	9	
Acot13	1,39539E-10	0,28001	0,497	0,256	2,343E-06	9	
Fxr1	1,50851E-10	0,38573	0,916	0,751	2,53294E-06	9	
Txndc17	1,54242E-10	0,38571	0,874	0,7	2,58988E-06	9	
Nmt1	1,58178E-10	0,39127	0,986	0,921	2,65597E-06	9	
Cycs	1,59259E-10	0,36935	0,776	0,515	2,67411E-06	9	
Gabarap	1,62934E-10	0,32912	0,986	0,922	2,73583E-06	9	
Spats2l	1,69753E-10	0,36026	0,811	0,534	2,85033E-06	9	

Psmc1	1,72752E-10	0,35442	0,902	0,649	2,90068E-06	9	
Sarnp	1,75643E-10	0,35588	0,72	0,459	2,94922E-06	9	
Eif3k	1,82764E-10	0,39945	0,93	0,709	3,06879E-06	9	
Pgam1	1,92926E-10	0,4178	0,937	0,81	3,23942E-06	9	
Pdcd5	1,98126E-10	0,34036	0,944	0,786	3,32673E-06	9	
Slc25a11	2,04323E-10	0,34386	0,699	0,447	3,43078E-06	9	
Pak1	2,12662E-10	0,40973	0,825	0,591	3,5708E-06	9	
Npm1	2,18724E-10	0,33667	0,979	0,889	3,67259E-06	9	
Mrps14	2,28934E-10	0,32235	0,65	0,394	3,84403E-06	9	
Pop7	2,30061E-10	0,32	0,706	0,42	3,86296E-06	9	
Commd4	2,3615E-10	0,29545	0,455	0,23	3,96519E-06	9	
Calm1	2,43881E-10	0,25318	1	1	4,09501E-06	9	
Arl3	2,48402E-10	0,35097	0,762	0,484	4,17092E-06	9	
Riiad1	2,51603E-10	0,32881	0,552	0,308	4,22466E-06	9	
Stmn2	2,54391E-10	0,30905	1	1	4,27148E-06	9	
Fnta	2,75805E-10	0,30765	0,615	0,351	4,63104E-06	9	
Eif3f	2,87201E-10	0,35507	0,881	0,688	4,82239E-06	9	
Spcs1	2,90601E-10	0,37664	0,797	0,53	4,87949E-06	9	
Dph3	3,06386E-10	0,30404	0,608	0,36	5,14452E-06	9	
Smim11	3,14441E-10	0,27536	0,42	0,206	5,27977E-06	9	
Galk1	3,31344E-10	0,26196	0,399	0,19	5,5636E-06	9	
Fkbp3	3,42443E-10	0,38098	0,874	0,659	5,74997E-06	9	
Cetn4	3,4368E-10	0,25334	0,329	0,143	5,77073E-06	9	
Ost4	3,50433E-10	0,36272	0,727	0,488	5,88412E-06	9	
Prdx1	3,54383E-10	0,3336	1	0,957	5,95045E-06	9	
Chrac1	3,55796E-10	0,26546	0,636	0,362	5,97418E-06	9	
Mrps33	3,67956E-10	0,38181	0,916	0,77	6,17835E-06	9	
Hmg20b	3,75017E-10	0,33758	0,608	0,358	6,29691E-06	9	
Bax	3,77278E-10	0,35421	0,678	0,435	6,33488E-06	9	
Tcp1	3,80911E-10	0,35821	0,867	0,642	6,39588E-06	9	
Snhg6	4,10796E-10	0,28022	0,441	0,22	6,89767E-06	9	
Lyrm2	4,21433E-10	0,32503	0,58	0,336	7,07627E-06	9	
Lsm5	4,22749E-10	0,35041	0,594	0,347	7,09837E-06	9	
Acadm	4,24609E-10	0,34873	0,587	0,347	7,1296E-06	9	
Psma1	4,3938E-10	0,361	0,783	0,551	7,37763E-06	9	
Hexa	4,72098E-10	0,28241	0,476	0,249	7,92699E-06	9	
Sdc2	4,80458E-10	0,31709	0,476	0,251	8,06737E-06	9	
0610009B22	5,01344E-10	0,28596	0,552	0,304	8,41806E-06	9	
Npdc1	5,03651E-10	0,32033	0,979	0,909	8,45681E-06	9	
1810043H04	5,38244E-10	0,28928	0,636	0,372	9,03765E-06	9	
Atp7a	5,57145E-10	0,29201	0,462	0,237	9,35501E-06	9	
Clic1	5,73839E-10	0,32435	0,986	0,843	9,63532E-06	9	
Bola3	5,83833E-10	0,32534	0,657	0,4	9,80314E-06	9	
Atp6v0b	6,08036E-10	0,32284	0,993	0,955	1,02095E-05	9	
Ifi27	6,16813E-10	0,39935	0,944	0,794	1,03569E-05	9	
Eif6	6,36211E-10	0,32796	0,608	0,363	1,06826E-05	9	
0610011F06	6,41934E-10	0,3026	0,538	0,302	1,07787E-05	9	
Hsbp1	6,5273E-10	0,38568	0,888	0,734	1,096E-05	9	

Psma6	6,68364E-10	0,34508	0,839	0,602	1,12225E-05	9	
Snf8	7,16046E-10	0,29468	0,671	0,4	1,20231E-05	9	
Dbi	7,1861E-10	0,35414	0,734	0,482	1,20662E-05	9	
Rras	7,41921E-10	0,31352	0,552	0,31	1,24576E-05	9	
Mrps24	7,42284E-10	0,34933	0,65	0,397	1,24637E-05	9	
Nhp2	7,73138E-10	0,28131	0,476	0,247	1,29818E-05	9	
Prdx6	7,86342E-10	0,31477	0,448	0,237	1,32035E-05	9	
Ramp2	7,88174E-10	0,36832	0,434	0,224	1,32342E-05	9	
Fam96b	8,09233E-10	0,32175	0,713	0,445	1,35878E-05	9	
Gsn	8,49165E-10	0,28272	0,517	0,285	1,42583E-05	9	
Hspb1	8,75005E-10	0,3223	0,566	0,301	1,46922E-05	9	
Atp5j	9,03812E-10	0,32865	0,951	0,833	1,51759E-05	9	
Asns	9,11552E-10	0,32194	0,587	0,344	1,53059E-05	9	
Spock1	9,15681E-10	0,2733	0,552	0,297	1,53752E-05	9	
Ccs	9,59058E-10	0,28714	0,469	0,247	1,61035E-05	9	
Lrrc73	1,03631E-09	0,2959	0,455	0,24	1,74007E-05	9	
Rgs10	1,04888E-09	0,31623	0,51	0,29	1,76118E-05	9	
Ostc	1,06152E-09	0,34289	0,657	0,43	1,7824E-05	9	
Gria3	1,0661E-09	0,25089	0,538	0,283	1,79008E-05	9	
Eapp	1,07432E-09	0,39161	0,804	0,552	1,8039E-05	9	
Mrfap1	1,1142E-09	0,35343	0,951	0,784	1,87085E-05	9	
Cops4	1,12066E-09	0,32286	0,559	0,331	1,88171E-05	9	
Taf10	1,14919E-09	0,27327	0,65	0,383	1,9296E-05	9	
Wdr61	1,25475E-09	0,35389	0,783	0,517	2,10685E-05	9	
Uqcc3	1,26461E-09	0,31628	0,559	0,327	2,1234E-05	9	
Uqcrfs1	1,28796E-09	0,34074	0,79	0,539	2,16261E-05	9	
Txndc15	1,29057E-09	0,3012	0,601	0,352	2,167E-05	9	
Ostf1	1,31663E-09	0,28363	0,35	0,164	2,21076E-05	9	
Tnni1	1,35032E-09	0,28728	0,434	0,221	2,26733E-05	9	
Ndufs6	1,35253E-09	0,33011	0,902	0,657	2,27103E-05	9	
Malsu1	1,37096E-09	0,2878	0,483	0,261	2,30198E-05	9	
Pcbd2	1,39587E-09	0,29709	0,476	0,256	2,3438E-05	9	
Snx17	1,56476E-09	0,33533	0,573	0,334	2,62738E-05	9	
Hist3h2ba	1,57785E-09	0,37436	0,741	0,486	2,64936E-05	9	
Psmb6	1,61404E-09	0,33868	0,93	0,774	2,71013E-05	9	
Selk	1,6428E-09	0,30727	0,993	0,973	2,75842E-05	9	
Galnt6	1,69253E-09	0,25392	0,441	0,219	2,84193E-05	9	
Psmd8	1,72075E-09	0,34996	0,846	0,62	2,88931E-05	9	
Ifitm2	1,73299E-09	0,29975	1	0,952	2,90986E-05	9	
Sh3kbp1	1,74253E-09	0,30649	0,671	0,412	2,92588E-05	9	
Uchl5	1,76383E-09	0,26074	0,413	0,207	2,96165E-05	9	
Lhfpl5	1,80734E-09	0,2979	0,636	0,393	3,0347E-05	9	
Pebp1	1,80748E-09	0,29973	1	0,961	3,03494E-05	9	
Zfp580	1,81321E-09	0,32597	0,51	0,288	3,04457E-05	9	
Zcrb1	1,81467E-09	0,31131	0,804	0,557	3,04701E-05	9	
Sars	1,83394E-09	0,3343	0,909	0,724	3,07937E-05	9	
Surf1	1,89394E-09	0,28382	0,538	0,301	3,18012E-05	9	
Aamdc	1,90831E-09	0,28484	0,462	0,246	3,20424E-05	9	

Ccdc80	1,92062E-09	0,31088	0,58	0,336	3,22491E-05	9	
Fam173a	1,92382E-09	0,29483	0,643	0,383	3,23028E-05	9	
Cyb5a	2,01878E-09	0,40757	0,818	0,611	3,38974E-05	9	
Abat	2,03826E-09	0,26372	0,371	0,176	3,42245E-05	9	
Fars2	2,03853E-09	0,25082	0,336	0,154	3,4229E-05	9	
Psme1	2,0509E-09	0,33241	0,986	0,851	3,44367E-05	9	
Yif1b	2,05526E-09	0,32747	0,734	0,479	3,45099E-05	9	
Chmp5	2,09899E-09	0,37607	0,902	0,727	3,52441E-05	9	
Trnt1	2,13491E-09	0,25848	0,497	0,267	3,58473E-05	9	
Cacybp	2,17229E-09	0,30885	0,839	0,563	3,64749E-05	9	
Psmb2	2,19786E-09	0,35342	0,902	0,705	3,69043E-05	9	
Nell2	2,1981E-09	0,25677	0,427	0,215	3,69083E-05	9	
Myo1b	2,25129E-09	0,27368	0,517	0,282	3,78014E-05	9	
Calr	2,27958E-09	0,38005	0,972	0,942	3,82765E-05	9	
Commd6	2,28386E-09	0,31184	0,483	0,271	3,83484E-05	9	
Erp29	2,34299E-09	0,35773	0,909	0,793	3,93412E-05	9	
Rpl28	2,41319E-09	0,32	1	0,965	4,05199E-05	9	
Mrpl41	2,4144E-09	0,32854	0,741	0,484	4,05401E-05	9	
Ifngr2	2,44944E-09	0,27171	0,531	0,294	4,11285E-05	9	
Nsa2	2,80263E-09	0,39864	0,881	0,74	4,7059E-05	9	
Mrpl14	2,85435E-09	0,34103	0,678	0,448	4,79273E-05	9	
Rpl8	2,91691E-09	0,30114	1	0,971	4,89779E-05	9	
Fam195b	2,93492E-09	0,38538	0,832	0,631	4,92802E-05	9	
Coq7	2,95256E-09	0,30964	0,622	0,379	4,95765E-05	9	
Smim19	2,95496E-09	0,31418	0,559	0,333	4,96167E-05	9	
Nol7	2,99715E-09	0,32473	0,804	0,551	5,03251E-05	9	
Mrps18b	3,06962E-09	0,27836	0,441	0,232	5,1542E-05	9	
Slc35b1	3,0729E-09	0,3027	0,643	0,403	5,1597E-05	9	
S100a10	3,21309E-09	0,2783	1	0,985	5,39509E-05	9	
Ap1s1	3,22389E-09	0,349	0,804	0,599	5,41324E-05	9	
Prelid1	3,26089E-09	0,34471	0,783	0,559	5,47536E-05	9	
Pdcd10	3,28145E-09	0,36574	0,818	0,606	5,50989E-05	9	
Nudt2	3,28467E-09	0,28402	0,385	0,192	5,5153E-05	9	
Nsmce1	3,52085E-09	0,30107	0,65	0,397	5,91185E-05	9	
Adk	3,53063E-09	0,25162	0,434	0,221	5,92829E-05	9	
Rpl21	3,54337E-09	0,30398	1	0,968	5,94968E-05	9	
Ric8	3,59149E-09	0,31683	0,748	0,483	6,03048E-05	9	
Timm8b	3,66575E-09	0,32248	0,867	0,646	6,15515E-05	9	
Bad	3,72548E-09	0,33544	0,587	0,364	6,25545E-05	9	
Yif1a	3,73147E-09	0,28543	0,51	0,287	6,26551E-05	9	
Rps19bp1	3,76563E-09	0,25727	0,497	0,269	6,32287E-05	9	
Cebpzos	3,87351E-09	0,28377	0,462	0,25	6,504E-05	9	
Mrps26	4,04036E-09	0,29667	0,615	0,374	6,78417E-05	9	
Bloc1s2	4,1718E-09	0,29027	0,455	0,241	7,00486E-05	9	
Mrpl43	4,17571E-09	0,32929	0,608	0,389	7,01144E-05	9	
Npy1r	4,37925E-09	0,28715	0,378	0,183	7,3532E-05	9	
Akap17b	4,39502E-09	0,28024	0,51	0,29	7,37968E-05	9	
Ranbp1	4,43179E-09	0,36075	0,874	0,685	7,44142E-05	9	

Hdac1	4,46526E-09	0,27044	0,455	0,244	7,49762E-05	9	
Pigyl	4,65425E-09	0,27376	0,497	0,273	7,81494E-05	9	
Chrna5	4,78383E-09	0,25948	0,455	0,241	8,03252E-05	9	
Hspe1	4,97272E-09	0,34313	0,867	0,65	8,3497E-05	9	
Eif4a3	4,97747E-09	0,29782	0,629	0,39	8,35767E-05	9	
Psmg4	5,04528E-09	0,28429	0,664	0,407	8,47154E-05	9	
Ndufaf3	5,06319E-09	0,33375	0,559	0,342	8,5016E-05	9	
Apbb1	5,08298E-09	0,32112	0,993	0,943	8,53483E-05	9	
Ndufa9	5,23517E-09	0,30676	0,643	0,397	8,79038E-05	9	
Hras	5,31778E-09	0,36918	0,937	0,735	8,92908E-05	9	
Lrpap1	5,31845E-09	0,34801	0,93	0,711	8,93021E-05	9	
Ift27	5,39917E-09	0,2953	0,497	0,278	9,06574E-05	9	
Tceal3	5,59695E-09	0,34813	0,741	0,51	9,39784E-05	9	
Adrm1	5,61655E-09	0,32172	0,692	0,45	9,43074E-05	9	
Irs3	5,67358E-09	0,30968	0,462	0,251	9,52651E-05	9	
Gap43	5,8838E-09	0,29003	1	0,998	9,87948E-05	9	
Naa38	5,88901E-09	0,29206	0,622	0,37	9,88824E-05	9	
Ccdc107	5,94121E-09	0,26763	0,531	0,302	9,97589E-05	9	
Snrpc	5,96579E-09	0,30546	0,531	0,31	0,000100172	9	
Ppp2r3c	6,13969E-09	0,29998	0,58	0,342	0,000103092	9	
Kcnv1	6,15878E-09	0,25695	0,343	0,161	0,000103412	9	
Ndufs7	6,658E-09	0,35872	0,804	0,62	0,000111795	9	
Krtcap2	6,71509E-09	0,33684	0,888	0,649	0,000112753	9	
Mrpl21	6,90824E-09	0,26933	0,601	0,355	0,000115996	9	
Arl4a	7,04215E-09	0,32747	0,678	0,433	0,000118245	9	
Vps29	7,04551E-09	0,3231	0,783	0,557	0,000118301	9	
Gm8730	7,07095E-09	0,42963	0,629	0,426	0,000118728	9	
Tmem208	7,1072E-09	0,32461	0,587	0,377	0,000119337	9	
Cuta	7,25293E-09	0,31659	0,699	0,463	0,000121784	9	
Pa2g4	7,28933E-09	0,26892	0,594	0,352	0,000122395	9	
Dnlz	7,32977E-09	0,29042	0,552	0,32	0,000123074	9	
Pdia6	7,3819E-09	0,35086	0,734	0,509	0,000123949	9	
Ndnl2	7,40376E-09	0,26369	0,434	0,229	0,000124316	9	
Ap2b1	7,41524E-09	0,31055	0,993	0,939	0,000124509	9	
Vcan	7,4556E-09	0,3247	0,413	0,21	0,000125187	9	
Auts2	7,76934E-09	0,27395	0,909	0,688	0,000130455	9	
Vamp8	7,92994E-09	0,27678	0,385	0,195	0,000133152	9	
Dnajc19	9,18777E-09	0,28362	0,545	0,318	0,000154272	9	
Ift74	9,83959E-09	0,31491	0,545	0,324	0,000165217	9	
Emc7	1,05293E-08	0,37345	0,825	0,629	0,000176798	9	
Hsd17b11	1,07206E-08	0,27802	0,483	0,269	0,00018001	9	
Mrpl42	1,07738E-08	0,30872	0,601	0,365	0,000180902	9	
Nop10	1,08334E-08	0,33676	0,783	0,572	0,000181903	9	
Lix1	1,09373E-08	0,41021	0,881	0,769	0,000183648	9	
Lrrc59	1,1035E-08	0,25321	0,441	0,236	0,000185288	9	
Sfr1	1,1184E-08	0,37321	0,853	0,692	0,000187791	9	
Ndufab1	1,13211E-08	0,30935	0,888	0,706	0,000190093	9	
Tgfb1i1	1,13552E-08	0,31762	0,734	0,499	0,000190666	9	

Ssna1	1,14785E-08	0,34402	0,615	0,397	0,000192735	9	
Tmem205	1,21511E-08	0,30513	0,622	0,39	0,000204028	9	
Babam1	1,28418E-08	0,28069	0,566	0,334	0,000215627	9	
Psma3	1,35078E-08	0,30323	0,951	0,863	0,000226809	9	
Lamtor5	1,35285E-08	0,32735	0,664	0,43	0,000227157	9	
Snrpe	1,36557E-08	0,30136	0,839	0,593	0,000229293	9	
Acadvl	1,37614E-08	0,25843	0,559	0,326	0,000231067	9	
Faim	1,37629E-08	0,28138	0,524	0,306	0,000231093	9	
Arhgef4	1,46263E-08	0,28247	0,42	0,227	0,00024559	9	
Crem	1,46569E-08	0,27818	0,622	0,382	0,000246104	9	
Uqcrc1	1,56364E-08	0,29276	0,874	0,672	0,000262551	9	
Tnfaip1	1,5689E-08	0,279	0,601	0,368	0,000263434	9	
Slbp	1,61616E-08	0,25043	0,497	0,28	0,000271369	9	
Uqcc2	1,6372E-08	0,31501	0,937	0,722	0,000274902	9	
Nnat	1,65826E-08	0,60574	0,692	0,5	0,000278438	9	
Aurkaip1	1,66504E-08	0,31575	0,664	0,428	0,000279577	9	
Ndufs8	1,76E-08	0,30039	0,741	0,496	0,000295522	9	
Snapc5	1,78271E-08	0,27019	0,455	0,25	0,000299334	9	
Eef1g	1,79406E-08	0,28186	0,965	0,866	0,000301241	9	
Hist1h2bc	1,81538E-08	0,26779	0,392	0,202	0,000304821	9	
Dgcr6	1,88229E-08	0,27397	0,685	0,431	0,000316055	9	
Gtf3a	1,93286E-08	0,26189	0,531	0,303	0,000324547	9	
Atg12	1,93527E-08	0,30907	0,853	0,623	0,00032495	9	
Lamtor4	1,97534E-08	0,3293	0,657	0,422	0,000331679	9	
Snrnp27	1,9891E-08	0,31936	0,993	0,891	0,000333989	9	
Pin1	2,09764E-08	0,29706	0,734	0,488	0,000352214	9	
Hadh	2,11231E-08	0,25523	0,385	0,198	0,000354678	9	
Mllt11	2,14652E-08	0,3398	0,979	0,938	0,000360422	9	
Nhp2l1	2,16253E-08	0,32239	0,839	0,62	0,000363111	9	
Mycbp	2,1784E-08	0,34237	0,636	0,409	0,000365776	9	
Eif2s1	2,23087E-08	0,28088	0,566	0,34	0,000374585	9	
Taldo1	2,30583E-08	0,27947	0,86	0,638	0,000387171	9	
Tlx2	2,31673E-08	0,28272	0,986	0,971	0,000389002	9	
Usp50	2,44685E-08	0,29784	0,629	0,391	0,000410851	9	
Atp5a1	2,57751E-08	0,29377	0,972	0,878	0,000432789	9	
Tmed10	2,60074E-08	0,30021	0,916	0,735	0,00043669	9	
Eif1ad	2,69803E-08	0,2632	0,483	0,275	0,000453026	9	
Atp5g1	2,73402E-08	0,30471	0,993	0,93	0,00045907	9	
Sumo1	2,76032E-08	0,34192	0,832	0,634	0,000463485	9	
Arl2	2,77353E-08	0,31685	0,804	0,574	0,000465703	9	
Tcta	2,7852E-08	0,27168	0,601	0,363	0,000467662	9	
Nucb2	2,96441E-08	0,2785	0,783	0,502	0,000497755	9	
Ccdc124	2,99359E-08	0,34024	0,699	0,466	0,000502654	9	
Isca2	2,99459E-08	0,28354	0,559	0,339	0,000502822	9	
Gamt	3,03475E-08	0,25509	0,392	0,206	0,000509565	9	
Serbp1	3,30483E-08	0,27981	0,993	0,956	0,000554914	9	
Mrpl33	3,32034E-08	0,35105	0,804	0,585	0,000557518	9	
Tmem242	3,3943E-08	0,26983	0,573	0,348	0,000569937	9	

Commd3	3,74058E-08	0,3219	0,524	0,32	0,00062808	9	
Gtf2h5	3,76075E-08	0,28755	0,839	0,627	0,000631467	9	
Mocs2	3,7882E-08	0,36723	0,783	0,589	0,000636076	9	
Alyref	3,86408E-08	0,26738	0,517	0,304	0,000648818	9	
Erh	3,94224E-08	0,33898	0,818	0,599	0,000661942	9	
Ica1	4,01828E-08	0,30575	0,503	0,296	0,000674709	9	
Ap2m1	4,11871E-08	0,30057	0,986	0,944	0,000691572	9	
Prmt2	4,16795E-08	0,33491	0,888	0,712	0,00069984	9	
Manbal	4,18449E-08	0,29007	0,727	0,479	0,000702617	9	
Fkbp2	4,19787E-08	0,32175	0,93	0,746	0,000704864	9	
Rwdd1	4,2147E-08	0,33517	0,762	0,555	0,000707691	9	
Npr1	4,21647E-08	0,25582	0,608	0,361	0,000707988	9	
Timm22	4,2362E-08	0,25106	0,559	0,335	0,0007113	9	
Sfxn3	4,3245E-08	0,28515	0,552	0,347	0,000726126	9	
Cwc15	4,34384E-08	0,31687	0,762	0,52	0,000729374	9	
Fam98c	4,35314E-08	0,25735	0,517	0,307	0,000730935	9	
Cct5	4,35885E-08	0,31347	0,86	0,699	0,000731895	9	
Ddx1	4,62978E-08	0,33714	0,86	0,647	0,000777386	9	
Timm10	4,66455E-08	0,27882	0,538	0,324	0,000783224	9	
Dnpep	4,84862E-08	0,29141	0,601	0,378	0,000814133	9	
Snrpb2	4,96249E-08	0,28883	0,671	0,441	0,000833252	9	
Vdac2	4,96697E-08	0,34857	0,853	0,688	0,000834004	9	
1700023F06	5,00415E-08	0,32725	0,615	0,388	0,000840246	9	
Emc6	5,04149E-08	0,30056	0,58	0,369	0,000846517	9	
S100a13	5,04796E-08	0,3094	1	0,899	0,000847603	9	
Fdx1	5,18959E-08	0,25317	0,469	0,268	0,000871384	9	
Tbc1d7	5,2175E-08	0,27231	0,392	0,212	0,00087607	9	
B230118H07	5,25382E-08	0,25308	0,378	0,196	0,000882169	9	
Snrpg	5,25554E-08	0,30096	0,608	0,39	0,000882458	9	
Bag1	5,36429E-08	0,29535	0,916	0,694	0,000900718	9	
Znhit1	5,59742E-08	0,29413	0,888	0,725	0,000939863	9	
Vti1b	5,69618E-08	0,35021	0,685	0,479	0,000956446	9	
Rae1	5,69646E-08	0,27248	0,51	0,303	0,000956493	9	
Cd59a	6,02683E-08	0,30867	0,601	0,382	0,001011966	9	
Gnai2	6,13401E-08	0,27661	0,993	0,962	0,001029962	9	
Psmc5	6,13905E-08	0,30072	0,909	0,78	0,001030809	9	
Kcnk2	6,21209E-08	0,2723	0,993	0,94	0,001043071	9	
Yipf1	6,41848E-08	0,29089	0,441	0,251	0,001077726	9	
Ctnnbip1	7,03491E-08	0,30835	0,706	0,492	0,001181231	9	
Trnp1	7,10628E-08	0,28875	0,51	0,307	0,001193216	9	
Ten1	7,40742E-08	0,31287	0,608	0,403	0,001243779	9	
Snw1	7,46267E-08	0,31743	0,72	0,506	0,001253057	9	
Ufm1	7,61163E-08	0,32137	0,678	0,462	0,001278068	9	
Chmp2a	8,19983E-08	0,3053	0,958	0,83	0,001376833	9	
Stard4	8,32103E-08	0,3107	0,65	0,422	0,001397185	9	
1500011K16	8,53972E-08	0,30163	0,587	0,379	0,001433904	9	
Slit2	8,61711E-08	0,29784	0,671	0,427	0,001446899	9	
Cisd3	9,07062E-08	0,27266	0,636	0,412	0,001523047	9	

Echs1	9,3047E-08	0,29721	0,601	0,378	0,001562353	9	
Mrps36	9,33725E-08	0,27355	0,608	0,383	0,001567818	9	
Wbp5	9,69573E-08	0,2581	0,979	0,944	0,001628011	9	
Mif	9,82532E-08	0,30993	0,909	0,753	0,001649769	9	
Cops5	1,04986E-07	0,33713	0,86	0,74	0,001762827	9	
Ttc39b	1,05843E-07	0,2837	0,566	0,351	0,001777208	9	
Mrps7	1,07282E-07	0,27303	0,517	0,307	0,001801372	9	
Hotairm1	1,08132E-07	0,32157	0,664	0,434	0,00181565	9	
Tagln2	1,13104E-07	0,3097	1	0,97	0,001899131	9	
Gtf2b	1,13657E-07	0,29246	0,629	0,417	0,001908413	9	
Tceb2	1,15475E-07	0,298	1	0,958	0,001938947	9	
Gm16286	1,16163E-07	0,29206	0,65	0,431	0,001950493	9	
Ttc1	1,16981E-07	0,25041	0,594	0,368	0,001964235	9	
Mrps12	1,21534E-07	0,25838	0,727	0,491	0,00204067	9	
Gde1	1,23557E-07	0,29865	0,811	0,574	0,002074646	9	
Map1lc3b	1,28986E-07	0,32961	0,895	0,777	0,002165812	9	
Nudc	1,31035E-07	0,2627	0,916	0,705	0,002200202	9	
Cdk2ap1	1,36924E-07	0,27288	0,524	0,32	0,002299094	9	
Ndufs2	1,37383E-07	0,30546	0,72	0,498	0,002306795	9	
Suclg1	1,37884E-07	0,28092	0,657	0,421	0,00231521	9	
Arl6ip5	1,39206E-07	0,30417	0,706	0,49	0,002337412	9	
Bcl10	1,39617E-07	0,25881	0,531	0,328	0,002344306	9	
Letm1	1,72959E-07	0,27856	0,685	0,469	0,002904157	9	
Lcmt1	1,75731E-07	0,27014	0,594	0,387	0,002950705	9	
Rpp21	1,78887E-07	0,27538	0,566	0,36	0,00300369	9	
Sptssa	1,79767E-07	0,28176	0,811	0,598	0,003018464	9	
Dnajc2	1,86941E-07	0,25336	0,517	0,317	0,003138922	9	
Prdx5	1,87811E-07	0,29107	0,916	0,722	0,003153537	9	
Smim12	1,88352E-07	0,27052	0,524	0,322	0,003162623	9	
Grpel1	1,92758E-07	0,26654	0,601	0,384	0,0032366	9	
Serf2	1,93709E-07	0,27624	0,972	0,889	0,003252575	9	
Prr13	2,06778E-07	0,3215	0,727	0,532	0,003472001	9	
Exosc4	2,06801E-07	0,29644	0,587	0,383	0,003472402	9	
Jagn1	2,13851E-07	0,26299	0,455	0,264	0,003590777	9	
Vmp1	2,23322E-07	0,27263	0,573	0,363	0,003749806	9	
Mydgf	2,26057E-07	0,26387	0,643	0,429	0,003795729	9	
Capns1	2,36375E-07	0,27181	0,993	0,962	0,00396897	9	
Xrcc4	2,40238E-07	0,25622	0,392	0,219	0,004033838	9	
Gtf3c6	2,42945E-07	0,25266	0,413	0,236	0,004079285	9	
Txnl4a	2,55569E-07	0,30805	0,517	0,332	0,004291264	9	
Sra1	2,56429E-07	0,27986	0,664	0,447	0,004305704	9	
Ppp2r2d	2,67092E-07	0,31414	0,65	0,445	0,004484744	9	
Hmox2	2,6711E-07	0,2566	0,769	0,533	0,00448504	9	
Lsm6	2,69188E-07	0,294	0,559	0,361	0,004519944	9	
Smap1	2,8505E-07	0,31173	0,811	0,626	0,004786274	9	
2310011J03R	2,8989E-07	0,2732	0,524	0,325	0,004867546	9	
Esd	2,91344E-07	0,27367	0,769	0,56	0,004891954	9	
Mrpl28	2,9786E-07	0,28574	0,51	0,31	0,005001373	9	

Cnbp	2,98419E-07	0,29069	0,832	0,687	0,005010754	9	
Gnai1	3,00207E-07	0,27355	0,566	0,368	0,005040783	9	
Mrps15	3,14647E-07	0,26086	0,559	0,357	0,005283242	9	
Gps1	3,14662E-07	0,25855	0,699	0,48	0,005283484	9	
Psmg2	3,17868E-07	0,25134	0,587	0,375	0,005337316	9	
Bud31	3,28689E-07	0,25535	0,51	0,31	0,005519009	9	
Snrpa	3,31105E-07	0,25157	0,545	0,344	0,005559585	9	
1810037I17F	3,41232E-07	0,30428	0,797	0,611	0,005729627	9	
Nrp1	3,46719E-07	0,31224	0,93	0,794	0,005821755	9	
Mrpl34	3,47439E-07	0,25215	0,497	0,298	0,005833848	9	
Mitd1	3,53635E-07	0,25551	0,559	0,359	0,005937877	9	
Hist1h4d	3,64293E-07	0,25117	0,629	0,406	0,006116847	9	
Tusc3	3,86772E-07	0,2728	0,902	0,776	0,006494295	9	
BC031181	3,94686E-07	0,29463	0,916	0,758	0,006627166	9	
Sept7	4,09149E-07	0,26272	0,972	0,911	0,006870019	9	
Fzd3	4,33267E-07	0,25495	0,713	0,486	0,007274985	9	
Faf1	4,43464E-07	0,25707	0,545	0,343	0,0074462	9	
Mrpl12	4,43525E-07	0,26879	0,65	0,427	0,007447234	9	
Cisd1	4,54132E-07	0,26247	0,881	0,715	0,007625326	9	
Pam	4,81092E-07	0,25537	1	0,989	0,008078008	9	
4921524J17F	4,86458E-07	0,27411	0,636	0,421	0,008168118	9	
Pdzd11	4,94764E-07	0,28686	0,427	0,255	0,008307575	9	
Commd1	5,02058E-07	0,29396	0,657	0,461	0,00843005	9	
Eno1	5,071E-07	0,31291	0,762	0,583	0,008514713	9	
Pmvk	5,76176E-07	0,25562	0,797	0,542	0,009674566	9	
Aplp1	5,78718E-07	0,28519	0,986	0,943	0,00971725	9	
Larp7	5,84599E-07	0,30266	0,483	0,307	0,009816009	9	
Tmbim1	5,89742E-07	0,26895	0,531	0,337	0,009902352	9	
Pomp	6,16113E-07	0,27606	0,909	0,77	0,010345157	9	
Tpm4	6,16466E-07	0,28005	0,678	0,476	0,010351073	9	
Desi1	6,40126E-07	0,25458	0,413	0,238	0,010748353	9	
Cnpy2	6,87552E-07	0,293	0,566	0,371	0,011544688	9	
Polr2g	7,19573E-07	0,27815	0,685	0,482	0,012082356	9	
Ccdc184	7,67445E-07	0,26064	0,587	0,387	0,012886177	9	
Vamp7	7,78452E-07	0,25548	0,65	0,447	0,013070979	9	
Sec11a	7,93637E-07	0,28042	0,587	0,386	0,01332596	9	
Olfm1	8,10253E-07	0,29332	0,888	0,758	0,013604956	9	
Rpl27	8,29605E-07	0,28272	0,706	0,49	0,013929896	9	
Erlec1	8,46476E-07	0,27347	0,552	0,357	0,014213182	9	
Lsm8	8,60791E-07	0,25858	0,392	0,225	0,014453535	9	
Rtcb	8,61641E-07	0,26574	0,636	0,429	0,014467809	9	
Frg1	8,64352E-07	0,26672	0,839	0,643	0,014513339	9	
Emd	8,77605E-07	0,27841	0,741	0,524	0,014735867	9	
Nmt2	8,81042E-07	0,34734	0,916	0,814	0,014793583	9	
Brk1	9,19885E-07	0,2571	0,769	0,547	0,015445797	9	
Wbp11	9,76232E-07	0,265	0,769	0,55	0,016391909	9	
Tceb1	1,00753E-06	0,28562	0,818	0,615	0,016917377	9	
Nipsnap1	1,02932E-06	0,27026	0,594	0,4	0,017283379	9	

Sec11c	1,0605E-06	0,29925	0,399	0,236	0,017806788	9	
Rab4a	1,09129E-06	0,26387	0,587	0,385	0,018323899	9	
Carhsp1	1,15684E-06	0,26094	0,65	0,439	0,019424546	9	
Map7d2	1,16626E-06	0,33645	0,811	0,658	0,019582625	9	
Plpp1	1,20599E-06	0,29634	0,706	0,502	0,020249711	9	
H2-D1	1,22848E-06	0,3129	0,909	0,748	0,020627449	9	
Emc4	1,24624E-06	0,26225	0,643	0,442	0,020925651	9	
1110008P14	1,30266E-06	0,28438	0,839	0,638	0,021873013	9	
Ndufb3	1,36537E-06	0,2893	0,839	0,655	0,022925966	9	
Bhlhb9	1,39339E-06	0,25136	0,531	0,347	0,023396368	9	
Polr2i	1,44236E-06	0,26024	0,573	0,379	0,024218718	9	
Smn1	1,49437E-06	0,25922	0,51	0,319	0,02509194	9	
Necab2	1,49533E-06	0,32563	0,657	0,466	0,025108057	9	
Sh3gl2	1,50859E-06	0,26058	0,615	0,421	0,025330816	9	
Acadl	1,51257E-06	0,26628	0,909	0,764	0,025397635	9	
Dpy30	1,52535E-06	0,32263	0,538	0,372	0,025612071	9	
Banf1	1,5532E-06	0,25348	0,678	0,474	0,026079758	9	
Psmd2	1,56561E-06	0,28367	0,895	0,777	0,026288079	9	
Cryab	1,58931E-06	0,33679	0,615	0,413	0,026686064	9	
Med29	1,77236E-06	0,25751	0,566	0,374	0,029759634	9	
Atraid	1,79384E-06	0,25656	0,608	0,401	0,030120329	9	
Snrpd2	1,81853E-06	0,26888	0,895	0,741	0,03053489	9	
C1qbp	1,97911E-06	0,25913	0,727	0,5	0,033231244	9	
Gorasp2	1,98294E-06	0,25258	0,49	0,309	0,033295517	9	
1700037H04	1,98879E-06	0,25448	0,678	0,467	0,033393709	9	
Pop4	1,99382E-06	0,2627	0,483	0,303	0,033478231	9	
Pdap1	2,05945E-06	0,26076	0,986	0,94	0,034580289	9	
Mesdc2	2,07338E-06	0,26234	0,734	0,512	0,034814071	9	
Ndufv1	2,08123E-06	0,30439	0,825	0,624	0,034945935	9	
Psma5	2,11704E-06	0,30576	0,783	0,588	0,035547174	9	
Map2k2	2,29359E-06	0,28733	0,804	0,624	0,038511738	9	
Mcee	2,36199E-06	0,26771	0,622	0,427	0,039660164	9	
Stoml2	2,42202E-06	0,2766	0,552	0,366	0,040668133	9	
Nap1l5	2,57889E-06	0,27997	0,979	0,944	0,043302208	9	
Polr2f	2,80974E-06	0,25429	0,734	0,534	0,047178306	9	
Ddx21	2,88284E-06	0,27989	0,741	0,526	0,048405731	9	
Cdc37	2,89983E-06	0,29258	0,79	0,612	0,048691089	9	
Arpp19	3,20945E-06	0,25273	0,923	0,81	0,053889855	9	
Ube2a	3,42317E-06	0,2591	0,573	0,383	0,057478377	9	
Chmp2b	3,61203E-06	0,25194	0,643	0,447	0,060649589	9	
Sept6	3,66338E-06	0,26619	0,762	0,575	0,06151186	9	
Snrpb	3,71778E-06	0,28652	0,825	0,682	0,062425251	9	
2700060E02	4,09869E-06	0,2566	0,713	0,517	0,068821042	9	
Dbnl	4,22597E-06	0,25748	0,643	0,459	0,070958293	9	
Srpr	4,84855E-06	0,25783	0,685	0,487	0,08141192	9	
Anapc5	4,9902E-06	0,25114	0,916	0,852	0,083790371	9	
Rpap3	5,07501E-06	0,28214	0,594	0,415	0,08521444	9	
Vapb	5,21048E-06	0,26498	0,881	0,732	0,0874891	9	

Slc3a2	5,38467E-06	0,32603	0,762	0,649	0,090414053	9	
Tmem59	5,96808E-06	0,25278	0,923	0,849	0,100210039	9	
Mob4	6,42065E-06	0,26042	0,671	0,513	0,107809205	9	
Tmem176b	6,78579E-06	0,26542	0,993	0,941	0,113940172	9	
Scand1	6,7984E-06	0,27777	0,867	0,706	0,114151935	9	
Tm4sf1	8,00483E-06	0,27726	0,881	0,755	0,134409119	9	
Ndufa10	8,54989E-06	0,25718	0,804	0,635	0,143561226	9	
Vdac3	8,74261E-06	0,27298	0,776	0,596	0,146797203	9	
Smpd1	9,82904E-06	0,27178	0,573	0,402	0,165039354	9	
Fez1	9,87702E-06	0,25617	0,937	0,805	0,165844963	9	
Pfdn6	9,99848E-06	0,25772	0,72	0,525	0,16788448	9	
Cfl2	1,07764E-05	0,25984	0,867	0,707	0,180945945	9	
AW551984	1,15582E-05	0,33085	0,874	0,761	0,194073853	9	
Ppp1r14b	1,18406E-05	0,29433	0,72	0,569	0,198815692	9	
Sdf2	1,19583E-05	0,27818	0,699	0,514	0,200792631	9	
Pdcd4	1,20252E-05	0,33162	0,741	0,573	0,201914699	9	
Tomm22	1,22651E-05	0,25556	0,58	0,407	0,205943818	9	
Pcmt1	1,24652E-05	0,26108	0,881	0,751	0,209303416	9	
2810428I15R	1,33627E-05	0,25863	0,832	0,63	0,224373362	9	
Nacad	1,34634E-05	0,26422	0,643	0,468	0,226063476	9	
Rab2a	1,43498E-05	0,26205	0,951	0,9	0,240946914	9	
Emc2	1,80988E-05	0,25612	0,622	0,445	0,303897237	9	
Ndufs3	1,88554E-05	0,28274	0,713	0,532	0,316601791	9	
Serpinf1	2,29023E-05	0,31687	0,392	0,248	0,384551703	9	
Mphosph8	2,36647E-05	0,2702	0,741	0,591	0,397353732	9	
Ier3ip1	2,49401E-05	0,2761	0,797	0,622	0,418768923	9	
Tceal8	3,23193E-05	0,26871	0,664	0,497	0,54267301	9	
Tmem147	3,5881E-05	0,26722	0,699	0,562	0,602478644	9	
Ankra2	5,23165E-05	0,26508	0,832	0,701	0,878446933	9	
Sh3gl3	5,40305E-05	0,27203	0,804	0,724	0,907225637	9	
Cdkn1a	6,09777E-05	0,2643	0,629	0,444	1	9	
Stmn4	7,83147E-05	0,25002	0,622	0,46	1	9	
Mt3	9,74027E-05	0,30716	0,818	0,735	1	9	
Astn2	0,000112934	0,26868	0,503	0,359	1	9	
Bcas2	0,000116647	0,25664	0,727	0,593	1	9	
Ilk	0,0001513	0,2548	0,741	0,605	1	9	
E530001K10	0,000185267	0,25017	0,643	0,495	1	9	
