# Supplementary material for: The transcriptomes, connections and development of submucosal neuron classes in the mouse small intestine
Source: Nat Neurosci. 2025 May 29;28(6):1146–59. doi: 10.1038/s41593-025-01962-x (PMC12148937; doi:10.1038/s41593-025-01962-x)
Supplement: Supplementary file 11 — DE genes in P7 submucosal ENS clusters. [file 41593_2025_1962_MOESM11_ESM.pdf]

Supplementary Table 4. Enriched genes in ENS at P7

p-val: unadjusted p value; avg_logFC: log fold-change of the average expression between two groups. Positive values indicate high gene expression; pct.1: the percentage of cells where the gene is detected in the group; pct.2: The percentage of cells where the gene is detected in the rest of the dataset; p_val_adj: adjusted p-value based on Bonferroni correction using all genes in the dataset; Statistical analysis was two-sided but only enriched genes are shown. UMAP is shown in Extended Data Figure 8b. Only top 100 genes are shown, due to space limitation.

gene	p_val	avg_log2FC	pct.1	pct.2	p_val_adj	cluster
Itih5	0	2,917874821	0,911	0,217	0	0
Matn2	5,9755E-300	2,676872246	0,817	0,164	9,5889E-296	0
Gja1	4,1549E-269	4,37982931	0,404	0,028	6,6674E-265	0
Col20a1	1,3005E-225	2,257534163	0,812	0,219	2,0869E-221	0
Mmp2	1,7992E-222	2,40542036	0,794	0,231	2,8872E-218	0
Nrn1	2,1105E-215	3,129769937	0,486	0,067	3,3868E-211	0
Dbi	8,2499E-201	1,613856027	1	0,626	1,3239E-196	0
Pou3f1	2,4641E-199	3,439285609	0,439	0,058	3,9542E-195	0
Cnp	4,5419E-185	1,810502783	0,939	0,515	7,2883E-181	0
Lmo4	5,4107E-176	1,883355428	0,886	0,38	8,6826E-172	0
Tagln2	2,1867E-175	1,362816357	0,995	0,857	3,509E-171	0
Arpc1b	4,0468E-172	1,551095369	0,968	0,488	6,4939E-168	0
Plp1	1,4174E-168	1,624506164	0,935	0,402	2,2744E-164	0
Rpl13	1,678E-165	0,749002	1	1	2,6926E-161	0
Gpm6b	8,9803E-163	1,625968875	0,933	0,493	1,4411E-158	0
Postn	9,0337E-162	1,56248173	0,898	0,349	1,4496E-157	0
Galnt18	5,8273E-159	3,281904323	0,377	0,055	9,3511E-155	0
Irf6	6,4794E-157	3,211392493	0,338	0,041	1,0398E-152	0
Rps27	2,0369E-156	0,758866493	1	1	3,2686E-152	0
Rpl35a	8,9568E-155	0,792945614	1	0,999	1,4373E-150	0
Rps19	1,6936E-153	0,726458889	1	0,999	2,7177E-149	0
Rpl37a	1,8333E-152	0,661869415	1	1	2,9419E-148	0
Clec14a	8,8939E-151	2,728443811	0,48	0,101	1,4272E-146	0
Rps23	3,2459E-150	0,69230575	1	1	5,2087E-146	0
Rps29	1,0963E-148	0,626148765	1	1	1,7592E-144	0
Rpl18a	8,145E-148	0,758453866	1	1	1,307E-143	0
Eef1a1	2,1009E-147	0,657102423	1	1	3,3713E-143	0
Rps4x	5,529E-147	0,7305119	1	1	8,8724E-143	0
Anxa5	4,3797E-145	1,555104353	0,92	0,542	7,0281E-141	0
Tpt1	3,9737E-144	0,8131536	1	0,998	6,3766E-140	0
Rpl17	7,6085E-142	0,742473665	1	0,999	1,2209E-137	0
Pde8a	9,2435E-142	2,955667571	0,355	0,053	1,4833E-137	0
Ccnd2	5,9817E-138	2,047363998	0,66	0,21	9,5988E-134	0
Rpl30	5,6671E-136	1,0063711	0,997	0,936	9,0939E-132	0
Ccnd1	1,5388E-135	2,028795293	0,633	0,194	2,4693E-131	0
Rps16	2,0946E-134	0,682342339	1	0,999	3,3612E-130	0
Rpl35	2,1449E-134	0,699126825	1	0,998	3,4419E-130	0
Rps3a1	5,8841E-133	0,740360278	1	0,998	9,4422E-129	0
Rpl11	9,1238E-131	0,663858736	1	1	1,4641E-126	0

Rpl39	3,4351E-130	0,681193243	1	0,999	5,5123E-126	0
Rpl32	5,2445E-129	0,666072731	1	0,999	8,4158E-125	0
Rps15a	1,0649E-128	0,755525994	1	0,997	1,7089E-124	0
Aspa	5,2703E-128	2,645028802	0,396	0,075	8,4573E-124	0
Rpl13a	6,5101E-126	0,653604244	1	1	1,0447E-121	0
Rasgef1c	1,0954E-125	2,487840836	0,457	0,108	1,7578E-121	0
Rpl23a	1,9669E-125	0,636268311	1	0,999	3,1563E-121	0
Rpl36	6,8274E-125	0,681114739	1	0,999	1,0956E-120	0
Rps27a	1,806E-122	0,641138127	1	1	2,8981E-118	0
Rps17	2,9376E-120	0,696079561	1	0,996	4,7139E-116	0
Gulp1	4,0933E-118	1,960862663	0,614	0,208	6,5686E-114	0
Tax1bp3	1,1857E-116	1,653942027	0,761	0,378	1,9027E-112	0
Rpl9	2,1357E-116	0,641601525	1	0,998	3,4271E-112	0
Gjc3	3,0823E-116	1,72755671	0,667	0,239	4,9461E-112	0
Rps14	3,5908E-116	0,626744753	1	0,999	5,7621E-112	0
Serpinh1	4,1623E-116	1,232050286	0,952	0,525	6,6793E-112	0
Rps6	9,1812E-116	0,651148171	1	0,999	1,4733E-111	0
Rpl10	1,061E-115	0,566416665	1	1	1,7026E-111	0
Rpl24	2,2709E-114	0,668555298	1	0,999	3,6442E-110	0
Rpl34	5,1399E-114	0,683277864	1	0,997	8,2481E-110	0
Rps12	1,705E-113	0,851831743	1	0,942	2,736E-109	0
Camk2b	1,4312E-112	2,04786654	0,645	0,283	2,2967E-108	0
Pdlim4	2,9318E-112	2,054662662	0,565	0,179	4,7047E-108	0
Rpl7	3,0641E-112	0,738469373	1	0,99	4,917E-108	0
Vim	1,0212E-109	1,050963023	0,998	0,682	1,6387E-105	0
Rps5	1,0125E-104	0,556457098	1	0,999	1,6247E-100	0
Fbln1	2,2835E-104	1,436522839	0,744	0,322	3,6643E-100	0
Rps18	3,0614E-104	0,563996185	1	0,999	4,9126E-100	0
Rpl37	1,4138E-103	0,529590427	1	1	2,2686E-99	0
Rps20	2,9408E-103	0,736307893	1	0,985	4,719E-99	0
Ppic	9,5743E-103	1,479069086	0,756	0,345	1,53639E-98	0
Rpl23	3,2026E-102	0,566087978	1	0,999	5,13917E-98	0
Zeb2	4,8221E-102	1,197392032	0,898	0,562	7,73796E-98	0
Rplp2	8,4327E-102	0,582943913	1	0,999	1,3532E-97	0
Rps15	1,2558E-101	0,600382264	1	0,998	2,01524E-97	0
Rpl28	3,134E-101	0,71467794	1	0,982	5,02909E-97	0
Sox10	7,0387E-101	1,318841241	0,817	0,386	1,1295E-96	0
Rpl36a	7,8184E-101	0,726762936	1	0,986	1,25462E-96	0
Rps28	3,5377E-99	0,610405781	1	0,999	5,6769E-95	0
Rpl31	3,9433E-99	0,657749112	1	0,991	6,32778E-95	0
Rhob	1,33246E-98	1,644070607	0,754	0,437	2,1382E-94	0
Rps8	5,58645E-98	0,504506759	1	1	8,96458E-94	0
Itm2a	3,8862E-96	1,513327951	0,735	0,349	6,23619E-92	0
Rrad	7,44886E-96	2,576412439	0,377	0,094	1,19532E-91	0
Rps3	2,05333E-95	0,566545028	1	0,999	3,29497E-91	0
Rps11	3,96488E-95	0,581134478	1	0,999	6,36244E-91	0
Rap1a	6,44104E-95	1,347543643	0,787	0,458	1,03359E-90	0
Rpl27a	1,36506E-94	0,586661003	1	0,997	2,19051E-90	0

Cald1	1,93436E-94	1,421322266	0,761	0,403	3,10407E-90	0
Rps9	2,92433E-94	0,560220405	1	0,999	4,69268E-90	0
Ldb2	5,27883E-94	1,911140126	0,532	0,185	8,47094E-90	0
Fxyd1	7,44734E-94	1,078725221	0,932	0,529	1,19507E-89	0
Ajap1	7,68363E-94	2,484066966	0,295	0,055	1,23299E-89	0
Rpl26	2,08779E-93	0,549506099	1	1	3,35028E-89	0
Myl12a	5,18861E-92	1,133001343	0,879	0,549	8,32616E-88	0
Marcks	3,3709E-91	0,894015229	0,961	0,699	5,40928E-87	0
Rps10	3,61842E-91	0,674559355	0,995	0,981	5,80648E-87	0
Rpsa	7,17135E-90	0,628518711	1	0,991	1,15079E-85	0
Rpl10a	7,61029E-90	0,617785177	1	0,993	1,22122E-85	0
1700047M11Rik	9,5437E-90	2,542511077	0,287	0,054	1,53148E-85	0
Rpl22	4,86654E-89	0,909193924	0,959	0,794	7,80934E-85	0
Mcam	3,2958E-86	1,633338636	0,584	0,249	5,28878E-82	0
Apoe	1,5562E-186	1,578629644	1	0,58	2,4972E-182	1
Cst3	4,9273E-155	1,437227007	0,997	0,905	7,9068E-151	1
Tmprss5	3,2021E-128	1,899490513	0,633	0,198	5,1384E-124	1
Hes1	1,4732E-127	1,839343382	0,81	0,37	2,3641E-123	1
Sparc	6,1017E-125	1,125077203	1	0,783	9,7914E-121	1
Rbp1	4,4423E-124	1,204572793	0,993	0,655	7,1285E-120	1
Fabp7	8,3442E-120	1,193357531	0,99	0,524	1,339E-115	1
Gpr37l1	2,3798E-119	1,417500783	0,871	0,467	3,8188E-115	1
Plekhb1	1,9516E-116	1,31769898	0,889	0,452	3,1317E-112	1
Tgfb2	4,3503E-116	1,393500206	0,861	0,48	6,981E-112	1
Lpar1	3,7907E-112	1,362959795	0,813	0,351	6,0829E-108	1
Fos	4,3129E-111	1,631759885	0,928	0,65	6,9209E-107	1
Col18a1	1,3048E-109	1,447487715	0,798	0,373	2,0938E-105	1
Olfml3	7,0591E-108	1,384759775	0,772	0,344	1,1328E-103	1
Ckb	1,4636E-99	1,054437373	0,954	0,699	2,34867E-95	1
Entpd2	1,49908E-97	1,208160011	0,734	0,288	2,40557E-93	1
Atp1a2	1,97105E-96	1,487647668	0,696	0,296	3,16294E-92	1
Timp3	4,71485E-96	1,323351853	0,83	0,467	7,56592E-92	1
Junb	2,98972E-94	1,621923727	0,782	0,453	4,7976E-90	1
Hspa1a	4,29533E-94	1,910360959	0,664	0,298	6,89272E-90	1
Serpine2	7,63044E-94	0,979488888	0,983	0,607	1,22446E-89	1
Fxyd1	4,20266E-92	1,053391858	0,938	0,528	6,74401E-88	1
Tspan3	4,68839E-88	0,862466589	0,969	0,864	7,52346E-84	1
Cryab	1,96694E-87	0,90341511	0,985	0,798	3,15635E-83	1
Jun	3,82186E-85	1,459354775	0,864	0,619	6,13294E-81	1
Car12	1,76519E-84	1,619346898	0,616	0,27	2,83261E-80	1
Plcb1	3,48624E-84	1,687670297	0,563	0,226	5,59437E-80	1
Cyr61	4,52792E-82	1,801675945	0,617	0,271	7,26596E-78	1
Gas7	8,92549E-81	1,176279792	0,799	0,406	1,43227E-76	1
Egr1	2,45294E-79	1,754079837	0,604	0,283	3,93623E-75	1
Zbtb20	1,60112E-78	1,004585634	0,925	0,753	2,56931E-74	1
Cd59a	4,70963E-76	1,2220992	0,772	0,468	7,55754E-72	1
Rps14	4,69198E-75	0,48517651	1	0,999	7,52923E-71	1
Fibin	4,36637E-74	1,743561609	0,518	0,21	7,00671E-70	1

Nell2	1,98033E-73	1,192238958	0,768	0,466	3,17783E-69	1
Gpm6b	1,86172E-72	0,938211931	0,873	0,5	2,9875E-68	1
Crtac1	4,326E-72	1,486530198	0,647	0,361	6,94193E-68	1
Arpc1b	6,54451E-72	0,920636874	0,868	0,5	1,0502E-67	1
Rps27	3,15332E-71	0,476443927	1	1	5,06014E-67	1
S1pr3	5,01525E-70	1,85951181	0,389	0,123	8,04797E-66	1
Gsn	2,31802E-68	1,225046361	0,705	0,403	3,71973E-64	1
Pmepa1	5,17911E-68	1,361662269	0,6	0,284	8,31091E-64	1
Tpt1	1,17265E-67	0,52451633	1	0,998	1,88174E-63	1
Tbx3os1	1,53223E-67	1,531354595	0,563	0,254	2,45878E-63	1
Metrn	3,95709E-67	1,091844832	0,753	0,411	6,34994E-63	1
Taf13	3,87585E-65	1,198946801	0,674	0,395	6,21958E-61	1
Sox10	2,06105E-63	1,072942171	0,731	0,397	3,30737E-59	1
Gsta4	7,22935E-63	1,344634583	0,482	0,189	1,16009E-58	1
Vim	9,86102E-62	0,711514216	0,993	0,683	1,5824E-57	1
Eif3f	1,0464E-61	0,622706963	0,991	0,926	1,67916E-57	1
Rpl35a	1,2004E-61	0,464586575	1	0,999	1,92629E-57	1
Mmd2	1,3529E-61	1,469141866	0,455	0,176	2,17099E-57	1
Ttyh1	6,70505E-61	1,188326286	0,616	0,309	1,07596E-56	1
Bsg	1,04711E-60	0,658850494	0,988	0,938	1,6803E-56	1
Prdx6	4,08241E-60	1,002198116	0,782	0,527	6,55105E-56	1
Cnn3	4,19136E-60	0,780884684	0,926	0,741	6,72587E-56	1
Shc4	6,1506E-59	1,765707638	0,396	0,146	9,86987E-55	1
Dhrs3	1,40931E-58	1,265852553	0,569	0,274	2,26152E-54	1
Crym	2,61872E-58	1,61480952	0,46	0,191	4,20226E-54	1
Dbi	1,35899E-57	0,583046775	0,99	0,627	2,18077E-53	1
Rpl37a	2,40942E-57	0,367416398	1	1	3,8664E-53	1
Mef2c	2,20424E-56	1,059545245	0,702	0,421	3,53714E-52	1
Rpl36	2,23324E-56	0,404750727	1	0,999	3,58368E-52	1
Ndrg2	3,16859E-56	0,874444831	0,834	0,67	5,08464E-52	1
Socs3	3,37418E-56	1,788267683	0,443	0,189	5,41455E-52	1
Grb14	3,56278E-56	1,398908235	0,491	0,218	5,71719E-52	1
Rpl18a	3,99036E-56	0,416908744	1	1	6,40334E-52	1
Serpinh1	5,72592E-56	0,789306158	0,864	0,535	9,18838E-52	1
Nkain4	1,79108E-55	1,025879117	0,672	0,363	2,87414E-51	1
Rpl13	3,09489E-55	0,383170609	1	1	4,96636E-51	1
Ctgf	4,29372E-55	1,381261299	0,597	0,325	6,89014E-51	1
Tsc22d4	9,82806E-55	1,135923579	0,695	0,442	1,57711E-50	1
Ptn	1,9958E-54	0,599723105	0,844	0,577	3,20266E-50	1
Col27a1	3,66122E-54	1,768469166	0,376	0,141	5,87516E-50	1
Rps28	3,97945E-53	0,408805448	1	0,999	6,38583E-49	1
Col9a2	9,1215E-53	0,830953557	0,527	0,219	1,46373E-48	1
Nid1	4,03716E-52	1,209837151	0,542	0,26	6,47842E-48	1
Pdgfa	4,78959E-52	1,139532904	0,676	0,429	7,68585E-48	1
Ppib	6,26991E-52	0,712452388	0,882	0,759	1,00613E-47	1
Sep15	1,49867E-51	0,663146778	0,943	0,842	2,40491E-47	1
Qpct	1,57608E-51	1,055035255	0,509	0,233	2,52914E-47	1
Rpl34	5,97734E-51	0,414067199	1	0,997	9,59184E-47	1

Marcks	1,36361E-50	0,643178665	0,895	0,707	2,18818E-46	1
Crip2	3,10994E-50	0,550811474	0,993	0,963	4,99052E-46	1
Zfp36l1	2,98678E-49	1,107758851	0,59	0,311	4,79288E-45	1
Gas1	7,16559E-48	1,407829707	0,449	0,208	1,14986E-43	1
Poc1a	1,6806E-47	1,403231847	0,443	0,195	2,69686E-43	1
Sepp1	6,76469E-47	1,05618425	0,617	0,365	1,08553E-42	1
Gfra1	1,37291E-46	1,235124856	0,551	0,312	2,20311E-42	1
Rpl3	8,49669E-46	0,362920332	1	0,999	1,36346E-41	1
Plp1	2,04749E-45	0,681775843	0,767	0,421	3,2856E-41	1
Olfml2a	3,09167E-45	1,51234614	0,343	0,13	4,96121E-41	1
Zfp36l2	7,10205E-45	1,167122792	0,547	0,295	1,13967E-40	1
Ngfr	8,9254E-45	1,066735098	0,672	0,415	1,43226E-40	1
Mbp	9,24743E-45	1,073963797	0,576	0,315	1,48394E-40	1
Uba52	1,44008E-44	0,517946817	0,993	0,947	2,31089E-40	1
Hmcn1	2,38401E-44	1,57507346	0,401	0,179	3,82563E-40	1
Myl12a	4,39756E-44	0,75304298	0,798	0,559	7,05676E-40	1
Laptm4a	9,49946E-44	0,597169531	0,947	0,86	1,52438E-39	1
Ldhb	1,4376E-43	0,842712368	0,731	0,533	2,30692E-39	1
Rpl30	2,11871E-43	0,483024806	0,99	0,937	3,39989E-39	1
Qpct	7,1284E-273	3,003005797	0,876	0,207	1,1439E-268	2
Gsta4	3,0572E-259	2,885446209	0,814	0,166	4,9058E-255	2
Ptn	6,1599E-258	2,675336568	1	0,569	9,8848E-254	2
Apoe	1,5737E-256	2,714646292	1	0,59	2,5253E-252	2
Fbln2	5,6547E-235	2,944147688	0,679	0,114	9,0741E-231	2
Mmd2	2,9983E-233	2,736864249	0,762	0,155	4,8113E-229	2
Olfml3	2,9601E-230	2,368910413	0,961	0,336	4,75E-226	2
Tbx3os1	6,2352E-226	2,60348994	0,861	0,234	1,0006E-221	2
Tmprss5	1,4949E-225	2,561095815	0,818	0,191	2,3989E-221	2
Entpd2	3,3805E-224	2,125468507	0,961	0,278	5,4246E-220	2
Cthrc1	1,3582E-213	2,169475427	0,876	0,224	2,1796E-209	2
Prss56	7,8841E-203	4,632805493	0,298	0,016	1,2652E-198	2
Grb14	3,9511E-201	2,442404413	0,794	0,197	6,3404E-197	2
Chadl	4,8782E-196	2,549745179	0,784	0,224	7,8281E-192	2
Taf13	1,9759E-190	2,291990093	0,916	0,379	3,1708E-186	2
Sparc	7,8231E-182	1,53227069	1	0,788	1,2554E-177	2
S1pr3	1,4563E-175	2,427333445	0,6	0,111	2,337E-171	2
Vcam1	6,7711E-171	3,259318903	0,375	0,04	1,0866E-166	2
H19	1,9136E-169	3,71325008	0,385	0,045	3,0708E-165	2
Gpr37	3,6237E-169	3,022278585	0,467	0,07	5,815E-165	2
Gm2115	1,6666E-165	2,485706274	0,645	0,141	2,6744E-161	2
Tspan3	2,9389E-164	1,399867484	0,998	0,864	4,716E-160	2
Cpxm2	3,3662E-164	3,708219115	0,317	0,027	5,4017E-160	2
Gpr37l1	1,1305E-163	1,767292616	0,972	0,467	1,8141E-159	2
Cst3	3,7509E-159	1,518614916	1	0,907	6,0191E-155	2
Nid1	1,8054E-158	2,055730234	0,801	0,243	2,8972E-154	2
Hey2	3,2785E-158	2,668165863	0,493	0,081	5,261E-154	2
Lpar1	2,5784E-154	1,753626231	0,925	0,351	4,1376E-150	2
Tgfb2	8,9957E-147	1,673191379	0,966	0,479	1,4435E-142	2

Gas1	3,8541E-144	2,204986042	0,692	0,192	6,1847E-140	2
Atp1a2	1,1483E-142	1,897239239	0,835	0,293	1,8426E-138	2
Gsn	6,4724E-142	1,959340882	0,872	0,395	1,0386E-137	2
Ttyh1	2,5246E-141	1,900882054	0,824	0,298	4,0512E-137	2
Itm2b	3,2763E-141	0,83953576	1	0,998	5,2574E-137	2
Plekhb1	1,1032E-138	1,609875774	0,964	0,455	1,7703E-134	2
Ldhb	1,4173E-138	1,722763951	0,929	0,52	2,2743E-134	2
Slc18a2	2,8899E-138	2,501926205	0,593	0,156	4,6374E-134	2
Igfbp2	6,4663E-138	3,489208299	0,328	0,039	1,0376E-133	2
Fxyd3	1,791E-133	3,054247543	0,358	0,049	2,8741E-129	2
Scrg1	5,7302E-133	2,526479489	0,463	0,084	9,1952E-129	2
Rbp1	1,095E-131	1,373912217	0,994	0,662	1,7572E-127	2
Cmtm5	2,605E-130	2,045385988	0,685	0,2	4,1802E-126	2
Ctsb	4,1244E-127	1,267509833	0,983	0,86	6,6185E-123	2
Col11a1	2,4238E-125	2,499251941	0,473	0,094	3,8894E-121	2
Mest	1,095E-123	1,890440947	0,82	0,365	1,7571E-119	2
Col18a1	2,229E-120	1,533960333	0,906	0,372	3,5769E-116	2
Etl4	1,8223E-119	2,956807112	0,334	0,048	2,9242E-115	2
Phgdh	4,2995E-118	1,837130402	0,784	0,321	6,8994E-114	2
Itm2c	6,7119E-117	1,665514606	0,887	0,546	1,0771E-112	2
Fam107a	2,7196E-116	3,323320336	0,259	0,027	4,3641E-112	2
Fabp7	5,4234E-115	1,343345744	0,983	0,535	8,7029E-111	2
Timp3	4,9118E-114	1,458367929	0,912	0,468	7,8819E-110	2
Spint2	1,3948E-112	2,036523381	0,715	0,309	2,2382E-108	2
S100b	2,1335E-112	1,623242711	0,827	0,332	3,4237E-108	2
Hmcn1	5,9498E-111	2,002629498	0,591	0,167	9,5477E-107	2
Tsc22d4	3,5075E-110	1,506502528	0,878	0,431	5,6284E-106	2
Nkain4	5,6173E-110	1,569167541	0,846	0,354	9,0141E-106	2
Cpe	2,706E-106	1,605009451	0,916	0,64	4,3423E-102	2
Plcb1	2,507E-105	1,867818165	0,67	0,224	4,0231E-101	2
Tbc1d10a	2,9543E-105	1,975203448	0,608	0,189	4,7408E-101	2
Ptrf	4,9738E-105	2,301582473	0,448	0,098	7,9815E-101	2
Wnt6	7,0662E-105	2,268887533	0,415	0,083	1,1339E-100	2
Pla2g16	1,4495E-103	2,185513639	0,527	0,141	2,3261E-99	2
Ndrg2	4,5317E-101	1,302678274	0,944	0,664	7,27205E-97	2
Emilin1	7,9965E-101	1,923015209	0,597	0,2	1,2832E-96	2
Mdfi	2,49562E-98	2,27806423	0,424	0,093	4,00472E-94	2
Lrrtm1	5,46384E-98	1,757014443	0,597	0,186	8,76782E-94	2
Ndrg1	9,81375E-98	1,650530223	0,764	0,353	1,57481E-93	2
Mdk	6,61395E-97	1,674651955	0,719	0,283	1,06134E-92	2
Prdx6	6,71215E-97	1,342501762	0,91	0,521	1,0771E-92	2
Ugdh	7,6816E-96	1,857723552	0,649	0,247	1,23267E-91	2
Car12	1,30143E-92	1,68242783	0,704	0,269	2,0884E-88	2
Ngfr	2,03348E-91	1,431637222	0,833	0,407	3,26313E-87	2
Gdf10	2,44765E-90	2,906164883	0,272	0,043	3,92774E-86	2
Slc9a3r1	1,2288E-89	2,040343706	0,467	0,123	1,97185E-85	2
Cd59a	1,79868E-89	1,335922537	0,865	0,466	2,88634E-85	2
Col12a1	1,13656E-88	1,590468097	0,623	0,21	1,82383E-84	2

Gpx4	1,67652E-88	0,833138662	0,987	0,947	2,6903E-84	2
Eln	4,94551E-88	1,664062489	0,672	0,285	7,93606E-84	2
Islr	7,14633E-88	2,519919557	0,355	0,074	1,14677E-83	2
Ramp1	1,16929E-86	1,361551161	0,837	0,432	1,87636E-82	2
Pmepa1	4,08117E-86	1,510006561	0,715	0,281	6,54905E-82	2
Gfra1	3,21038E-85	1,565425378	0,704	0,303	5,1517E-81	2
Serpinh1	4,44085E-85	1,106330897	0,951	0,535	7,12623E-81	2
Cdkn1c	8,82765E-85	1,638692371	0,685	0,302	1,41657E-80	2
Dtna	1,32005E-84	2,075010698	0,512	0,167	2,11829E-80	2
Wdr1	1,53656E-84	1,561382454	0,752	0,405	2,46572E-80	2
Vim	1,63706E-84	1,014245909	1	0,689	2,62698E-80	2
Dnajc1	3,85836E-84	1,876994234	0,54	0,181	6,19151E-80	2
Fxyd1	7,18602E-84	1,103496728	0,944	0,537	1,15314E-79	2
Loxl1	8,06528E-83	2,508382925	0,336	0,072	1,29423E-78	2
Rasl11b	6,33888E-81	2,006228603	0,495	0,158	1,0172E-76	2
Mapre2	1,22895E-79	1,434216822	0,777	0,447	1,9721E-75	2
Tst	3,15735E-78	1,931701979	0,48	0,146	5,0666E-74	2
Gas7	8,01448E-78	1,134676324	0,874	0,408	1,28608E-73	2
mt-Co1	1,69474E-77	0,511693594	1	1	2,71955E-73	2
Sdc4	1,29852E-76	1,775785044	0,46	0,131	2,08373E-72	2
Mycl	5,13041E-74	1,982716272	0,454	0,151	8,23277E-70	2
Poc1a	1,53943E-72	1,474009727	0,557	0,19	2,47032E-68	2
Sepp1	1,54201E-72	1,245627645	0,769	0,357	2,47446E-68	2
Shc4	3,57952E-72	1,822707093	0,467	0,146	5,74405E-68	2
Art3	0	3,527907792	0,841	0,129	0	3
Hmgcs2	2,1412E-244	3,525892191	0,625	0,089	3,436E-240	3
Mal	2,8541E-233	3,724735404	0,464	0,043	4,58E-229	3
Scn7a	1,1145E-232	2,71583983	0,786	0,162	1,7884E-228	3
Sostdc1	1,4908E-207	3,213041643	0,686	0,138	2,3923E-203	3
Rarres2	1,087E-177	2,845819165	0,698	0,169	1,7443E-173	3
Plp1	8,7705E-177	1,917638863	0,98	0,413	1,4074E-172	3
Fbln5	5,4673E-175	2,456886032	0,807	0,24	8,7735E-171	3
Sfrp1	1,8542E-170	2,607483052	0,625	0,128	2,9754E-166	3
Entpd2	6,4073E-168	2,052253671	0,891	0,287	1,0282E-163	3
Arpc1b	6,2584E-158	1,775356337	0,98	0,5	1,0043E-153	3
Postn	2,4739E-148	1,848991541	0,923	0,362	3,9699E-144	3
Tpt1	1,4604E-130	0,931733813	1	0,998	2,3436E-126	3
Matn4	7,1641E-127	2,704816362	0,484	0,1	1,1496E-122	3
Dbi	1,0309E-121	1,373846611	1	0,636	1,6543E-117	3
Mbp	2,5473E-118	1,977108172	0,768	0,306	4,0876E-114	3
Marcks	1,5315E-115	1,356600791	0,966	0,706	2,4575E-111	3
Vim	6,8472E-105	1,196252675	1	0,691	1,0988E-100	3
Fam198b	2,4802E-100	2,928338677	0,311	0,048	3,97995E-96	3
Fabp7	6,7058E-99	1,295560999	0,989	0,537	1,07609E-94	3
Ifitm3	1,03267E-96	1,9565434	0,659	0,238	1,65713E-92	3
Abca8a	1,22878E-96	2,138712532	0,495	0,129	1,97183E-92	3
Sparc	7,16766E-95	1,083326207	1	0,789	1,15019E-90	3
Emp2	9,44273E-93	2,15168188	0,548	0,173	1,51527E-88	3

Cryab	1,01562E-89	1,011876608	0,993	0,802	1,62977E-85	3
Cnp	2,26029E-88	1,464454175	0,857	0,534	3,62709E-84	3
Igfbp7	3,6556E-87	1,69047799	0,564	0,186	5,86615E-83	3
Acot1	1,2938E-86	1,876073633	0,618	0,225	2,07616E-82	3
Ctnnal1	2,24908E-86	1,871218066	0,607	0,217	3,60909E-82	3
Myl12a	1,39503E-81	1,221905571	0,891	0,558	2,23861E-77	3
Dhrs3	3,71692E-81	1,664411072	0,682	0,273	5,96453E-77	3
Serpine2	1,46403E-80	1,03300047	0,986	0,616	2,34933E-76	3
B2m	9,07353E-80	1,549790714	0,798	0,448	1,45603E-75	3
Zeb2	5,11707E-79	1,171337396	0,905	0,571	8,21137E-75	3
Fxyd1	8,39587E-79	1,126679634	0,939	0,539	1,34728E-74	3
Lpar1	8,22129E-76	1,227542009	0,811	0,363	1,31927E-71	3
Cthrc1	1,16445E-75	1,553312345	0,636	0,247	1,8686E-71	3
Serpinh1	2,08552E-75	1,131741721	0,909	0,54	3,34663E-71	3
Eva1b	1,77846E-74	2,225164392	0,418	0,117	2,8539E-70	3
Mmp2	1,9717E-74	1,539257506	0,641	0,259	3,16399E-70	3
Col20a1	3,31977E-74	1,429638754	0,655	0,248	5,32724E-70	3
Pmp22	5,31623E-74	1,291914676	0,85	0,533	8,53095E-70	3
Rpl13	1,59494E-72	0,520940249	1	1	2,55941E-68	3
Anxa5	4,79467E-71	1,151052711	0,875	0,556	7,69401E-67	3
Cd81	1,64637E-69	0,692603534	1	0,981	2,64193E-65	3
Lmo4	1,91705E-69	1,191557586	0,793	0,402	3,07629E-65	3
Ppic	1,11258E-68	1,274490091	0,755	0,356	1,78536E-64	3
Myl9	1,2591E-68	1,510142119	0,634	0,271	2,02048E-64	3
Pdlim4	1,72114E-67	1,654648221	0,534	0,192	2,76192E-63	3
Gas7	3,00323E-67	1,130270603	0,836	0,414	4,81928E-63	3
Ednrb	3,85877E-66	1,297769544	0,809	0,487	6,19217E-62	3
Sox10	2,04472E-65	1,194738606	0,789	0,401	3,28117E-61	3
Plekhb1	1,6577E-64	0,994635575	0,88	0,464	2,6601E-60	3
Capg	6,87336E-63	2,122200611	0,341	0,089	1,10297E-58	3
Timp3	2,32213E-62	1,103304308	0,832	0,477	3,72633E-58	3
Cnn3	2,63066E-61	0,893134917	0,925	0,746	4,22142E-57	3
Rps27	4,91748E-61	0,504151426	1	1	7,89109E-57	3
Apoe	7,09818E-61	0,414691161	0,991	0,592	1,13904E-56	3
Lama4	1,18558E-60	1,695803952	0,448	0,15	1,9025E-56	3
Bsg	5,47384E-59	0,744101115	0,984	0,94	8,78387E-55	3
Prex2	1,02414E-57	2,179916634	0,282	0,066	1,64343E-53	3
Sfrp5	1,89389E-57	1,85170374	0,389	0,118	3,03913E-53	3
Olfml3	2,4208E-57	1,060269174	0,741	0,358	3,88466E-53	3
Prnp	2,60252E-57	1,000484995	0,941	0,837	4,17626E-53	3
Ccnd1	8,51664E-57	1,43652093	0,541	0,214	1,36667E-52	3
Cald1	4,82716E-56	1,306535713	0,723	0,416	7,74614E-52	3
Fkbp9	7,15517E-56	1,615445634	0,523	0,219	1,14819E-51	3
Lhfp	3,26149E-55	2,111320009	0,28	0,068	5,23372E-51	3
Taldo1	4,86516E-55	1,25325958	0,768	0,59	7,80713E-51	3
Gulp1	2,31922E-54	1,356265186	0,555	0,224	3,72165E-50	3
Sorbs2	1,60218E-53	1,664192909	0,5	0,21	2,57102E-49	3
Rps14	5,08735E-53	0,455516381	1	0,999	8,16367E-49	3

Tax1bp3	9,53067E-53	1,241074144	0,693	0,394	1,52939E-48	3
Rpl37a	1,94397E-52	0,395778609	1	1	3,11949E-48	3
Gpr37l1	2,00142E-52	0,904578343	0,843	0,48	3,21167E-48	3
Rpl13a	2,24057E-52	0,452480592	1	1	3,59544E-48	3
Ltbp1	4,14027E-52	1,881046007	0,311	0,087	6,64389E-48	3
Lamb1	4,42716E-52	1,760879255	0,357	0,111	7,10426E-48	3
Ctgf	4,84404E-52	1,302714493	0,648	0,328	7,77324E-48	3
Gfra2	1,15395E-51	1,314158018	0,67	0,383	1,85175E-47	3
Sema3b	2,10087E-51	1,46830762	0,514	0,219	3,37127E-47	3
Laptm4a	2,32824E-51	0,740075414	0,957	0,862	3,73612E-47	3
Lrrtm1	4,3946E-51	1,58109179	0,484	0,198	7,05202E-47	3
Metrn	8,57211E-50	0,960693921	0,784	0,417	1,37557E-45	3
Rhoc	9,74472E-50	0,99655597	0,845	0,649	1,56374E-45	3
Atp1a2	1,37872E-49	1,018041657	0,67	0,309	2,21244E-45	3
Ckb	2,86498E-49	0,818301464	0,909	0,709	4,59744E-45	3
Rpl23a	3,21656E-49	0,432030096	1	0,999	5,16162E-45	3
Slitrk6	8,77556E-49	1,528360681	0,441	0,168	1,40821E-44	3
Col3a1	9,89796E-49	1,099416397	0,611	0,287	1,58833E-44	3
Rps16	1,33187E-48	0,436584065	1	0,999	2,13725E-44	3
Rpl35	2,14673E-48	0,4245185	1	0,998	3,44485E-44	3
Rpl35a	3,24699E-48	0,445994181	1	0,999	5,21045E-44	3
Gpx8	5,89705E-48	1,259790187	0,559	0,25	9,46299E-44	3
Sepp1	9,99257E-48	1,193884067	0,68	0,366	1,60351E-43	3
Nell2	3,5259E-47	1,073805084	0,752	0,475	5,65801E-43	3
Afap1l2	1,00181E-46	1,370580485	0,525	0,231	1,6076E-42	3
Rpl36	3,42186E-46	0,418440671	1	0,999	5,49106E-42	3
Vgll3	3,93399E-46	1,880152787	0,339	0,111	6,31288E-42	3
Rps29	1,0937E-45	0,35275872	1	1	1,75505E-41	3
Rps4x	1,19471E-45	0,417461154	1	1	1,91715E-41	3
Tac1	0	3,309746079	0,971	0,107	0	4
Calb2	0	3,492154261	0,998	0,196	0	4
Dmkn	0	4,185782199	0,89	0,09	0	4
Rgs4	0	3,542291724	1	0,227	0	4
Oprk1	0	4,031330237	0,809	0,059	0	4
Penk	0	3,591590792	0,804	0,065	0	4
Aqp1	0	3,931063777	0,784	0,062	0	4
Gch1	0	3,771515524	0,797	0,081	0	4
Brinp2	0	3,551283185	0,76	0,068	0	4
Sncb	0	3,441804477	0,743	0,078	0	4
Necab2	0	3,437449323	0,728	0,08	0	4
Prmt8	0	4,042388792	0,581	0,034	0	4
Syt6	0	4,00417166	0,554	0,033	0	4
Ngef	0	4,346664843	0,542	0,026	0	4
Chgb	2,2891E-298	3,158307217	0,988	0,262	3,6734E-294	4
Ndufa4l2	5,848E-271	2,51402373	0,88	0,152	9,3843E-267	4
Rab3b	1,2644E-269	2,874175424	0,814	0,126	2,0291E-265	4
Tm4sf4	5,0347E-246	2,992848444	0,89	0,202	8,0791E-242	4
Slc18a3	8,6128E-246	2,481910707	0,978	0,251	1,3821E-241	4

Prkcb	5,1843E-241	2,737291197	0,787	0,125	8,3193E-237	4
Pi15	1,2283E-230	5,160175007	0,26	0,006	1,9711E-226	4
Tm4sf1	3,8969E-222	2,475411059	0,946	0,263	6,2533E-218	4
Snap25	3,304E-220	2,235519361	1	0,481	5,302E-216	4
Plcxd3	8,0873E-216	2,745930848	0,745	0,128	1,2978E-211	4
Hoxa5	1,0321E-215	2,330100408	1	0,632	1,6562E-211	4
Sez6	5,796E-215	3,195902814	0,534	0,059	9,3009E-211	4
Ffar3	1,3168E-213	3,462394237	0,449	0,039	2,113E-209	4
Tpd52l1	1,4978E-210	3,10993824	0,574	0,072	2,4036E-206	4
Plpp4	1,7445E-210	4,416604982	0,297	0,012	2,7995E-206	4
3110079O15Rik	1,5733E-209	4,239560041	0,292	0,012	2,5247E-205	4
Chrm2	6,9845E-206	3,537931879	0,414	0,034	1,1208E-201	4
Slc5a7	1,1534E-199	2,560663935	0,784	0,16	1,8508E-195	4
Grik1	6,1607E-199	4,04211499	0,343	0,022	9,886E-195	4
E530001K10Rik	1,7019E-197	2,575426438	0,797	0,174	2,7311E-193	4
Atp1b1	1,9761E-190	2,103157653	0,953	0,312	3,171E-186	4
Hoxb5	1,0684E-187	2,026971471	0,995	0,509	1,7144E-183	4
Cpne8	1,3643E-187	2,73948855	0,566	0,077	2,1893E-183	4
Ly6h	2,1253E-185	2,032776328	0,983	0,39	3,4105E-181	4
Scg2	4,3177E-183	1,956855659	0,998	0,457	6,9286E-179	4
Fxyd7	1,1328E-179	1,888237484	0,998	0,456	1,8178E-175	4
Nap1l5	2,4133E-175	2,104737073	0,912	0,28	3,8726E-171	4
Plxdc1	1,7554E-173	3,74046541	0,324	0,023	2,817E-169	4
Ddah1	9,5862E-172	2,030676992	0,757	0,153	1,5383E-167	4
Elmo1	3,6011E-171	2,757977879	0,527	0,073	5,7787E-167	4
Tmem59l	7,3851E-171	2,512624276	0,586	0,091	1,1851E-166	4
Pcsk2	6,1965E-170	2,109472226	0,882	0,24	9,9435E-166	4
Lrrfip1	9,6632E-167	1,974160603	0,951	0,341	1,5507E-162	4
Hmx2	8,5776E-165	2,285923732	0,777	0,179	1,3764E-160	4
Slc10a4	1,2248E-164	1,780791974	0,971	0,314	1,9654E-160	4
Tox	1,4668E-164	2,307858013	0,75	0,16	2,3538E-160	4
Zcchc12	1,5187E-164	1,943569052	0,931	0,301	2,4371E-160	4
Chst1	3,4601E-164	3,20149218	0,417	0,046	5,5525E-160	4
Nxph4	3,6117E-162	2,046167555	0,784	0,171	5,7957E-158	4
Hdac11	5,2744E-161	2,348752047	0,733	0,157	8,4639E-157	4
Asic4	7,2523E-160	2,509479277	0,551	0,084	1,1638E-155	4
Satb1	7,4824E-156	2,365716652	0,713	0,156	1,2007E-151	4
Lrrtm3	6,3107E-154	2,626451965	0,532	0,085	1,0127E-149	4
Pgm5	2,0595E-152	3,932916023	0,252	0,015	3,3049E-148	4
Anxa3	1,1967E-151	3,093184853	0,412	0,05	1,9204E-147	4
Agtr1a	1,0237E-144	2,912540866	0,417	0,053	1,6427E-140	4
Parvb	1,8259E-144	2,477853564	0,608	0,119	2,93E-140	4
Mab21l2	5,7769E-144	2,227822468	0,76	0,194	9,2701E-140	4
Pdlim3	1,9887E-143	3,579769285	0,297	0,025	3,1912E-139	4
Lgi1	1,7209E-142	2,404443563	0,542	0,092	2,7615E-138	4
Dner	3,3924E-142	2,206996752	0,635	0,127	5,4438E-138	4
Rasl11a	6,4726E-141	2,349654515	0,652	0,143	1,0387E-136	4
Bub3	3,8819E-138	1,486519695	0,998	0,616	6,2293E-134	4

Ndst4	1,4681E-136	2,272151904	0,569	0,104	2,3558E-132	4
Plppr5	6,2962E-135	2,002818523	0,73	0,18	1,0104E-130	4
Calm1	5,1431E-133	1,236390119	1	0,924	8,2531E-129	4
Rasd2	6,7613E-133	2,572953472	0,466	0,073	1,085E-128	4
Vamp1	1,1829E-132	2,419672577	0,551	0,105	1,8982E-128	4
Casz1	1,5148E-132	2,237409375	0,564	0,106	2,4308E-128	4
Celf4	2,547E-132	1,554974654	0,956	0,37	4,0872E-128	4
Rbfox1	4,9813E-132	2,023288168	0,674	0,153	7,9936E-128	4
Gm2694	2,8746E-131	2,768301342	0,316	0,032	4,6128E-127	4
Caly	1,0328E-130	1,870409564	0,799	0,225	1,6574E-126	4
Eef1a2	2,1005E-130	1,746154699	0,887	0,3	3,3707E-126	4
Ina	3,1401E-130	1,927131253	0,836	0,264	5,0389E-126	4
Tmem158	3,656E-130	1,938056159	0,855	0,284	5,8668E-126	4
Pfkfb3	6,0466E-130	3,081108419	0,321	0,034	9,703E-126	4
Galnt9	1,5222E-129	3,314638753	0,306	0,031	2,4427E-125	4
Kcns3	1,9621E-129	3,321411145	0,297	0,029	3,1486E-125	4
Colq	6,0715E-129	3,34808885	0,292	0,027	9,7429E-125	4
Stmn2	2,3751E-128	1,462039181	1	0,679	3,8113E-124	4
Cxcl12	5,617E-128	2,833177153	0,358	0,044	9,0136E-124	4
Pld5	3,7659E-127	2,704902266	0,419	0,063	6,0432E-123	4
Adgre1	4,8603E-127	1,984881333	0,735	0,205	7,7993E-123	4
St3gal6	2,8728E-126	2,341162834	0,466	0,076	4,61E-122	4
Faim2	1,5543E-124	2,298408432	0,475	0,08	2,4942E-120	4
Tns3	2,4257E-124	1,912807925	0,811	0,242	3,8925E-120	4
Gpr22	3,5179E-124	1,862319694	0,804	0,239	5,6452E-120	4
Cpne5	3,9216E-124	2,763583204	0,412	0,062	6,293E-120	4
Cplx2	1,3305E-123	2,054182439	0,681	0,161	2,135E-119	4
Dpp6	1,6568E-123	2,347196492	0,502	0,092	2,6587E-119	4
Bnc2	3,6169E-122	2,025496808	0,593	0,122	5,8041E-118	4
Aldoa	2,4617E-120	1,24745261	0,995	0,741	3,9503E-116	4
Nrsn1	3,0856E-120	1,879746093	0,792	0,245	4,9515E-116	4
Caln1	1,3264E-119	3,043407085	0,301	0,033	2,1285E-115	4
Rtn1	3,189E-119	1,359065643	1	0,681	5,1174E-115	4
Cnga3	3,4436E-119	3,070182765	0,282	0,028	5,526E-115	4
Ndufa4l2	3,6033E-279	3,072098213	0,93	0,158	5,7822E-275	5
Asic4	1,5307E-234	3,261660926	0,651	0,084	2,4564E-230	5
Rgs4	5,7211E-223	2,62043504	0,968	0,238	9,1806E-219	5
Nxph4	5,9719E-209	2,557879764	0,848	0,175	9,5832E-205	5
Prkcb	1,7047E-181	2,658426183	0,713	0,138	2,7356E-177	5
Chgb	6,213E-175	2,272834963	0,938	0,274	9,97E-171	5
Slc10a4	4,1423E-164	2,027931391	0,971	0,322	6,6471E-160	5
Oprk1	1,6932E-163	2,621500343	0,563	0,084	2,7171E-159	5
Pdzrn3	2,3933E-146	2,445619257	0,707	0,16	3,8405E-142	5
Bnc2	3,4618E-144	2,486441577	0,622	0,126	5,5551E-140	5
Dlx5	2,5851E-141	3,244734086	0,396	0,046	4,1483E-137	5
Phox2a	2,3453E-137	1,824123669	0,95	0,359	3,7635E-133	5
Nptx2	3,0527E-134	3,09775376	0,419	0,056	4,8987E-130	5
Gpr22	4,3237E-134	2,026120674	0,821	0,245	6,9383E-130	5

Hmx2	4,7036E-129	2,156944332	0,724	0,19	7,5479E-125	5
Hoxa5	3,1201E-123	1,765536344	0,974	0,639	5,0068E-119	5
Pcsk2	9,2324E-123	1,863870173	0,818	0,252	1,4815E-118	5
Slc18a3	1,4182E-122	1,743121877	0,871	0,267	2,2759E-118	5
Ptma	7,3796E-119	0,806897805	1	1	1,1842E-114	5
Nt5dc2	8,1351E-113	1,915838675	0,856	0,321	1,3054E-108	5
Snap25	1,4716E-107	1,459617985	0,991	0,488	2,3614E-103	5
Plcxd3	2,5719E-106	2,155342595	0,589	0,146	4,1271E-102	5
Bub3	4,0949E-106	1,424231811	0,982	0,622	6,5711E-102	5
Nfix	1,0901E-105	1,511058343	0,968	0,475	1,7492E-101	5
Satb1	4,5767E-104	1,992792991	0,642	0,168	7,3442E-100	5
Arhgef28	4,2212E-101	1,871843038	0,707	0,223	6,77376E-97	5
Syt17	8,82552E-95	1,90944565	0,672	0,209	1,41623E-90	5
Casz1	3,47042E-94	2,175525395	0,499	0,115	5,56898E-90	5
Fgf13	1,86567E-91	1,207842798	0,994	0,527	2,99384E-87	5
Hmx3	1,18691E-90	2,009461518	0,595	0,169	1,90464E-86	5
Il11ra1	1,21608E-89	1,651956614	0,815	0,311	1,95144E-85	5
Mab21l2	1,23038E-87	1,795352687	0,657	0,208	1,97439E-83	5
Brinp2	2,08966E-86	2,257625904	0,443	0,097	3,35328E-82	5
Dmkn	1,36958E-84	1,866895343	0,51	0,124	2,19776E-80	5
Elavl4	5,785E-82	1,206104514	0,988	0,52	9,28319E-78	5
Stard10	1,02172E-80	1,606987085	0,739	0,272	1,63955E-76	5
Tns3	4,2182E-80	1,599800348	0,718	0,255	6,76895E-76	5
Ndst4	6,19145E-79	2,137668422	0,46	0,117	9,93542E-75	5
Sez6	7,27634E-79	2,369386122	0,37	0,075	1,16763E-74	5
Rgmb	2,57955E-77	1,334597483	0,909	0,459	4,13941E-73	5
Rasl11a	7,13252E-77	1,932059333	0,537	0,156	1,14456E-72	5
Nfib	4,41394E-76	1,300222248	0,927	0,508	7,08305E-72	5
Aprt	2,29353E-75	1,444538892	0,827	0,34	3,68043E-71	5
Sh3gl3	5,46239E-74	1,596191235	0,66	0,229	8,7655E-70	5
Elmo1	1,2859E-72	2,312449847	0,387	0,088	2,06349E-68	5
Hoxa3	2,22478E-72	1,814839179	0,557	0,174	3,57011E-68	5
Ina	4,1666E-72	1,428799732	0,736	0,277	6,68615E-68	5
Basp1	9,95098E-72	1,207978149	0,938	0,432	1,59683E-67	5
Tshz2	2,40093E-70	1,244854178	0,924	0,467	3,85277E-66	5
Crmp1	2,77387E-67	1,21431626	0,833	0,35	4,45122E-63	5
Stmn4	3,35179E-67	1,216829795	0,871	0,374	5,37862E-63	5
Pde2a	4,91589E-67	1,690947411	0,548	0,177	7,88853E-63	5
Hist3h2ba	3,21954E-66	1,40036071	0,777	0,337	5,16639E-62	5
Eef2	9,47811E-66	0,621938996	1	0,996	1,52095E-61	5
Cacna2d1	1,54508E-65	1,435674621	0,713	0,288	2,47939E-61	5
Smpd3	2,4985E-65	1,082373436	0,868	0,372	4,00934E-61	5
Chga	8,90585E-65	1,32823416	0,716	0,275	1,42912E-60	5
Rab3b	9,70784E-63	1,547092943	0,507	0,154	1,55782E-58	5
Hoxb5	1,02046E-62	1,169584936	0,921	0,52	1,63754E-58	5
Elavl3	2,13754E-61	1,235353959	0,765	0,321	3,43012E-57	5
Slit2	2,69526E-61	1,565824064	0,548	0,184	4,32508E-57	5
Sox11	3,71747E-61	1,220400868	0,762	0,317	5,96542E-57	5

Snrk	4,84151E-61	1,377462507	0,733	0,302	7,76916E-57	5
Calb2	1,60461E-60	0,801366021	0,66	0,228	2,57492E-56	5
Sulf2	3,90396E-60	1,751802566	0,472	0,143	6,26469E-56	5
Nrsn1	4,78722E-60	1,254365109	0,672	0,259	7,68205E-56	5
Dusp26	3,19499E-59	1,1502345	0,85	0,391	5,127E-55	5
Akap13	5,33645E-59	1,298304217	0,754	0,322	8,56341E-55	5
Hdac11	2,20498E-58	1,750945467	0,519	0,177	3,53832E-54	5
Cmpk2	1,68109E-56	1,992401983	0,381	0,103	2,69764E-52	5
Rundc3a	3,1076E-56	1,216215908	0,786	0,364	4,98677E-52	5
Nrep	1,8065E-54	1,088104985	0,894	0,513	2,89888E-50	5
Fam171b	2,35358E-54	1,436240895	0,587	0,225	3,77679E-50	5
Mxra7	3,11244E-54	1,360180472	0,657	0,27	4,99454E-50	5
Tmem158	4,08073E-54	1,182614692	0,718	0,3	6,54835E-50	5
Rgs3	1,59333E-53	1,156405428	0,765	0,328	2,55682E-49	5
Asl	4,09234E-53	1,080758876	0,721	0,304	6,56699E-49	5
Nrxn2	6,74627E-53	1,254362687	0,642	0,258	1,08257E-48	5
Rps24	7,97667E-53	0,483834136	1	0,999	1,28002E-48	5
Lrrfip1	4,03198E-52	1,113682465	0,789	0,359	6,47012E-48	5
Slitrk5	6,14697E-52	1,600492762	0,487	0,158	9,86404E-48	5
Tox	1,22457E-51	1,511182546	0,525	0,181	1,96506E-47	5
Ly6h	1,52802E-51	0,940388179	0,853	0,405	2,45201E-47	5
Ramp1	2,07441E-51	0,990772474	0,877	0,439	3,32881E-47	5
E530001K10Rik	2,19386E-51	1,584424824	0,531	0,198	3,52048E-47	5
Vstm2l	1,7106E-50	1,240254349	0,604	0,238	2,745E-46	5
Meis1	5,48425E-50	1,640385954	0,46	0,151	8,80057E-46	5
Stmn2	8,4582E-50	0,912298768	0,997	0,684	1,35729E-45	5
Tagln3	9,90218E-50	0,861567547	0,818	0,365	1,589E-45	5
Pde1b	2,16788E-49	2,316270701	0,255	0,054	3,4788E-45	5
Pth1r	2,9424E-49	1,786671724	0,425	0,141	4,72167E-45	5
Mfng	2,71881E-48	1,361785403	0,493	0,175	4,36288E-44	5
Cpne8	9,86112E-48	1,81331901	0,346	0,097	1,58241E-43	5
Fcho1	1,05591E-47	1,955555206	0,349	0,1	1,69442E-43	5
Klhl32	1,17839E-47	1,921818311	0,349	0,099	1,89096E-43	5
Rab3a	1,74982E-47	0,862076212	0,918	0,467	2,80794E-43	5
Kif5c	1,82343E-47	1,2166659	0,654	0,29	2,92606E-43	5
Hap1	1,99305E-47	1,169379146	0,686	0,302	3,19824E-43	5
Fxyd7	9,68326E-47	0,839869268	0,891	0,47	1,55387E-42	5
Cadm1	1,25565E-46	0,794017089	0,977	0,737	2,01495E-42	5
Ptgds	2,41471E-46	1,927448714	0,337	0,095	3,87488E-42	5
Scgn	0	4,799529456	0,985	0,084	0	6
Moxd1	0	5,311446504	0,942	0,045	0	6
Npy	0	5,805618979	0,982	0,161	0	6
Cidea	0	3,871536711	0,902	0,096	0	6
Hpca	0	3,794922065	0,744	0,074	0	6
Kcnd2	0	3,810340722	0,646	0,048	0	6
Gpr149	0	4,530822046	0,576	0,026	0	6
Cpne4	0	3,912253258	0,54	0,031	0	6
Prokr1	0	4,58491498	0,503	0,022	0	6

Crabp1	0	5,978128143	0,488	0,013	0	6
Dlk1	0	5,524261336	0,482	0,016	0	6
Th	0	7,105431621	0,418	0,005	0	6
Npr1	0	5,298698092	0,418	0,011	0	6
Fibcd1	7,6401E-300	4,150045789	0,521	0,03	1,226E-295	6
Vip	3,0802E-290	5,571662606	0,997	0,275	4,9427E-286	6
Tcerg1l	5,5138E-286	4,756697846	0,405	0,015	8,848E-282	6
Etv1	7,8236E-286	2,799778164	0,988	0,172	1,2555E-281	6
Camk2a	7,6275E-284	4,187254698	0,494	0,028	1,224E-279	6
Dbh	2,8616E-281	4,468650465	0,686	0,074	4,592E-277	6
Npy2r	2,5508E-276	3,594581712	0,637	0,058	4,0932E-272	6
Syndig1	4,588E-268	3,262711215	0,768	0,098	7,3624E-264	6
Adora2a	3,7676E-266	4,295246821	0,451	0,024	6,0458E-262	6
Ccdc80	1,4613E-261	4,97697524	0,357	0,012	2,345E-257	6
9530059O14Rik	2,8233E-261	3,278856146	0,68	0,074	4,5305E-257	6
F2r	1,2535E-256	3,501358986	0,753	0,101	2,0115E-252	6
Hspb8	1,3731E-241	2,842404216	0,924	0,184	2,2035E-237	6
Tspan12	1,2185E-238	3,691403662	0,5	0,038	1,9553E-234	6
Ptger4	2,9504E-238	3,889455594	0,491	0,037	4,7346E-234	6
Oas1a	4,9282E-229	3,619568444	0,476	0,035	7,9083E-225	6
Tbx3os2	7,1005E-229	5,159617614	0,296	0,009	1,1394E-224	6
Tspan13	1,0888E-228	2,924685176	0,982	0,286	1,7472E-224	6
Spock1	2,3794E-224	3,782079656	0,476	0,037	3,8182E-220	6
Serpini1	1,066E-219	2,148352768	0,784	0,127	1,7106E-215	6
1500009L16Rik	2,6998E-218	2,997536903	0,848	0,167	4,3324E-214	6
Rit2	5,5234E-218	3,405187104	0,604	0,071	8,8634E-214	6
Spock3	8,7675E-213	2,777852828	0,951	0,263	1,4069E-208	6
Insm1	1,2427E-208	3,321381835	0,591	0,07	1,9941E-204	6
Spint1	1,2907E-208	4,674352135	0,299	0,011	2,0712E-204	6
Sstr1	7,5557E-203	3,218615746	0,671	0,101	1,2125E-198	6
Tlx2	1,5551E-202	2,377275325	0,988	0,275	2,4955E-198	6
Astn2	1,9758E-198	3,819197602	0,399	0,028	3,1705E-194	6
Syn2	3,7384E-198	2,635298704	0,988	0,334	5,999E-194	6
Ptpre	3,2618E-195	4,224659276	0,317	0,015	5,2342E-191	6
Psmb8	6,8747E-194	3,457688514	0,473	0,043	1,1032E-189	6
Fst	1,5807E-193	2,89899891	0,723	0,121	2,5365E-189	6
Pxylp1	1,7006E-191	2,792172305	0,805	0,166	2,729E-187	6
Tgfb1	4,6563E-189	2,823601222	0,747	0,134	7,4719E-185	6
Snca	7,5601E-188	2,531817882	0,921	0,249	1,2132E-183	6
Resp18	3,7425E-183	2,217615387	0,915	0,227	6,0057E-179	6
Tmem255b	5,1451E-182	2,511027682	0,884	0,224	8,2563E-178	6
Chst8	3,8952E-177	3,233157633	0,448	0,043	6,2506E-173	6
Syt1	2,8348E-176	2,2065268	0,994	0,4	4,5491E-172	6
Kcnv1	1,0008E-174	3,926385832	0,305	0,017	1,606E-170	6
Cntnap5a	1,945E-167	2,924944242	0,582	0,087	3,1211E-163	6
Tmem150c	8,1664E-167	3,370486952	0,396	0,035	1,3105E-162	6
Myl1	8,8196E-165	2,007658573	0,97	0,311	1,4153E-160	6
Ptprr	1,8382E-164	2,666790355	0,662	0,116	2,9497E-160	6

Sertm1	5,4363E-163	3,9229849	0,311	0,02	8,7236E-159	6
Socs2	6,0193E-163	2,361101161	0,899	0,267	9,6593E-159	6
Oas1d	5,8158E-157	3,232864482	0,363	0,031	9,3326E-153	6
Kif26a	6,4904E-155	2,900268245	0,491	0,062	1,0415E-150	6
Gap43	8,4268E-153	1,821211736	1	0,704	1,3522E-148	6
Thy1	1,6614E-152	2,664311697	0,482	0,061	2,6661E-148	6
Uchl1	2,1498E-152	1,774111318	1	0,682	3,4499E-148	6
Gm13889	4,5315E-152	2,481691847	0,677	0,128	7,2717E-148	6
Tmod1	3,467E-151	2,243315973	0,713	0,142	5,5635E-147	6
A730017C20Rik	5,434E-151	2,351155475	0,701	0,141	8,7199E-147	6
Arpp21	1,1299E-147	2,477034437	0,698	0,152	1,8131E-143	6
P2rx2	6,0429E-147	2,614973411	0,619	0,113	9,6971E-143	6
Mgll	6,5794E-146	2,805201141	0,549	0,085	1,0558E-141	6
Ano2	3,5195E-145	3,356692413	0,326	0,027	5,6478E-141	6
Entpd3	1,9322E-143	3,053086182	0,43	0,052	3,1005E-139	6
Parm1	2,9787E-139	2,695634097	0,524	0,081	4,7799E-135	6
Cacng3	5,0311E-139	4,139378466	0,256	0,016	8,0734E-135	6
Calm2	4,1007E-138	1,335642752	1	0,978	6,5804E-134	6
Nrp1	4,9874E-138	2,040141178	0,89	0,275	8,0033E-134	6
Mllt11	3,9717E-136	1,785747265	0,991	0,488	6,3733E-132	6
Cd24a	1,9599E-135	1,712997148	0,997	0,667	3,145E-131	6
Pgf	6,1142E-134	3,007316492	0,454	0,063	9,8114E-130	6
Pwwp2b	2,2563E-133	3,558380852	0,302	0,025	3,6207E-129	6
Tmem56	3,0446E-133	3,810425001	0,287	0,022	4,8857E-129	6
S100a1	4,4711E-132	1,936415845	0,939	0,342	7,1748E-128	6
Bex2	4,8959E-132	1,513966203	1	0,67	7,8565E-128	6
Pirt	7,5893E-130	1,819260721	0,902	0,275	1,2179E-125	6
Fam159a	5,199E-128	3,615129549	0,28	0,022	8,3429E-124	6
Fhod3	7,478E-127	1,932423398	0,841	0,254	1,2E-122	6
Tubb3	5,0775E-126	1,681573163	1	0,565	8,1479E-122	6
S100a13	1,481E-124	1,786869665	0,945	0,353	2,3766E-120	6
Syt5	6,8055E-124	2,020437677	0,793	0,224	1,0921E-119	6
Rph3a	1,9643E-123	2,74738008	0,412	0,054	3,1521E-119	6
Mirg	2,4137E-123	3,154630759	0,357	0,041	3,8733E-119	6
Nnat	4,8874E-122	2,031711189	0,957	0,512	7,8428E-118	6
Slc36a1	5,1186E-122	2,990190389	0,369	0,044	8,2139E-118	6
Ret	2,1017E-121	1,944270557	0,759	0,198	3,3726E-117	6
Ywhah	3,7273E-121	1,438311306	1	0,757	5,9812E-117	6
Pcbp3	9,7099E-121	1,698622347	0,973	0,423	1,5581E-116	6
Fam163b	1,0206E-120	3,447161761	0,28	0,024	1,6378E-116	6
Stmn3	2,0501E-120	1,523198882	1	0,564	3,2898E-116	6
Maoa	9,7415E-118	2,322179324	0,665	0,163	1,5632E-113	6
Cnr1	6,4799E-117	1,969222596	0,72	0,185	1,0398E-112	6
Rgs7bp	8,7314E-117	3,095908818	0,338	0,039	1,4011E-112	6
Myl1	2,4862E-179	2,523373915	0,997	0,315	3,9896E-175	7
Tlx2	8,5493E-168	2,359494116	0,972	0,281	1,3719E-163	7
Phox2a	1,0222E-137	2,062494901	0,972	0,364	1,6403E-133	7
Hspb8	2,3222E-120	1,964825285	0,797	0,196	3,7264E-116	7

Bnc2	8,0919E-119	2,422401868	0,626	0,131	1,2985E-114	7
Ptma	2,4869E-118	0,923414363	1	1	3,9907E-114	7
Htr3a	2,8586E-116	2,319134874	0,769	0,208	4,5871E-112	7
Erich2	1,3088E-111	4,048695458	0,255	0,02	2,1002E-107	7
Fgf13	1,6166E-108	1,778843335	0,972	0,533	2,5942E-104	7
Slc18a3	2,192E-102	1,657850074	0,892	0,272	3,51755E-98	7
Id2	9,0783E-102	1,825698411	0,923	0,384	1,4568E-97	7
Tcf7l2	6,2406E-99	1,884776881	0,881	0,332	1,00143E-94	7
Celf3	1,53941E-98	1,808105912	0,836	0,278	2,47029E-94	7
Slc10a4	1,33257E-96	1,510603784	0,941	0,33	2,13837E-92	7
Plscr1	1,61306E-96	2,361653632	0,629	0,164	2,58848E-92	7
Kcnk2	1,13546E-95	2,169980657	0,689	0,195	1,82207E-91	7
Id1	5,33037E-92	1,882570516	0,699	0,193	8,55365E-88	7
Cct7	2,5316E-88	1,47745536	0,983	0,739	4,06246E-84	7
Riiad1	4,8028E-88	2,239494039	0,591	0,151	7,70706E-84	7
Cadm1	2,66324E-87	1,298125413	0,986	0,739	4,27371E-83	7
Pcgf1	3,03413E-84	1,939039569	0,685	0,204	4,86886E-80	7
Ckmt1	4,11894E-82	1,984784916	0,643	0,19	6,60967E-78	7
Rbfox1	2,94381E-79	2,184102764	0,591	0,169	4,72393E-75	7
Elavl3	1,11411E-78	1,592326592	0,843	0,321	1,7878E-74	7
Elavl4	1,61682E-77	1,377832066	0,986	0,524	2,59451E-73	7
Snap25	3,23071E-77	1,337574546	0,965	0,494	5,18433E-73	7
Sox11	1,95413E-76	1,593820706	0,839	0,317	3,13579E-72	7
Pcbp3	2,14266E-75	1,428268444	0,944	0,429	3,43832E-71	7
Hist3h2ba	4,72867E-75	1,633442669	0,846	0,338	7,5881E-71	7
Nt5dc2	5,14223E-75	1,563787359	0,836	0,328	8,25173E-71	7
Dcx	4,09932E-74	1,957990816	0,57	0,163	6,57818E-70	7
Ifitm2	7,25171E-73	1,019877467	0,997	0,895	1,16368E-68	7
Tubb3	2,41462E-71	1,205536534	0,99	0,569	3,87473E-67	7
Crmp1	8,3643E-70	1,47453336	0,843	0,354	1,34222E-65	7
Tuba1a	1,15124E-68	0,755448902	1	0,999	1,84739E-64	7
Dpysl3	3,9543E-67	1,038652992	0,993	0,672	6,34546E-63	7
Faah	4,43798E-67	2,70435095	0,336	0,063	7,12162E-63	7
Cited2	2,23764E-66	1,565739097	0,902	0,452	3,59074E-62	7
Nxph4	5,07619E-65	1,707293173	0,612	0,194	8,14576E-61	7
Basp1	8,88948E-65	1,347536646	0,923	0,438	1,4265E-60	7
Scx	9,11299E-65	2,175723818	0,441	0,11	1,46236E-60	7
A830018L16Rik	4,08462E-64	2,741588594	0,399	0,096	6,55459E-60	7
Ndufa4l2	1,56443E-63	1,437975461	0,615	0,182	2,51044E-59	7
Tubb2b	3,06411E-63	1,073398757	0,997	0,624	4,91697E-59	7
H3f3a	5,86128E-63	0,634796595	1	0,998	9,4056E-59	7
Fhod3	5,35472E-62	1,551896453	0,713	0,265	8,59272E-58	7
Gpr22	1,40601E-61	1,396170059	0,71	0,257	2,25623E-57	7
Slc4a4	2,89888E-61	2,615809464	0,297	0,053	4,65183E-57	7
Hmx3	1,44914E-60	1,716571614	0,559	0,176	2,32544E-56	7
Actg1	8,82384E-60	0,646858028	1	0,999	1,41596E-55	7
Arhgef28	1,01578E-58	1,617381459	0,64	0,231	1,63002E-54	7
Hoxc4	8,07597E-57	1,39957633	0,832	0,39	1,29595E-52	7

Tsc22d1	2,03965E-56	1,38838469	0,895	0,521	3,27303E-52	7
Klhl32	1,18048E-55	2,019469078	0,399	0,099	1,89431E-51	7
Syt1	1,3385E-55	1,149949844	0,899	0,409	2,14789E-51	7
Syt17	1,26946E-54	1,647007259	0,594	0,217	2,0371E-50	7
Bend5	2,18358E-54	1,692917898	0,566	0,191	3,504E-50	7
Chgb	2,58271E-54	0,76845669	0,78	0,289	4,14448E-50	7
Kif5c	3,72076E-54	1,350647297	0,731	0,29	5,9707E-50	7
Ppp2r2b	5,36919E-54	1,400588419	0,731	0,297	8,61593E-50	7
Gm2990	6,99294E-54	2,036913077	0,374	0,093	1,12216E-49	7
Ahcyl2	1,23456E-53	1,742986058	0,556	0,186	1,98111E-49	7
Plcxd3	1,65125E-53	1,81745318	0,497	0,155	2,64977E-49	7
Mapk10	2,11351E-52	1,318983704	0,682	0,259	3,39156E-48	7
Csrp2	4,22888E-51	1,496358814	0,608	0,218	6,78609E-47	7
Lix1	7,44261E-51	1,207376655	0,748	0,305	1,19432E-46	7
St3gal6	2,19021E-50	2,249314809	0,357	0,09	3,51463E-46	7
Uchl1	4,8584E-50	0,908149866	0,997	0,684	7,79628E-46	7
Gap43	5,3578E-50	0,851953129	0,986	0,707	8,59767E-46	7
Tmsb10	1,01804E-48	0,594311593	1	0,994	1,63365E-44	7
Tubb5	1,24585E-48	0,596228868	1	0,996	1,99921E-44	7
Eef2	1,3323E-48	0,614327074	1	0,996	2,13794E-44	7
Aplp1	2,39037E-48	0,99379951	0,885	0,411	3,83582E-44	7
Hmx2	3,72975E-48	1,529965701	0,566	0,204	5,98513E-44	7
Snca	4,07705E-48	1,115899473	0,675	0,267	6,54245E-44	7
Cacna2d1	4,48814E-48	1,234023762	0,713	0,292	7,20211E-44	7
Adrbk2	5,0773E-48	1,203926114	0,727	0,309	8,14755E-44	7
Tmem255b	5,63065E-48	1,170261144	0,65	0,241	9,03551E-44	7
Gcnt2	1,00443E-47	1,885894312	0,42	0,127	1,61182E-43	7
Dlx5	1,78396E-47	2,447689669	0,273	0,056	2,86272E-43	7
Stmn4	2,26196E-47	1,095529102	0,829	0,381	3,62977E-43	7
Tagln3	5,75075E-47	0,9468741	0,839	0,368	9,22823E-43	7
Rab3c	1,22289E-46	1,27432314	0,64	0,249	1,96237E-42	7
Syn2	2,90958E-46	0,832345613	0,811	0,348	4,66901E-42	7
Pde2a	1,8464E-45	1,376844011	0,528	0,182	2,96291E-41	7
Garnl3	2,92323E-45	1,224676221	0,703	0,291	4,6909E-41	7
Sh3gl3	3,08219E-45	1,373414121	0,615	0,235	4,94599E-41	7
Rundc3a	7,84476E-45	1,088260725	0,804	0,368	1,25885E-40	7
Gse1	5,71661E-44	1,585131529	0,434	0,133	9,17344E-40	7
H2afy2	6,17915E-44	1,206124186	0,762	0,336	9,91568E-40	7
App	7,38783E-44	0,747083446	0,986	0,86	1,18552E-39	7
Cidea	8,52752E-44	1,230368489	0,437	0,127	1,36841E-39	7
Frmd4b	1,43487E-43	1,785928272	0,378	0,106	2,30253E-39	7
Plod2	8,54266E-43	1,635398534	0,465	0,156	1,37084E-38	7
Tnpo1	1,17584E-42	1,517196333	0,549	0,205	1,88686E-38	7
Hoxa5	1,25822E-42	0,734923565	0,948	0,643	2,01907E-38	7
Prph	1,26438E-42	1,377768049	0,783	0,483	2,02895E-38	7
Cacnb3	1,01246E-41	1,318158836	0,58	0,225	1,62469E-37	7
Ptgds	1,52991E-41	1,854660236	0,35	0,097	2,45505E-37	7
Trp53i11	3,92657E-41	1,20460159	0,668	0,289	6,30097E-37	7

Zfp804a	9,05328E-41	1,945550709	0,339	0,095	1,45278E-36	7
Krt19	0	6,176148932	0,772	0,059	0	8
Ntrk3	0	4,079607687	0,747	0,055	0	8
Wif1	0	5,590048816	0,66	0,029	0	8
Sst	0	6,786591602	0,61	0,025	0	8
Edn1	2,7722E-241	5,646978625	0,34	0,01	4,4486E-237	8
Cbln2	8,2969E-241	5,472936824	0,614	0,052	1,3314E-236	8
6330403A02Rik	9,1904E-205	3,445783173	0,685	0,079	1,4748E-200	8
Calcb	1,9683E-203	5,034984315	0,427	0,026	3,1586E-199	8
Nog	1,4468E-201	5,290142417	0,432	0,027	2,3217E-197	8
Cidea	2,6404E-197	2,961273791	0,797	0,114	4,2371E-193	8
Htr3b	6,1011E-196	3,632482353	0,56	0,052	9,7904E-192	8
Rph3a	7,8623E-189	3,641502557	0,56	0,053	1,2617E-184	8
Myl1	1,4042E-178	3,021184636	1	0,32	2,2533E-174	8
Htr3a	8,8344E-178	2,969547957	0,934	0,205	1,4177E-173	8
P2rx2	9,2587E-178	3,408079041	0,751	0,115	1,4857E-173	8
Il13ra1	1,424E-173	3,75844373	0,436	0,033	2,2851E-169	8
Nrxn3	6,6082E-165	3,420282823	0,423	0,032	1,0604E-160	8
Fam19a1	3,8497E-158	2,688734924	0,718	0,112	6,1776E-154	8
Syn2	1,659E-155	2,604162714	0,996	0,344	2,6621E-151	8
Pgf	2,5029E-152	3,206525777	0,548	0,065	4,0163E-148	8
Prph	1,2292E-149	2,628047828	1	0,476	1,9725E-145	8
9530059O14Rik	2,1084E-148	3,298552976	0,61	0,087	3,3834E-144	8
Oas1d	1,394E-145	4,236630288	0,402	0,035	2,237E-141	8
Calb2	3,3337E-144	3,032915457	0,88	0,226	5,3495E-140	8
Id2	1,8679E-139	2,589243314	0,975	0,386	2,9974E-135	8
Pcdh10	2,7752E-139	3,401534188	0,519	0,064	4,4533E-135	8
Cnr1	4,5186E-137	2,773801671	0,817	0,189	7,2511E-133	8
Npy2r	1,9643E-136	2,867232831	0,548	0,071	3,1521E-132	8
Scube1	8,5234E-136	3,335946761	0,444	0,046	1,3677E-131	8
Fxyd7	9,3176E-136	2,298507525	1	0,473	1,4952E-131	8
Krt15	4,9915E-134	3,883114565	0,328	0,024	8,0098E-130	8
Oas1a	1,0753E-133	3,392694959	0,432	0,044	1,7256E-129	8
Scg2	4,6614E-132	2,646658595	1	0,474	7,4802E-128	8
Tlx2	1,2974E-130	2,169717677	0,971	0,287	2,0819E-126	8
Ano2	4,2204E-130	3,765154244	0,357	0,03	6,7724E-126	8
Hspb8	1,1272E-128	2,310645659	0,851	0,199	1,8088E-124	8
Anxa2	3,3167E-121	2,427024124	0,938	0,33	5,3222E-117	8
Fgf13	1,9476E-120	2,00867408	1	0,535	3,1254E-116	8
Robo2	1,2906E-119	2,958609299	0,469	0,058	2,071E-115	8
Rprm	3,7439E-119	2,846061406	0,51	0,072	6,0079E-115	8
Sncg	4,6173E-117	2,597653826	0,896	0,306	7,4094E-113	8
Gap43	2,1698E-115	1,876155277	1	0,709	3,482E-111	8
Cpne4	1,0181E-113	3,651620567	0,394	0,045	1,6337E-109	8
Plppr5	9,0368E-113	2,382160726	0,776	0,195	1,4501E-108	8
Cux2	4,3441E-112	3,109127079	0,519	0,079	6,971E-108	8
Ckmt1	3,3255E-110	2,30403281	0,768	0,188	5,3364E-106	8
Slc18a3	2,6695E-109	1,916166738	0,925	0,275	4,2838E-105	8

Plxna4	6,0849E-107	2,618581606	0,577	0,099	9,7645E-103	8
Ifitm2	7,0744E-107	1,526937976	1	0,896	1,1352E-102	8
Ddah1	1,3704E-105	2,306904969	0,734	0,173	2,1991E-101	8
Adgre1	1,4554E-104	2,377216048	0,788	0,219	2,3355E-100	8
Fabp3	1,8759E-104	2,539572079	0,614	0,124	3,0103E-100	8
Rbfox1	1,7491E-103	2,154492559	0,726	0,167	2,8069E-99	8
Zbtb7c	1,7814E-102	2,898717504	0,456	0,067	2,85866E-98	8
Stmn3	4,0302E-96	1,673669353	1	0,57	6,46726E-92	8
Syt2	1,98912E-94	3,394983397	0,299	0,03	3,19195E-90	8
Tcf7l2	2,91657E-94	1,967218759	0,892	0,336	4,68022E-90	8
Pxmp2	1,27351E-92	3,222953005	0,357	0,044	2,0436E-88	8
Rab3b	3,87897E-92	2,328433111	0,668	0,154	6,22459E-88	8
Uchl1	6,03783E-92	1,529532188	1	0,687	9,68891E-88	8
Pcsk1n	6,16015E-92	1,493704258	1	0,682	9,8852E-88	8
Necab1	7,01879E-91	2,613618689	0,527	0,1	1,12631E-86	8
Fdps	1,06055E-90	1,792171866	0,979	0,526	1,70186E-86	8
Snhg11	1,08723E-89	2,320238015	0,793	0,249	1,74468E-85	8
Cnih3	1,75799E-89	2,906499816	0,369	0,048	2,82105E-85	8
Klhdc8a	5,96147E-89	3,631643269	0,266	0,025	9,56638E-85	8
Fabp5	6,76651E-89	1,544075588	0,992	0,602	1,08582E-84	8
Scn11a	1,12291E-88	3,37616533	0,278	0,027	1,80194E-84	8
Ptprr	8,02575E-88	2,205074147	0,593	0,128	1,28789E-83	8
Tuba1a	2,42123E-87	1,134006859	1	0,999	3,88535E-83	8
Stmn2	4,66068E-86	1,511760158	1	0,689	7,47899E-82	8
Rtn1	3,98562E-85	1,463641224	1	0,69	6,39572E-81	8
Fhod3	9,12742E-85	1,832074	0,826	0,264	1,46468E-80	8
Trp53i11	1,53032E-84	1,949653309	0,838	0,284	2,4557E-80	8
Meg3	4,57382E-84	1,489681875	1	0,839	7,33961E-80	8
Coro2a	4,72444E-84	2,80063038	0,373	0,053	7,5813E-80	8
S100a10	7,00184E-84	1,57130253	0,979	0,652	1,12358E-79	8
Lrrc75b	2,4135E-83	2,714555677	0,415	0,066	3,87295E-79	8
Tubb2b	5,55161E-83	1,457293918	1	0,627	8,90866E-79	8
Ppp3ca	2,08499E-82	1,688434869	0,954	0,479	3,34578E-78	8
Gdf10	2,21437E-82	2,334505106	0,365	0,048	3,55339E-78	8
Dgkg	9,77825E-82	3,127895622	0,295	0,034	1,56912E-77	8
Cct7	3,49781E-81	1,423711232	0,983	0,741	5,61293E-77	8
Ache	5,45797E-81	1,707761134	0,859	0,29	8,75841E-77	8
Nos1ap	1,15409E-79	3,297087064	0,29	0,034	1,85196E-75	8
Syngr3	2,23324E-79	1,814286335	0,809	0,262	3,58369E-75	8
Tubb3	5,24455E-79	1,494892913	0,996	0,572	8,41593E-75	8
Robo1	5,64245E-79	2,805340357	0,344	0,047	9,05445E-75	8
March4	6,09965E-79	3,094528946	0,286	0,033	9,78811E-75	8
Pcbp3	6,97266E-79	1,652695941	0,934	0,434	1,1189E-74	8
Mllt11	1,85077E-78	1,493969821	0,988	0,496	2,96993E-74	8
Slc36a1	3,63452E-78	2,788945807	0,349	0,05	5,83232E-74	8
Csrp2	1,67596E-77	2,099555949	0,722	0,216	2,68941E-73	8
Faah	1,69659E-77	2,644597022	0,394	0,063	2,72252E-73	8
Gucy1a3	2,21545E-77	2,656507208	0,415	0,072	3,55513E-73	8

Fxyd6	3,8903E-76	1,329144749	0,996	0,594	6,24276E-72	8
Crmp1	4,68143E-76	1,663488025	0,9	0,356	7,51229E-72	8
Slc4a4	8,20178E-76	2,642080185	0,357	0,053	1,31614E-71	8
Rgs7bp	3,95028E-74	2,938390219	0,32	0,044	6,33901E-70	8
Casz1	1,11916E-73	1,984041821	0,548	0,12	1,79592E-69	8
Rac3	1,72801E-73	1,938231617	0,726	0,232	2,77293E-69	8
Nos1	0	5,728695432	0,992	0,062	0	9
Epha5	0	4,57601984	0,954	0,085	0	9
Rprml	0	4,197148706	0,917	0,061	0	9
Mfsd4	0	4,727209953	0,542	0,024	0	9
Stxbp6	0	4,956064137	0,508	0,018	0	9
Arhgap15	0	5,738704444	0,388	0,008	0	9
Cartpt	8,933E-288	6,199839702	0,929	0,13	1,4335E-283	9
Slc35d3	3,5922E-286	5,234425079	0,392	0,01	5,7644E-282	9
Ngb	3,1722E-253	3,99871671	0,679	0,06	5,0904E-249	9
Kitl	1,2857E-247	4,308694829	0,492	0,026	2,0632E-243	9
Kcnab1	2,3273E-237	4,308207018	0,479	0,026	3,7346E-233	9
Cygb	5,2904E-237	3,663812182	0,829	0,11	8,4896E-233	9
Cadps2	8,2088E-233	5,230803662	0,333	0,01	1,3173E-228	9
Gsg1l	3,8477E-222	4,943580967	0,354	0,013	6,1744E-218	9
Ass1	4,8594E-221	4,568375966	1	0,275	7,7978E-217	9
Gng8	2,0679E-220	3,897441782	0,588	0,049	3,3183E-216	9
Gjd2	4,1505E-199	4,166846108	0,404	0,022	6,6603E-195	9
Gal	1,6579E-195	2,792474704	1	0,297	2,6604E-191	9
Etv1	6,7027E-194	2,457869907	0,983	0,185	1,0756E-189	9
Gm5424	1,7333E-192	4,38878369	0,367	0,018	2,7815E-188	9
Tmod1	1,3436E-191	2,956724138	0,867	0,145	2,156E-187	9
Ptgir	3,3449E-190	4,718182989	0,321	0,013	5,3676E-186	9
Cpne7	1,7329E-179	4,570858143	0,312	0,013	2,7807E-175	9
Tesc	2,9707E-157	3,152535864	0,512	0,053	4,7671E-153	9
S100a4	6,721E-156	3,374498269	0,846	0,196	1,0785E-151	9
S100a13	2,4433E-155	2,801139621	0,996	0,36	3,9207E-151	9
P2ry6	1,8901E-153	3,206133536	0,537	0,061	3,033E-149	9
Ank2	1,173E-149	2,628647583	0,996	0,468	1,8823E-145	9
Cntnap5a	4,8562E-149	2,862143306	0,642	0,092	7,7927E-145	9
Entpd3	1,9102E-146	3,2413204	0,5	0,055	3,0653E-142	9
Fam155a	5,013E-146	3,070044345	0,608	0,085	8,0444E-142	9
S100a6	6,8732E-143	2,830441543	1	0,837	1,1029E-138	9
Khdrbs2	1,1542E-136	3,112333822	0,517	0,064	1,8521E-132	9
Plch2	5,7985E-134	4,011400374	0,292	0,018	9,3048E-130	9
Nxph3	1,7823E-133	3,129947344	0,488	0,057	2,86E-129	9
Smpd3	6,3976E-133	2,316960305	0,992	0,376	1,0266E-128	9
C1ql1	3,3094E-128	2,51093368	0,892	0,27	5,3105E-124	9
Resp18	6,915E-128	1,97231582	0,917	0,238	1,1096E-123	9
Celf4	1,1508E-127	2,134993884	0,996	0,386	1,8468E-123	9
Rnf152	7,5458E-126	3,731542488	0,325	0,025	1,2109E-121	9
Tmem108	1,1867E-124	2,928958953	0,504	0,066	1,9043E-120	9
Kcnq3	2,5658E-122	2,509064126	0,688	0,131	4,1174E-118	9

Pid1	1,079E-121	2,593687705	0,642	0,113	1,7315E-117	9
Pirt	2,3787E-121	2,207504662	0,925	0,284	3,8171E-117	9
Asl	4,2857E-120	2,333114635	0,917	0,303	6,8773E-116	9
Camp	6,3013E-119	2,877247879	0,608	0,106	1,0112E-114	9
Pde1c	1,4532E-118	3,713287492	0,304	0,023	2,332E-114	9
Kctd12	1,5419E-118	2,902717122	0,508	0,069	2,4742E-114	9
Adamts5	1,4496E-117	2,67493638	0,671	0,131	2,3262E-113	9
Kcnab2	2,0287E-117	3,295442072	0,433	0,052	3,2555E-113	9
Vip	1,8854E-113	1,466888964	0,917	0,29	3,0255E-109	9
Tmem150c	3,0462E-113	3,165785875	0,388	0,041	4,8883E-109	9
S100a1	4,102E-112	2,231042082	0,942	0,351	6,5824E-108	9
Actn1	1,4909E-109	2,238888975	0,746	0,167	2,3925E-105	9
Syt4	3,0763E-109	2,355938763	0,792	0,205	4,9365E-105	9
Gm13889	2,3292E-108	2,344909062	0,675	0,137	3,7377E-104	9
Fam13c	1,5747E-106	3,449581742	0,321	0,03	2,5269E-102	9
Hs3st2	1,995E-106	3,853603201	0,254	0,017	3,2013E-102	9
Unc13c	3,4631E-105	3,672007083	0,267	0,02	5,5572E-101	9
Popdc3	1,0464E-104	3,354549579	0,333	0,033	1,6791E-100	9
Crip1	1,2899E-102	1,829810495	1	0,873	2,06994E-98	9
Lix1	1,5639E-102	1,886507117	0,929	0,301	2,50963E-98	9
Dner	2,9804E-102	2,364907077	0,662	0,141	4,7827E-98	9
Dpysl3	5,0158E-101	1,703590682	1	0,674	8,04888E-97	9
Acot7	2,9053E-100	2,10209838	0,904	0,322	4,66211E-96	9
Tmem255b	1,3646E-99	2,144034635	0,829	0,237	2,18979E-95	9
Cox6c	1,7315E-99	1,21049556	1	0,968	2,7785E-95	9
Ly6h	1,8562E-99	1,792543423	0,971	0,408	2,97858E-95	9
Lrrfip1	2,5392E-99	1,900144937	0,946	0,36	4,07468E-95	9
Ret	6,4846E-99	2,247243109	0,762	0,206	1,04058E-94	9
Arl4a	7,43578E-98	2,125403955	0,875	0,284	1,19322E-93	9
Grid2	1,56001E-97	2,608431088	0,479	0,073	2,50335E-93	9
Ltk	1,71527E-96	3,235033709	0,304	0,03	2,75249E-92	9
Tspan13	1,98089E-96	1,741576714	0,921	0,3	3,17874E-92	9
Adgrb3	3,24659E-96	2,214787173	0,742	0,189	5,2098E-92	9
Kcnt2	3,51347E-96	3,001829983	0,375	0,047	5,63807E-92	9
Dgkb	6,37213E-96	3,502145047	0,283	0,026	1,02254E-91	9
Nsg2	1,38304E-95	2,038655633	0,929	0,366	2,21936E-91	9
Eef1a2	1,56392E-95	1,85723394	0,929	0,316	2,50963E-91	9
Bglap2	2,97752E-95	3,344649653	0,388	0,052	4,77803E-91	9
Chga	1,08682E-94	2,087218215	0,858	0,277	1,74403E-90	9
Clmp	2,93851E-94	2,678756659	0,462	0,073	4,71543E-90	9
Alcam	1,34679E-93	1,9929668	0,892	0,297	2,1612E-89	9
Bglap	1,53046E-93	3,201447609	0,458	0,075	2,45592E-89	9
Cntn1	3,54785E-93	2,312934801	0,637	0,14	5,69323E-89	9
Scg2	1,08752E-92	1,776230276	0,988	0,474	1,74514E-88	9
Stmn3	4,34875E-92	1,573391157	1	0,571	6,97844E-88	9
Fstl5	4,01105E-91	2,267414064	0,646	0,148	6,43654E-87	9
Atp1b1	1,26605E-89	1,8142792	0,921	0,333	2,03164E-85	9
Mtus2	1,60465E-89	2,488943004	0,521	0,098	2,57498E-85	9

Stom	1,57873E-88	2,417128077	0,608	0,134	2,53339E-84	9
Nrg1	1,79985E-88	3,107995667	0,317	0,036	2,88822E-84	9
Pcbd1	2,28658E-88	2,108591342	0,771	0,219	3,66927E-84	9
Ncald	2,25074E-87	2,035066892	0,833	0,27	3,61176E-83	9
Cnga3	2,5908E-87	3,254386875	0,308	0,035	4,15746E-83	9
Fxyd6	4,2575E-87	1,483834346	1	0,594	6,832E-83	9
Calm2	2,27896E-86	1,165194416	1	0,978	3,65705E-82	9
Dleu7	2,3812E-86	2,888344734	0,392	0,057	3,82111E-82	9
Tmem158	4,9552E-85	1,859959355	0,863	0,301	7,95161E-81	9
Qdpr	1,62216E-84	2,078962972	0,812	0,267	2,60309E-80	9
Dpp6	3,15915E-83	2,418987881	0,521	0,104	5,06948E-79	9
Igfbpl1	0	6,136487959	0,617	0,017	0	10
Mfap4	1,4124E-199	3,912899077	0,753	0,102	2,2665E-195	10
Chodl	6,4359E-191	3,867752969	0,718	0,1	1,0328E-186	10
Cox8b	1,362E-190	4,529792779	0,489	0,038	2,1856E-186	10
Mfng	1,9065E-170	3,260942158	0,828	0,168	3,0593E-166	10
Btbd17	1,1311E-157	4,933014892	0,26	0,01	1,8151E-153	10
Nptx2	3,6823E-130	3,436289797	0,489	0,061	5,909E-126	10
Hes6	3,0175E-128	3,137060401	0,89	0,277	4,8422E-124	10
Pde2a	2,9628E-119	2,843801981	0,731	0,177	4,7544E-115	10
Dll3	9,8782E-117	3,306287903	0,551	0,093	1,5852E-112	10
Sec11c	5,3187E-115	2,699417237	0,771	0,199	8,535E-111	10
Gadd45g	3,9117E-113	3,226029997	0,771	0,206	6,277E-109	10
St18	1,4204E-110	3,564900352	0,41	0,05	2,2793E-106	10
Sox11	2,4706E-102	2,200463954	0,899	0,321	3,96456E-98	10
Ppp1r14b	7,7409E-99	2,164994943	0,978	0,482	1,24219E-94	10
Kcne1l	8,31597E-98	2,526519247	0,74	0,192	1,33446E-93	10
Hmgn2	2,14781E-97	1,93717994	0,991	0,683	3,44659E-93	10
Ptma	6,66587E-91	0,935891997	1	1	1,06967E-86	10
Miat	1,65564E-85	3,001671673	0,52	0,11	2,65681E-81	10
Rps3	1,68971E-83	0,889788321	1	0,999	2,71148E-79	10
Hnrnpa1	5,56749E-82	1,373282019	0,987	0,854	8,93416E-78	10
H3f3a	2,17003E-77	0,841459711	1	0,998	3,48225E-73	10
Mfap2	4,32889E-77	1,652552365	0,96	0,65	6,94657E-73	10
Syce2	8,67641E-76	2,860338664	0,432	0,08	1,3923E-71	10
Pdzrn3	2,37771E-73	2,284491695	0,63	0,174	3,81552E-69	10
Rps24	7,54781E-72	0,712210519	1	0,999	1,2112E-67	10
Ascl1	1,92401E-71	2,123919123	0,718	0,234	3,08745E-67	10
Nxph4	7,7141E-68	2,015122677	0,643	0,197	1,23788E-63	10
Slc10a4	1,31964E-67	2,002448343	0,793	0,343	2,11762E-63	10
Nt5dc2	2,00532E-67	1,867518777	0,797	0,335	3,21793E-63	10
Cdk2ap1	3,09053E-65	2,022718501	0,705	0,25	4,95937E-61	10
Rpl21	6,97927E-62	0,634246781	1	0,999	1,11996E-57	10
Bub3	2,47208E-59	1,547748497	0,952	0,631	3,96694E-55	10
Rpl4	2,93303E-58	0,696905099	1	0,997	4,70664E-54	10
Chd7	2,91789E-56	1,55614923	0,833	0,383	4,68234E-52	10
Srrm4	7,65141E-56	1,947601261	0,564	0,18	1,22782E-51	10
Eef2	1,16332E-55	0,743672678	1	0,996	1,86678E-51	10

Eef1a1	2,66225E-54	0,549858295	1	1	4,27212E-50	10
Rps28	4,64904E-54	0,65508838	1	0,999	7,46032E-50	10
Rps7	1,14655E-53	0,607937711	1	0,999	1,83987E-49	10
Rps26	4,89639E-52	0,67954938	1	0,996	7,85724E-48	10
Eif4a1	8,5152E-52	0,852536832	0,996	0,973	1,36643E-47	10
Anks1	1,74742E-51	2,414078645	0,344	0,068	2,80409E-47	10
Shmt2	1,04851E-50	1,869654006	0,568	0,188	1,68254E-46	10
Rps11	2,48165E-50	0,601899647	1	0,999	3,9823E-46	10
Rps18	2,93309E-50	0,595917415	1	0,999	4,70673E-46	10
Rps6	8,55573E-50	0,59559792	1	0,999	1,37294E-45	10
Cilp	1,02713E-49	2,92183741	0,269	0,046	1,64823E-45	10
Hsp90ab1	1,26452E-49	0,610419818	1	0,998	2,02917E-45	10
Tmeff2	1,6653E-49	1,383586746	0,366	0,078	2,6723E-45	10
Nfib	2,95208E-49	1,383545195	0,89	0,518	4,7372E-45	10
Phox2a	4,64554E-49	1,876788525	0,749	0,38	7,45469E-45	10
Nfix	1,0447E-48	1,417134559	0,868	0,489	1,67643E-44	10
Rcor2	1,3833E-48	2,210529834	0,37	0,084	2,21978E-44	10
Rplp0	2,67427E-48	0,637881015	1	0,999	4,2914E-44	10
Rps5	1,883E-47	0,531887799	1	0,999	3,02165E-43	10
Rpl27a	5,47848E-47	0,617975665	1	0,997	8,79131E-43	10
Shfm1	5,80474E-47	0,923827198	0,987	0,911	9,31486E-43	10
Rpl3	1,17149E-46	0,553746447	1	0,999	1,8799E-42	10
Rps13	6,1628E-46	0,605025327	1	0,995	9,88945E-42	10
Gadd45gip1	8,44709E-46	1,878406682	0,595	0,225	1,3555E-41	10
Ndufa4l2	1,65684E-45	1,967721307	0,555	0,189	2,65874E-41	10
Rps9	2,19483E-45	0,549371718	1	0,999	3,52204E-41	10
Cdk4	2,62773E-45	1,230237234	0,925	0,562	4,21673E-41	10
Gpr22	2,47749E-44	1,623144967	0,643	0,264	3,97563E-40	10
Rpl15	2,81853E-44	0,5879372	1	0,996	4,52289E-40	10
Rps27a	2,89469E-44	0,544405081	1	1	4,64512E-40	10
Rps2	4,17868E-44	0,57907302	1	0,999	6,70553E-40	10
Chga	7,11426E-44	1,703041634	0,648	0,287	1,14163E-39	10
Hist3h2ba	2,10011E-43	1,454342312	0,731	0,348	3,37005E-39	10
Rps15a	3,08635E-43	0,615180227	1	0,997	4,95266E-39	10
Prdx2	3,72928E-43	0,648161053	0,996	0,98	5,98437E-39	10
Rps19	4,39327E-43	0,536272431	1	0,999	7,04989E-39	10
Rpl32	7,27148E-43	0,524133142	1	0,999	1,16686E-38	10
Rpl37	4,56396E-42	0,463041784	1	1	7,32379E-38	10
Slc25a5	1,8705E-41	0,963487241	0,965	0,75	3,00159E-37	10
Rpl18a	7,76849E-41	0,525116005	1	1	1,24661E-36	10
Rpl6	8,87135E-41	0,486681813	1	0,999	1,42359E-36	10
Rps3a1	1,3967E-40	0,552508357	1	0,998	2,24128E-36	10
Tle1	1,59922E-40	1,723935017	0,59	0,233	2,56627E-36	10
Amer2	6,00128E-40	2,184213491	0,317	0,073	9,63026E-36	10
Rps8	6,00769E-40	0,444496077	1	1	9,64055E-36	10
Tubb3	8,74017E-40	1,096759974	0,921	0,576	1,40254E-35	10
Elavl3	1,10348E-39	1,218924304	0,727	0,332	1,77075E-35	10
Podxl2	5,66762E-39	1,341684193	0,771	0,399	9,09483E-35	10

Rpl13a	1,65289E-38	0,500645735	1	1	2,6524E-34	10
Npm1	5,2203E-37	0,788111038	0,991	0,899	8,37702E-33	10
Kcnj12	1,39731E-36	1,982732243	0,405	0,13	2,24226E-32	10
Rpl19	1,89666E-36	0,454335463	1	1	3,04357E-32	10
Inppl1	3,52229E-36	2,405061433	0,26	0,055	5,65222E-32	10
Chd3	9,73527E-36	1,27206489	0,744	0,383	1,56222E-31	10
Rps21	1,38849E-35	0,593582574	0,996	0,983	2,22811E-31	10
Hnrnpab	1,43802E-35	1,076632823	0,859	0,51	2,30758E-31	10
Traf4	5,60425E-35	1,647115736	0,498	0,181	8,99314E-31	10
Rpl18	1,40709E-34	0,546261687	1	0,995	2,25796E-30	10
Gdpd1	1,39972E-33	1,512650776	0,537	0,229	2,24614E-29	10
Nbl1	2,42427E-33	1,394513575	0,626	0,282	3,89023E-29	10
Rpl10a	4,02512E-33	0,488392077	1	0,993	6,45912E-29	10
Abracl	5,12794E-33	1,601519705	0,463	0,166	8,22881E-29	10
Ddx5	1,31179E-32	0,677467409	0,991	0,968	2,10502E-28	10
Cacna2d1	1,13948E-31	1,435040876	0,612	0,301	1,82852E-27	10
Rpl32	1,17035E-71	0,830341974	1	0,999	1,87806E-67	11
Rps18	1,10206E-70	0,799015029	1	0,999	1,76847E-66	11
Rps3a1	3,22891E-70	0,852304774	1	0,998	5,18144E-66	11
Rpl13	8,91978E-67	0,741922566	1	1	1,43136E-62	11
Rpl18a	5,11783E-66	0,784996472	1	1	8,21259E-62	11
Rps19	1,05649E-65	0,74953161	1	0,999	1,69535E-61	11
Ckb	5,77569E-64	1,542687825	0,973	0,715	9,26824E-60	11
Rps27a	4,16643E-62	0,691025683	1	1	6,68587E-58	11
Rps15a	1,5301E-61	0,801372934	1	0,997	2,45534E-57	11
Rpl10a	2,43462E-60	0,786885334	1	0,993	3,90683E-56	11
Rpl36	7,87975E-60	0,765506199	1	0,999	1,26446E-55	11
Rps4x	9,47131E-58	0,669602207	1	1	1,51986E-53	11
Rpl13a	1,40246E-57	0,690753933	1	1	2,25053E-53	11
Rpl34	6,47634E-57	0,721327426	1	0,997	1,03926E-52	11
Rpl8	7,95648E-57	0,715849905	1	0,999	1,27678E-52	11
Rps3	1,05418E-56	0,698781435	1	0,999	1,69164E-52	11
Rpl23a	1,75727E-56	0,673224176	1	0,999	2,81989E-52	11
Rps2	6,72014E-56	0,747034116	1	0,999	1,07838E-51	11
Rps6	7,2891E-56	0,709117594	1	0,999	1,16968E-51	11
Rps9	2,72144E-55	0,667319977	1	0,999	4,36709E-51	11
Rps23	1,32037E-54	0,621326432	1	1	2,1188E-50	11
Rpl37a	1,88956E-54	0,585715534	1	1	3,03218E-50	11
Rps14	3,61483E-54	0,646202212	1	0,999	5,80071E-50	11
Rps27	3,41258E-53	0,664692775	1	1	5,47617E-49	11
Rps26	3,95947E-53	0,770483059	1	0,996	6,35376E-49	11
Rps12	1,31551E-52	0,910818127	0,996	0,946	2,111E-48	11
Rpl35	1,38838E-52	0,634007957	1	0,998	2,22793E-48	11
Rps8	3,00941E-52	0,603443957	1	1	4,82919E-48	11
Rpl23	3,17077E-52	0,678187228	1	0,999	5,08814E-48	11
Rpl18	1,09619E-51	0,707838828	1	0,995	1,75906E-47	11
Rplp2	3,26225E-51	0,691856182	1	0,999	5,23493E-47	11
Rpl35a	5,74312E-51	0,668246897	1	0,999	9,21599E-47	11

Rpl37	9,81133E-51	0,596352621	1	1	1,57442E-46	11
Rpl17	3,16704E-50	0,668310353	1	0,999	5,08214E-46	11
Rpl9	1,16836E-49	0,645670586	1	0,999	1,87488E-45	11
Rplp0	1,53308E-49	0,663423239	1	0,999	2,46013E-45	11
Rps11	1,88256E-49	0,648001678	1	0,999	3,02095E-45	11
Rpl3	6,21101E-49	0,645249529	1	0,999	9,96681E-45	11
Rps16	2,53333E-48	0,65545602	1	0,999	4,06524E-44	11
Rps13	3,40627E-48	0,731220143	1	0,995	5,46605E-44	11
Naca	1,01934E-47	0,855817253	0,996	0,967	1,63574E-43	11
Rps29	1,13841E-47	0,506254283	1	1	1,82681E-43	11
Rpsa	2,12704E-47	0,723390598	1	0,992	3,41327E-43	11
Rpl36a	1,22597E-46	0,761615648	1	0,987	1,96731E-42	11
Rps5	1,34961E-46	0,585784241	1	0,999	2,16571E-42	11
Rpl4	1,66478E-46	0,672897139	1	0,997	2,67148E-42	11
Rps28	5,17219E-46	0,646313811	1	0,999	8,29981E-42	11
Rpl27a	7,8127E-46	0,643552403	1	0,997	1,2537E-41	11
Npm1	1,30102E-44	0,961388161	0,973	0,9	2,08775E-40	11
Ccnd1	3,07615E-43	1,758472113	0,602	0,224	4,9363E-39	11
Rpl39	3,59968E-43	0,599627681	1	0,999	5,7764E-39	11
Rpl28	8,77195E-43	0,746311038	1	0,983	1,40763E-38	11
Rpl6	9,86005E-43	0,554609581	1	0,999	1,58224E-38	11
Rpl7	2,25155E-42	0,685347178	1	0,99	3,61305E-38	11
Rpl26	2,69183E-42	0,593235659	1	1	4,31958E-38	11
Eef1a1	5,93691E-42	0,507367588	1	1	9,52696E-38	11
Rps20	3,63412E-41	0,687869325	1	0,986	5,83168E-37	11
Rps17	6,30626E-40	0,603849895	1	0,996	1,01197E-35	11
Cnp	9,07793E-40	1,234235625	0,863	0,546	1,45674E-35	11
Rpl31	1,10593E-39	0,647815092	1	0,992	1,77469E-35	11
Eif3f	1,57384E-39	0,848982337	0,996	0,93	2,52554E-35	11
Gnb2l1	1,9602E-39	0,874514311	0,969	0,87	3,14554E-35	11
Rpl22	2,26285E-39	0,947429757	0,951	0,805	3,6312E-35	11
Rpl24	2,2651E-39	0,594092549	1	0,999	3,63481E-35	11
Rpl11	3,77207E-39	0,57279076	1	1	6,05304E-35	11
Rps25	4,01541E-39	0,648790977	1	0,994	6,44352E-35	11
Rpl15	3,27396E-38	0,598271119	1	0,996	5,25372E-34	11
Rpl19	3,68462E-38	0,536609679	1	1	5,91271E-34	11
Rps10	9,23794E-38	0,714701141	1	0,982	1,48241E-33	11
Rpl5	1,4543E-37	0,815962404	0,978	0,903	2,33371E-33	11
Rpl12	2,79837E-37	0,872166242	0,965	0,902	4,49055E-33	11
Rpl21	5,30929E-37	0,484085001	1	0,999	8,51982E-33	11
Rpl27	2,86086E-36	0,56067173	1	0,993	4,59082E-32	11
Rps7	4,79368E-36	0,520739533	1	0,999	7,69242E-32	11
Rpl14	5,376E-36	0,469148881	1	1	8,62687E-32	11
Uba52	1,50503E-35	0,722064346	1	0,95	2,41512E-31	11
Rpl30	2,81342E-35	0,712272756	1	0,94	4,5147E-31	11
Rps15	4,64791E-35	0,523128996	1	0,999	7,4585E-31	11
Rplp1	2,4091E-34	0,511704378	1	0,999	3,86589E-30	11
Csrp1	2,30434E-33	0,776178591	0,987	0,942	3,69777E-29	11

Ascl1	1,78613E-32	2,06136602	0,527	0,242	2,8662E-28	11
Tpt1	4,86756E-32	0,552671716	1	0,999	7,81098E-28	11
Fau	5,35509E-32	0,466726988	1	0,999	8,59331E-28	11
Ppp1r14b	5,50492E-32	1,29594347	0,748	0,491	8,83375E-28	11
Ednrb	8,64913E-31	1,141246803	0,796	0,5	1,38793E-26	11
Fbln1	1,25709E-29	1,274295195	0,686	0,352	2,01726E-25	11
Nell2	1,37171E-29	1,131144466	0,779	0,485	2,20118E-25	11
Tmem123	2,44639E-28	1,841693986	0,416	0,163	3,92573E-24	11
Rps24	2,76691E-28	0,429633999	1	0,999	4,44006E-24	11
Eef1g	2,39839E-27	0,632588351	0,991	0,964	3,8487E-23	11
Rpl10	1,07532E-26	0,395047887	1	1	1,72557E-22	11
Cryab	5,71101E-26	0,66947409	0,982	0,81	9,16446E-22	11
Rpl41	1,64191E-25	0,311313661	1	1	2,63477E-21	11
Btf3	7,81812E-25	0,584821082	0,996	0,959	1,25457E-20	11
Cox7a2l	1,49319E-24	0,739129618	0,942	0,866	2,39612E-20	11
Eef1d	1,66619E-24	0,977156368	0,757	0,516	2,67373E-20	11
Snrpg	2,49789E-24	0,682708497	0,92	0,797	4,00837E-20	11
Pabpc1	1,38627E-23	0,832973629	0,898	0,777	2,22455E-19	11
Rpl7a	9,3414E-23	0,600212634	0,987	0,952	1,49901E-18	11
Rbp1	1,10234E-22	0,676658169	0,951	0,678	1,76893E-18	11
Plekhb1	6,39671E-22	0,773498784	0,814	0,483	1,02648E-17	11
Etv1	1,841E-192	2,901021458	0,99	0,189	2,9542E-188	12
Nos1	1,1966E-189	2,095604953	0,722	0,077	1,9201E-185	12
Tmod1	1,673E-176	3,086747568	0,861	0,149	2,6847E-172	12
Fhod3	6,4124E-126	2,353506569	0,943	0,263	1,029E-121	12
Gal	1,9232E-114	1,552649254	0,928	0,304	3,0862E-110	12
Nrp1	2,2658E-114	2,566808716	0,923	0,287	3,636E-110	12
Ass1	1,7154E-112	2,138251601	0,938	0,282	2,7526E-108	12
Pirt	1,3407E-108	2,274135932	0,919	0,288	2,1515E-104	12
Dpysl3	1,6413E-107	2,065168463	1	0,676	2,6339E-103	12
Mtus2	4,616E-107	3,010764555	0,569	0,098	7,4073E-103	12
Tesc	1,30029E-95	2,992607303	0,431	0,058	2,08657E-91	12
Kcnq3	1,63412E-91	2,448268846	0,632	0,137	2,62228E-87	12
Ret	5,06937E-88	2,042977969	0,775	0,209	8,13482E-84	12
Adgrb3	5,15309E-88	2,286693855	0,727	0,193	8,26916E-84	12
Camkk2	8,38339E-86	2,404905613	0,689	0,173	1,34528E-81	12
Gm13889	8,66115E-86	2,258044847	0,646	0,141	1,38986E-81	12
Clvs1	3,22441E-83	2,536865068	0,522	0,102	5,17421E-79	12
Elavl4	1,53512E-82	1,637974106	0,995	0,53	2,46341E-78	12
Tmem255b	1,49224E-81	1,928788434	0,804	0,241	2,3946E-77	12
Fam155a	4,22305E-77	2,472086592	0,488	0,092	6,77673E-73	12
Gng8	7,97986E-77	2,964900623	0,392	0,06	1,28053E-72	12
Hs3st5	9,17551E-77	2,640480023	0,507	0,102	1,47239E-72	12
Lix1	6,84914E-76	1,699242259	0,885	0,306	1,09908E-71	12
Celf4	6,61296E-73	1,678393579	0,947	0,391	1,06118E-68	12
Ank2	1,85169E-70	1,606071714	0,947	0,473	2,97141E-66	12
P2ry6	2,28042E-70	2,851559721	0,402	0,069	3,65939E-66	12
Shc1	6,20752E-70	2,288923074	0,541	0,12	9,96121E-66	12

Cox8b	1,1124E-67	2,238151855	0,33	0,046	1,78506E-63	12
Spock3	8,00319E-67	1,440710457	0,837	0,282	1,28427E-62	12
Trib2	5,8899E-65	1,857592933	0,699	0,21	9,45152E-61	12
Fst	6,18655E-65	2,283010534	0,565	0,14	9,92756E-61	12
Ppm1h	7,12401E-65	1,837889819	0,699	0,214	1,14319E-60	12
Ptma	1,85862E-62	0,70526302	1	1	2,98253E-58	12
Garnl3	2,98175E-61	1,702414694	0,799	0,293	4,78482E-57	12
Tspan13	7,22386E-61	1,68588809	0,813	0,307	1,15921E-56	12
Tbpl1	3,92254E-60	1,885219516	0,746	0,25	6,2945E-56	12
Cntnap5a	4,25616E-60	2,261137587	0,464	0,102	6,82986E-56	12
Stmn4	1,15036E-58	1,570441916	0,88	0,386	1,84599E-54	12
Tubb3	3,1126E-58	1,298093569	0,99	0,575	4,99479E-54	12
Smpd3	5,02616E-58	1,455914685	0,885	0,383	8,06547E-54	12
Cd1d1	6,34631E-58	2,146526	0,569	0,157	1,01839E-53	12
Arpp21	4,18089E-57	1,854696461	0,593	0,167	6,70908E-53	12
Aldh1a3	5,33243E-57	2,28258026	0,517	0,127	8,55696E-53	12
Gria2	4,33582E-56	1,831047621	0,622	0,197	6,95769E-52	12
Slit2	4,59171E-55	2,077030483	0,608	0,19	7,36832E-51	12
Mapk10	2,56117E-54	1,782996206	0,708	0,264	4,10991E-50	12
Map1b	6,27141E-54	1,051742871	0,995	0,845	1,00637E-49	12
S100a1	7,08294E-54	1,522590433	0,861	0,357	1,1366E-49	12
Kcnq4	7,67469E-53	2,346400762	0,416	0,092	1,23156E-48	12
Calm2	1,281E-52	0,944523408	1	0,978	2,05561E-48	12
Moxd1	2,8134E-52	1,425190486	0,416	0,084	4,51467E-48	12
Crmp1	9,03625E-51	1,330869234	0,866	0,36	1,45005E-46	12
Ebf1	1,59973E-49	2,097153383	0,287	0,047	2,56709E-45	12
Mllt11	2,51822E-49	1,112731937	0,962	0,5	4,04099E-45	12
Srrm4	2,76521E-49	1,725990311	0,589	0,18	4,43733E-45	12
Tagln3	2,96365E-49	1,299796465	0,856	0,374	4,75577E-45	12
Tbx3	5,33315E-49	1,461201047	0,847	0,363	8,5581E-45	12
Aplp1	1,75866E-48	1,184522615	0,928	0,416	2,82213E-44	12
Calm1	5,89224E-48	0,986558723	1	0,927	9,45528E-44	12
Tuba1a	1,08667E-47	0,757165114	1	0,999	1,74378E-43	12
Lrrfip1	1,13168E-47	1,399755711	0,833	0,368	1,81601E-43	12
Hspb8	1,25758E-47	1,696519727	0,632	0,211	2,01805E-43	12
Glipr2	3,76054E-47	1,81329491	0,569	0,177	6,03454E-43	12
Tcf4	1,80824E-46	1,012215049	0,981	0,729	2,90168E-42	12
Ptprr	4,83995E-46	1,946508724	0,483	0,135	7,76667E-42	12
Ptgds	5,9687E-46	2,273944249	0,402	0,099	9,57797E-42	12
Enpp1	6,73535E-46	2,362483974	0,325	0,065	1,08082E-41	12
S100a13	9,33522E-46	1,273562121	0,847	0,37	1,49802E-41	12
Bex2	7,78981E-45	0,982637295	0,995	0,677	1,25003E-40	12
S100a16	1,10534E-44	1,146043833	0,976	0,773	1,77375E-40	12
Pcbp3	4,23648E-43	1,128185172	0,909	0,437	6,79828E-39	12
Chrnb4	3,77808E-42	1,666744764	0,589	0,204	6,06268E-38	12
Klf7	4,58337E-42	1,417833311	0,799	0,376	7,35493E-38	12
Elavl3	8,59218E-42	1,319851126	0,775	0,331	1,37879E-37	12
Mapt	1,24897E-41	1,261553054	0,842	0,415	2,00422E-37	12

Cd24a	3,31705E-41	1,178750622	0,957	0,676	5,32286E-37	12
Basp1	6,23875E-41	1,194051217	0,904	0,445	1,00113E-36	12
Dach1	8,75807E-41	2,481752965	0,263	0,048	1,40541E-36	12
Sh3gl2	4,76395E-40	1,982219398	0,426	0,123	7,64472E-36	12
Azin2	4,88448E-40	1,524910362	0,636	0,243	7,83813E-36	12
Fstl5	9,16452E-40	1,797978567	0,488	0,157	1,47063E-35	12
Eml5	7,28077E-39	1,608589925	0,512	0,171	1,16835E-34	12
Tmem108	8,82173E-39	2,17646417	0,325	0,075	1,41562E-34	12
Klc1	1,58895E-38	0,992097469	0,943	0,577	2,54979E-34	12
Tubb2b	2,42957E-38	0,967115878	0,99	0,63	3,89873E-34	12
Gm1673	1,69152E-37	1,116110373	0,919	0,508	2,71438E-33	12
Fam57b	2,13842E-37	1,622445212	0,555	0,2	3,43153E-33	12
Gap43	2,62662E-37	0,940188835	0,967	0,712	4,21494E-33	12
Stx3	6,41422E-37	2,006280291	0,34	0,084	1,02929E-32	12
Tril	1,17263E-36	1,761745644	0,474	0,151	1,88173E-32	12
Id1	8,76507E-36	1,66495801	0,565	0,205	1,40653E-31	12
Pfkp	8,96511E-36	1,352478344	0,66	0,268	1,43863E-31	12
Npy	9,49562E-36	0,670162234	0,56	0,195	1,52376E-31	12
Creb5	1,23603E-35	1,432711752	0,684	0,283	1,98346E-31	12
Samd5	1,42387E-35	2,406544918	0,258	0,052	2,28488E-31	12
Rbms3	2,12009E-35	1,136162086	0,856	0,472	3,40211E-31	12
Camta1	3,78289E-35	1,157283126	0,818	0,405	6,07041E-31	12
Trim62	4,33301E-35	2,114486093	0,297	0,067	6,95318E-31	12
Pcbd1	4,78887E-35	1,290590103	0,617	0,228	7,68471E-31	12
Cav2	2,03931E-34	2,276331468	0,282	0,063	3,27248E-30	12
R3hdm1	2,85735E-34	1,381461963	0,694	0,315	4,58519E-30	12
Frzb	2,4587E-162	2,937469064	0,787	0,12	3,9455E-158	13
Col9a2	1,6575E-158	2,733947297	0,98	0,224	2,6597E-154	13
Igfbp4	4,5359E-134	2,687351895	0,96	0,28	7,2788E-130	13
S1pr3	1,6917E-122	2,767627004	0,718	0,13	2,7146E-118	13
Hey2	1,0606E-114	3,012892695	0,599	0,096	1,702E-110	13
Egr1	3,0946E-111	2,694279828	0,911	0,294	4,9659E-107	13
Btg2	2,5161E-101	2,817174949	0,807	0,232	4,03765E-97	13
Ptn	6,1891E-100	2,196972107	0,995	0,59	9,93159E-96	13
Junb	9,1616E-100	2,788976437	0,965	0,469	1,47016E-95	13
Apoe	8,60829E-95	2,136356338	1	0,609	1,38137E-90	13
Mmd2	5,97168E-94	2,204943334	0,767	0,184	9,58276E-90	13
Fos	7,07191E-92	2,327041432	0,99	0,667	1,13483E-87	13
Qpct	2,17325E-90	2,326467536	0,817	0,241	3,48742E-86	13
Cyr61	1,22435E-89	2,613401091	0,847	0,287	1,96471E-85	13
Socs3	3,18825E-89	2,828372057	0,728	0,196	5,11618E-85	13
Hes1	3,70004E-85	2,495123628	0,901	0,397	5,93745E-81	13
Tbx3os1	4,40094E-85	2,315120012	0,827	0,266	7,06219E-81	13
Entpd2	3,08487E-79	1,693580342	0,936	0,311	4,95028E-75	13
Taf13	3,91104E-79	1,937110771	0,921	0,405	6,27605E-75	13
Tmprss5	5,97083E-76	1,984249717	0,777	0,223	9,58139E-72	13
Lpar1	6,8699E-73	1,738790436	0,946	0,378	1,10241E-68	13
Ier2	6,96279E-73	2,386543143	0,871	0,414	1,11732E-68	13

Fam107a	1,10247E-72	3,088742904	0,307	0,036	1,76913E-68	13
Grb14	1,58485E-72	2,036599672	0,748	0,227	2,54321E-68	13
Hspa1a	2,48692E-71	2,15386263	0,832	0,317	3,99076E-67	13
Cpxm2	2,1372E-68	2,909912559	0,317	0,041	3,42957E-64	13
Olfml3	1,54574E-67	1,607958881	0,926	0,367	2,48045E-63	13
Chadl	3,5825E-67	1,950116261	0,748	0,252	5,74883E-63	13
Zfp36	5,8518E-66	3,180263236	0,406	0,074	9,39039E-62	13
Jun	2,09053E-65	2,000592169	0,941	0,633	3,35468E-61	13
Nid1	1,14092E-63	1,889388535	0,782	0,271	1,83083E-59	13
Timp3	2,60121E-63	1,597528125	0,955	0,488	4,17416E-59	13
Fbln2	5,7985E-63	2,147706705	0,574	0,145	9,30485E-59	13
Prss56	1,005E-62	2,915518466	0,262	0,031	1,61273E-58	13
Eln	5,19607E-61	1,971824744	0,762	0,3	8,33813E-57	13
Sdc4	7,61835E-61	2,298295724	0,559	0,143	1,22252E-56	13
Sparc	5,43644E-60	1,247717528	1	0,798	8,72385E-56	13
Tgfb2	6,58752E-59	1,512226771	0,955	0,503	1,0571E-54	13
Cmtm5	7,23564E-59	1,831895422	0,708	0,222	1,1611E-54	13
Cst3	1,0433E-57	1,260808424	1	0,912	1,67419E-53	13
Loxl1	6,56814E-57	2,715058524	0,401	0,082	1,05399E-52	13
Gsta4	7,8603E-57	1,79418667	0,668	0,202	1,26134E-52	13
Plekhb1	5,09971E-56	1,396656412	0,96	0,479	8,18351E-52	13
Pla2g16	6,62001E-55	2,067070532	0,574	0,158	1,06231E-50	13
Col11a1	7,04154E-54	2,259035242	0,47	0,113	1,12996E-49	13
Gm2115	4,98897E-53	2,018462523	0,579	0,168	8,00581E-49	13
Cthrc1	2,42781E-52	1,404937312	0,757	0,26	3,89591E-48	13
Ngfr	2,4945E-52	1,522863303	0,896	0,425	4,00292E-48	13
Slc18a2	5,44347E-52	1,731658721	0,584	0,177	8,73514E-48	13
Gpr37	9,45407E-52	2,365396653	0,416	0,09	1,51709E-47	13
Atp1a2	2,01946E-51	1,518543217	0,812	0,32	3,24063E-47	13
Gas1	1,44681E-50	1,984028305	0,644	0,218	2,32169E-46	13
Ttyh1	1,49476E-50	1,682115057	0,782	0,324	2,39865E-46	13
Tspan3	2,56287E-50	1,063329802	0,99	0,871	4,11263E-46	13
Tbc1d10a	1,56515E-49	1,823737005	0,634	0,209	2,5116E-45	13
Itm2b	1,78445E-48	0,670157471	1	0,998	2,86351E-44	13
Nr4a1	2,34281E-48	2,36352888	0,624	0,233	3,75951E-44	13
Gpr37l1	1,94678E-47	1,301565789	0,931	0,493	3,12399E-43	13
Pmepa1	2,4664E-46	1,513789288	0,752	0,3	3,95783E-42	13
Ptrf	5,05306E-46	1,965614545	0,455	0,114	8,10864E-42	13
H19	1,42648E-45	2,69198416	0,322	0,063	2,28907E-41	13
Nrarp	2,05564E-44	2,411701197	0,351	0,076	3,29868E-40	13
Wnt6	3,13658E-44	2,089586709	0,411	0,099	5,03328E-40	13
Mest	6,33975E-44	1,624561152	0,787	0,388	1,01734E-39	13
Spint2	8,47284E-44	1,714967851	0,708	0,328	1,35964E-39	13
Hmcn1	1,69552E-43	1,788201205	0,569	0,189	2,72079E-39	13
Ccnd2	3,04104E-43	1,527412462	0,678	0,241	4,87995E-39	13
Mdfi	1,18562E-42	2,173329989	0,426	0,109	1,90257E-38	13
Gsn	4,87825E-42	1,473624482	0,807	0,42	7,82813E-38	13
Fosb	8,2259E-42	2,341776851	0,426	0,114	1,32001E-37	13

Igfbp2	1,0234E-40	2,580040201	0,282	0,054	1,64225E-36	13
Zfp36l1	1,22477E-40	1,570467575	0,767	0,324	1,96538E-36	13
Itm2c	1,50354E-40	1,285949297	0,866	0,563	2,41274E-36	13
Adgra3	1,71326E-40	2,03927878	0,416	0,11	2,74926E-36	13
Scrg1	1,45193E-39	2,149204281	0,401	0,105	2,32991E-35	13
Rbp1	2,49663E-39	1,014254208	0,995	0,678	4,00634E-35	13
Gfap	9,38632E-39	2,150846329	0,332	0,074	1,50622E-34	13
Sat1	2,0594E-38	1,550346181	0,757	0,373	3,30473E-34	13
S100b	2,92924E-38	1,34830364	0,777	0,357	4,70055E-34	13
Tst	4,65205E-38	2,050436746	0,49	0,161	7,46515E-34	13
Ramp1	7,84241E-38	1,302545044	0,832	0,452	1,25847E-33	13
Vim	1,58151E-37	0,960623553	0,995	0,704	2,53785E-33	13
Gas7	2,01223E-37	1,18793924	0,871	0,43	3,22902E-33	13
Fabp7	4,57061E-37	1,051750708	0,975	0,557	7,33446E-33	13
Mdk	9,33944E-37	1,404557912	0,708	0,304	1,4987E-32	13
Gfra1	1,09392E-36	1,509550512	0,703	0,322	1,75541E-32	13
Islr	1,46867E-36	1,995870414	0,356	0,087	2,35677E-32	13
Zfp36l2	1,51892E-36	1,497031494	0,698	0,307	2,43741E-32	13
Gab1	1,61588E-36	1,659391984	0,535	0,185	2,59301E-32	13
Poc1a	1,55178E-35	1,435897468	0,584	0,207	2,49014E-31	13
Rasl11b	1,88341E-35	1,765405203	0,505	0,174	3,0223E-31	13
Lrrtm1	4,58261E-35	1,62061785	0,559	0,207	7,35372E-31	13
Phgdh	7,53384E-35	1,450839596	0,713	0,345	1,20896E-30	13
Plcb1	1,35184E-34	1,477122486	0,624	0,247	2,16929E-30	13
Sox2	2,00582E-34	1,951766325	0,475	0,157	3,21874E-30	13
Fxyd1	4,75896E-34	0,946564147	0,975	0,555	7,6367E-30	13
Cd59a	1,1307E-33	1,189535512	0,842	0,486	1,81444E-29	13
Ptgfrn	1,23599E-33	2,256518345	0,282	0,062	1,98339E-29	13
Col18a1	2,67165E-33	1,12744863	0,822	0,401	4,28719E-29	13
Etl4	6,3846E-33	2,151629978	0,282	0,063	1,02454E-28	13
Col12a1	1,00309E-32	1,467605919	0,594	0,231	1,60966E-28	13
Scn7a	3,09349E-58	2,93623621	0,598	0,197	4,96412E-54	14
Marcks	1,60467E-57	1,586175186	1	0,716	2,57501E-53	14
Dbi	1,80878E-45	1,228463731	1	0,652	2,90255E-41	14
Col20a1	7,02239E-44	2,082917055	0,649	0,267	1,12688E-39	14
Son	4,48221E-40	1,285766279	0,948	0,75	7,1926E-36	14
mt-Co3	1,02314E-38	0,662188789	1	1	1,64183E-34	14
Igfbp7	1,08688E-38	3,909022895	0,515	0,205	1,74412E-34	14
Gm42418	1,68682E-37	1,471193421	0,99	0,729	2,70683E-33	14
Zeb2	6,22201E-37	1,339008656	0,907	0,586	9,98446E-33	14
Abca8a	1,86369E-35	2,759549536	0,433	0,147	2,99066E-31	14
Plp1	5,3454E-35	1,201277183	0,876	0,442	8,57776E-31	14
mt-Atp6	9,86973E-35	0,618170779	1	1	1,58379E-30	14
Itgb1	1,38646E-30	1,196616663	0,825	0,578	2,22486E-26	14
Fabp7	1,28645E-28	1,068541427	0,99	0,557	2,06436E-24	14
Lama4	1,45557E-28	2,27231969	0,423	0,164	2,33576E-24	14
Tnc	5,21678E-28	2,946232648	0,309	0,091	8,37137E-24	14
Col12a1	8,70896E-28	2,20288606	0,505	0,234	1,39753E-23	14

Fus	1,41342E-27	0,998930965	0,938	0,765	2,26811E-23	14
Col16a1	1,54047E-27	2,414815143	0,407	0,159	2,472E-23	14
Postn	2,4781E-27	1,254996036	0,716	0,394	3,97661E-23	14
Hspg2	1,99042E-26	2,613304442	0,325	0,106	3,19403E-22	14
Fxyd1	2,06262E-26	0,860141149	0,954	0,556	3,30988E-22	14
Col4a1	2,25319E-26	2,358343172	0,443	0,192	3,61569E-22	14
Malat1	6,45157E-25	0,645504447	1	1	1,03528E-20	14
Anxa5	7,89138E-24	0,753437147	0,907	0,569	1,26633E-19	14
Myl12a	2,46E-23	0,971808901	0,866	0,573	3,94757E-19	14
Lmo4	6,18798E-23	1,533942338	0,655	0,424	9,92985E-19	14
mt-Nd3	1,8053E-22	1,117977276	0,835	0,666	2,89697E-18	14
Col5a2	3,86818E-22	2,373291164	0,371	0,152	6,20728E-18	14
Pmepa1	4,34698E-22	1,779819004	0,541	0,309	6,97559E-18	14
Arpc1b	2,15808E-21	0,535729372	0,938	0,523	3,46308E-17	14
Serpine2	2,25711E-21	0,750730491	0,985	0,633	3,62199E-17	14
Col3a1	1,00324E-20	1,6416342	0,541	0,304	1,60989E-16	14
Slitrk6	1,27685E-20	2,197965149	0,397	0,182	2,04897E-16	14
Cd63	2,01419E-20	0,777802699	0,907	0,676	3,23216E-16	14
Hsp90b1	4,24513E-20	0,693443174	0,876	0,638	6,81216E-16	14
Timp2	7,94929E-20	1,028492534	0,773	0,593	1,27562E-15	14
Lamc1	8,01079E-20	2,368914362	0,371	0,166	1,28549E-15	14
Gstp1	9,17411E-20	0,928897733	0,773	0,554	1,47217E-15	14
Art3	1,46548E-19	1,846030863	0,397	0,176	2,35166E-15	14
Tra2b	1,82785E-19	0,953778565	0,856	0,636	2,93315E-15	14
Il1rap	1,88456E-19	2,546102128	0,309	0,119	3,02415E-15	14
Lamb1	2,40188E-19	2,503087373	0,314	0,123	3,85429E-15	14
Pnisr	2,52501E-19	1,087061426	0,763	0,604	4,05188E-15	14
Srsf5	2,69581E-19	0,816976057	0,835	0,629	4,32597E-15	14
Nedd4	7,03544E-19	0,723030868	0,907	0,697	1,12898E-14	14
Myeov2	1,37461E-18	0,812151059	0,881	0,719	2,20584E-14	14
Srrm2	3,48988E-18	0,856842329	0,814	0,634	5,60022E-14	14
Gm10076	4,27547E-18	0,757014166	0,897	0,679	6,86085E-14	14
Dad1	5,76099E-18	0,788135119	0,918	0,774	9,24467E-14	14
Rhoc	1,09264E-17	0,762777627	0,887	0,657	1,75336E-13	14
Cst3	1,11041E-17	0,555262726	1	0,912	1,78188E-13	14
Serf2	1,69118E-17	0,674711872	0,99	0,867	2,71384E-13	14
Rbm39	2,73047E-17	0,801257833	0,985	0,884	4,38158E-13	14
Pdlim4	3,42555E-17	1,74549735	0,418	0,212	5,49698E-13	14
Sfpq	1,07072E-16	0,811658765	0,825	0,644	1,71819E-12	14
Rhoa	1,19591E-16	0,672900822	0,856	0,644	1,91908E-12	14
mt-Nd1	1,401E-16	0,434807139	1	1	2,24818E-12	14
Rsrp1	4,22255E-16	0,75810373	0,948	0,795	6,77592E-12	14
Tmed10	4,29381E-16	0,614121324	0,881	0,667	6,89028E-12	14
Frmd6	4,65084E-16	2,305826322	0,278	0,109	7,4632E-12	14
mt-Nd2	6,28592E-16	0,706330975	0,979	0,819	1,0087E-11	14
Agrn	8,35953E-16	2,370826213	0,356	0,175	1,34145E-11	14
Fis1	1,41743E-15	0,707533468	0,83	0,664	2,27455E-11	14
mt-Cytb	1,53876E-15	0,392924548	1	1	2,46924E-11	14

Tpt1	1,62477E-15	0,43804175	1	0,999	2,60727E-11	14
Timp3	3,51443E-15	1,178936092	0,68	0,498	5,63961E-11	14
Serpinh1	4,71092E-15	0,54411407	0,892	0,557	7,55961E-11	14
Pfn1	5,41385E-15	0,593974304	1	0,936	8,6876E-11	14
Col4a2	8,55474E-15	2,433507142	0,309	0,145	1,37278E-10	14
Sf3b1	8,56796E-15	0,931862315	0,711	0,584	1,3749E-10	14
Hspa5	1,07114E-14	0,575318639	0,928	0,671	1,71886E-10	14
Hnrnpf	2,83284E-14	0,579560541	0,835	0,626	4,54586E-10	14
Nisch	6,61182E-14	1,102238098	0,66	0,573	1,061E-09	14
Rhbdf1	7,7834E-14	2,172729198	0,284	0,125	1,249E-09	14
Snrpf	7,9697E-14	0,581715573	0,845	0,659	1,2789E-09	14
Gng5	1,24745E-13	0,644017661	0,804	0,591	2,00179E-09	14
Mrpl52	1,79508E-13	0,683836444	0,835	0,677	2,88057E-09	14
Clic1	1,92365E-13	0,654129188	0,856	0,682	3,08689E-09	14
Cd200	2,27072E-13	1,066861518	0,737	0,569	3,64382E-09	14
Id3	3,30785E-13	1,881266679	0,546	0,416	5,3081E-09	14
Ndufa3	5,27056E-13	0,766734743	0,82	0,687	8,45767E-09	14
Kcna6	5,83731E-13	1,98463433	0,299	0,143	9,36713E-09	14
Tmem256	8,22251E-13	0,750704407	0,763	0,622	1,31947E-08	14
Rac1	8,28613E-13	0,580483281	0,881	0,7	1,32968E-08	14
Rps12	1,30924E-12	0,453056023	1	0,946	2,10093E-08	14
mt-Co2	1,43022E-12	0,408532283	1	1	2,29507E-08	14
Csnk1a1	1,94887E-12	0,608309901	0,845	0,696	3,12735E-08	14
Slc35f1	2,04612E-12	1,900948459	0,34	0,183	3,28341E-08	14
mt-Nd4	2,2288E-12	0,359934904	1	0,998	3,57656E-08	14
Sema3c	3,50184E-12	1,825920456	0,34	0,183	5,6194E-08	14
Sash1	4,08361E-12	1,738180802	0,361	0,207	6,55297E-08	14
Tomm7	4,24746E-12	0,613365938	0,861	0,71	6,8159E-08	14
Ddx39b	6,7925E-12	0,603531729	0,856	0,681	1,08999E-07	14
Srsf2	7,11836E-12	0,696898464	0,933	0,805	1,14228E-07	14
Rps27l	7,43218E-12	0,558039238	0,907	0,72	1,19264E-07	14
Diablo	1,00742E-11	0,699182985	0,84	0,645	1,61661E-07	14
Sec62	1,13492E-11	0,607028569	0,897	0,739	1,82121E-07	14
Qk	1,14449E-11	1,376327852	0,474	0,323	1,83656E-07	14
Tagln2	1,16297E-11	0,438430857	0,995	0,867	1,86622E-07	14
Cd81	1,44351E-11	0,443348495	1	0,982	2,3164E-07	14
Top2a	0	7,505629739	0,971	0,019	0	15
Birc5	0	6,709652198	0,953	0,018	0	15
Pbk	0	7,15782157	0,942	0,017	0	15
2810417H13Rik	0	6,031829886	0,93	0,027	0	15
Cdk1	0	6,912745643	0,895	0,02	0	15
Nusap1	0	6,794967512	0,895	0,022	0	15
Spc24	0	5,951190126	0,901	0,03	0	15
Spc25	0	7,139272287	0,872	0,012	0	15
Lockd	0	4,852464128	0,924	0,073	0	15
Prc1	0	7,209090909	0,855	0,013	0	15
Ccna2	0	7,015267513	0,849	0,011	0	15
Smc2	0	4,663869498	0,901	0,068	0	15

Racgap1	0	5,101035223	0,878	0,057	0	15
Cdca3	0	6,424849723	0,837	0,018	0	15
Cks2	0	5,343731486	0,866	0,055	0	15
Ccnb2	0	5,535682757	0,826	0,021	0	15
Mis18bp1	0	6,716759196	0,808	0,011	0	15
Neil3	0	7,253278688	0,802	0,007	0	15
Cdca8	0	5,827842336	0,82	0,025	0	15
Hist1h2ap	0	7,890316717	0,831	0,038	0	15
Ube2c	0	7,93346823	0,802	0,014	0	15
Mxd3	0	7,328432668	0,791	0,009	0	15
Tpx2	0	5,81786341	0,779	0,018	0	15
Aurkb	0	7,832472821	0,738	0,005	0	15
Rrm2	0	6,335683901	0,75	0,028	0	15
Mki67	0	5,124598005	0,727	0,037	0	15
Fam64a	0	6,385298993	0,698	0,01	0	15
Incenp	0	4,911997358	0,715	0,037	0	15
Mad2l1	0	4,88048649	0,709	0,038	0	15
Tk1	0	6,602450584	0,663	0,009	0	15
Cenpm	0	5,270277763	0,68	0,027	0	15
Ckap2l	0	6,168774464	0,657	0,01	0	15
Melk	0	6,582272641	0,628	0,007	0	15
Esco2	0	7,952774925	0,61	0,003	0	15
Ccnb1	0	6,535330856	0,616	0,011	0	15
Ndc80	0	7,594942479	0,61	0,005	0	15
Shcbp1	0	5,490163248	0,622	0,018	0	15
Cdca2	0	7,628824778	0,605	0,004	0	15
Nuf2	0	6,465363948	0,605	0,009	0	15
Kif23	0	6,467498992	0,599	0,008	0	15
Ska1	0	7,296228426	0,581	0,004	0	15
Depdc1a	0	6,292925917	0,576	0,007	0	15
Kifc1	0	6,314090427	0,564	0,01	0	15
Asf1b	0	5,739792913	0,564	0,013	0	15
Casc5	0	6,250763591	0,547	0,009	0	15
Tuba1c	0	6,463564133	0,547	0,016	0	15
Aurka	0	6,069855521	0,535	0,011	0	15
Cenpe	0	5,394163347	0,529	0,015	0	15
Hist1h2ae	0	6,840023465	0,523	0,01	0	15
Kif20a	0	5,766310543	0,523	0,01	0	15
Sgol1	0	6,181842393	0,512	0,007	0	15
Plk1	0	7,473694819	0,494	0,004	0	15
Bub1b	0	6,500938705	0,494	0,006	0	15
Cdca5	0	7,592604954	0,483	0,003	0	15
Psrc1	0	6,273111329	0,483	0,007	0	15
Bub1	0	7,392735445	0,477	0,003	0	15
Ncapg	0	6,527474563	0,471	0,004	0	15
Hmmr	0	6,068772131	0,471	0,006	0	15
Kif20b	0	5,511608008	0,477	0,012	0	15
Kif11	0	6,970764057	0,436	0,004	0	15

Sapcd2	0	6,112587443	0,436	0,008	0	15
Spag5	0	6,544820479	0,43	0,004	0	15
Kif15	0	6,802499934	0,407	0,003	0	15
Iqgap3	0	8,713123808	0,395	0,001	0	15
Aspm	0	6,433278132	0,384	0,005	0	15
Hist1h1b	0	7,706229544	0,331	0,002	0	15
E2f8	0	7,441764879	0,314	0,002	0	15
Fam83d	0	7,470557682	0,308	0,002	0	15
Cdkn3	3,0965E-304	5,474507217	0,436	0,01	4,969E-300	15
Rad51ap1	5,876E-301	5,253863961	0,494	0,014	9,4291E-297	15
Ect2	5,5713E-300	5,634238276	0,424	0,009	8,9402E-296	15
Cdc20	2,3038E-299	5,15049052	0,663	0,033	3,697E-295	15
Ankle1	9,6423E-297	7,601928181	0,279	0,001	1,5473E-292	15
Kif2c	7,3515E-294	5,480754345	0,395	0,007	1,1797E-289	15
Dlgap5	1,6111E-293	5,628111168	0,39	0,007	2,5853E-289	15
Tubb6	1,561E-291	4,908165909	0,837	0,066	2,5049E-287	15
Arhgef39	3,0431E-289	7,372946977	0,291	0,002	4,8832E-285	15
Knstrn	9,2684E-289	5,017054265	0,529	0,019	1,4873E-284	15
Cep55	2,3141E-287	5,94942796	0,366	0,006	3,7134E-283	15
Ttk	2,5838E-283	5,509388587	0,436	0,011	4,1462E-279	15
Ska3	5,325E-282	6,004979441	0,343	0,005	8,545E-278	15
Kif4	1,2743E-281	6,594288	0,297	0,003	2,0448E-277	15
Nek2	3,8606E-279	6,303937014	0,302	0,003	6,195E-275	15
Tacc3	5,9287E-272	4,935633001	0,68	0,042	9,5138E-268	15
Foxm1	2,0027E-263	5,416949479	0,384	0,009	3,2138E-259	15
Cenpa	1,8917E-261	4,836494467	0,703	0,048	3,0356E-257	15
Cenpf	6,0167E-260	5,275543672	0,744	0,057	9,655E-256	15
Ckap2	2,0977E-253	4,543826583	0,669	0,043	3,3662E-249	15
Trip13	1,5398E-250	5,101140758	0,436	0,014	2,471E-246	15
Sgol2a	4,8537E-248	6,349251532	0,308	0,005	7,7888E-244	15
Cdc25c	2,1893E-243	5,371072828	0,326	0,006	3,5131E-239	15
Ncaph	3,2658E-239	4,902540457	0,453	0,017	5,2406E-235	15
H2afx	4,462E-232	4,754266459	0,872	0,102	7,1601E-228	15
Cenpp	1,9628E-231	4,867005202	0,436	0,016	3,1497E-227	15
Cdkn2c	6,0435E-230	4,364320051	0,843	0,091	9,698E-226	15
Gmnn	6,6172E-223	4,230439076	0,634	0,044	1,0619E-218	15
Arhgap11a	1,2084E-219	5,633671947	0,302	0,006	1,9391E-215	15
Troap	1,7189E-218	6,501154613	0,256	0,003	2,7584E-214	15
Uhrf1	1,2856E-217	4,466081511	0,436	0,018	2,0631E-213	15
Ube2t	1,598E-214	4,903498892	0,477	0,023	2,5643E-210	15
Fbxo5	7,7065E-214	4,625095865	0,535	0,031	1,2367E-209	15
Mgat4c	0	7,626968286	0,989	0,017	0	16
Brinp3	0	6,179135621	0,851	0,031	0	16
Ucn3	0	10,42271637	0,819	0,003	0	16
Gabrg3	0	6,487823966	0,83	0,019	0	16
Cck	0	7,651700648	0,819	0,011	0	16
Klhl1	0	7,893877267	0,777	0,005	0	16
Cdh9	0	8,682356524	0,734	0,003	0	16

Pbx3	0	5,560335025	0,745	0,023	0	16
RP23-407N2.2	0	8,398928819	0,67	0,002	0	16
A930011G23Rik	0	8,044622819	0,543	0,003	0	16
Ecel1	0	6,405073697	0,543	0,008	0	16
Nmur2	0	8,614157694	0,479	0,001	0	16
Apela	0	9,075576356	0,468	0,001	0	16
Grm5	0	9,023223545	0,34	0,001	0	16
Vwc2l	0	9,001185742	0,287	0,001	0	16
March1	7,2713E-303	6,344765572	0,5	0,009	1,1668E-298	16
Amigo2	4,5015E-260	5,756946696	0,617	0,019	7,2236E-256	16
Nefl	1,0186E-258	5,755983062	0,787	0,036	1,6346E-254	16
Gm2694	4,7235E-255	5,168097933	0,809	0,039	7,5799E-251	16
Tmeff2	1,3304E-240	5,920696761	0,989	0,074	2,1349E-236	16
A330102I10Rik	1,4296E-240	5,817683708	0,574	0,018	2,2941E-236	16
Ctxn2	4,4963E-236	6,577973256	0,351	0,005	7,2152E-232	16
Enc1	7,8429E-236	5,270893306	0,532	0,015	1,2585E-231	16
Dgkg	1,3546E-235	5,093039041	0,723	0,033	2,1738E-231	16
Atp1a3	2,9936E-233	4,71569118	0,755	0,037	4,8039E-229	16
Car10	4,4697E-232	6,709944074	0,351	0,005	7,1725E-228	16
Avil	3,8359E-227	6,289776158	0,394	0,007	6,1554E-223	16
Ntrk3	5,0268E-213	4,372713836	0,936	0,07	8,0664E-209	16
Sema5a	5,7246E-208	5,128598919	0,468	0,013	9,1863E-204	16
Nrsn2	2,9017E-207	4,493620031	0,883	0,064	4,6564E-203	16
Cntnap2	7,0934E-201	4,720778016	0,713	0,039	1,1383E-196	16
Tmem130	2,8166E-198	4,397956024	0,936	0,077	4,5197E-194	16
Rspo3	1,1237E-188	5,610287421	0,404	0,011	1,8032E-184	16
Galnt13	4,8546E-186	5,017440735	0,521	0,021	7,7902E-182	16
Nefm	1,0255E-180	5,129898966	0,532	0,022	1,6457E-176	16
Dpp10	6,0853E-177	5,517715641	0,351	0,008	9,765E-173	16
Pcsk1	2,3105E-172	4,757640204	0,617	0,034	3,7076E-168	16
Fam19a1	8,6938E-159	4,341061656	0,989	0,124	1,3951E-154	16
Prune2	2,2633E-151	4,904383451	0,511	0,026	3,6319E-147	16
Kcna5	2,3942E-146	4,240299299	0,755	0,067	3,8419E-142	16
Serpini1	1,3104E-144	5,211552449	1	0,151	2,1027E-140	16
Etaa1os	2,8776E-134	4,56648769	0,511	0,029	4,6177E-130	16
Gpx3	4,2382E-133	3,738995981	1	0,151	6,801E-129	16
Pkib	7,8755E-132	5,98037505	0,319	0,01	1,2638E-127	16
Rasgef1b	2,4836E-130	5,250170733	0,277	0,007	3,9854E-126	16
Scgn	4,1309E-129	3,563563489	0,947	0,122	6,6289E-125	16
Snx7	1,0431E-122	3,804654641	0,862	0,111	1,6738E-118	16
Ddah1	3,9152E-122	3,860567638	1	0,183	6,2827E-118	16
Rit2	8,9115E-122	3,336595884	0,809	0,089	1,43E-117	16
Sncb	1,8342E-118	3,097885935	0,883	0,113	2,9434E-114	16
Faim2	6,7412E-117	3,532252853	0,798	0,097	1,0818E-112	16
Nrxn3	1,5068E-116	4,328080192	0,543	0,04	2,418E-112	16
Rgs17	1,4163E-115	3,767634053	0,862	0,121	2,2727E-111	16
B3glct	3,8045E-114	3,67669329	0,798	0,097	6,1051E-110	16
Rab27b	3,9151E-113	4,397956308	0,511	0,036	6,2826E-109	16

Chst8	5,0406E-112	3,752226459	0,628	0,057	8,0886E-108	16
Kif26b	3,9791E-110	4,416087577	0,362	0,017	6,3852E-106	16
2900011O08Rik	3,3991E-104	3,586778973	0,713	0,085	5,4545E-100	16
Kcnip4	3,3951E-99	4,524065243	0,362	0,02	5,44813E-95	16
P2ry1	2,62844E-97	4,396669648	0,479	0,037	4,21785E-93	16
Rprm	4,83687E-96	3,411905446	0,681	0,08	7,76173E-92	16
Dner	1,77001E-93	3,07630338	0,872	0,151	2,84034E-89	16
Cnih2	3,73264E-92	3,094101314	0,957	0,205	5,98976E-88	16
Resp18	3,79091E-91	3,293672029	1	0,254	6,08327E-87	16
AW551984	3,36114E-90	3,430305686	0,989	0,251	5,39362E-86	16
Snhg11	6,1679E-89	3,494718437	0,989	0,259	9,89763E-85	16
Sntg1	3,11709E-88	4,754512147	0,255	0,01	5,00199E-84	16
Caly	2,25823E-87	3,051063462	0,989	0,254	3,62379E-83	16
Adra2a	3,71168E-87	4,011026607	0,404	0,029	5,95613E-83	16
Spock3	9,86858E-87	3,356324259	1	0,291	1,58361E-82	16
Cyb561	2,43953E-86	3,807680025	0,606	0,072	3,91471E-82	16
Slitrk3	6,48001E-86	4,207415265	0,33	0,019	1,03985E-81	16
Jakmip1	8,04639E-86	3,423650109	0,617	0,075	1,2912E-81	16
Pcdh9	2,05967E-85	3,76610782	0,787	0,134	3,30516E-81	16
Ralyl	1,35122E-83	3,225524229	0,734	0,114	2,16831E-79	16
Id4	1,96209E-83	3,05346957	0,777	0,119	3,14857E-79	16
Pcp4	4,86117E-83	3,66513908	0,521	0,053	7,80072E-79	16
Sncg	1,36542E-81	3,591031993	1	0,319	2,19108E-77	16
Alcam	1,05003E-80	3,077298052	1	0,31	1,68499E-76	16
Cadm2	5,0493E-80	4,219138271	0,298	0,016	8,10261E-76	16
Cbarp	1,74396E-79	2,637843211	0,989	0,235	2,79853E-75	16
Syt9	7,46654E-78	3,36841069	0,553	0,064	1,19815E-73	16
Pnmal2	6,90415E-76	2,760974666	0,936	0,234	1,10791E-71	16
Pgm2l1	2,29358E-75	2,866849023	0,787	0,143	3,68051E-71	16
Tenm2	2,36283E-75	4,465868885	0,266	0,014	3,79164E-71	16
Celf6	2,79573E-75	2,71911083	0,968	0,259	4,48631E-71	16
A730017C20Rik	3,37229E-74	2,732361218	0,83	0,162	5,41152E-70	16
Phyhipl	1,43666E-73	3,185979474	0,723	0,126	2,30541E-69	16
Atp1b1	6,51799E-73	2,819953847	1	0,347	1,04594E-68	16
Dpf1	4,03462E-72	3,244082182	0,489	0,053	6,47436E-68	16
Tmem59l	7,72251E-72	2,749833288	0,702	0,116	1,23923E-67	16
Nrip3	5,24039E-70	3,833389926	0,309	0,021	8,40925E-66	16
Tagln3	5,34315E-70	2,801781599	1	0,382	8,57416E-66	16
Atp6v1g2	1,91881E-68	2,659191764	0,777	0,149	3,07912E-64	16
Hap1	1,92652E-68	2,636606551	0,968	0,314	3,09148E-64	16
Kcnk2	3,1813E-68	2,612753821	0,883	0,208	5,10503E-64	16
Ndrg4	8,27035E-68	2,616699321	0,947	0,268	1,32714E-63	16
Col8a1	1,06724E-67	3,127871951	0,33	0,024	1,7126E-63	16
Fam171b	4,29915E-67	2,637512949	0,915	0,236	6,89885E-63	16
Zcchc12	6,79592E-67	2,577033763	1	0,335	1,09054E-62	16
Gucy1b3	1,04898E-66	2,979926882	0,691	0,125	1,6833E-62	16
Ccnb2	6,6036E-220	4,92014843	0,722	0,034	1,0597E-215	17
Hist1h2bc	1,0228E-144	5,385378381	0,778	0,071	1,6413E-140	17

Cenpa	4,8705E-138	4,729922904	0,7	0,057	7,8157E-134	17
Kif20a	1,7972E-135	4,733633914	0,433	0,019	2,884E-131	17
Cenpe	7,9412E-134	4,598467204	0,467	0,023	1,2743E-129	17
Cdc25c	6,0383E-126	5,406998879	0,322	0,011	9,6897E-122	17
Hist1h1c	9,2225E-117	4,686112472	0,678	0,065	1,4799E-112	17
Cdc20	1,5027E-114	4,795617893	0,567	0,044	2,4114E-110	17
Racgap1	1,0474E-113	3,946506922	0,711	0,072	1,6807E-109	17
Cdca3	3,8344E-108	3,687250837	0,511	0,035	6,153E-104	17
Cdca8	1,0516E-105	4,001086257	0,533	0,041	1,6875E-101	17
Birc5	1,4633E-103	3,548261893	0,522	0,039	2,3482E-99	17
Hmmr	1,3434E-100	4,699272142	0,333	0,015	2,15571E-96	17
Ccnb1	5,84221E-94	3,906904866	0,389	0,023	9,375E-90	17
Ckap2l	8,40047E-92	4,196344721	0,389	0,024	1,34802E-87	17
Cdkn3	1,02483E-91	4,758161379	0,333	0,017	1,64455E-87	17
Ckap2	1,42415E-91	4,21697623	0,556	0,054	2,28534E-87	17
Tpx2	1,8215E-91	4,113031038	0,456	0,034	2,92296E-87	17
Knstrn	1,94328E-89	4,198756861	0,411	0,028	3,11838E-85	17
Lockd	2,19188E-88	3,656159945	0,689	0,089	3,51732E-84	17
Cep55	3,9313E-85	4,514042566	0,278	0,013	6,30856E-81	17
Kif2c	1,09145E-80	4,79493515	0,289	0,015	1,75145E-76	17
Cks2	1,10006E-75	4,07529907	0,578	0,072	1,76527E-71	17
Fam64a	7,09233E-68	4,048584877	0,344	0,026	1,13811E-63	17
Hist1h4i	1,4257E-62	4,116274025	0,289	0,02	2,28783E-58	17
Nuf2	6,88074E-54	3,640459482	0,289	0,023	1,10415E-49	17
Depdc1a	3,77155E-53	4,153139739	0,267	0,02	6,05221E-49	17
Cenpf	4,32336E-53	3,294994742	0,489	0,071	6,9377E-49	17
Pttg1	5,04172E-50	3,346302763	0,633	0,136	8,09046E-46	17
Hmgn2	2,24831E-48	2,299783144	0,989	0,691	3,60786E-44	17
H2afz	8,58063E-48	2,417341871	1	0,919	1,37693E-43	17
Kif23	1,95818E-47	3,688906969	0,267	0,022	3,14229E-43	17
Hmgb2	2,21951E-46	3,454905903	0,933	0,491	3,56165E-42	17
Hmgb1	3,18362E-45	1,52920136	1	0,991	5,10876E-41	17
Hsp90b1	3,074E-42	2,150367291	0,978	0,641	4,93285E-38	17
Nde1	7,73474E-42	2,956222521	0,622	0,149	1,24119E-37	17
Lgals1	1,77635E-41	2,168669722	0,989	0,754	2,85051E-37	17
Ccna2	8,41383E-41	2,99978624	0,3	0,032	1,35017E-36	17
Tubb6	2,47442E-39	2,925758471	0,467	0,083	3,9707E-35	17
Lmnb1	8,92119E-38	2,88111661	0,589	0,148	1,43158E-33	17
Mki67	9,02092E-36	2,90461385	0,356	0,053	1,44759E-31	17
Rdm1	1,44408E-33	3,347457532	0,356	0,058	2,31731E-29	17
Mns1	6,73331E-32	3,31179431	0,278	0,037	1,08049E-27	17
Tacc3	2,16684E-31	2,802341727	0,344	0,056	3,47713E-27	17
Ube2c	1,42604E-30	1,8170998	0,267	0,034	2,28837E-26	17
Mad2l1	2,64458E-30	2,875726825	0,333	0,054	4,24375E-26	17
Hmgn1	3,95065E-30	1,32863635	1	0,788	6,33961E-26	17
2700094K13Rik	9,16405E-30	2,476007142	0,744	0,343	1,47056E-25	17
Cep89	1,03074E-28	2,976433287	0,367	0,07	1,65404E-24	17
Cenpm	1,54666E-28	2,853704081	0,289	0,043	2,48192E-24	17

Rbm3	8,68298E-28	0,965538497	1	0,986	1,39336E-23	17
H2afv	3,63514E-27	1,937263594	0,778	0,38	5,83331E-23	17
Dbf4	9,16114E-27	3,081160853	0,256	0,036	1,47009E-22	17
Smc4	1,73366E-26	2,423978136	0,533	0,158	2,78201E-22	17
Vim	2,58137E-26	1,289929305	1	0,71	4,14233E-22	17
Pbk	4,81425E-26	2,054314592	0,278	0,041	7,72542E-22	17
Dbi	4,89907E-25	1,283534147	1	0,659	7,86153E-21	17
Lbr	8,36074E-25	2,777537491	0,433	0,109	1,34165E-20	17
Hint1	1,61026E-24	1,018333655	1	0,959	2,58398E-20	17
Morf4l2	2,63483E-24	1,072625339	1	0,873	4,22811E-20	17
Fbln1	9,00243E-24	1,821513785	0,811	0,358	1,44462E-19	17
Nucks1	5,94164E-23	2,019203061	0,733	0,404	9,53456E-19	17
Ptms	6,89023E-23	1,276147109	0,944	0,829	1,10568E-18	17
Tmpo	1,15672E-22	1,992260466	0,722	0,354	1,85619E-18	17
Tmed2	4,47839E-22	1,314147853	0,9	0,673	7,18647E-18	17
Ran	6,28012E-22	1,400043458	0,889	0,742	1,00777E-17	17
Postn	6,6694E-22	1,423944253	0,856	0,398	1,07024E-17	17
Tubb4b	1,06078E-21	2,192141868	0,767	0,494	1,70223E-17	17
Dynll1	3,17107E-21	1,145822985	1	0,97	5,08861E-17	17
Clic1	3,52878E-21	1,274894411	0,933	0,684	5,66263E-17	17
Psat1	2,04467E-20	1,995920286	0,444	0,126	3,28108E-16	17
Pfn1	3,82433E-20	0,855345275	1	0,937	6,13691E-16	17
Nup37	7,57136E-20	2,769706535	0,311	0,071	1,21498E-15	17
Snrpf	5,18265E-19	1,252509151	0,867	0,662	8,3166E-15	17
Anxa5	7,94976E-19	1,144146063	0,922	0,575	1,2757E-14	17
Tnfaip6	1,00553E-18	2,23575421	0,256	0,048	1,61358E-14	17
Lmna	1,07284E-18	1,561107618	0,822	0,607	1,72159E-14	17
2810417H13Rik	1,4315E-18	1,488317077	0,267	0,051	2,29713E-14	17
Rnf26	3,37681E-18	2,385512136	0,422	0,135	5,41877E-14	17
Srsf3	4,17972E-18	1,112307929	0,944	0,808	6,7072E-14	17
Rap1a	8,461E-18	1,460561397	0,8	0,487	1,35774E-13	17
Ptgr1	1,34458E-17	2,619073925	0,256	0,053	2,15764E-13	17
Rpn2	1,51961E-17	1,481615633	0,789	0,459	2,43852E-13	17
Ube2s	2,12343E-17	1,674397801	0,822	0,711	3,40747E-13	17
Marcks	1,63581E-16	1,073450853	0,967	0,722	2,62498E-12	17
Ccdc34	1,69828E-16	1,723235007	0,656	0,355	2,72524E-12	17
H3f3b	4,43906E-16	0,555449518	1	1	7,12336E-12	17
Hnrnpa1	5,65852E-16	0,841187084	0,989	0,857	9,08022E-12	17
Emp3	5,74567E-16	1,522370252	0,644	0,288	9,22008E-12	17
Hmgn3	7,29876E-16	1,362706691	0,778	0,585	1,17123E-11	17
Sept7	2,67457E-15	1,015371955	0,911	0,767	4,29188E-11	17
Odc1	3,57969E-15	2,028289923	0,489	0,204	5,74433E-11	17
Snrpg	3,89687E-15	0,94600587	0,944	0,799	6,25331E-11	17
2700060E02Rik	4,45171E-15	1,028479094	0,911	0,701	7,14366E-11	17
Calm3	4,64174E-15	1,140672073	0,867	0,74	7,4486E-11	17
Hnrnpu	4,94275E-15	1,224267518	0,8	0,562	7,93163E-11	17
Arf4	9,00739E-15	1,0013043	0,878	0,753	1,44542E-10	17
Rcn3	9,05171E-15	1,250246453	0,667	0,324	1,45253E-10	17

Serpinh1	2,39779E-14	0,947581776	0,911	0,563	3,84773E-10	17
Lsm5	2,65974E-14	1,435879622	0,689	0,422	4,26808E-10	17
Calu	3,37242E-14	1,280662245	0,667	0,337	5,41172E-10	17
mt-Co3	4,82309E-45	1,166857906	1	1	7,73962E-41	18
mt-Atp6	3,3802E-44	1,203124862	1	1	5,42421E-40	18
mt-Cytb	2,91961E-37	1,063008083	1	1	4,6851E-33	18
Malat1	3,20534E-37	1,390608036	1	1	5,1436E-33	18
mt-Co1	1,17474E-35	0,979996429	1	1	1,88511E-31	18
mt-Nd4	5,47361E-32	1,002138866	1	0,998	8,78351E-28	18
Meg3	6,38753E-32	1,933393142	1	0,844	1,02501E-27	18
mt-Nd1	2,46209E-30	0,984908994	1	1	3,95092E-26	18
mt-Co2	1,92058E-29	0,950282451	1	1	3,08195E-25	18
mt-Nd2	5,08041E-25	1,268354149	0,977	0,822	8,15254E-21	18
Smpd3	2,59423E-24	2,022411552	0,791	0,395	4,16296E-20	18
Ank2	3,45883E-21	1,996431883	0,814	0,485	5,55038E-17	18
Adrbk2	5,48021E-21	2,459260058	0,663	0,325	8,79409E-17	18
Elavl4	5,51452E-19	1,123125783	0,965	0,541	8,84914E-15	18
Snap25	4,35697E-18	1,284580545	0,907	0,512	6,99163E-14	18
Ppm1h	5,83563E-18	2,153563377	0,558	0,227	9,36444E-14	18
Rsrp1	2,80741E-17	1,0912923	0,977	0,797	4,50505E-13	18
Celf3	3,50727E-17	2,10749622	0,616	0,301	5,62811E-13	18
Tcf4	4,45267E-17	1,171482504	0,942	0,735	7,14521E-13	18
mt-Nd3	1,30777E-16	1,1674436	0,884	0,668	2,09857E-12	18
Scg2	2,77311E-16	1,218647377	0,884	0,49	4,45E-12	18
Cadm1	7,3183E-16	1,181343919	0,977	0,747	1,17437E-11	18
Celf4	1,48882E-15	1,621764171	0,744	0,406	2,38911E-11	18
Rtn1	2,31719E-15	0,932138409	1	0,699	3,71839E-11	18
Tshz2	2,8E-15	1,506641285	0,767	0,49	4,49316E-11	18
Stmn4	4,89486E-15	1,415455321	0,733	0,399	7,85478E-11	18
Hoxa5	6,04232E-15	0,993050539	0,977	0,654	9,69611E-11	18
App	1,56896E-14	0,985837359	0,977	0,865	2,51772E-10	18
Gsk3b	9,99424E-14	1,138318407	0,872	0,688	1,60378E-09	18
Ogt	1,36806E-13	1,720330837	0,698	0,478	2,19533E-09	18
Kif1b	2,55286E-13	1,741739077	0,663	0,444	4,09658E-09	18
Tns3	3,80559E-13	2,166762519	0,535	0,278	6,10683E-09	18
Syt1	1,08218E-12	1,487989458	0,698	0,43	1,73658E-08	18
Gpr22	1,20619E-12	1,930368976	0,535	0,275	1,93557E-08	18
Ttc3	1,95311E-12	0,975763774	0,965	0,736	3,13415E-08	18
Cd27	2,04866E-12	2,655406077	0,302	0,095	3,28749E-08	18
Klc1	3,70138E-12	1,042181747	0,849	0,586	5,93961E-08	18
Fgf13	2,44857E-11	0,955768976	0,895	0,55	3,92922E-07	18
Nfib	2,94547E-11	1,491533473	0,721	0,53	4,7266E-07	18
Dst	3,22104E-11	1,401657553	0,686	0,496	5,1688E-07	18
Atp1a1	3,6436E-11	1,11543567	0,837	0,637	5,84688E-07	18
Rbms3	4,08812E-11	1,302769996	0,698	0,483	6,5602E-07	18
Fus	5,09307E-11	0,872483404	0,942	0,769	8,17284E-07	18
Dpysl3	6,97382E-11	0,813610364	0,93	0,684	1,11909E-06	18
Dync1i2	7,41818E-11	0,837053596	0,86	0,706	1,1904E-06	18

Fxyd6	1,02908E-10	0,746365391	0,942	0,606	1,65137E-06	18
Thra	1,4712E-10	0,986892387	0,884	0,694	2,36083E-06	18
Gria2	2,25529E-10	2,255150964	0,442	0,209	3,61906E-06	18
Pcsk1n	3,10101E-10	0,437363415	1	0,69	4,97619E-06	18
Map1b	7,43038E-10	0,805327638	0,942	0,849	1,19235E-05	18
Ly6h	8,84683E-10	1,100543801	0,721	0,427	1,41965E-05	18
Ahi1	9,28083E-10	1,755589214	0,57	0,352	1,48929E-05	18
Cct7	9,63914E-10	0,829884059	0,919	0,748	1,54679E-05	18
Ube3a	1,43211E-09	1,242735017	0,686	0,508	2,2981E-05	18
Rbm39	1,54291E-09	0,699847303	0,977	0,886	2,47591E-05	18
Rgmb	1,66801E-09	1,299031726	0,686	0,483	2,67665E-05	18
Atp6v0b	3,41794E-09	0,854920472	0,884	0,683	5,48476E-05	18
Slc10a4	4,28149E-09	1,370455092	0,616	0,357	6,87051E-05	18
Gap43	4,823E-09	0,539381202	0,977	0,717	7,73946E-05	18
Eml5	5,30024E-09	2,089468971	0,384	0,18	8,50529E-05	18
Aplp1	7,36605E-09	1,063145662	0,686	0,43	0,000118203	18
Nsg1	7,88137E-09	0,83603716	0,86	0,608	0,000126472	18
Ddx5	1,14967E-08	0,648537391	0,988	0,969	0,000184488	18
Tubb2b	1,34871E-08	0,559215423	0,953	0,638	0,000216428	18
Nrep	1,39463E-08	1,113816314	0,709	0,533	0,000223796	18
Rgs4	1,90617E-08	1,032044546	0,535	0,278	0,000305884	18
Cox6a1	3,09849E-08	0,610974584	0,965	0,93	0,000497215	18
2010111I01Rik	3,24693E-08	1,348341327	0,593	0,419	0,000521034	18
Pcbp3	3,40017E-08	1,019440518	0,686	0,451	0,000545626	18
Huwe1	3,98244E-08	1,800121945	0,465	0,275	0,000639062	18
AY036118	6,06006E-08	1,098675959	0,663	0,535	0,000972458	18
Tubb2a	7,05834E-08	0,49073074	0,942	0,608	0,001132652	18
Xist	8,5683E-08	1,324369999	0,872	0,616	0,001374955	18
Stmn2	1,04464E-07	0,477462026	0,988	0,698	0,001676341	18
Nfix	1,04623E-07	1,115345619	0,686	0,501	0,00167888	18
Gdi1	1,10538E-07	0,975409335	0,733	0,587	0,001773802	18
Uchl1	1,10768E-07	0,475804639	0,965	0,696	0,001777491	18
Calm2	1,81391E-07	0,398482591	1	0,979	0,002910781	18
Diablo	2,27366E-07	0,756763986	0,837	0,649	0,003648547	18
Bub3	3,38661E-07	0,813037048	0,814	0,641	0,005434497	18
Hsp90aa1	3,40746E-07	0,625216408	1	0,895	0,005467956	18
Dbn1	3,63725E-07	0,854793175	0,744	0,606	0,005836691	18
Gm42418	3,66227E-07	0,640848285	0,895	0,735	0,005876846	18
Cacna2d1	3,9878E-07	1,350436189	0,5	0,31	0,006399222	18
Pik3r1	4,11897E-07	1,798639196	0,453	0,29	0,006609718	18
Nedd4	4,70365E-07	0,568201993	0,849	0,702	0,007547941	18
Sox11	6,44281E-07	1,391444796	0,512	0,341	0,010338771	18
Bex2	6,52293E-07	0,584548209	0,965	0,685	0,010467351	18
Shank1	8,46132E-07	1,896076317	0,314	0,143	0,013577878	18
Basp1	8,55782E-07	0,92290727	0,674	0,459	0,013732729	18
Phox2b	9,11914E-07	0,74864845	0,895	0,738	0,01463348	18
Myl1	9,15744E-07	0,920925135	0,593	0,345	0,014694947	18
Kif5c	1,10657E-06	1,483159848	0,477	0,309	0,017757108	18

Miat	1,19065E-06	1,995875608	0,279	0,124	0,0191064	18
Slit2	1,38154E-06	2,102879997	0,372	0,203	0,022169588	18
Cpe	2,07013E-06	0,646474742	0,907	0,658	0,03321933	18
Snhg11	2,50861E-06	1,247024327	0,465	0,268	0,040255675	18
Pcdh15	2,94389E-06	1,986641558	0,337	0,174	0,047240545	18
Hoxb5	3,67163E-06	0,6672923	0,744	0,541	0,058918614	18
Aplp2	3,68473E-06	0,832862493	0,756	0,666	0,059128891	18
Slc7a14	3,8571E-06	1,966967621	0,267	0,121	0,061894858	18
Mcm3	1,6429E-167	4,483307842	0,722	0,041	2,6364E-163	19
Mcm5	2,0398E-152	4,2713896	0,722	0,047	3,2733E-148	19
Uhrf1	3,1848E-138	4,737967954	0,506	0,023	5,1106E-134	19
Mcm2	1,9068E-116	4,020313303	0,759	0,073	3,0599E-112	19
Ung	1,0462E-114	4,381119272	0,544	0,034	1,6788E-110	19
Cdc6	9,9919E-109	5,193897068	0,253	0,006	1,6034E-104	19
Gins2	6,8389E-95	4,028436431	0,519	0,038	1,09744E-90	19
Tcf19	9,56127E-95	3,999278799	0,468	0,031	1,5343E-90	19
Mcm6	5,68106E-92	3,594291629	0,797	0,105	9,11639E-88	19
E2f1	3,10333E-89	4,215654719	0,481	0,035	4,97991E-85	19
Cdca7	4,48783E-87	3,707215683	0,519	0,041	7,20162E-83	19
Dtl	9,36475E-87	3,881484362	0,519	0,042	1,50276E-82	19
Chaf1b	4,3958E-81	4,05873722	0,443	0,033	7,05394E-77	19
Hells	9,49231E-74	3,976649261	0,418	0,032	1,52323E-69	19
Lig1	3,72524E-66	3,088715338	0,747	0,124	5,97789E-62	19
Rrm2	3,50822E-61	2,948636419	0,456	0,044	5,62965E-57	19
Dhfr	2,17142E-57	3,398832915	0,481	0,055	3,48448E-53	19
Gmnn	2,3598E-56	3,192977582	0,481	0,055	3,78677E-52	19
2810417H13Rik	7,17291E-50	3,203707865	0,43	0,049	1,15104E-45	19
Cenph	5,37819E-48	3,720148756	0,291	0,024	8,63039E-44	19
Itih5	8,19519E-47	1,951269953	0,987	0,278	1,31508E-42	19
Pde8a	1,5803E-46	2,775408773	0,532	0,078	2,53591E-42	19
Fen1	1,53839E-45	2,822799548	0,519	0,079	2,46865E-41	19
Cdt1	2,88371E-45	3,300538929	0,367	0,04	4,62749E-41	19
Tipin	9,62458E-45	2,451552018	0,848	0,253	1,54446E-40	19
Prim1	1,32989E-43	3,01560248	0,557	0,098	2,13408E-39	19
Matn2	2,05355E-43	2,128559087	0,861	0,222	3,29533E-39	19
Pcna	7,44094E-43	2,848042367	0,797	0,244	1,19405E-38	19
Fignl1	2,2041E-42	3,406029388	0,329	0,034	3,53692E-38	19
Mcm4	4,12245E-42	2,891527194	0,468	0,068	6,6153E-38	19
Galnt18	1,12199E-41	2,52832121	0,519	0,081	1,80046E-37	19
Ccne2	1,78422E-41	3,619581913	0,253	0,021	2,86313E-37	19
Pou3f1	9,36182E-39	2,388563875	0,532	0,09	1,50229E-34	19
Top2a	1,00007E-38	1,765291886	0,367	0,043	1,60481E-34	19
Fam111a	2,93961E-38	3,121715929	0,367	0,047	4,7172E-34	19
Mmp2	1,2204E-37	2,008667999	0,886	0,28	1,95837E-33	19
Cdca7l	4,59711E-37	3,032936639	0,329	0,038	7,37699E-33	19
Tk1	8,62435E-37	3,210207098	0,266	0,025	1,38395E-32	19
Rpa2	1,16075E-36	2,659340905	0,582	0,122	1,86266E-32	19
Hmgb2	2,24898E-36	1,448944149	0,987	0,491	3,60893E-32	19

Gltp	1,2418E-35	2,108259389	0,759	0,194	1,99272E-31	19
Lmnb1	3,75883E-35	2,395389068	0,633	0,148	6,03179E-31	19
Rrad	3,16161E-34	2,327186037	0,57	0,117	5,07344E-30	19
Ccnd1	3,27144E-34	1,965627784	0,81	0,231	5,24968E-30	19
Hat1	6,56863E-34	2,3503537	0,646	0,154	1,05407E-29	19
Timeless	2,45155E-33	3,153263323	0,266	0,028	3,93401E-29	19
Slbp	1,12652E-32	2,083066554	0,797	0,28	1,80773E-28	19
Mcm7	4,97495E-32	2,392659572	0,722	0,22	7,9833E-28	19
Asf1b	8,4041E-32	3,182301403	0,253	0,027	1,34861E-27	19
Tpm4	1,36614E-31	1,826310206	0,949	0,426	2,19224E-27	19
Col6a1	3,55728E-31	2,00335385	0,608	0,136	5,70837E-27	19
Postn	3,80886E-31	1,605944627	0,975	0,397	6,11208E-27	19
Cdk2	8,74987E-31	2,456756964	0,443	0,076	1,40409E-26	19
Dbi	1,01733E-30	1,50098572	1	0,659	1,63251E-26	19
Fam210b	1,87727E-30	2,495732338	0,43	0,074	3,01245E-26	19
Spc24	2,47948E-30	2,380596804	0,354	0,052	3,97883E-26	19
Col20a1	3,08343E-30	1,607369434	0,861	0,272	4,94798E-26	19
Pold1	1,98153E-29	2,996325512	0,342	0,052	3,17976E-25	19
Tubb6	2,74377E-29	1,93635638	0,456	0,084	4,40293E-25	19
Dnajc9	3,07297E-29	2,185688479	0,684	0,207	4,9312E-25	19
Tuba1b	6,04232E-29	1,095736614	1	0,949	9,69611E-25	19
Tagln2	6,47624E-29	1,234267754	1	0,869	1,03924E-24	19
Gulp1	9,85802E-29	1,86715796	0,759	0,242	1,58192E-24	19
Gja1	1,03683E-28	2,574399251	0,38	0,062	1,6638E-24	19
Chaf1a	3,73942E-28	2,764613729	0,278	0,036	6,00065E-24	19
Syce2	6,00858E-28	2,366216129	0,443	0,089	9,64197E-24	19
Cenpm	7,08498E-28	2,750471768	0,304	0,043	1,13693E-23	19
Nrn1	1,18659E-27	2,323910574	0,494	0,104	1,90412E-23	19
Rfc5	1,83641E-27	2,716031553	0,405	0,076	2,94689E-23	19
Pkmyt1	2,12261E-27	3,053624687	0,316	0,048	3,40616E-23	19
Pdlim4	2,2919E-27	1,903100715	0,696	0,212	3,67781E-23	19
Apitd1	4,91496E-27	3,19627476	0,291	0,041	7,88704E-23	19
Cdk1	5,91267E-27	1,849365677	0,304	0,042	9,48806E-23	19
Siva1	1,50256E-26	2,20000715	0,684	0,225	2,41116E-22	19
Rfc3	2,8467E-26	2,25428816	0,57	0,151	4,56809E-22	19
Fbln1	3,7367E-26	1,408633646	0,924	0,358	5,99629E-22	19
Rfc4	2,49419E-25	2,466179617	0,418	0,085	4,00243E-21	19
Tnc	2,51854E-25	2,049901668	0,456	0,093	4,0415E-21	19
Loxl2	2,63198E-25	2,350094869	0,418	0,083	4,22354E-21	19
Tyms	1,01413E-24	2,486490149	0,43	0,097	1,62737E-20	19
Mcam	1,07462E-24	1,618431768	0,785	0,276	1,72444E-20	19
Igfbp3	1,41453E-24	2,069024403	0,443	0,09	2,26989E-20	19
Tmpo	3,57146E-24	1,401467399	0,873	0,353	5,73113E-20	19
Sipa1	5,41662E-24	2,139479076	0,443	0,094	8,69205E-20	19
Il1rap	1,09168E-23	1,769690895	0,519	0,12	1,75182E-19	19
Tnfrsf12a	1,21048E-23	2,382780033	0,392	0,077	1,94246E-19	19
Irf6	1,29374E-23	2,293457295	0,367	0,067	2,07606E-19	19
Pdlim1	2,84228E-23	1,681861387	0,797	0,341	4,56101E-19	19

Copz2	4,1773E-23	2,018991922	0,532	0,129	6,70331E-19	19
Vcan	1,09284E-22	2,358056864	0,354	0,066	1,75369E-18	19
Nav2	2,34254E-22	1,844053035	0,494	0,124	3,75908E-18	19
Ezh2	5,31141E-22	1,971056899	0,519	0,137	8,52322E-18	19
Myl12a	6,52416E-22	1,238793668	0,987	0,577	1,04693E-17	19
Mtap	1,45639E-21	2,223935052	0,418	0,094	2,33706E-17	19
Emp3	2,96292E-21	1,564072197	0,785	0,287	4,7546E-17	19
Hnrnpf	3,44073E-21	1,342181606	0,975	0,629	5,52133E-17	19
Ppic	3,99132E-21	1,48930484	0,848	0,38	6,40487E-17	19
Clec14a	4,11175E-21	1,994686618	0,506	0,135	6,59813E-17	19
Ranbp1	5,17168E-21	1,255867908	0,949	0,721	8,299E-17	19
Ldb2	6,87812E-21	1,602540775	0,671	0,214	1,10373E-16	19
Metrn	8,93904E-21	1,265691881	0,962	0,438	1,43445E-16	19
Neurod6	0	8,188044506	1	0,011	0	20
AI593442	0	7,303022251	0,872	0,014	0	20
Adcyap1	3,4161E-289	5,756467933	0,59	0,012	5,4818E-285	20
Nrip3	1,3887E-269	5,976307844	0,641	0,017	2,2285E-265	20
Sct	1,5778E-246	7,749133377	0,538	0,013	2,5319E-242	20
Pcp4	1,7073E-227	6,303610386	0,897	0,049	2,7398E-223	20
Rtn4rl1	4,132E-214	5,206163471	0,615	0,021	6,6307E-210	20
Galr2	1,3213E-213	6,782137648	0,295	0,003	2,1202E-209	20
Gna14	4,4355E-207	5,832664518	0,41	0,008	7,1176E-203	20
Ebf1	8,8305E-205	4,973648403	0,821	0,045	1,417E-200	20
Rprml	1,1001E-180	4,998205524	0,987	0,084	1,7653E-176	20
Rmst	1,7447E-169	4,751266668	0,59	0,025	2,7997E-165	20
Slitrk4	7,9751E-161	4,835938447	0,487	0,017	1,2798E-156	20
Tmc3	7,9182E-151	4,244091537	0,679	0,04	1,2706E-146	20
Sstr2	1,322E-149	4,654034733	0,718	0,047	2,1213E-145	20
Kcnd2	2,7147E-149	4,243400588	0,859	0,071	4,3562E-145	20
Ltk	4,7388E-149	4,443944936	0,615	0,033	7,6044E-145	20
Tpbg	3,1296E-136	4,876535003	0,474	0,02	5,0221E-132	20
Lancl3	1,1218E-128	4,469311264	0,474	0,022	1,8002E-124	20
Asic2	1,1722E-124	3,979293308	0,949	0,115	1,8811E-120	20
Nefl	3,28781E-94	2,84434667	0,551	0,042	5,27594E-90	20
Synpo2	2,25088E-92	4,495679995	0,321	0,014	3,61199E-88	20
Camk2a	3,9709E-91	3,890034509	0,564	0,048	6,3721E-87	20
Synpr	1,04398E-86	5,060941922	0,397	0,024	1,67528E-82	20
Plch2	2,68613E-85	4,15768817	0,397	0,024	4,31043E-81	20
Thy1	3,79701E-84	4,254984778	0,667	0,077	6,09306E-80	20
Etv1	9,33927E-84	3,161785517	1	0,208	1,49867E-79	20
Tceal6	1,79037E-83	3,396927188	0,782	0,107	2,873E-79	20
Rasgrf2	4,80508E-82	4,608713202	0,256	0,01	7,7107E-78	20
Rbp4	3,77507E-79	4,604997088	0,385	0,025	6,05786E-75	20
Ttc39b	1,86707E-78	3,963461492	0,397	0,026	2,99608E-74	20
Vat1l	1,63859E-75	3,308660013	0,962	0,218	2,62944E-71	20
Cntn1	1,26354E-74	3,119635436	0,859	0,152	2,02761E-70	20
Snca	1,56774E-74	3,719901205	1	0,278	2,51575E-70	20
Gal	1,72906E-73	6,457178163	1	0,318	2,77462E-69	20

Tmem130	2,09332E-73	3,24500812	0,667	0,084	3,35916E-69	20
Klc3	2,71414E-73	3,571762945	0,603	0,069	4,35538E-69	20
Fibcd1	8,20927E-73	3,491337186	0,526	0,051	1,31734E-68	20
A730017C20Rik	3,58981E-72	3,174199684	0,872	0,163	5,76057E-68	20
St18	7,74338E-71	3,368517982	0,551	0,057	1,24258E-66	20
Hpcal1	2,07307E-70	3,235888007	0,897	0,184	3,32665E-66	20
Tuba4a	5,51233E-70	4,574445283	0,295	0,016	8,84563E-66	20
Fam131c	5,08273E-67	4,158308259	0,346	0,023	8,15625E-63	20
Gng4	3,5634E-66	3,02402654	0,782	0,138	5,71819E-62	20
Cntnap2	3,88668E-66	3,218669441	0,474	0,044	6,23696E-62	20
Syne1	2,44967E-65	3,517980288	0,551	0,063	3,93099E-61	20
Bdnf	1,48266E-64	4,196331607	0,269	0,014	2,37922E-60	20
Spock1	2,9825E-63	3,470364953	0,513	0,056	4,78602E-59	20
Emb	5,07362E-63	3,644330801	0,436	0,041	8,14165E-59	20
Fam89a	9,14381E-63	2,817798049	0,821	0,155	1,46731E-58	20
Grid2	1,11216E-62	3,077986845	0,628	0,083	1,78468E-58	20
Gng8	6,36536E-62	3,276371177	0,551	0,065	1,02145E-57	20
Tesc	4,7884E-60	3,473316562	0,538	0,066	7,68394E-56	20
Resp18	8,14893E-60	3,346597846	0,962	0,257	1,30766E-55	20
Snhg11	1,08071E-59	2,758770617	0,962	0,262	1,73422E-55	20
Cacng3	1,96181E-58	3,862422842	0,333	0,025	3,14811E-54	20
Moxd1	2,94031E-58	2,756478531	0,628	0,088	4,71832E-54	20
Tspan13	3,20354E-58	2,668804101	0,987	0,316	5,14072E-54	20
Cplx2	1,78308E-57	2,816952239	0,859	0,189	2,86131E-53	20
Plekhb2	1,20951E-56	2,810216373	0,808	0,17	1,9409E-52	20
Cnst	1,56984E-56	3,186900894	0,474	0,052	2,51912E-52	20
Synm	6,27618E-56	3,105682535	0,59	0,084	1,00714E-51	20
Cntnap5a	8,74616E-56	2,738536517	0,667	0,107	1,4035E-51	20
Pcp4l1	1,15074E-54	2,805953595	0,256	0,015	1,8466E-50	20
1500009L16Rik	1,26798E-54	2,538159568	0,859	0,197	2,03474E-50	20
Pcdh17	1,86587E-54	2,739821772	0,833	0,181	2,99417E-50	20
Lynx1	4,02761E-54	3,760494953	0,282	0,019	6,4631E-50	20
Trib2	1,85181E-53	2,518467885	0,885	0,219	2,9716E-49	20
Cystm1	3,82855E-53	2,46643508	0,962	0,297	6,14368E-49	20
Ppp1r14a	1,02206E-52	3,497506796	0,321	0,025	1,64011E-48	20
Pid1	5,48478E-52	2,670580568	0,692	0,127	8,80143E-48	20
Sybu	6,71275E-52	3,585152581	0,346	0,031	1,0772E-47	20
B630019K06Rik	1,13644E-51	2,687441219	0,756	0,155	1,82364E-47	20
Arpp21	1,75758E-50	2,590168366	0,782	0,175	2,8204E-46	20
Rgs17	2,21048E-50	2,537327062	0,692	0,126	3,54715E-46	20
Clmp	2,87526E-50	2,96191901	0,551	0,083	4,61392E-46	20
Kitl	5,19852E-50	3,096252036	0,397	0,041	8,34206E-46	20
Pcsk1n	9,63808E-50	2,924144594	1	0,691	1,54662E-45	20
Aplp1	1,21185E-49	2,413625581	1	0,427	1,94466E-45	20
Chchd10	1,38358E-48	2,540594478	0,872	0,227	2,22023E-44	20
Pgm2l1	2,10142E-48	2,72851001	0,718	0,146	3,37214E-44	20
Fkbp1b	9,56998E-48	2,903644124	0,769	0,178	1,53569E-43	20
Gpx3	9,69494E-48	2,591468774	0,744	0,157	1,55575E-43	20

Stxbp1	1,98594E-47	2,25730002	0,962	0,303	3,18684E-43	20
Nap1l5	2,72825E-47	2,175418065	0,987	0,315	4,37802E-43	20
Serpini1	8,93402E-47	1,744567795	0,756	0,157	1,43364E-42	20
Ccdc184	2,13592E-46	2,66735346	0,603	0,106	3,4275E-42	20
Sncg	3,46138E-46	2,232046081	0,974	0,321	5,55448E-42	20
Acot7	6,17329E-46	2,412971317	0,962	0,338	9,90627E-42	20
Ncoa7	9,22884E-46	2,689003	0,769	0,194	1,48095E-41	20
Celf6	1,42762E-45	2,153342026	0,91	0,262	2,2909E-41	20
Gm13889	1,55569E-45	2,322652162	0,744	0,151	2,49641E-41	20
Camk4	2,78165E-45	2,761638636	0,59	0,102	4,46372E-41	20
Plxnc1	2,88806E-45	3,705337612	0,282	0,023	4,63446E-41	20
Tbc1d9	3,2375E-45	3,214548489	0,397	0,046	5,19522E-41	20
Tmem255b	3,41541E-45	2,108304221	0,91	0,253	5,48071E-41	20
Sept6	8,86725E-45	2,464444998	0,795	0,201	1,42293E-40	20
Ache	1,46011E-44	2,571012647	0,91	0,306	2,34304E-40	20
Kcnq3	8,50975E-44	2,397821546	0,705	0,147	1,36556E-39	20
Cbln4	9,8206E-44	3,875806219	0,256	0,02	1,57591E-39	20
Bex2	3,24035E-43	1,833140834	1	0,685	5,19979E-39	20
Htr2b	0	6,953547573	0,803	0,011	0	21
Gda	0	6,456793873	0,803	0,015	0	21
Adgrg2	4,7048E-188	5,633074805	0,434	0,01	7,5498E-184	21
Grem2	2,5776E-187	5,4728582	0,434	0,01	4,1363E-183	21
Kctd8	6,2772E-168	5,539974747	0,368	0,008	1,0073E-163	21
Lrfn5	4,3317E-165	4,376414223	0,724	0,04	6,9511E-161	21
Kcnip4	1,6112E-154	4,704441312	0,5	0,019	2,5856E-150	21
Unc5d	2,5978E-150	4,947183719	0,382	0,01	4,1688E-146	21
Sphkap	1,3601E-142	4,256565146	0,684	0,042	2,1825E-138	21
Rasgrf2	1,0031E-133	5,553372565	0,329	0,009	1,6097E-129	21
Bace2	3,8619E-132	4,934193836	0,342	0,01	6,1973E-128	21
Penk	8,7376E-117	5,345885088	0,921	0,107	1,4021E-112	21
Tac1	1,4569E-111	5,717660713	0,987	0,158	2,338E-107	21
Necab2	4,7342E-103	3,467200361	0,908	0,116	7,597E-99	21
Csmd3	2,6439E-102	3,990859659	0,539	0,036	4,24263E-98	21
Skap1	3,71781E-97	5,385503236	0,263	0,008	5,96596E-93	21
Gpx3	1,06066E-94	3,891981902	0,961	0,155	1,70204E-90	21
Epha5	1,21716E-94	3,358868176	0,868	0,112	1,95318E-90	21
Lrp1b	1,20074E-93	4,044651277	0,474	0,03	1,92683E-89	21
Gch1	3,87209E-91	3,705065239	0,868	0,122	6,21354E-87	21
Sncb	1,11743E-90	3,450770299	0,868	0,115	1,79314E-86	21
Lingo2	2,16583E-86	3,89644764	0,526	0,041	3,47551E-82	21
Prom1	2,20022E-85	4,863034518	0,25	0,008	3,5307E-81	21
Ndst4	6,31056E-85	3,275484151	0,868	0,127	1,01266E-80	21
Cyb561	5,52269E-84	3,331843044	0,684	0,073	8,86226E-80	21
Pcp4	7,60661E-84	2,862320776	0,592	0,054	1,22063E-79	21
Nrsn2	1,63043E-83	3,595758999	0,658	0,069	2,61635E-79	21
Grm7	6,74945E-76	3,825757734	0,474	0,038	1,08308E-71	21
Stac	1,38249E-75	3,631431839	0,474	0,038	2,21848E-71	21
Cd79a	6,7981E-71	4,352166896	0,368	0,024	1,09089E-66	21

Gucy1a3	1,35626E-70	3,431913971	0,645	0,079	2,17639E-66	21
Rprm	2,65345E-69	3,563911524	0,658	0,083	4,25799E-65	21
BC030500	4,8626E-67	4,251020243	0,329	0,021	7,80301E-63	21
Tm4sf4	1,10466E-66	3,320127426	0,974	0,241	1,77264E-62	21
Tpd52l1	2,56961E-66	3,184761303	0,711	0,1	4,12345E-62	21
Colq	1,14955E-62	3,474033804	0,447	0,041	1,84469E-58	21
Fam183b	2,36757E-61	3,676560708	0,395	0,033	3,79925E-57	21
Sncg	2,08931E-60	3,120258021	1	0,321	3,35271E-56	21
Dcaf12l1	3,14979E-59	2,955196713	0,658	0,094	5,05447E-55	21
Ccdc109b	4,01177E-59	3,310193096	0,5	0,054	6,43768E-55	21
Chchd10	3,16601E-58	3,183593004	0,908	0,227	5,0805E-54	21
Tmem255a	3,44482E-58	3,97631131	0,289	0,018	5,5279E-54	21
Syt6	1,08416E-57	3,05695895	0,539	0,064	1,73975E-53	21
Faim2	1,9036E-57	2,978983326	0,671	0,101	3,0547E-53	21
Rab3b	1,53089E-56	2,68365309	0,829	0,166	2,45661E-52	21
Rit2	1,61838E-56	2,728209207	0,658	0,094	2,59701E-52	21
Tmem59l	3,92608E-56	2,901989442	0,711	0,118	6,30018E-52	21
Nrsn1	4,15907E-56	2,85812273	0,974	0,274	6,67407E-52	21
Rogdi	9,27924E-54	3,062096612	0,842	0,196	1,48904E-49	21
Mt3	1,67382E-52	2,835526475	0,737	0,134	2,68599E-48	21
Resp18	2,13757E-52	2,70677677	0,961	0,257	3,43017E-48	21
Skap2	3,99766E-52	2,981336415	0,921	0,298	6,41504E-48	21
Atp1a3	9,5802E-52	3,515777163	0,421	0,044	1,53733E-47	21
Atp1b1	1,72578E-51	2,471034401	1	0,349	2,76936E-47	21
A730017C20Rik	3,91576E-51	2,439684373	0,816	0,164	6,28362E-47	21
Lix1	4,81974E-51	2,386932502	1	0,318	7,73424E-47	21
Chgb	2,21607E-50	2,46320099	0,987	0,305	3,55613E-46	21
Grik1	6,1305E-50	3,30981779	0,395	0,04	9,83762E-46	21
Dok5	2,32387E-49	3,516589539	0,382	0,038	3,72912E-45	21
Sybu	7,1173E-49	3,55781781	0,342	0,031	1,14211E-44	21
Pcsk1	3,64369E-48	3,346909258	0,382	0,039	5,84703E-44	21
Il23a	4,75834E-48	3,978727809	0,25	0,017	7,63571E-44	21
Prkcb	8,12414E-48	2,451748209	0,789	0,164	1,30368E-43	21
Nap1l5	6,34801E-47	2,714070959	0,934	0,316	1,01867E-42	21
Tmie	8,70563E-46	3,299018386	0,382	0,041	1,39699E-41	21
Rgs17	1,1641E-45	2,621289918	0,671	0,126	1,86803E-41	21
Gpsm3	1,80424E-45	2,842829594	0,658	0,118	2,89526E-41	21
Gria1	1,35754E-44	3,455265646	0,289	0,024	2,17845E-40	21
Khdrbs2	1,93043E-44	2,73372357	0,526	0,077	3,09777E-40	21
Psd3	6,53127E-44	3,143211318	0,395	0,044	1,04807E-39	21
Ptchd1	8,41806E-44	3,530893961	0,289	0,024	1,35085E-39	21
Dmtn	1,25248E-43	2,637602974	0,618	0,108	2,00985E-39	21
Parvb	1,83528E-42	2,566178859	0,711	0,146	2,94508E-38	21
Prph	1,54235E-41	2,13896339	1	0,491	2,47502E-37	21
Caly	1,70143E-41	2,185418571	0,895	0,257	2,73029E-37	21
Gucy1b3	2,36695E-41	2,42233102	0,658	0,128	3,79825E-37	21
Spock1	4,62204E-41	2,906206505	0,434	0,057	7,41698E-37	21
Hpcal4	6,84937E-41	3,080977094	0,355	0,039	1,09912E-36	21

Chrm1	1,26338E-40	3,047911452	0,382	0,044	2,02735E-36	21
Runx1t1	1,32257E-40	2,760082991	0,434	0,056	2,12232E-36	21
Rwdd3	1,33096E-40	3,242940696	0,434	0,058	2,13579E-36	21
Snhg11	1,52425E-40	1,986599185	0,934	0,263	2,44596E-36	21
Casz1	1,55929E-39	2,655690626	0,645	0,131	2,5022E-35	21
Smyd3	1,65895E-39	2,770189615	0,618	0,118	2,66211E-35	21
Clu	2,73621E-39	3,07026767	0,461	0,067	4,39079E-35	21
Sgpp2	2,75039E-39	3,060755575	0,382	0,047	4,41355E-35	21
Ndrg4	6,88991E-39	2,221248829	0,882	0,271	1,10562E-34	21
Fstl5	1,37465E-38	2,539323993	0,711	0,161	2,2059E-34	21
Pcsk1n	1,423E-38	2,184323291	1	0,691	2,28348E-34	21
Abca5	2,24389E-38	2,910953144	0,526	0,089	3,60077E-34	21
Mdga2	3,58398E-38	2,782094109	0,447	0,064	5,75121E-34	21
Tubb2a	6,84693E-38	1,902394486	1	0,607	1,09873E-33	21
Edil3	8,77515E-38	2,647138041	0,526	0,088	1,40815E-33	21
Bend6	1,33878E-37	3,226130214	0,342	0,04	2,14834E-33	21
Slc5a7	1,95702E-37	2,439475409	0,763	0,197	3,14043E-33	21
Cacna1e	3,96748E-37	2,716866352	0,5	0,082	6,36661E-33	21
Aldoa	4,28824E-37	1,824359216	1	0,755	6,88134E-33	21
Gnai1	7,68014E-37	2,401199094	0,711	0,163	1,23243E-32	21
Nell1	2,04514E-36	3,387763056	0,263	0,024	3,28184E-32	21
Asns	2,39296E-36	2,6125624	0,645	0,146	3,83998E-32	21
Tox2	2,7629E-36	2,533922019	0,526	0,088	4,43363E-32	21
Fgl2	2,0639E-136	6,704316448	0,304	0,006	3,3119E-132	22
Ctsc	1,1381E-118	5,575785459	0,304	0,008	1,8263E-114	22
Col1a1	1,281E-111	6,395045563	0,464	0,023	2,0557E-107	22
Col1a2	2,1549E-111	4,991290713	0,783	0,072	3,458E-107	22
Mpz	1,9976E-105	4,705005849	0,565	0,036	3,2055E-101	22
Gfra3	1,09381E-98	5,753066647	0,275	0,008	1,75524E-94	22
Egfl8	1,00278E-91	4,789698737	0,638	0,057	1,60915E-87	22
Col14a1	1,20594E-90	4,831035354	0,377	0,018	1,93518E-86	22
Fam198b	1,63899E-90	3,903410684	0,667	0,061	2,63009E-86	22
Prss23	2,23496E-85	4,329318125	0,507	0,037	3,58644E-81	22
Plekha4	1,54461E-80	3,876336767	0,522	0,041	2,47864E-76	22
Sfrp5	1,73647E-80	3,460357403	0,884	0,13	2,78651E-76	22
Fam101a	1,41816E-78	5,634395692	0,261	0,01	2,27572E-74	22
Vwa1	6,13667E-67	5,404521302	0,391	0,028	9,84751E-63	22
Sfrp1	1,59222E-66	3,450523119	0,87	0,158	2,55504E-62	22
Rxrg	7,20144E-63	4,320053493	0,29	0,015	1,15561E-58	22
Matn2	1,27679E-59	3,039748621	0,957	0,222	2,04887E-55	22
Gatm	3,52096E-52	3,328058085	0,884	0,234	5,65009E-48	22
Sostdc1	1,3844E-50	3,200492076	0,812	0,172	2,22154E-46	22
Col3a1	1,68389E-50	3,327660376	0,971	0,304	2,70213E-46	22
Kcna1	9,77846E-50	3,310846173	0,58	0,078	1,56915E-45	22
Igfbp7	5,13477E-44	2,462069964	0,841	0,208	8,23976E-40	22
Aspa	2,27798E-42	2,745532061	0,609	0,102	3,65547E-38	22
Hbegf	2,19529E-40	3,24222638	0,551	0,092	3,52278E-36	22
Hmgcs2	1,29461E-39	2,583355146	0,652	0,124	2,07746E-35	22

Capg	5,57852E-38	2,821802964	0,58	0,102	8,95185E-34	22
Postn	6,68012E-38	2,282685805	0,986	0,398	1,07196E-33	22
Mrgprf	8,72274E-38	3,283731577	0,304	0,029	1,39974E-33	22
Arpc1b	2,73659E-35	2,044899493	0,986	0,531	4,39141E-31	22
Sparc	3,26992E-35	1,702684973	1	0,803	5,24723E-31	22
Dbi	3,35899E-35	1,84172352	1	0,66	5,39017E-31	22
Fbln5	5,74045E-34	2,297291008	0,87	0,276	9,2117E-30	22
Mal	4,52375E-33	3,035376659	0,449	0,07	7,25927E-29	22
Itih5	1,77961E-31	1,995813039	0,884	0,28	2,85574E-27	22
Olfml2a	5,3747E-31	2,413734901	0,652	0,145	8,62477E-27	22
Nrn1	7,15803E-30	2,331124582	0,536	0,105	1,14865E-25	22
Olfml2b	9,53769E-29	3,893744842	0,304	0,039	1,53051E-24	22
Acot1	5,79442E-28	1,983070951	0,797	0,249	9,2983E-24	22
Plp1	5,98715E-28	1,611432841	1	0,45	9,60758E-24	22
Sparcl1	1,67282E-27	3,538977078	0,348	0,052	2,68438E-23	22
Vim	3,07037E-25	1,423598269	1	0,711	4,92702E-21	22
Col18a1	5,90862E-24	1,561302096	0,971	0,409	9,48156E-20	22
Fign	6,21102E-24	2,038908365	0,594	0,147	9,96682E-20	22
Tspan15	1,16806E-23	1,847864439	0,768	0,257	1,87438E-19	22
Hspg2	1,25629E-23	2,227702444	0,493	0,109	2,01597E-19	22
Anxa2	1,56721E-23	2,074374304	0,826	0,349	2,51491E-19	22
Pmp22	2,15845E-23	1,703931761	0,928	0,552	3,46366E-19	22
Ifitm3	3,46471E-22	1,531185266	0,783	0,264	5,55983E-18	22
Col6a3	8,66861E-22	2,57000126	0,319	0,053	1,39105E-17	22
Nid2	1,37703E-21	3,053169308	0,29	0,045	2,20972E-17	22
Sema3b	1,09266E-20	2,022779355	0,681	0,237	1,7534E-16	22
Rcn1	1,23458E-20	1,53730817	0,826	0,32	1,98113E-16	22
Pdlim4	1,76837E-20	1,716556697	0,681	0,213	2,8377E-16	22
Col15a1	1,82115E-20	2,555924006	0,377	0,075	2,9224E-16	22
Col26a1	4,72517E-20	2,768422104	0,362	0,072	7,58248E-16	22
Lpar1	1,00974E-19	1,385942008	0,913	0,391	1,62033E-15	22
Tnc	1,40146E-19	2,275249326	0,42	0,094	2,24892E-15	22
Rap1a	2,14123E-19	1,50950482	0,913	0,486	3,43603E-15	22
Anxa5	7,70931E-19	1,458908789	0,957	0,576	1,23711E-14	22
Cnn3	8,14895E-19	1,245732434	0,971	0,757	1,30766E-14	22
Cnp	1,3366E-18	1,354646564	0,971	0,554	2,14484E-14	22
Serpine2	1,39007E-18	1,217322501	1	0,64	2,23064E-14	22
Slc1a5	3,52332E-18	2,742499757	0,275	0,048	5,65388E-14	22
Nid1	6,40486E-18	1,627207788	0,739	0,283	1,02779E-13	22
Sep15	9,27605E-18	1,023471892	1	0,851	1,48853E-13	22
Lamb1	1,37953E-17	1,85807108	0,478	0,125	2,21373E-13	22
Gal3st1	1,83304E-17	2,2396405	0,377	0,085	2,94149E-13	22
Tax1bp3	2,1877E-17	1,48841516	0,841	0,412	3,51061E-13	22
Fth1	3,02354E-17	0,777941867	1	0,994	4,85188E-13	22
Wnt6	3,23946E-17	2,335975465	0,42	0,106	5,19835E-13	22
Serpinh1	3,85155E-17	1,202888732	0,971	0,564	6,18059E-13	22
Rarres2	6,09841E-17	1,551153241	0,623	0,205	9,78611E-13	22
Deptor	7,27742E-17	2,299159697	0,362	0,083	1,16781E-12	22

Abca8a	1,37543E-16	1,797034953	0,522	0,153	2,20715E-12	22
Scn7a	1,55528E-16	1,170270868	0,652	0,205	2,49575E-12	22
Sat1	1,82857E-16	1,459394495	0,812	0,381	2,9343E-12	22
Gulp1	7,03635E-16	1,492720483	0,667	0,244	1,12912E-11	22
Gjc3	9,11226E-16	1,3177768	0,739	0,277	1,46224E-11	22
Itm2a	9,19558E-16	1,498156822	0,797	0,384	1,47561E-11	22
Tiam1	1,20683E-15	2,506683738	0,261	0,048	1,93661E-11	22
Snta1	2,85684E-15	2,573037403	0,29	0,06	4,58438E-11	22
Aatk	2,97375E-15	2,31097989	0,362	0,093	4,77197E-11	22
Fkbp10	3,44404E-15	1,659692473	0,507	0,146	5,52665E-11	22
Prex2	3,83391E-15	2,025688719	0,348	0,08	6,15227E-11	22
Cd63	3,87644E-15	1,064352878	0,957	0,68	6,22053E-11	22
Dag1	4,7557E-15	1,719826103	0,58	0,21	7,63147E-11	22
Plin3	5,31075E-15	1,702779046	0,565	0,193	8,52216E-11	22
Col6a1	5,81624E-15	1,989978507	0,464	0,139	9,33332E-11	22
Gsn	6,05543E-15	1,124547814	0,87	0,429	9,71715E-11	22
Afap1l2	1,11609E-14	1,638322959	0,652	0,248	1,79099E-10	22
Fkbp9	1,82209E-14	1,673951557	0,609	0,238	2,92392E-10	22
Nek6	3,28594E-14	2,365373134	0,275	0,057	5,27295E-10	22
Tsc22d4	4,1351E-14	1,176649143	0,884	0,462	6,63559E-10	22
Ahnak	9,84104E-14	1,86222061	0,507	0,174	1,57919E-09	22
Col9a3	1,02788E-13	2,603209305	0,319	0,078	1,64944E-09	22
Emp3	1,12929E-13	1,470743586	0,696	0,288	1,81217E-09	22
Gpx8	1,29181E-13	1,371822407	0,696	0,268	2,07296E-09	22
Apoe	1,97997E-13	0,583946116	1	0,618	3,17725E-09	22
Angptl4	2,7612E-13	2,321715782	0,275	0,06	4,4309E-09	22
Cryab	3,87463E-13	0,810943575	1	0,815	6,21762E-09	22
Sema3c	4,13266E-13	1,520064495	0,536	0,184	6,63169E-09	22
Slc17a6	0	8,497812165	0,778	0,004	0	23
Calb1	0	8,559940615	0,778	0,005	0	23
Nxph2	0	10,63624086	0,722	0,001	0	23
Npy5r	0	9,834828876	0,556	0,001	0	23
Ano5	0	10,53480612	0,333	0	0	23
Npy1r	6,6259E-178	6,352088253	0,778	0,011	1,0633E-173	23
Pcp4l1	2,6586E-169	8,634637236	0,889	0,016	4,2662E-165	23
Skap1	2,9417E-153	6,340343308	0,667	0,009	4,7205E-149	23
Ush1c	2,5934E-139	6,51648139	0,611	0,009	4,1616E-135	23
Calca	2,555E-130	6,910085905	0,5	0,006	4,1E-126	23
Tnr	4,292E-123	6,426866371	0,556	0,008	6,8873E-119	23
Slc16a12	8,2385E-119	6,99528791	0,333	0,003	1,322E-114	23
Islr2	9,6487E-116	5,700868689	1	0,031	1,5483E-111	23
Irf5	1,3448E-113	6,898885318	0,333	0,003	2,1581E-109	23
Pbx3	2,7272E-100	5,091188916	0,944	0,032	4,37639E-96	23
Nefm	1,54195E-90	6,320140338	0,833	0,028	2,47437E-86	23
Ccdc85a	9,20944E-82	5,998919523	0,444	0,008	1,47784E-77	23
Nefl	1,241E-74	5,932345666	0,944	0,046	1,99143E-70	23
Acta1	8,13648E-72	4,987465704	0,667	0,022	1,30566E-67	23
Cckar	4,99535E-70	7,501593789	0,389	0,007	8,01603E-66	23

Klhl1	6,1308E-68	4,946610471	0,556	0,016	9,8381E-64	23
Vstm2a	8,50487E-65	6,323321641	0,667	0,025	1,36478E-60	23
Gna14	1,20225E-57	5,780839837	0,444	0,012	1,92925E-53	23
Ntng1	1,16703E-52	5,009974172	1	0,079	1,87273E-48	23
Gmpr	5,64541E-47	5,571888478	1	0,092	9,05919E-43	23
Rasgrf2	3,04408E-45	4,986729799	0,389	0,012	4,88483E-41	23
Kctd12	1,79711E-43	4,783624491	0,944	0,085	2,88383E-39	23
Aard	3,3628E-43	6,097849351	0,333	0,009	5,39628E-39	23
Calcb	1,19292E-41	6,122496364	0,667	0,041	1,91428E-37	23
Nrsn2	1,42929E-41	4,431742155	0,889	0,075	2,29358E-37	23
Atp1a3	7,47927E-41	4,064866062	0,722	0,047	1,2002E-36	23
Slitrk1	8,14303E-41	4,221405663	0,556	0,027	1,30671E-36	23
Fam122b	2,45446E-40	5,780189234	0,444	0,018	3,93868E-36	23
Rab27b	4,41473E-40	5,05234005	0,667	0,042	7,08431E-36	23
Stra6	1,0505E-38	5,604389126	0,278	0,007	1,68573E-34	23
Adcyap1	3,23404E-38	5,190593446	0,444	0,019	5,18966E-34	23
Sema5a	1,43985E-37	5,091446083	0,444	0,019	2,31053E-33	23
Tmeff2	1,87993E-36	4,433525398	0,889	0,087	3,01672E-32	23
Nrp2	1,51311E-35	4,515116445	0,889	0,09	2,42809E-31	23
Pdyn	2,45848E-35	5,239631099	0,333	0,011	3,94512E-31	23
Chst8	4,78348E-35	4,054454377	0,778	0,064	7,67605E-31	23
March1	9,51076E-35	4,333251258	0,389	0,015	1,52619E-30	23
Prune2	3,25195E-34	4,0225613	0,556	0,032	5,2184E-30	23
Cpne8	4,90133E-33	3,905476111	0,944	0,109	7,86517E-29	23
Adra2a	8,19085E-33	3,941523619	0,556	0,034	1,31439E-28	23
Kif26b	1,30553E-32	4,161311397	0,444	0,021	2,09498E-28	23
Mt3	1,51075E-32	5,384428587	1	0,139	2,4243E-28	23
Rit2	5,19822E-31	3,812284921	0,889	0,099	8,34158E-27	23
Sntg1	2,86936E-30	5,028962712	0,333	0,013	4,60446E-26	23
Id4	3,62125E-30	4,079697973	0,944	0,128	5,81102E-26	23
Nrip3	1,49307E-29	4,314275405	0,444	0,024	2,39593E-25	23
Cpne7	2,32213E-29	4,15481971	0,444	0,024	3,72633E-25	23
Tmem35	2,67815E-29	4,29374113	0,778	0,082	4,29763E-25	23
Tmc3	3,17515E-29	4,251136818	0,611	0,047	5,09517E-25	23
Kcnv1	4,29276E-29	4,800343641	0,5	0,032	6,88859E-25	23
Gm8773	6,29405E-29	3,997558421	0,556	0,039	1,01001E-24	23
Lrfn5	1,76996E-28	3,706682033	0,611	0,048	2,84025E-24	23
Akap7	2,05445E-28	5,157406986	0,778	0,088	3,29678E-24	23
Gria1	6,02296E-28	4,23527801	0,444	0,026	9,66505E-24	23
Grp	7,33875E-28	6,160483886	0,389	0,02	1,17765E-23	23
Gpx3	8,89451E-28	4,602599911	1	0,163	1,4273E-23	23
A330102I10Rik	1,36929E-27	3,95364362	0,444	0,026	2,1973E-23	23
Serpini1	1,42404E-27	4,123713893	1	0,162	2,28516E-23	23
Hpcal4	1,41911E-26	3,680000959	0,556	0,042	2,27725E-22	23
Lrat	1,05471E-25	4,385552435	0,278	0,011	1,69249E-21	23
Ntrk3	1,52434E-25	2,781692647	0,778	0,082	2,44611E-21	23
Parm1	2,70728E-25	3,321465206	0,833	0,104	4,34437E-21	23
Frmpd4	4,33947E-25	4,403522297	0,333	0,016	6,96355E-21	23

Bdnf	1,61808E-24	4,831889769	0,333	0,017	2,59653E-20	23
Ccdc184	5,23731E-23	3,079164887	0,833	0,111	8,40432E-19	23
Kcnb2	1,10177E-22	3,664676559	0,778	0,109	1,76801E-18	23
A730017C20Rik	1,31867E-22	3,392844971	0,944	0,171	2,11608E-18	23
Ngb	1,87385E-22	3,474352097	0,722	0,084	3,00697E-18	23
Ebf1	4,86489E-22	4,656992321	0,556	0,054	7,80669E-18	23
Bace2	1,34931E-21	4,454208128	0,278	0,013	2,16523E-17	23
Stk32c	1,4296E-21	3,203157733	0,667	0,075	2,29408E-17	23
Fam19a2	2,43929E-21	4,374717482	0,389	0,026	3,91433E-17	23
Pgm2l1	5,03023E-21	3,209723244	0,889	0,152	8,07201E-17	23
Ddah1	1,19949E-20	3,427415512	0,944	0,194	1,92482E-16	23
Cartpt	1,59658E-20	1,981287011	0,944	0,161	2,56203E-16	23
Scrn1	3,17529E-20	3,044332728	0,833	0,129	5,0954E-16	23
Hspb1	5,72977E-20	3,158115792	0,778	0,113	9,19457E-16	23
Reps2	6,1434E-20	4,091822455	0,333	0,02	9,85832E-16	23
9530059O14Rik	6,43719E-20	2,760883231	0,778	0,106	1,03298E-15	23
Cntn5	6,81646E-20	4,021382182	0,333	0,021	1,09384E-15	23
Htr2b	6,91576E-20	4,305065659	0,333	0,021	1,10977E-15	23
Atp7a	1,89659E-19	3,857222806	0,333	0,021	3,04347E-15	23
Tmem59l	6,05042E-19	3,124629154	0,778	0,124	9,70911E-15	23
Sez6l	7,01574E-19	3,848488272	0,444	0,037	1,12582E-14	23
Caly	1,19201E-18	3,103734238	1	0,264	1,91283E-14	23
Pcp4	1,20276E-18	2,496930808	0,556	0,059	1,93007E-14	23
Ltk	3,10706E-18	3,505531304	0,444	0,04	4,9859E-14	23
Tmem130	3,48017E-18	3,185050022	0,667	0,09	5,58463E-14	23
Snhg11	6,43785E-18	3,081014228	1	0,269	1,03308E-13	23
Slc35g2	6,49022E-18	3,507477309	0,556	0,063	1,04149E-13	23
Cask	8,578E-18	3,034133598	0,556	0,058	1,37651E-13	23
Lin7a	1,05504E-17	3,20807464	0,556	0,06	1,69303E-13	23
Resp18	1,25093E-17	3,074254389	1	0,264	2,00737E-13	23
Pclo	1,66279E-17	3,776293838	0,667	0,098	2,66828E-13	23
Fam155a	2,84247E-17	2,834779259	0,722	0,105	4,56131E-13	23
Sncg	4,4872E-17	3,545743924	1	0,328	7,20061E-13	23
