# Supplementary material for: The transcriptomes, connections and development of submucosal neuron classes in the mouse small intestine
Source: Nat Neurosci. 2025 May 29;28(6):1146–59. doi: 10.1038/s41593-025-01962-x (PMC12148937; doi:10.1038/s41593-025-01962-x)
Supplement: Supplementary file 12 — Primary antibodies, secondary antibodies and AAV virus used in the study. [file 41593_2025_1962_MOESM12_ESM.pdf]

SUPPLEMENTARY TABLE 5

a) Primary Antibodies used in the study:

Target	Host	Dilution	Source	Comment:
5-HT	Goat	1:500	Abcam ab66047	
CALB	Mouse	1:500	Swant CB300	IgG1
CALR	Mouse	1:300	Santa Cruz sc-365956	IgG2b
CD31	Goat	1:500	Novus Bio AF3628	
ENK	Mouse	1:300	Abcam ab150346	IgG1
GFP	Chicken	1:1000	Abcam ab13970	
HuC/D	Mouse	1:300	Molecular Probes A21271	IgG2b
HuC/D	Rabbit	1:300	Abcam ab184267	
LYVE-1	Rat	1:400	Novus Bio MAB2125	
DCLK1	Rabbit	1:400	Abcam ab31704	
NDUFA4L2	Rabbit	1:200	Proteintech 16480-1-AP	
NF-M	Mouse	1:500	Abcam ab7794	IgG2a
NOS1	Goat	1:1000	Abcam ab1376	
NOS1	Rabbit	1:300	Santa Cruz sc-648	
NTNG1	Rabbit	1:200	Abcam ab221456	
PAIP2B	Rabbit	1:200	Invitrogen PA5-61323	
PGP9.5	Rabbit	1:500-1000	Invitrogen PA5-29012	
PGP9.5	Mouse	1:300	Novus Bio NB600-1160	IgG1
PSD95 (nanobody)	Camel	1:300	N3702-AB635P-L	FluoTag®-X2 anti-PSD95AbberiorStar635P
RFP/Tomato	Rat	1:1000	Chromotek (Proteintech), 5F8	
SOM	Rat	1:100	Merck MAB354	
SOX10	Goat	1:2000	R&D Systems AF2864	
SYN1	Rabbit	1:100	Abcam ab64581	
tdTomato	Goat	1:200	Antibodies.com A121690	
TH	Sheep	1:300	Novus Bio NB300-110	
VIP	Rabbit	1:1000	Immunostar 20077	

b) Secondary Antibodies used in the study:

Antibodies (current study):	Source:	Dilution:
Donkey anti-chicken 488	Jackson ImmunoResearch 703-545-155	1:400
Donkey anti-goat 488	ThermoFisher A11055	1:400
Donkey anti-goat 555	ThermoFisher A21432	1:1000
Donkey anti-goat 647	ThermoFisher A21447	1:250
Goat anti-mouse IgG1 488	ThermoFisher A21121	1:400

Goat anti-mouse IgG1 555	ThermoFisher A21127	1:1000
Goat anti-mouse IgG1 647	ThermoFisher A21240	1:250
Goat anti-mouse IgG2a 488	ThermoFisher A21131	1:400
Goat anti-mouse IgG2b 555	ThermoFisher A21147	1:1000
Donkey anti-rat 488	ThermoFisher A21209	1:400
Donkey anti-rat 594	ThermoFisher A21208	1:1000
Donkey anti-rabbit 404	ThermoFisher A48258	1:200
Donkey anti-rabbit 488	ThermoFisher A32790	1:400
Donkey anti-rabbit 555	ThermoFisher A31572	1:1000
Donkey anti-rabbit 647	ThermoFisher A31573	1:250
Donkey anti-sheep 647	ThermoFisher A21488	1:250

c) Virus used in the study:

**name**	**Expression Cassette**	**Mouse line**	**Final dose (viral genomes)**
v309-PHP.S	shortCAG-dlox-EYFP(rev)-dlox-WPRE-hGHp(A)	*Nmu-Cre*	1.1-2.3*10^12
		Sst-IRES-Cre	1.15-2.3*10^11
		Vip-IRES-Cre	8*10^10 - 1.15*10^11
		Cck-IRES-Cre	5.5*10^11
v167-PHP.S	shortCAG-dlox-tdtomato(rev)-dlox-WPRE-hGHp(A)	*Nmu-Cre*	3*10^11
		Cck-IRES-Cre	7*10^11
		Sst-IRES-Cre	7*10^11
		Vip-IRES-Cre	7*10^11
VSA3(ZH743)-PHP.S	shortCAG-dlox-rSyp1_EGFP(rev)-dlox-WPRE-hGHp(A)	*Nmu-Cre*	1.0*10^12
		Sst-IRES-Cre	1.0*10^12
		Vip-IRES-Cre	1.0*10^12
